# Human Leukocyte Antigen Susceptibility Map for Severe Acute Respiratory Syndrome Coronavirus 2

**DOI:** 10.1128/JVI.00510-20

**Published:** 2020-06-16

**Authors:** Austin Nguyen, Julianne K. David, Sean K. Maden, Mary A. Wood, Benjamin R. Weeder, Abhinav Nellore, Reid F. Thompson

**Affiliations:** a Computational Biology Program, Oregon Health & Science University, Portland, Oregon, USA; b Department of Biomedical Engineering, Oregon Health & Science University, Portland, Oregon, USA; c Portland VA Research Foundation, Portland, Oregon, USA; d Department of Surgery, Oregon Health & Science University, Portland, Oregon, USA; e Department of Radiation Medicine, Oregon Health & Science University, Portland, Oregon, USA; f Department of Medical Informatics and Clinical Epidemiology, Oregon Health & Science University, Portland, Oregon, USA; g Division of Hospital and Specialty Medicine, VA Portland Healthcare System, Portland, Oregon, USA; Loyola University Chicago

**Keywords:** COVID-19, HLA, MHC class I, SARS-CoV-2, coronavirus

## Abstract

Genetic variability across the three major histocompatibility complex (MHC) class I genes (human leukocyte antigen A [HLA-A], -B, and -C genes) may affect susceptibility to and severity of the disease caused by severe acute respiratory syndrome coronavirus 2 (SARS-CoV-2), the virus responsible for coronavirus disease 2019 (COVID-19). We performed a comprehensive *in silico* analysis of viral peptide-MHC class I binding affinity across 145 HLA-A, -B, and -C genotypes for all SARS-CoV-2 peptides. We further explored the potential for cross-protective immunity conferred by prior exposure to four common human coronaviruses. The SARS-CoV-2 proteome was successfully sampled and was represented by a diversity of HLA alleles. However, we found that HLA-B*46:01 had the fewest predicted binding peptides for SARS-CoV-2, suggesting that individuals with this allele may be particularly vulnerable to COVID-19, as they were previously shown to be for SARS (M. Lin, H.-T. Tseng, J. A. Trejaut, H.-L. Lee, et al., BMC Med Genet 4:9, 2003, https://bmcmedgenet.biomedcentral.com/articles/10.1186/1471-2350-4-9). Conversely, we found that HLA-B*15:03 showed the greatest capacity to present highly conserved SARS-CoV-2 peptides that are shared among common human coronaviruses, suggesting that it could enable cross-protective T-cell-based immunity. Finally, we reported global distributions of HLA types with potential epidemiological ramifications in the setting of the current pandemic.

**IMPORTANCE** Individual genetic variation may help to explain different immune responses to a virus across a population. In particular, understanding how variation in HLA may affect the course of COVID-19 could help identify individuals at higher risk from the disease. HLA typing can be fast and inexpensive. Pairing HLA typing with COVID-19 testing where feasible could improve assessment of severity of viral disease in the population. Following the development of a vaccine against SARS-CoV-2, the virus that causes COVID-19, individuals with high-risk HLA types could be prioritized for vaccination.

## Abstract (precis)

Individual genetic variation may help to explain different immune responses to a virus across a population. In particular, understanding how variation in HLA may affect the course of COVID-19 could help identify individuals at higher risk from the disease. HLA typing can be fast and inexpensive. Pairing HLA typing with COVID-19 testing where feasible could improve assessment of severity of viral disease in the population. Following the development of a vaccine against SARS-CoV-2, the virus that causes COVID-19, individuals with high-risk HLA types could be prioritized for vaccination.

## INTRODUCTION

Recently, a new strain of betacoronavirus (severe acute respiratory syndrome coronavirus 2, or SARS-CoV-2) emerged as a global pathogen, prompting the World Health Organization in January 2020 to declare an international public health emergency (1). In the large coronavirus family, comprising enveloped positive-strand RNA viruses, SARS-CoV-2 is the seventh encountered strain that causes respiratory disease in humans (2) ranging from mild—the common cold—to severe—disease caused by the zoonotic Middle East respiratory syndrome coronavirus (MERS-CoV) and severe acute respiratory syndrome coronavirus (SARS-CoV). As of April 2020, there are over one million presumed or confirmed cases of coronavirus disease 19 (COVID-19) worldwide, with total deaths exceeding 50,000 (3). While age and many comorbidities, including cardiovascular and pulmonary disease, appear to increase the severity and mortality of COVID-19 (4–9), approximately 80% of infected individuals have mild symptoms (10). As with SARS-CoV (11, 12) and MERS-CoV (13, 14), children seem to have low susceptibility to the disease (15–17); despite infection rates similar to those seen with adults (18), only 5.9% of pediatric cases are severe or critical, possibly due to lower binding ability of the ACE2 receptor in children or generally higher levels of antiviral antibodies (19). Other similarities (20–22), including genomic (23, 24) and immune system response (25–33) similarities, between SARS-CoV-2 and other coronaviruses (34), especially SARS-CoV and MERS-CoV, are topics of ongoing active research, results of which may inform an understanding of the severity of infection (35) and improve the ongoing work of immune landscape profiling (36–40) and vaccine discovery (28, 37, 41–48).

Human leukocyte antigen (HLA) alleles, which are critical components of the viral antigen presentation pathway, have been shown in previous studies to confer differential viral susceptibility and severity of disease. For instance, disease caused by the closely related SARS-CoV (23, 24) shows increased severity among individuals with the HLA-B*46:01 genotype (49). Associations between HLA genotype and disease severity extend broadly to several other unrelated viruses. For example, in human immunodeficiency virus 1 (HIV-1), certain HLA types (e.g., HLA-A*02:05) may reduce risk of seroconversion (50), and in dengue virus, certain HLA alleles (e.g., HLA-A*02:07 and HLA-B*51) are associated with increased secondary disease severity among ethnic Thais (51).

While the details of the clinical picture of the COVID-19 pandemic continue to emerge, there remain substantial unanswered questions regarding the role of individual genetic variability in the immune response against SARS-CoV-2 (51). We hypothesize that individual HLA genotypes may differentially induce the T-cell mediated antiviral response and could potentially alter the course of disease and its transmission. In this study, we performed a comprehensive *in silico* analysis of viral peptide-major histocompatibility complex (MHC) class I binding affinity across 145 different HLA types for the entire SARS-CoV-2 proteome.

## RESULTS

**FIG 1:**
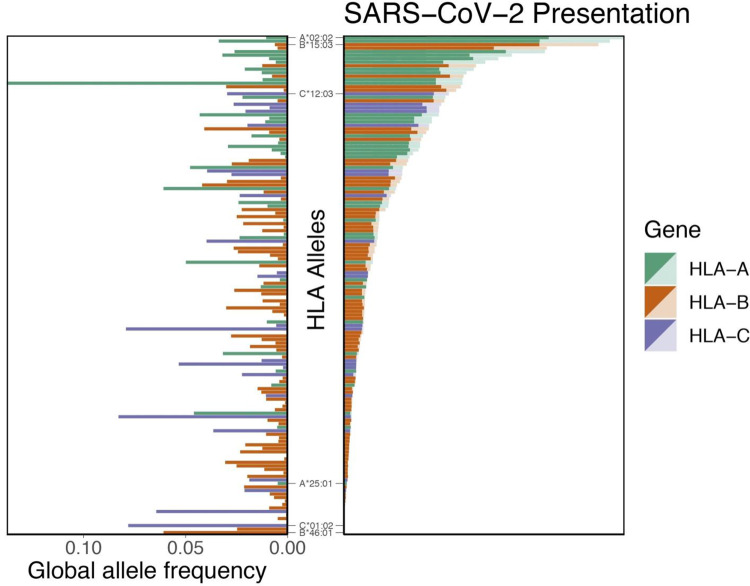
Distribution of HLA allelic presentation of 8- to 12-mers from the SARS-CoV-2 proteome. At right, the number of peptides (see Table S1) that putatively bind to each of 145 HLA alleles is shown as a series of horizontal bars, with dark and light shading indicating the number of tightly (<50 nM) and loosely (<500 nM) binding peptides, respectively, and with green, orange, and purple representing HLA-A, -B, and -C alleles, respectively. Alleles are sorted in descending order based on the number of peptides that they bind (<500 nM). The corresponding estimated allelic frequency in the global population is also shown (left), with the length of each horizontal bar indicating absolute frequency in the population.

To explore the potential for a given HLA allele to produce an antiviral response, we assessed the HLA binding affinity of all possible 8-mers to 12-mers from the SARS-CoV-2 proteome (*n* = 48,395 unique peptides). We then removed from further consideration 16,138 peptides that were not predicted to enter the MHC class I antigen processing pathway via proteasomal cleavage. For the remaining 32,257 peptides, we repeated binding affinity predictions for a total of 145 different HLA types, and we show here the SARS-CoV-2-specific distribution of per-allele proteome presentation (predicted binding affinity threshold of <500 nM) (Fig. 1; see also Table S1 in the supplemental material). Importantly, we note that the putative capacity for SARS-CoV-2 antigen presentation is unrelated to the HLA allelic frequency in the population (Fig. 1). We identify HLA-B*46:01 as the HLA allele with the fewest predicted binding peptides for SARS-CoV-2. We performed the same analyses for the closely related SARS-CoV proteome (see Fig. S1 in the supplemental material) and similarly note that HLA-B*46:01 was predicted to present the fewest SARS-CoV peptides, in keeping with previous clinical data associating this allele with severe disease (49).

**FIG 2:**
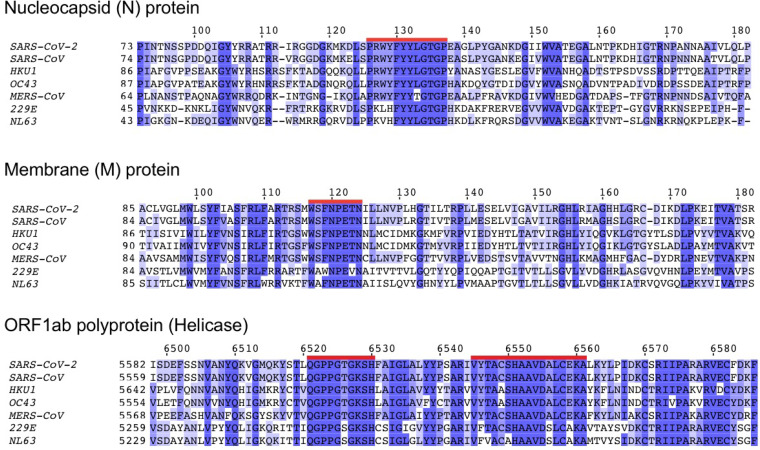
Amino acid sequence conservation of four linear peptide example sequences from three human coronavirus proteins. Protein sequence alignments are shown for nucleocapsid (N), membrane (M), and ORF1ab polyprotein (helicase) across all five known human betacoronaviruses (SARS-CoV-2, SARS-CoV, HKU1, OC43, and MERS-CoV) and two known human alphacoronaviruses (229E and NL63). Each row in the three depicted sequence alignments corresponds to the protein sequence from the indicated coronavirus, with the starting coordinate of the viral protein sequence shown at left and position coordinates of the overall alignment displayed above. Blue shading indicates the extent of sequence identity, with the darkest blue shading indicating a 100% match for that amino acid across all sequences. The four red-highlighted sequences correspond to highly conserved peptides ≥8 amino acids in length (PRWYFYYLGTGP, WSFNPETN, QPPGTGKSH, and VYTACSHAAVDALCEKA, see Table S2).

To assess the potential for cross-protective immunity conferred by prior exposure to common human coronaviruses (i.e., HKU1, OC43, NL63, and 229E), we next sought to characterize the conservation of the SARS-CoV-2 proteome across diverse coronavirus subgenera to identify highly conserved linear epitopes. After aligning reference proteome sequence data for 5 essential viral components (ORF1ab, S, E, M, and N proteins) across 34 distinct alpha- and betacoronaviruses, including all known human coronaviruses, we identified 48 highly conserved amino acid sequence spans (see Data File S1 in the supplemental material). Acknowledging the challenges to inferring cross-protective immunity among closely related peptides, we confined our attention exclusively to identical peptide matches. Among the conserved sequences, 44 SARS-CoV-2 sequences would each be anticipated to generate at least one 8- to 12-mer linear peptide epitope also present within at least one other common human coronavirus (Fig. 2; see also Table S2). In total, 564 such 8- to 12-mer peptides were found to share 100% identity with corresponding OC43, HKU1, NL63, and 229E sequences (467, 460, 179, and 157 peptides, respectively) (Table S3).

**FIG 3:**
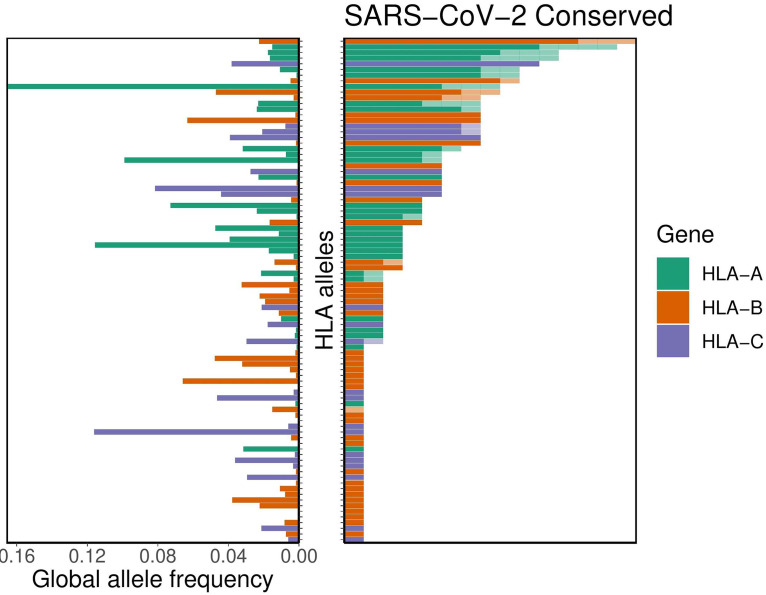
Distribution of HLA allelic presentations of highly conserved human coronavirus peptides with potential to elicit cross-protective immunity to COVID-19. At right, the number of conserved peptides (see Table S3) that putatively bind to a subset of 89 HLA alleles is shown as a series of horizontal bars, with dark and light shading indicating the number of tightly (<50 nM) and loosely (<500 nM) binding peptides, respectively, and with green, orange, and purple representing HLA-A, -B, and -C alleles, respectively. Alleles are sorted in descending order based on the number of peptides they are anticipated to present (binding affinity, <500 nM). The corresponding allelic frequency in the global population is also shown (left), with the length of each horizontal bar indicating absolute frequency in the population.

For the subset of these potentially cross-protective peptides that are anticipated to be generated via the MHC class I antigen processing pathway, we performed binding affinity predictions across 145 different HLA-A, -B, and -C alleles (see Data File S3). As described above, we demonstrated the SARS-CoV-2-specific distribution of per-allele presentation for these conserved peptides. We found that alleles HLA-A*02:02, HLA-B*15:03, and HLA-C*12:03 were the top presenters of conserved peptides. Conversely, we note that 56 different HLA alleles demonstrated no appreciable binding affinity (<500 nM) to any of the conserved SARS-CoV-2 peptides, suggesting a concomitant lack of potential for cross-protective immunity from other human coronaviruses. We note, in particular, that HLA-B*46:01 was among these alleles. We note also that the putative capacity for conserved peptide presentation is unrelated to the HLA allelic frequency in the population (Fig. 3). Moreover, we see no appreciable global correlation between conservation of the SARS-CoV-2 proteome and its predicted MHC binding affinity, suggesting a lack of selective pressure for or against the capacity to present coronavirus epitopes (*P* = 0.27 [Fisher’s exact test]; see Fig. S2).

**FIG 4:**
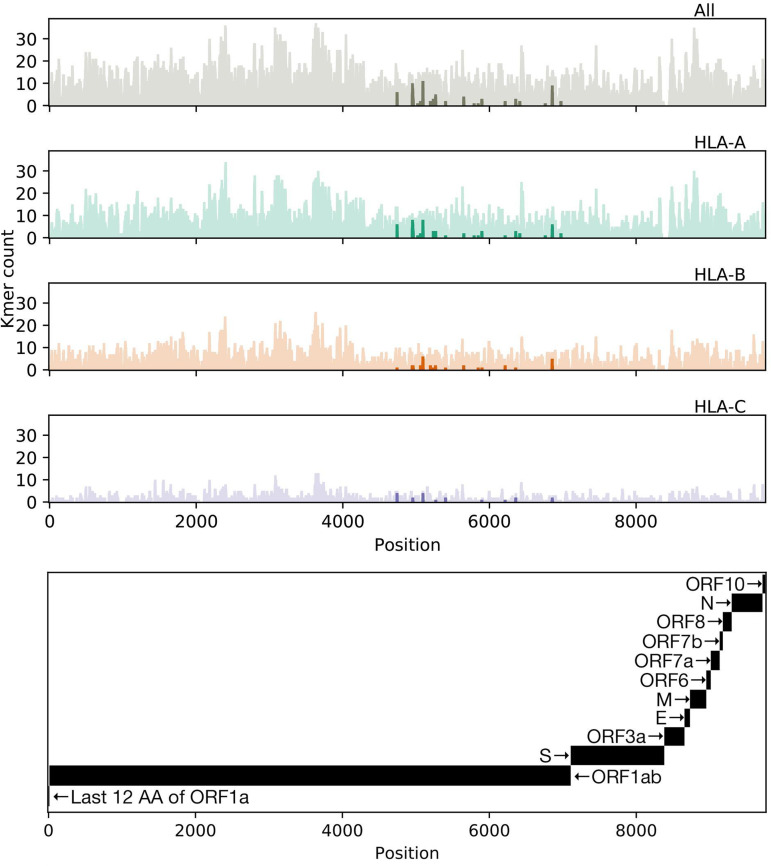
Distribution of allelic presentation of conserved 8- to 12-mers across the entire SARS-CoV-2 proteome for all HLA alleles and individually for HLA-A, HLA-B, and HLA-C (first, second, third, and fourth plots from top, respectively) with dark and light shading indicating the number of tightly (<50 nM) and loosely (<500 nM) binding peptides, respectively. Positions are derived from a concatenation of coding sequences (CDSs) as indicated in the bottom panel. Tightly binding peptides are confined to ORF1ab. The sequence begins with only the last 12 amino acids of ORF1a because all but the last four amino acids of ORF1a are contained in ORF1ab, and we considered binding peptides up to 12 amino acids (AA) in length.

We were further interested in whether certain regions of the SARS-CoV-2 proteome showed differential presentation by the MHC class I pathway. Accordingly, we surveyed the distribution of antigen presentation capacity across the entire proteome, highlighting its most conserved peptide sequences (Fig. 4). Throughout the entire proteome, HLA-A and HLA-C alleles exhibited the relatively largest and smallest capacities to present SARS-CoV-2 antigens, respectively. However, each of the three major class I genes exhibited very similar patterns of peptide presentation across the proteome (Fig. S3). We additionally note that peptide presentation appears to be independent of estimated time of peptide production during viral life cycle, with indistinguishable levels of peptide presentation of both early and late SARS-CoV-2 peptides (Fig. S4).

**FIG 5:**
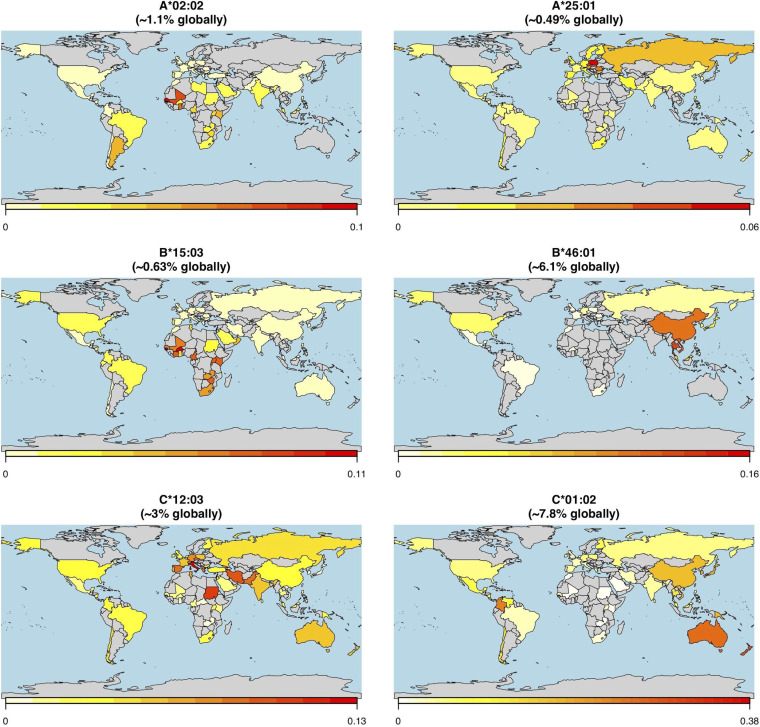
Global HLA allele frequency distribution heat maps for six HLA-A, -B, and -C alleles. The leftmost panels show the global allele frequency distributions by country for three representative alleles (HLA-A*02:02, HLA-B*15:03, and HLA-C*12:03) with the predicted capacities to present the greatest repertoire of epitopes from the SARS-CoV-2 proteome (21.1%, 19.1%, and 7.9% of presentable epitopes, respectively). The rightmost panels show the global allele frequency distributions by country for three representative alleles (HLA-A*25:01, HLA-B*46:01, and HLA-C*01:02) with the lowest predicted levels epitope presentation from the SARS-CoV-2 proteome (0.2%, 0%, and 0% of presentable epitopes, respectively). Heat map coloring corresponds to the individual HLA allele frequency within each country, ranging from lowest (white/yellow) to highest (red) frequency as indicated in the legend below each map.

Given the global nature of the current COVID-19 pandemic, we sought to describe population-level distributions of the HLA alleles most (and least) capable of generating a repertoire of SARS-CoV-2 epitopes in support of a T-cell-based immune response. While we present global maps of individual HLA allele frequencies for the full set of 145 different alleles studied here (Data File S2), we specifically highlight the global distributions of the three best-presenting (A*02:02, B*15:03, and C*12:03) and three of the worst-presenting (A*25:01, B*46:01, and C*01:02) HLA-A, -B, and -C alleles (Fig. 5). Note that all allelic frequencies are aggregated by country but that they implicitly reflect the distribution of HLA data available on the Allele Frequency Net Database (52).

**FIG 6:**
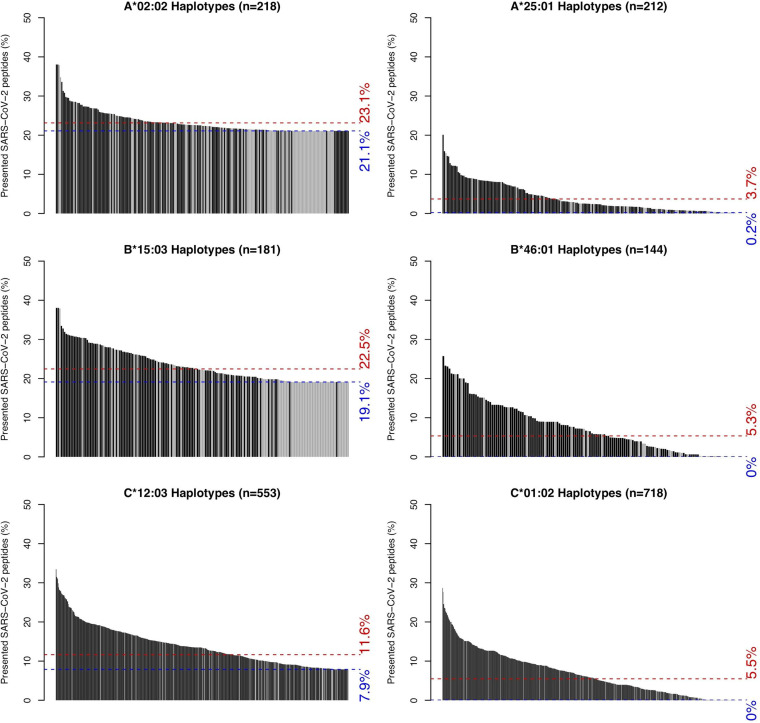
Distributions of SARS-CoV-2 peptide presentation across HLA haplotypes. The leftmost panels show the distributions of SARS-CoV-2 peptide presentation capacity for haplotypes containing one of three representative HLA alleles (HLA-A*02:02, HLA-B*15:03, and HLA-C*12:03) with the greatest predicted repertoire of epitopes from the SARS-CoV-2 proteome. The rightmost panels show the distributions of SARS-CoV-2 peptide presentation capacity for haplotypes containing one of three representative alleles (HLA-A*25:01, HLA-B*46:01, and HLA-C*01:02) with the lowest predicted levels of epitope presentation from the SARS-CoV-2 proteome. Black and gray bars represent full and partial haplotypes, respectively. Blue and red dashed lines represent the percentages of presented SARS-CoV-2 peptides for the indicated allele itself (blue) and its global population frequency weighted average presentation across its observed haplotypes (red).

Finally, we acknowledge that nearly all individuals have two HLA-A/B/C haplotypes constituting as few as three but as many as six distinct alleles, potentially buffering against the lack of presentation from a single poorly presenting allele. We sought to determine whether allele-specific variability in SARS-CoV-2 presentation extends to full HLA haplotypes and to whole individual HLA genotypes. For six representative alleles with the highest (HLA-A*02:02, HLA-B*15:03, and HLA-C*12:03) and lowest (HLA-A*25:01, HLA-B*46:01, and HLA-C*01:02) predicted capacity for SARS-CoV-2 epitope presentation, these differences remain significant at the haplotype level, albeit with wide variability in presentation among different haplotypes (Fig. 6). Haplotype-level data for all 145 alleles are included in Fig. S5 and Data File S2. We then identified 3,382 individuals with full HLA genotype data and noted wide variability in their capacity to present peptides from the SARS-CoV-2 proteome, albeit with a small minority of individuals at either extreme (Fig. S6).

## DISCUSSION

To the best of our knowledge, this was the first study to evaluate per-allele viral proteome presentation across a wide range of HLA alleles using MHC-peptide binding affinity predictors. This report also introduces the relationship between coronavirus sequence conservation and MHC class I antigen presentation. We show that individual HLA, haplotype, and full-genotype variability likely influence the capacity to respond to SARS-CoV-2 infection, and we note certain alleles in particular (e.g., HLA-B*46:01) that could be associated with more-severe infection, as previously shown with SARS-CoV (49). Indeed, we further compare SARS-CoV and SARS-CoV-2 peptide presentation data and note a high degree of similarity between the two across HLA types. Finally, this is the first report to present global distributions of HLA types and haplotypes with potential epidemiological ramifications in the setting of the current pandemic. We found that, in general, there is no correlation between the HLA allelic frequency in the population and allelic capacity to bind SARS-CoV or SARS-CoV-2 peptides, irrespective of the estimated timing of peptide production during the viral replication cycle. While we are not aware of any studies explicitly reporting the relationship between human coronavirus epitope abundance and immune response, there are vaccinia virus data that suggest that early peptide antigens are more likely to generate CD8^+^ T-cell responses whereas antibody and CD4^+^ T-cell responses are more likely to target later mRNA expression with higher peptide abundance in the virion (53).

We note, however, several limitations to our work. First and foremost, while we note that a few of our binding affinity predictions were borne out in experimentally validated SARS-CoV peptides (see Table S4 in the supplemental material), we acknowledge that ours was a study performed entirely *in silico*. As we are unable to obtain individual-level HLA typing and clinical outcome data for any real-world COVID-19 populations at this time, the data presented are theoretical in nature and are subject to many of the same limitations implicit in the MHC binding affinity prediction tool(s) upon which it is based. As such, we are unable to assess the relative importance of HLA type compared to known disease-modifying risk factors such as age and clinical comorbidities (4–9). We further note that peptide-MHC binding affinity is limited in its utility as a predictor of subsequent T-cell responses (54–56), and we did not study T-cell responses here. As such, we are ill-equipped to explore phenomena such as original antigenic sin (57–59), where prior exposure to a closely related infection(s) might trigger T-cell anergy (60–62) or immunopathogenesis (63) in the setting of a novel infection. We explored only a limited set of 145 well-studied HLA alleles but note that this analysis could be performed across a wider diversity of genotypes (48). Additionally, we did not assess genotypic heterogeneity or *in vivo* evolution of SARS-CoV-2, which could modify the repertoire of viral epitopes presented or could otherwise modulate virulence in an HLA-independent manner (64, 65) (https://nextstrain.org/ncov). We also did not address the potential for individual-level genetic variation in other proteins (e.g., angiotensin converting enzyme 2 [ACE2] or transmembrane serine protease 2 [TMPRSS2], essential host proteins for SARS-CoV-2 priming and cell entry [66]) to modulate the host-pathogen interface.

Unless and until the findings we present here are clinically validated, they should not be employed for any clinical purposes. However, we do at this juncture recommend integrating HLA testing into clinical trials and pairing HLA typing with COVID-19 testing where feasible to more rapidly develop and deploy a predictor(s) of viral severity in the population and, potentially, to tailor future vaccination strategies to genotypically at-risk populations. This approach may have additional implications for the management of a broad array of other viruses.

## MATERIALS AND METHODS

### Sequence retrieval and alignments.

Full polyprotein 1ab (ORF1ab), spike (S) protein, membrane (M) protein, envelope (E) protein, and nucleocapsid (N) protein sequences were obtained for each of 34 distinct but representative alpha and betacoronaviruses from broad genus and subgenus distributions, including all known human coronaviruses (i.e., SARS-CoV, SARS-CoV-2, MERS-CoV, HKU1, OC43, NL63, and 229E). FASTA-formatted protein sequence data (the full accession number list is available in Table S5 in the supplemental material) were retrieved from the National Center of Biotechnology Information (NCBI) (67). For each of the protein classes (i.e., ORF1ab, S, M, E, and N), all 34 coronavirus sequences were aligned using the Clustal Omega v1.2.4 multisequence aligner tool employing the following parameters: sequence type [Protein], output alignment format [clustal_num], dealign [false], mBed-like clustering guide-tree [true], mBed-like clustering iteration [true], number of combined iterations 0, maximum guide tree iterations [-1], and maximum HMM iterations [-1] (68). For the purposes of estimating time of viral peptide production, we classified ORF1a and ORF1b peptides as “early” whereas all other peptides produced by subgenomic mRNAs were classified as “late” (69, 70).

### Conserved peptide assessment.

Aligned sequences were imported into Jalview v. 2.1.1 (71) with automated generation of the following alignment annotations: (i) sequence consensus, calculated as the percentage of the modal residue per column; (ii) sequence conservation (0 to 11), measured as a numerical index reflecting conservation of amino acid physicochemical properties in the alignment; (iii) alignment quality (0 to 1), measured as a normalized sum of BLOSUM62 ratios for all residues at each position; and (iv) occupancy, calculated as the number of aligned residues (not including gaps) for each position. In all cases, sequence conservation was assessed for each of the following three groups: only human-infecting coronavirus sequences (*n* = 7), all betacoronavirus sequences (*n* = 16), and all alpha- and betacoronavirus sequences combined (*n* = 34). Aligned SARS-CoV-2 sequences and all annotations were manually exported for subsequent analysis. Conserved human coronavirus peptides were defined as those with a length of ≥8 consecutive amino acids, each showing agreement with SARS-CoV-2 sequences and ≥4 other human coronavirus sequences with the consensus sequence (Table S2). For each of these conserved peptides, we also assessed the component number of 8- to 12-mers sharing identical amino acid sequence between SARS-CoV-2 and each of the four other common human coronaviruses (i.e., OC43, HKU1, NL63, and 229E) (Table S3). For all peptides, human, beta, and combined conservation scores were obtained using a custom R v.3.6.2 script representing mean sequence conservation (minus gap penalties where relevant) (see https://github.com/pdxgx/covid19).

### Peptide-MHC class I binding affinity predictions.

FASTA-formatted input protein sequences from the entire SARS-CoV-2 and SARS-CoV proteomes were obtained from the NCBI RefSeq database (67) under accession numbers NC_045512.2 and NC_004718.3. We kmerized each of these sequences into 8- to 12-mers to assess MHC class I-peptide binding affinity across the entire proteome. MHC class I binding affinity predictions were performed using 145 different HLA alleles for which global allele frequency data were available as described previously (72) (see Table S5) with netMHCpan v4.0 (73) using the ‘-BA’ option to include binding affinity predictions and the ‘-l’ option to specify peptides 8 to 12 amino acids in length (Table S1). Binding affinity was not predicted for peptides containing the character ‘|’ in their sequences. Additional MHC class I binding affinity predictions were performed on all 66 MHCflurry-supported alleles (–list-supported-alleles; Table S6) using both MHCnuggets 2.3.2 (74) and MHCflurry 1.4.3 (75) (see Tables S7, S8, and S9 and Fig. S7 to S10 in the supplemental material). We further cross-referenced these lists of peptides with existing experimentally validated SARS-CoV epitopes present in the Immune Epitope Database (Table S4) (76). We then performed consensus binding affinity predictions for the 66 supported alleles shared by all three tools by taking the union set of alleles and filtering for peptide-allele pairs matching the union set of alleles. For the SARS-CoV-specific and SARS-CoV-2-specific distributions of per-allele proteome presentation, we exclude all peptide-allele pairs with >500 nM predicted binding. In all cases, we used the netchop v3.0 (77) “C-term” model with a cleavage threshold of 0.1 to further remove any peptides that were not predicted to undergo canonical MHC class I antigen processing via proteasomal cleavage (of the peptide’s C terminus).

### Global HLA allele and haplotype frequencies.

HLA-A, -B, and -C allele and haplotype frequency data were obtained from the Allele Frequency Net Database (52) for 805 distinct populations pertaining to 101 different countries and 2,628 distinct major/minor (4-digit) alleles, corresponding to 20,478 distinct haplotypes (https://github.com/pdxgx/covid19). We also identified full HLA genotype data for 3,382 individuals whose HLA types were confined to the 145 HLA alleles studied here. Population allele and haplotype frequency data were aggregated by country as a mean of all constituent population allele or haplotype frequencies weighted by sample size of the population but not accounting for the representative ethnic demographic size of the population. Global allele frequency maps were generated using the rworldmap v1.3-6 package (78), with total global allele and haplotype frequency estimates calculated as the mean of per-country allele and haplotype frequencies, weighted by each country’s population in 2005.

### Data availability.

Source code is available at https://github.com/pdxgx/covid19 under the Massachusetts Institute of Technology (MIT) license. Data File S4 can be found at https://github.com/pdxgx/covid19/blob/master/supporting_data/Appendix_4.zip.

## Supplementary Material

Supplemental file 1

Supplemental file 2

Supplemental file 3

Supplemental file 4
