# Supplementary material for: Human Leukocyte Antigen Susceptibility Map for Severe Acute Respiratory Syndrome Coronavirus 2
Source: J Virol. 2020 Jun 16;94(13):e00510-20. doi: 10.1128/JVI.00510-20 (PMC7307149; doi:10.1128/JVI.00510-20)
Supplement: Supplemental file 1 [file JVI.00510-20-s0001.pdf]

ORF1ab polyprotein

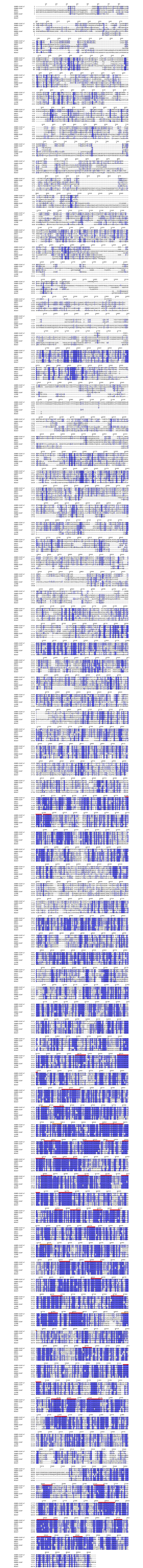

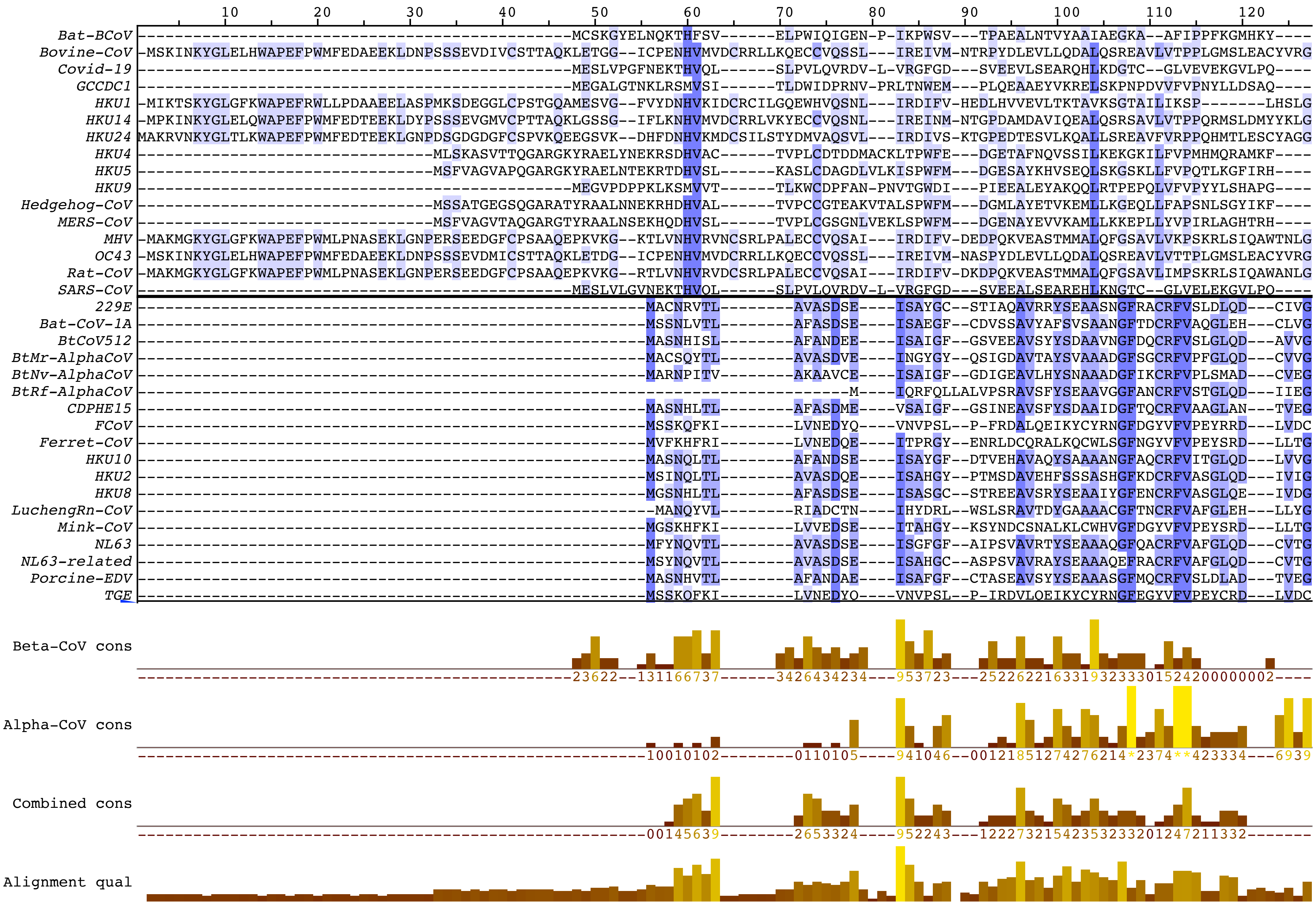
10 20 30 40 50 60 70 80 90 100 110 120
Bat-BCoV
Bovine-CoV
Covid-19
GCCDC1
HKU1
HKU14
HKU24
HKU4
HKU5
HKU9
Hedgehog-CoV
MERS-CoV
MHV
OC43
Rat-CoV
SARS-CoV
229E
Bat-CoV-1A
BtCoV512
BtMr-AlphaCoV
BtNv-AlphaCoV
BtRf-AlphaCoV
CDPHE15
FCoV
Ferret-CoV
HKU10
HKU2
HKU8
LuchengRn-CoV
Mink-CoV
NL63
NL63-related
Porcine-EDV
TGE
Beta-CoV cons
Alpha-CoV cons
Combined cons
Alignment qual

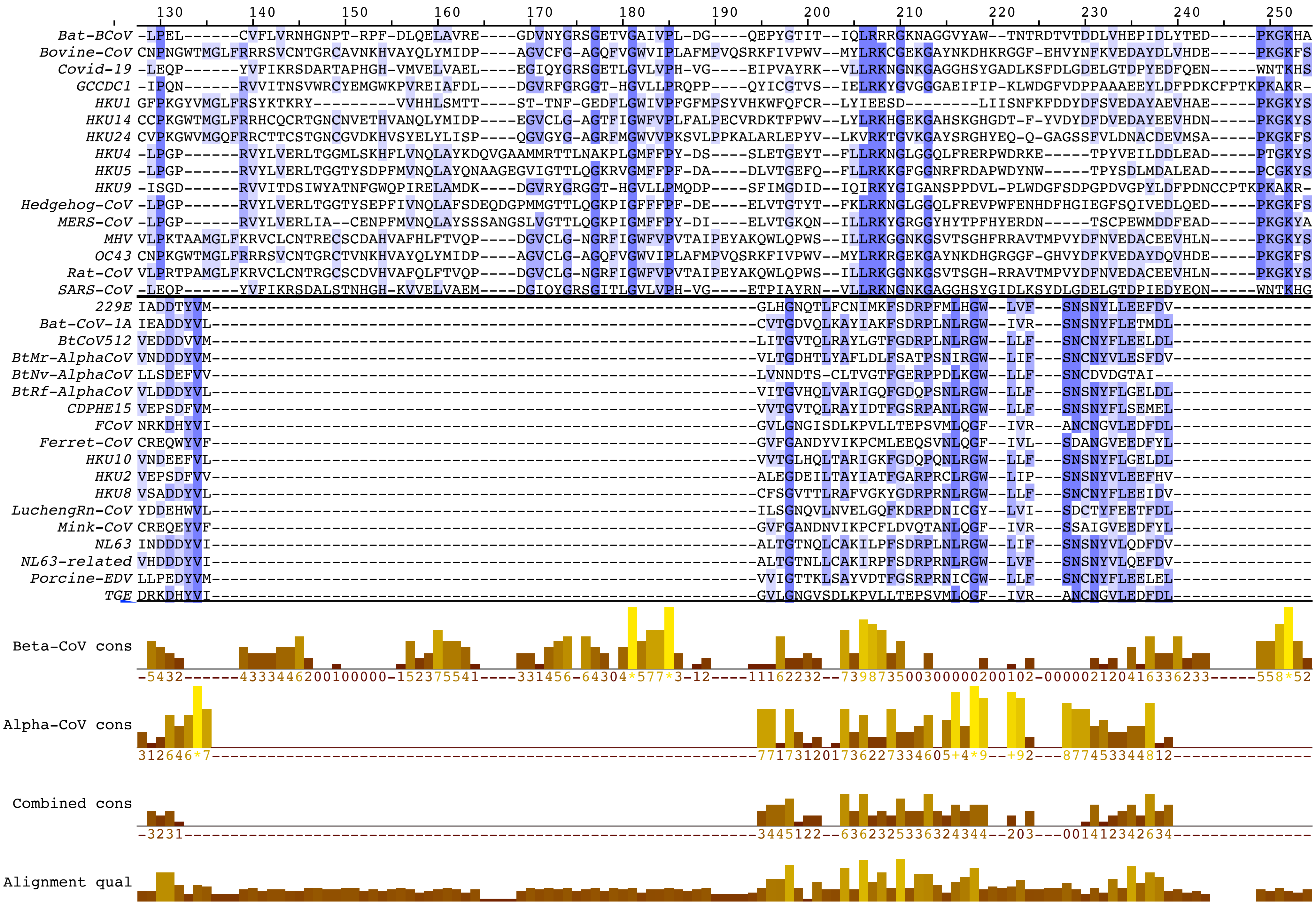

130 140 150 160 170 180 190 200 210 220 230 240 250
Bat-BCoV -LPEL------CVFLVRNHGNPT-RPF-DLQELAVRE----GDVNYGRSGETVGAIVPL-DG----QEPYGTIT--IQLRRRGKNAGGVYAW--TNTRDTVTDDLVHEPIDLYTED-----PKGKHA
Bovine-CoV CNPNGWTMGLFRRRSVCNTGRCAVNKHVAYQLYMIDP----AGVCFG-AGQFVGWVIPLAFMPVQSRKFIVPWV--MYLRKCGEKGAYNKDHKRGGF-EHVYNFKVEDAYDLVHDE-----PKGKFS
Covid-19 -LEQP------YVFIKRSDARTAPHGH-VMVELVAEL----EGIQYGRSGETLGVLVPH-VG----EIPVAYRK--VLLRKNGNKGAGGHSYGADLKSFDLGDELGTDPYEDFQEN-----WNTKHS
GCCDC1 -IPQN------RVVITNSVWRCYEMGWKPVREIAFDL----DGVRFGRGGT-HGVLLPRQPP----QYICGTVS--IELRKYGVGGGAEIFIP-KLWDGFVDPPAAAEEYLDFPDKCFPTKPKAKR-
HKU1 GFPKGYVMGLFRSYKTKRY---------VVHHLSMTT----ST-TNF-GEDFLGWIVPFGFMPSYVHKWFQFCR--LYIEESD---------LIISNFKFDDYDFSVEDAYAEVHAE-----PKGKYS
HKU14 CCPKGWTMGLFRRHCQCRTGNCNVETHVANQLYMIDP----EGVCLG-AGTFIGWFVPLFALPECVRDKTFPWV--LYLRKHGEKGAHSKGHGDT-F-YVDYDFDVEDAYEEVHDN-----PKGKYS
HKU24 CVPKGWVMGQFRRCTTCSTGNCGVDKHVSYELYLISP----QGVGYG-AGRFMGWVVPKSVLPPKALARLEPYV--LKVRKTGVKGAYSRGHYEQ-Q-GAGSSFVLDNACDEVMSA-----PKGKFS
HKU4 -LPGP------RVYLVERLTGGMLSKHFLVNQLAYKDQVGAAMMRTTLNAKPLGMFFPY-DS----SLETGEYT--FLLRKNGLGGQLFRERPWDRKE-----TPYVEILDDLEAD-----PTGKYS
HKU5 -LPGP------RVYLVERLTGGTYSDPFMVNQLAYQNAAGEGVIGTTLQGKRVGMFFPF-DA----DLVTGEFQ--FLLRKKGFGGNRFRDAPWDYNW-----TPYSDLMDALEAD-----PCGKYS
HKU9 -ISGD------RVVITDSIWYATNFGWQPIRELAMDK----DGVRYGRGGT-HGVLLPMQDP----SFIMGDID--IQIRKYGIGANSPPDVL-PLWDGFSDPGPDVGPYLDFPDNCCPTKPKAKR-
Hedgehog-CoV -LPGP------RVYLVERLTGGTYSEPFIVNQLAFSDEQDGPMMGTTLQGKPIGFFFPF-DE----ELVTGTYT--FKLRKNGLGGQLFREVPWFENHDFHGIEGFSQIVEDLQED-----PKGKFS
MERS-CoV -LPGP------RVYLVERLIA--CENPFMVNQLAYSSSANGSLVGTTLQGKPIGMFFPY-DI----ELVTGKQN--ILLRKYGRGGYHYTPFHYERDN-----TSCPEWMDDFEAD-----PKGKYA
MHV VLPKTAAMGLFKRVCLCNTRECSCDAHVAFHLFTVQP----DGVCLG-NGRFIGWFVPVTAIPEYAKQWLQPWS--ILLRKGGNKGSVTSGHFRRAVTMPVYDFNVEDACEEVHLN-----PKGKYS
OC43 CNPKGWTMGLFRRRSVCNTGRCTVNKHVAYQLYMIDP----AGVCLG-AGQFVGWVIPLAFMPVQSRKFIVPWV--MYLRKRGEKGAYNKDHGRGGF-GHVYDFKVEDAYDQVHDE-----PKGKFS
Rat-CoV VLPRTPAMGLFKRVCLCNTRGCSCDVHVAFQLFTVQP----DGVCLG-NGRFIGWFVPVTAIPEYAKQWLQPWS--ILLRKGGNKGSVTSGH-RRAVTMPVYDFNVEDACEEVHLN-----PKGKYS
SARS-CoV -LEQP------YVFIKRSDALSTNHGH-KVVELVAEM----DGIQYGRSGITLGVLVPH-VG----ETPIAYRN--VLLRKNGNKGAGGHSYGIDLKSYDLGDELGTDPIEDYEQN-----WNTKHG
229E IADDTYVM--GLHGNQTLFCNIMKFSDRPFMLHGW--LVF---SNSNYLLEEFDV---------------
Bat-CoV-1A IEADDYVL--CVTGDVQLKAYIAKFSDRPLNLRGW--IVR---SNSNYFLETMDL---------------
BtCoV512 VEDDDVVM--LITGVTQLRAYLGTFGDRPLNLRGW--LLF---SNCNYFLEELDL---------------
BtMr-AlphaCoV VNDDDYVM--VLTGDHTLYAFLDLFSATPSNIRGW--LIF---SNCNYVLESFDV---------------
BtNv-AlphaCoV LLSDEFVV--LVNNDTS-CLTVGTFGERPPDLKGW--LLF---SNCDVDGTAI----------------
BtRf-AlphaCoV VLDDDYVL--VITGVHQLVARIGQFGDQPSNLRGW--LLF---SNSNYFLGELDL---------------
CDPHE15 VEPSDFVM--VVTGVTQLRAYIDTFGSRPANLRGW--LLF---SNSNYFLSEMEL---------------
FCoV NRKDHYVI--GVLGNGISDLKPVLLTEPSVMLQGF--IVR---ANCNGVLEDFDL---------------
Ferret-CoV CREQWYVF--GVFGANDYVIKPCMLEEQSVNLQGF--IVL---SDANGVEEDFYL---------------
HKU10 VNDEEFVL--VVTGLHQLTARIGKFGDQPQNLRGW--LLF---SNSNYFLGELDL---------------
HKU2 VEPSDFVV--ALEGDEILTAYIATFGARPRCLRGW--LIP---SNSNYVLEEFHV---------------
HKU8 VSADDYVL--CFSGVTTLRAFVGKYGDRPRNLRGW--LLF---SNCNYFLEEIDV---------------
LuchengRn-CoV YDDEHWVL--ILSGNQVLNVELGQFKDRPDNICGY--LVI---SDCTYFEETFDL---------------
Mink-CoV CREQEYVF--GVFGANDNVIKPCFLDVQTANLQGF--IVR---SSAIGVEEDFYL---------------
NL63 INDDDYVI--ALTGTNQLCAKILPFSDRPLNLRGW--LIF---SNSNYVLQDFDV---------------
NL63-related VHDDDYVI--ALTGTNLLCAKIRPFSDRPRNLRGW--LVF---SNSNYVLQEFDV---------------
Porcine-EDV LLPEDYVM--VVIGTTKLSAYVDTFGSRPRNICGW--LLF---SNCNYFLEELEL---------------
TGE DRKDHYVI--GVLGNGVSDLKPVLLTEPSVMLQGF--IVR---ANCNGVLEDFDL---------------
Beta-CoV cons
-5432------4333446200100000-152375541----331456-64304*577*3-12----11162232--739873500300000200102-000002120416336233-----558*52
Alpha-CoV cons
312646*7--771731201736227334605+4*9--+92---877453344812---------------
Combined cons
-3231--3445122--6362325336324344--203---001412342634---------------
Alignment qual

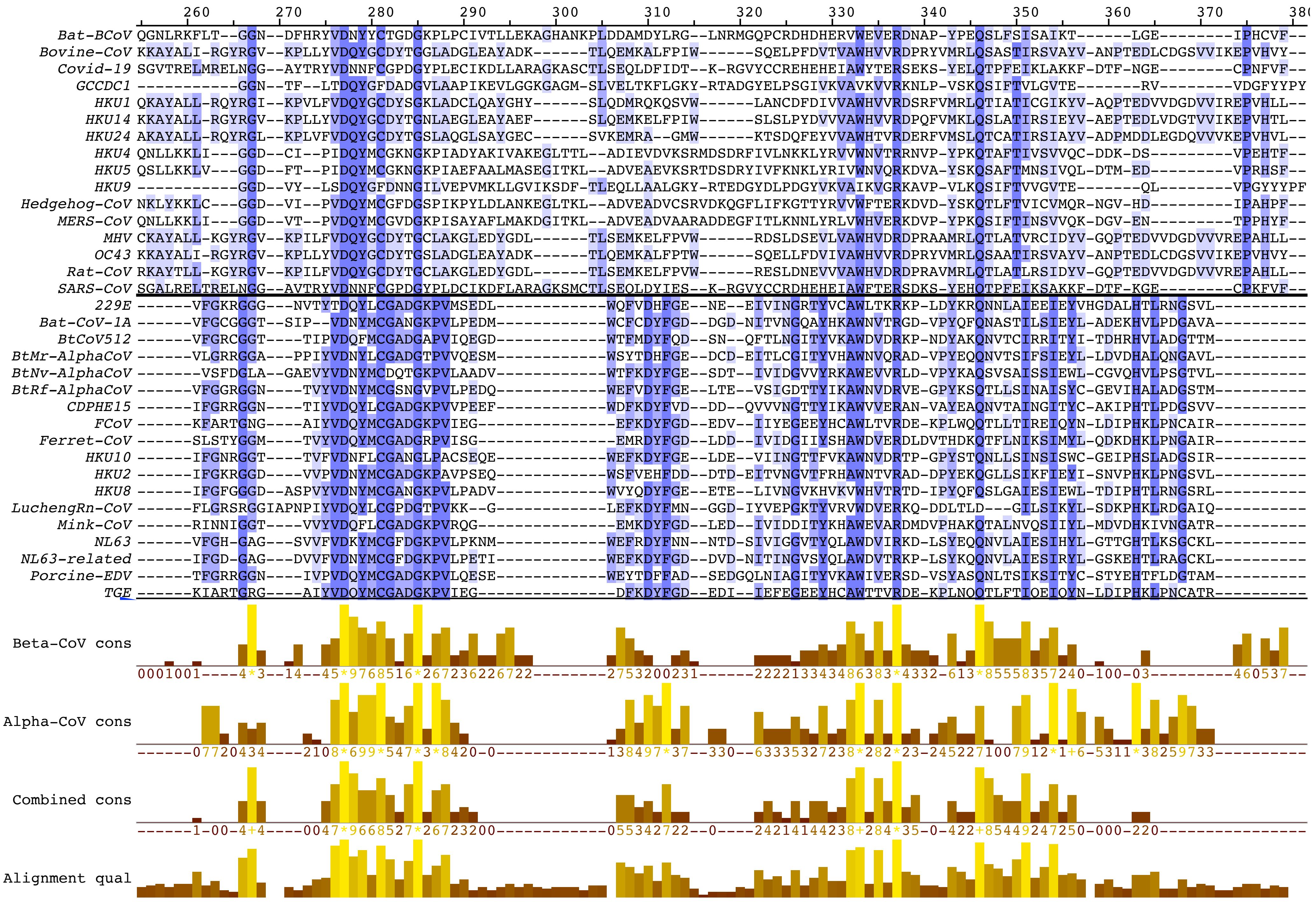

260
270
280
290
300
310
320
330
340
350
360
370
380
Bat-BCoV
Bovine-CoV
Covid-19
GCCDC1
HKU1
HKU14
HKU24
HKU4
HKU5
HKU9
Hedgehog-CoV
MERS-CoV
MHV
OC43
Rat-CoV
SARS-CoV
229E
Bat-CoV-1A
BtCoV512
BtMr-AlphaCoV
BtNv-AlphaCoV
BtRf-AlphaCoV
CDPHE15
FCoV
Ferret-CoV
HKU10
HKU2
HKU8
LuchengRn-CoV
Mink-CoV
NL63
NL63-related
Porcine-EDV
TGE
Beta-CoV cons
Alpha-CoV cons
Combined cons
Alignment qual

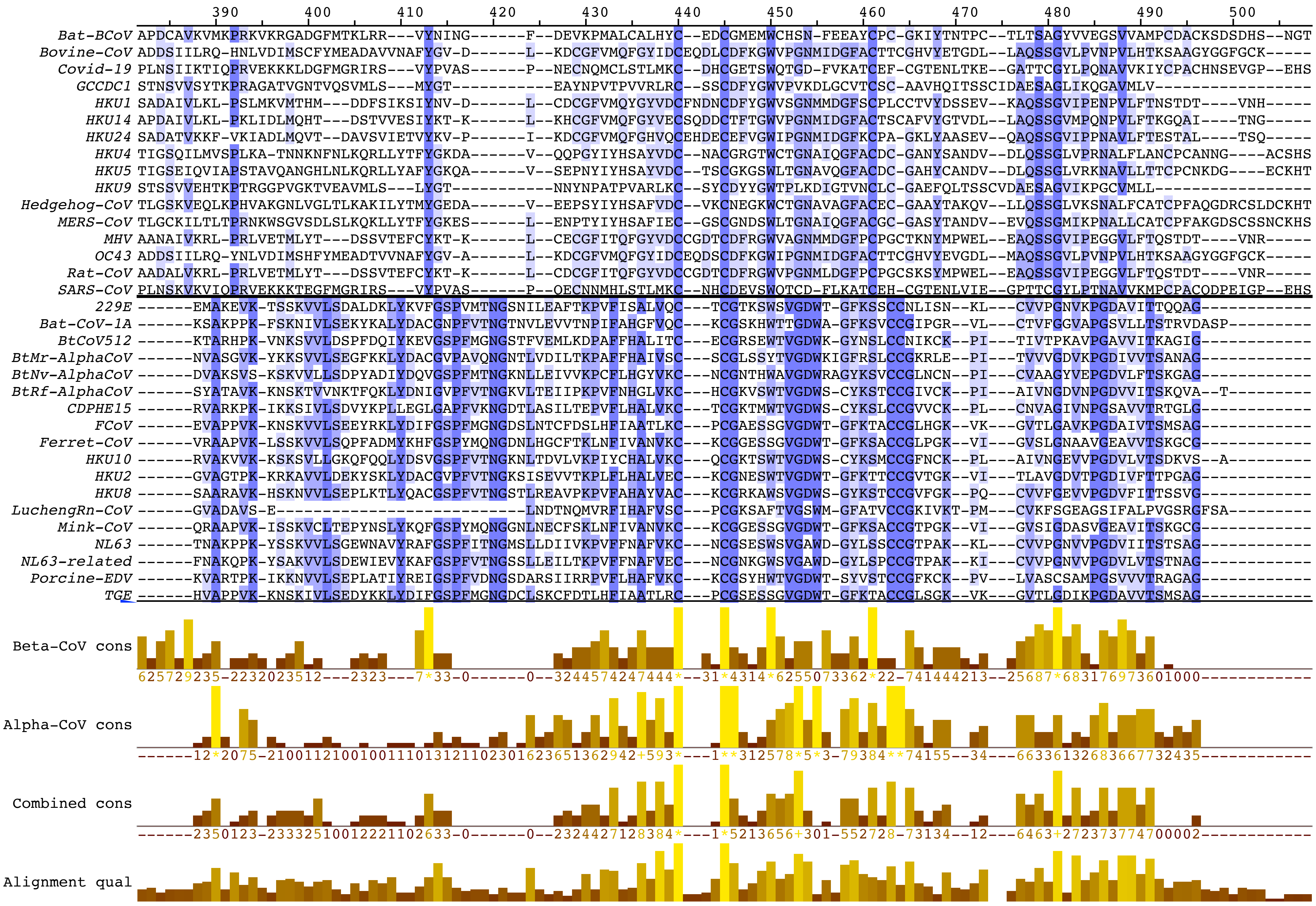

390
400
410
420
430
440
450
460
470
480
490
500
Bat-BCoV
Bovine-CoV
Covid-19
GCCDC1
HKU1
HKU14
HKU24
HKU4
HKU5
HKU9
Hedgehog-CoV
MERS-CoV
MHV
OC43
Rat-CoV
SARS-CoV
229E
Bat-CoV-1A
BtCoV512
BtMr-AlphaCoV
BtNv-AlphaCoV
BtRf-AlphaCoV
CDPHE15
FCoV
Ferret-CoV
HKU10
HKU2
HKU8
LuchengRn-CoV
Mink-CoV
NL63
NL63-related
Porcine-EDV
TGE
Beta-CoV cons
Alpha-CoV cons
Combined cons
Alignment qual

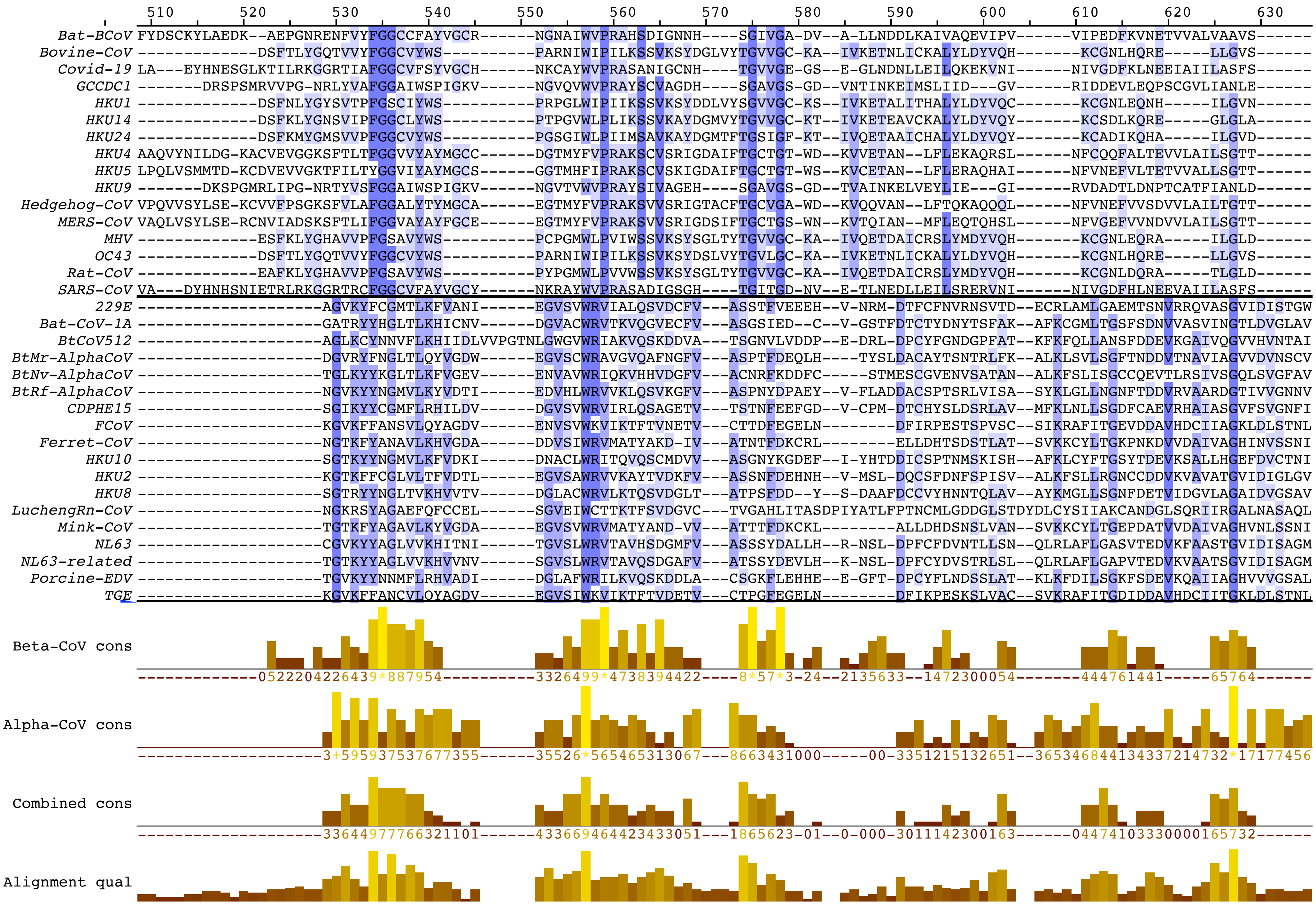

510 520 530 540 550 560 570 580 590 600 610 620 630
Bat-BCoV
Bovine-CoV
Covid-19
GCCDC1
HKU1
HKU14
HKU24
HKU4
HKU5
HKU9
Hedgehog-CoV
MERS-CoV
MHV
OC43
Rat-CoV
SARS-CoV
229E
Bat-CoV-1A
BtCoV512
BtMr-AlphaCoV
BtNv-AlphaCoV
BtRf-AlphaCoV
CDPHE15
FCoV
Ferret-CoV
HKU10
HKU2
HKU8
LuchengRn-CoV
Mink-CoV
NL63
NL63-related
Porcine-EDV
TGE
Beta-CoV cons
Alpha-CoV cons
Combined cons
Alignment qual

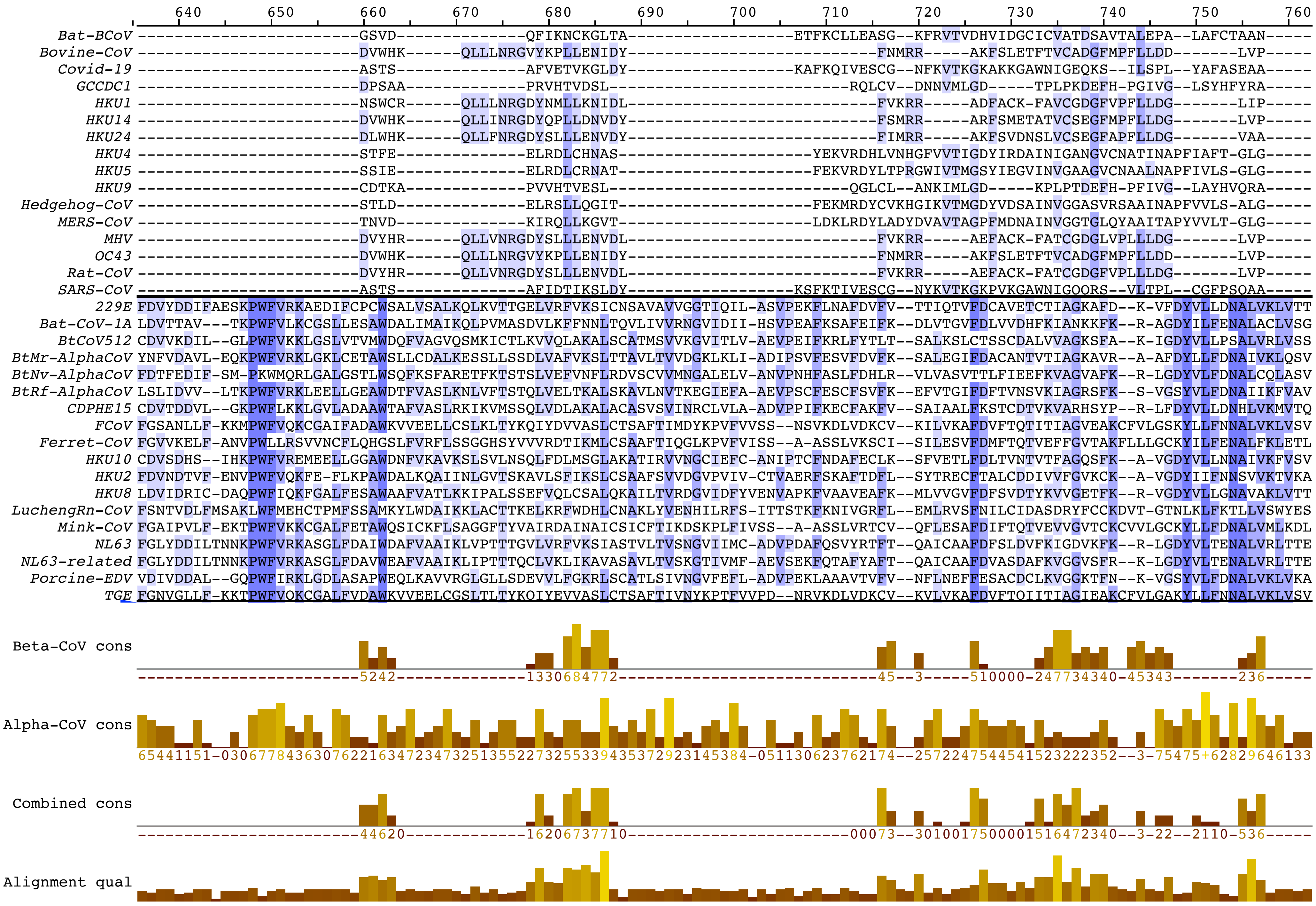

640
650
660
670
680
690
700
710
720
730
740
750
760
Bat-BCoV
Bovine-CoV
Covid-19
GCCDC1
HKU1
HKU14
HKU24
HKU4
HKU5
HKU9
Hedgehog-CoV
MERS-CoV
MHV
OC43
Rat-CoV
SARS-CoV
229E
Bat-CoV-1A
BtCoV512
BtMr-AlphaCoV
BtNv-AlphaCoV
BtRf-AlphaCoV
CDPHE15
FCoV
Ferret-CoV
HKU10
HKU2
HKU8
LuchengRn-CoV
Mink-CoV
NL63
NL63-related
Porcine-EDV
TGE
Beta-CoV cons
Alpha-CoV cons
Combined cons
Alignment qual

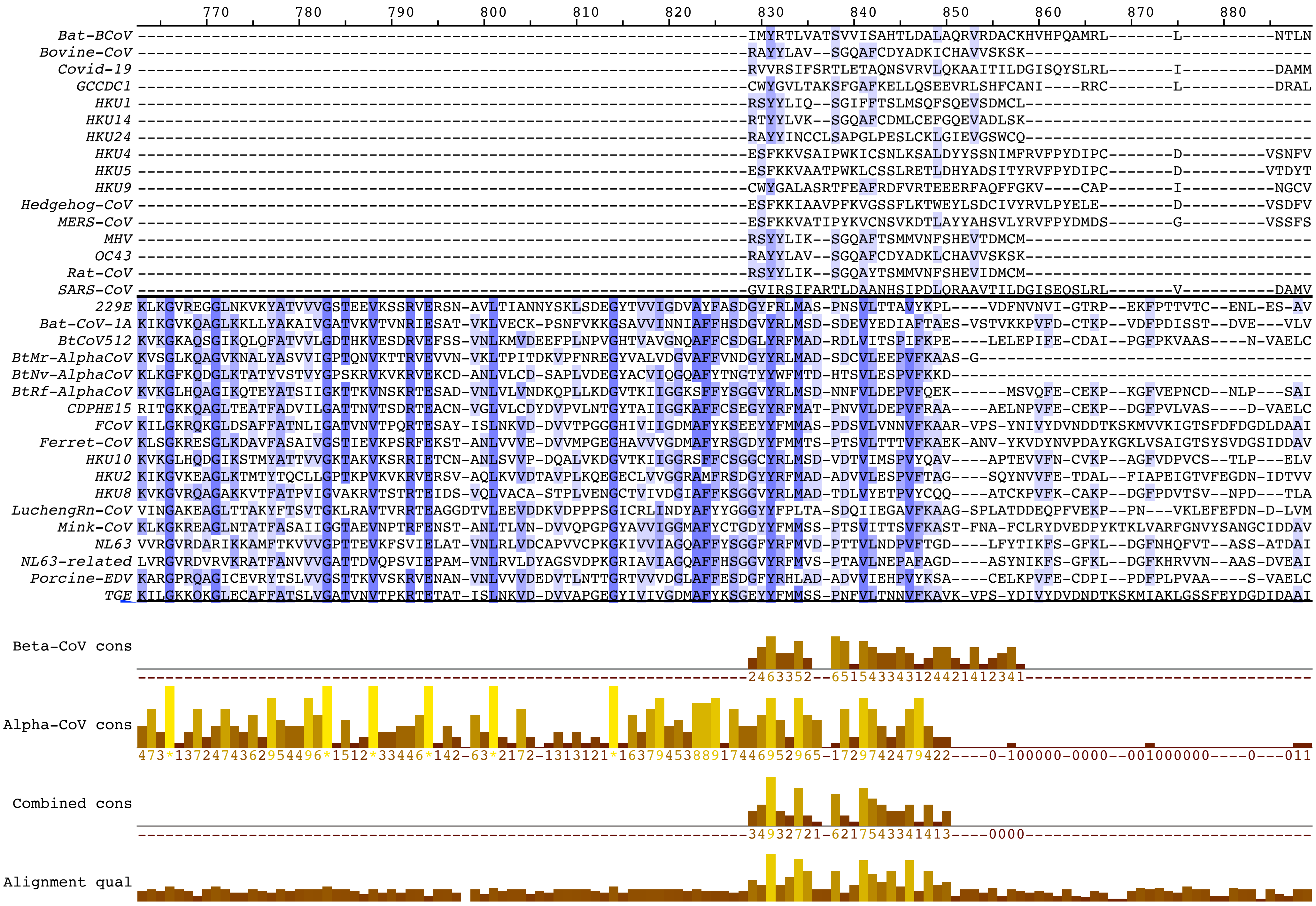
770
780
790
800
810
820
830
840
850
860
870
880
Bat-BCoV
Bovine-CoV
Covid-19
GCCDC1
HKU1
HKU14
HKU24
HKU4
HKU5
HKU9
Hedgehog-CoV
MERS-CoV
MHV
OC43
Rat-CoV
SARS-CoV
229E
Bat-CoV-1A
BtCoV512
BtMr-AlphaCoV
BtNv-AlphaCoV
BtRf-AlphaCoV
CDPHE15
FCoV
Ferret-CoV
HKU10
HKU2
HKU8
LuchengRn-CoV
Mink-CoV
NL63
NL63-related
Porcine-EDV
TGE
Beta-CoV cons
Alpha-CoV cons
Combined cons
Alignment qual

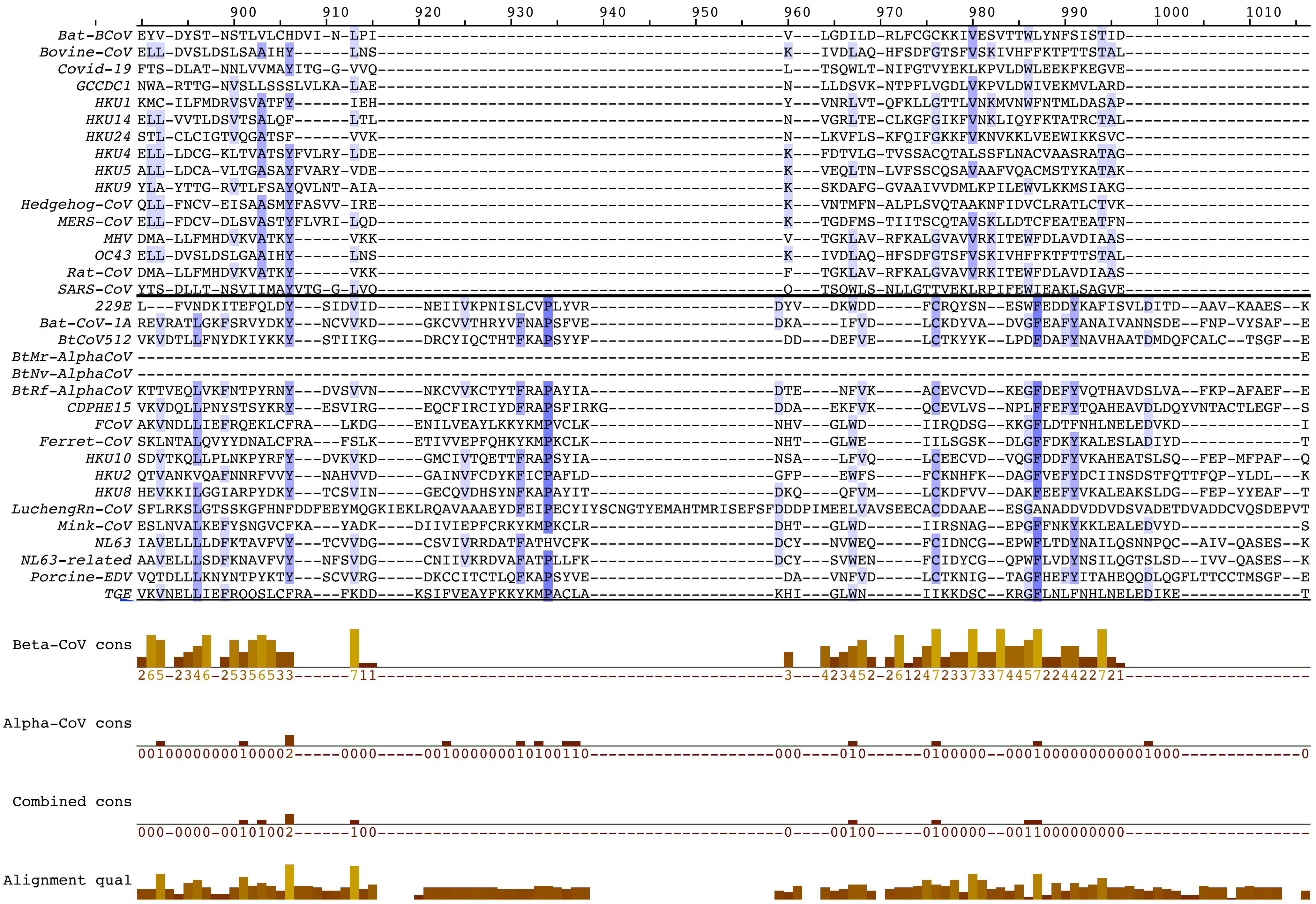

900
910
920
930
940
950
960
970
980
990
1000
1010
Bat-BCoV
Bovine-CoV
Covid-19
GCCDC1
HKU1
HKU14
HKU24
HKU4
HKU5
HKU9
Hedgehog-CoV
MERS-CoV
MHV
OC43
Rat-CoV
SARS-CoV
229E
Bat-CoV-1A
BtCoV512
BtMr-AlphaCoV
BtNv-AlphaCoV
BtRf-AlphaCoV
CDPHE15
FCoV
Ferret-CoV
HKU10
HKU2
HKU8
LuchengRn-CoV
Mink-CoV
NL63
NL63-related
Porcine-EDV
TGE
Beta-CoV cons
Alpha-CoV cons
Combined cons
Alignment qual

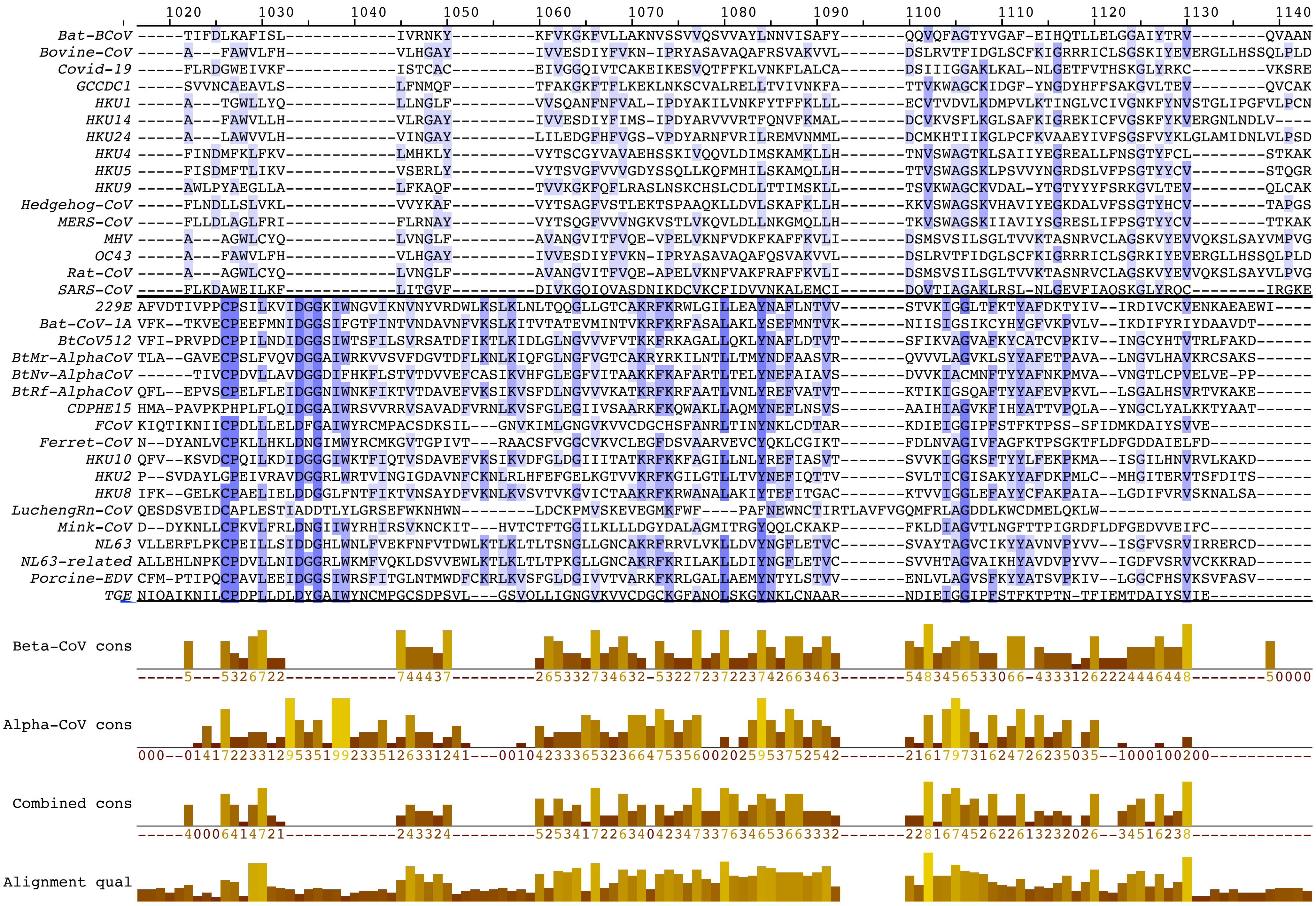

1020
1030
1040
1050
1060
1070
1080
1090
1100
1110
1120
1130
1140
Bat-BCoV
Bovine-CoV
Covid-19
GCCDC1
HKU1
HKU14
HKU24
HKU4
HKU5
HKU9
Hedgehog-CoV
MERS-CoV
MHV
OC43
Rat-CoV
SARS-CoV
229E
Bat-CoV-1A
BtCoV512
BtMr-AlphaCoV
BtNv-AlphaCoV
BtRf-AlphaCoV
CDPHE15
FCoV
Ferret-CoV
HKU10
HKU2
HKU8
LuchengRn-CoV
Mink-CoV
NL63
NL63-related
Porcine-EDV
TGE
Beta-CoV cons
Alpha-CoV cons
Combined cons
Alignment qual

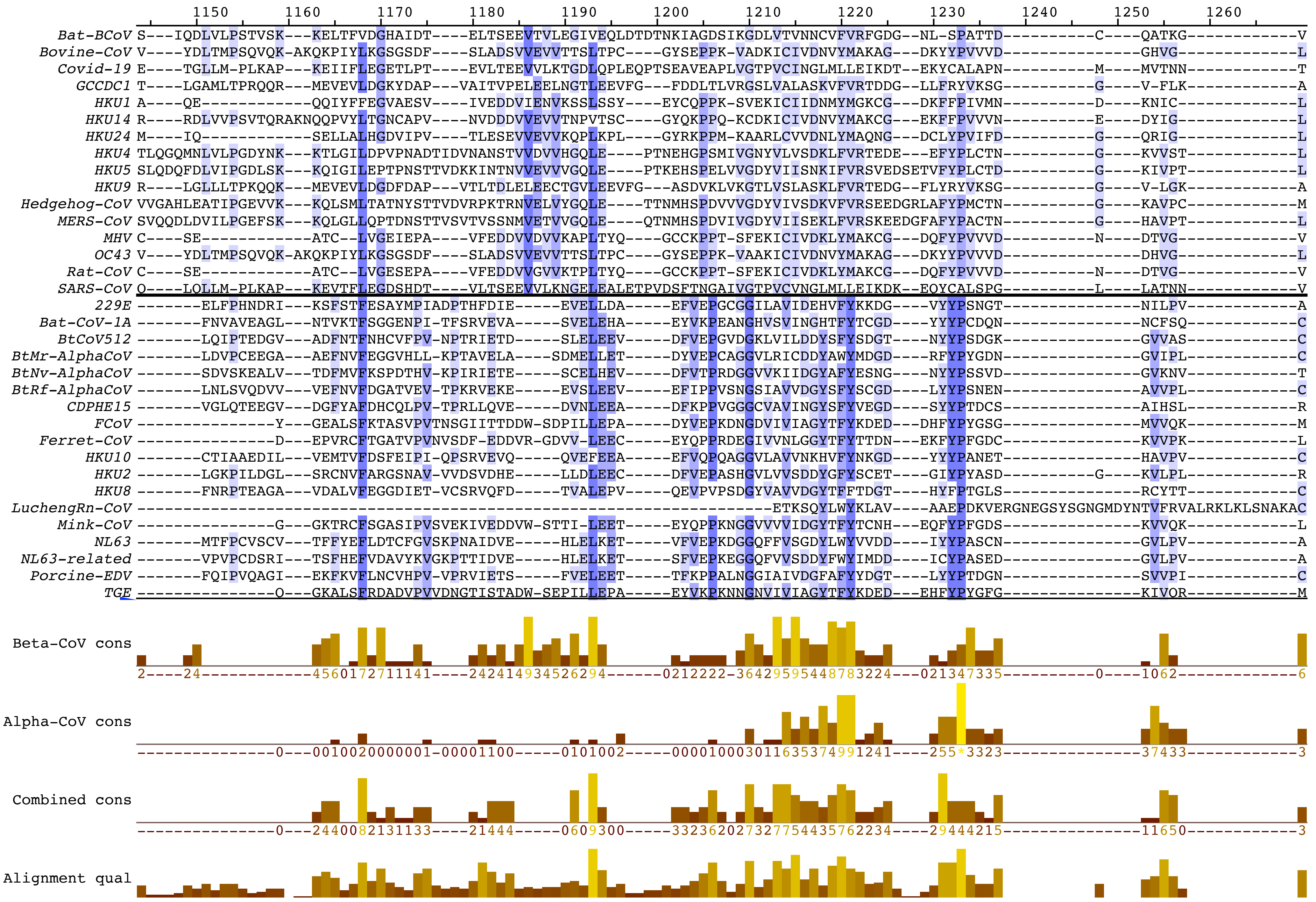
1150 1160 1170 1180 1190 1200 1210 1220 1230 1240 1250 1260
Bat-BCoV S---IQDLVLPSTVSK---KELTFVDGHAIDT----ELTSEEVTVLEGIVEQLDTDTNKIAGDSIKGDLVTVNNCVFVRFGDG--NL-SPATTD----------C----QATKG------------V
Bovine-CoV V----YDLTMPSQVQK-AKQKPIYLKGSGSDF----SLADSVVEVVTTSLTPC----GYSEPPK-VADKICIVDNVYMAKAG---DKYYPVVVD---------------GHVG-------------L
Covid-19 E----TGLLM-PLKAP---KEIIFLEGETLPT----EVLTEEVVLKTGDLQPLEQPTSEAVEAPLVGTPVCINGLMLLEIKDT--EKYCALAPN----------M----MVTNN------------T
GCCDC1 T----LGAMLTPRQQR---MEVEVLDGKYDAP---VAITVPELEELNGTLEEVFG---FDDLTLVRGSLVALASKVFVRTDDG--LLFRYVKSG----------G----V-FLK------------A
HKU1 A----QE------------QQIYFFEGVAESV----IVEDDVIENVKSSLSSY----EYCQPPK-SVEKICIIDNMYMGKCG---DKFFPIVMN----------D----KNIC-------------L
HKU14 R----RDLVVPSVTQRAKNQQPVYLTGNCAPV----NVDDDVVEVVTNPVTSC----GYQKPPQ-KCDKICIVDNVYMAKCG---EKFFPVVVN----------E----DYIG-------------L
HKU24 M----IQ------------SELLALHGDVIPV----TLESEVVEVVKQPLKPL----GYRKPPM-KAARLCVVDNLYMAQNG---DCLYPVIFD----------G----QRIG-------------L
HKU4 TLQGQMNLVLPGDYNK---KTLGILDPVPNADTIDVNANSTVVDVVHGQLE----PTNEHGPSMIVGNYVLVSDKLFVRTEDE---EFYPLCTN----------G----KVVST------------L
HKU5 SLQDQFDLVIPGDLSK---KQIGILEPTPNSTTVDKKINTNVVEVVGQLE----PTKEHSPELVVGDYVIISNKIFVRSVEDSETVFYPLCTD----------G----KIVPT------------L
HKU9 R----LGLLLTPKQQK---MEVEVLDGDFDAP---VTLTDLELEECTGVLEEVFG---ASDVKLVKGTLVSLASKLFVRTEDG--FLYRYVKSG----------G----V-LGK------------A
Hedgehog-CoV VVGAHLEATIPGEVVK---KQLSMLTATNYSTTVDVRPKTRNVELVYGQLE----TTNMHSPDVVVGDYVIVSDKVFVRSEEDGRLAFYPMCTN----------G----KAVPC------------M
MERS-CoV SVQQDLDVILPGEFSK---KQLGLLQPTDNSTTVSVTVSSNMVETVVGQLE----QTNMHSPDVIVGDYVIISEKLFVRSKEEDGFAFYPACTN----------G----HAVPT------------L
MHV C----SE------------ATC--LVGEIEPA----VFEDDVVDVVKAPLTYQ----GCCKPPT-SFEKICIVDKLYMAKCG---DQFYPVVVD----------N----DTVG-------------V
OC43 V----YDLTMPSQVQK-AKQKPIYLKGSGSDF----SLADSVVEVVTTSLTPC----GYSEPPK-VAAKICIVDNVYMAKAG---DKYYPVVVD---------------DHVG-------------L
Rat-CoV C----SE------------ATC--LVGESEPA----VFEDDVVGVVKTPLTYQ----GCCKPPT-SFEKICIVDKLYMAKCG---DQFYPVVVD----------N----DTVG-------------V
SARS-CoV Q----LQLLM-PLKAP---KEVTFLEGDSHDT----VLTSEEVVLKNGELEALETPVDSFTNGAIVGTPVCVNGLMLLEIKDK--EQYCALSPG----------L----LATNN------------V
229E -------ELFPHNDRI---KSFSTFESAYMPIADPTHFDIE-----EVELLDA-----EFVEPGCGGILAVIDEHVFYKKDG----VYYPSNGT---------------NILPV------------A
Bat-CoV-1A -------FNVAVEAGL---NTVKTFSGGENPI-TFSRVEVA-----SVELEHA-----EYVKPEANGHVSVINGHTFYTCGD----YYYPCDQN---------------NCFSQ------------C
BtCoV512 -------LQIPTEDGV---ADFNTFNHCVFPV-NPTRIETD-----SLELEEV-----DFVEPCGVDGKLVILDDYSFYSDGT----NYYPSDGK---------------GVVAS------------C
BtMr-AlphaCoV -------LDVPCEEGA---AEFNVFEGGVHLL-KPTAVELA----SDMELLET-----DYVEPCAGGVLRICDDYAWYMDGD----RFYPYGDN---------------GVIPL------------C
BtNv-AlphaCoV -------SDVSKEALV---TDFMVFKSPDTHV-KPIRIETE-----SCELHEV-----DFVTPRDGGVVKIIDGYAFYESNG----NYYPSSVD---------------GVKNV------------T
BtRf-AlphaCoV -------LNLSVQDVV---VEFNVFDGATVEV-TPKRVEKE-----EVSLEEV-----EFIPPVSNGSIAVVDGYSFYSCGD----LYYPSNEN---------------AVVPL------------C
CDPHE15 -------VGLQTEEGV---DGFYAFDHCQLPV-TPRLLQVE-----DVNLEEA-----DFKPPVGGGCVAVINGYSFYVEGD----SYYPTDCS---------------AIHSL------------R
FCoV ---------------Y---GEALSFKTASVPVTNSGIITTDDW-SDPILLEPA-----DYVEPKDNGDVIVIAGYTFYKDED---DHFYPYGSG---------------MVVQK------------M
Ferret-CoV ---------------D---EPVRCFTGATVPVNVSDF-EDDVR-GDVV-LEEC-----EYQPPRDEGIVVNLGGYTFYTTDN---EKFYPFGDC---------------KVVPK------------L
HKU10 -------CTIAAEDIL---VEMTVFDSFEIPI-QPSRVEVQ-----QVEFEEA-----EFVQPQAGGVLAVVNKHVFYNKGD----YYYPANET---------------HAVPV------------C
HKU2 -------LGKPILDGL---SRCNVFARGSNAV-VVDSVDHE-----LLDLEEC-----DFVEPASHGVLVVSDDYGFYSCET----GIYPYASD----------G----KVLPL------------R
HKU8 -------FNRPTEAGA---VDALVFEGGDIET-VCSRVQFD-----TVALEPV-----QEVPVPSDGYVAVVDGYTFFTDGT----HYFPTGLS---------------RCYTT------------C
LuchengRn-CoV --ETKSQYLWYKLAV----AAEPDKVERGNEGSYSGNGMDYNTVFRVALRKLKLSNAKAC
Mink-CoV ---------------G---GKTRCFSGASIPVSVEKIVEDDVW-STTI-LEET-----EYQPPKNGGVVVVIDGYTFYTCNH---EQFYPFGDS---------------KVVQK------------L
NL63 -------MTFPCVSCV---TFFYEFLDTCFGVSKPNAIDVE-----HLELKET-----VFVEPKDGGQFFVSGDYLWYVVDD----IYYPASCN---------------GVLPV------------A
NL63-related -------VPVPCDSRI---TSFHEFVDAVYKVGKPTTIDVE-----HLELKET-----SFVEPKEGGQFVVSDDYFWYIMDD----ICYPASED---------------GVLPV------------A
Porcine-EDV -------FQIPVQAGI---EKFKVFLNCVHPV-VPRVIETS-----FVELEET-----TFKPPALNGGIAIVDGFAFYYDGT----LYYPTDGN---------------SVVPI------------C
TGE ---------------Q---GKALSFRDADVPVVDNGTISTADW-SEPILLEPA-----EYVKPKNNGNVIVIAGYTFYKDED---EHFYPYGFG---------------KIVQR------------M
Beta-CoV cons
2----24------------456017271114 1----2424149345262 94------0212222-3642959544878 3224---021347335----------0----1062------------6
Alpha-CoV cons
---------------0---0010020000001-00001100-----0101002-----0000100030116353749912 41----255*3323---------------37433------------3
Combined cons
---------------0---24400821311 33----21444-----0609300-----33236202732775443576 2234----29444215---------------11650------------3
Alignment qual

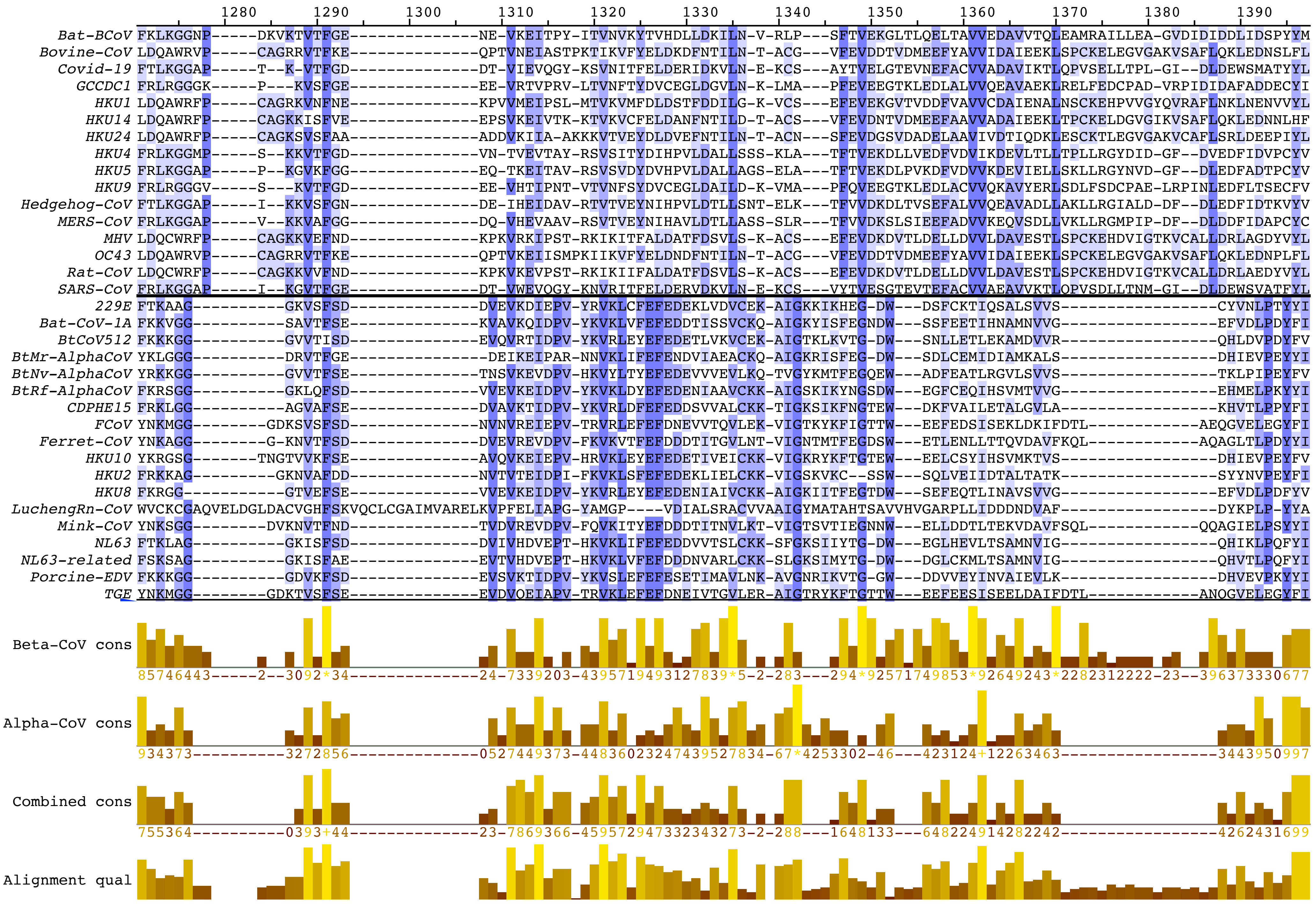

1280
1290
1300
1310
1320
1330
1340
1350
1360
1370
1380
1390
Bat-BCoV
Bovine-CoV
Covid-19
GCCDC1
HKU1
HKU14
HKU24
HKU4
HKU5
HKU9
Hedgehog-CoV
MERS-CoV
MHV
OC43
Rat-CoV
SARS-CoV
229E
Bat-CoV-1A
BtCoV512
BtMr-AlphaCoV
BtNv-AlphaCoV
BtRf-AlphaCoV
CDPHE15
FCoV
Ferret-CoV
HKU10
HKU2
HKU8
LuchengRn-CoV
Mink-CoV
NL63
NL63-related
Porcine-EDV
TGE
Beta-CoV cons
Alpha-CoV cons
Combined cons
Alignment qual

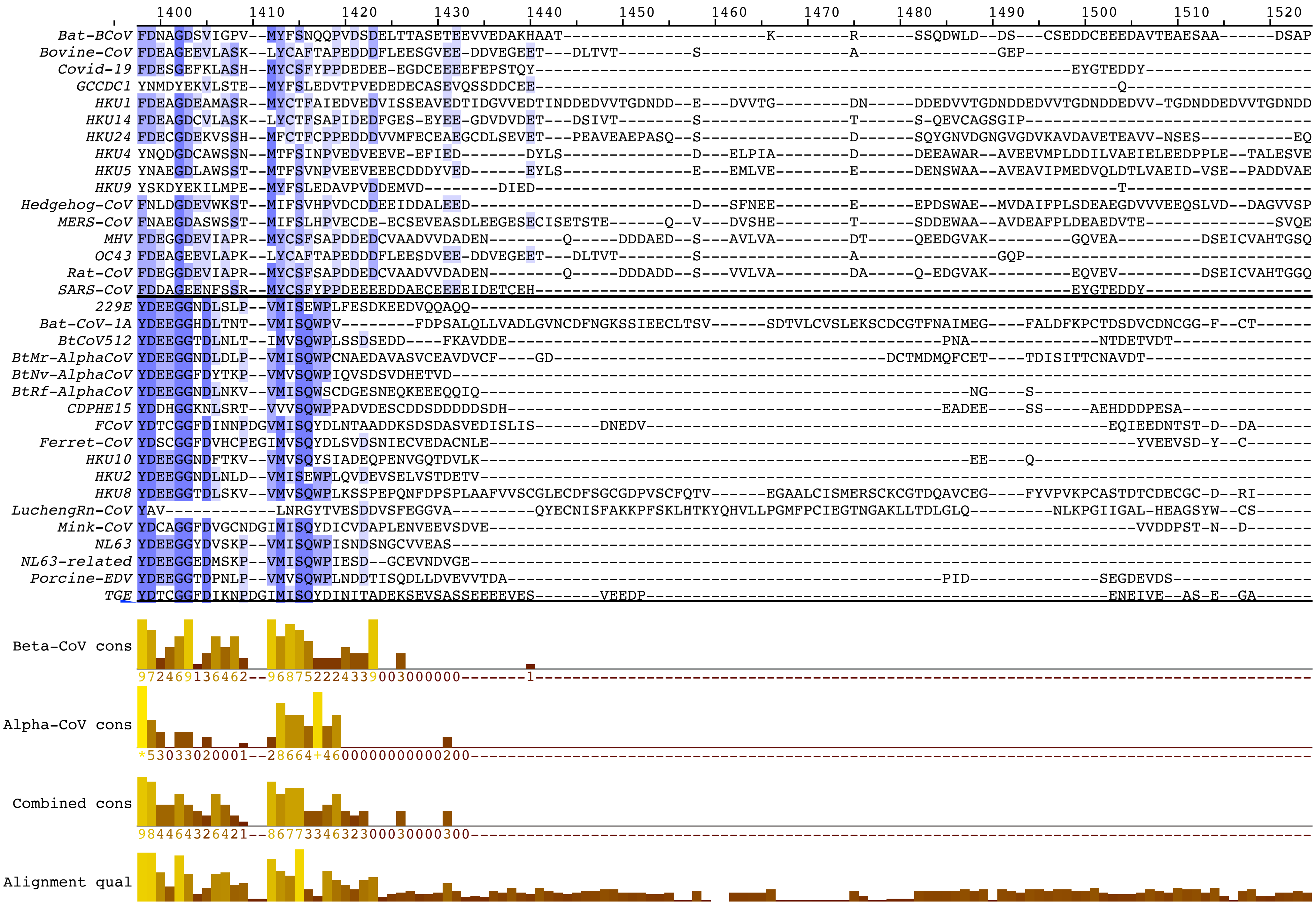
Bat-BCoV
Bovine-CoV
Covid-19
GCCDC1
HKU1
HKU14
HKU24
HKU4
HKU5
HKU9
Hedgehog-CoV
MERS-CoV
MHV
OC43
Rat-CoV
SARS-CoV
229E
Bat-CoV-1A
BtCoV512
BtMr-AlphaCoV
BtNv-AlphaCoV
BtRf-AlphaCoV
CDPHE15
FCoV
Ferret-CoV
HKU10
HKU2
HKU8
LuchengRn-CoV
Mink-CoV
NL63
NL63-related
Porcine-EDV
TGE
Beta-CoV cons
Alpha-CoV cons
Combined cons
Alignment qual

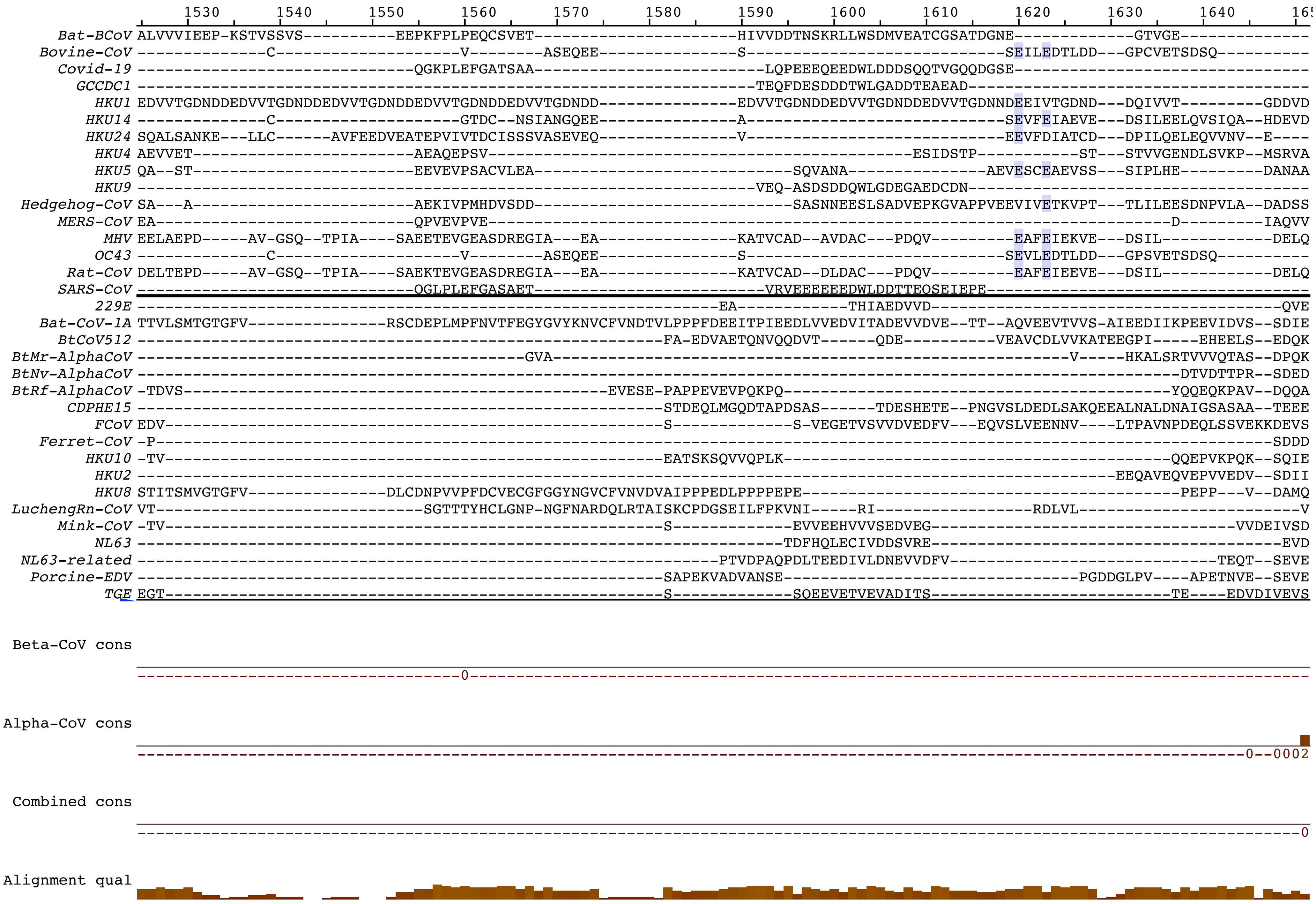
1530
1540
1550
1560
1570
1580
1590
1600
1610
1620
1630
1640
Bat-BCoV
Bovine-CoV
Covid-19
GCCDC1
HKU1
HKU14
HKU24
HKU4
HKU5
HKU9
Hedgehog-CoV
MERS-CoV
MHV
OC43
Rat-CoV
SARS-CoV
229E
Bat-CoV-1A
BtCoV512
BtMr-AlphaCoV
BtNv-AlphaCoV
BtRf-AlphaCoV
CDPHE15
FCoV
Ferret-CoV
HKU10
HKU2
HKU8
LuchengRn-CoV
Mink-CoV
NL63
NL63-related
Porcine-EDV
TGE
Beta-CoV cons
Alpha-CoV cons
Combined cons
Alignment qual

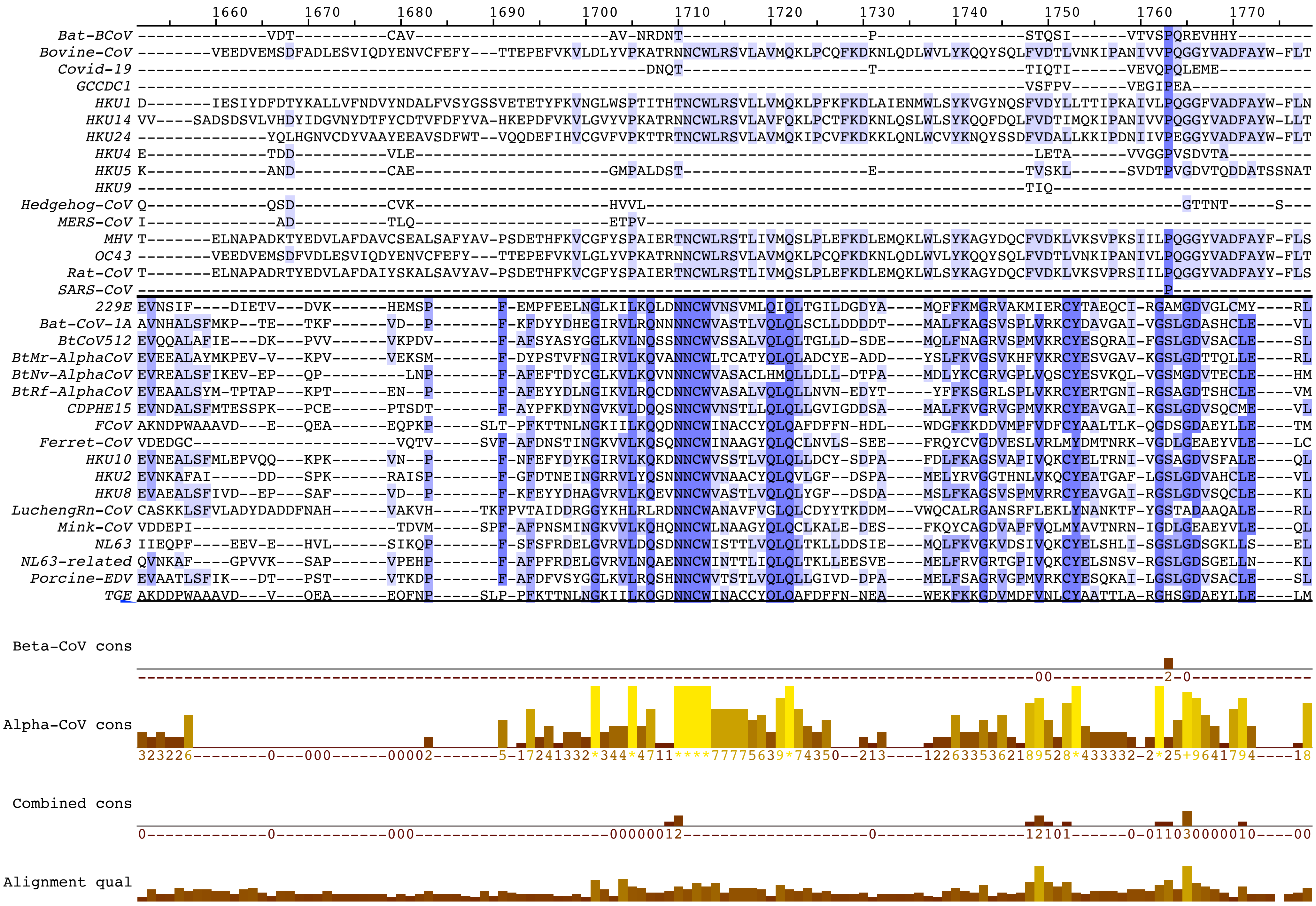

1660
1670
1680
1690
1700
1710
1720
1730
1740
1750
1760
1770
Bat-BCoV
Bovine-CoV
Covid-19
GCCDC1
HKU1
HKU14
HKU24
HKU4
HKU5
HKU9
Hedgehog-CoV
MERS-CoV
MHV
OC43
Rat-CoV
SARS-CoV
229E
Bat-CoV-1A
BtCoV512
BtMr-AlphaCoV
BtNv-AlphaCoV
BtRf-AlphaCoV
CDPHE15
FCoV
Ferret-CoV
HKU10
HKU2
HKU8
LuchengRn-CoV
Mink-CoV
NL63
NL63-related
Porcine-EDV
TGE
Beta-CoV cons
Alpha-CoV cons
Combined cons
Alignment qual

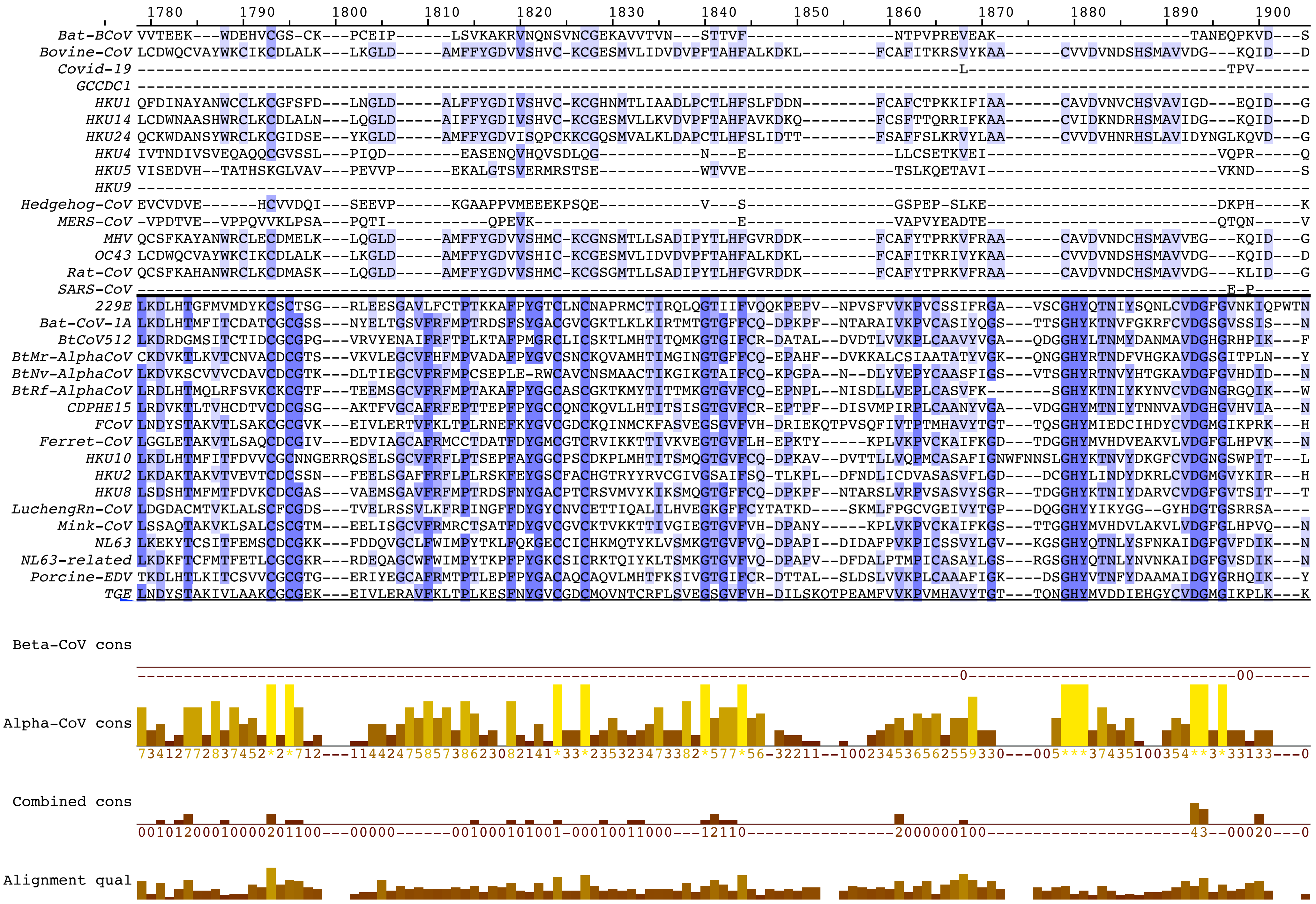

1780
1790
1800
1810
1820
1830
1840
1850
1860
1870
1880
1890
1900
Bat-BCoV
Bovine-CoV
Covid-19
GCCDC1
HKU1
HKU14
HKU24
HKU4
HKU5
HKU9
Hedgehog-CoV
MERS-CoV
MHV
OC43
Rat-CoV
SARS-CoV
229E
Bat-CoV-1A
BtCoV512
BtMr-AlphaCoV
BtNv-AlphaCoV
BtRf-AlphaCoV
CDPHE15
FCoV
Ferret-CoV
HKU10
HKU2
HKU8
LuchengRn-CoV
Mink-CoV
NL63
NL63-related
Porcine-EDV
TGE
Beta-CoV cons
Alpha-CoV cons
Combined cons
Alignment qual

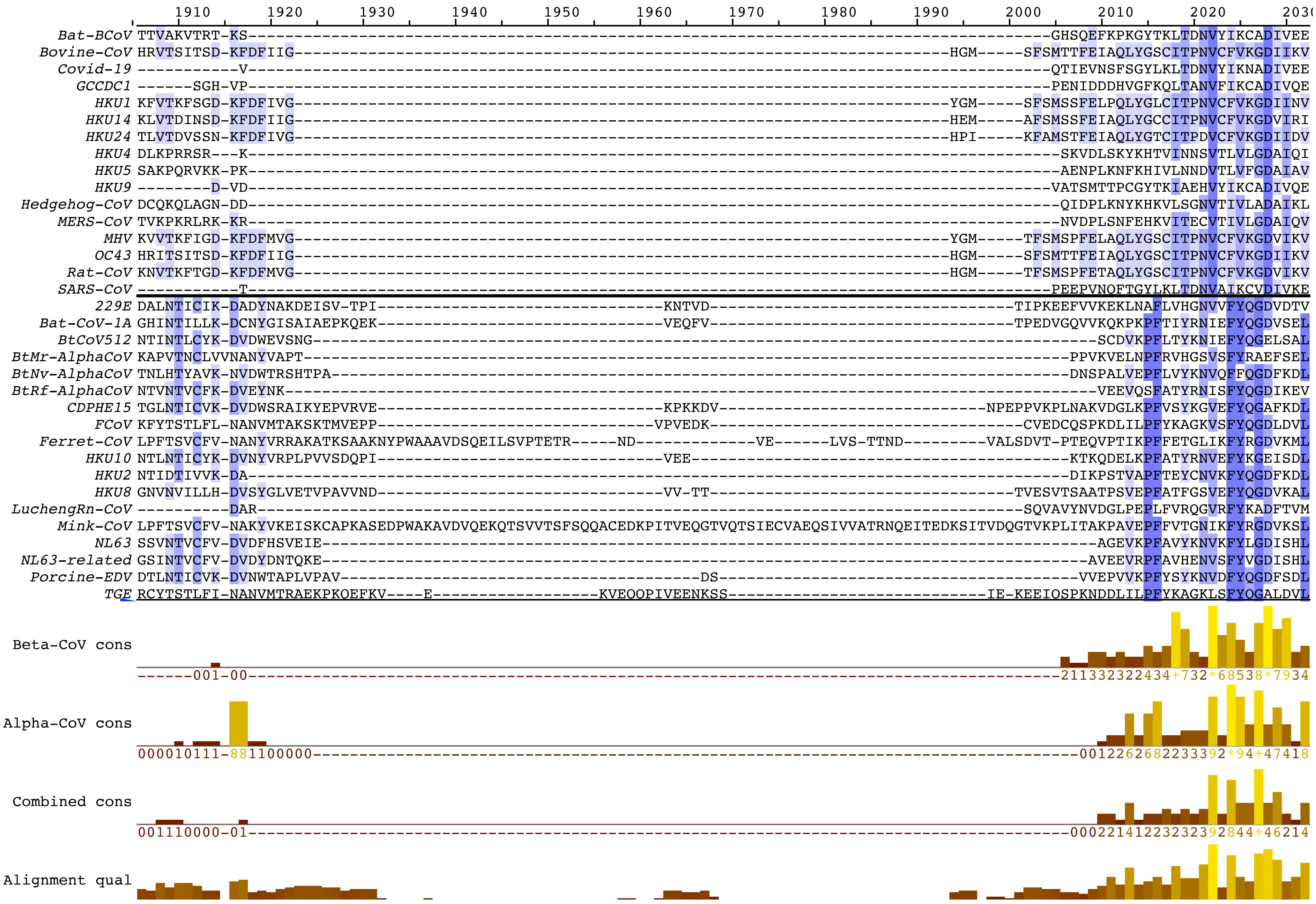
1910 1920 1930 1940 1950 1960 1970 1980 1990 2000 2010 2020 2030
Bat-BCoV TTVAKVTRT-KS---GHSQEFKPKGYTKLTDNVYIKCADIVEE
Bovine-CoV HRVTSITSD-KFDFIIG---HGM-----SFSMTTFEIAQLYGSCITPNVCFVKGDIIKV
Covid-19 -----------V---QTIEVNSFSGYLKLTDNVYIKNADIVEE
GCCDC1 ------SGH-VP---PENIDDDHVGFKQLTANVFIKCADIVQE
HKU1 KFVTKFSGD-KFDFIVG---YGM-----SFSMSSFELPQLYGLCITPNVCFVKGDIINV
HKU14 KLVTDINSD-KFDFIIG---HEM-----AFSMSSFEIAQLYGCCITPNVCFVKGDVIRI
HKU24 TLVTDVSSN-KFDFIVG---HPI-----KFAMSTFEIAQLYGTCITPDVCFVKGDIIDV
HKU4 DLKPRRSR---K--SKVDLSKYKHTVINNSVTLVLGDAIQI
HKU5 SAKPQRVKK-PK--AENPLKNFKHIVLNNDVTLVFGDAIAV
HKU9 --------D-VD---VATSMTTPCGYTKIAEHVYIKCADIVQE
Hedgehog-CoV DCQKQLAGN-DD--QIDPLKNYKHKVLSGNVTIVLADAIKL
MERS-CoV TVKPKRLRK-KR--NVDPLSNFEHKVITECVTIVLGDAIQV
MHV KVVTKFIGD-KFDFMVG---YGM-----TFSMSPFELAQLYGSCITPNVCFVKGDVIKV
OC43 HRITSITSD-KFDFIIG---HGM-----SFSMTTFEIAQLYGSCITPNVCFVKGDIIKV
Rat-CoV KNVTKFTGD-KFDFMVG---HGM-----TFSMSPFETAQLYGSCITPNVCFVKGDVIKV
SARS-CoV -----------T---PEEPVNQFTGYLKLTDNVAIKCVDIVKE
229E DALNTICIK-DADYNAKDEISV-TPI-------------------------------KNTVD---------------------------------TIPKEEFVVKEKLNAFLVHGNVVFYQGDVDTV
Bat-CoV-1A GHINTILLK-DCNYGISAIAEPKQEK-------------------------------VEQFV---------------------------------TPEDVGQVVKQKPKPFTIYRNIEFYQGDVSEL
BtCoV512 NTINTLCYK-DVDWEVSNG---SCDVKPFLTYKNIEFYQGELSAL
BtMr-AlphaCoV KAPVTNCLVVNANYVAPT---PPVKVELNPFRVHGSVSFYRAEFSEL
BtNv-AlphaCoV TNLHTYAVK-NVDWTRSHTPA--DNSPALVEPFLVYKNVQFFQGDFKDL
BtRf-AlphaCoV NTVNTVCFK-DVEYNK--VEEVQSFATYRNISFYQGDIKEV
CDPHE15 TGLNTICVK-DVDWSRAIKYEPVRVE-------------------------------KPKKDV-----------------------------NPEPPVKPLNAKVDGLKPFVSYKGVEFYQGAFKDL
FCoV KFYTSTLFL-NANVMTAKSKTMVEPP------------------------------VPVEDK----------------------------------CVEDCQSPKDLILPFYKAGKVSFYQGDLDVL
Ferret-CoV LPFTSVCFV-NANYVRRAKATKSAAKNYPWAAAVDSQEILSVPTETR-----ND-------------VE------LVS-TTND---------VALSDVT-PTEQVPTIKPFFETGLIKFYRGDVKML
HKU10 NTLNTICYK-DVNYVRPLPVVSDQPI-------------------------------VEE---KTKQDELKPFATYRNVEFYKGEISDL
HKU2 NTIDTIVVK-DA---DIKPSTVAPFTEYCNVKFYQGDFKDL
HKU8 GNVNVILLH-DVSYGLVETVPAVVND-------------------------------VV-TT---------------------------------TVESVTSAATPSVEPFATFGSVEFYQGDVKAL
LuchengRn-CoV ----------DAR---SQVAVYNVDGLPEPLFVRQGVRFYKADFTVM
Mink-CoV LPFTSVCFV-NAKYVKEISKCAPKASEDPWAKAVDVQEKQTSVVTSFSQQACEDKPITVEQGTVQTSIECVAEQSIVVATRNQEITEDKSITVDQGTVKPLITAKPAVEPFFVTGNIKFYRGDVKSL
NL63 SSVNTVCFV-DVDFHSVEIE--AGEVKPFAVYKNVKFYLGDISHL
NL63-related GSINTVCFV-DVDYDNTQKE---AVEEVRPFAVHENVSFYVGDISHL
Porcine-EDV DTLNTICVK-DVNWTAPLPVAV---------------------------------------DS---------------------------------------VVEPVVKPFYSYKNVDFYQGDFSDL
TGE RCYTSTLFI-NANVMTRAEKPKQEFKV----E------------------KVEQQPIVEENKSS----------------------------IE-KEEIQSPKNDDLILPFYKAGKLSFYQGALDVL
Beta-CoV cons
------001-00--211332322434+732*68538*7934
Alpha-CoV cons
000010111-881100000---0012262682233392*94+47418
Combined cons
001110000-01---00022141223232392844+46214
Alignment qual

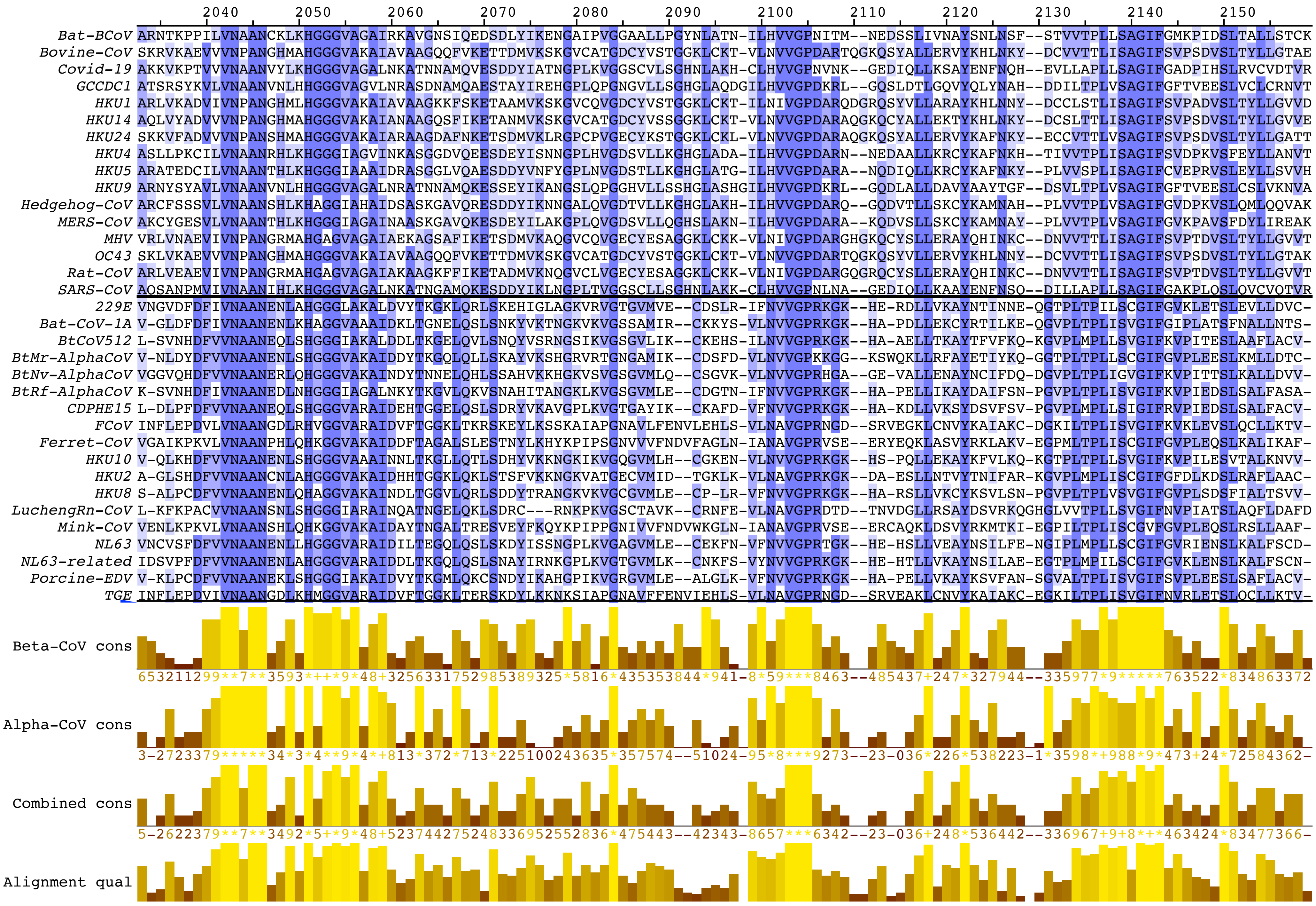
2040
2050
2060
2070
2080
2090
2100
2110
2120
2130
2140
2150
Bat-BCoV
Bovine-CoV
Covid-19
GCCDC1
HKU1
HKU14
HKU24
HKU4
HKU5
HKU9
Hedgehog-CoV
MERS-CoV
MHV
OC43
Rat-CoV
SARS-CoV
229E
Bat-CoV-1A
BtCoV512
BtMr-AlphaCoV
BtNv-AlphaCoV
BtRf-AlphaCoV
CDPHE15
FCoV
Ferret-CoV
HKU10
HKU2
HKU8
LuchengRn-CoV
Mink-CoV
NL63
NL63-related
Porcine-EDV
TGE
Beta-CoV cons
Alpha-CoV cons
Combined cons
Alignment qual

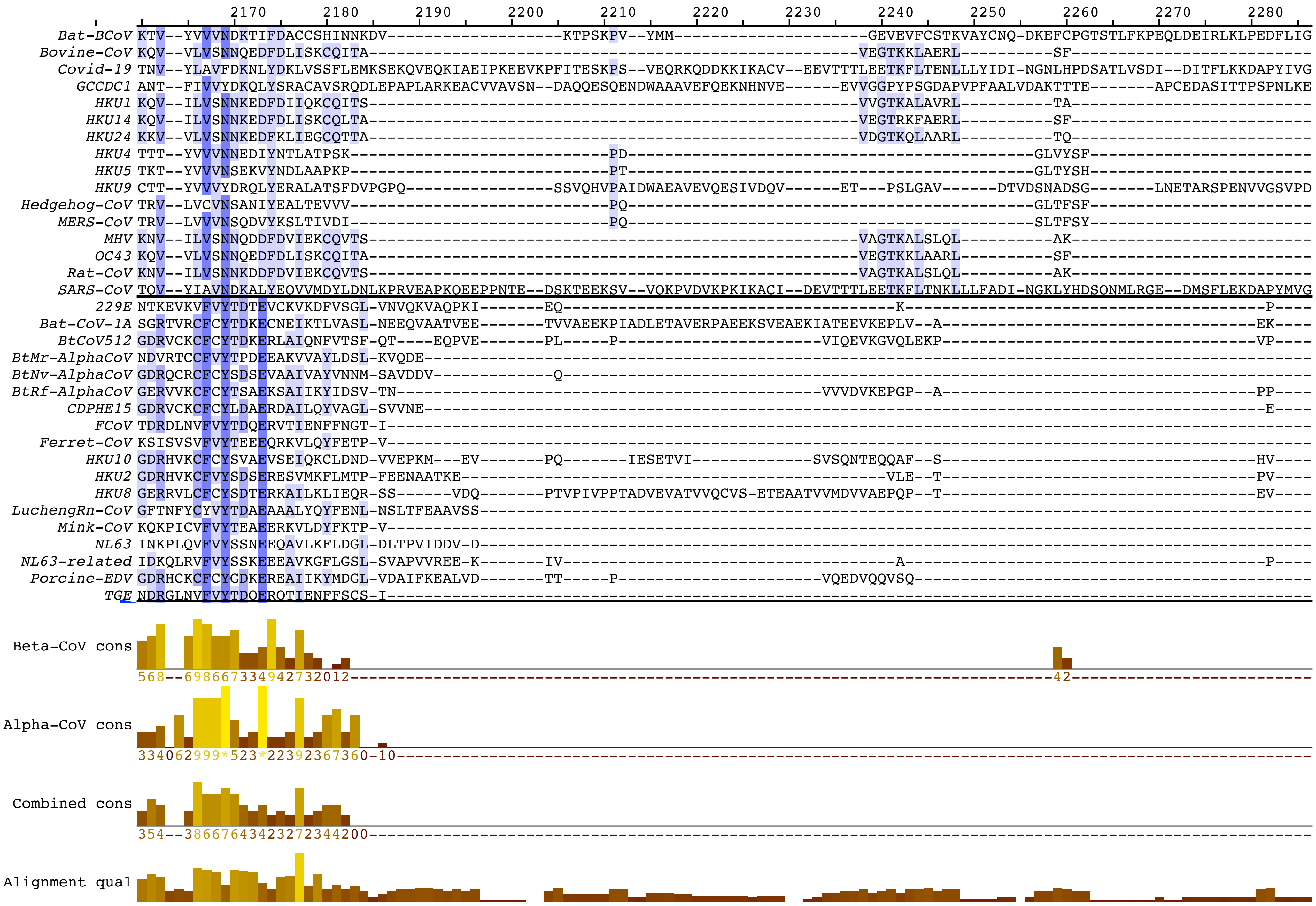

2170
2180
2190
2200
2210
2220
2230
2240
2250
2260
2270
2280
Bat-BCoV
Bovine-CoV
Covid-19
GCCDC1
HKU1
HKU14
HKU24
HKU4
HKU5
HKU9
Hedgehog-CoV
MERS-CoV
MHV
OC43
Rat-CoV
SARS-CoV
229E
Bat-CoV-1A
BtCoV512
BtMr-AlphaCoV
BtNv-AlphaCoV
BtRf-AlphaCoV
CDPHE15
FCoV
Ferret-CoV
HKU10
HKU2
HKU8
LuchengRn-CoV
Mink-CoV
NL63
NL63-related
Porcine-EDV
TGE
Beta-CoV cons
Alpha-CoV cons
Combined cons
Alignment qual

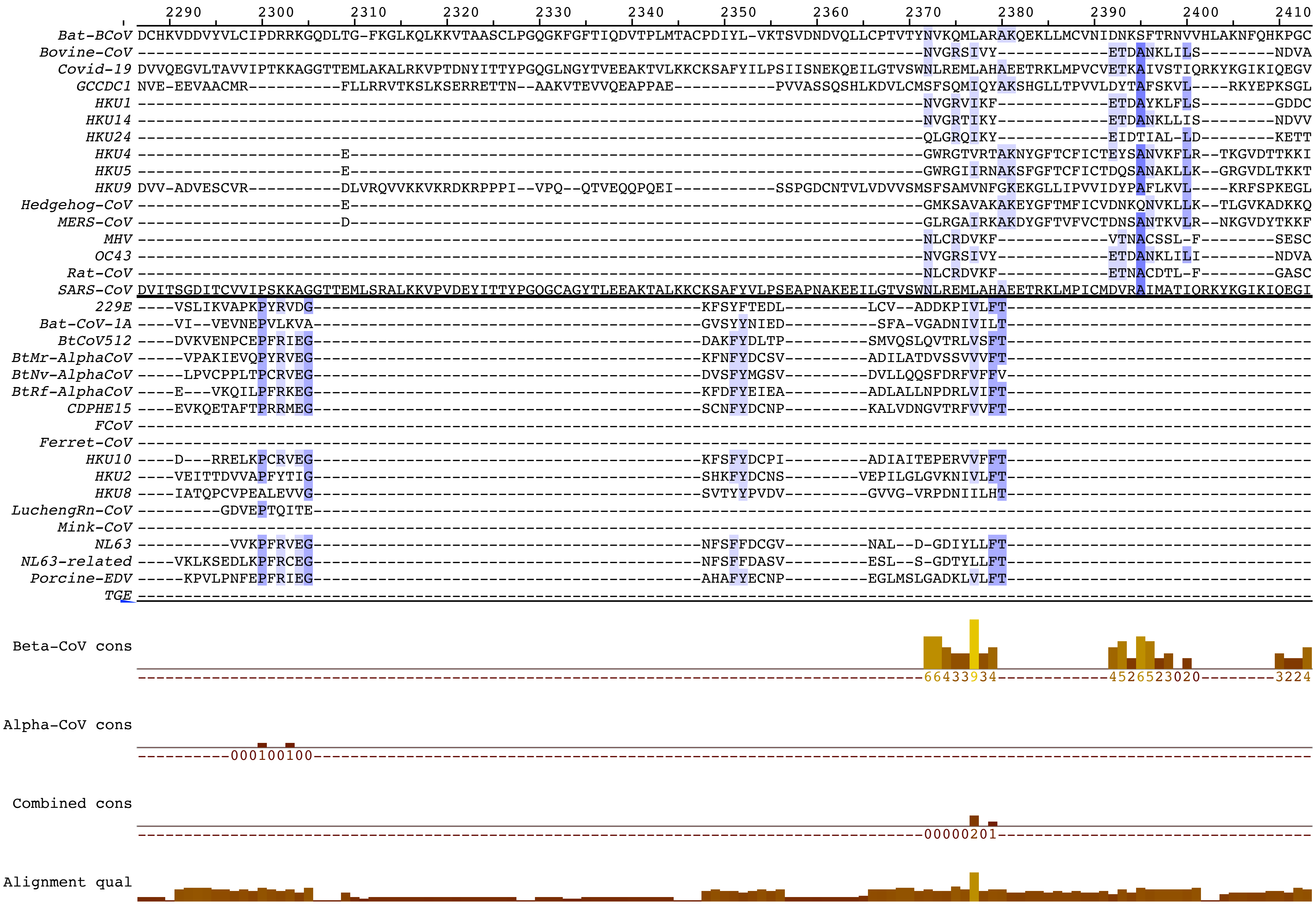
2290
2300
2310
2320
2330
2340
2350
2360
2370
2380
2390
2400
2410
Bat-BCoV
Bovine-CoV
Covid-19
GCCDC1
HKU1
HKU14
HKU24
HKU4
HKU5
HKU9
Hedgehog-CoV
MERS-CoV
MHV
OC43
Rat-CoV
SARS-CoV
229E
Bat-CoV-1A
BtCoV512
BtMr-AlphaCoV
BtNv-AlphaCoV
BtRf-AlphaCoV
CDPHE15
FCoV
Ferret-CoV
HKU10
HKU2
HKU8
LuchengRn-CoV
Mink-CoV
NL63
NL63-related
Porcine-EDV
TGE
Beta-CoV cons
Alpha-CoV cons
Combined cons
Alignment qual

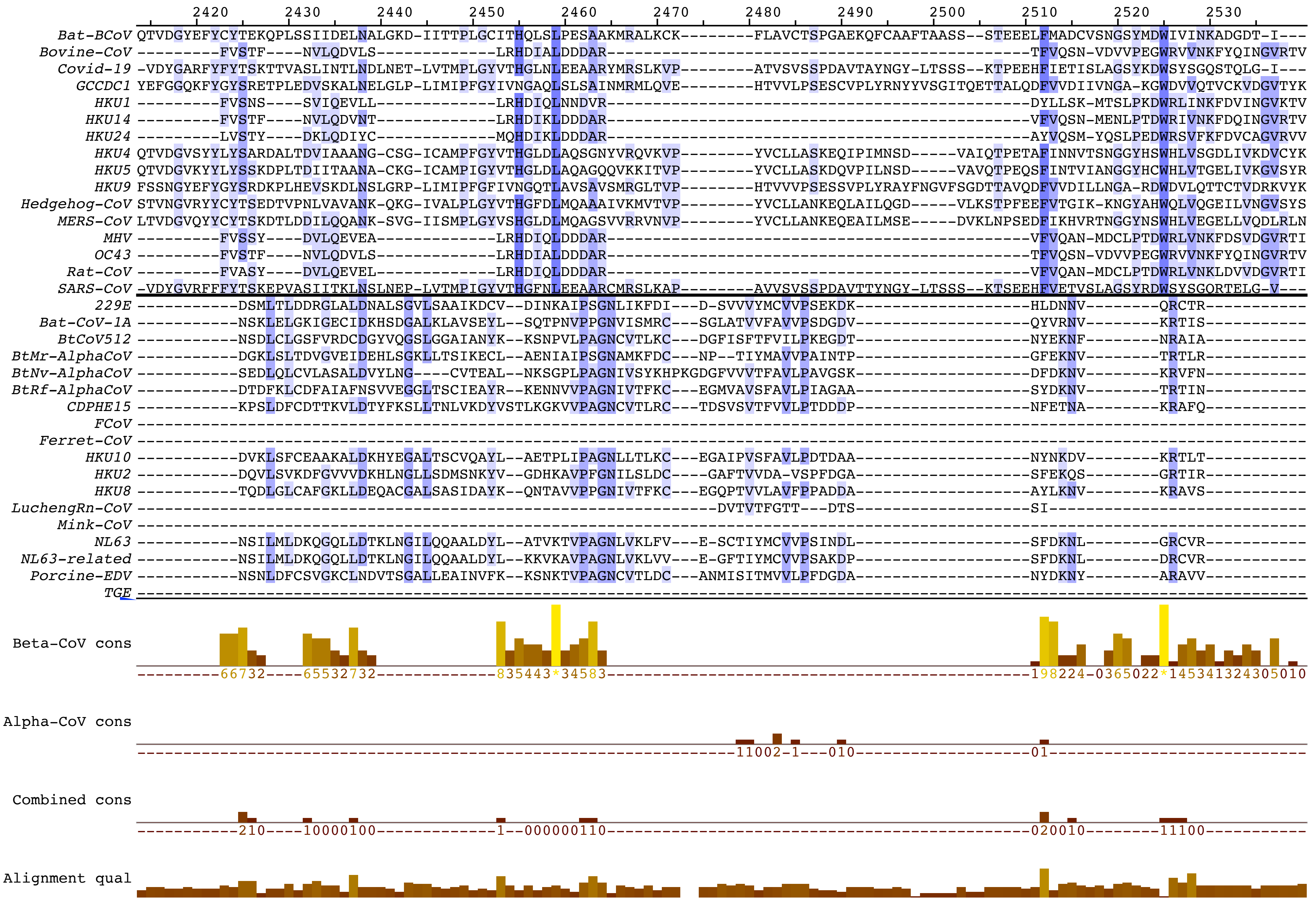
2420 2430 2440 2450 2460 2470 2480 2490 2500 2510 2520 2530
Bat-BCoV QTVDGYEFYCYTEKQPLSSIIDELNALGKD-IITTPLGCITHQLSLPESAAKMRALKCK--------FLAVCTSPGAEKQFCAAFTAASS--STEEELFMADCVSNGSYMDWIVINKADGDT-I---
Bovine-CoV ---------FVSTF----NVLQDVLS-------------LRHDIALDDDAR--TFVQSN-VDVVPEGWRVVNKFYQINGVRTV
Covid-19 -VDYGARFYFYTSKTTVASLINTLNDLNET-LVTMPLGYVTHGLNLEEAARYMRSLKVP--------ATVSVSSPDAVTAYNGY-LTSSS--KTPEEHFIETISLAGSYKDWSYSGQSTQLG-I---
GCCDC1 YEFGGQKFYGYSRETPLEDVSKALNELGLP-LIMIPFGYIVNGAQLSLSAINMRMLQVE--------HTVVLPSESCVPLYRNYYVSGITQETTALQDFVVDIIVNGA-KGWDVVQTVCKVDGVTYK
HKU1 ---------FVSNS----SVIQEVLL-------------LRHDIQLNNDVR--DYLLSK-MTSLPKDWRLINKFDVINGVKTV
HKU14 ---------FVSTF----NVLQDVNT-------------LRHDIKLDDDAR--VFVQSN-MENLPTDWRIVNKFDQINGVRTV
HKU24 ---------LVSTY----DKLQDIYC-------------MQHDIKLDDDAR--AYVQSM-YQSLPEDWRSVFKFDVCAGVRVV
HKU4 QTVDGVSYYLYSARDALTDVIAAANG-CSG-ICAMPFGYVTHGLDLAQSGNYVRQVKVP--------YVCLLASKEQIPIMNSD-----VAIQTPETAFINNVTSNGGYHSWHLVSGDLIVKDVCYK
HKU5 QTVDGVKYYLYSSKDPLTDIITAANA-CKG-ICAMPIGYVTHGLDLAQAGQQVKKITVP--------YVCLLASKDQVPILNSD-----VAVQTPEQSFINTVIANGGYHCWHLVTGELIVKGVSYR
HKU9 FSSNGYEFYGYSRDKPLHEVSKDLNSLGRP-LIMIPFGFIVNGQTLAVSAVSMRGLTVP--------HTVVVPSESSVPLYRAYFNGVFSGDTTAVQDFVVDILLNGA-RDWDVLQTTCTVDRKVYK
Hedgehog-CoV STVNGVRYYCYTSEDTVPNLVAVANK-QKG-IVALPLGYVTHGFDLMQAAAIVKMVTVP--------YVCLLANKEQLAILQGD-----VLKSTPFEEFVTGIK-KNGYAHWQLVQGEILVNGVSYS
MERS-CoV LTVDGVQYYCYTSKDTLDDILQQANK-SVG-IISMPLGYVSHGLDLMQAGSVVRRVNVP--------YVCLLANKEQEAILMSE-----DVKLNPSEDFIKHVRTNGGYNSWHLVEGELLVQDLRLN
MHV ---------FVSSY----DVLQEVEA-------------LRHDIQLDDDAR--VFVQAN-MDCLPTDWRLVNKFDSVDGVRTI
OC43 ---------FVSTF----NVLQDVLS-------------LRHDIALDDDAR--TFVQSN-VDVVPEGWRVVNKFYQINGVRTV
Rat-CoV ---------FVASY----DVLQEVEL-------------LRHDIQLDDDAR--VFVQAN-MDCLPTDWRLVNKLDVVDGVRTI
SARS-CoV -VDYGVRFFFYTSKEPVASIITKLNSLNEP-LVTMPIGYVTHGFNLEEAARCMRSLKAP--------AVVSVSSPDAVTTYNGY-LTSSS--KTSEEHFVETVSLAGSYRDWSYSGQRTELG-V---
229E -----------DSMLTLDDRGLALDNALSGVLSAAIKDCV--DINKAIPSGNLIKFDI---D-SVVVYMCVVPSEKDK-------------------HLDNNV--------QRCTR-----------
Bat-CoV-1A -----------NSKLELGKIGECIDKHSDGALKLAVSEYL--SQTPNVPPGNVISMRC---SGLATVVFAVVPSDGDV-------------------QYVRNV--------KRTIS-----------
BtCoV512 -----------NSDLCLGSFVRDCDGYVQGSLGGAIANYK--KSNPVLPAGNCVTLKC---DGFISFTFVILPKEGDT-------------------NYEKNF--------NRAIA-----------
BtMr-AlphaCoV -----------DGKLSLTDVGVEIDEHLSGKLLTSIKECL--AENIAIPSGNAMKFDC---NP--TIYMAVVPAINTP-------------------GFEKNV--------TRTLR-----------
BtNv-AlphaCoV -----------SEDLQLCVLASALDVYLNG----CVTEAL--NKSGPLPAGNIVSYKHPKGDGFVVVTFAVLPAVGSK-------------------DFDKNV--------KRVFN-----------
BtRf-AlphaCoV -----------DTDFKLCDFAIAFNSVVEGGLTSCIEAYR--KENNVVPAGNIVTFKC---EGMVAVSFAVLPIAGAA-------------------SYDKNV--------TRTIN-----------
CDPHE15 -----------KPSLDFCDTTKVLDTYFKSLLTNLVKDYVSTLKGKVVPAGNCVTLRC---TDSVSVTFVVLPTDDDP-------------------NFETNA--------KRAFQ-----------
FCoV ---
Ferret-CoV ---
HKU10 -----------DVKLSFCEAAKALDKHYEGALTSCVQAYL--AETPLIPAGNLLTLKC---EGAIPVSFAVLPDTDAA-------------------NYNKDV--------KRTLT-----------
HKU2 -----------DQVLSVKDFGVVVDKHLNGLLSDMSNKYV--GDHKAVPFGNILSLDC----GAFTVVDA-VSPFDGA-------------------SFEKQS--------GRTIR-----------
HKU8 -----------TQDLGLCAFGKLLDEQACGALSASIDAYK--QNTAVVPPGNIVTFKC---EGQPTVVLAVFPPADDA-------------------AYLKNV--------KRAVS-----------
LuchengRn-CoV ---DVTVTFGTT---DTS-------------------SI----------------------------
Mink-CoV ---
NL63 -----------NSILMLDKQGQLLDTKLNGILQQAALDYL--ATVKTVPAGNLVKLFV---E-SCTIYMCVVPSINDL-------------------SFDKNL--------GRCVR-----------
NL63-related -----------NSILMLDKQGQLLDTKLNGILQQAALDYL--KKVKAVPAGNLVKLVV---E-GFTIYMCVVPSAKDP-------------------SFDKNL--------DRCVR-----------
Porcine-EDV -----------NSNLDFCSVGKCLNDVTSGALLEAINVFK--KSNKTVPAGNCVTLDC---ANMISITMVVLPFDGDA-------------------NYDKNY--------ARAVV-----------
TGE ---
Beta-CoV cons
---------66732----65532732-------------835443*34583--198224-0365022*145341324305010
Alpha-CoV cons
---11002-1---010-------------------01----------------------------
Combined cons
-----------210----10000100-------------1--000000110--020010--------11100-----------
Alignment qual

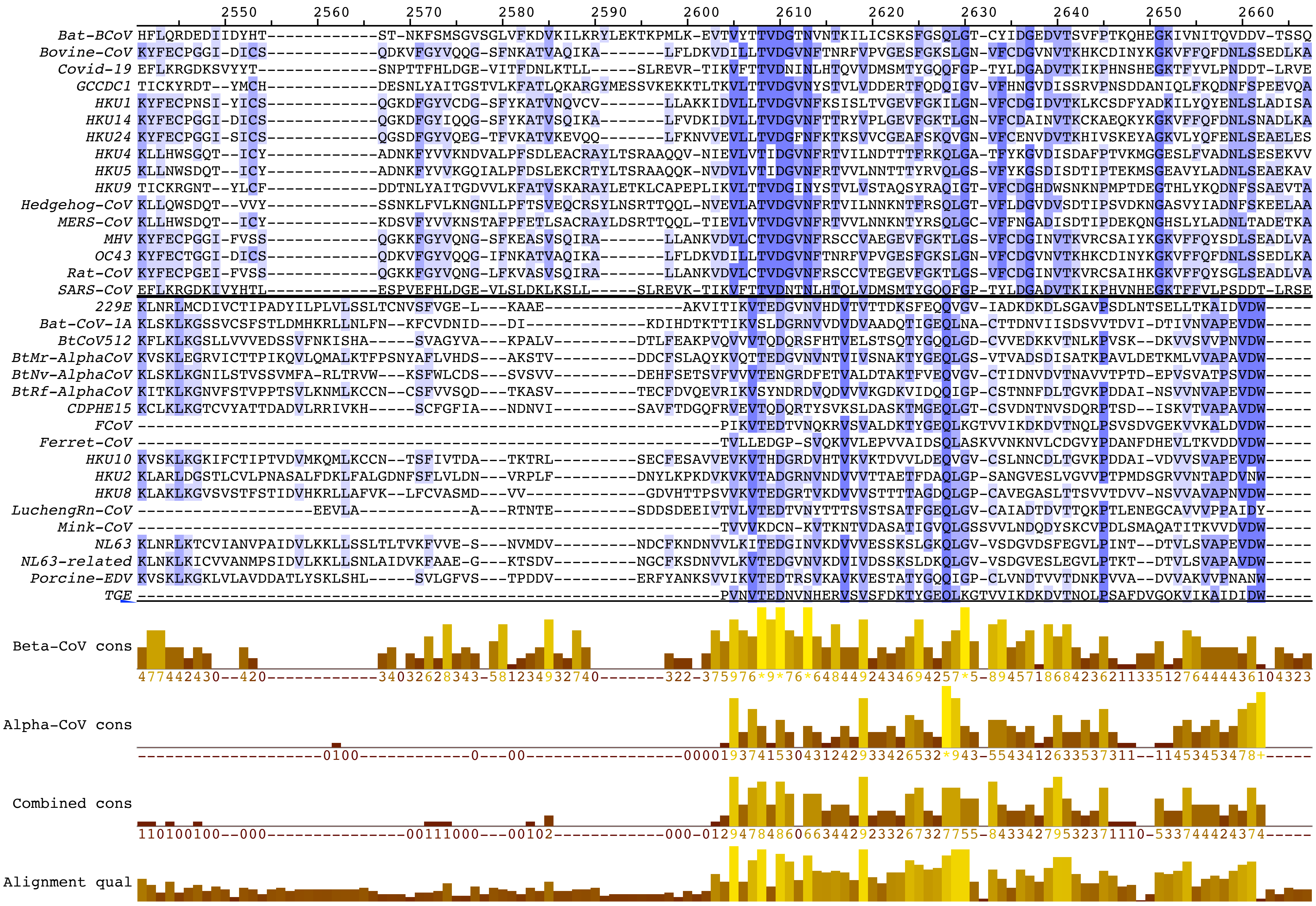

2550
2560
2570
2580
2590
2600
2610
2620
2630
2640
2650
2660
Bat-BCoV
Bovine-CoV
Covid-19
GCCDC1
HKU1
HKU14
HKU24
HKU4
HKU5
HKU9
Hedgehog-CoV
MERS-CoV
MHV
OC43
Rat-CoV
SARS-CoV
229E
Bat-CoV-1A
BtCoV512
BtMr-AlphaCoV
BtNv-AlphaCoV
BtRf-AlphaCoV
CDPHE15
FCoV
Ferret-CoV
HKU10
HKU2
HKU8
LuchengRn-CoV
Mink-CoV
NL63
NL63-related
Porcine-EDV
TGE
Beta-CoV cons
Alpha-CoV cons
Combined cons
Alignment qual

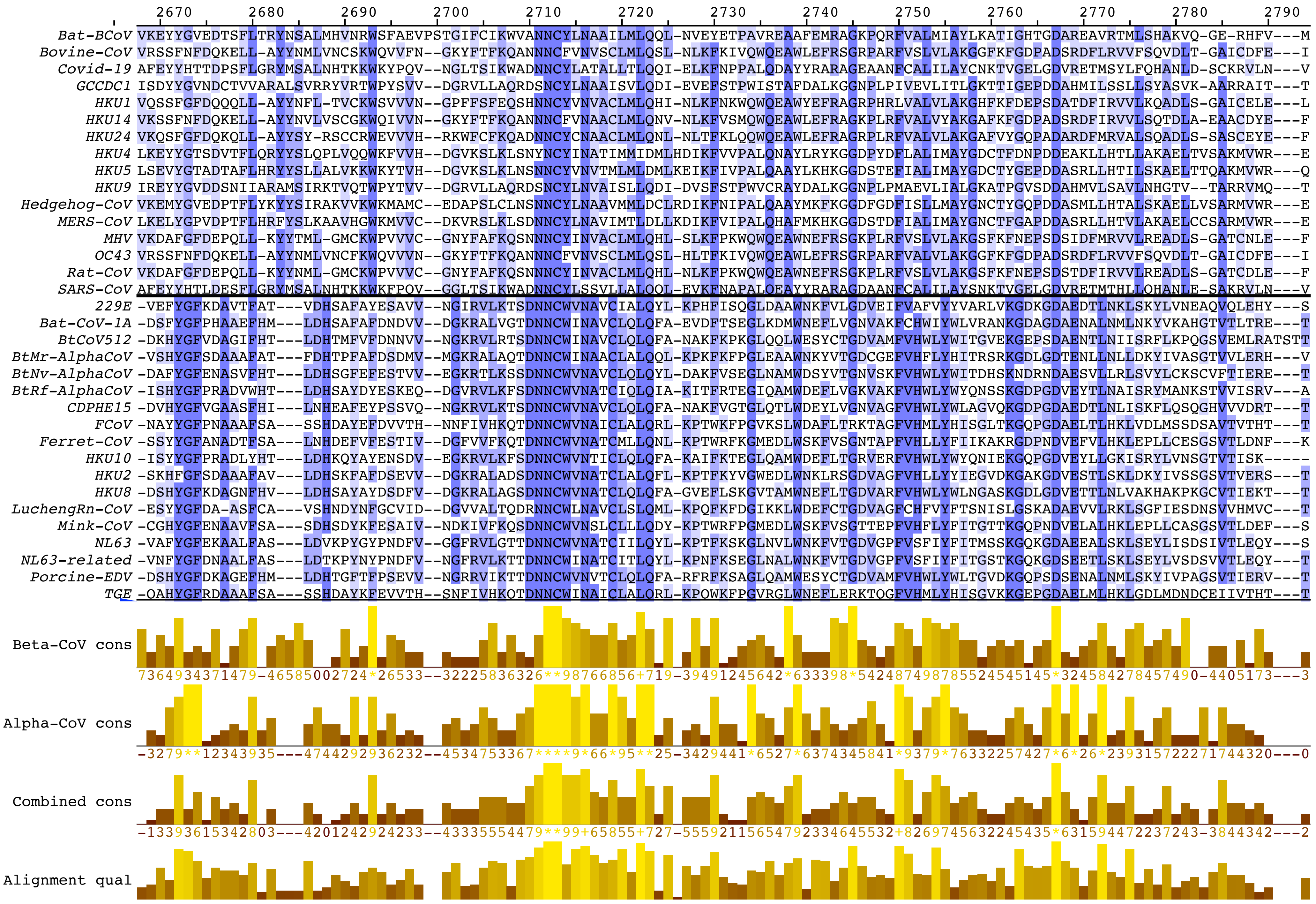

2670
2680
2690
2700
2710
2720
2730
2740
2750
2760
2770
2780
2790
Bat-BCoV
Bovine-CoV
Covid-19
GCCDC1
HKU1
HKU14
HKU24
HKU4
HKU5
HKU9
Hedgehog-CoV
MERS-CoV
MHV
OC43
Rat-CoV
SARS-CoV
229E
Bat-CoV-1A
BtCoV512
BtMr-AlphaCoV
BtNv-AlphaCoV
BtRf-AlphaCoV
CDPHE15
FCoV
Ferret-CoV
HKU10
HKU2
HKU8
LuchengRn-CoV
Mink-CoV
NL63
NL63-related
Porcine-EDV
TGE
Beta-CoV cons
Alpha-CoV cons
Combined cons
Alignment qual

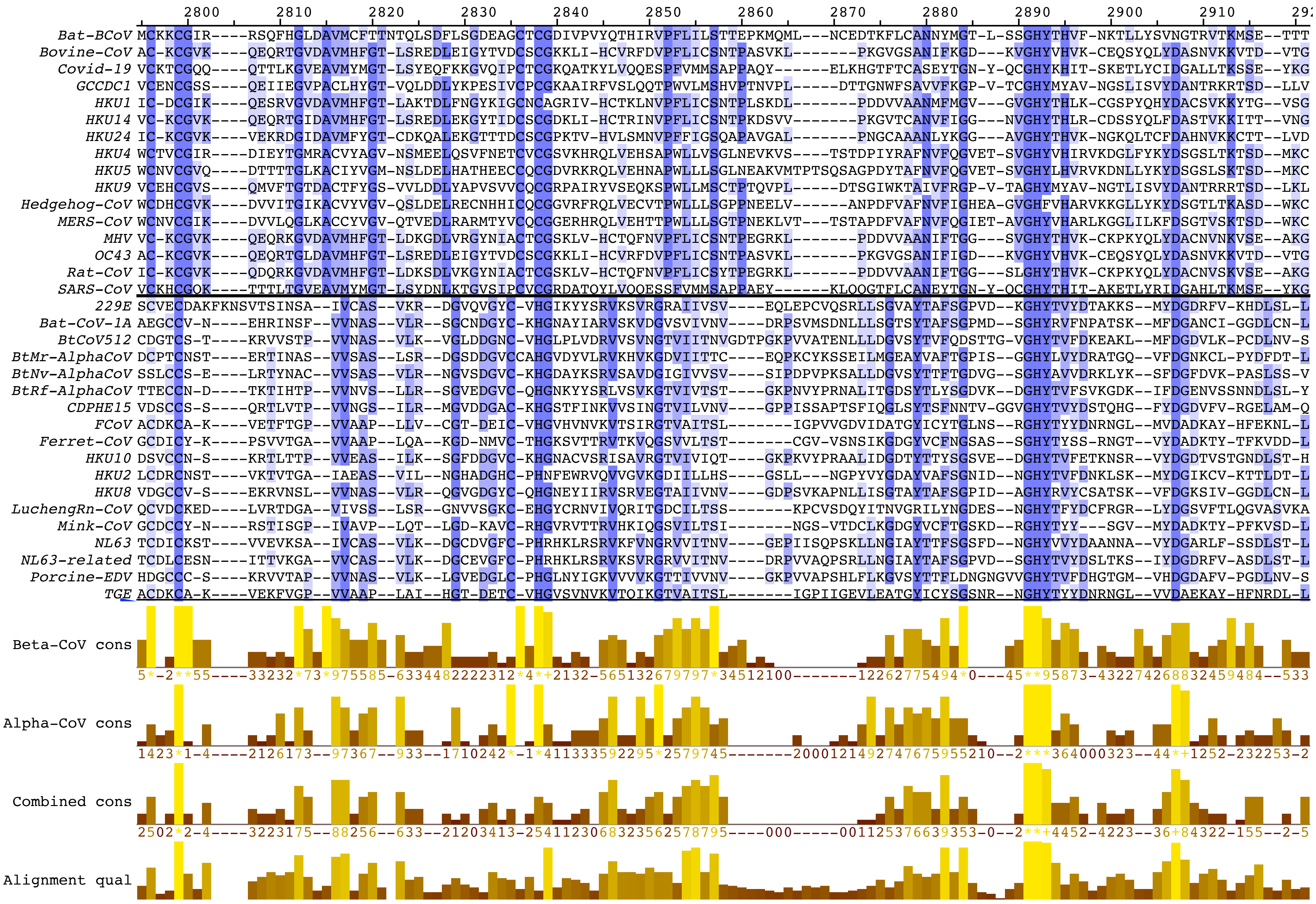
2800
2810
2820
2830
2840
2850
2860
2870
2880
2890
2900
2910
Bat-BCoV
Bovine-CoV
Covid-19
GCCDC1
HKU1
HKU14
HKU24
HKU4
HKU5
HKU9
Hedgehog-CoV
MERS-CoV
MHV
OC43
Rat-CoV
SARS-CoV
229E
Bat-CoV-1A
BtCoV512
BtMr-AlphaCoV
BtNv-AlphaCoV
BtRf-AlphaCoV
CDPHE15
FCoV
Ferret-CoV
HKU10
HKU2
HKU8
LuchengRn-CoV
Mink-CoV
NL63
NL63-related
Porcine-EDV
TGE
Beta-CoV cons
Alpha-CoV cons
Combined cons
Alignment qual

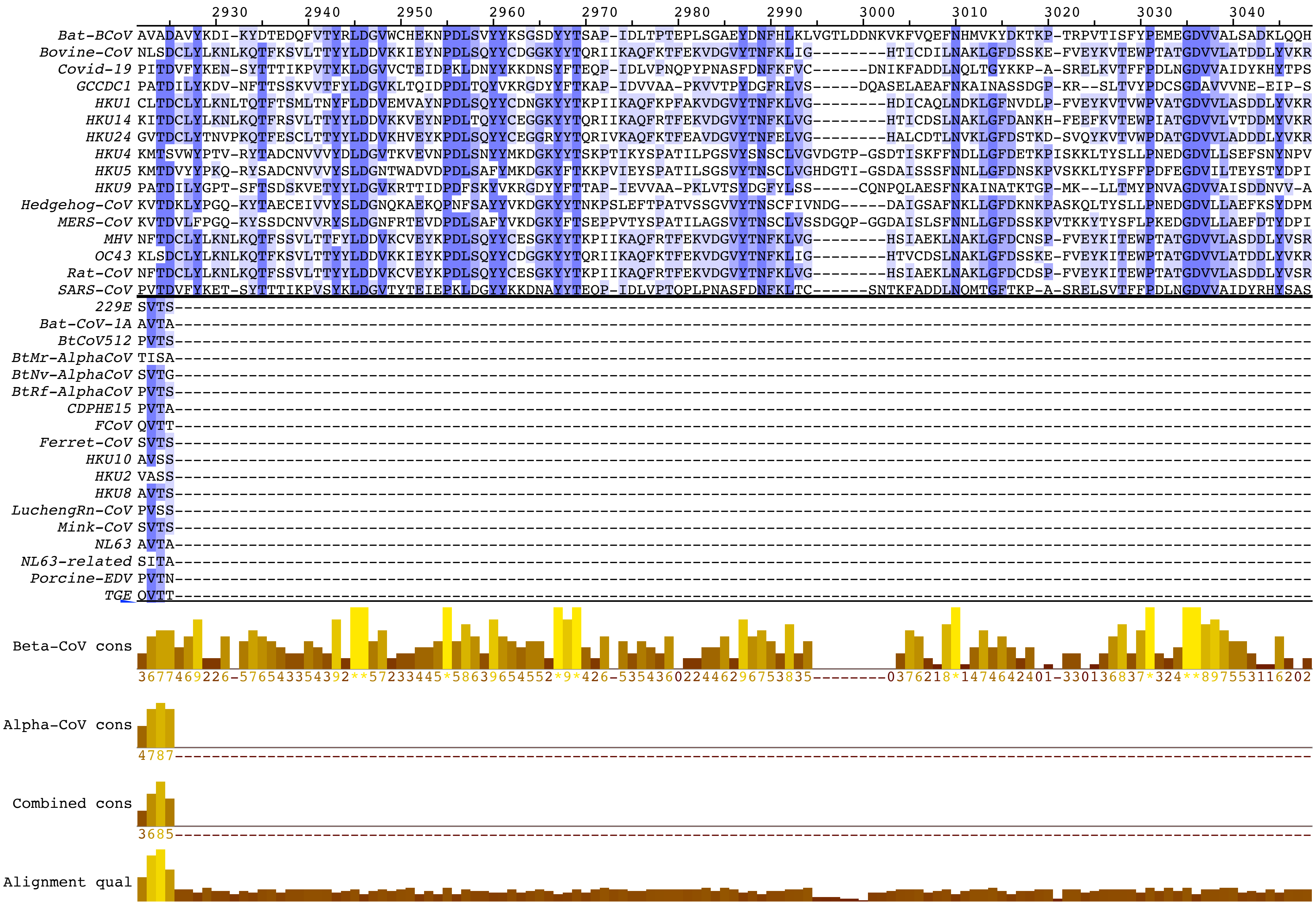
2930
2940
2950
2960
2970
2980
2990
3000
3010
3020
3030
3040
Bat-BCoV
Bovine-CoV
Covid-19
GCCDC1
HKU1
HKU14
HKU24
HKU4
HKU5
HKU9
Hedgehog-CoV
MERS-CoV
MHV
OC43
Rat-CoV
SARS-CoV
229E
Bat-CoV-1A
BtCoV512
BtMr-AlphaCoV
BtNv-AlphaCoV
BtRf-AlphaCoV
CDPHE15
FCoV
Ferret-CoV
HKU10
HKU2
HKU8
LuchengRn-CoV
Mink-CoV
NL63
NL63-related
Porcine-EDV
TGE
Beta-CoV cons
Alpha-CoV cons
Combined cons
Alignment qual

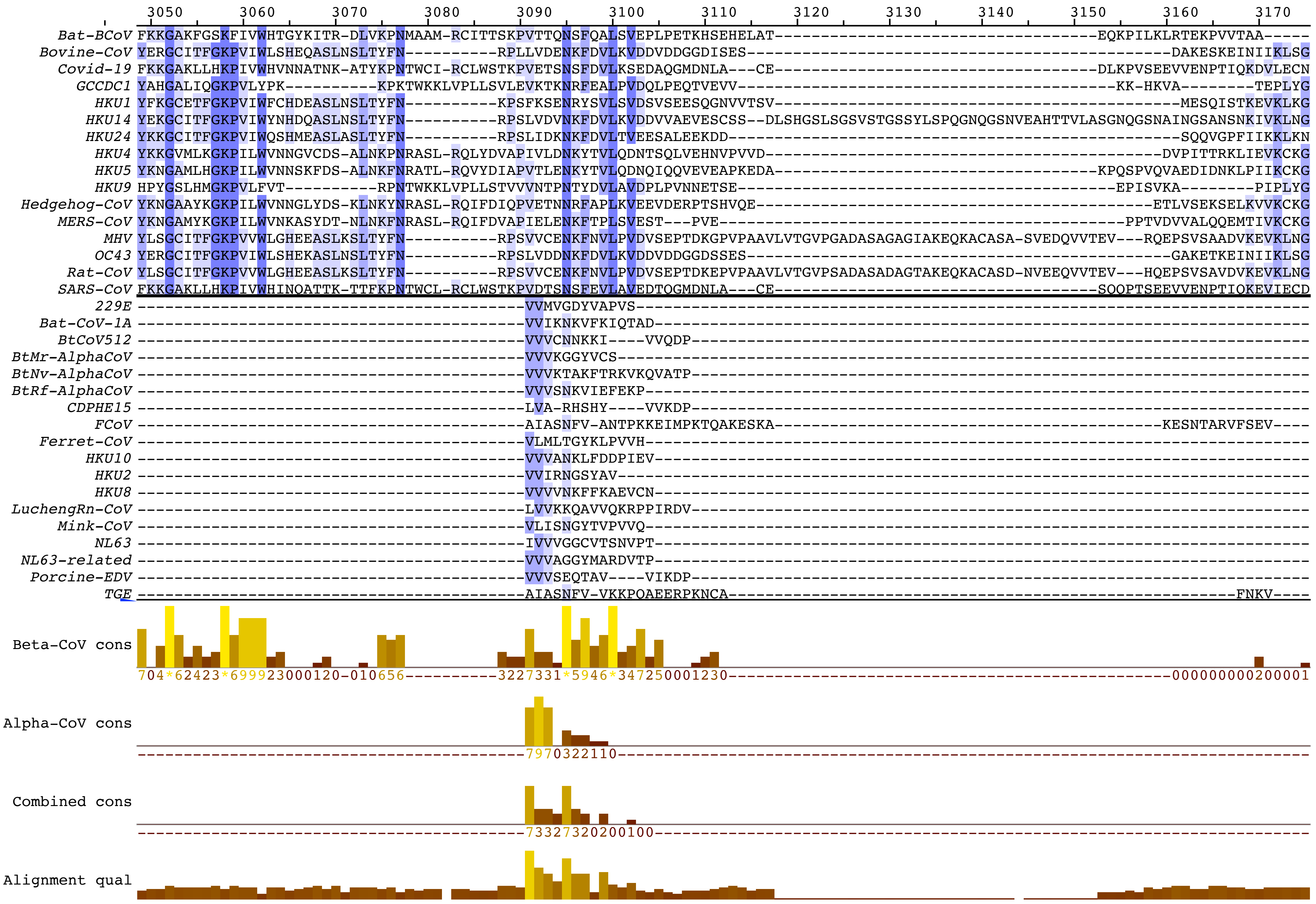

3050 3060 3070 3080 3090 3100 3110 3120 3130 3140 3150 3160 3170
Bat-BCoV
Bovine-CoV
Covid-19
GCCDC1
HKU1
HKU14
HKU24
HKU4
HKU5
HKU9
Hedgehog-CoV
MERS-CoV
MHV
OC43
Rat-CoV
SARS-CoV
229E
Bat-CoV-1A
BtCoV512
BtMr-AlphaCoV
BtNv-AlphaCoV
BtRf-AlphaCoV
CDPHE15
FCoV
Ferret-CoV
HKU10
HKU2
HKU8
LuchengRn-CoV
Mink-CoV
NL63
NL63-related
Porcine-EDV
TGE
Beta-CoV cons
Alpha-CoV cons
Combined cons
Alignment qual

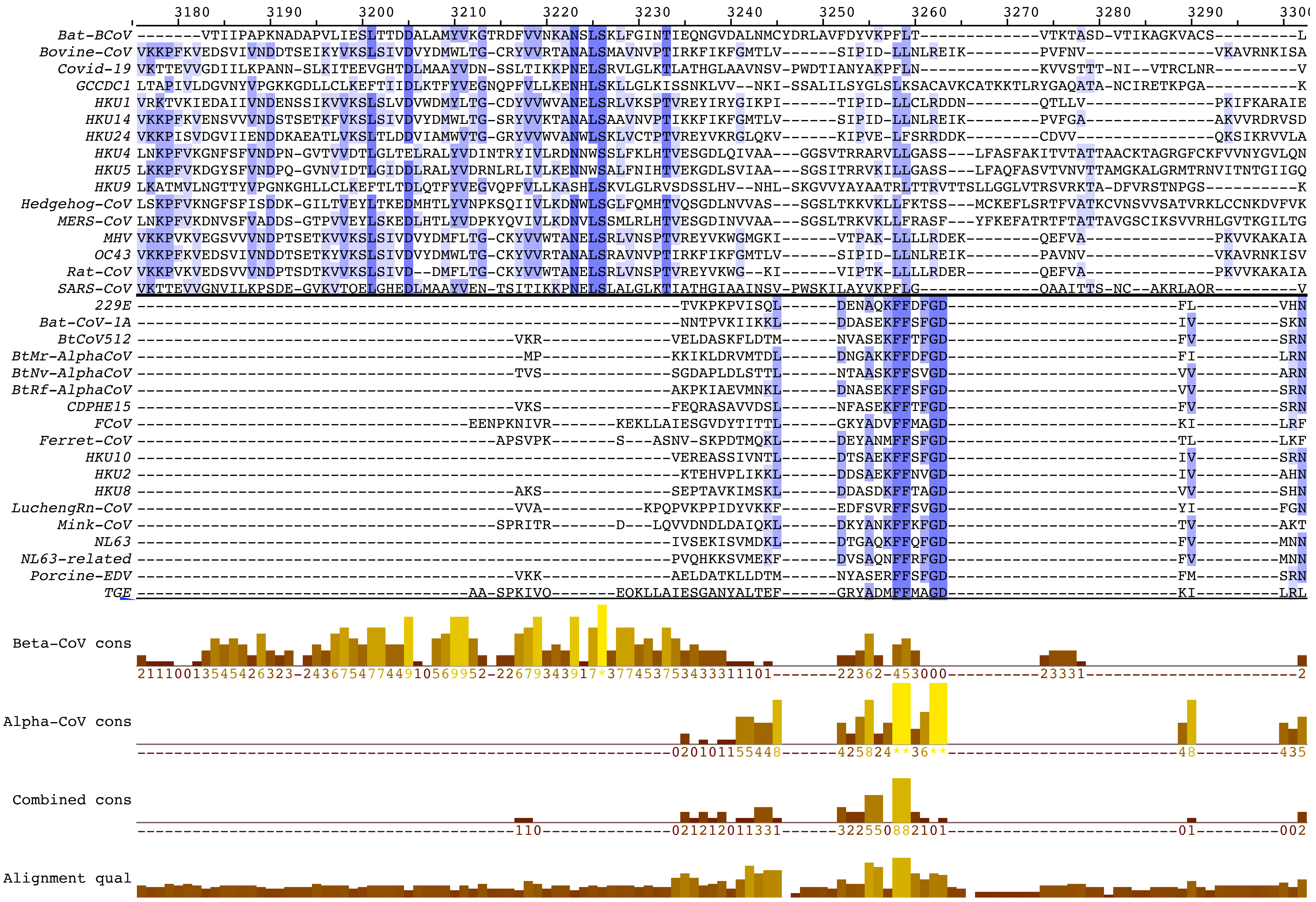

3180 3190 3200 3210 3220 3230 3240 3250 3260 3270 3280 3290 3300
Bat-BCoV -------VTIIPAPKNADAPVLIESLTTDDALAMYVKGTRDFVVNKANSLSKLFGINTIEQNGVDALNMCYDRLAVFDYVKPFLT-------------VTKTASD-VTIKAGKVACS---------L
Bovine-CoV VKKPFKVEDSVIVNDDTSEIKYVKSLSIVDVYDMWLTG-CRYVVRTANALSMAVNVPTIRKFIKFGMTLV------SIPID-LLNLREIK--------PVFNV---------------VKAVRNKISA
Covid-19 VKTTEVVGDIILKPANN-SLKITEEVGHTDLMAAYVDN-SSLTIKKPNELSRVLGLKTLATHGLAAVNSV-PWDTIANYAKPFLN-------------KVVSTTT-NI--VTRCLNR---------V
GCCDC1 LTAPIVLDGVNYVPGKKGDLLCLKEFTIIDLKTFYVEGNQPFVLLKENHLSKLLGLKISSNKLVV--NKI-SSALILSYGLSLKSACAVKCATKKTLRYGAQATA-NCIRETKPGA----------K
HKU1 VRKTVKIEDAIIVNDENSSIKVVKSLSLVDVWDMYLTG-CDYVVWVANELSRLVKSPTVREYIRYGIKPI------TIPID-LLCLRDDN--------QTLLV---------------PKIFKARAIE
HKU14 VKKPFKVENSVVVNDSTSETKFVKSLSIVDVYDMWLTG-SRYVVKTANALSAAVNVPTIKKFIKFGMTLV------SIPID-LLNLREIK--------PVFGA---------------AKVVRDRVSD
HKU24 VKKPISVDGVIIENDDKAEATLVKSLTLDDVIAMWVTG-GRYVVWVANWLSKLVCTPTVREYVKRGLQKV------KIPVE-LFSRRDDK--------CDVV----------------QKSIKRVVLA
HKU4 LNKPFVVKGNFSFVNDPN-GVTVVDTLGLTELRALYVDINTRYIVLRDNNWSSLFKLHTVESGDLQIVAA---GGSVTRRARVLLGASS---LFASFAKITVTATTAACKTAGRGFCKFVVNYGVLQN
HKU5 LKKPFVVKDGYSFVNDPQ-GVNVIDTLGIDDLRALYVDRNLRLIVLKENNWSALFNIHTVEKGDLSVIAA---SGSITRRVKILLGASS---LFAQFASVTVNVTTAMGKALGRMTRNVITNTGIIGQ
HKU9 LKATMVLNGTTYVPGNKGHLLCLKEFTLTDLQTFYVEGVQPFVLLKASHLSKVLGLRVSDSSLHV--NHL-SKGVVYAYAATRLTTRVTTSLLGGLVTRSVRKTA-DFVRSTNPGS----------K
Hedgehog-CoV LSKPFVVKNGFSFISDDK-GILTVEYLTKEDMHTLYVNPKSQIIVLKDNWLSGLFQMHTVQSGDLNVVAS---SGSLTKKVKLLFKTSS---MCKEFLSRTFVATKCVNSVVSATVRKLCCNKDVFVK
MERS-CoV LNKPFVVKDNVSFVADDS-GTPVVEYLSKEDLHTLYVDPKYQVIVLKDNVLSSMLRLHTVESGDINVVAA---SGSLTRKVKLLFRASF---YFKEFATRTFTATTAVGSCIKSVVRHLGVTKGILTG
MHV VKKPVKVEGSVVVNDPTSETKVVKSLSIVDVYDMFLTG-CKYVVWTANELSRLVNSPTVREYVKWGMGKI------VTPAK-LLLLRDEK--------QEFVA---------------PKVVKAKAIA
OC43 VKKPFKVEDSVIVNDDTSETKYVKSLSIVDVYDMWLTG-CKYVVRTANALSRAVNVPTIRKFIKFGMTLV------SIPID-LLNLREIK--------PAVNV---------------VKAVRNKISV
Rat-CoV VKKPVKVEDSVVVNDPTSDTKVVKSLSIVD--DMFLTG-CKYVVWTANELSRLVNSPTVREYVKWG--KI------VIPTK-LLLLRDER--------QEFVA---------------PKVVKAKAIA
SARS-CoV VKTTEVVGNVILKPSDE-GVKVTQELGHEDLMAAYVEN-TSITIKKPNELSLALGLKTIATHGIAAINSV-PWSKILAYVKPFLG-------------QAAITTS-NC--AKRLAQR---------V
229E ---TVKPKPVISQL------DENAQKFFDFGD--------------------------FL----------VHN
Bat-CoV-1A --NNTPVKIIKKL------DDASEKFFSFGD--------------------------IV----------SKN
BtCoV512 --VKR--------------VELDASKFLDTM------NVASEKFFTFGD--------------------------FV----------SRN
BtMr-AlphaCoV ---MP---------------KKIKLDRVMTDL------DNGAKKFFDFGD--------------------------FI----------LRN
BtNv-AlphaCoV --TVS---------------SGDAPLDLSTTL------NTAASKFFSVGD--------------------------VV----------ARN
BtRf-AlphaCoV ---AKPKIAEVMNKL------DNASEKFFSFGD--------------------------VV----------SRN
CDPHE15 --VKS---------------FEQRASAVVDSL------NFASEKFFTFGD--------------------------FV----------SRN
FCoV ------------------------------------EENPKNIVR-------KEKLLAIESGVDYTITTL------GKYADVFFMAGD--------------------------KI----------LRF
Ferret-CoV --------------------------------------APSVPK-------S---ASNV-SKPDTMQKL------DEYANMFFSFGD--------------------------TL----------LKF
HKU10 ---VEREASSIVNTL------DTSAEKFFSFGD--------------------------IV----------SRN
HKU2 --KTEHVPLIKKL------DDSAEKFFNVGD--------------------------IV----------AHN
HKU8 --AKS---------------SEPTAVKIMSKL------DDASDKFFTAGD--------------------------VV----------SHN
LuchengRn-CoV --VVA-----------KPQPVKPPIDYVKKF------EDFSVRFFSVGD--------------------------YI----------FGN
Mink-CoV --------------------------------------SPRITR-------D---LQVVDNDLDAIQKL------DKYANKFFKFGD--------------------------TV----------AKT
NL63 ---IVSEKISVMDKL------DTGAQKFFQFGD--------------------------FV----------MNN
NL63-related ---PVQHKKSVMEKF------DVSAQNFFRFGD--------------------------FV----------MNN
Porcine-EDV --VKK---------------AELDATKLLDTM------NYASERFFSFGD--------------------------FM----------SRN
TGE ------------------------------------AA-SPKIVQ-------EQKLLAIESGANYALTEF------GRYADMFFMAGD--------------------------KI----------LRL
Beta-CoV cons
211100135454262323-24367547744910569952-22679343917*377453753433311101-------22362-453000-----------23331-------------------------2
Alpha-CoV cons
---020101155448------425824**36**--------------------------48----------435
Combined cons
--110---------------021212011331------322550882101--------------------------01----------002
Alignment qual

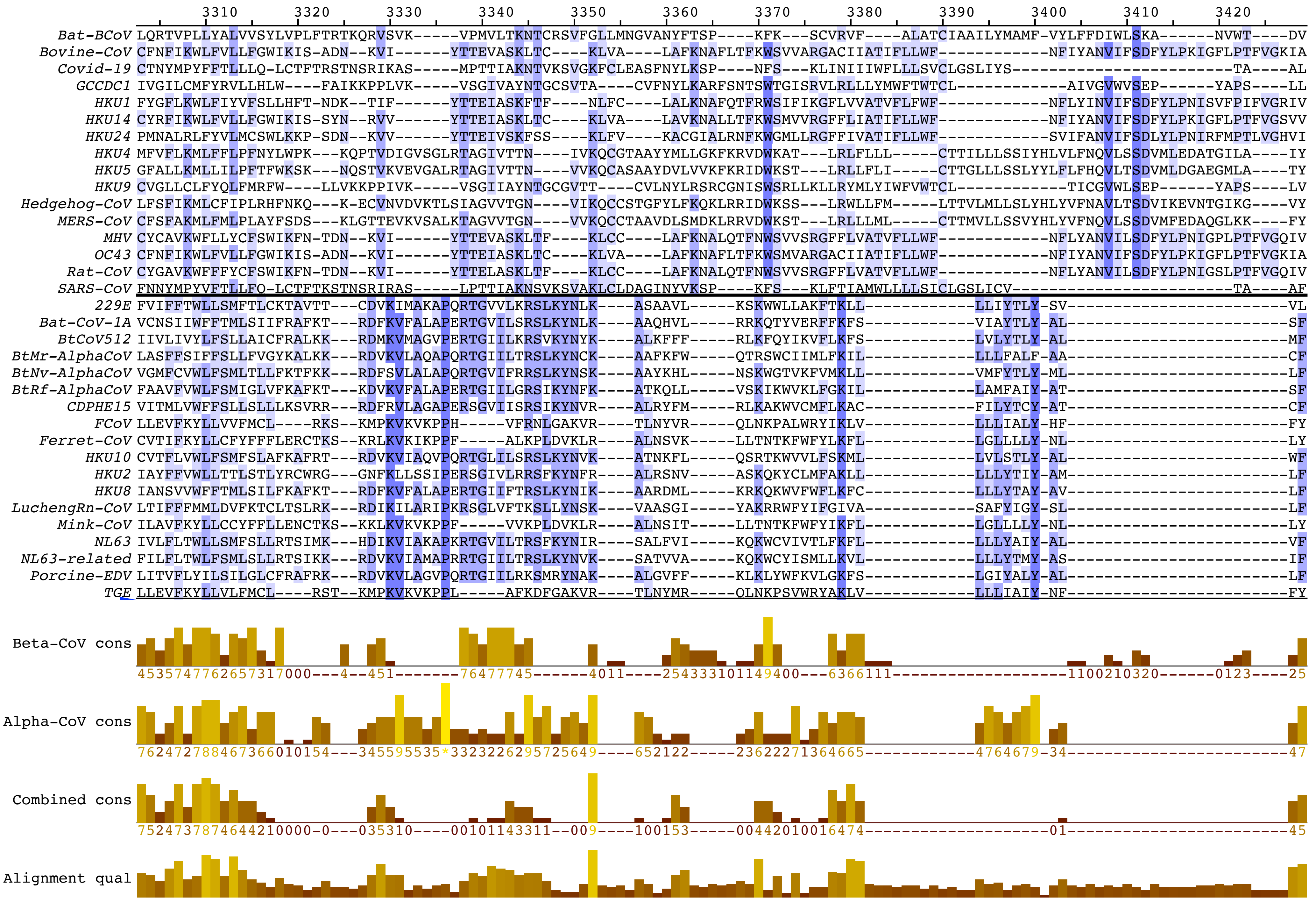

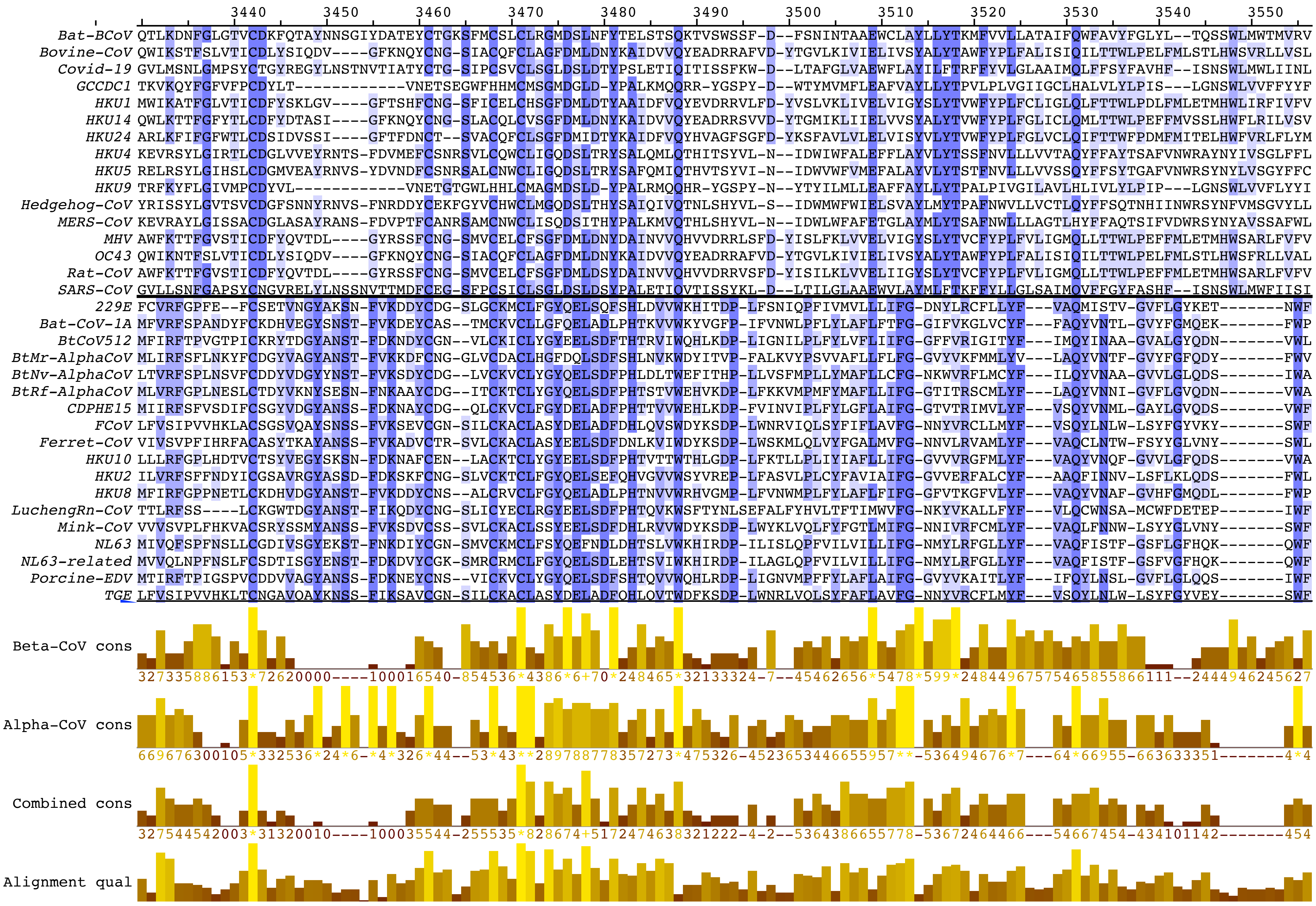
3440
3450
3460
3470
3480
3490
3500
3510
3520
3530
3540
3550
Bat-BCoV
Bovine-CoV
Covid-19
GCCDC1
HKU1
HKU14
HKU24
HKU4
HKU5
HKU9
Hedgehog-CoV
MERS-CoV
MHV
OC43
Rat-CoV
SARS-CoV
229E
Bat-CoV-1A
BtCoV512
BtMr-AlphaCoV
BtNv-AlphaCoV
BtRf-AlphaCoV
CDPHE15
FCoV
Ferret-CoV
HKU10
HKU2
HKU8
LuchengRn-CoV
Mink-CoV
NL63
NL63-related
Porcine-EDV
TGE
Beta-CoV cons
Alpha-CoV cons
Combined cons
Alignment qual

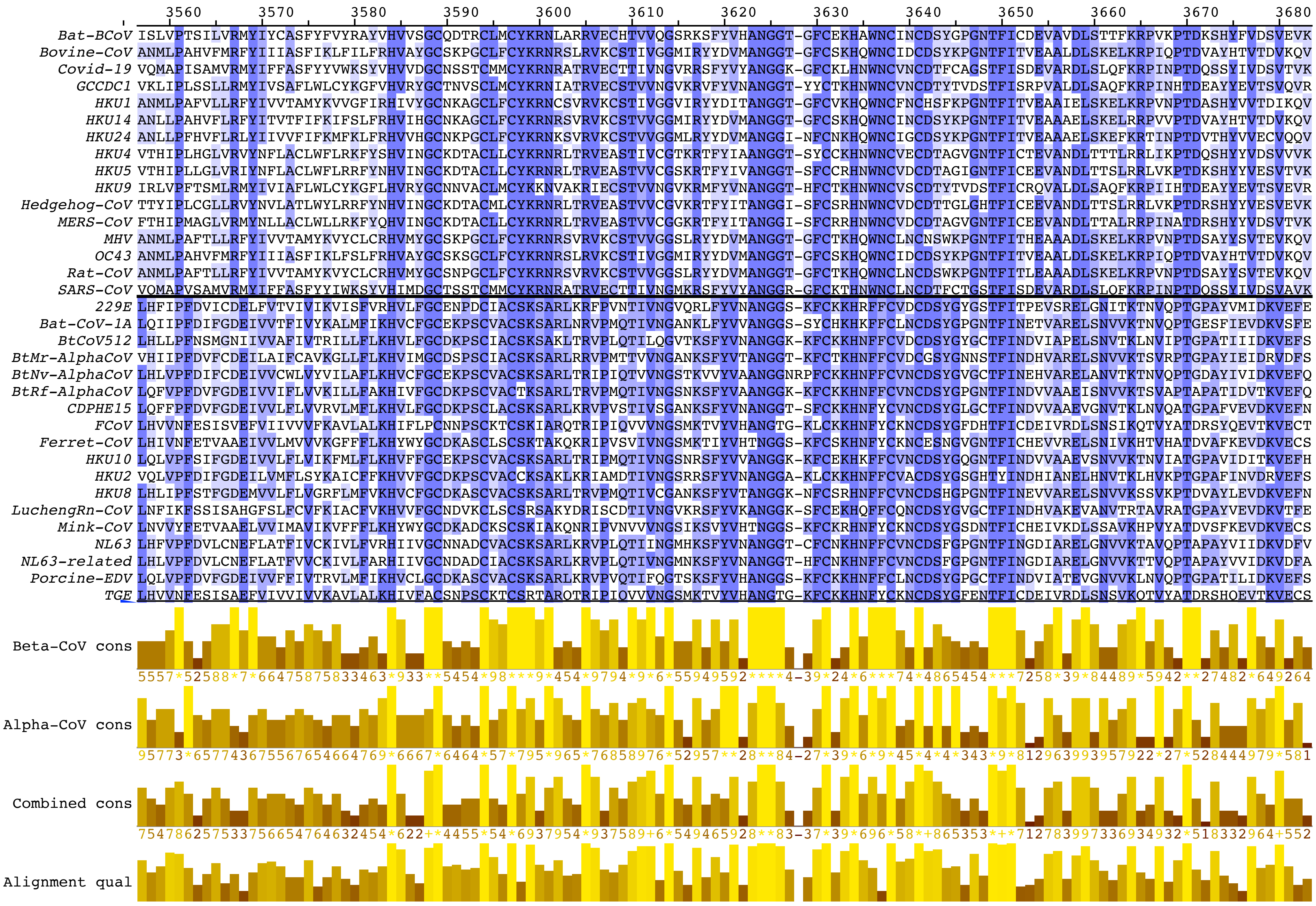

3560
3570
3580
3590
3600
3610
3620
3630
3640
3650
3660
3670
3680
Bat-BCoV
Bovine-CoV
Covid-19
GCCDC1
HKU1
HKU14
HKU24
HKU4
HKU5
HKU9
Hedgehog-CoV
MERS-CoV
MHV
OC43
Rat-CoV
SARS-CoV
229E
Bat-CoV-1A
BtCoV512
BtMr-AlphaCoV
BtNv-AlphaCoV
BtRf-AlphaCoV
CDPHE15
FCoV
Ferret-CoV
HKU10
HKU2
HKU8
LuchengRn-CoV
Mink-CoV
NL63
NL63-related
Porcine-EDV
TGE
Beta-CoV cons
Alpha-CoV cons
Combined cons
Alignment qual

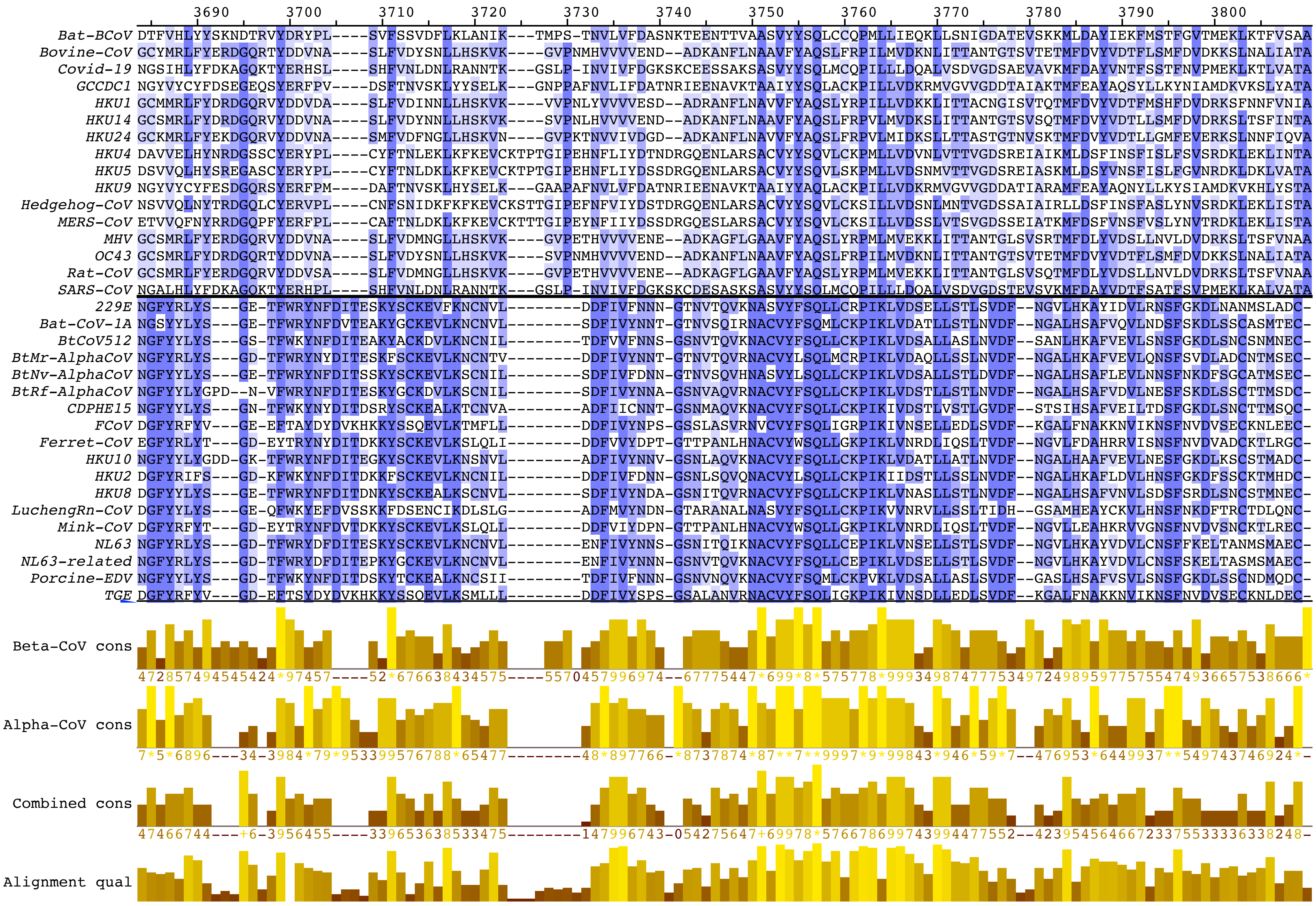

3690
3700
3710
3720
3730
3740
3750
3760
3770
3780
3790
3800
Bat-BCoV
Bovine-CoV
Covid-19
GCCDC1
HKU1
HKU14
HKU24
HKU4
HKU5
HKU9
Hedgehog-CoV
MERS-CoV
MHV
OC43
Rat-CoV
SARS-CoV
229E
Bat-CoV-1A
BtCoV512
BtMr-AlphaCoV
BtNv-AlphaCoV
BtRf-AlphaCoV
CDPHE15
FCoV
Ferret-CoV
HKU10
HKU2
HKU8
LuchengRn-CoV
Mink-CoV
NL63
NL63-related
Porcine-EDV
TGE
Beta-CoV cons
Alpha-CoV cons
Combined cons
Alignment qual

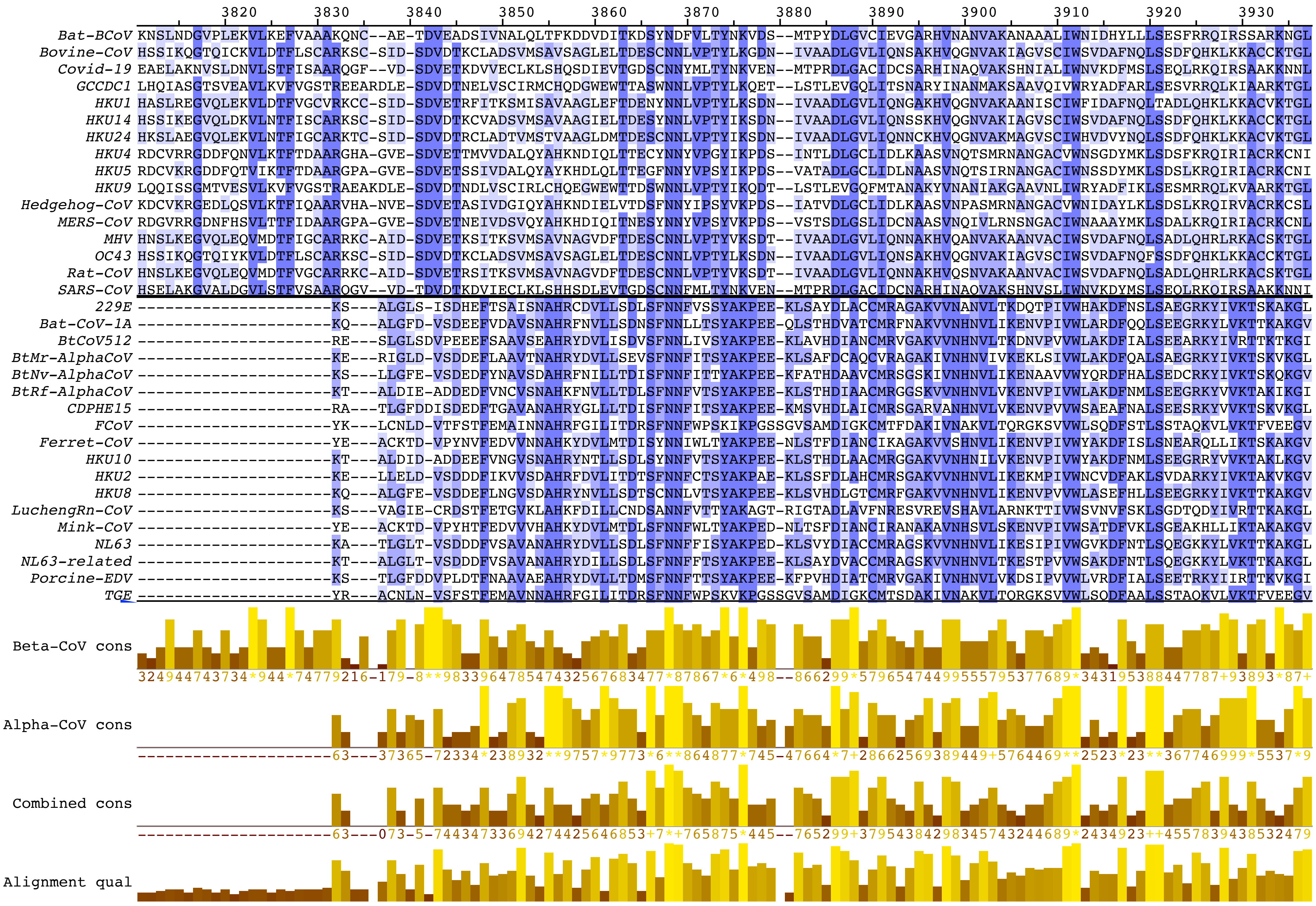
3820 3830 3840 3850 3860 3870 3880 3890 3900 3910 3920 3930
Bat-BCoV KNSLNDGVPLEKVLKEFVAAAKQNC--AE-TDVEADSIVNALQLTFKDDVDITKDSYNDFVLTYNKVDS--MTPYDLGVCIEVGARHVNANVAKANAAALIWNIDHYLLLSESFRRQIRSSARKNGL
Bovine-CoV HSSIKQGTQICKVLDTFLSCARKSC-SID-SDVDTKCLADSVMSAVSAGLELTDESCNNLVPTYLKGDN--IVAADLGVLIQNSAKHVQGNVAKIAGVSCIWSVDAFNQLSSDFQHKLKKACCKTGL
Covid-19 EAELAKNVSLDNVLSTFISAARQGF--VD-SDVETKDVVECLKLSHQSDIEVTGDSCNNYMLTYNKVEN--MTPRDLGACIDCSARHINAQVAKSHNIALIWNVKDFMSLSEQLRKQIRSAAKKNNL
GCCDC1 LHQIASGTSVEAVLKVFVGSTREEARDLE-SDVDTNELVSCIRMCHQDGWEWTTASWNNLVPTYLKQET--LSTLEVGQLITSNARYINANMAKSAAVQIVWRYADFARLSESVRRQLKIAARKTGL
HKU1 HASLREGVQLEKVLDTFVGCVRKCC-SID-SDVETRFITKSMISAVAAGLEFTDENYNNLVPTYLKSDN--IVAADLGVLIQNGAKHVQGNVAKAANISCIWFIDAFNQLTADLQHKLKKACVKTGL
HKU14 HSSIKEGVQLDKVLNTFISCARKSC-SID-SDVDTKCVADSVMSAVAAGIELTDESYNNLVPTYIKSDN--IVAADLGVLIQNSSKHVQGNVAKIAGVSCIWSVDAFNQLSSDFQHKLKKACCKTGL
HKU24 HKSLAEGVQLEKVLNTFIGCARKTC-SID-SDVDTRCLADTVMSTVAAGLDMTDESCNNLVPTYIKSDN--IVAADLGVLIQNNCKHVQGNVAKMAGVSCIWHVDVYNQLSSDFQHKLKKACVKTGL
HKU4 RDCVRRGDDFQNVLKTFTDAARGHA-GVE-SDVETTMVVDALQYAHKNDIQLTTECYNNYVPGYIKPDS--INTLDLGCLIDLKAASVNQTSMRNANGACVWNSGDYMKLSDSFKRQIRIACRKCNI
HKU5 RDCVKRGDDFQTVIKTFTDAARGPA-GVE-SDVETSSIVDALQYAYKHDLQLTTEGFNNYVPSYIKPDS--VATADLGCLIDLNAASVNQTSIRNANGACIWNSSDYMKLSDSLKRQIRIACRKCNI
HKU9 LQQISSGMTVESVLKVFVGSTRAEAKDLE-SDVDTNDLVSCIRLCHQEGWEWTTDSWNNLVPTYIKQDT--LSTLEVGQFMTANAKYVNANIAKGAAVNLIWRYADFIKLSESMRRQLKVAARKTGL
Hedgehog-CoV KDCVKRGEDLQSVLKTFIQAARVHA-NVE-SDVETASIVDGIQYAHKNDIELVTDSFNNYIPSYVKPDS--IATVDLGCLIDLKAASVNPASMRNANGACVWNIDAYLKLSDSLKRQIRVACRKCSL
MERS-CoV RDGVRRGDNFHSVLTTFIDAARGPA-GVE-SDVETNEIVDSVQYAHKHDIQITNESYNNYVPSYVKPDS--VSTSDLGSLIDCNAASVNQIVLRNSNGACIWNAAAYMKLSDALKRQIRIACRKCNL
MHV HNSLKEGVQLEQVMDTFIGCARRKC-AID-SDVETKSITKSVMSAVNAGVDFTDESCNNLVPTYVKSDT--IVAADLGVLIQNNAKHVQANVAKAANVACIWSVDAFNQLSADLQHRLRKACSKTGL
OC43 HSSIKQGTQIYKVLDTFLSCARKSC-SID-SDVDTKCLADSVMSAVSAGLELTDESCNNLVPTYLKSDN--IVAADLGVLIQNSAKHVQGNVAKIAGVSCIWSVDAFNQFSSDFQHKLKKACCKTGL
Rat-CoV HNSLKEGVQLEQVMDTFVGCARRKC-AID-SDVETRSITKSVMSAVNAGVDFTDESCNNLVPTYVKSDT--IVAADLGVLIQNNAKHVQSNVAKAANVACIWSVDAFNQLSADLQHRLRKACSKTGL
SARS-CoV HSELAKGVALDGVLSTFVSAARQGV--VD-TDVDTKDVIECLKLSHHSDLEVTGDSCNNFMLTYNKVEN--MTPRDLGACIDCNARHINAQVAKSHNVSLIWNVKDYMSLSEQLRKQIRSAAKKNNI
229E ---------------------KS---ALGLS-ISDHEFTSAISNAHRCDVLLSDLSFNNFVSSYAKPEE-KLSAYDLACCMRAGAKVVNANVLTKDQTPIVWHAKDFNSLSAEGRKYIVKTSKAKGL
Bat-CoV-1A ---------------------KQ---ALGFD-VSDEEFVDAVSNAHRFNVLLSDNSFNNLLTSYAKPEE-QLSTHDVATCMRFNAKVVNHNVLIKENVPIVWLARDFQQLSEEGRKYLVKTTKAKGV
BtCoV512 ---------------------RE---SLGLSDVPEEEFSAAVSEAHRYDVLISDVSFNNLIVSYAKPEE-KLAVHDIANCMRVGAKVVNHNVLTKDNVPVVWLAKDFIALSEEARKYIVRTTKTKGI
BtMr-AlphaCoV ---------------------KE---RIGLD-VSDDEFLAAVTNAHRYDVLLSEVSFNNFITSYAKPEE-KLSAFDCAQCVRAGAKIVNHNVIVKEKLSIVWLAKDFQALSAEGRKYIVKTSKVKGL
BtNv-AlphaCoV ---------------------KS---LLGFE-VSDEDFYNAVSDAHRFNILLTDISFNNFITTYAKPEE-KFATHDAAVCMRSGSKIVNHNVLIKENAAVVWYQRDFHALSEDCRKYIVKTSKQKGV
BtRf-AlphaCoV ---------------------KT---ALDIE-ADDEDFVNCVSNAHKFNVLLTDLSFNNFVTSYAKPEE-KLSTHDIAACMRGGSKVVNHNVLVKENVPIVWLAKDFNMLSEEGRKYVVKTAKIKGI
CDPHE15 ---------------------RA---TLGFDDISDEDFTGAVANAHRYGLLLTDISFNNFITSYAKPEE-KMSVHDLAICMRSGARVANHNVLVKENVPVVWSAEAFNALSEESRKYVVKTSKVKGL
FCoV ---------------------YK---LCNLD-VTFSTFEMAINNAHRFGILITDRSFNNFWPSKIKPGSSGVSAMDIGKCMTFDAKIVNAKVLTQRGKSVVWLSQDFSTLSSTAQKVLVKTFVEEGV
Ferret-CoV ---------------------YE---ACKTD-VPYNVFEDVVNNAHKYDVLMTDISYNNIWLTYAKPEE-NLSTFDIANCIKAGAKVVSHNVLIKENVPIVWYAKDFISLSNEARQLLIKTSKAKGV
HKU10 ---------------------KT---ALDID-ADDEEFVNGVSNAHRYNTLLSDLSYNNFVTSYAKPEE-KLSTHDLAACMRGGAKVVNHNILVKENVPIVWYAKDFNMLSEEGRRYVVKTAKLKGV
HKU2 ---------------------KE---LLELD-VSDDDFIKVVSDAHRFDVLITDTSFNNFCTSYAKPAE-KLSSFDLAHCMRSGAKVVNHNVLIKEKMPIVWNCVDFAKLSVDARKYIVKTAKVKGV
HKU8 ---------------------KQ---ALGFE-VSDDEFLNGVSDAHRYNVLLSDTSCNNLVTSYAKPEE-KLSVHDLGTCMRFGAKVVNHNVLIKENVPVVWLASEFHLLSEEGRKYIVKTTKAKGV
LuchengRn-CoV ---------------------KS---VAGIE-CRDSTFETGVKLAHKFDILLCNDSANNFVTTYAKAGT-RIGTADLAVFNRESVREVSHAVLARNKTTIVWSVNVFSKLSGDTQDYIVRTTKAKGL
Mink-CoV ---------------------YE---ACKTD-VPYHTFEDVVVHAHKYDVLMTDLSFNNFWLTYAKPED-NLTSFDIANCIRANAKAVNHSVLSKENVPIVWSATDFVKLSGEAKHLLIKTAKAKGV
NL63 ---------------------KA---TLGLT-VSDDDFVSAVANAHRYDVLLSDLSFNNFFISYAKPED-KLSVYDIACCMRAGSKVVNHNVLIKESIPIVWGVKDFNTLSQEGKKYLVKTTKAKGL
NL63-related ---------------------KT---ALGLT-VSDDDFVSAVANAHRYDILLSDLSFNNFFTSYAKPEE-KLSAYDVACCMRAGSKVVNHNVLTKESTPVVWSAKDFNTLSQEGKKYLVKTTKAKGL
Porcine-EDV ---------------------KS---TLGFDDVPLDTFNAAVAEAHRYDVLLTDMSFNNFTTSYAKPEE-KFPVHDIATCMRVGAKIVNHNVLVKDSIPVVWLVRDFIALSEETRKYIIRTTKVKGI
TGE ---------------------YR---ACNLN-VSFSTFEMAVNNAHRFGILITDRSFNNFWPSKVKPGSSGVSAMDIGKCMTSDAKIVNAKVLTQRGKSVVWLSQDFAALSSTAQKVLVKTFVEEGV
Beta-CoV cons
324944743734*944*74779216-179-8**98339647854743256768347 7*87867*6*498--866299*579654744995557953776 89*3431953884477 87+93893*87+
Alpha-CoV cons
---------------------63---37365-72334*238932**9757*9773*6**864877*745-47664*7+286625693894 49+5764469**2523*23**367746999*5537*9
Combined cons
---------------------63---073-5-744347336942744256468 53+7*+765875*445--765299+379543842983457432446 89*2434923++455783943853247 9
Alignment qual

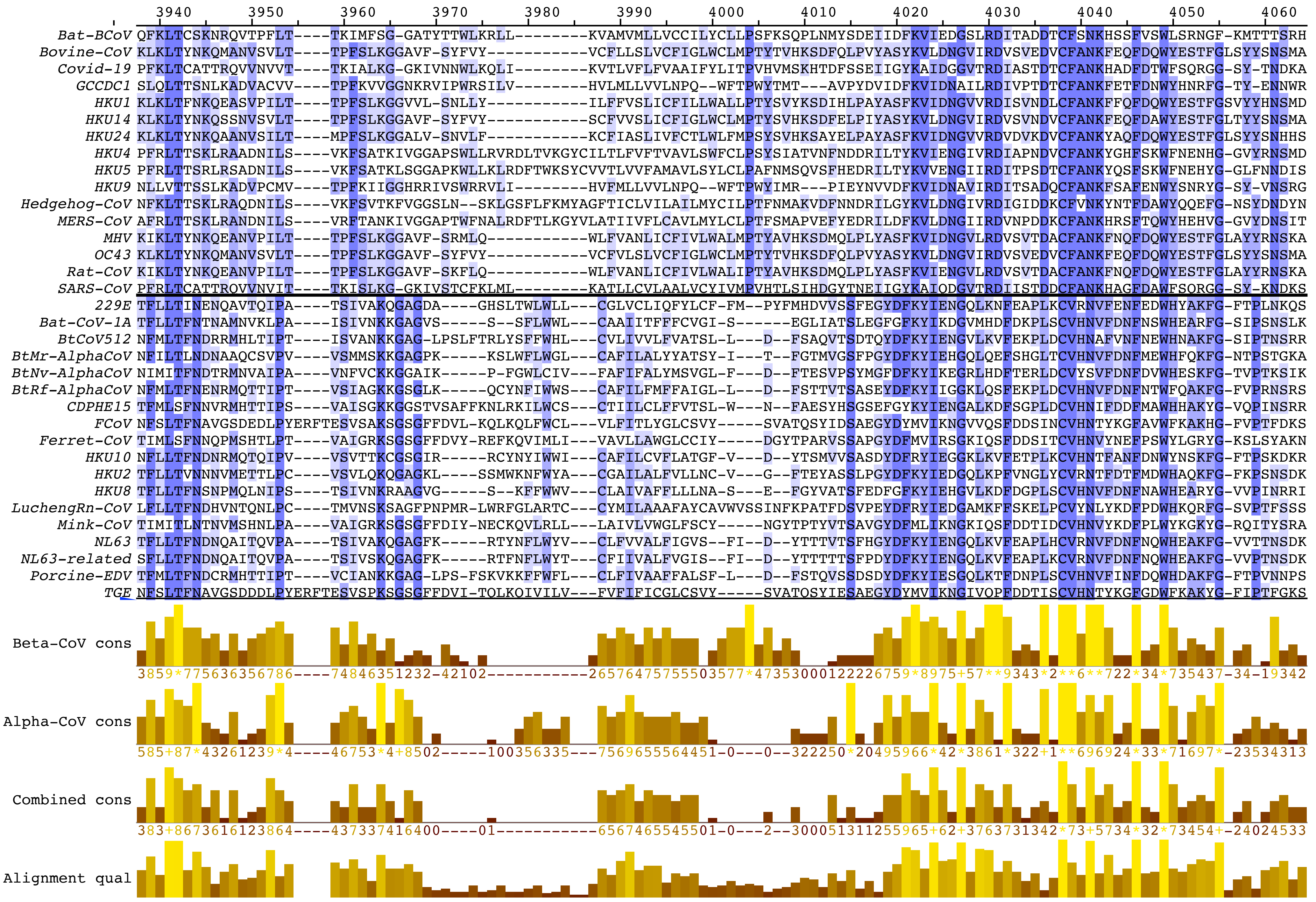

3940 3950 3960 3970 3980 3990 4000 4010 4020 4030 4040 4050 4060
Bat-BCoV QFKLTCSKNRQVTPFLT----TKIMFSG-GATYTTWLKRLL--------KVAMVMLLVCCILYCLLPSFKSQPLNMYSDEIIDFKVIEDGSLRDITADDTCFSNKHSSFVSWLSRNGF-KMTTTSRH
Bovine-CoV KLKLTYNKQMANVSVLT----TPFSLKGGAVF-SYFVY-----------VCFLLSLVCFIGLWCLMPTYTVHKSDFQLPVYASYKVLDNGVIRDVSVEDVCFANKFEQFDQWYESTFGLSYYSNSMA
Covid-19 PFKLTCATTRQVVNVVT----TKIALKG-GKIVNNWLKQLI--------KVTLVFLFVAAIFYLITPVHVMSKHTDFSSEIIGYKAIDGGVTRDIASTDTCFANKHADFDTWFSQRGG-SY-TNDKA
GCCDC1 SLQLTTSNLKADVACVV----TPFKVVGGNKRVIPWRSILV--------HVLMLLVVLNPQ--WFTPWYTMT---AVPYDVIDFKVIDNAILRDIVPTDTCFANKFETFDNWYHNRFG-TY-ENNWR
HKU1 KLKLTFNKQEASVPILT----TPFSLKGGVVL-SNLLY-----------ILFFVSLICFILLWALLPTYSVYKSDIHLPAYASFKVIDNGVVRDISVNDLCFANKFFQFDQWYESTFGSVYYHNSMD
HKU14 KLKLTYNKQSSNVSVLT----TPFSLKGGAVF-SYFVY-----------SCFVVSLICFIGLWCLMPTYSVHKSDFELPIYASYKVLDNGVIRDVSVNDVCFANKFEQFDAWYESTFGLTYYSNSMA
HKU24 KLKLTYNKQAANVSILT----MPFSLKGGALV-SNVLF-----------KCFIASLIVFCTLWLFMPSYSVHKSAYELPAYASFKVIDNGVVRDVDVKDVCFANKYAQFDQWYESTFGLSYYSNHHS
HKU4 PFRLTTSKLRAADNILS----VKFSATKIVGGAPSWLLRVRDLTVKGYCILTLFVFTVAVLSWFCLPSYSIATVNFNDDRILTYKVIENGIVRDIAPNDVCFANKYGHFSKWFNENHG-GVYRNSMD
HKU5 PFRLTTSRLRSADNILS----VKFSATKLSGGAPKWLLKLRDFTWKSYCVVTLVVFAMAVLSYLCLPAFNMSQVSFHEDRILTYKVVENGIIRDITPSDTCFANKYQSFSKWFNEHYG-GLFNNDIS
HKU9 NLLVTTSSLKADVPCMV----TPFKIIGGHRRIVSWRRVLI--------HVFMLLVVLNPQ--WFTPWYIMR---PIEYNVVDFKVIDNAVIRDITSADQCFANKFSAFENWYSNRYG-SY-VNSRG
Hedgehog-CoV NFKLTTSKLRAQDNILS----VKFSVTKFVGGSLN--SKLGSFLFKMYAGFTICLVILAILMYCILPTFNMAKVDFNNDRILGYKVLDNGIVRDIGIDDKCFVNKYNTFDAWYQQEFG-NSYDNDYN
MERS-CoV AFRLTTSKLRANDNILS----VRFTANKIVGGAPTWFNALRDFTLKGYVLATIIVFLCAVLMYLCLPTFSMAPVEFYEDRILDFKVLDNGIIRDVNPDDKCFANKHRSFTQWYHEHVG-GVYDNSIT
MHV KIKLTYNKQEANVPILT----TPFSLKGGAVF-SRMLQ-----------WLFVANLICFIVLWALMPTYAVHKSDMQLPLYASFKVIDNGVLRDVSVTDACFANKFNQFDQWYESTFGLAYYRNSKA
OC43 KLKLTYNKQMANVSVLT----TPFSLKGGAVF-SYFVY-----------VCFVLSLVCFIGLWCLMPTYTVHKSDFQLPVYASYKVLDNGVIRDVSVEDVCFANKFEQFDQWYESTFGLSYYSNSMA
Rat-CoV KIKLTYNKQEANVPILT----TPFSLKGGAVF-SKFLQ-----------WLFVANLICFIVLWALIPTYAVHKSDMQLPLYASFKVIENGVLRDVSVTDACFANKFNQFDQWYESTFGLAYYRNSKA
SARS-CoV PFRLTCATTRQVVNVIT----TKISLKG-GKIVSTCFKLML--------KATLLCVLAALVCYIVMPVHTLSIHDGYTNEIIGYKAIQDGVTRDIISTDDCFANKHAGFDAWFSQRGG-SY-KNDKS
229E TFLLTINENQAVTQIPA----TSIVAKQGAGDA----GHSLTWLWLL---CGLVCLIQFYLCF-FM--PYFMHDVVSSFEGYDFKYIENGQLKNFEAPLKCVRNVFENFEDWHYAKFG-FTPLNKQS
Bat-CoV-1A TFLLTFNTNAMNVKLPA----ISIVNKKGAGVS-----S--SFLWWL---CAAIITFFFCVGI-S------EGLIATSLEGFGFKYIKDGVMHDFDKPLSCVHNVFDNFNSWHEARFG-SIPSNSLK
BtCoV512 NFMLTFNDRRMHLTIPT----ISVANKKGAG-LPSLFTRLYSFFWHL---CVLIVVLFVATSL-L---D--FSAQVTSDTQYDFKYIENGVLKVFEKPLDCVHNAFVNFNEWHNAKFG-SIPTNSRR
BtMr-AlphaCoV NFILTLNDNAAQCSVPV----VSMMSKKGAGPK-----KSLWFLWGL---CAFILALYYATSY-I---T--FGTMVGSFPGYDFKYIEHGQLQEFSHGLTCVHNVFDNFMEWHFQKFG-NTPSTGKA
BtNv-AlphaCoV NIMITFNDTRMNVAIPA----VNFVCKKGGAIK-----P-FGWLCIV---FAFIFALYMSVGL-F---D--FTESVPSYMGFDFKYIKEGRLHDFTERLDCVYSVFDNFDVWHESKFG-TVPTKSIK
BtRf-AlphaCoV NFMLTFNENRMQTTIPT----VSIAGKKGSGLK-----QCYNFIWWS---CAFILFMFFAIGL-L---D--FSTTVTSASEYDFKYIIGGKLQSFEKPLDCVHNVFDNFNTWFQAKFG-FVPRNSRS
CDPHE15 TFMLSFNNVRMHTTIPS----VAISGKKGGSTVSAFFKNLRKILWCS---CTIILCLFFVTSL-W---N--FAESYHSGSEFGYKYIENGALKDFSGPLDCVHNIFDDFMAWHHAKYG-VQPINSRR
FCoV NFSLTFNAVGSDEDLPYERFTESVSAKSGSGFFDVL-KQLKQLFWCL---VLFITLYGLCSVY-----SVATQSYIDSAEGYDYMVIKNGVVQSFDDSINCVHNTYKGFAVWFKAKHG-FVPTFDKS
Ferret-CoV TIMLSFNNQPMSHTLPT----VAIGRKSGSGFFDVY-REFKQVIMLI---VAVLLAWGLCCIY-----DGYTPARVSSAPGYDFMVIRSGKIQSFDDSITCVHNVYNEFPSWYLGRYG-KSLSYAKN
HKU10 NFLLTFNDNRMQTQIPV----VSVTTKCGSGIR-----RCYNYIWWI---CAFILCVFLATGF-V---D--YTSMVVSASDYDFRYIEGGKLKVFETPLKCVHNTFANFDNWYNSKFG-FTPSKDKR
HKU2 TFLLTVNNNVMETTLPC----VSVLQKQGAGKL----SSMWKNFWYA---CGAILALFVLLNC-V---G--FTEYASSLPGYDFKYIEDGQLKPFVNGLYCVRNTFDTFMDWHAQKFG-FKPSNSDK
HKU8 TFLLTFNSNPMQLNIPS----TSIVNKRAAGVG-----S--KFFWWV---CLAIVAFFLLLNA-S---E--FGYVATSFEDFGFKYIEHGVLKDFDGPLSCVHNVFDNFNAWHEARYG-VVPINRRI
LuchengRn-CoV LFLLTFNDHVNTQNLPC----TMVNSKSAGFPNPMR-LWRFGLARTC---CYMILAAAFAYCAVWVSSINFKPATFDSVPEYDFRYIEDGAMKFFSKELPCVYNLYKDFPDWHKQRFG-SVPTFSSS
Mink-CoV TIMITLNTNVMSHNLPA----VAIGRKSGSGFFDIY-NECKQVLRLL---LAIVLVWGLFSCY-----NGYTPTYVTSAVGYDFMLIKNGKIQSFDDTIDCVHNVYKDFPLWYKGKYG-RQITYSRA
NL63 TFLLTFNDNQAITQVPA----TSIVAKQGAGFK-----RTYNFLWYV---CLFVVALFIGVS--FI--D--YTTTVTSFHGYDFKYIENGQLKVFEAPLHCVRNVFDNFNQWHEAKFG-VVTTNSDK
NL63-related SFLLTFNDNQAITQVPA----TSIVSKQGAGFK-----RTFNFLWYT---CFIIVALFVGIS--FI--D--YTTTTTSFPDYDFKYIENGQLKVFEAPLKCVRNVFDNFNQWHEAKFG-VVPTNSDK
Porcine-EDV TFMLTFNDCRMHTTIPT----VCIANKKGAG-LPS-FSKVKKFFWFL---CLFIVAAFFALSF-L---D--FSTQVSSDSDYDFKYIESGQLKTFDNPLSCVHNVFINFDQWHDAKFG-FTPVNNPS
TGE NFSLTFNAVGSDDDLPYERFTESVSPKSGSGFFDVI-TQLKQIVILV---FVFIFICGLCSVY-----SVATQSYIESAEGYDYMVIKNGIVQPFDDTISCVHNTYKGFGDWFKAKYG-FIPTFGKS
Beta-CoV cons
3859*77563635678б----7484635123 2-42102-----------265764757555035 77*47353000122226759*8975+57**9343*2**6**722*34*735437-34-19342
Alpha-CoV cons
585+87*43261239*4----46753*4+8502-----100356335---7569655564451-0---0--322250*20495966*42*3861*322+1**696924*33*71697*-23534313
Combined cons
383+8673616123864----437337416400----01-----------6567465545501-0---2--3000513112559б5+62+3763731342*73+5734*32*73454+-24024533
Alignment qual

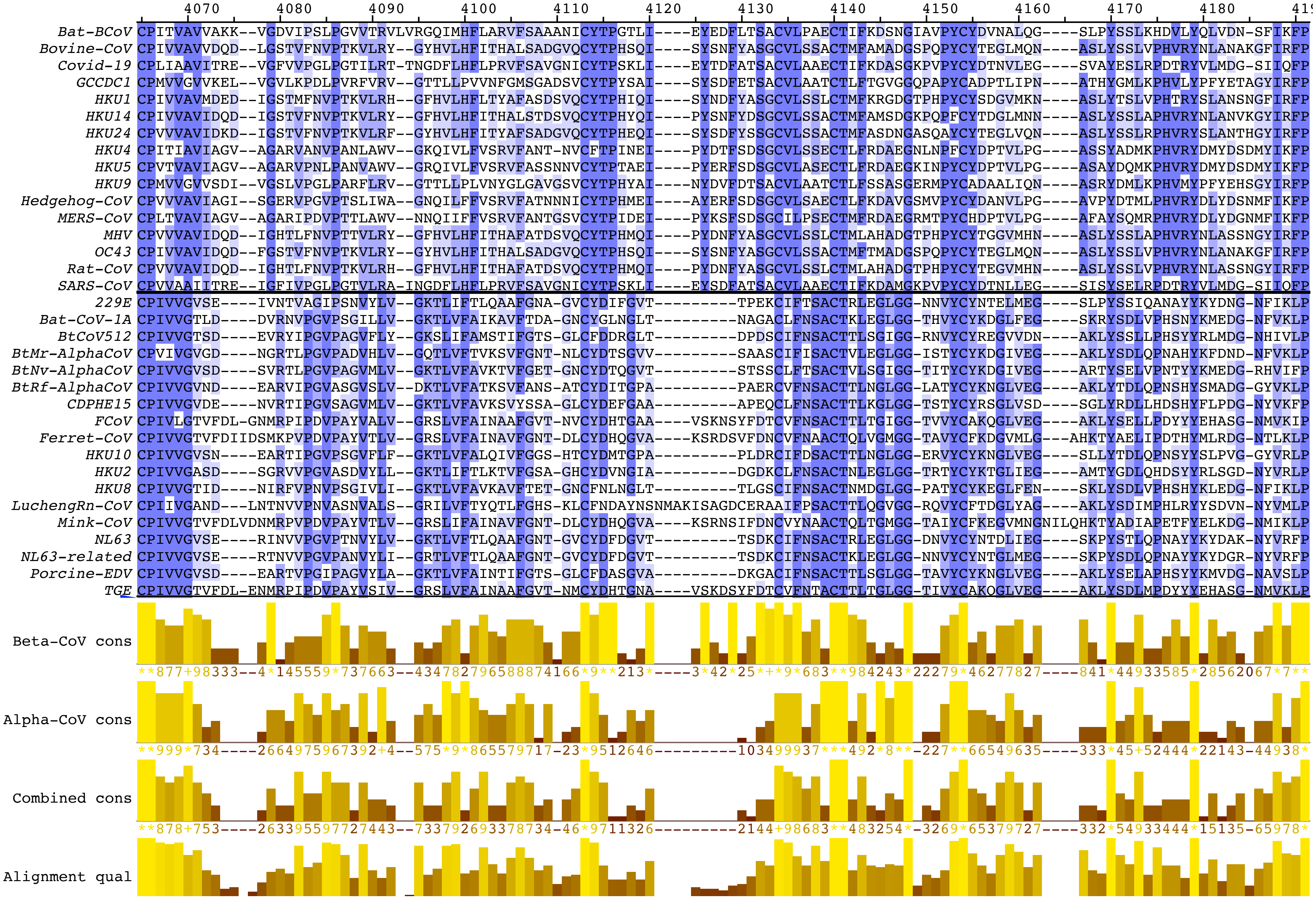
4070 4080 4090 4100 4110 4120 4130 4140 4150 4160 4170 4180
Bat-BCoV CPITVAVVAKK--VGDVIPSLPGVVTRVLVRGQIMHFLARVFSAAANICYTPGTLI----EYEDFLTSACVLPAECTIFKDSNGIAVPYCYDVNALQG----SLPYSSLKHDVLYQLVDN-SFIKFP
Bovine-CoV CPIVVAVVDQD--LGSTVFNVPTKVLRY--GYHVLHFITHALSADGVQCYTPHSQI----SYSNFYASGCVLSSACTMFAMADGSPQPYCYTEGLMQN----ASLYSSLVPHVRYNLANAKGFIRFP
Covid-19 CPLIAAVITRE--VGFVVPGLPGTILRT-TNGDFLHFLPRVFSAVGNICYTPSKLI----EYTDFATSACVLAAECTIFKDASGKPVPYCYDTNVLEG----SVAYESLRPDTRYVLMDG-SIIQFP
GCCDC1 CPMVVGVVKEL--VGVLKPDLPVRFVRV--GTTLLPVVNFGMSGADSVCYTPYSAI----SYSDFETSACVLAATCTLFTGVGGQPAPYCADPTLIPN----ATHYGMLKPHVLYPFYETAGYIRFP
HKU1 CPIVVAVMDED--IGSTMFNVPTKVLRH--GFHVLHFLTYAFASDSVQCYTPHIQI----SYNDFYASGCVLSSLCTMFKRGDGTPHPYCYSDGVMKN----ASLYTSLVPHTRYSLANSNGFIRFP
HKU14 CPIVVAVIDQD--IGSTVFNVPTKVLRY--GFHVLHFITHALSTDSVQCYTPHYQI----PYSNFYDSGCVLSSACTMFAMSDGKPQPFCYTDGLMNN----ASLYSSLAPHVRYNLANVKGYIRFP
HKU24 CPVVVAVIDKD--IGSTVFNVPTKVLRF--GYHVLHFITYAFSADGVQCYTPHEQI----SYSDFYSSGCVLSSACTMFASDNGASQAYCYTEGLVQN----ASLYSSLRPHVRYSLANTHGYIRFP
HKU4 CPITIAVIAGV--AGARVANVPANLAWV--GKQIVLFVSRVFANT-NVCFTPINEI----PYDTFSDSGCVLSSECTLFRDAEGNLNPFCYDPTVLPG----ASSYADMKPHVRYDMYDSDMYIKFP
HKU5 CPVTVAVIAGV--AGARVPNLPANVAWV--GRQIVLFVSRVFASSNNVCYTPTAEI----PYERFSDSGCVLASECTLFRDAEGKINPYCYDPTVLPG----ASAYDQMKPHVRYDMYDSDMYIKFP
HKU9 CPMVVGVVSDI--VGSLVPGLPARFLRV--GTTLLPLVNYGLGAVGSVCYTPHYAI----NYDVFDTSACVLAATCTLFSSASGERMPYCADAALIQN----ASRYDMLKPHVMYPFYEHSGYIRFP
Hedgehog-CoV CPVVVAVIAGI--SGERVPGVPTSLIWA--GNQILFFVSRVFATNNNICYTPHMEI----AYERFSDSGCVLSAECTLFKDAVGSMVPYCYDANVLPG----AVPYDTMLPHVRYDLYDSNMFIKFP
MERS-CoV CPLTVAVIAGV--AGARIPDVPTTLAWV--NNQIIFFVSRVFANTGSVCYTPIDEI----PYKSFSDSGCILPSECTMFRDAEGRMTPYCHDPTVLPG----AFAYSQMRPHVRYDLYDGNMFIKFP
MHV CPVVVAVIDQD--IGHTLFNVPTTVLRY--GFHVLHFITHAFATDSVQCYTPHMQI----PYDNFYASGCVLSSLCTMLAHADGTPHPYCYTGGVMHN----ASLYSSLAPHVRYNLASSNGYIRFP
OC43 CPIVVAVIDQD--FGSTVFNVPTKVLRY--GYHVLHFITHALSADGVQCYTPHSQI----SYSNFYASGCVLSSACTMFTMADGSPQPYCYTEGLMQN----ASLYSSLVPHVRYNLANAKGFIRFP
Rat-CoV CPVVVAVIDQD--IGHTLFNVPTKVLRH--GFHVLHFITHAFATDSVQCYTPHMQI----PYDNFYASGCVLSSLCTMLAHADGTPHPYCYTEGVMHN----ASLYSSLVPHVRYNLASSNGYIRFP
SARS-CoV CPVVAAIITRE--IGFIVPGLPGTVLRA-INGDFLHFLPRVFSAVGNICYTPSKLI----EYSDFATSACVLAAECTIFKDAMGKPVPYCYDTNLLEG----SISYSELRPDTRYVLMDG-SIIQFP
229E CPIVVGVSE----IVNTVAGIPSNVYLV--GKTLIFTLQAAFGNA-GVCYDIFGVT---------TPEKCIFTSACTRLEGLGG-NNVYCYNTELMEG----SLPYSSIQANAYYKYDNG-NFIKLP
Bat-CoV-1A CPIVVGTLD----DVRNVPGVPSGILLV--GKTLVFAIKAVFTDA-GNCYGLNGLT---------NAGACLFNSACTKLEGLGG-THVYCYKDGLFEG----SKRYSDLVPHSNYKMEDG-NFVKLP
BtCoV512 CPIVVGTSD----EVRYIPGVPAGVFLY--GKSLIFAMSTIFGTS-GLCFDDRGLT---------DPDSCIFNSACTTLSGIGG-RNVYCYREGVVDN----AKLYSSLLPHSYYRLMDG-NHIVLP
BtMr-AlphaCoV CPVIVGVGD----NGRTLPGVPADVHLV--GQTLVFTVKSVFGNT-NLCYDTSGVV---------SAASCIFISACTVLEGLGG-ISTYCYKDGIVEG----AKLYSDLQPNAHYKFDND-NFVKLP
BtNv-AlphaCoV CPIVVGVSD----SVRTLPGVPAGVMLV--GKTLVFAVKTVFGET-GNCYDTQGVT---------STSSCLFTSACTVLSGIGG-TITYCYKDGIVEG----ARTYSELVPNTYYKMEDG-RHVIFP
BtRf-AlphaCoV CPIVVGVND----EARVIPGVASGVSLV--DKTLVFATKSVFANS-ATCYDITGPA---------PAERCVFNSACTTLNGLGG-LATYCYKNGLVEG----AKLYTDLQPNSHYSMADG-GYVKLP
CDPHE15 CPIVVGVDE----NVRTIPGVSAGVMLV--GKTLVFAVKSVYSSA-GLCYDEFGAA---------APEQCLFNSACTTLKGLGG-TSTYCYRSGLVSD----SGLYRDLLHDSHYFLPDG-NYVKFP
FCoV CPIVLGTVFDL-GNMRPIPDVPAYVALV--GRSLVFAINAAFGVT-NVCYDHTGAA----VSKNSYFDTCVFNSACTTLTGIGG-TVVYCAKQGLVEG----AKLYSELLPDYYYEHASG-NMVKIP
Ferret-CoV CPIVVGTVFDIIDSMKPVPDVPAYVTLV--GRSLVFAINAVFGNT-DLCYDHQGVA----KSRDSVFDNCVFNAACTQLVGMGG-TAVYCFKDGVMLG---AHKTYAELIPDTHYMLRDG-NTLKLP
HKU10 CPIVVGVSN----EARTIPGVPSGVFLF--GKTLVFALQIVFGGS-HTCYDMTGPA---------PLDRCIFDSACTTLNGLGG-ERVYCYKNGLVEG----SLLYTDLQPNSYYSLPVG-GYVRLP
HKU2 CPIVVGASD----SGRVVPGVASDVYLL--GKTLIFTLKTVFGSA-GHCYDVNGIA---------DGDKCLFNSACTNLEGLGG-TRTYCYKTGLIEG----AMTYGDLQHDSYYRLSGD-NYVRLP
HKU8 CPIVVGTID----NIRFVPNVPSGIVLI--GKTLVFAVKAVFTET-GNCFNLNGLT---------TLGSCIFNSACTNMDGLGG-PATYCYKEGLFEN----SKLYSDLVPHSHYKLEDG-NFIKLP
LuchengRn-CoV CPIIVGAND---LNTNVVPNVASNVALS--GRILVFTYQTLFGHS-KLCFNDAYNSNMAKISAGDCERAAIFPSACTTLQGVGG-RQVYCFTDGLYAG----AKLYSDIMPHLRYYSDVN-NYVMLP
Mink-CoV CPIVVGTVFDLVDNMRPVPDVPAYVTLV--GRSLIFAINAVFGNT-DLCYDHQGVA----KSRNSIFDNCVYNAACTQLTGMGG-TAIYCFKEGVMNGNILQHKTYADIAPETFYELKDG-NMIKLP
NL63 CPIVVGVSE----RINVVPGVPTNVYLV--GKTLVFTLQAAFGNT-GVCYDFDGVT---------TSDKCIFNSACTRLEGLGG-DNVYCYNTDLIEG----SKPYSTLQPNAYYKYDAK-NYVRFP
NL63-related CPIVVGVSE----RTNVVPGVPANVYLI--GRTLVFTLQAAFGNT-GVCYDFDGVT---------TSDKCIFNSACTKLEGLGG-NNVYCYNTGLMEG----SKPYSDLQPNAYYKYDGR-NYVRFP
Porcine-EDV CPIVVGVSD----EARTVPGIPAGVYLA--GKTLVFAINTIFGTS-GLCFDASGVA---------DKGACIFNSACTTLSGLGG-TAVYCYKNGLVEG----AKLYSELAPHSYYKMVDG-NAVSLP
TGE CPIVVGTVFDL-ENMRPIPDVPAYVSIV--GRSLVFAINAAFGVT-NMCYDHTGNA----VSKDSYFDTCVFNTACTTLTGLGG-TIVYCAKQGLVEG----AKLYSDLMPDYYYEHASG-NMVKLP
Beta-CoV cons
877+98333--4*145559*737663--434782796588874166*9213*----3*42*25*+*9*683**984243*22279*46277827----841*44933585*28562067*7**
Alpha-CoV cons
999*734----2664975967392+4--575*9*865579717-23*9512646---------103499937*492*8**-227**66549635----333*45+52444*22143-44938*
Combined cons
878+753----2633955977274443--73379269337873 4-46*9711326---------2144+98683483254*-3269*65379727----332*54933444*15135-65978*
Alignment qual

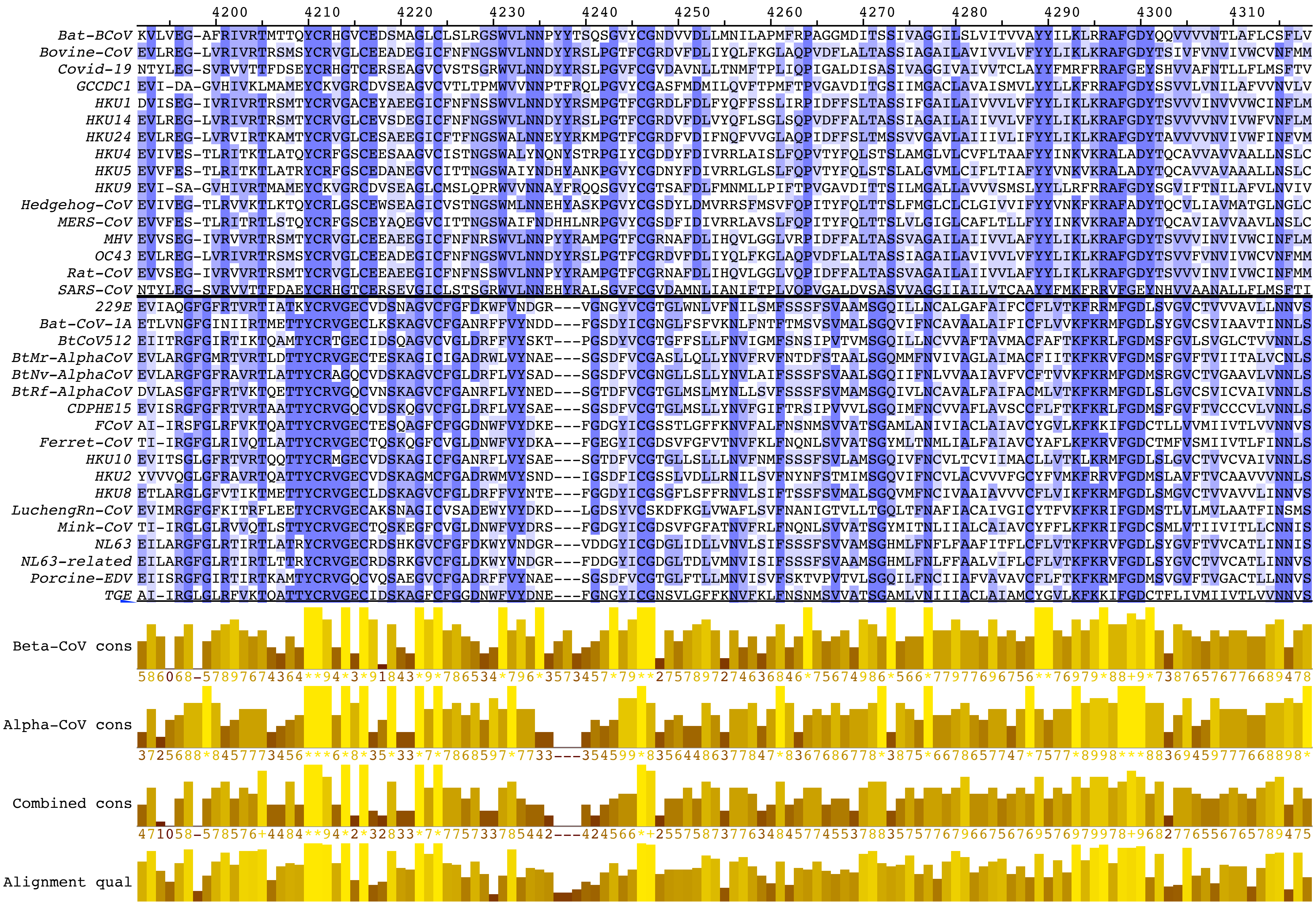
4200 4210 4220 4230 4240 4250 4260 4270 4280 4290 4300 4310
Bat-BCoV
Bovine-CoV
Covid-19
GCCDC1
HKU1
HKU14
HKU24
HKU4
HKU5
HKU9
Hedgehog-CoV
MERS-CoV
MHV
OC43
Rat-CoV
SARS-CoV
229E
Bat-CoV-1A
BtCoV512
BtMr-AlphaCoV
BtNv-AlphaCoV
BtRf-AlphaCoV
CDPHE15
FCoV
Ferret-CoV
HKU10
HKU2
HKU8
LuchengRn-CoV
Mink-CoV
NL63
NL63-related
Porcine-EDV
TGE
Beta-CoV cons
Alpha-CoV cons
Combined cons
Alignment qual

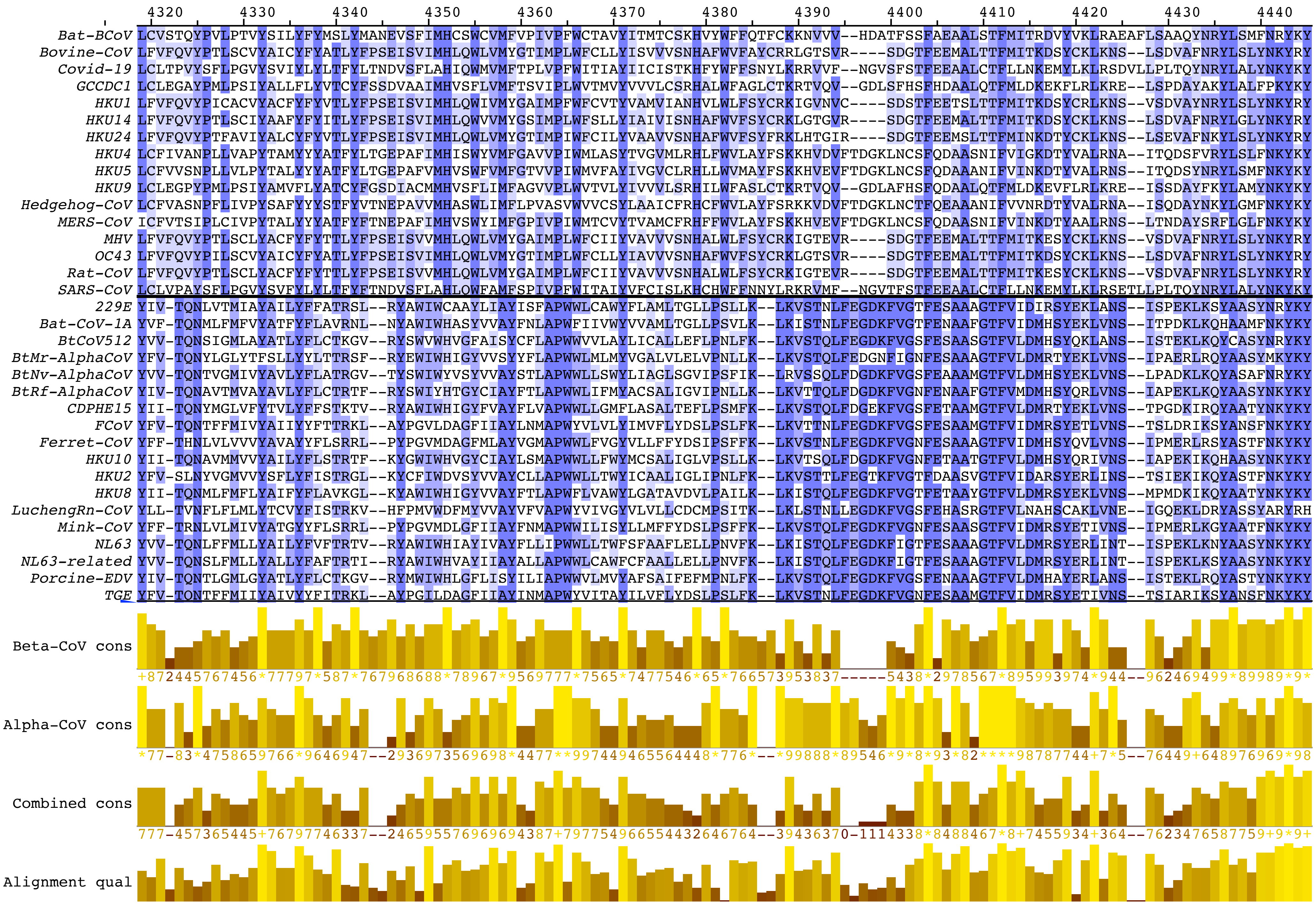

4320
4330
4340
4350
4360
4370
4380
4390
4400
4410
4420
4430
4440
Bat-BCoV
Bovine-CoV
Covid-19
GCCDC1
HKU1
HKU14
HKU24
HKU4
HKU5
HKU9
Hedgehog-CoV
MERS-CoV
MHV
OC43
Rat-CoV
SARS-CoV
229E
Bat-CoV-1A
BtCoV512
BtMr-AlphaCoV
BtNv-AlphaCoV
BtRf-AlphaCoV
CDPHE15
FCoV
Ferret-CoV
HKU10
HKU2
HKU8
LuchengRn-CoV
Mink-CoV
NL63
NL63-related
Porcine-EDV
TGE
Beta-CoV cons
Alpha-CoV cons
Combined cons
Alignment qual

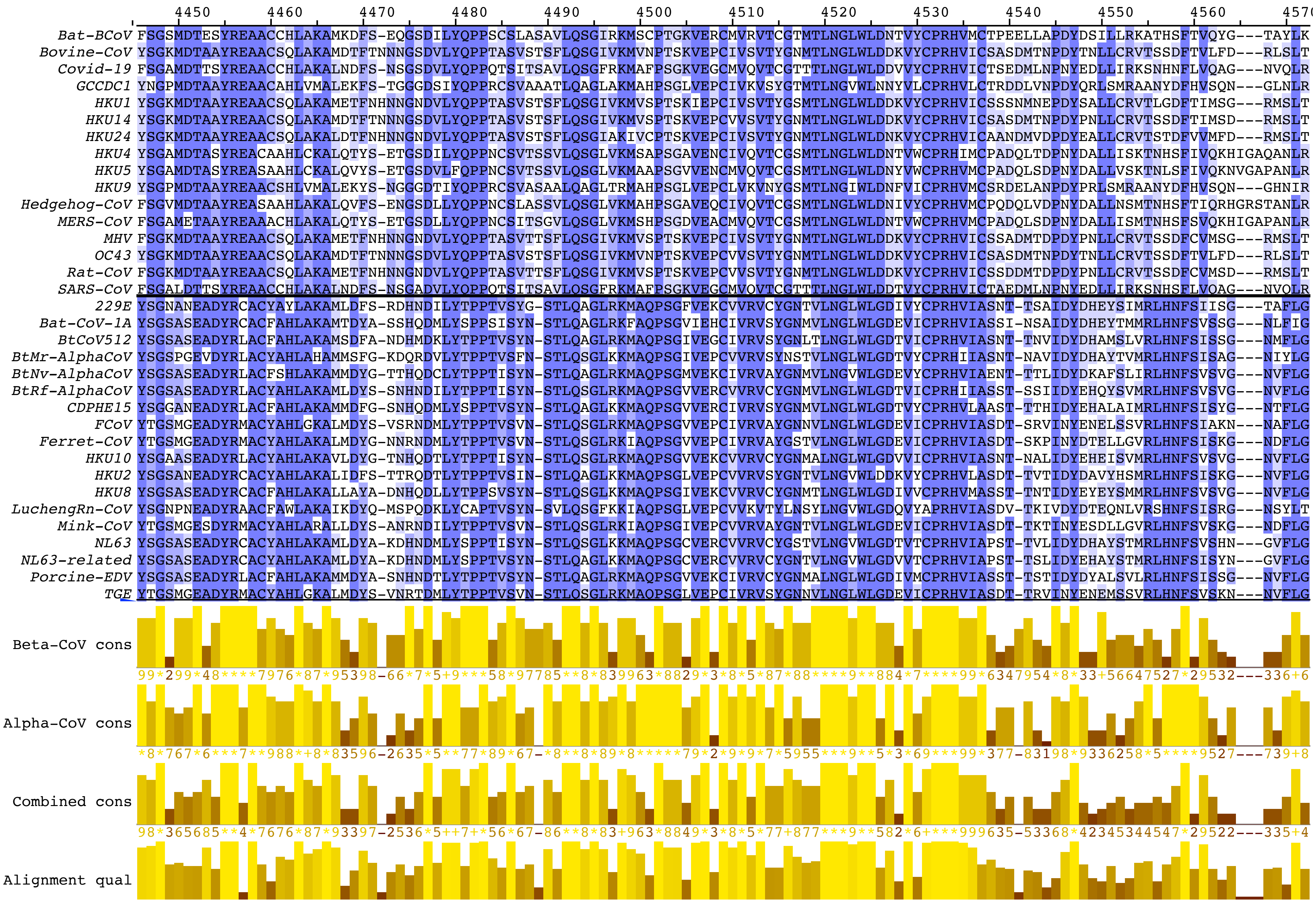

4450
4460
4470
4480
4490
4500
4510
4520
4530
4540
4550
4560
4570
Bat-BCoV
Bovine-CoV
Covid-19
GCCDC1
HKU1
HKU14
HKU24
HKU4
HKU5
HKU9
Hedgehog-CoV
MERS-CoV
MHV
OC43
Rat-CoV
SARS-CoV
229E
Bat-CoV-1A
BtCoV512
BtMr-AlphaCoV
BtNv-AlphaCoV
BtRf-AlphaCoV
CDPHE15
FCoV
Ferret-CoV
HKU10
HKU2
HKU8
LuchengRn-CoV
Mink-CoV
NL63
NL63-related
Porcine-EDV
TGE
Beta-CoV cons
Alpha-CoV cons
Combined cons
Alignment qual

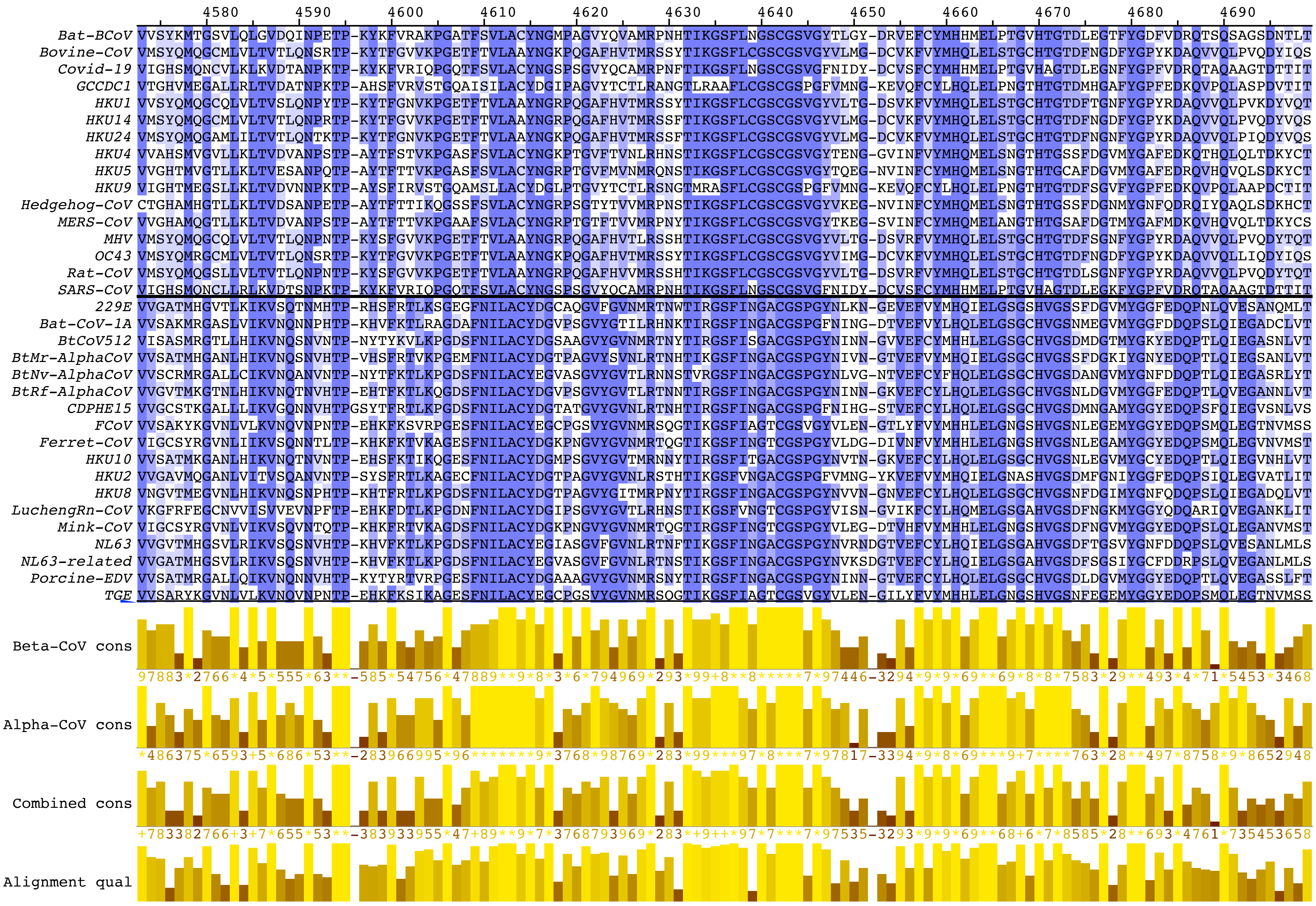
4580
4590
4600
4610
4620
4630
4640
4650
4660
4670
4680
4690
Bat-BCoV
Bovine-CoV
Covid-19
GCCDC1
HKU1
HKU14
HKU24
HKU4
HKU5
HKU9
Hedgehog-CoV
MERS-CoV
MHV
OC43
Rat-CoV
SARS-CoV
229E
Bat-CoV-1A
BtCoV512
BtMr-AlphaCoV
BtNv-AlphaCoV
BtRf-AlphaCoV
CDPHE15
FCoV
Ferret-CoV
HKU10
HKU2
HKU8
LuchengRn-CoV
Mink-CoV
NL63
NL63-related
Porcine-EDV
TGE
Beta-CoV cons
Alpha-CoV cons
Combined cons
Alignment qual

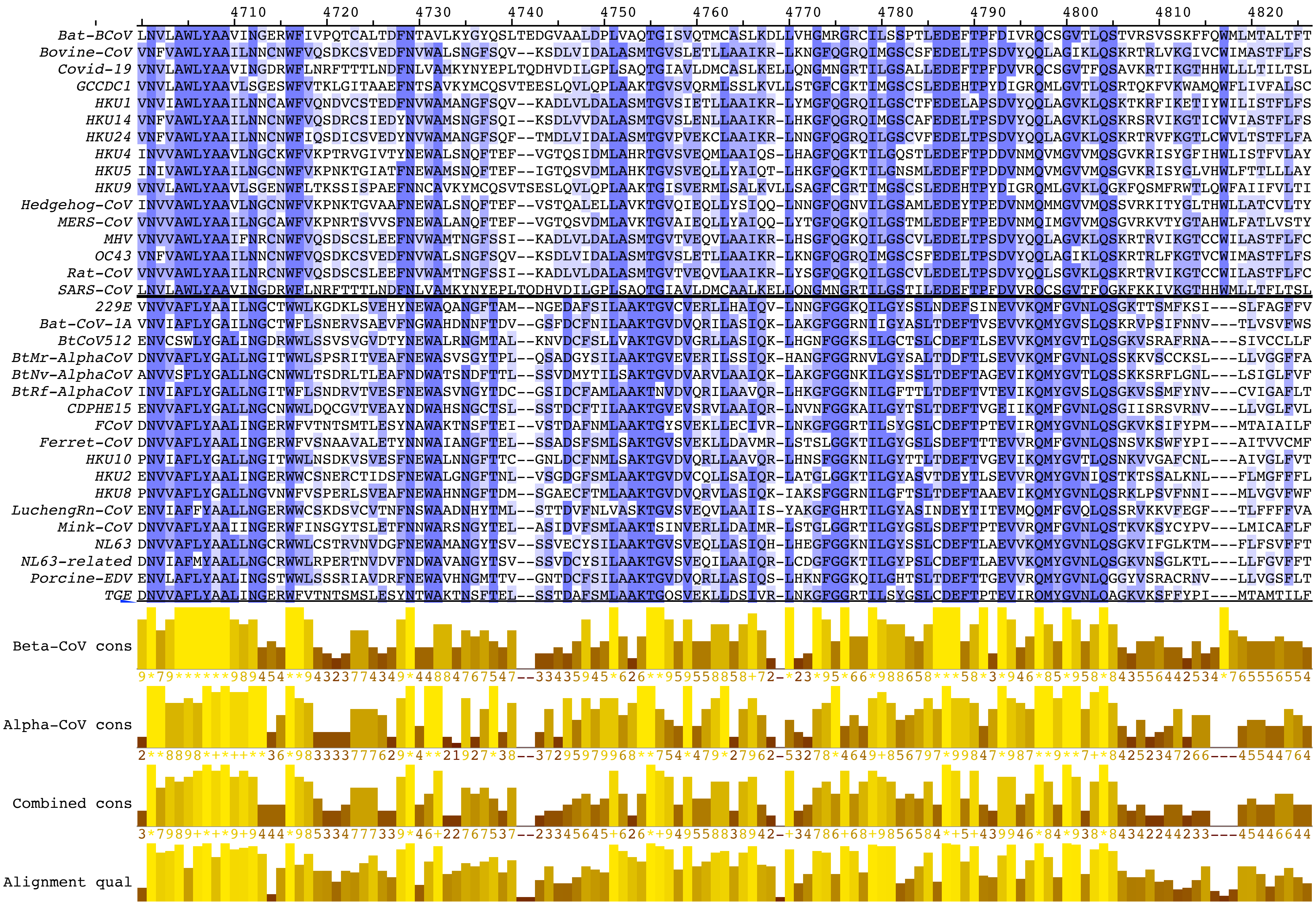

4710
4720
4730
4740
4750
4760
4770
4780
4790
4800
4810
4820
Bat-BCoV
Bovine-CoV
Covid-19
GCCDC1
HKU1
HKU14
HKU24
HKU4
HKU5
HKU9
Hedgehog-CoV
MERS-CoV
MHV
OC43
Rat-CoV
SARS-CoV
229E
Bat-CoV-1A
BtCoV512
BtMr-AlphaCoV
BtNv-AlphaCoV
BtRf-AlphaCoV
CDPHE15
FCoV
Ferret-CoV
HKU10
HKU2
HKU8
LuchengRn-CoV
Mink-CoV
NL63
NL63-related
Porcine-EDV
TGE
Beta-CoV cons
Alpha-CoV cons
Combined cons
Alignment qual

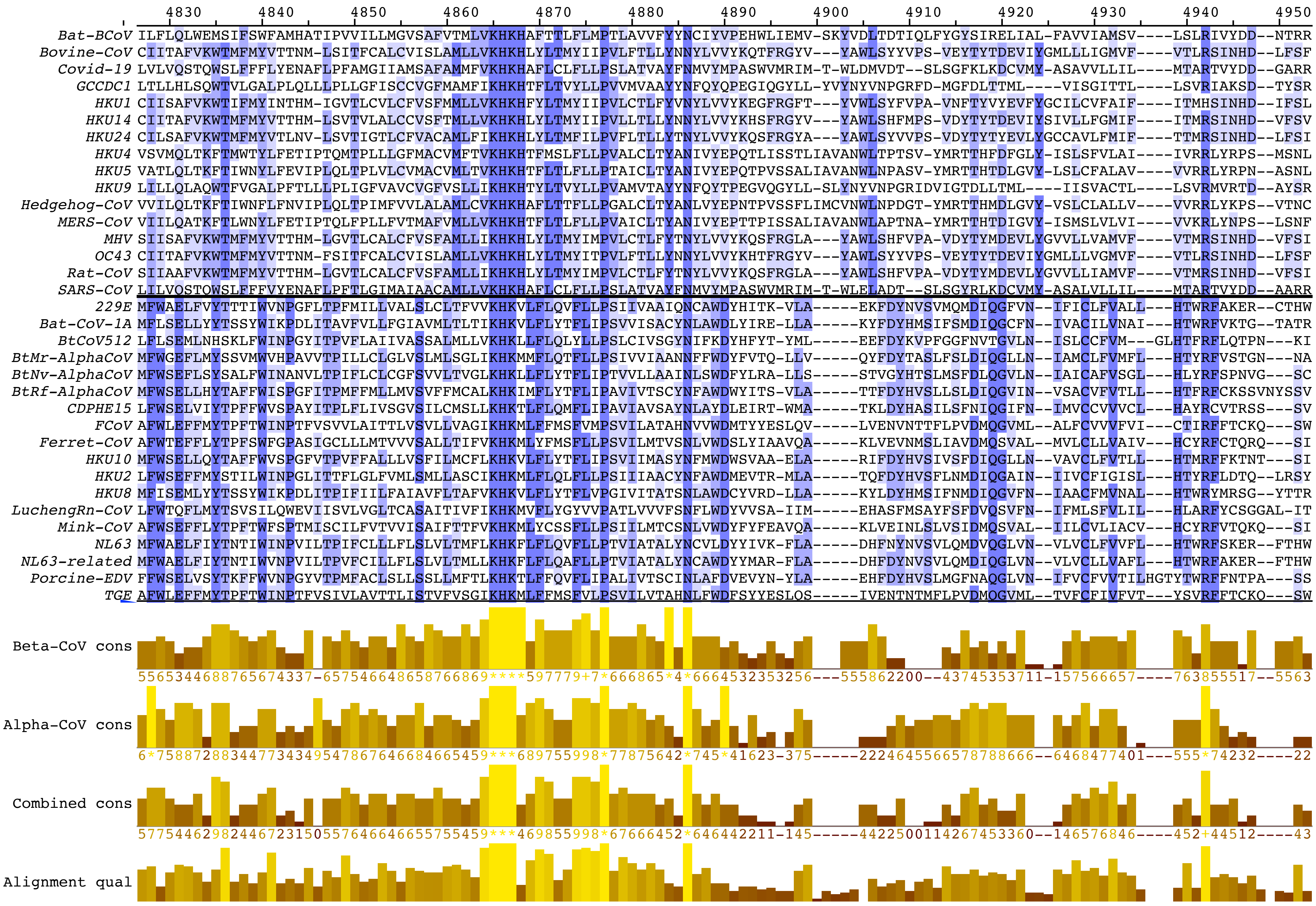
4830 4840 4850 4860 4870 4880 4890 4900 4910 4920 4930 4940 4950
Bat-BCoV
Bovine-CoV
Covid-19
GCCDC1
HKU1
HKU14
HKU24
HKU4
HKU5
HKU9
Hedgehog-CoV
MERS-CoV
MHV
OC43
Rat-CoV
SARS-CoV
229E
Bat-CoV-1A
BtCoV512
BtMr-AlphaCoV
BtNv-AlphaCoV
BtRf-AlphaCoV
CDPHE15
FCoV
Ferret-CoV
HKU10
HKU2
HKU8
LuchengRn-CoV
Mink-CoV
NL63
NL63-related
Porcine-EDV
TGE
Beta-CoV cons
Alpha-CoV cons
Combined cons
Alignment qual

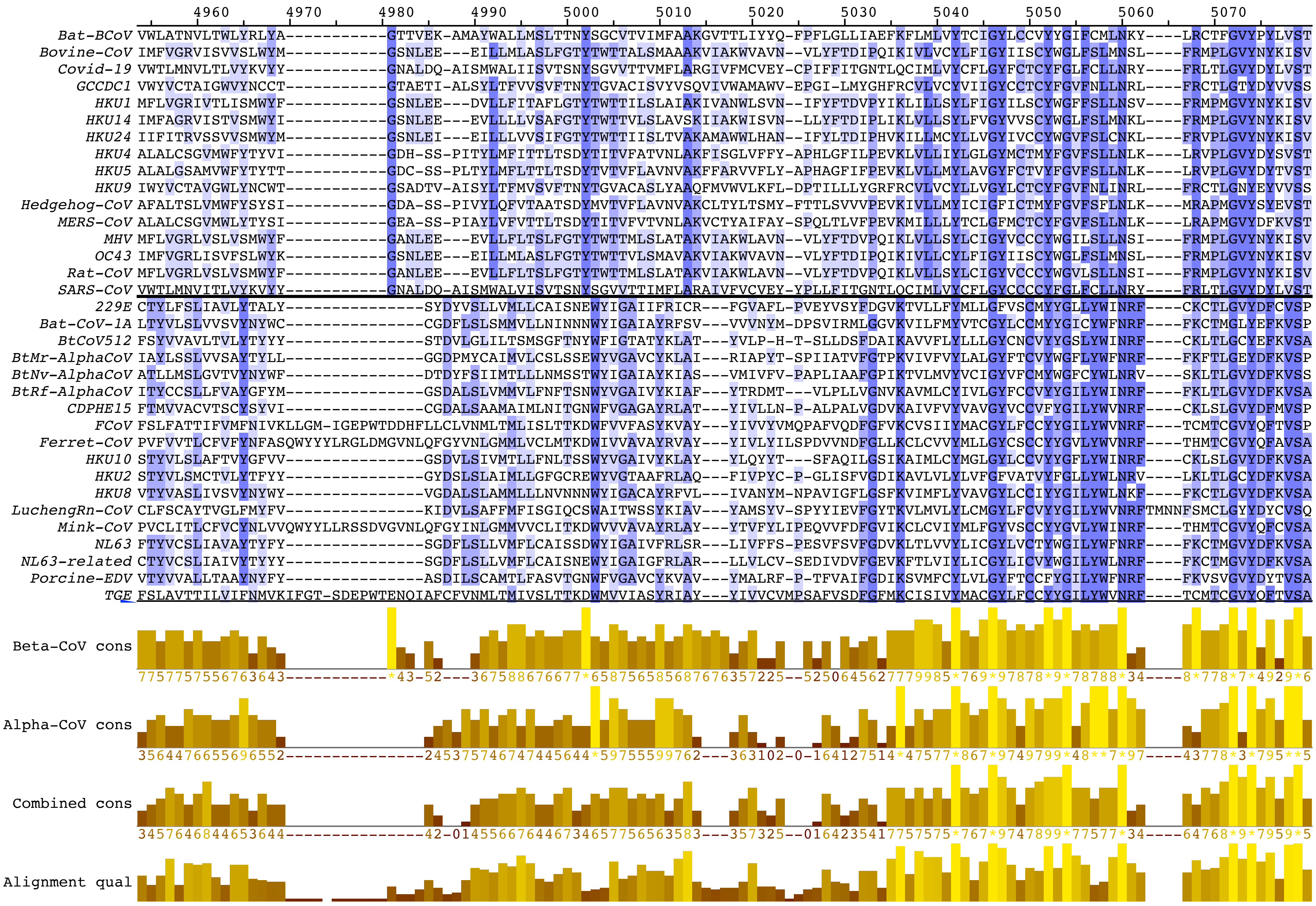
4960
4970
4980
4990
5000
5010
5020
5030
5040
5050
5060
5070
Bat-BCoV
Bovine-CoV
Covid-19
GCCDC1
HKU1
HKU14
HKU24
HKU4
HKU5
HKU9
Hedgehog-CoV
MERS-CoV
MHV
OC43
Rat-CoV
SARS-CoV
229E
Bat-CoV-1A
BtCoV512
BtMr-AlphaCoV
BtNv-AlphaCoV
BtRf-AlphaCoV
CDPHE15
FCoV
Ferret-CoV
HKU10
HKU2
HKU8
LuchengRn-CoV
Mink-CoV
NL63
NL63-related
Porcine-EDV
TGE
Beta-CoV cons
Alpha-CoV cons
Combined cons
Alignment qual

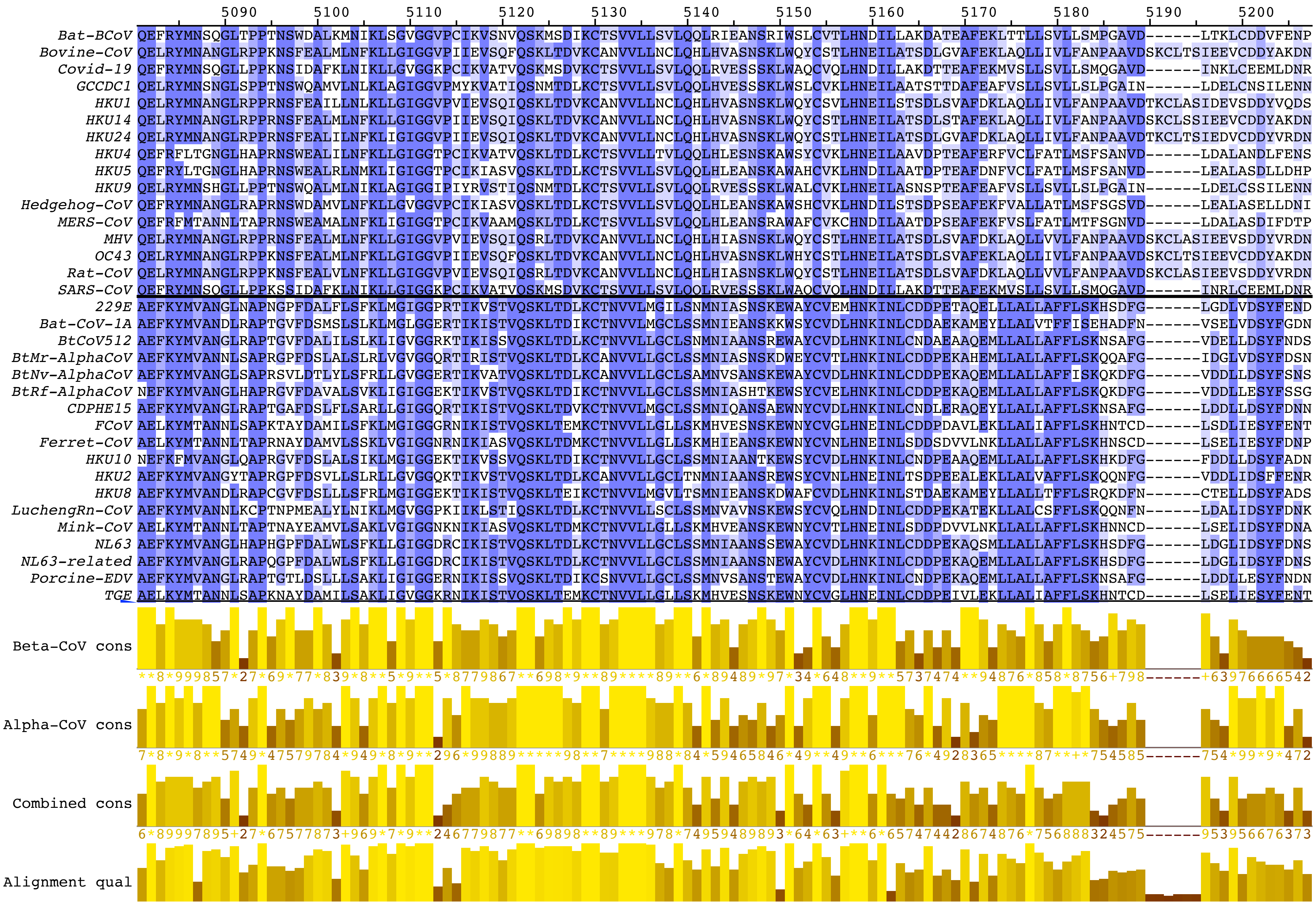

5090
5100
5110
5120
5130
5140
5150
5160
5170
5180
5190
5200
Bat-BCoV
Bovine-CoV
Covid-19
GCCDC1
HKU1
HKU14
HKU24
HKU4
HKU5
HKU9
Hedgehog-CoV
MERS-CoV
MHV
OC43
Rat-CoV
SARS-CoV
229E
Bat-CoV-1A
BtCoV512
BtMr-AlphaCoV
BtNv-AlphaCoV
BtRf-AlphaCoV
CDPHE15
FCoV
Ferret-CoV
HKU10
HKU2
HKU8
LuchengRn-CoV
Mink-CoV
NL63
NL63-related
Porcine-EDV
TGE
Beta-CoV cons
Alpha-CoV cons
Combined cons
Alignment qual

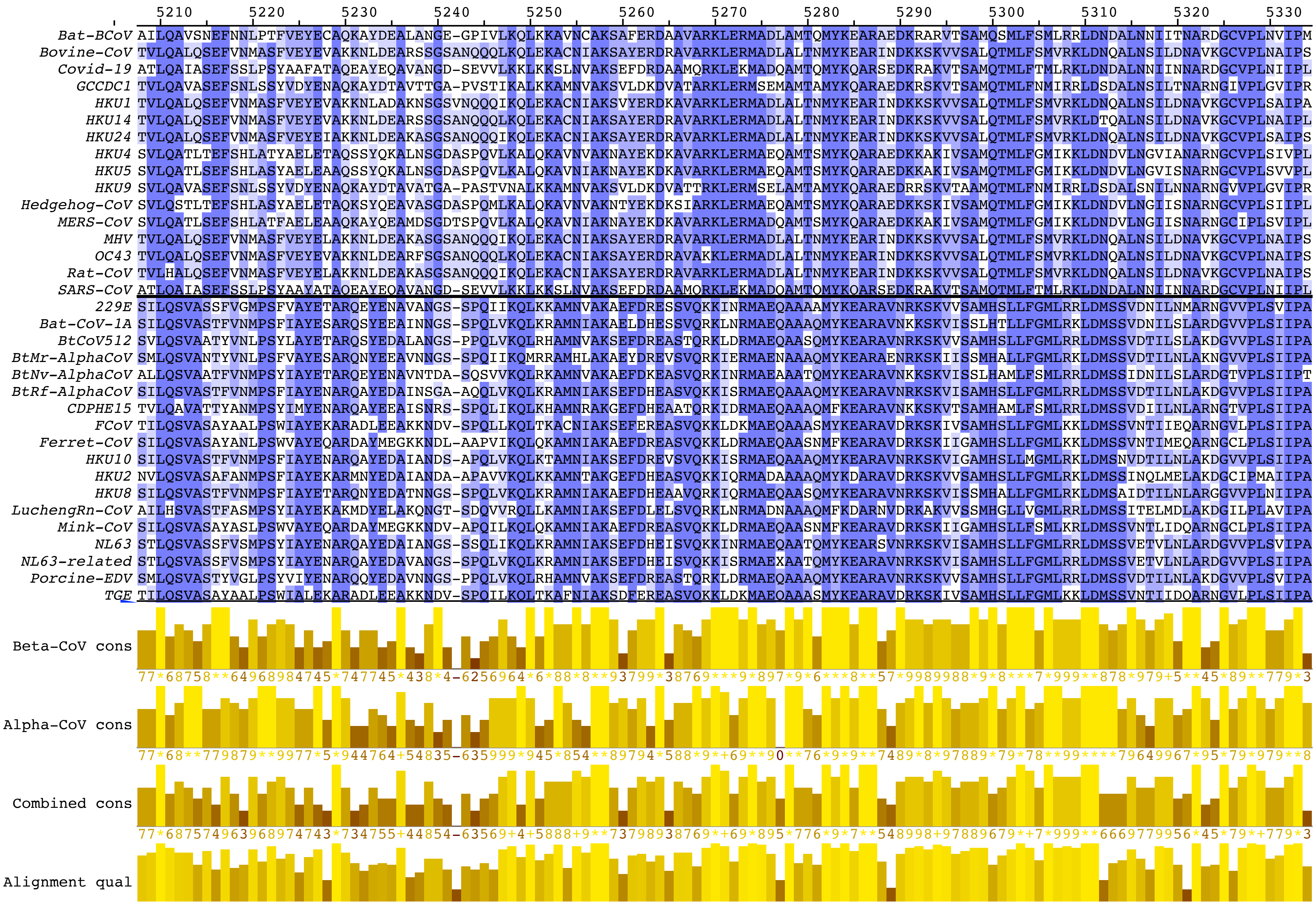

5210 5220 5230 5240 5250 5260 5270 5280 5290 5300 5310 5320 5330
Bat-BCoV AILQAVSNEFNNLPTFVEYECAQKAYDEALANGE-GPIVLKQLKKAVNCAKSAFERDAAVARKLERMADLAMTQMYKEARAEDKRARVTSAMQSMLFSMLRRLDNDALNNIITNARDGCVPLNVIPM
Bovine-CoV TVLQALQSEFVNMASFVEYEVAKKNLDEARSSGSANQQQLKQLEKACNIAKSAYERDRAVARKLERMADLALTNMYKEARINDKKSKVVSALQTMLFSMVRKLDNQALNSILDNAVKGCVPLNAIPS
Covid-19 ATLQAIASEFSSLPSYAAFATAQEAYEQAVANGD-SEVVLKKLKKSLNVAKSEFDRDAAMQRKLEKMADQAMTQMYKQARSEDKRAKVTSAMQTMLFTMLRKLDNDALNNIINNARDGCVPLNIIPL
GCCDC1 TVLQAVASEFSNLSSYVDYENAQKAYDTAVTTGA-PVSTIKALKKAMNVAKSVLDKDVATARKLERMSEMAMTAMYKQARAEDKRSKVTSAMQTMLFNMIRRLDSDALNSILTNARNGIVPLGVIPR
HKU1 TVLQALQSEFVNMASFVEYEVAKKNLADAKNSGSVNQQQIKQLEKACNIAKSVYERDKAVARKLERMADLALTNMYKEARINDKKSKVVSALQTMLFSMVRKLDNQALNSILDNAVKGCVPLSAIPA
HKU14 TVLQALQSEFVNMASFVEYEVAKKNLDEARSSGSANQQQLKQLEKACNIAKSAYERDRAVARKLERMADLALTNMYKEARINDKKSKVVSALQTMLFSMVRKLDTQALNSILDNAVKGCVPLNAIPL
HKU24 TVLQALQSEFVNMASFVEYEIAKKNLDEAKASGSANQQQIKQLEKACNIAKSAYERDRAVARKLERMADLALTNMYKEARINDKKSKVVSALQTMLFSMVRKLDNQALNSILDNAVKGCVPLSAIPS
HKU4 SVLQATLTEFSHLATYAELETAQSSYQKALNSGDASPQVLKALQKAVNVAKNAYEKDKAVARKLERMAEQAMTSMYKQARAEDKKAKIVSAMQTMLFGMIKKLDNDVLNGVIANARNGCVPLSIVPL
HKU5 SVLQATLSEFSHLASYAELEAAQSSYQKALNSGDASPQVLKALQKAVNIAKNAYEKDKAVARKLERMAEQAMTSMYKQARAEDKKAKIVSAMQTMLFGMIKKLDNDVLNGVISNARNGCVPLSVVPL
HKU9 SVLQAVASEFSNLSSYVDYENAQKAYDTAVATGA-PASTVNALKKAMNVAKSVLDKDVATTRKLERMSELAMTAMYKQARAEDRRSKVTAAMQTMLFNMIRRLDSDALSNILNNARNGVVPLGVIPR
Hedgehog-CoV SVLQSTLTEFSHLASYAELETAQKSYQEAVASGDASPQMLKALQKAVNVAKNTYEKDKSIARKLERMAEQAMTSMYKQARAEDKKSKIVSAMQTMLFGMIKKLDNDVLNGIISNARNGCVPLSIIPL
MERS-CoV SVLQATLSEFSHLATFAELEAAQKAYQEAMDSGDTSPQVLKALQKAVNIAKNAYEKDKAVARKLERMADQAMTSMYKQARAEDKKAKIVSAMQTMLFGMIKKLDNDVLNGIISNARNGCIPLSVIPL
MHV TVLQALQSEFVNMASFVEYELAKKNLDEAKASGSANQQQIKQLEKACNIAKSAYERDRAVARKLERMADLALTNMYKEARINDKKSKVVSALQTMLFSMVRKLDNQALNSILDNAVKGCVPLNAIPS
OC43 TVLQALQSEFVNMASFVEYEVAKKNLDEARFSGSANQQQLKQLEKACNIAKSAYERDRAVAKKLERMADLALTNMYKEARINDKKSKVVSALQTMLFSMVRKLDNQALNSILDNAVKGCVPLNAIPS
Rat-CoV TVLHALQSEFVNMASFVEYELAKKNLDEAKASGSANQQQIKQLEKACNIAKSAYERDRAVARKLERMADLALTNMYKEARINDKKSKVVSALQTMLFSMVRKLDNQALNSILDNAVKGCVPLNAIPS
SARS-CoV ATLQAIASEFSSLPSYAAYATAQEAYEQAVANGD-SEVVLKKLKKSLNVAKSEFDRDAAMQRKLEKMADQAMTQMYKQARSEDKRAKVTSAMQTMLFTMLRKLDNDALNNIINNARDGCVPLNIIPL
229E SILQSVASSFVGMPSFVAYETARQEYENAVANGS-SPQIIKQLKKAMNVAKAEFDRESSVQKKINRMAEQAAAAMYKEARAVNRKSKVVSAMHSLLFGMLRRLDMSSVDNILNMARNGVVPLSVIPA
Bat-CoV-1A SILQSVASTFVNMPSFIAYESARQSYEEAINNGS-SPQLVKQLKRAMNIAKAELDHESSVQKKLNRMAEQAAAQMYKEARAVNKKSKVISSLHTLLFGMLRRLDMSSVDNILSLARDGVVPLSIIPA
BtCoV512 SVLQSVAATYVNLPSYLAYETARQSYEDALANGS-PPQLVKQLRHAMNVAKSEFDREASTQRKLDRMAEQAASQMYKEARAVNRKSKVVSAMHSLLFGMLRRLDMSSVDTILSLAKDGVVPLSIIPA
BtMr-AlphaCoV SMLQSVANTYVNLPSFVAYESARQNYEEAVNNGS-SPQIIKQMRRAMHLAKAEYDREVSVQRKIERMAENAAAQMYKEARAENRKSKIISSMHALLFGMLRKLDMSSVDTILNLAKNGVVPLSIIPA
BtNv-AlphaCoV ALLQSVAATFVNMPSYIAYETARQEYENAVNTDA-SQSVVKQLRKAMNVAKAEFDKEASVQRKINRMAEAAATQMYKEARAVNKKSKVISSLHAMLFSMLRRLDMSSIDNILSLARDGTVPLSIIPT
BtRf-AlphaCoV SILQSVASTFVNMPSFIAYENARQAYEDAINSGA-AQQLVKQLKRAMNIAKSEFDHEASVQKKISRMAEQAAAQMYKEARAVNRKSKVISAMHSLLFGMLRRLDMSSVDTILNLAKDGVVPLSIIPA
CDPHE15 TVLQAVATTYANMPSYIMYENARQAYEEAISNRS-SPQLIKQLKHAMNRAKGEFDHEAATQRKIDRMAEQAAAQMFKEARAVNKKSKVTSAMHAMLFSMLRRLDMSSVDIILNLARNGTVPLSIIPA
FCoV TILQSVASAYAALPSWIAYEKARADLEEAKKNDV-SPQLLKQLTKACNIAKSEFEREASVQKKLDKMAEQAASMYKEARAVDRKSKIVSAMHSLLFGMLKKLDMSSVNTIIEQARNGVLPLSIIPA
Ferret-CoV SILQSVASAYANLPSWVAYEQARDAYMEGKKNDL-AAPVIKQLQKAMNIAKAEFDREASVQKKLDRMAEQAASNMFKEARAVDRKSKIIGAMHSLLFGMLKKLDMSSVNTIMEQARNGCLPLSIIPA
HKU10 SILQSVASTFVNMPSFIAYENARQAYEDAIANDS-APQLVKQLKTAMNIAKSEFDREVSVQKKISRMAEQAAAQMYKEARAVNRKSKVIGAMHSLLMGMLRKLDMSNVDTILNLAKDGVVPLSIIPA
HKU2 NVLQSVASAFANMPSFIAYEKARMNYEDAIANDA-APAVVKQLKKAMNTAKGEFDHEASVQKKIQRMADAAAAQMYKDARAVDRKSKVVSAMHSLLFGMLRKLDMSSINQLMELAKDGCIPMAIIPA
HKU8 SILQSVASTFVNMPSFIAYETARQNYEDATNNGS-SPQLVKQLKRAMNIAKAEFDHEAAVQRKIQRMAEQAAASQMYKEARAVNRKSKVISSMHALLFGMLRKLDMSAIDTILNLARGGVVPLNIIPA
LuchengRn-CoV AILHSVASTFASMPSYIAYEKAKMDYELAKQNGT-SDQVVRQLLKAMNIAKSEFDLELSVQRKLNRMADNAAAQMFKDARNVDRKAKVVSSMHGLLVGMLRRLDMSSITELMDLAKDGILPLAVIPA
Mink-CoV SILQSVASAYASLPSWVAYEQARDAYMEGKKNDV-APQILKQLQKAMNIAKAEFDREASVQKKLDRMAEQAASNMFKEARAVDRKSKIIGAMHSLLFSMLKRLDMSSVNTLIDQARNGCLPLSIIPA
NL63 STLQSVASSFVSMPSYIAYENARQAYEDAIANGS-SSQLIKQLKRAMNIAKSEFDHEISVQKKINRMAEQAATQMYKEARSVNRKSKVISAMHSLLFGMLRRLDMSSVETVLNLARDGVVPLSVIPA
NL63-related STLQSVASSFVSMPSYIAYENARQAYEDAVANGS-SPQLVKQLKRAMNIAKSEFDHEISVQKKISRMAEXAATQMYKEARAVNRKSKVISAMHSLLFGMLRRLDMSSVETVLNLARDGVVPLSIIPA
Porcine-EDV SMLQSVASTYVGLPSYVIYENARQQYEDAVNNGS-PPQLVKQLRHAMNVAKSEFDREASTQRKLDRMAEQAAAQMYKEARAVNRKSKVVSAMHSLLFGMLRRLDMSSVDTILNLAKDGVVPLSVIPA
TGE TILQSVASAYAALPSWIALEKARADLEEAKKNDV-SPQILKQLTKAFNIAKSDFEREASVQKKLDKMAEQAAASMYKEARAVDRKSKIVSAMHSLLFGMLKKLDMSSVNTIIDQARNGVLPLSIIPA
Beta-CoV cons
77*68758**64968984745*747745*438*4-6256964*6*88*8**93799*38769***9*897*9*6***8**57*9989988*9*8***7*999**878*979+5**45*89**779*3
Alpha-CoV cons
77*68**779879**9977*5*944764+54835-635999*945*854**89794*588*9*+69**0**76*9*9**7489*8*97889*79*78**99****79649967*95*79*979**8
Combined cons
77*687574963968974743*734755+44854-63569+4+5888+9**737989387 69*+69*895*776*9*7**548998+97889679*+7*999**6669779956*45*79*+779*3
Alignment qual

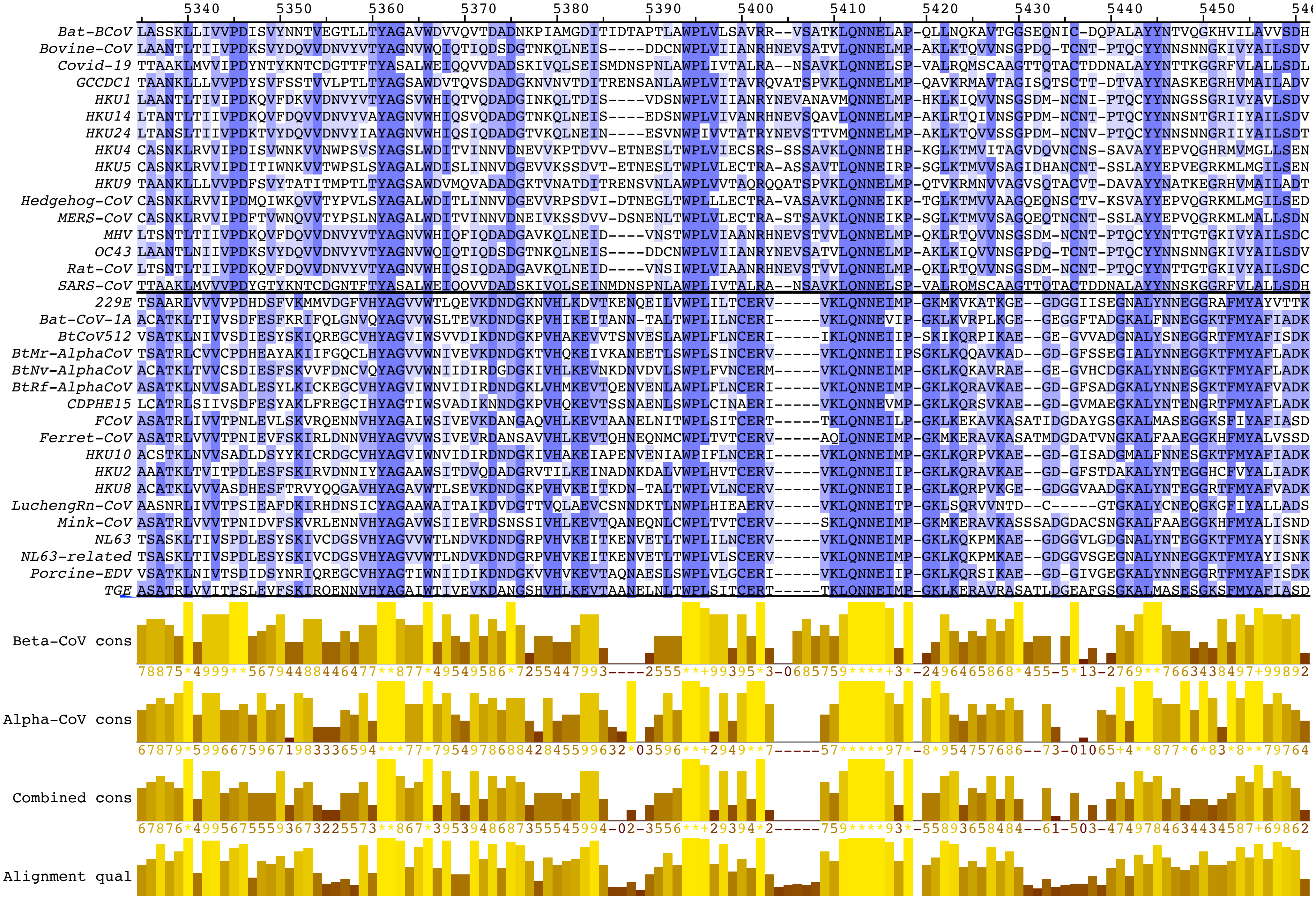
5340 5350 5360 5370 5380 5390 5400 5410 5420 5430 5440 5450
Bat-BCoV LASSKLLIVVPDISVYNNTVEGTLLTYAGAVWDVVQVTDADNKPIAMGDITIDTAPTLAWPLVLSAVRR--VSATKLQNNELAP-QLLNQKAVTGGSEQNIC-DQPALAYYNTVQGKHVILAVVSDH
Bovine-CoV LAANTLTIIVPDKSVYDQVVDNVYVTYAGNVWQIQTIQDSDGTNKQLNEIS----DDCNWPLVIIANRHNEVSATVLQNNELMP-AKLKTQVVNSGPDQ-TCNT-PTQCYYNNSNNGKIVYAILSDV
Covid-19 TTAAKLMVVIPDYNTYKNTCDGTTFTYASALWEIQQVVDADSKIVQLSEISMDNSPNLAWPLIVTALRA--NSAVKLQNNELSP-VALRQMSCAAGTTQTACTDDNALAYYNTTKGGRFVLALLSDL
GCCDC1 TAANKLLLVVPDYSVFSSTVVLPTLTYAGSAWDVTQVSDADGKNVNVTDITRENSANLAWPLVITAVRQVATSPVKLQNNELMP-QAVKRMAVTAGISQTSCTT-DTVAYYNASKEGRHVMAILADV
HKU1 LAANTLTIVIPDKQVFDKVVDNVYVTYAGSVWHIQTVQDADGINKQLTDIS----VDSNWPLVIIANRYNEVANAVMQNNELMP-HKLKIQVVNSGSDM-NCNI-PTQCYYNNGSSGRIVYAVLSDV
HKU14 LTANTLTIIVPDKQVFDQVVDNVYVAYAGNVWHIQSVQDADGTNKQLNEIS----EDSNWPLVIVANRHNEVSQAVLQNNELMP-AKLRTQIVNSGPDM-NCNT-PTQCYYNNSNTGRIIYAILSDV
HKU24 LTANSLTIIVPDKTVYDQVVDNVYIAYAGNVWHIQSIQDADGTVKQLNEIN----ESVNWPIVVTATRYNEVSTTVMQNNELMP-AKLKTQVVSSGPDM-NCNV-PTQCYYNNSNNGRIIYAILSDT
HKU4 CASNKLRVVIPDISVWNKVVNWPSVSYAGSLWDITVINNVDNEVVKPTDVV-ETNESLTWPLVIECSRS-SSSAVKLQNNEIHP-KGLKTMVITAGVDQVNCNS-SAVAYYEPVQGHRMVMGLLSEN
HKU5 CASNKLRVVIPDITIWNKVVTWPSLSYAGALWDISLINNVDGEVVKSSDVT-ETNESLTWPLVLECTRA-ASSAVTLQNNEIRP-SGLKTMVVSAGIDHANCNT-SSLAYYEPVEGRKMLMGILSEN
HKU9 TAANKLLLVVPDFSVYTATITMPTLTYAGSAWDVMQVADADGKTVNATDITRENSVNLAWPLVVTAQRQQATSPVKLQNNELMP-QTVKRMNVVAGVSQTACVT-DAVAYYNATKEGRHVMAILADT
Hedgehog-CoV CASNKLRVVIPDMQIWKQVVTYPVLSYAGALWDITLINNVDGEVVRPSDVI-DTNEGLTWPLLLECTRA-VASAVKLQNNEIKP-TGLKTMVVAAGQEQNSCTV-KSVAYYEPVQGRKMLMGILSED
MERS-CoV CASNKLRVVIPDFTVWNQVVTYPSLNYAGALWDITVINNVDNEIVKSSDVV-DSNENLTWPLVLECTRA-STSAVKLQNNEIKP-SGLKTMVVSAGQEQTNCNT-SSLAYYEPVQGRKMLMALLSDN
MHV LTSNTLTIIVPDKQVFDQVVDNVYVTYAGNVWHIQFIQDADGAVKQLNEID----VNSTWPLVIAANRHNEVSTVVLQNNELMP-QKLRTQVVNSGSDM-NCNT-PTQCYYNTTGTGKIVYAILSDC
OC43 LAANTLNIIVPDKSVYDQVVDNVYVTYAGNVWQIQTIQDSDGTNKQLNEIS----DDCNWPLVIIANRYNEVSATVLQNNELMP-AKLKIQVVNSGPDQ-TCNT-PTQCYYNNSNNGKIVYAILSDV
Rat-CoV LTSNTLTIIVPDKQVFDQVVDNVYVTYAGNVWHIQSIQDADGAVKQLNEID----VNSIWPLVIAANRHNEVSTVVLQNNELMP-QKLRTQVVNSGSDM-NCNT-PTQCYYNTTGTGKIVYAILSDC
SARS-CoV TTAAKLMVVVPDYGTYKNTCDGNTFTYASALWEIQQVVDADSKIVQLSEINMDNSPNLAWPLIVTALRA--NSAVKLQNNELSP-VALRQMSCAAGTTQTACTDDNALAYYNNSKGGRFVLALLSDH
229E TSAARLVVVVPDHDSFVKMMVDGFVHYAGVVWTLQEVKDNDGKNVHLKDVTKENQEILVWPLILTCERV-----VKLQNNEIMP-GKMKVKATKGE--GDGGIISEGNALYNNEGGRAFMYAYVTTK
Bat-CoV-1A ACATKLTIVVSDFESFKRIFQLGNVQYAGVVWSLTEVKDNDGKPVHIKEITANN-TALTWPLILNCERI-----VKLQNNEVIP-GKLKVRPLKGE--GEGGFTADGKALFNNEGGKTFMYAFIADK
BtCoV512 VSATKLNIVVSDIESYSKIQREGCVHYAGVIWSVVDIKDNDGKPVHAKEVVTSNVESLAWPLFLNCERI-----IKLQNNEIIP-SKIKQRPIKAE--GE-GVVADGNALYSNEGGRTFMYAFISDK
BtMr-AlphaCoV TSATRLCVVCPDHEAYAKIIFGQCLHYAGVVWNIVEVKDNDGKTVHQKEIVKANEETLSWPLSINCERV-----VKLQNNEIIPSGKLKQQAVKAD--GD-GFSSEGIALYNNEGGKTFMYAFLADK
BtNv-AlphaCoV ACATKLTVVCSDIESFSKVVFDNCVQYAGVVWNIIDIRDGDGKIVHLKEVNKDNVDVLSWPLFVNCERM-----VKLQNNEIMP-GKLKQKAVRAE--GE-GVHCDGKALYNNEGGKTFMYAFLADK
BtRf-AlphaCoV ASATKLNVVSADLESYLKICKEGCVHYAGVIWNVIDIRDNDGKLVHMKEVTQENVENLAWPLFLNCERI-----VKLQNNEIMP-GKLKQRAVKAE--GD-GFSADGKALYNNESGKTFMYAFVADK
CDPHE15 LCATRLSIIVSDFESYAKLFREGCIHYAGTIWSVADIKNNDGKPVHQKEVTSSNAENLSWPLCINAERI-----VKLQNNEVMP-GKLKQRSVKAE--GD-GVMAEGKALYNTENGRTFMYAFLADK
FCoV ASATRLIVVTPNLEVLSKVRQENNVHYAGAIWSIVEVKDANGAQVHLKEVTAANELNITWPLSITCERT-----TKLQNNEILP-GKLKEKAVKASATIDGDAYGSGKALMASEGGKSFIYAFIASD
Ferret-CoV ASATRLVVVTPNIEVFSKIRLDNNVHYAGVVWSIVEVRDANSAVVHLKEVTQHNEQNMCWPLTVTCERV-----AQLQNNEIMP-GKMKERAVKASATMDGDATVNGKALFAAEGGKHFMYALVSSD
HKU10 ACSTKLNVVSADLDSYYKICRDGCVHYAGVIWNVIDIRDNDGKIVHAKEIAPENVENIAWPIFLNCERI-----VKLQNNEIMP-GKLKQRPVKAE--GD-GISADGMALFNNESGKTFMYAFIADK
HKU2 AAATKLTVITPDLESFSKIRVDNNIYYAGAAWSITDVQDADGRVTILKEINADNKDALVWPLHVTCERV-----VKLQNNEIIP-GKLKQRAVKAE--GD-GFSTDAKALYNTEGGHCFVYALIADK
HKU8 ACATKLVVVASDHESFTRVYQQGAVHYAGAVWTLSEVKDNDGKPVHVKEITKDN-TALTWPLVLNCERV-----VKLQNNEIMP-GKLKQRPVKGE--GDGGVAADGKALYNTEGGRTFMYAFVADK
LuchengRn-CoV AASNRLIVVTPSIEAFDKIRHDNSICYAGAAWAITAIKDVDGTTVQLAEVCSNNDKTLNWPLHIEAERV-----VKLQNNEITP-GKLSQRVVNTD--C-----GTGKALYCNEQGKGFIYALLADS
Mink-CoV ASATRLVVVTPNIDVFSKVRLENNVHYAGAVWSIIEVRDSNSSIVHLKEVTQANEQNLCWPLTVTCERV-----SKLQNNEIMP-GKMKERAVKASSSADGDACSNGKALFAAEGGKHFMYALISND
NL63 TSASKLTIVSPDLESYSKIVCDGSVHYAGVVWTLNDVKDNDGRPVHVKEITKENVETLTWPLILNCERV-----VKLQNNEIMP-GKLKQKPMKAE--GDGGVLGDGNALYNTEGGKTFMYAYISNK
NL63-related TSASKLTIVSPDLESYSKIVCDGSVHYAGVVWTLNDVKDNDGRPVHVKEITKENVETLTWPLVLSCERV-----VKLQNNEIMP-GKLKQKPMKAE--GDGGVSGEGNALYNNEGGKTFMYAYISNK
Porcine-EDV VSATKLNIVTSDIDSYNRIQREGCVHYAGTIWNIIDIKDNDGKVVHVKEVTAQNAESLSWPLVLGCERI-----VKLQNNEIIP-GKLKQRSIKAE--GD-GIVGEGKALYNNEGGRTFMYAFISDK
TGE ASATRLVVITPSLEVFSKIRQENNVHYAGAIWTIVEVKDANGSHVHLKEVTAANELNLTWPLSITCERT-----TKLQNNEIMP-GKLKERAVRASATLDGEAFGSGKALMASESGKSFMYAFIASD
Beta-CoV cons
78875*4999**5679448844647**877*49549586*7255447993----2555**+99395*3-0685759****+3*-2496465868*455-5*13-2769**766343849 7+99892
Alpha-CoV cons
67879*59966759671983336594***77*7954978688428455996 32*03596**+2949**7-----57*****97*-8*954757686--73-01065+4**877*6*83*8**79764
Combined cons
67876*4995675559367322557 3**867*395394868735554599 4-02-3556**+29394*2-----759****93*-55893658484--61-503-474978463443458 7+69862
Alignment qual

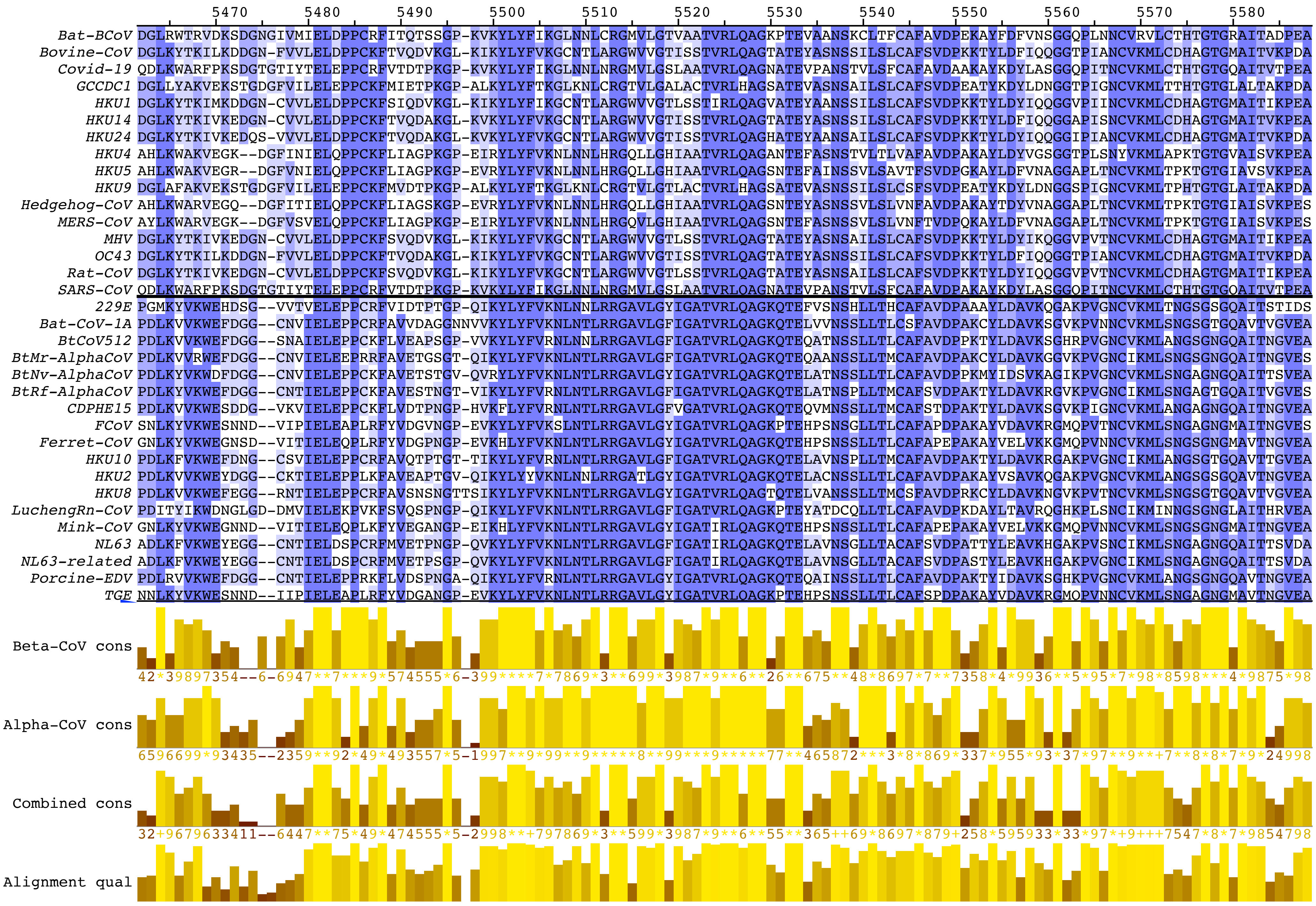
5470 5480 5490 5500 5510 5520 5530 5540 5550 5560 5570 5580
Bat-BCoV DGLRWTRVDKSDGNGIVMIELEDPPCRFITQTSSGP-KVKYLYFIKGLNNLCRGMVLGTVAATVRLQAGKPTEVAANSKCLTFCAFAVDPEKAYFDFVNSGGQPLNNCVRVLCTHTGTGRAITADPEA
Bovine-CoV DGLKYTKILKDDGN-FVVLELDPPCKFTVQDVKGL-KIKYLYFVKGCNTLARGWVVGTISSTVRLQAGTATEYASNSSILSLCAFSVDPKKTYLDFIQQGGTPIANCVKMLCDHAGTGMAITVKPDA
Covid-19 QDLKWARFPKSDGTGTIYTELEPPCRFVTDTPKGP-KVKYLYFIKGLNNLNRGMVLGSLAATVRLQAGNATEVPANSTVLSFCAFAVDAAKAYKDYLASGGQPITNCVKMLCTHTGTGQAITVTPEA
GCCDC1 DGLLYAKVEKSTGDGFVILELEPPCKFMIETPKGP-ALKYLYFTKGLKNLCRGTVLGALACTVRLHAGSATEVASNSAILSLCAFSVDPEATYKDYLDNGGTPIGNCVKMLTTHTGTGLALTAKPDA
HKU1 DGLKYTKIMKDDGN-CVVLELDPPCKFSIQDVKGL-KIKYLYFIKGCNTLARGWVVGTLSSTIRLQAGVATEYAANSSILSLCAFSVDPKKTYLDYIQQGGVPIINCVKMLCDHAGTGMAITIKPEA
HKU14 DGLKYTKIVKEDGN-CVVLELDPPCKFTVQDAKGL-KVKYLYFVKGCNTLARGWVVGTISSTVRLQAGTATEYASNSSILSLCAFSVDPKKTYLDFIQQGGAPISNCVKMLCDHAGTGMAITVKPEA
HKU24 DGLKYTKIVKEDQS-VVVLELDPPCKFTVQDAKGL-KVKYLYFVKGCNTLARGWVVGTISSTVRLQAGHATEYAANSAILSLCAFSVDPKKTYLDYIQQGGIPIANCVKMLCDHAGTGMAITVKPDA
HKU4 AHLKWAKVEGK--DGFINIELQPPCKFLIAGPKGP-EIRYLYFVKNLNNLHRGQLLGHIAATVRLQAGANTEFASNSTVLTLVAFAVDPAKAYLDYVGSGGTPLSNYVKMLAPKTGTGVAISVKPEA
HKU5 AHLKWAKVEGR--DGFVNIELQPPCKFLIAGPKGP-EVRYLYFVKNLNNLHRGQLLGHIAATVRLQAGSNTEFAINSSVLSAVTFSVDPGKAYLDFVNAGGAPLTNCVKMLTPKTGTGIAVSVKPEA
HKU9 DGLAFAKVEKSTGDGFVILELEPPCKFMVDTPKGP-ALKYLYFTKGLKNLCRGTVLGTLACTVRLHAGSATEVASNSSILSLCSFSVDPEATYKDYLDNGGSPIGNCVKMLTPHTGTGLAITAKPDA
Hedgehog-CoV AHLKWARVEGQ--DGFITIELQPPCKFLIAGSKGP-EVRYLYFVKNLNNLHRGQLLGHIAATVRLQAGSNTEYASNSSVLSLVNFAVDPAKAYTDYVNAGGAPLTNCVKMLTPKTGTGIAISVKPES
MERS-CoV AYLKWARVEGK--DGFVSVELQPPCKFLIAGPKGP-EIRYLYFVKNLNNLHRGQVLGHIAATVRLQAGSNTEFASNSSVLSLVNFTVDPQKAYLDFVNAGGAPLTNCVKMLTPKTGTGIAISVKPES
MHV DGLKYTKIVKEDGN-CVVLELDPPCKFSVQDVKGL-KIKYLYFVKGCNTLARGWVVGTLSSTVRLQAGTATEYASNSAILSLCAFSVDPKKTYLDYIKQGGVPVTNCVKMLCDHAGTGMAITIKPEA
OC43 DGLKYTKILKDDGN-FVVLELDPPCKFTVQDAKGL-KIKYLYFVKGCNTLARGWVVGTISSTVRLQAGTATEYASNSSILSLCAFSVDPKKTYLDFIQQGGTPIANCVKMLCDHAGTGMAITVKPDA
Rat-CoV DGLKYTKIVKEDGN-CVVLELDPPCKFSVQDVKGL-KIKYLYFVKGCNTLARGWVVGTLSSTVRLQAGTATEYASNSAILSLCAFSVDPKKTYLDYIQQGGVPVTNCVKMLCDHAGTGMAITIKPEA
SARS-CoV QDLKWARFPKSDGTGTIYTELEPPCRFVTDTPKGP-KVKYLYFIKGLNNLNRGMVLGSLAATVRLQAGNATEVPANSTVLSFCAFAVDPAKAYKDYLASGGQPITNCVKMLCTHTGTGQAITVTPEA
229E PGMKYVKWEHDSG--VVTVELEPPCRFVIDTPTGP-QIKYLYFVKNLNNLRRGAVLGYIGATVRLQAGKQTEFVSNSHLLTHCAFAVDPAAAYLDAVKQGAKPVGNCVKMLTNGSGSGQAITSTIDS
Bat-CoV-1A PDLKVVKWEFDGG--CNVIELEPPCRFAVVDAGGNNVVKYLYFVRNLNTLRRGAVLGFIGATVRLQAGKQTELVVNSSLLTLCSFAVDPAKCYLDAVKSGVKPVNNCVKMLSNGSGTGQAVTVGVEA
BtCoV512 PDLKVVKWEFDGG--SNAIELEPPCKFLVEAPSGP-VVKYLYFVRNLNNLRRGAVLGFIGATVRLQAGKQTEQATNSSLLTLCAFAVDPPKTYLDAVKSGHRPVGNCVKMLANGSGNGQAITNGVEA
BtMr-AlphaCoV PDLKVVRWEFDGG--CNVIELEEPRRFAVETGSGT-QIKYLYFVKNLNTLRRGAVLGFIGATVRLQAGKQTEQAANSSLLTMCAFAVDPAKCYLDAVKGGVKPVGNCIKMLSNGSGNGQAITNGVES
BtNv-AlphaCoV PDLKYVKWDFDGG--CNVIELEPPCKFAVETSTGV-QVRYLYFVKNLNTLRRGAVLGYIGATVRLQAGKQTELATNSSLLTLCAFAVDPPKMYIDSVKAGIKPVGNCVKMLSNGAGNGQAITTSVEA
BtRf-AlphaCoV PDLKYVKWEFDGG--CNTIELEPPCKFAVESTNGT-VIKYLYFVRNLNTLRRGAVLGFIGATVRLQAGKQTELATNSPLLTMCAFSVDPAKTYLDAVKRGVKPVGNCIKMLSNGAGTGQAITVGVES
CDPHE15 PDLKVVKWESDDG--VKVIELEPPCKFLVDTPNGP-HVKFLYFVRNLNTLRRGAVLGFVGATVRLQAGKQTEQVMNSSLLTMCAFSTDPAKTYLDAVKSGVKPIGNCVKMLANGAGNGQAITNGVEA
FCoV SNLKYVKWESNND--VIPIELEAPLRFYVDGVNGP-EVKYLYFVKSLNTLRRGAVLGYIGATVRLQAGKPTEHPSNSGLLTLCAFAPDPAKAYVDAVKRGMQPVTNCVKMLSNGAGNGMAITNGVES
Ferret-CoV GNLKYVKWEGNSD--VITIELEQPLRFYVDGPNGP-EVKHLYFVKNLNTLRRGAVLGYIGATVRLQAGKQTEHPSNSSLLTLCAFAPEPAKAYVELVKKGMQPVNNCVKMLSNGSGNGMAVTNGVEA
HKU10 PDLKFVKWEFDNG--CSVIELEPPCRFAVQTPTGT-TIKYLYFVRNLNTLRRGAVLGFIGATVRLQAGKQTELAVNSPLLTMCAFAVDPAKTYLDAVKRGAKPVGNCIKMLANGSGTGQAVTTGVEA
HKU2 PDLKVVKWEYDGG--CKTIELEPPLKFAVEAPTGV-QIKYLYYVKNLNNLRRGATLGYIGATVRLQAGKQTELACNSSLLTLCAFAVDPAKAYVSAVKQGAKPVGNCVKMLANGSGSGQAVTNGVEA
HKU8 PDLKVVKWEFEGG--RNTIELEPPCRFAVSNSNGTTSIKYLYFVKNLNTLRRGAVLGYIGATVRLQAGTQTELVANSSLLTMCSFAVDPRKCYLDAVKNGVKPVGNCVKMLSNGSGTGQAVTVGVEA
LuchengRn-CoV PDITYIKWDNGLGD-DMVIELEKPVKFSVQSPNGP-QIKYLYFVKNLNTLRRGAVLGFIGATVRLQAGKPTEYATDCQLLTLCAFAVDPKDAYLTAVRQGHKPLSNCIKMINNGSGNGLAITHRVEA
Mink-CoV GNLKYVKWEGNND--VITIELEQPLKFYVEGANGP-EIKHLYFVKNLNTLRRGAVLGYIGATIRLQAGKQTEHPSNSSLLTLCAFAPEPAKAYVELVKKGMQPVNNCVKMLSNGSGNGMAITNGVEA
NL63 ADLKFVKWEYEGG--CNTIELDSPCRFMVETPNGP-QVKYLYFVKNLNTLRRGAVLGFIGATIRLQAGKQTELAVNSGLLTACAFSVDPATTYLEAVKHGAKPVSNCIKMLSNGAGNGQAITTSVDA
NL63-related ADLKFVKWEYEGG--CNTIELDSPCRFMVETPSGP-QVKYLYFVKNLNTLRRGAVLGFIGATIRLQAGKQTELAVNSGLLTACAFSVDPASTYLEAVKHGAKPVGNCIKMLSNGAGNGQAITTSVDA
Porcine-EDV PDLRVVKWEFDGG--CNTIELEPPRKFLVDSPNGA-QIKYLYFVRNLNTLRRGAVLGYIGATVRLQAGKQTEQAINSSLLTLCAFAVDPAKTYIDAVKSGHKPVGNCVKMLANGSGNGQAVTNGVEA
TGE NNLKYVKWESNND--IIPIELEAPLRFYVDGANGP-EVKYLYFVKNLNTLRRGAVLGYIGATVRLQAGKPTEHPSNSSLLTLCAFSPDPAKAYVDAVKRGMQPVNNCVKMLSNGAGNGMAVTNGVEA
Beta-CoV cons
42*39897354--6-6947**7***9*574555*6-399****7*7869*3**699*3987*9**6**26**675**48*8697*7**7358*4*9936**5*95*7*98*8598***4*9875*98
Alpha-CoV cons
6596699*93435--2359**92*49*493557*5-1997**9*99**9*****8**99***9*****77**465872***3*8*869*337*955*93*37*97**9**+7**8*8*7*9*24998
Combined cons
32+9679633411--6447**75*49*474555*5-2998**+797869*3**599*3987*9**6**55**365++69*8697*879+258*595933*33*97*+9+++7547*8*7*9854798
Alignment qual

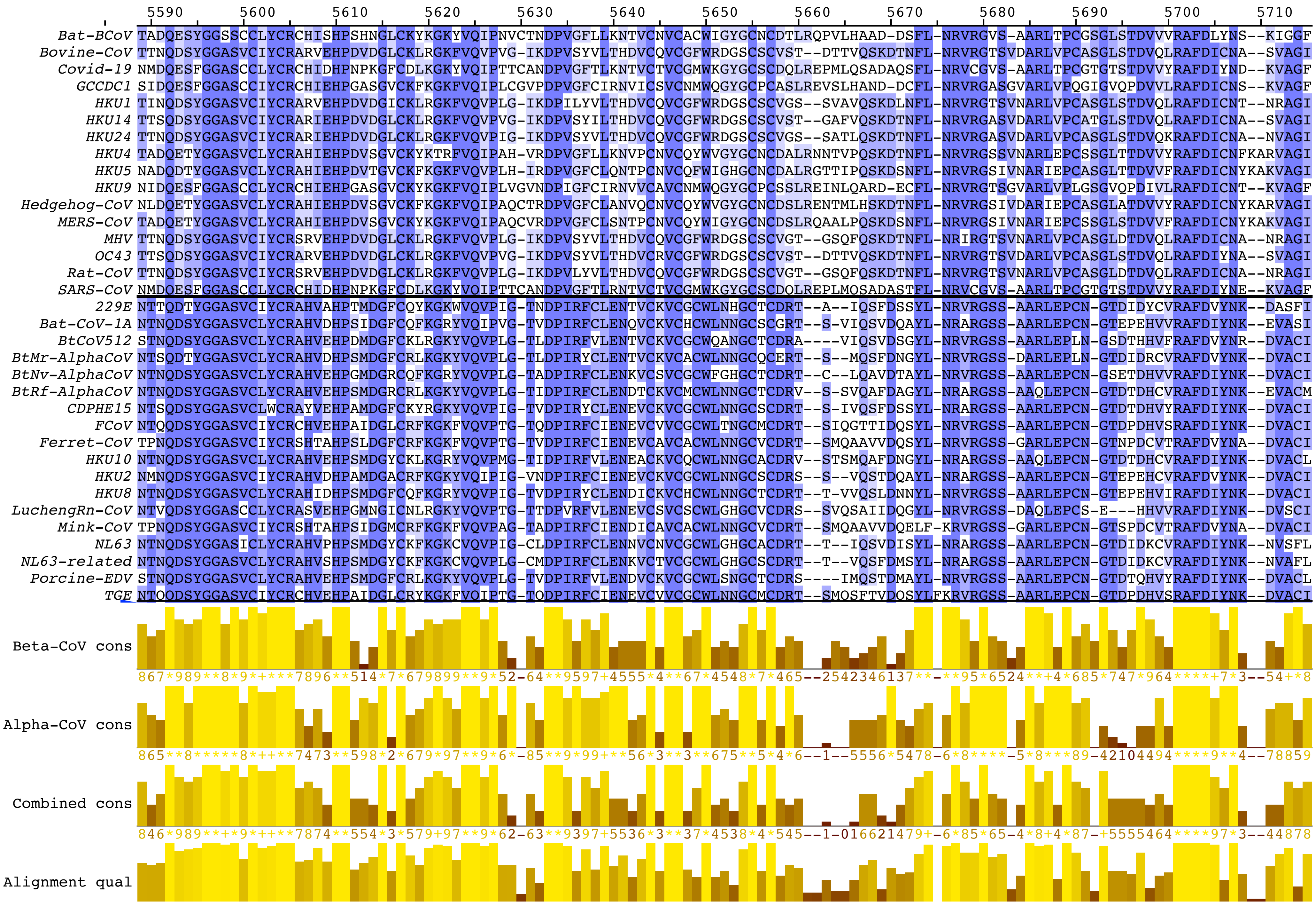
5590
5600
5610
5620
5630
5640
5650
5660
5670
5680
5690
5700
5710
Bat-BCoV
Bovine-CoV
Covid-19
GCCDC1
HKU1
HKU14
HKU24
HKU4
HKU5
HKU9
Hedgehog-CoV
MERS-CoV
MHV
OC43
Rat-CoV
SARS-CoV
229E
Bat-CoV-1A
BtCoV512
BtMr-AlphaCoV
BtNv-AlphaCoV
BtRf-AlphaCoV
CDPHE15
FCoV
Ferret-CoV
HKU10
HKU2
HKU8
LuchengRn-CoV
Mink-CoV
NL63
NL63-related
Porcine-EDV
TGE
Beta-CoV cons
Alpha-CoV cons
Combined cons
Alignment qual

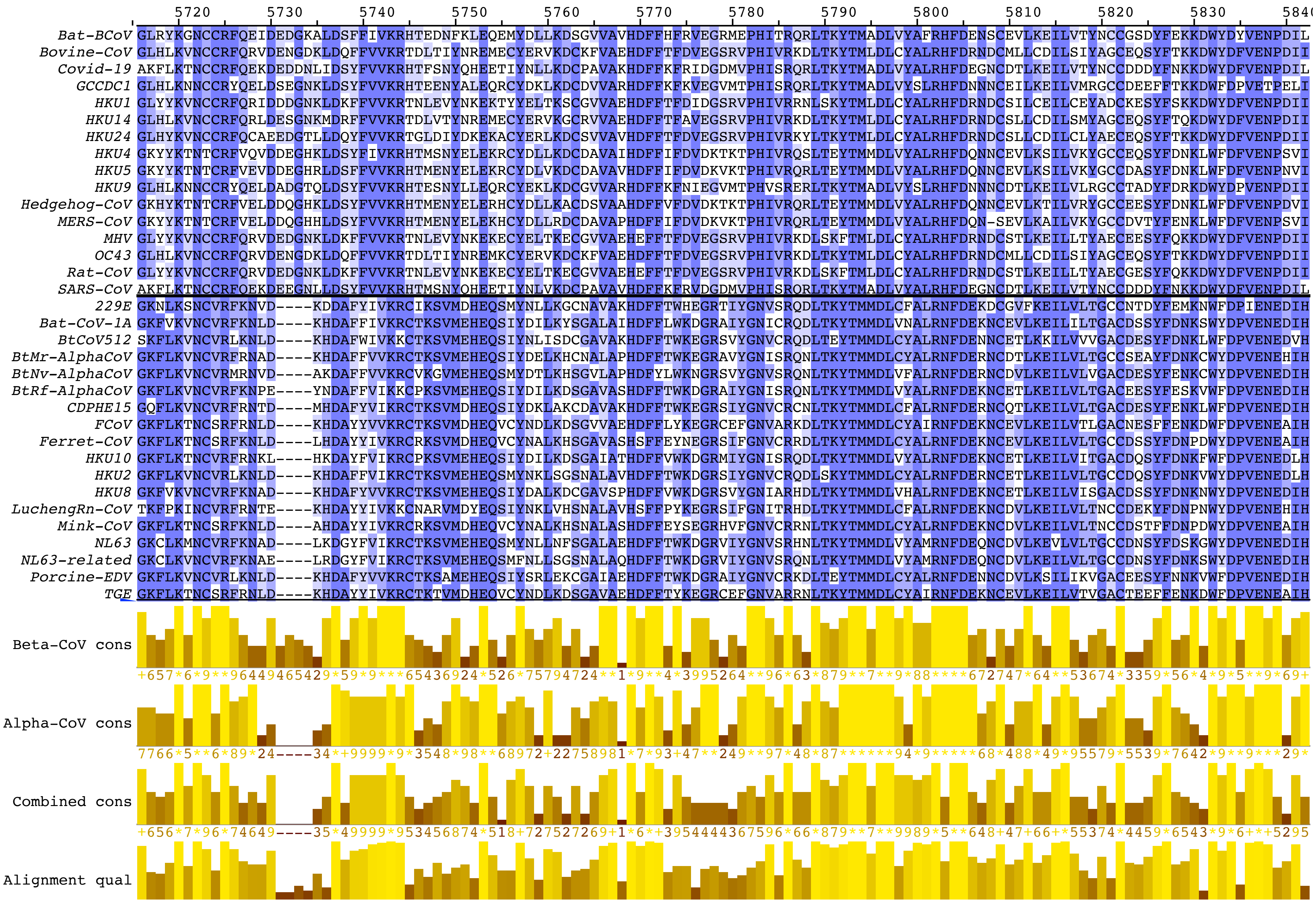

5720
5730
5740
5750
5760
5770
5780
5790
5800
5810
5820
5830
Bat-BCoV
Bovine-CoV
Covid-19
GCCDC1
HKU1
HKU14
HKU24
HKU4
HKU5
HKU9
Hedgehog-CoV
MERS-CoV
MHV
OC43
Rat-CoV
SARS-CoV
229E
Bat-CoV-1A
BtCoV512
BtMr-AlphaCoV
BtNv-AlphaCoV
BtRf-AlphaCoV
CDPHE15
FCoV
Ferret-CoV
HKU10
HKU2
HKU8
LuchengRn-CoV
Mink-CoV
NL63
NL63-related
Porcine-EDV
TGE
Beta-CoV cons
Alpha-CoV cons
Combined cons
Alignment qual

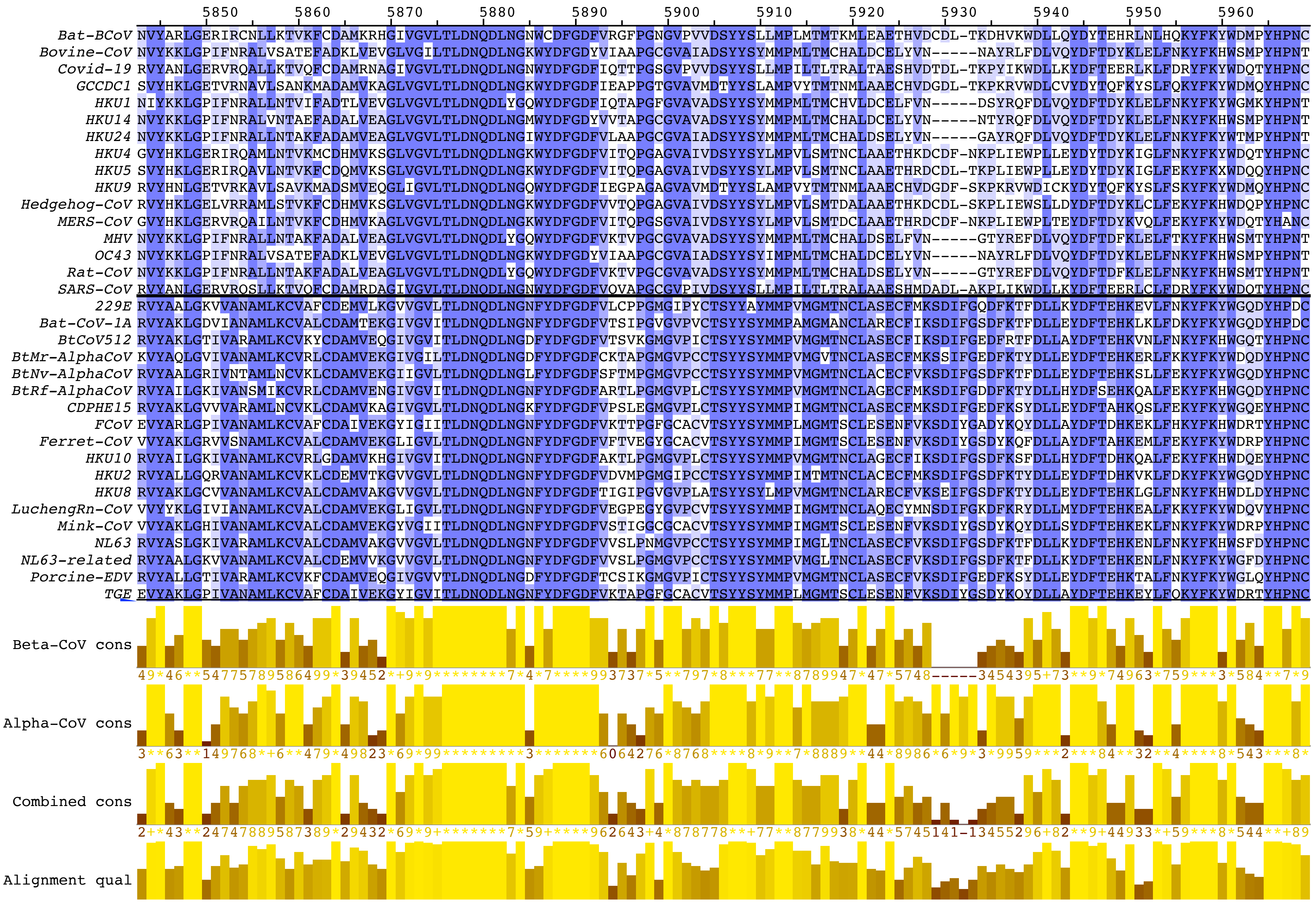
5850 5860 5870 5880 5890 5900 5910 5920 5930 5940 5950 5960
Bat-BCoV NVYARLGERIRCNLLKTVKFCDAMKRHGIVGVLTLDNQDLNGNWCDFGDFVRGFPGNGVPVVDSYYSLLMPLMTMTKMLEAETHVDCDL-TKDHVKWDLLQYDYTEHRLNLHQKYFKYWDMPYHPNC
Bovine-CoV NVYKKLGPIFNRALVSATEFADKLVEVGLVGILTLDNQDLNGKWYDFGDYVIAAPGCGVAIADSYYSYMMPMLTMCHALDCELYVN-----NAYRLFDLVQYDFTDYKLELFNKYFKHWSMPYHPNT
Covid-19 RVYANLGERVRQALLKTVQFCDAMRNAGIVGVLTLDNQDLNGNWYDFGDFIQTTPGSGVPVVDSYYSLLMPILTLTRALTAESHVDTDL-TKPYIKWDLLKYDFTEERLKLFDRYFKYWDQTYHPNC
GCCDC1 SVYHKLGETVRNAVLSANKMADAMVKAGLVGVLTLDNQDLNGKWYDFGDFIEAPPGTGVAVMDTYYSLAMPVYTMTNMLAAECHVDGDL-TKPKRVWDLCVYDYTQFKYSLFQKYFKYWDMQYHPNC
HKU1 NIYKKLGPIFNRALLNTVIFADTLVEVGLVGVLTLDNQDLYGQWYDFGDFIQTAPGFGVAVADSYYSYMMPMLTMCHVLDCELFVN-----DSYRQFDLVQYDFTDYKLELFNKYFKYWGMKYHPNT
HKU14 NVYKKLGPIFNRALVNTAEFADALVEAGLVGVLTLDNQDLNGMWYDFGDYVVTAPGCGVAVADSYYSYMMPMLTMCHALDCELYVN-----NTYRQFDLVQYDFTDYKLELFNKYFKHWSMPYHPNT
HKU24 NVYKKLGPIFNRALLNTAKFADAMVEAGLVGVLTLDNQDLNGIWYDFGDFVLAAPGCGVAIADSYYSYMMPMLTMCHALDSELYVN-----GAYRQFDLVQYDFTDYKLELFNKYFKYWTMPYHPNT
HKU4 GVYHKLGERIRQAMLNTVKMCDHMVKSGLVGVLTLDNQDLNGKWYDFGDFVITQPGAGVAIVDSYYSYLMPVLSMTNCLAAETHKDCDF-NKPLIEWPLLEYDYTDYKIGLFNKYFKYWDQTYHPNC
HKU5 SVYHKLGERIRQAVLNTVKFCDQMVKSGLVGVLTLDNQDLNGKWYDFGDFVITQPGAGVAIVDSYYSYLMPVLSMTNCLAAETHRDCDL-TKPLIEWPLLEYDYTDYKIGLFEKYFKXWDQQYHPNC
HKU9 RVYHNLGETVRKAVLSAVKMADSMVEQGLIGVLTLDNQDLNGQWYDFGDFIEGPAGAGVAVMDTYYSLAMPVYTMTNMLAAECHVDGDF-SKPKRVWDICKYDYTQFKYSLFSKYFKYWDMQYHPNC
Hedgehog-CoV RVYHKLGELVRRAMLSTVKFCDHMVKSGLVGVLTLDNQDLNGKWYDFGDFVVTQPGAGVAIVDSYYSYLMPVLSMTDALAAETHKDCDL-SKPLIEWSLLDYDFTDYKLCLFEKYFKHWDQPYHPNC
MERS-CoV GVYHKLGERVRQAILNTVKFCDHMVKAGLVGVLTLDNQDLNGKWYDFGDFVITQPGSGVAIVDSYYSYLMPVLSMTDCLAAETHRDCDF-NKPLIEWPLTEYDFTDYKVQLFEKYFKYWDQTYHANC
MHV NVYKKLGPIFNRALLNTAKFADALVEAGLVGVLTLDNQDLYGQWYDFGDFVKTVPGCGVAVADSYYSYMMPMLTMCHALDSELFVN-----GTYREFDLVQYDFTDFKLELFTKYFKHWSMTYHPNT
OC43 NVYKKLGPIFNRALVSATEFADKLVEVGLVGVLTLDNQDLNGKWYDFGDYVIAAPGCGVAIADSYYSYIMPMLTMCHALDCELYVN-----NAYRLFDLVQYDFTDYKLELFNKYFKHWSMPYHPNT
Rat-CoV NVYKKLGPIFNRALLNTAKFADALVEAGLVGVLTLDNQDLYGQWYDFGDFVKTVPGCGVAVADSYYSYMMPMLTMCHALDSELYVN-----GTYREFDLVQYDFTDFKLELFNKYFKHWSMTYHPNT
SARS-CoV RVYANLGERVRQSLLKTVQFCDAMRDAGIVGVLTLDNQDLNGNWYDFGDFVQVAPGCGVPIVDSYYSLLMPILTLTRALAAESHMDADL-AKPLIKWDLLKYDFTEERLCLFDRYFKYWDQTYHPNC
229E RVYAALGKVVANAMLKCVAFCDEMVLKGVVGVLTLDNQDLNGNFYDFGDFVLCPPGMGIPYCTSYYAYMMPVMGMTNCLASECFMKSDIFGQDFKTFDLLKYDFTEHKEVLFNKYFKYWGQDYHPDC
Bat-CoV-1A RVYAKLGDVIANAMLKCVALCDAMTEKGIVGVITLDNQDLNGNFYDFGDFVTSIPGVGVPVCTSYYSYMMPAMGMANCLARECFIKSDIFGEDFKTFDLLEYDFTEHKLKLFDKYFKYWGQDYHPDC
BtCoV512 RVYAKLGTIVARAMLKCVKYCDAMVEQGIVGVITLDNQDLNGDFYDFGDFVTSVKGMGVPICTSYYSYMMPVMGMTNCLASECFIKSDIFGEDFRTFDLLAYDFTEHKVNLFNKYFKHWGQTYHPNC
BtMr-AlphaCoV KVYAQLGVIVANAMLKCVRLCDAMVEKGIVGILTLDNQDLNGDFYDFGDFCKTAPGMGVPCCTSYYSYMMPVMGVTNCLASECFMKSSIFGEDFKTYDLLEYDFTEHKERLFKKYFKYWDQDYHPNC
BtNv-AlphaCoV RVYAALGRIVNTAMLNCVKLCDAMVEKGIIGVLTLDNQDLNGLFYDFGDFSFTMPGMGVPCCTSYYSYMMPVMGMTNCLACECFVKSDIFGSDFKTFDLLEYDFTEHKSLLFEKYFKYWGQDYHPNC
BtRf-AlphaCoV RVYAILGKIVANSMIKCVRLCDAMVENGIVGVITLDNQDLNGNFYDFGDFARTLPGMGVPLCTSYYSYMMPVMGMTNCLAGECFMKSDIFGDDFKTYDLLHYDFSEHKQALFEKYFKHWGQDYHPNC
CDPHE15 RVYAKLGVVVARAMLNCVKLCDAMVKAGIVGVLTLDNQDLNGKFYDFGDFVPSLEGMGVPLCTSYYSYMMPIMGMTNCLASECFMKSDIFGEDFKSYDLLEYDFTAHKQSLFEKYFKYWGQEYHPNC
FCoV EVYARLGPIVANAMLKCVAFCDAIVEKGYIGIITLDNQDLNGNFYDFGDFVKTTPGFGCACVTSYYSYMMPLMGMTSCLESENFVKSDIYGADYKQYDLLAYDFTDHKEKLFHKYFKHWDRTYHPNC
Ferret-CoV VVYAKLGRVVSNAMLKCVALCDAMVEKGLIGVLTLDNQDLNGNFYDFGDFVFTVEGYGCACVTSYYSYMMPIMGMTSCLESENFVKSDIYGSDYKQFDLLAYDFTAHKEMLFEKYFKYWDRPYHPNC
HKU10 RVYAILGKIVANAMLKCVRLGDAMVKHGIVGVITLDNQDLNGNFYDFGDFAKTLPGMGVPLCTSYYSYMMPVMGMTNCLAGECFIKSDIFGSDFKSFDLLHYDFTDHKQALFEKYFKHWDQEYHPNC
HKU2 RVYALLGQRVANAMLKCVKLCDEMVTKGVVGVLTLDNQDLNGNFYDFGDFVDVMPGMGIPCCTSYYSYMMPIMTMTNCLACECFMKSDIFGSDFKTYDLLEYDFTDHKVKLFDKYFKYWGQDYHPNC
HKU8 RVYAKLGCVVANAMLKCVALCDAMVAKGVVGVLTLDNQDLNGNFYDFGDFTIGIPGVGVPLATSYYSYLMPVMGMTNCLARECFVKSEIFGSDFKTYDLLEYDFTEHKLGLFNKYFKHWDLDYHPNC
LuchengRn-CoV VVYYKLGIVIANAMLKCVALCDAMVEKGLIGVLTLDNQDLNGNFYDFGDFVEGPEGYGVPCVTSYYSYMMPIMGMTNCLAQECYMNSDIFGKDFKRYDLLMYDFTEHKEALFKKYFKYWDQVYHPNC
Mink-CoV VVYAKLGHIVANAMLKCVALCDAMVEKGYVGIITLDNQDLNGNFYDFGDFVSTIGGCGCACVTSYYSYMMPIMGMTSCLESENFVKSDIYGSDYKQYDLLSYDFTEHKEKLFNKYFKYWDRPYHPNC
NL63 RVYASLGKIVARAMLKCVALCDAMVAKGVVGVLTLDNQDLNGNFYDFGDFVVSLPNMGVPCCTSYYSYMMPIMGLTNCLASECFVKSDIFGSDFKTFDLLKYDFTEHKENLFNKYFKHWSFDYHPNC
NL63-related RVYAALGKVVANAMLKCVALCDEMVVKGVVGVLTLDNQDLNGNFYDFGDFVVSLPGMGVPCCTSYYSYMMPVMGLTNCLASECFVKSDIFGSDFKTFDLLKYDFTEHKENLFNKYFKYWGFDYHPNC
Porcine-EDV RVYALLGTIVARAMLKCVKFCDAMVEQGIVGVVTLDNQDLNGDFYDFGDFTCSIKGMGVPICTSYYSYMMPVMGMTNCLASECFVKSDIFGEDFKSYDLLEYDFTEHKTALFNKYFKYWGLQYHPNC
TGE EVYAKLGPIVANAMLKCVAFCDAIVEKGYIGVITLDNQDLNGNFYDFGDFVKTAPGFGCACVTSYYSYMMPLMGMTSCLESENFVKSDIYGSDYKQYDLLAYDFTEHKEYLFQKYFKYWDRTYHPNC
Beta-CoV cons
49*46**54775789586499*39452*+9*9*******7*4*7****993737*5**797*8***77**8789947*47*5748-----3454395+73**9*74963*759***3*584**7*9
Alpha-CoV cons
3**63**149768*+6**479*49823*69*99********3******6064276*8768****8*9**7*8889**44*8986*6*9*3*9959***2***84**32**4****8*543***8*
Combined cons
2+*43**24747889587389*29432*69*9+*******7*59+****962643+4*878778**+77**8779938*44*5745141-13455296+82**9+44933*+59***8*544**+89
Alignment qual

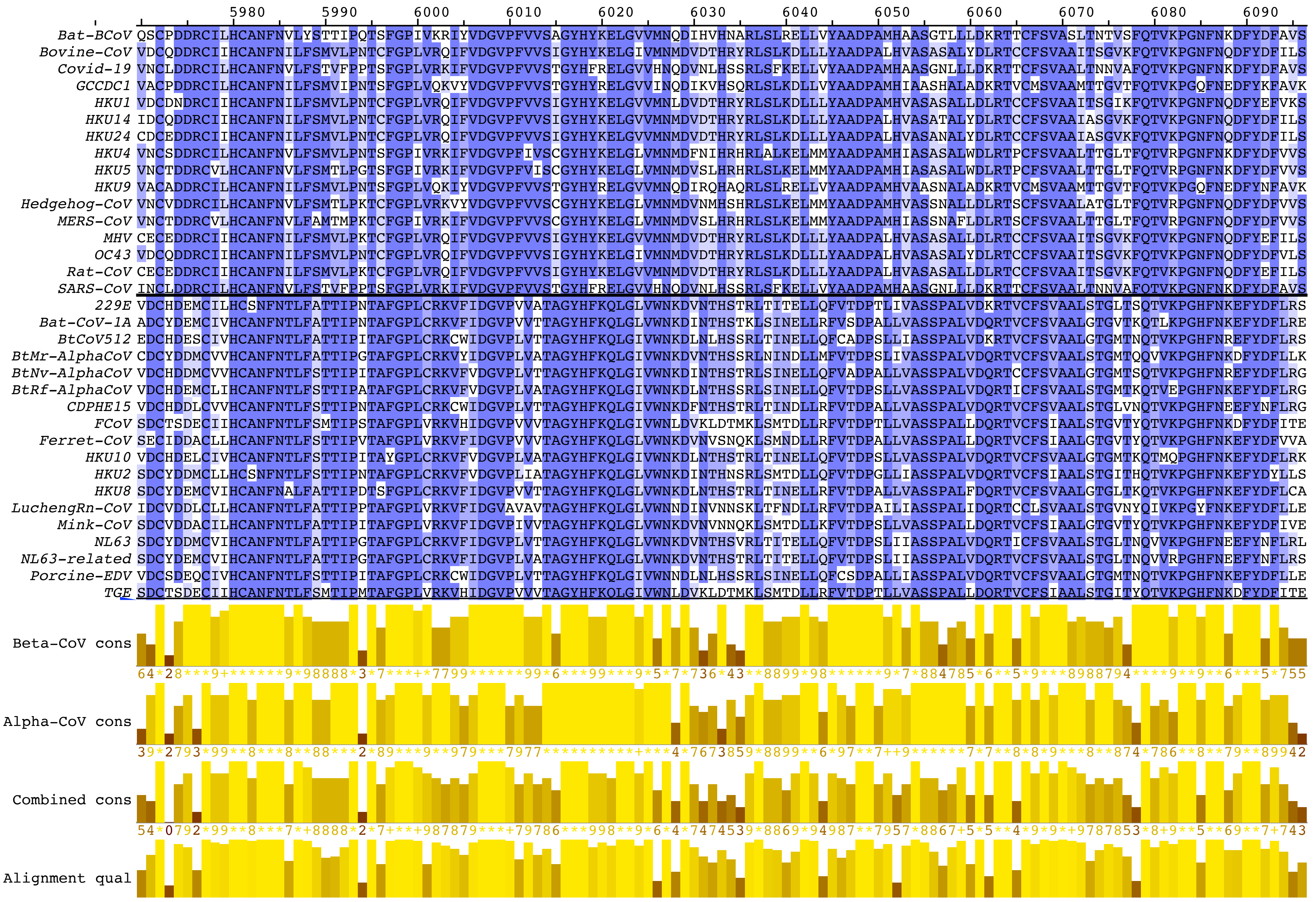
5980 5990 6000 6010 6020 6030 6040 6050 6060 6070 6080 6090
Bat-BCoV QSCPDDRCILHCANFNVLYSTTIPQTSFGPIVKRIYVDGVPFVVSAGYHYKELGVVMNQDIHVHNARLSLRELLVYAADPAMHAASGTLLLDKRTTCFSVASLTNTVSFQTVKPGNFNKDFYDFAVS
Bovine-CoV VDCQDDRCIIHCANFNILFSMVLPNTCFGPLVRQIFVDGVPFVVSIGYHYKELGIVMNMDVDTHRYRLSLKDLLLYAADPALHVASASALYDLRTCCFSVAAITSGVKFQTVKPGNFNQDFYDFILS
Covid-19 VNCLDDRCILHCANFNVLFSTVFPPTSFGPLVRKIFVDGVPFVVSTGYHFRELGVVHNQDVNLHSSRLSFKELLVYAADPAMHAASGNLLLDKRTTCFSVAALTNNVAFQTVKPGNFNKDFYDFAVS
GCCDC1 VACPDDRCILHCANFNILFSMVIPNTSFGPLVQKVYVDGVPFVVSTGYHYRELGVVINQDIKVHSQRLSLKDLLVYAADPAMHIAASHALADKRTVCMSVAAMTTGVTFQTVKPGQFNEDFYKFAVK
HKU1 VDCDNDRCIIHCANFNILFSMVLPNTCFGPLVRQIFVDGVPFVVSIGYHYKELGVVMNLDVDTHRYRLSLKDLLLYAADPAMHVASASALLDLRTCCFSVAAITSGIKFQTVKPGNFNQDFYEFVKS
HKU14 IDCQDDRCIIHCANFNILFSMVLPNTCFGPLVRQIFVDGVPFVVSIGYHYKELGVVMNMDVDTHRYRLSLKDLLLYAADPALHVASATALYDLRTCCFSVAAIASGVKFQTVKPGNFNQDFYDFILS
HKU24 CDCEDDRCIIHCANFNILFSMVLPNTCFGPLVRQIFVDGVPFVVSIGYHYKELGVVMNMDVDTHRYRLSLKDLLLYAADPALHVASANALYDLRTCCFSVAAIASGVKFQTVKPGNFNQDFYDFILS
HKU4 VNCSDDRCILHCANFNVLFSMVLPNTSFGPIVRKIFVDGVPFIVSCGYHYKELGLVMNMDFNIHRHRLALKELMMYAADPAMHIASASALWDLRTPCFSVAALTTGLTFQTVRPGNFNKDFYDFVVS
HKU5 VNCTDDRCVLHCANFNVLFSMTLPGTSFGPIVRKIFVDGVPFVISCGYHYKELGLVMNMDVSLHRHRLSLKELMMYAADPAMHIASASALWDLRTPCFSVAALTTGLTFQTVRPGNFNKDFYDFVVS
HKU9 VACADDRCILHCANFNILFSMVLPNTSFGPLVQKIYVDGVPFVVSTGYHYRELGVVMNQDIRQHAQRLSLRELLVYAADPAMHVAASNALADKRTVCMSVAAMTTGVTFQTVKPGQFNEDFYNFAVK
Hedgehog-CoV VNCVDDRCILHCANFNVLFSMTLPKTCFGPLVRKVYVDGVPFVVSCGYHYKELGLVMNMDVNMHSHRLSLKELMMYAADPAMHVASSNALLDLRTSCFSVAALATGLTFQTVRPGNFNQDFYDFVVS
MERS-CoV VNCTDDRCVLHCANFNVLFAMTMPKTCFGPIVRKIFVDGVPFVVSCGYHYKELGLVMNMDVSLHRHRLSLKELMMYAADPAMHIASSNAFLDLRTSCFSVAALTTGLTFQTVRPGNFNQDFYDFVVS
MHV CECEDDRCIIHCANFNILFSMVLPKTCFGPLVRQIFVDGVPFVVSIGYHYKELGVVMNMDVDTHRYRLSLKDLLLYAADPALHVASASALLDLRTCCFSVAAITSGVKFQTVKPGNFNQDFYEFILS
OC43 VDCQDDRCIIHCANFNILFSMVLPNTCFGPLVRQIFVDGVPFVVSIGYHYKELGIVMNMDVDTHRYRLSLKDLLLYAADPALHVASASALYDLRTCCFSVAAITSGVKFQTVKPGNFNQDFYDFVLS
Rat-CoV CECEDDRCIIHCANFNILFSMVLPKTCFGPLVRQIFVDGVPFVVSIGYHYKELGVVMNMDVDTHRYRLSLKDLLLYAADPALHVASASALLDLRTCCFSVAAITSGVKFQTVKPGNFNQDFYEFILS
SARS-CoV INCLDDRCILHCANFNVLFSTVFPPTSFGPLVRKIFVDGVPFVVSTGYHFRELGVVHNQDVNLHSSRLSFKELLVYAADPAMHAASGNLLLDKRTTCFSVAALTNNVAFQTVKPGNFNKDFYDFAVS
229E VDCHDEMCILHCSNFNTLFATTIPNTAFGPLCRKVFIDGVPVVATAGYHFKQLGLVWNKDVNTHSTRLTITELLQFVTDPTLIVASSPALVDKRTVCFSVAALSTGLTSQTVKPGHFNKEFYDFLRS
Bat-CoV-1A ADCYDEMCIVHCANFNTLFATTIPNTAFGPLCRKVFIDGVPVVTTAGYHFKQLGLVWNKDVNTHSTKLSINELLRFVSDPALLVASSPALVDQRTVCFSVAALGTGVTKQTLKPGHFNKEFYDFLRE
BtCoV512 EDCHDESCIVHCANFNTLFATTIPITAFGPLCRKCWIDGVPLVTTAGYHFKQLGIVWNKDLNLHSSRLTINELLQFCADPSLLIASSPALVDKRTVCFSVAALGTGMTNQTVKPGHFNREFYDFLRS
BtMr-AlphaCoV CDCYDDMCVVHCANFNTLFATTIPGTAFGPLCRKVYIDGVPLVATAGYHFKQLGIVWNKDVNTHSSRLNINDLLMFVTDPSLIVASSPALVDQRTVCFSVAALSTGMTQQVVKPGHFNKDFYDFLLK
BtNv-AlphaCoV VDCHDDMCVVHCANFNTLFSTTIPITAFGPLCRKVFVDGVPLVTTAGYHFKQLGIVWNKDINTHSTRLSINELLQFVADPALLVASSPALVDQRTCCFSVAALGTGMTSQTVKPGHFNREFYDFLRG
BtRf-AlphaCoV VDCHDEMCLIHCANFNTLFSTTIPATAFGPLCRKVFVDGVPLVATAGYHFKQLGIVWNKDLNTHSSRLSINELLQFVTDPSLLVASSPALVDQRTICFSVAALSTGMTKQTVEPGHFNKEFYDFLRG
CDPHE15 VDCHDDLCVVHCANFNTLFSTTIPNTAFGPLCRKCWIDGVPLVTTAGYHFKQLGIVWNKDFNTHSTRLTINDLLRFVTDPALLVASSPALVDQRTVCFSVAALSTGLVNQTVKPGHFNEEFYNFLRG
FCoV SDCTSDECIIHCANFNTLFSMTIPSTAFGPLVRKVHIDGVPVVVTAGYHFKQLGIVWNLDVKLDTMKLSMTDLLRFVTDPTLLVASSPALLDQRTVCFSIAALSTGVTYQTVKPGHFNKDFYDFITE
Ferret-CoV SECIDDACLLHCANFNTLFSTTIPVTAFGPLVRKVFIDGVPVVVTAGYHFKQLGLVWNKDVNVSNQKLSMNDLLRFVTDPALLVASSPALLDQRTVCFSIAALGTGVTYQTVKPGHFNKEFYDFVVA
HKU10 VDCHDELCIVHCANFNTLFSTTIPITAYGPLCRKVFVDGVPLVATAGYHFKQLGIVWNKDLNTHSTRLTINELLQFVTDPSLLVASSPALVDQRTVCFSVAALGTGMTKQTMQPGHFNKEFYDFLRK
HKU2 SDCYDDMCLLHCSNFNTLFSTTIPNTAFGPLCRKVFVDGVPLIATAGYHFKQLGLVWNKDINTHNSRLSMTDLLQFVTDPGLLIASSPALVDQRTVCFSIAALSTGITHQTVKPGHFNKEFYDYLLS
HKU8 SDCYDEMCVIHCANFNALFATTIPDTSFGPLCRKVFIDGVPVVTTAGYHFKQLGLVWNKDLNTHSTRLTINELLRFVTDPALLVASSPALFDQRTVCFSVAALGTGLTKQTVKPGHFNKEFYDFLCA
LuchengRn-CoV IDCVDDLCLLHCANFNTLFATTIPPTAFGPLVRKVFIDGVAVAVTAGYHFKQLGLVWNNDINVNNSKLTFNDLLRFVTDPAILIASSPALIDQRTCCLSVAALSTGVNYQIVKPGYFNKEFYDFLLE
Mink-CoV SDCVDDACILHCANFNTLFATTIPITAFGPLVRKVFIDGVPIVVTAGYHFKQLGLVWNKDVNVNNQKLSMTDLLKFVTDPSLLVASSPALLDQRTVCFSIAALGTGVTYQTVKPGHFNKEFYDFIVE
NL63 SDCYDDMCVIHCANFNTLFATTIPGTAFGPLCRKVFIDGVPLVTTAGYHFKQLGLVWNKDVNTHSVRLTITELLQFVTDPSLIIASSPALVDQRTICFSVAALSTGLTNQVVKPGHFNEEFYNFLRL
NL63-related SDCYDEMCVIHCANFNTLFATTIPGTAFGPLCRKVFIDGVPLVATAGYHFKQLGLVWNKDVNTHSTRLTITELLQFVTDPSLIIASSPALVDQRTVCFSVAALSTGLTNQVVRPGHFNEEFYNFLRS
Porcine-EDV VDCSDEQCIVHCANFNTLFSTTIPITAFGPLCRKCWIDGVPLVTTAGYHFKQLGIVWNNDLNLHSSRLSINELLQFCSDPALLIASSPALVDQRTVCFSVAALGTGMTNQTVKPGHFNKEFYDFLLE
TGE SDCTSDECIIHCANFNTLFSMTIPMTAFGPLVRKVHIDGVPVVVTAGYHFKQLGIVWNLDVKLDTMKLSMTDLLRFVTDPTLLVASSPALLDQRTVCFSIAALSTGITYQTVKPGHFNKDFYDFITE
Beta-CoV cons
64*28***9+******9*98888*3*7***+*7799*****99*6***99***9*5*7*736*43**8899*98******9*7*884785*6**5*9***898794****9**9**6***5*755
Alpha-CoV cons
39*2793*99**8***8**88***2*89***9**979***7977**********+***4*7673859*8899**6*97**7++9******7*7**8*8*9***8**874*786**8**79**89942
Combined cons
54*0792*99**8***7*+8888*2*7+**+987879***+79786***998**9*6*4*7474539*8869*94987**7957*8867+5*5**4*9*9*+9787853*8+9**5**69**7+743
Alignment qual

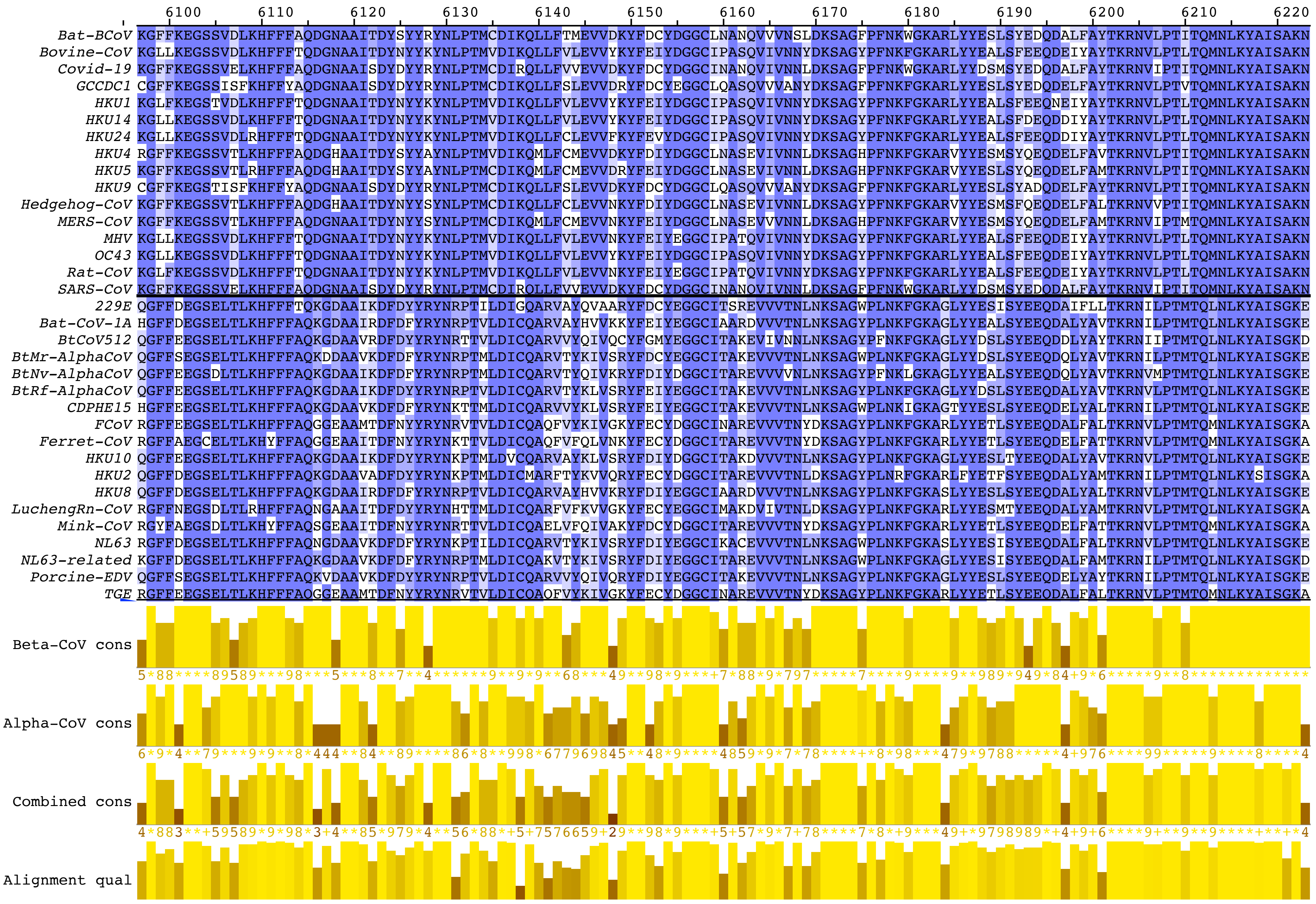
6100 6110 6120 6130 6140 6150 6160 6170 6180 6190 6200 6210 6220
Bat-BCoV
Bovine-CoV
Covid-19
GCCDC1
HKU1
HKU14
HKU24
HKU4
HKU5
HKU9
Hedgehog-CoV
MERS-CoV
MHV
OC43
Rat-CoV
SARS-CoV
229E
Bat-CoV-1A
BtCoV512
BtMr-AlphaCoV
BtNv-AlphaCoV
BtRf-AlphaCoV
CDPHE15
FCoV
Ferret-CoV
HKU10
HKU2
HKU8
LuchengRn-CoV
Mink-CoV
NL63
NL63-related
Porcine-EDV
TGE
Beta-CoV cons
Alpha-CoV cons
Combined cons
Alignment qual

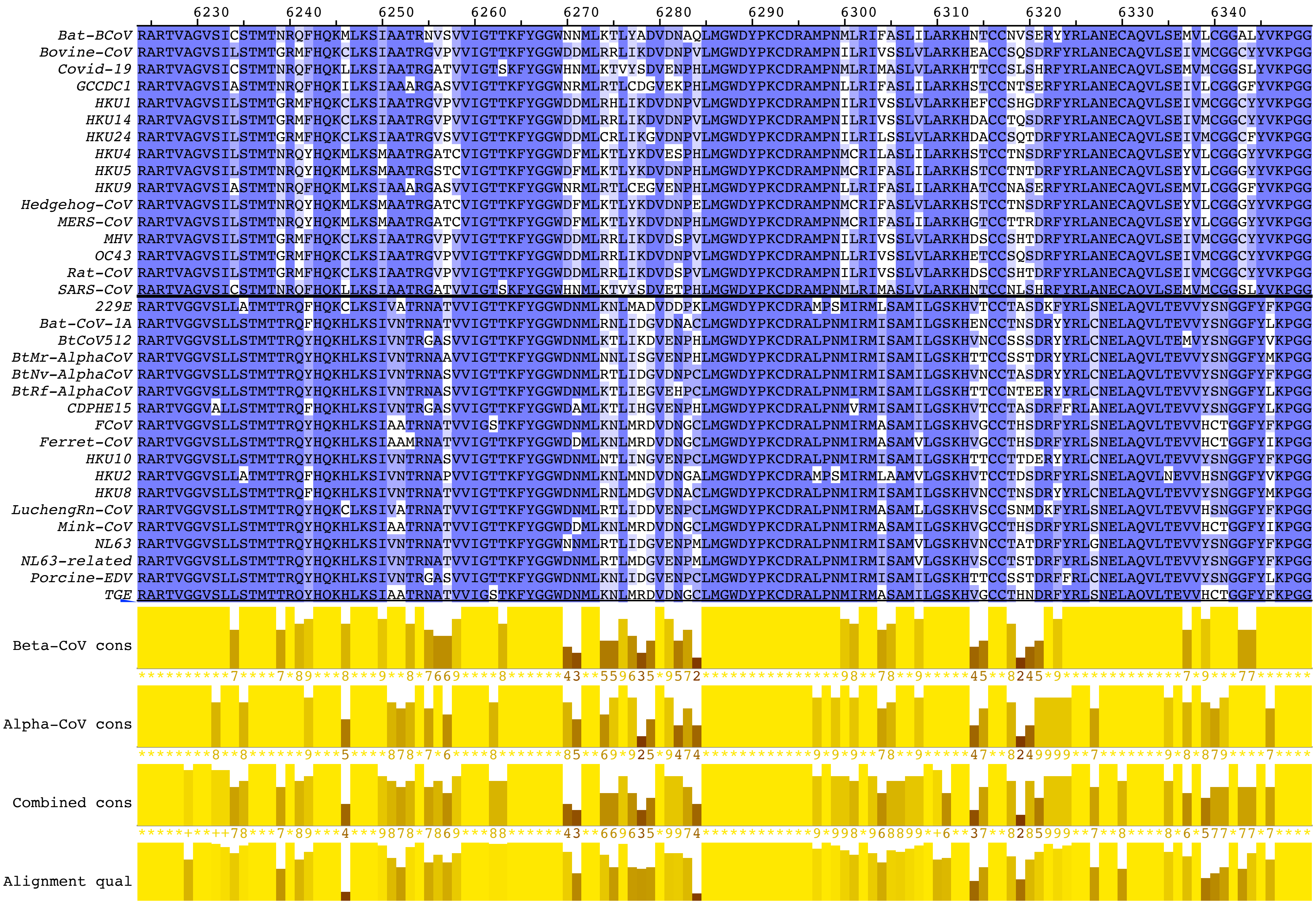
6230 6240 6250 6260 6270 6280 6290 6300 6310 6320 6330 6340
Bat-BCoV RARTVAGVSICSTMTNRQFHQKMLKSIAATRNVSVVIGTTKFYGGWNNMLKTLYADVDNAQLMGWDYPKCDRAMPNMLRIFASLILARKHNTCCNVSERYYRLANECAQVLSEMVLCGGALYVVKPGG
Bovine-CoV RARTVAGVSILSTMTGRMFHQKCLKSIAATRGVPVVIGTTKFYGGWDDMLRRLIKDVDNPVLMGWDYPKCDRAMPNILRIVSSLVLARKHEACCSQSDRFYRLANECAQVLSEIVMCGGCYYVKPGG
Covid-19 RARTVAGVSICSTMTNRQFHQKLLKSIAATRGATVVIGTSKFYGGWHNMLKTVYSDVENPHLMGWDYPKCDRAMPNMLRIMASLVLARKHTTCCSLSHRFYRLANECAQVLSEMVMCGGSLYVVKPGG
GCCDC1 RARTVAGVSIASTMTNRQFHQKILKSIAAARGASVVIGTTKFYGGWNRMLRTLCDGVEKPHLMGWDYPKCDRAMPNLLRIFASLILARKHSTCCNTSERFYRLANECAQVLSEIVLCGGGFYVKPGG
HKU1 RARTVAGVSILSTMTGRMFHQKCLKSIAATRGVPVVIGTTKFYGGWDDMLRHLIKDVDNPVLMGWDYPKCDRAMPNILRIVSSLVLARKHEFCCSHGDRFYRLANECAQVLSEIVMCGGCYYVKPGG
HKU14 RARTVAGVSILSTMTGRMFHQKCLKSIAATRGVPVVIGTTKFYGGWDDMLRRLIKDVDNPVLMGWDYPKCDRAMPNILRIVSSLVLARKHDACCTQSDRFYRLANECAQVLSEIVMCGGCYYVKPGG
HKU24 RARTVAGVSILSTMTGRMFHQKCLKSIAATRGVSVVIGTTKFYGGWDDMLCRLIKGVDNPVLMGWDYPKCDRAMPNILRILSSLVLARKHDACCSQTDRFYRLANECAQVLSEIVMCGGCFYVKPGG
HKU4 RARTVAGVSILSTMTNRQYHQKMLKSMAATRGATCVIGTTKFYGGWDFMLKTLYKDVESPHLMGWDYPKCDRAMPNMCRILASLILARKHSTCCTNSDRFYRLANECAQVLSEYVLCGGGYYVKPGG
HKU5 RARTVAGVSILSTMTNRQYHQKMLKSMAATRGSTCVIGTTKFYGGWDFMLKTLYKDVDNPHLMGWDYPKCDRAMPNMCRIFASLILARKHSTCCTNTDRFYRLANECAQVLSEYVLCGGGYYVKPGG
HKU9 RARTVAGVSIASTMTNRQFHQKMLKSIAAARGASVVIGTTKFYGGWNRMLRTLCEGVENPHLMGWDYPKCDRAMPNLLRIFASLILARKHATCCNASERFYRLANECAQVLSEMVLCGGGFYVKPGG
Hedgehog-CoV RARTVAGVSILSTMTNRQYHQKMLKSMAATRGATCVIGTTKFYGGWDFMLKTLYKDVDNPELMGWDYPKCDRAMPNMCRIFASLVLARKHSTCCTNSDRFYRLANECAQVLSEYVLCGGGYYVKPGG
MERS-CoV RARTVAGVSILSTMTNRQYHQKMLKSMAATRGATCVIGTTKFYGGWDFMLKTLYKDVDNPHLMGWDYPKCDRAMPNMCRIFASLILARKHGTCCTTRDRFYRLANECAQVLSEYVLCGGGYYVKPGG
MHV RARTVAGVSILSTMTGRMFHQKCLKSIAATRGVPVVIGTTKFYGGWDDMLRRLIKDVDSPVLMGWDYPKCDRAMPNILRIVSSLVLARKHDSCCSHTDRFYRLANECAQVLSEIVMCGGCYYVKPGG
OC43 RARTVAGVSILSTMTGRMFHQKCLKSIAATRGVPVVIGTTKFYGGWDDMLRRLIKDVDNPVLMGWDYPKCDRAMPNLLRIVSSLVLARKHETCCSQSDRFYRLANECAQVLSEIVMCGGCYYVKPGG
Rat-CoV RARTVAGVSILSTMTGRMFHQKCLKSIAATRGVPVVIGTTKFYGGWDDMLRRLIKDVDSPVLMGWDYPKCDRAMPNILRIVSSLVLARKHDSCCSHTDRFYRLANECAQVLSEIVMCGGCYYVKPGG
SARS-CoV RARTVAGVSICSTMTNRQFHQKLLKSIAATRGATVVIGTSKFYGGWHNMLKTVYSDVETPHLMGWDYPKCDRAMPNMLRIMASLVLARKHNTCCNLSHRFYRLANECAQVLSEMVMCGGSLYVVKPGG
229E RARTVGGVSLLATMTTRQFHQKCLKSIVATRNATVVIGTTKFYGGWDNMLKNLMADVDDPKLMGWDYPKCDRAMPSMIRMLSAMILGSKHVTCCTASDKFYRLSNELAQVLTEVVYSNGGFYFKPGG
Bat-CoV-1A RARTVGGVSLLSTMTTRQFHQKHLKSIVNTRNATVVIGTTKFYGGWDNMLRNLIDGVDNACLMGWDYPKCDRALPNMIRMISAMILGSKHENCCTNSDRYYRLCNELAQVLTEVVYSNGGFYVKPGG
BtCoV512 RARTVGGVSLLSTMTTRQYHQKHLKSIVNTRGASVVIGTTKFYGGWDNMLKTLIKDVENPHLMGWDYPKCDRALPNMIRMISAMILGSKHVNCCSSSDRYYRLCNELAQVLTEMVYSNGGFYVKPGG
BtMr-AlphaCoV RARTVGGVSLLSTMTTRQYHQKHLKSIVNTRNAAVVIGTTKFYGGWDNMLNNLISGVENPHLMGWDYPKCDRALPNMIRMISAMILGSKHTTCCSSTDRYYRLCNELAQVLTEVVYSNGGFYMKPGG
BtNv-AlphaCoV RARTVGGVSLLSTMTTRQYHQKHLKSIVNTRNASVVIGTTKFYGGWDNMLRTLIDGVDNPCLMGWDYPKCDRALPNMIRMISAMILGSKHVNCCTASDRYYRLCNELAQVLTEVVYSNGGFYFKPGG
BtRf-AlphaCoV RARTVGGVSLLSTMTTRQYHQKHLKSIVNTRNASVVIGTTKFYGGWDNMLKTLIEGVEHPCLMGWDYPKCDRALPNMIRMISAMILGSKHTTCCNTEERYYRLCNELAQVLTEVVYSNGGFYLKPGG
CDPHE15 RARTVGGVALLSTMTTRQFHQKHLKSIVNTRGASVVIGTTKFYGGWDAMLKTLIHGVENPHLMGWDYPKCDRALPNMVRMISAMILGSKHVTCCTASDRFFRLANELAQVLTEVVYSNGGFYLKPGG
FCoV RARTVGGVSLLSTMTTRQYHQKHLKSIAATRNATVVIGSTKFYGGWDNMLKNLMRDVDNGCLMGWDYPKCDRALPNMIRMASAMILGSKHVGCCTHSDRFYRLSNELAQVLTEVVHCTGGFYFKPGG
Ferret-CoV RARTVGGVSLLSTMTTRQYHQKHLKSIAAMRNATVVIGTTKFYGGWDDMLKNLMRDVDNGCLMGWDYPKCDRALPNMIRMASAMVLGSKHVGCCTHSDRFYRLSNELAQVLTEVVHCTGGFYIKPGG
HKU10 RARTVGGVSLLSTMTTRQYHQKHLKSIVNTRNASVVIGTTKFYGGWDNMLNTLINGVENPCLMGWDYPKCDRALPNMIRMISAMILGSKHTTCCTTDERYYRLCNELAQVLTEVVYSNGGFYFKPGG
HKU2 RARTVGGVSLLATMTTRQFHQKHLKSIVNTRNAPVVIGTTKFYGGWDNMLKNLMNDVDNGALMGWDYPKCDRAMPSMIRMLAAMVLGSKHVTCCTDSDRFYRLSNELAQVLNEVVHSNGGFYVKPGG
HKU8 RARTVGGVSLLSTMTTRQFHQKHLKSIVNTRNATVVIGTTKFYGGWDNMLRNLMDGVDNACLMGWDYPKCDRALPNMIRMISAMILGSKHVNCCTNSDRYYRLCNELAQVLTEVVYSNGGFYMKPGG
LuchengRn-CoV RARTVGGVSLLSTMTTRQYHQKCLKSIVATRNATVVIGTTKFYGGWDNMLRTLIDDVENPCLMGWDYPKCDRALPNMIRMASAMLLGSKHVSCCSNMDKFYRLSNELAQVLTEVVHSNGGFYFKPGG
Mink-CoV RARTVGGVSLLSTMTTRQYHQKHLKSIAATRNATVVIGTTKFYGGWDDMLKNLMRDVDNGCLMGWDYPKCDRALPNMIRMASAMILGSKHVGCCTHSDRFYRLSNELAQVLTEVVHCTGGFYIKPGG
NL63 RARTVGGVSLLSTMTTRQYHQKHLKSIVNTRNATVVIGTTKFYGGWNNMLRTLIDGVENPMLMGWDYPKCDRALPNMIRMISAMVLGSKHVNCCTATDRFYRLGNELAQVLTEVVYSNGGFYFKPGG
NL63-related RARTVGGVSLLSTMTTRQYHQKHLKSIVNTRNATVVIGTTKFYGGWDNMLRTLMDGVENPMLMGWDYPKCDRALPNMIRMISAMVLGSKHVSCCTSTDRFYRLSNELAQVLTEVVYSNGGFYFKPGG
Porcine-EDV RARTVGGVSLLSTMTTRQYHQKHLKSIVNTRGASVVIGTTKFYGGWDNMLKNLIDGVENPCLMGWDYPKCDRALPNMIRMISAMILGSKHTTCCSSTDRFFRLCNELAQVLTEVVYSNGGFYLKPGG
TGE RARTVGGVSLLSTMTTRQYHQKHLKSIAATRNATVVIGSTKFYGGWDNMLKNLMRDVDNGCLMGWDYPKCDRALPNMIRMASAMILGSKHVGCCTHNDRFYRLSNELAQVLTEVVHCTGGFYFKPGG
Beta-CoV cons
*********7****7*89***8***9**8*7669****8******43**559635*9572**************98**78**9*****45**8245*9*************7*9***77******
Alpha-CoV cons
********8**8******9***5****878*7*6****8*******85**69*925*9474************9*9*9**78**9*****47**824999**7*******9*8*879****7****
Combined cons
*****+**++78***7*89***4***9878*7869***88******43**669635*9974************9*998*968899*+6**37**8285999**7**8****8*6*577*77*7****
Alignment qual

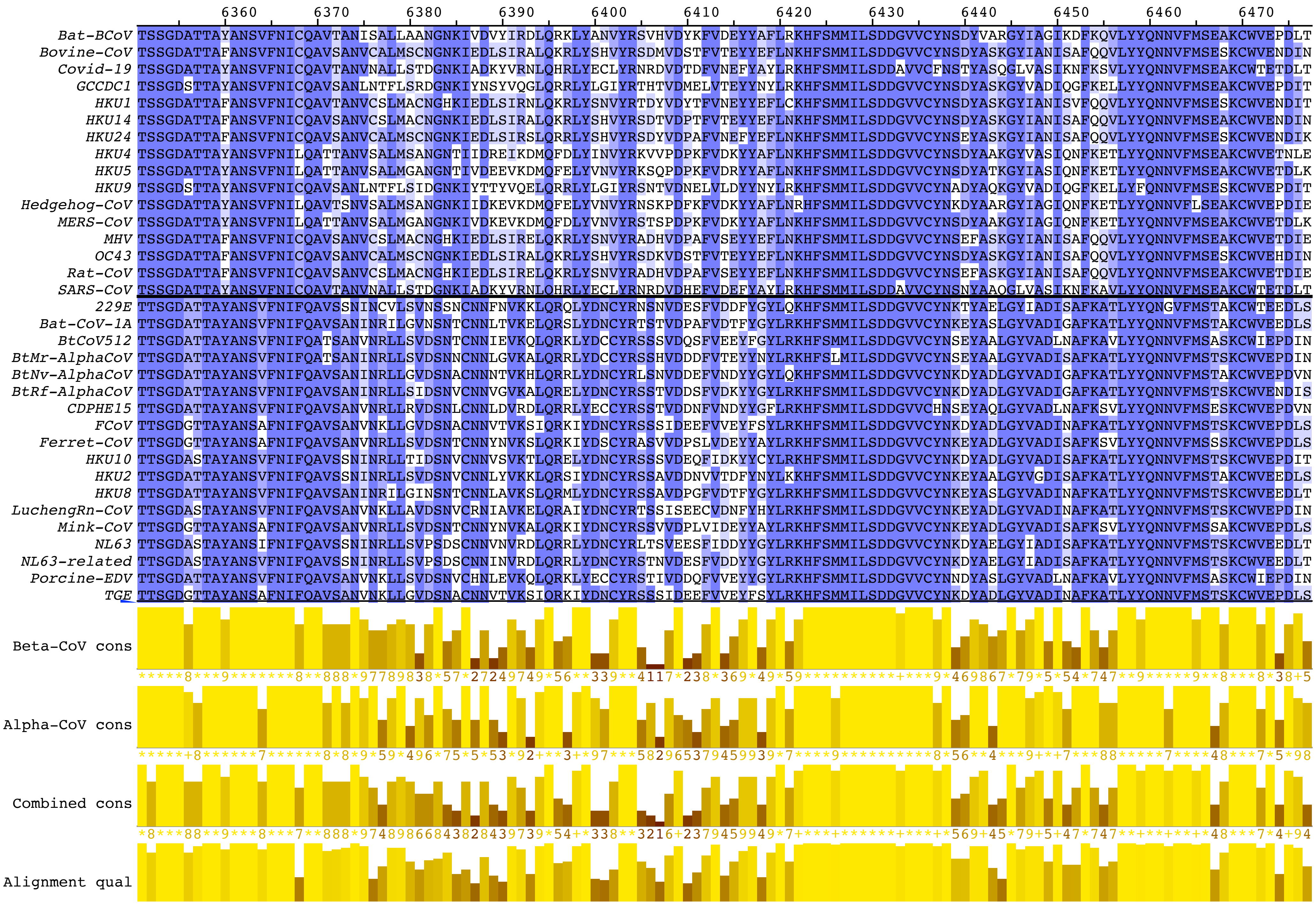
6360 6370 6380 6390 6400 6410 6420 6430 6440 6450 6460 6470
Bat-BCoV TSSGDATTAYANSVFNICQAVTANISALLAANGNKIVDVYIRDLQRKLYANVYRSVHVDYKFVDEYYAFLRKHFSMMILSDDGVVCYNSDYVARGYIAGIKDFKQVLYYQNNVFMSEAKCWVEPDLT
Bovine-CoV TSSGDATTAFANSVFNICQAVSANVCALMSCNGNKIEDLSIRALQKRLYSHVYRSDMVDSTFVTEYYEFLNKHFSMMILSDDGVVCYNSDYASKGYIANISAFQQVLYYQNNVFMSESKCWVENDIN
Covid-19 TSSGDATTAYANSVFNICQAVTANVNALLSTDGNKIADKYVRNLQHRLYECLYRNRDVDTDFVNEFYAYLRKHFSMMILSDDAVVCFNSTYASQGLVASIKNFKSVLYYQNNVFMSEAKCWTETDLT
GCCDC1 TSSGDSTTAYANSVFNICQAVSANLNTFLSRDGNKIYNSYVQGLQRRLYLGIYRTHTVDMELVTEYYNYLRKHFSMMILSDDGVVCYNSDYASKGYVADIQGFKELLYYQNNVFMSEAKCWVEPDIT
HKU1 TSSGDATTAFANSVFNICQAVTANVCSLMACNGHKIEDLSIRNLQKRLYSNVYRTDYVDYTFVNEYYEFLCKHFSMMILSDDGVVCYNSDYASKGYIANISVFQQVLYYQNNVFMSESKCWVENDIT
HKU14 TSSGDATTAFANSVFNICQAVSANVCSLMACNGNKIEDLSIRALQKRLYSHVYRSDTVDPTFVTEYYEFLNKHFSMMILSDDGVVCYNSDYASKGYIANISAFQQVLYYQNNVFMSEAKCWVENDIN
HKU24 TSSGDATTAFANSVFNICQAVSANVCALMSCNGNKIEDLSIRSLQRRLYSHVYRSDYVDPAFVNEFYEFLNKHFSMMILSDDGVVCYNSEYASKGYIANISAFQQVLYYQNNVFMSESKCWVENDIK
HKU4 TSSGDATTAYANSVFNILQATTANVSALMSANGNTIIDREIKDMQFDLYINVYRKVVPDPKFVDKYYAFLNKHFSMMILSDDGVVCYNSDYAAKGYVASIQNFKETLYYQNNVFMSEAKCWVETNLE
HKU5 TSSGDATTAYANSVFNILQATTANVSALMGANGNTIVDEEVKDMQFELYVNVYRKSQPDPKFVDRYYAFLNKHFSMMILSDDGVVCYNSDYATKGYIASIQNFKETLYYQNNVFMSEAKCWVETDLK
HKU9 TSSGDSTTAYANSVFNICQAVSANLNTFLSIDGNKIYTTYVQELQRRLYLGIYRSNTVDNELVLDYYNYLRKHFSMMILSDDGVVCYNADYAQKGYVADIQGFKELLYFQNNVFMSESKCWVEPDIT
Hedgehog-CoV TSSGDATTAYANSVFNILQAVTSNVSALMSANGNKIIDKEVKDMQFELYVNVYRNSKPDFKFVDKYYAFLNRHFSMMILSDDGVVCYNKDYAARGYIAGIQNFKETLYYQNNVFLSEAKCWVEPDIE
MERS-CoV TSSGDATTAYANSVFNILQATTANVSALMGANGNKIVDKEVKDMQFDLYVNVYRSTSPDPKFVDKYYAFLNKHFSMMILSDDGVVCYNSDYAAKGYIAGIQNFKETLYYQNNVFMSEAKCWVETDLK
MHV TSSGDATTAFANSVFNICQAVSANVCSLMACNGHKIEDLSIRELQKRLYSNVYRADHVDPAFVSEYYEFLNKHFSMMILSDDGVVCYNSEFASKGYIANISAFQQVLYYQNNVFMSEAKCWVETDIE
OC43 TSSGDATTAFANSVFNICQAVSANVCALMSCNGNKIEDLSIRALQKRLYSHVYRSDKVDSTFVTEYYEFLNKHFSMMILSDDGVVCYNSDYASKGYIANISAFQQVLYYQNNVFMSESKCWVEHDIN
Rat-CoV TSSGDATTAFANSVFNICQAVSANVCSLMACNGHKIEDLSIRELQKRLYSNVYRADHVDPAFVSEYYEFLNKHFSMMILSDDGVVCYNSEFASKGYIANISAFQQVLYYQNNVFMSEAKCWVETDIE
SARS-CoV TSSGDATTAYANSVFNICQAVTANVNALLSTDGNKIADKYVRNLQHRLYECLYRNRDVDHEFVDEFYAYLRKHFSMMILSDDAVVCYNSNYAAQGLVASIKNFKAVLYYQNNVFMSEAKCWTETDLT
229E TTSGDATTAYANSVFNIFQAVSSNINCVLSVNSSNCNNFNVKKLQRQLYDNCYRNSNVDESFVDDFYGYLQKHFSMMILSDDGVVCYNKTYAELGYIADISAFKATLYYQNGVFMSTAKCWTEEDLS
Bat-CoV-1A TTSGDATTAYANSVFNIFQAVSANINRILGVNSNTCNNLTVKELQRSLYDNCYRTSTVDPAFVDTFYGYLRKHFSMMILSDDGVVCYNKEYASLGYVADISAFKATLYYQNNVFMSTAKCWVEEDLS
BtCoV512 TTSGDATTAYANSVFNIFQATSANVNRLLSVDSNTCNNIEVKQLQRKLYDCCYRSSSVDQSFVEEYFGYLRKHFSMMILSDDGVVCYNSEYAALGYVADLNAFKAVLYYQNNVFMSASKCWIEPDIN
BtMr-AlphaCoV TTSGDATTAYANSVFNIFQATSANINRLLSVDSNNCNNLGVKALQRRLYDCCYRSSHVDDDFVTEYYNYLRKHFSLMILSDDGVVCYNSEYAALGYVADISAFKATLYYQNNVFMSTSKCWVEPDIN
BtNv-AlphaCoV TTSGDATTAYANSVFNIFQAVSANINRLLGVDSNACNNNTVKHLQRRLYDNCYRLSNVDDEFVNDYYGYLQKHFSMMILSDDGVVCYNKDYADLGYVADIGAFKATLYYQNNVFMSTAKCWVEPDVN
BtRf-AlphaCoV TTSGDATTAYANSVFNIFQAVSANINRLLSIDSNVCNNVGVKALQRELYDNCYRSSTVDDSFVDKYYGYLRKHFSMMILSDDGVVCYNKDYADLGYVADIGAFKATLYYQNNVFMSTSKCWVENDIS
CDPHE15 TTSGDATTAYANSVFNIFQAVSANVNRLLRVDSNLCNNLDVRDLQRRLYECCYRSSTVDDNFVNDYYGFLRKHFSMMILSDDGVVCHNSEYAQLGYVADLNAFKSVLYYQNNVFMSESKCWVEPDVN
FCoV TTSGDGTTAYANSAFNIFQAVSANVNKLLGVDSNACNNVTVKSIQRKIYDNCYRSSSIDEEFVVEYFSYLRKHFSMMILSDDGVVCYNKDYADLGYVADINAFKATLYYQNNVFMSTSKCWVEPDLS
Ferret-CoV TTSGDGTTAYANSAFNIFQAVSANVNRLLSVDSNTCNNYNVKSLQRKIYDSCYRASVVDPSLVDEYYAYLRKHFSMMILSDDGVVCYNKEYADLGYVADISAFKSVLYYQNNVFMSSSKCWVEPDLS
HKU10 TTSGDASTAYANSVFNIFQAVSSNINRLLTIDSNVCNNVSVKTLQRELYDNCYRSSSVDEQFIDKYYCYLRKHFSMMILSDDGVVCYNKDYADLGYVADISAFKATLYYQNNVFMSTSKCWVEPDIT
HKU2 TTSGDATTAYANSVFNIFQAVSSNINRLLSVDSNVCNNLYVKKLQRSIYDNCYRSSAVDDNVVTDFYNYLKKHFSMMILSDDGVVCYNKEYAALGYVGDISAFKATLYYQNNVFMSTAKCWVEEDLS
HKU8 TTSGDATTAYANSVFNIFQAVSANINRILGINSNTCNNLAVKSLQRMLYDNCYRSSAVDPGFVDTFYGYLRKHFSMMILSDDGVVCYNKEYASLGYVADINAFKATLYYQNNVFMSTSKCWVEEDLT
LuchengRn-CoV TTSGDASTAYANSVFNIFQAVSANVNKLLAVDSNVCRNIAVKELQRAIYDNCYRTSSISEECVDNFYHYLRKHFSMMILSDDGVVCYNKEYADLGYVADINAFKATLYYQNNVFMSTSKCWVEPDIN
Mink-CoV TTSGDGTTAYANSAFNIFQAVSANVNRLLSVDSNTCNNYNVKALQRKIYDNCYRSSVVDPLVIDEYYAYLRKHFSMMILSDDGVVCYNKEYADLGYVADISAFKSVLYYQNNVFMSSAKCWVEPDLS
NL63 TTSGDASTAYANSIFNIFQAVSSNINRLLSVPSDSCNNVNVRDLQRRLYDNCYRLTSVEESFIDDYYGYLRKHFSMMILSDDGVVCYNKDYAELGYIADISAFKATLYYQNNVFMSTSKCWVEEDLT
NL63-related TTSGDASTAYANSVFNIFQAVSSNINRLLSVPSDSCNNINVRDLQRRLYDNCYRSTNVDESFVDDYYGYLRKHFSMMILSDDGVVCYNKDYAELGYIADISAFKATLYYQNNVFMSTSKCWVEEDLT
Porcine-EDV TTSGDATTAYANSVFNIFQAVSANVNKLLSVDSNVCHNLEVKQLQRKLYECCYRSTIVDDQFVVEYYGYLRKHFSMMILSDDGVVCYNNDYASLGYVADLNAFKAVLYYQNNVFMSASKCWIEPDIN
TGE TTSGDGTTAYANSAFNIFQAVSANVNKLLGVDSNACNNVTVKSIQRKIYDNCYRSSSIDEEFVVEYFSYLRKHFSMMILSDDGVVCYNKDYADLGYVADINAFKATLYYQNNVFMSTSKCWVEPDLS
Beta-CoV cons
*****8***9******8**888*97789838*57*2724974 9*56**339**4117*238*369*49*59*********+****9*469867*79*5*54*747**9*****9**8***8*38+5
Alpha-CoV cons
*****+8******7******8*8*9*59*496*75*5*53*92+**3+*97***582965379459939*7****9*********8*56**4***9+*+7***88*****7****48***7*5*98
Combined cons
*8***88**9***8***7**888*97489866843828439739*54+*338**3216+2379459949*7+***+******+****+*569+45*79+5+47*747**+***+**+*48***7*4+94
Alignment qual

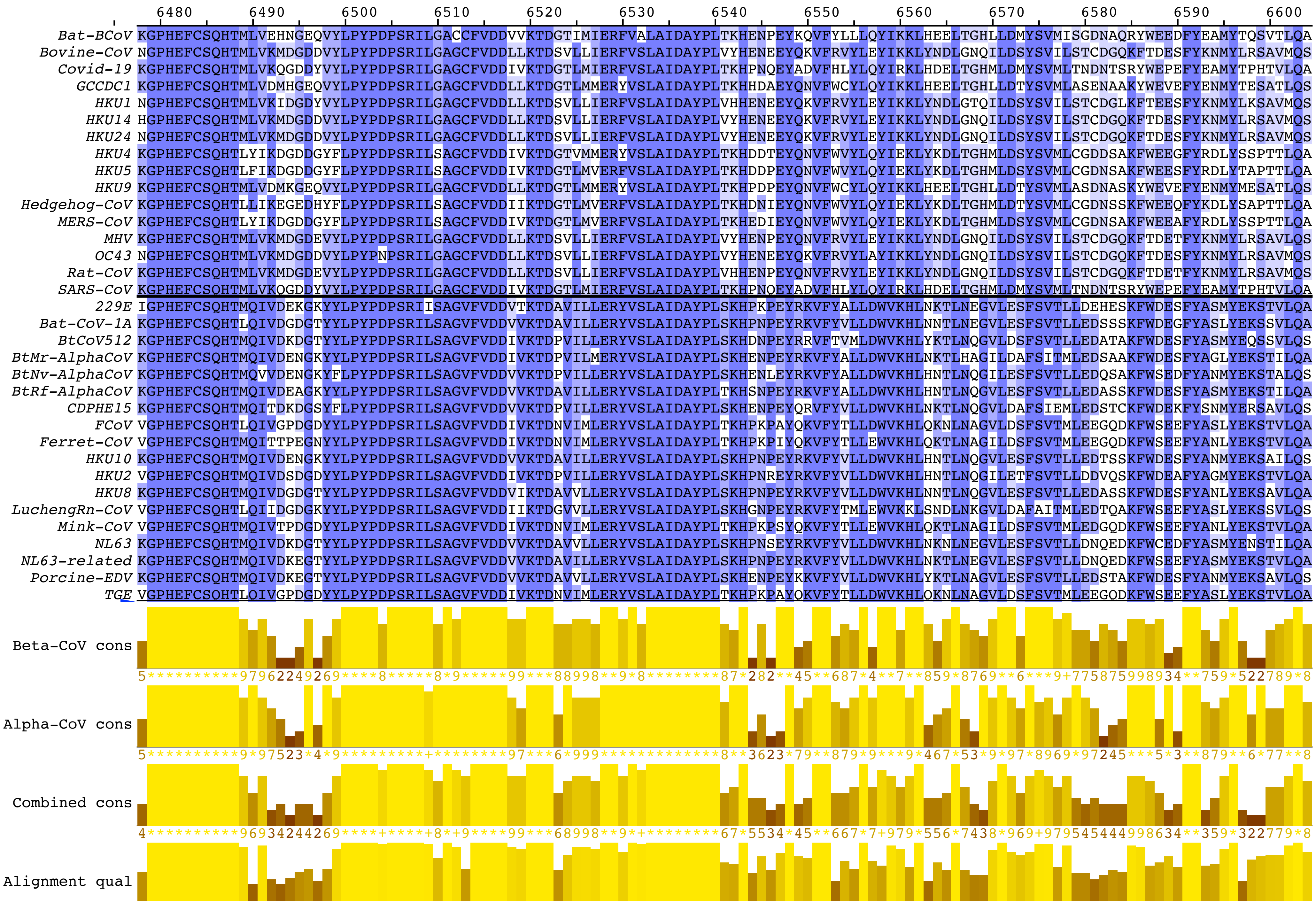
6480 6490 6500 6510 6520 6530 6540 6550 6560 6570 6580 6590 6600
Bat-BCoV KGPHEFCSQHTMLVEHNGEQVYLPYPDPSRILGACCFVDDVVKTDGTIMIERFVALAIDAYPLTKHENPEYKQVFYLLLQYIKKLHEELTGHLLDMYSVMISGDNAQRYWEEDFYEAMYTQSVTLQA
Bovine-CoV NGPHEFCSQHTMLVKMDGDDVYLPYPDPSRILGAGCFVDDLLKTDSVLLIERFVSLAIDAYPLVYHENEEYQKVFRVYLEYIKKLYNDLGNQILDSYSVILSTCDGQKFTDESFYKNMYLRSAVMQS
Covid-19 KGPHEFCSQHTMLVKQGDDYVYLPYPDPSRILGAGCFVDDIVKTDGTLMIERFVSLAIDAYPLTKHPNQEYADVFHLYLQYIRKLHDELTGHMLDMYSVMLTNDNTSRYWEPEFYEAMYTPHTVLQA
GCCDC1 KGPHEFCSQHTMLVDMHGEQVYLPYPDPSRILGAGCFVDDLLKTDGTLMMERYVSLAIDAYPLTKHHDAEYQNVFWCYLQYIKKLHEELTGHLLDTYSVMLASENAAKYWEVEFYENMYTESATLQS
HKU1 NGPHEFCSQHTMLVKIDGDYVYLPYPDPSRILGAGCFVDDLLKTDSVLLIERFVSLAIDAYPLVHHENEEYQKVFRVYLEYIKKLYNDLGTQILDSYSVILSTCDGLKFTEESFYKNMYLKSAVMQS
HKU14 HGPHEFCSQHTMLVKMDGDDVYLPYPDPSRILGAGCFVDDLLKTDSVLLIERFVSLAIDAYPLVYHENEEYQKVFRVYLEYIKKLYNDLGNQILDSYSVILSTCDGQKFTDESFYKNMYLRSAVMQS
HKU24 NGPHEFCSQHTMLVKMDGDDVYLPYPDPSRILGAGCFVDDLLKTDSVLLIERFVSLAIDAYPLVYHENEEYQKVFRVYLEYIKKLYNDLGNQILDSYSVILSTCDGQKFTDESFYKNMYLRSAVMQS
HKU4 KGPHEFCSQHTLYIKDGDDGYFLPYPDPSRILSAGCFVDDIVKTDGTVMMERYVSLAIDAYPLTKHDDTEYQNVFWVYLQYIEKLYKDLTGHMLDSYSVMLCGDDSAKFWEEGFYRDLYSSPTTLQA
HKU5 KGPHEFCSQHTLFIKDGDDGYFLPYPDPSRILSAGCFVDDIVKTDGTLMVERFVSLAIDAYPLTKHDDPEYQNVFWVYLQYIEKLYKDLTGHMLDSYSVMLCGDNSAKFWEESFYRDLYTAPTTLQA
HKU9 KGPHEFCSQHTMLVDMKGEQVYLPYPDPSRILGAGCFVDDLLKTDGTLMMERYVSLAIDAYPLTKHPDPEYQNVFWCYLQYIKKLHEELTGHLLDTYSVMLASDNASKYWEVEFYENMYMESATLQS
Hedgehog-CoV KGPHEFCSQHTLLIKEGEDHYFLPYPDPSRILSAGCFVDDIIKTDGTLMVERFVSLAIDAYPLTKHDNIEYQNVFWVYLQYIEKLYKDLTGHMLDTYSVMLCGDNSSKFWEEQFYKDLYSAPTTLQA
MERS-CoV KGPHEFCSQHTLYIKDGDDGYFLPYPDPSRILSAGCFVDDIVKTDGTLMVERFVSLAIDAYPLTKHEDIEYQNVFWVYLQYIEKLYKDLTGHMLDSYSVMLCGDNSAKFWEEAFYRDLYSSPTTLQA
MHV KGPHEFCSQHTMLVKMDGDEVYLPYPDPSRILGAGCFVDDLLKTDSVLLIERFVSLAIDAYPLVYHENPEYQNVFRVYLEYIKKLYNDLGNQILDSYSVILSTCDGQKFTDETFYKNMYLRSAVLQS
OC43 NGPHEFCSQHTMLVKMDGDDVYLPYPNPSRILGAGCFVDDLLKTDSVLLIERFVSLAIDAYPLVYHENEEYQKVFRVYLAYIKKLYNDLGNQILDSYSVILSTCDGQKFTDESFYKNMYLRSAVMQS
Rat-CoV KGPHEFCSQHTMLVKMDGDEVYLPYPDPSRILGAGCFVDDLLKTDSVLLIERFVSLAIDAYPLVHHENPEYQNVFRVYLEYIKKLYNDLGNQILDSYSVILSTCDGQKFTDETFYKNMYLRSAVMQS
SARS-CoV KGPHEFCSQHTMLVKQGDDYVYLPYPDPSRILGAGCFVDDIVKTDGTLMIERFVSLAIDAYPLTKHPNQEYADVFHLYLQYIRKLHDELTGHMLDMYSVMLTNDNTSRYWEPEFYEAMYTPHTVLQA
229E IGPHEFCSQHTMQIVDEKGKYYLPYPDPSRIISAGVFVDDVTKTDAVILLERYVSLAIDAYPLSKHPKPEYRKVFYALLDWVKHLNKTLNEGVLESFSVTLLDEHESKFWDESFYASMYEKSTVLQA
Bat-CoV-1A KGPHEFCSQHTLQIVDGDGTYYLPYPDPSRILSAGVFVDDIVKTDAVILLERYVSLAIDAYPLSKHPNPEYRKVFYVLLDWVKHLNNTLNEGVLESFSVTLLEDSSSKFWDEGFYASLYEKSSVLQA
BtCoV512 KGPHEFCSQHTMQIVDKDGTYYLPYPDPSRILSAGVFVDDIVKTDPVILLERYVSLAIDAYPLSKHDNPEYRRVFTVMLDWVKHLYKTLNQGVLDSFSVTLLEDATAKFWDESFYASMYEQSSVLQS
BtMr-AlphaCoV KGPHEFCSQHTMQIVDENGKYYLPYPDPSRILSAGVFVDDIVKTDPVILMERYVSLAIDAYPLSKHENPEYRKVFYALLDWVKHLNKTLHAGILDAFSITMLEDSAAKFWDESFYAGLYEKSTILQA
BtNv-AlphaCoV KGPHEFCSQHTMQVVDENGKYFLPYPDPSRILSAGVFVDDVVKTDPVILLERYVSLAIDAYPLSKHENLEYRKVFYALLDWVKHLHNTLNQGILESFSVTLLEDQSAKFWSEDFYANMYEKSTALQS
BtRf-AlphaCoV KGPHEFCSQHTMQIVDEAGKYYLPYPDPSRILSAGVFVDDVVKTDAVILLERYVSLAIDAYPLTKHSNPEYRKVFYALLDWVKHLHNTLNQGVLESFSVTLLEDASSKFWDESFYASMYEKSTILQA
CDPHE15 KGPHEFCSQHTMQITDKDGSYFLPYPDPSRILSAGVFVDDVVKTDPVILLERYVSLAIDAYPLSKHENPEYQRVFYVLLDWVKHLNKTLNQGVLDAFSIEMLEDSTCKFWDEKFYSNMYERSAVLQS
FCoV VGPHEFCSQHTLQIVGPDGDYYLPYPDPSRILSAGVFVDDIVKTDNVIMLERYVSLAIDAYPLTKHPKPAYQKVFYTLLDWVKHLQKNLNAGVLDSFSVTMLEEGQDKFWSEEFYASLYEKSTVLQA
Ferret-CoV VGPHEFCSQHTMQITTPEGNYYLPYPDPSRILSAGVFVDDIVKTDNVIMLERYVSLAIDAYPLTKHPKPIYQKVFYTLLEWVKHLQKTLNAGILDSFSVTMLEEGQDKFWSEEFYANLYEKSTVLQA
HKU10 KGPHEFCSQHTMQIVDENGKYYLPYPDPSRILSAGVFVDDVVKTDAVILLERYVSLAIDAYPLSKHPNPEYRKVFYVLLDWVKHLHNTLNQGVLESFSVTLLEDTSSKFWDESFYANMYEKSAILQS
HKU2 VGPHEFCSQHTMQIVDSDGDYYLPYPDPSRILSAGVFVDDIVKTDPVILLERYVSLAIDAYPLSKHPNREYRKVFYVLLDWVKHLHNTLNQGILETFSVTLLDDVQSKFWDEAFYAGMYEKSTVLQA
HKU8 KGPHEFCSQHTMQIVDGDGTYYLPYPDPSRILSAGVFVDDVIKTDAVVLLERYVSLAIDAYPLSKHPNPEYRKVFYVLLDWVKHLNNTLNQGVLESFSVTLLEDASSKFWDESFYANLYEKSAVLQA
LuchengRn-CoV VGPHEFCSQHTLQIIDGDGKYYLPYPDPSRILSAGVFVDDIIKTDGVVLLERYVSLAIDAYPLSKHGNPEYRKVFYTMLEWVKKLSNDLNKGVLDAFAITMLEDTQAKFWSEEFYASLYEKSSVLQS
Mink-CoV VGPHEFCSQHTMQIVTPDGDYYLPYPDPSRILSAGVFVDDIVKTDNVIMLERYVSLAIDAYPLTKHPKPSYQKVFYTLLEWVKHLQKTLNAGILDSFSVTMLEDGQDKFWSEEFYANLYEKSTVLQA
NL63 KGPHEFCSQHTMQIVDKDGTYYLPYPDPSRILSAGVFVDDVVKTDAVVLLERYVSLAIDAYPLSKHPNSEYRKVFYVLLDWVKHLNKNLNEGVLESFSVTLLDNQEDKFWCEDFYASMYENSTILQA
NL63-related KGPHEFCSQHTMQIVDKEGTYYLPYPDPSRILSAGVFVDDVVKTDAVILLERYVSLAIDAYPLSKHPNPEYRKVFYVLLDWVKHLNKTLNEGVLESFSVTLLDNQEDKFWSEEFYASMYEKSTVLQA
Porcine-EDV KGPHEFCSQHTMQIVDKEGTYYLPYPDPSRILSAGVFVDDVVKTDAVVLLERYVSLAIDAYPLSKHENPEYKKVFYVLLDWVKHLYKTLNAGVLESFSVTLLEDSTAKFWDESFYANMYEKSAVLQS
TGE VGPHEFCSQHTLQIVGPDGDYYLPYPDPSRILSAGVFVDDIVKTDNVIMLERYVSLAIDAYPLTKHPKPAYQKVFYTLLDWVKHLQKNLNAGVLDSFSVTMLEEGQDKFWSEEFYASLYEKSTVLQA
Beta-CoV cons
5*********9796224926 9****8*****8*9*****99***88998**9*8********87*282**45**687*4**7**859*8769**6***9+775875998934**759*522789*8
Alpha-CoV cons
5*********9*97523*4*9*********+********97***6*999************8**3623*79**879*9***9*467*53*9*97*8969*97245***5*3**879**6*77**8
Combined cons
4*********969342442 69****+****+8*+9****99***68998**9*+********67*5534*45**667*7+979*556*7438*969+979545444998634**359*322779*8
Alignment qual

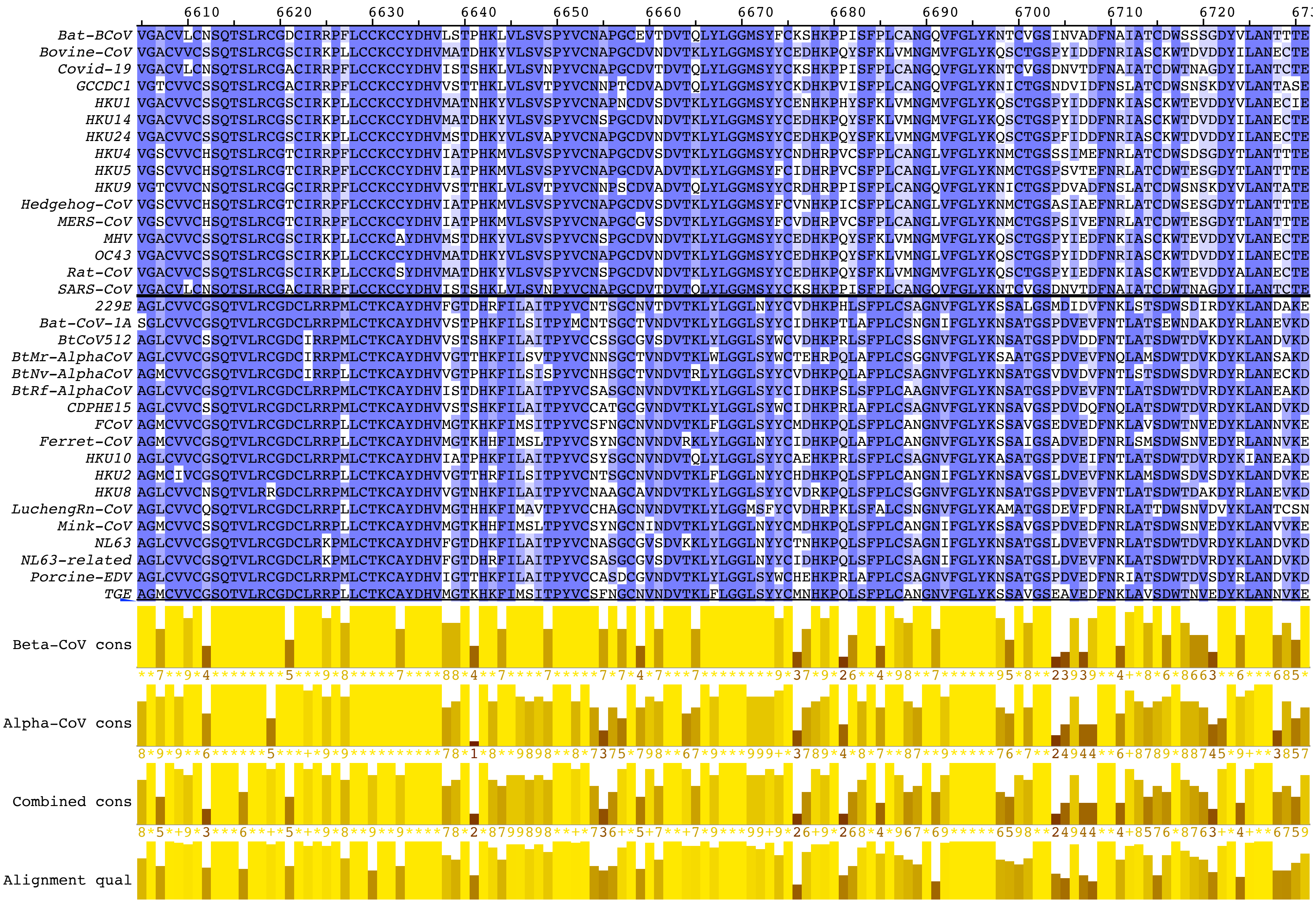
6610
6620
6630
6640
6650
6660
6670
6680
6690
6700
6710
6720
Bat-BCoV
Bovine-CoV
Covid-19
GCCDC1
HKU1
HKU14
HKU24
HKU4
HKU5
HKU9
Hedgehog-CoV
MERS-CoV
MHV
OC43
Rat-CoV
SARS-CoV
229E
Bat-CoV-1A
BtCoV512
BtMr-AlphaCoV
BtNv-AlphaCoV
BtRf-AlphaCoV
CDPHE15
FCoV
Ferret-CoV
HKU10
HKU2
HKU8
LuchengRn-CoV
Mink-CoV
NL63
NL63-related
Porcine-EDV
TGE
Beta-CoV cons
Alpha-CoV cons
Combined cons
Alignment qual

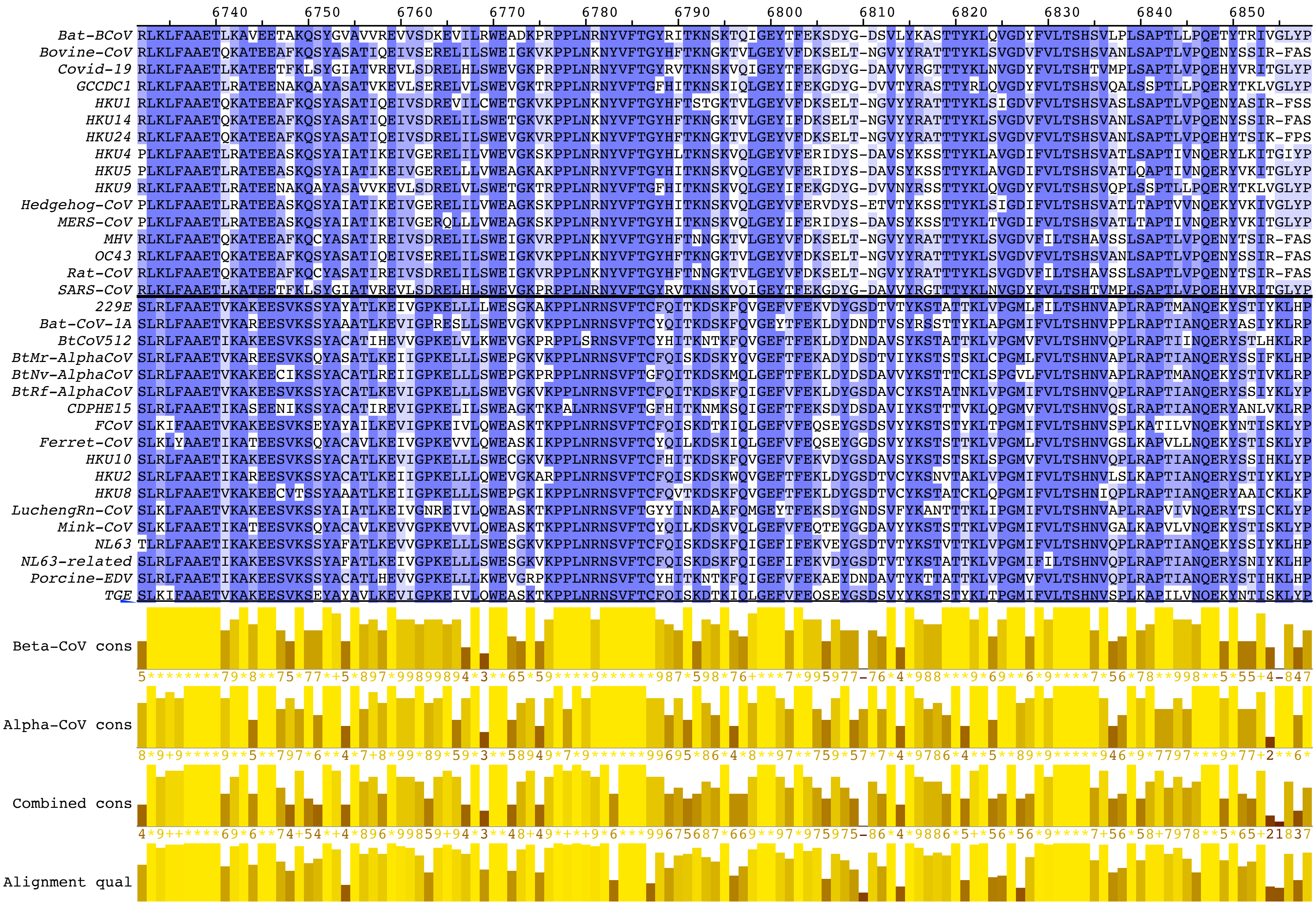

6740
6750
6760
6770
6780
6790
6800
6810
6820
6830
6840
6850
Bat-BCoV
Bovine-CoV
Covid-19
GCCDC1
HKU1
HKU14
HKU24
HKU4
HKU5
HKU9
Hedgehog-CoV
MERS-CoV
MHV
OC43
Rat-CoV
SARS-CoV
229E
Bat-CoV-1A
BtCoV512
BtMr-AlphaCoV
BtNv-AlphaCoV
BtRf-AlphaCoV
CDPHE15
FCoV
Ferret-CoV
HKU10
HKU2
HKU8
LuchengRn-CoV
Mink-CoV
NL63
NL63-related
Porcine-EDV
TGE
Beta-CoV cons
Alpha-CoV cons
Combined cons
Alignment qual

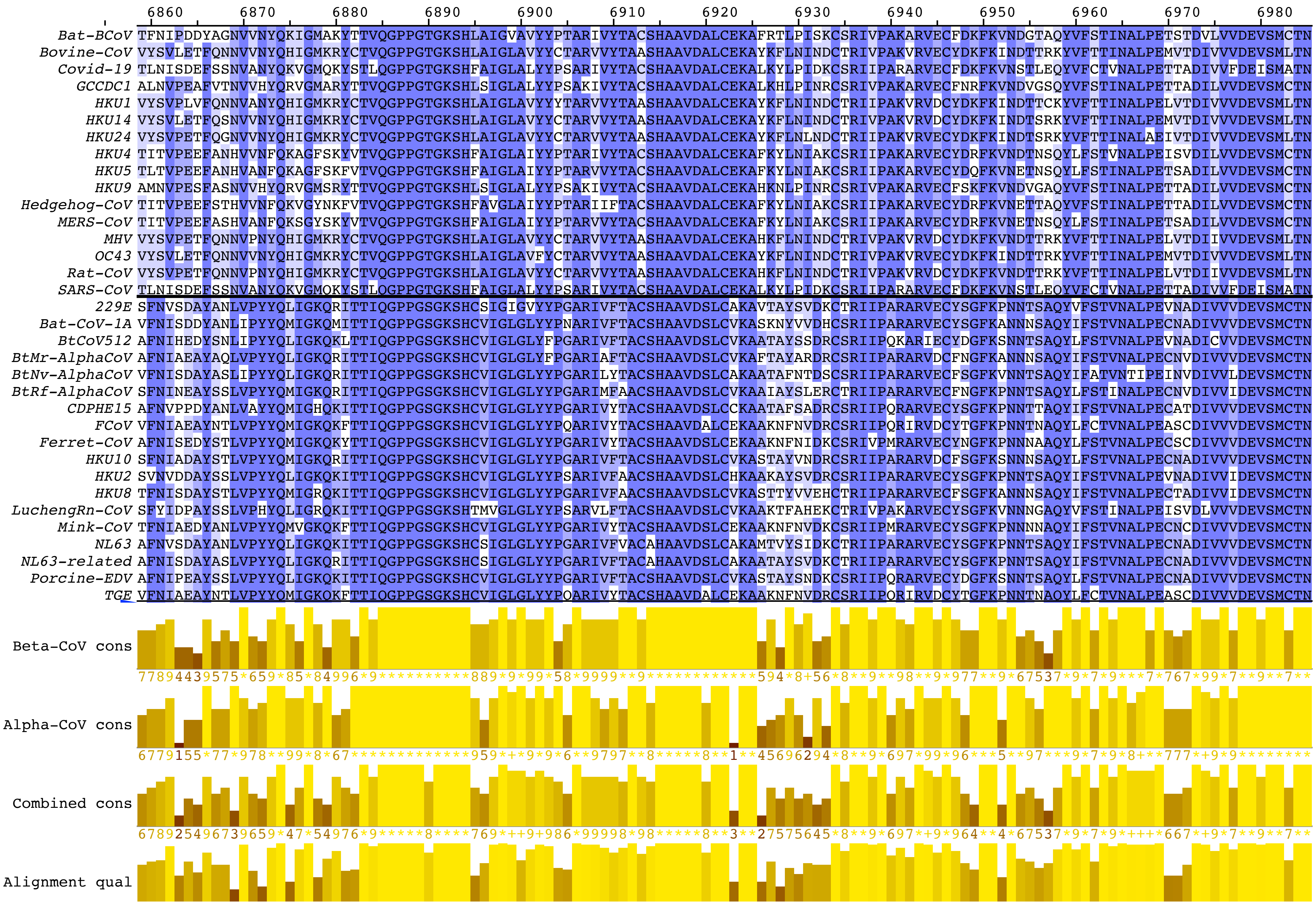
6860 6870 6880 6890 6900 6910 6920 6930 6940 6950 6960 6970 6980
Bat-BCoV TFNIPDDYAGNVVNYQKIGMAKYTTVQGPPGTGKSHLAIGVAVYYPTARIVYTACSHAAVDALCEKAFRTLPISKCSRIVPAKARVECFDKFKVNDGTAQYVFSTINALPETSTDVLVVDEVSMCTN
Bovine-CoV VYSVLETFQNNVVNYQHIGMKRYCTVQGPPGTGKSHLAIGLAVYYCTARVVYTAASHAAVDALCEKAYKFLNINDCTRIVPAKVRVECYDKFKINDTTRKYVFTTINALPEMVTDIVVVDEVSMLTN
Covid-19 TLNISDEFSSNVANYQKVGMQKYSTLQGPPGTGKSHFAIGLALYYPSARIVYTACSHAAVDALCEKALKYLPIDKCSRIIPARARVECFDKFKVNSTLEQYVFCTVNALPETTADIVVFDEISMATN
GCCDC1 ALNVPEAFVTNVVHYQRVGMARYTTVQGPPGTGKSHLSIGLALYYPSAKIVYTACSHAAVDALCEKALKHLPINRCSRIVPAKARVECFNRFKVNDVGSQYVFSTINALPETTADILVVDEVSMCTN
HKU1 VYSVPLVFQNNVANYQHIGMKRYCTVQGPPGTGKSHLAIGLAVYYYTARVVYTAASHAAVDALCEKAYKFLNINDCTRIIPAKVRVDCYDKFKINDTTCKYVFTTINALPELVTDIVVVDEVSMLTN
HKU14 VYSVLETFQSNVVNYQHIGMKRYCTVQGPPGTGKSHLAIGLAVYYCTARVVYTAASHAAVDALCEKAYKFLNINDCTRIVPAKVRVDCYDKFKINDTSRKYVFTTINALPEMVTDIVVVDEVSMLTN
HKU24 VYSVPETFQGNVVNYQHIGMKRYCTVQGPPGTGKSHLAIGLAVYYCTARVVYTAASHAAVDALCEKAYKFLNLNDCTRIIPAKVRVECYDKFKINDTSRKYVFTTINALAEIVTDIVVVDEVSMLTN
HKU4 TITVPEEFANHVVNFQKAGFSKYVTVQGPPGTGKSHFAIGLAIYYPTARIVYTACSHAAVDALCEKAFKYLNIAKCSRIIPAKARVECYDRFKVNDTNSQYLFSTVNALPEISVDILVVDEVSMCTN
HKU5 TLTVPEEFANHVANFQKAGFSKFVTVQGPPGTGKSHFAIGLAIYYPTARVVYTACSHAAVDALCEKAFKYLNIAKCSRIIPAKARVECYDQFKVNETNSQYLFSTINALPETSADILVVDEVSMCTN
HKU9 AMNVPESFASNVVHYQRVGMSRYTTVQGPPGTGKSHLSIGLALYYPSAKIVYTACSHAAVDALCEKAHKNLPINRCSRIVPAKARVECFSKFKVNDVGAQYVFSTINALPETTADILVVDEVSMCTN
Hedgehog-CoV TITVPEEFSTHVVNFQKVGYNKFVTVQGPPGTGKSHFAVGLAIYYPTARIIFTACSHAAVDALCEKAFKYLNIAKCSRIIPAKARVECYDRFKVNETTAQYVFSTINALPETTADILVVDEVSMCTN
MERS-CoV TITVPEEFASHVANFQKSGYSKYVTVQGPPGTGKSHFAIGLAIYYPTARVVYTACSHAAVDALCEKAFKYLNIAKCSRIIPAKARVECYDRFKVNETNSQYLFSTINALPETSADILVVDEVSMCTN
MHV VYSVPETFQNNVPNYQHIGMKRYCTVQGPPGTGKSHLAIGLAVYYCTARVVYTAASHAAVDALCEKAHKFLNINDCTRIVPAKVRVDCYDKFKVNDTTRKYVFTTINALPELVTDIIVVDEVSMLTN
OC43 VYSVLETFQNNVVNYQHIGMKRYCTVQGPPGTGKSHLAIGLAVFYCTARVVYTAASHAAVDALCEKAYKFLNINDCTRIVPAKVRVECYDKFKINDTTRKYVFTTINALPEMVTDIVVVDEVSMLTN
Rat-CoV VYSVPETFQNNVPNYQHIGMKRYCTVQGPPGTGKSHLAIGLAVYYCTARVVYTAASHAAVDALCEKAHKFLNINDCTRIVPAKVRVDCYDKFKVNDTTRKYVFTTINALPELVTDIIVVDEVSMLTN
SARS-CoV TLNISDEFSSNVANYQKVGMQKYSTLQGPPGTGKSHFAIGLALYYPSARIVYTACSHAAVDALCEKALKYLPIDKCSRIIPARARVECFDKFKVNSTLEQYVFCTVNALPETTADIVVFDEISMATN
229E SFNVSDAYANLVPYYQLIGKQRITTIQGPPGSGKSHCSIGIGVYYPGARIVFTACSHAAVDSLCAKAVTAYSVDKCTRIIPARARVECYSGFKPNNTSAQYVFSTVNALPEVNADIVVVDEVSMCTN
Bat-CoV-1A VFNISDDYANLIPYYQMIGKQMITTIQGPPGSGKSHCVIGLGLYYPNARIVFTACSHAAVDSLCVKASKNYVVDHCSRIIPARARVECYSGFKANNNSAQYIFSTVNALPECNADIVVVDEVSMCTN
BtCoV512 AFNIHEDYSNLIPYYQLIGKQKLTTIQGPPGSGKSHCVIGLGLYFPGARIVFTACSHAAVDSLCVKAATAYSSDRCSRIIPQKARIECYDGFKSNNTSAQYLFSTVNALPEVNADICVVDEVSMCTN
BtMr-AlphaCoV AFNIAEAYAQLVPYYQLIGKQRITTIQGPPGSGKSHCVIGLGLYFPGARIAFTACSHAAVDSLCVKAFTAYARDRCSRIIPARARVDCFNGFKANNNSAQYIFSTVNALPECNVDIVVVDEVSMCTN
BtNv-AlphaCoV VFNISDAYASLIPYYQLIGKQRITTIQGPPGSGKSHCVIGLGLYYPGARILYTACSHAAVDSLCAKAATAFNTDSCSRIIPARARVECFSGFKVNNTSAQYIFATVNTIPEINVDIVVLDEVSMCTN
BtRf-AlphaCoV SFNINEAYSSLVPYYQMIGKQRITTIQGPPGSGKSHCVIGLGLYYPGARIMFAACSHAAVDSLCVKAAIAYSLERCTRIIPARARVECFNGFKPNNTSAQYLFSTINALPECNVDIVVIDEVSMCTN
CDPHE15 AFNVPPDYANLVAYYQMIGHQKITTIQGPPGSGKSHCVIGLGLYYPGARIVYTACSHAAVDSLCCKAATAFSADRCSRIIPQRARVECYSGFKPNNTTAQYIFSTVNALPECATDIVVVDEVSMCTN
FCoV VFNIAEAYNTLVPYYQMIGKQKFTTIQGPPGSGKSHCVIGLGLYYPQARIVYTACSHAAVDALCEKAAKNFNVDRCSRIIPQRIRVDCYTGFKPNNTNAQYLFCTVNALPEASCDIVVVDEVSMCTN
Ferret-CoV AFNISEDYSTLVPYYQMIGKQKYTTIQGPPGSGKSHCVIGLGLYYPGARIVYTACSHAAVDSLCEKAAKNFNIDKCSRIVPMRARVECYNGFKPNNNAAQYLFSTVNALPECSCDIVVVDEVSMCTN
HKU10 SFNIADAYSTLVPYYQMIGKQRITTIQGPPGSGKSHCVIGLGLYYPGARIVFTACSHAAVDSLCVKASTAYVNDRCSRIIPARARVDCFSGFKSNNNSAQYLFSTVNALPECNADIVVVDEVSMCTN
HKU2 SVNVDDAYSSLVPYYQLIGKQKITTIQGPPGSGKSHCVIGLGLYYPSARIVFAACSHAAVDSLCHKAAKAYSVDRCSRIIPARARVECYSGFKPNNTSAQYIFSTVNALPEVNADIVVIDEVSMCTN
HKU8 TFNISDAYSTLVPYYQMIGRQKITTIQGPPGSGKSHCVIGLGLYYPGARIVFAACSHAAVDSLCVKASTTYVVEHCTRIIPARARVECFSGFKANNNSAQYIFSTVNALPECTADIVVIDEVSMCTN
LuchengRn-CoV SFYIDPAYSSLVPHYQLIGRQKITTIQGPPGSGKSHTMVGLGLYYPSARVLFTACSHAAVDSLCVKAAKTFAHEKCTRIVPAKARVECYSGFKVNNNGAQYVFSTINALPEISVDLVVVDEVSMCTN
Mink-CoV TFNIAEDYANLVPYYQMVGKQKFTTIQGPPGSGKSHCVIGLGLYYPGARIVYTACSHAAVDSLCEKAAKNFNVDKCSRIIPMRARVECYSGFKPNNNNAQYIFSTVNALPECNCDIVVVDEVSMCTN
NL63 AFNVSDAYANLVPYYQLIGKQKITTIQGPPGSGKSHCSIGLGLYYPGARIVFVACAHAAVDSLCAKAMTVYSIDKCTRIIPARARVECYSGFKPNNTSAQYIFSTVNALPECNADIVVVDEVSMCTN
NL63-related AFNISDAYASLVPYYQLIGKQRITTIQGPPGSGKSHCSIGLGLYYPGARIVFTACAHAAVDSLCAKAATAYSVDKCTRIIPARARVECYSGFKPNNTSAQYIFSTVNALPECNADIVVVDEVSMCTN
Porcine-EDV AFNIPEAYSSLVPYYQLIGKQKITTIQGPPGSGKSHCVIGLGLYYPGARIVFTACSHAAVDSLCVKASTAYSNDKCSRIIPQRARVECYDGFKSNNTSAQYLFSTVNALPECNADIVVVDEVSMCTN
TGE VFNIAEAYNTLVPYYQMIGKQKFTTIQGPPGSGKSHCVIGLGLYYPQARIVYTACSHAAVDALCEKAAKNFNVDRCSRIIPQRIRVDCYTGFKPNNTNAQYLFCTVNALPEASCDIVVVDEVSMCTN
Beta-CoV cons
7789443957 5*659*85*84996*9**********889*9*99*58*9999**9***********594*8+56*8**9**98**9*977**9*67537*9*7*9***7*767*99*7**9**7**
Alpha-CoV cons
6779155*77*978**99*8*67************959*+*9*9*6**9797**8*****8**1**45696294*8**9*697*99*96***5**97***9*7*9*8+**777*+9*9********
Combined cons
6789254967396 59*47*54976*9*****8****769*++9+986*99998*98*****8**3**27575645*8**9*697*+9*964**4*67537*9*7*9*+++*667*+9*7**9**7**
Alignment qual

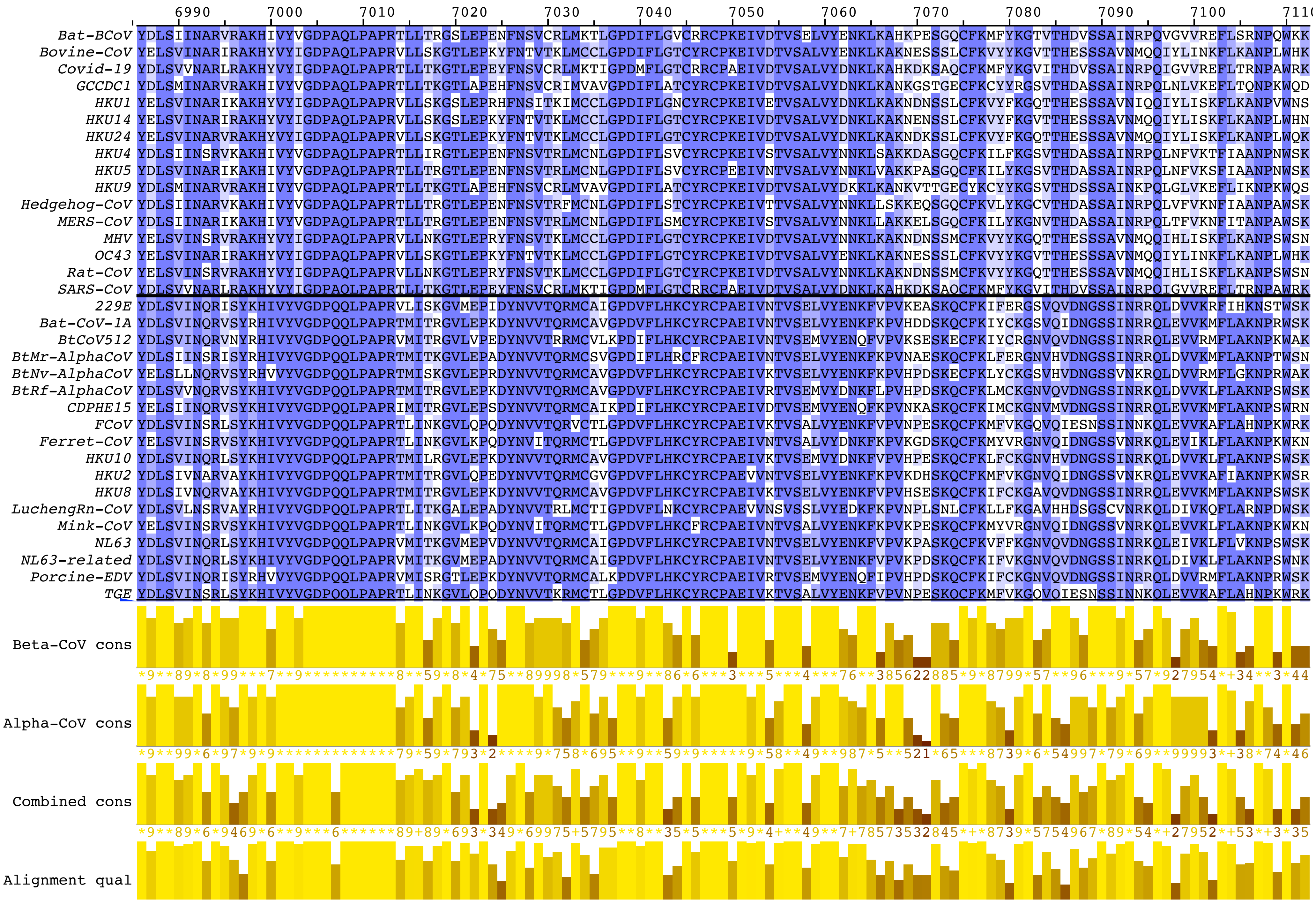
6990 7000 7010 7020 7030 7040 7050 7060 7070 7080 7090 7100
Bat-BCoV YDLSIINARVRAKHIVYVGDPAQLPAPRTLLTRGSLEPENFNSVCRLMKTLGPDIFLGVCRRCPKEIVDTVSELVYENKLKAHKPESGQCFKMFYKGTVTHDVSSAINRPQVGVVREFLSRNPQWKK
Bovine-CoV YELSVINARIRAKHYVYIGDPAQLPAPRVLLSKGTLEPKYFNTVTKLMCCLGPDIFLGTCYRCPKEIVDTVSALVYENKLKAKNESSSLCFKVYYKGVTTHESSSAVNMQQIYLINKFLKANPLWHK
Covid-19 YDLSVVNARLRAKHYVYIGDPAQLPAPRTLLTKGTLEPEYFNSVCRLMKTIGPDMFLGTCRRCPAEIVDTVSALVYDNKLKAHKDKSAQCFKMFYKGVITHDVSSAINRPQIGVVREFLTRNPAWRK
GCCDC1 YDLSMINARVRAKHIVYVGDPAQLPAPRTLLTKGTLAPEHFNSVCRIMVAVGPDIFLATCYRCPKEIVDTVSALVYDNKLKANKGSTGECFKCYYRGSVTHDASSAINRPQLNLVKEFLTQNPKWQD
HKU1 YELSVINARIKAKHYVYIGDPAQLPAPRVLLSKGSLEPRHFNSITKIMCCLGPDIFLGNCYRCPKEIVETVSALVYDNKLKAKNDNSSLCFKVYFKGQTTHESSSAVNIQQIYLISKFLKANPVWNS
HKU14 YELSVINARIRAKHYVYIGDPAQLPAPRVLLSKGSLEPKYFNTVTKLMCCLGPDIFLGTCYRCPKEIVDTVSALVYDNKLKAKNENSSLCFKVYFKGVTTHESSSAVNMQQIYLISKFLKANPLWHN
HKU24 YELSVINARVRAKHYVYIGDPAQLPAPRVLLSKGTLEPKYFNTVTKLMCCLGPDIFLGTCYRCPKEIVDTVSALVYDNKLKAKNDKSSLCFKVYFKGQTTHESSSAVNMQQIYLISKFLKANPLWQK
HKU4 YDLSIINSRVKAKHIVYVGDPAQLPAPRTLLIRGTLEPENFNSVTRLMCNLGPDIFLSVCYRCPKEIVSTVSALVYNNKLSAKKDASGQCFKILFKGSVTHDASSAINRPQLNFVKTFIAANPNWSK
HKU5 YDLSVINARIKAKHIVYVGDPAQLPAPRTLLTRGTLEPENFNSVTRLMCNLGPDIFLSVCYRCPEEIVNTVSALVYNNKLVAKKPASGQCFKILYKGSVTHDASSAINRPQLNFVKSFIAANPNWSK
HKU9 YDLSMINARVRAKHIVYVGDPAQLPAPRTLLTKGTLAPEHFNSVCRLMVAVGPDIFLATCYRCPKEIVDTVSALVYDKKLKANKVTTGECYKCYYKGSVTHDSSSAINKPQLGLVKEFLIKNPKWQS
Hedgehog-CoV YDLSIINARVKAKHIVYVGDPAQLPAPRTLLTRGTLEPENFNSVTRFMCNLGPDIFLSTCYRCPKEIVTTVSALVYNNKLLSKKEQSGQCFKVLYKGCVTHDASSAINRPQLVFVKNFIAANPAWSK
MERS-CoV YDLSIINARIKAKHIVYVGDPAQLPAPRTLLTRGTLEPENFNSVTRLMCNLGPDIFLSMCYRCPKEIVSTVSALVYNNKLLAKKELSGQCFKILYKGNVTHDASSAINRPQLTFVKNFITANPAWSK
MHV YELSVINSRVRAKHYVYIGDPAQLPAPRVLLNKGTLEPRYFNSVTKLMCCLGPDIFLGTCYRCPKEIVDTVSALVYNNKLKAKNDNSSMCFKVYYKGQTTHESSSAVNMQQIHLISKFLKANPSWSN
OC43 YELSVINARIRAKHYVYIGDPAQLPAPRVLLSKGTLEPKYFNTVTKLMCCLGPDIFLGTCYRCPKEIVDTVSALVYENKLKAKNESSSLCFKVYYKGVTTHESSSAVNMQQIYLINKFLKANPLWHK
Rat-CoV YELSVINSRVRAKHYVYIGDPAQLPAPRVLLNKGTLEPRYFNSVTKLMCCLGPDIFLGTCYRCPKEIVDTVSALVYNNKLKAKNDNSSMCFKVYYKGQTTHESSSAVNMQQIHLISKFLKANPSWSN
SARS-CoV YDLSVVNARLRAKHYVYIGDPAQLPAPRTLLTKGTLEPEYFNSVCRLMKTIGPDMFLGTCRRCPAEIVDTVSALVYDNKLKAHKDKSAQCFKMFYKGVITHDVSSAINRPQIGVVREFLTRNPAWRK
229E YDLSVINQRISYKHIVYVGDPQQLPAPRVLISKGVMEPIDYNVVTQRMCAIGPDVFLHKCYRCPAEIVNTVSELVYENKFVPVKEASKQCFKIFERGSVQVDNGSSINRRQLDVVKRFIHKNSTWSK
Bat-CoV-1A YDLSVINQRVSYRHIVYVGDPQQLPAPRTMITRGVLEPKDYNVVTQRMCAVGPDVFLHKCYRCPAEIVNTVSELVYENKFKPVHDDSKQCFKIYCKGSVQIDNGSSINRRQLEVVKMFLAKNPRWSK
BtCoV512 YDLSVINQRVNYRHIVYVGDPQQLPAPRVMITRGVLVPEDYNVVTRRMCVLKPDIFLHKCYRCPAEIVNTVSEMVYENQFVPVKSESKECFKIYCRGNVQVDNGSSINRRQLEVVRMFLAKNPKWAK
BtMr-AlphaCoV YDLSIINSRISYRHIVYVGDPQQLPAPRTMITKGVLEPADYNVVTQRMCSVGPDIFLHRCFRCPAEIVNTVSELVYENKFKPVNAESKQCFKLFERGNVHVDNGSSINRRQLDVVKMFLAKNPTWSN
BtNv-AlphaCoV YELSLLNQRVSYRHVVYVGDPQQLPAPRTMISKGVLEPRDYNVVTQRMCAVGPDVFLHKCYRCPAEIVKTVSELVYENKFKPVHPDSKECFKLYCKGSVHVDNGSSVNKRQLDVVRMFLGKNPRWAK
BtRf-AlphaCoV YDLSVVNQRVSYKHIVYVGDPQQLPAPRTMITRGVLEPKDYNVVTQRMCAVGPDVFLHKCYRCPAEIVRTVSEMVYDNKFLPVHPDSKQCFKLMCKGNVQVDNGSSINRKQLDVVKLFLAKNPSWSK
CDPHE15 YELSIINQRVSYKHIVYVGDPQQLPAPRIMITRGVLEPSDYNVVTQRMCAIKPDIFLHKCYRCPAEIVDTVSEMVYENQFKPVNKASKQCFKIMCKGNVMVDNGSSINRRQLEVVKMFLAKNPSWRN
FCoV YDLSVINSRLSYKHIVYVGDPQQLPAPRTLINKGVLQPQDYNVVTQRVCTLGPDVFLHKCYRCPAEIVKTVSALVYENKFVPVNPESKQCFKMFVKGQVQIESNSSINNKQLEVVKAFLAHNPKWRK
Ferret-CoV YELSVINSRVSYKHIVYVGDPQQLPAPRTLINKGVLKPQDYNVITQRMCTLGPDVFLHKCYRCPAEIVNTVSALVYDNKFKPVKGDSKQCFKMYVRGNVQIDNGSSVNRKQLEVIKLFLAKNPKWKN
HKU10 YDLSVINQRLSYKHIVYVGDPQQLPAPRTMILRGVLEPKDYNVVTQRMCAVGPDVFLHKCYRCPAEIVKTVSEMVYDNKFVPVHPESKQCFKLFCKGNVHVDNGSSINRKQLDVVKLFLAKNPSWSK
HKU2 YDLSIVNARVAYKHIVYVGDPQQLPAPRTMITRGVLQPEDYNVVTQRMCGVGPDVFLHKCYRCPAEVVNTVSELVYENKFKPVKDHSKQCFKMFVKGNVQIDNGSSVNKRQLEVVKAFIAKNPKWSR
HKU8 YDLSIVNQRVAYKHIVYVGDPQQLPAPRTMITRGVLEPKDYNVVTQRMCAVGPDVFLHKCYRCPAEIVKTVSELVYENKFVPVHSESKQCFKIFCKGAVQVDNGSSINRRQLEVVKMFLAKNPSWSK
LuchengRn-CoV YDLSVLNSRVAYRHIVYVGDPQQLPAPRTLITKGALEPADYNVVTRLMCTIGPDVFLNKCYRCPAEVVNSVSSLVYEDKFKPVNPLSNLCFKLLFKGAVHHDSGSCVNRKQLDIVKQFLARNPDWSK
Mink-CoV YELSVINSRVSYKHIVYVGDPQQLPAPRTLINKGVLKPQDYNVITQRMCTLGPDVFLHKCFRCPAEIVNTVSALVYENKFKPVKPESKQCFKMYVRGNVQIDNGSSVNRKQLEVVKLFLAKNPKWKN
NL63 YDLSVINQRLSYKHIVYVGDPQQLPAPRVMITKGVMEPVDYNVVTQRMCAIGPDVFLHKCYRCPAEIVNTVSELVYENKFVPVKPASKQCFKVFFKGNVQVDNGSSINRKQLEIVKLFLVKNPSWSK
NL63-related YDLSVINQRLSYKHIVYVGDPQQLPAPRVMITKGVMEPADYNVVTQRMCAIGPDVFLHKCYRCPAEIVNTVSELVYENKFVPVKPDSKQCFKIFVKGNVQVDNGSSINRKQLDIVKLFLAKNPSWNK
Porcine-EDV YDLSVINQRISYRHVVYVGDPQQLPAPRVMISRGTLEPKDYNVVTQRMCALKPDVFLHKCYRCPAEIVRTVSEMVYENQFIPVHPDSKQCFKIFCKGNVQVDNGSSINRRQLDVVRMFLAKNPRWSK
TGE YDLSVINSRLSYKHIVYVGDPQQLPAPRTLINKGVLQPQDYNVVTKRMCTLGPDVFLHKCYRCPAEIVKTVSALVYENKFVPVNPESKQCFKMFVKGQVQIESNSSINNKQLEVVKAFLAHNPKWRK
Beta-CoV cons
Alpha-CoV cons
Combined cons
Alignment qual

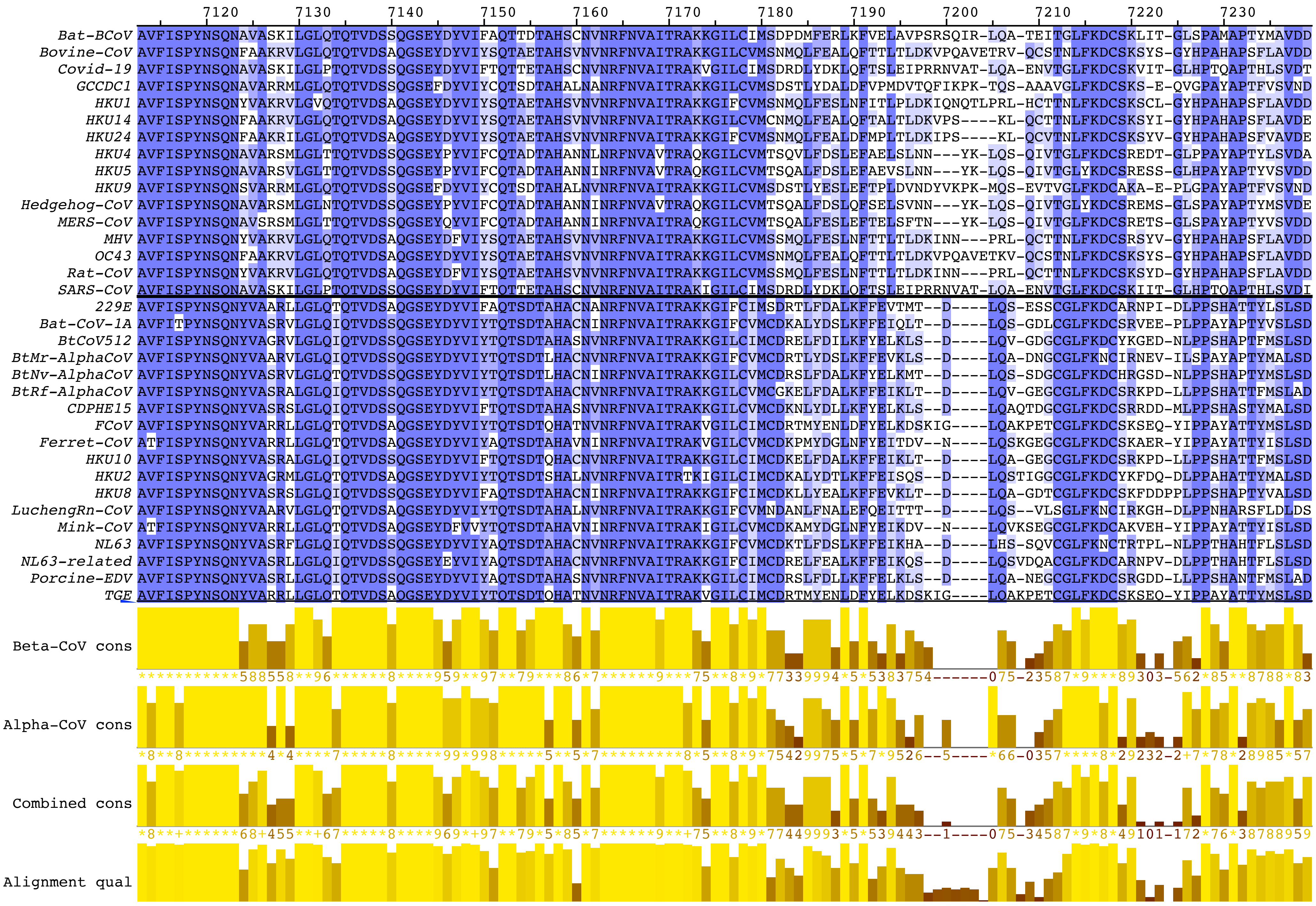
7120 7130 7140 7150 7160 7170 7180 7190 7200 7210 7220 7230
Bat-BCoV AVFISPYNSQNAVASKILGLQTQTVDSSQGSEYDYVIFAQTTDTAHSCNVNRFNVAITRAKKGILCIMSDPDMFERLKFVELAVPSRSQIR-LQA-TEITGLFKDCSKLIT-GLSPAMAPTYMAVDD
Bovine-CoV AVFISPYNSQNFAAKRVLGLQTQTVDSAQGSEYDYVIYSQTAETAHSVNVNRFNVAITRAKKGILCVMSNMQLFEALQFTTLTLDKVPQAVETRV-QCSTNLFKDCSKSYS-GYHPAHAPSFLAVDD
Covid-19 AVFISPYNSQNAVASKILGLPTQTVDSSQGSEYDYVIFTQTTETAHSCNVNRFNVAITRAKVGILCIMSDRDLYDKLQFTSLEIPRRNVAT-LQA-ENVTGLFKDCSKVIT-GLHPTQAPTHLSVDT
GCCDC1 AVFISPYNSQNAVARRMLGLQTQTVDSSQGSEFDYVIYCQTSDTAHALNANRFNVAITRAKKGILCVMSDSTLYDALDFVPMDVTQFIKPK-TQS-AAAVGLFKDCSKS-E-QVGPAYAPTFVSVND
HKU1 AVFISPYNSQNYVAKRVLGVQTQTVDSAQGSEYDYVIYSQTAETAHSVNVNRFNVAITRAKKGIFCVMSNMQLFESLNFITLPLDKIQNQTLPRL-HCTTNLFKDCSKSCL-GYHPAHAPSFLAVDD
HKU14 AVFISPYNSQNFAAKRILGLQTQTVDSAQGSEYDYVIYSQTAETAHSVNVNRFNVAITRAKKGILCVMCNMQLFEALQFTALTLDKVPS----KL-QCTTNLFKDCSKSYI-GYHPAHAPSFLAVDE
HKU24 AVFISPYNSQNFAAKRVLGLQTQTVDSAQGSEYDYVIYSQTAETAHSVNVNRFNVAITRAKKGIFCVMSNMQLFEALDFMPLTLDKIPS----KL-QCVTNLFKDCSKSYV-GYHPAHAPSFVAVDE
HKU4 AVFISPYNSQNAVARSMLGLTTQTVDSSQGSEYPYVIFCQTADTAHANNLNRFNVAVTRAQKGILCVMTSQVLFDSLEFAELSLNN---YK-LQS-QIVTGLFKDCSREDT-GLPPAYAPTYLSVDA
HKU5 AVFISPYNSQNAVARSVLGLTTQTVDSSQGSEYPYVIFCQTADTAHANNINRFNVAVTRAQKGILCVMTSQALFDSLEFAEVSLNN---YK-LQS-QIVTGLYKDCSRESS-GLHPAYAPTYVSVDD
HKU9 AVFISPYNSQNSVARRMLGLQTQTVDSSQGSEFDYVIYCQTSDTAHALNVNRFNVAITRAKKGILCVMSDSTLYESLEFTPLDVNDYVKPK-MQS-EVTVGLFKDCAKA-E-PLGPAYAPTFVSVND
Hedgehog-CoV AVFISPYNSQNAVARSMLGLNTQTVDSSQGSEYPYVIFCQTADTAHANNINRFNVAVTRAQKGILCVMTSQALFDSLQFSELSVNN---YK-LQS-QIVTGLYKDCSREMS-GLSPAYAPTYMSVDE
MERS-CoV AVFISPYNSQNAVSRSMLGLTTQTVDSSQGSEYQYVIFCQTADTAHANNINRFNVAITRAQKGILCVMTSQALFESLEFTELSFTN---YK-LQS-QIVTGLFKDCSRETS-GLSPAYAPTYVSVDD
MHV AVFISPYNSQNYVAKRVLGLQTQTVDSAQGSEYDFVIYSQTAETAHSVNVNRFNVAITRAKKGILCVMSSMQLFESLNFTTLTLDKINN---PRL-QCTTNLFKDCSRSYV-GYHPAHAPSFLAVDD
OC43 AVFISPYNSQNFAAKRVLGLQTQTVDSAQGSEYDYVIYSQTAETAHSVNVNRFNVAITRAKKGILCVMSNMQLFEALQFTTLTLDKVPQAVETKV-QCSTNLFKDCSKSYS-GYHPAHAPSFLAVDD
Rat-CoV AVFISPYNSQNYVAKRVLGLQTQTVDSAQGSEYDFVIYSQTAETAHSVNVNRFNVAITRAKKGILCVMSSMQLFESLNFTTLTLDKINN---PRL-QCTTNLFKDCSKSYD-GYHPAHAPSFLAVDD
SARS-CoV AVFISPYNSQNAVASKILGLPTQTVDSSQGSEYDYVIFTQTTETAHSCNVNRFNVAITRAKIGILCIMSDRDLYDKLQFTSLEIPRRNVAT-LQA-ENVTGLFKDCSKIIT-GLHPTQAPTHLSVDI
229E AVFISPYNSQNYVAARLLGLQTQTVDSAQGSEYDYVIFAQTSDTAHACNANRFNVAITRAKKGIFCIMSDRTLFDALKFFEVTMT--D----LQS-ESSCGLFKDCARNPI-DLPPSHATTYLSLSD
Bat-CoV-1A AVFITPYNSQNYVASRVLGLQIQTVDSSQGSEYDYVIYTQTSDTAHACNVNRFNVAITRAKKGILCVMCDKALYDSLKFFEIQLT--D----LQS-GDLCGLFKDCSRVEE-PLPPAYAPTYVSLSD
BtCoV512 AVFISPYNSQNYVAGRVLGLQIQTVDSSQGSEYDYVIYTQTSDTAHASNVNRFNVAITRAKKGILCIMCDRELFDILKFYELKLS--D----LQV-GDGCGLFKDCYKGED-NLPPSHAPTFMSLSD
BtMr-AlphaCoV AVFISPYNSQNYVAARVLGLQIQTVDSSQGSEYDYVIYTQTSDTLHACNVNRFNVAITRAKKGIFCVMCDRTLYDSLKFFEVKLS--D----LQA-DNGCGLFKNCIRNEV-ILSPAYAPTYMALSD
BtNv-AlphaCoV AVFISPYNSQNYVASRVLGLQTQTVDSSQGSEYDYVIYTQTSDTLHACNINRFNVAITRAKKGILCVMCDRSLFDALKFYELKMT--D----LQS-SDGCGLFKDCHRGSD-NLPPSHAPTYMSLSD
BtRf-AlphaCoV AVFISPYNSQNYVASRALGLQIQTVDSSQGSEYDYVIYTQTSDTAHACNVNRFNVAITRAKKGILCVMCGKELFDALKFFEIKLT--D----LQV-GEGCGLFKDCSRKPD-LLPPSHATTFMSLAD
CDPHE15 AVFISPYNSQNYVASRSLGLQIQTVDSSQGSEYDYVIFTQTSDTAHASNVNRFNVAITRAKKGILCVMCDKNLYDLLKFYELKLS--D----LQAQTDGCGLFKDCSRRDD-MLPPSHASTYMALSD
FCoV AVFISPYNSQNYVARRLLGLQTQTVDSAQGSEYDYVIYTQTSDTQHATNVNRFNVAITRAKVGILCIMCDRTMYENLDFYELKDSKIG----LQAKPETCGLFKDCSKSEQ-YIPPAYATTYMSLSD
Ferret-CoV ATFISPYNSQNYVARRLLGLQTQTVDSAQGSEYDYVIYAQTSDTAHAVNINRFNVAITRAKVGILCVMCDKPMYDGLNFYEITDV--N----LQSKGEGCGLFKDCSKAER-YIPPAYATTYISLSD
HKU10 AVFISPYNSQNYVASRALGLQIQTVDSSQGSEYDYVIFTQTSDTQHACNVNRFNVAITRAKKGILCIMCDKELFDALKFFEIKLT--D----LQA-GEGCGLFKDCSRKPD-LLPPSHATTFMSLSD
HKU2 AVFISPYNSQNYVAGRMLGLQTQTVDSAQGSEYDYVIYTQTSDTSHALNVNRFNVAITRTKIGILCIMCDKALYDTLKFFEISQS--D----LQSTIGGCGLFKDCYKFDQ-DLPPAHATTYMALSD
HKU8 AVFISPYNSQNYVASRSLGLQIQTVDSSQGSEYDYVIFAQTSDTAHACNVNRFNVAITRAKKGIFCIMCDKLLYEALKFFEVKLT--D----LQA-GDTCGLFKDCSKFDDPPLPPSHAPTYVALSD
LuchengRn-CoV AVFISPYNSQNYVAARVLGLQTQTVDSSQGSEYDYVIYTQTSDTAHALNVNRFNVAITRAKKGIFCVMNDANLFNALEFQEITTT--D----LQS--VLSGLFKNCIRKGH-DLPPNHARSFLDLDS
Mink-CoV ATFISPYNSQNYVARRLLGLQTQTVDSAQGSEYDFVVYTQTSDTAHAVNINRFNVAITRAKIGILCVMCDKAMYDGLNFYEIKDV--N----LQVKSEGCGLFKDCAKVEH-YIPPAYATTYISLSD
NL63 AVFISPYNSQNYVASRFLGLQIQTVDSSQGSEYDYVIYAQTSDTAHACNVNRFNVAITRAKKGIFCVMCDKTLFDSLKFFEIKHA--D----LHS-SQVCGLFKNCTRTPL-NLPPTHAHTFLSLSD
NL63-related AVFISPYNSQNYVASRLLGLQIQTVDSSQGSEYEYVIYAQTSDTAHACNVNRFNVAITRAKKGIFCIMCDRELFEALKFFEIKQS--D----LQSVDQACGLFKDCARNPV-DLPPTHAHTFLSLSD
Porcine-EDV AVFISPYNSQNYVASRLLGLQIQTVDSSQGSEYDYVIYAQTSDTAHASNVNRFNVAITRAKKGILCIMCDRSLFDLLKFFELKLS--D----LQA-NEGCGLFKDCSRGDD-LLPPSHANTFMSLAD
TGE AVFISPYNSQNYVARRLLGLQTQTVDSAQGSEYDYVIYTQTSDTQHATNVNRFNVAITRAKVGILCIMCDRTMYENLDFYELKDSKIG----LQAKPETCGLFKDCSKSEQ-YIPPAYATTYMSLSD
Beta-CoV cons
**********588558**96******8****959**97**79***86*7******9***75**8*9*77339994*5*5383754------075-23587*9***89303-562*85**8788*83
Alpha-CoV cons
*8**8*********4*4****7*****8*****99*998*****5**5*7*********8*5**8*9*7542997 5*5*7*9526--5----*66-0357****8*29232-2*+7*78*28985*57
Combined cons
*8**+******68+455**+67*****8****969*+97**79*5*85*7******9**+75**8*9*77449993*5*539443--1----075-34587*9*8*49101-172*76*38788959
Alignment qual

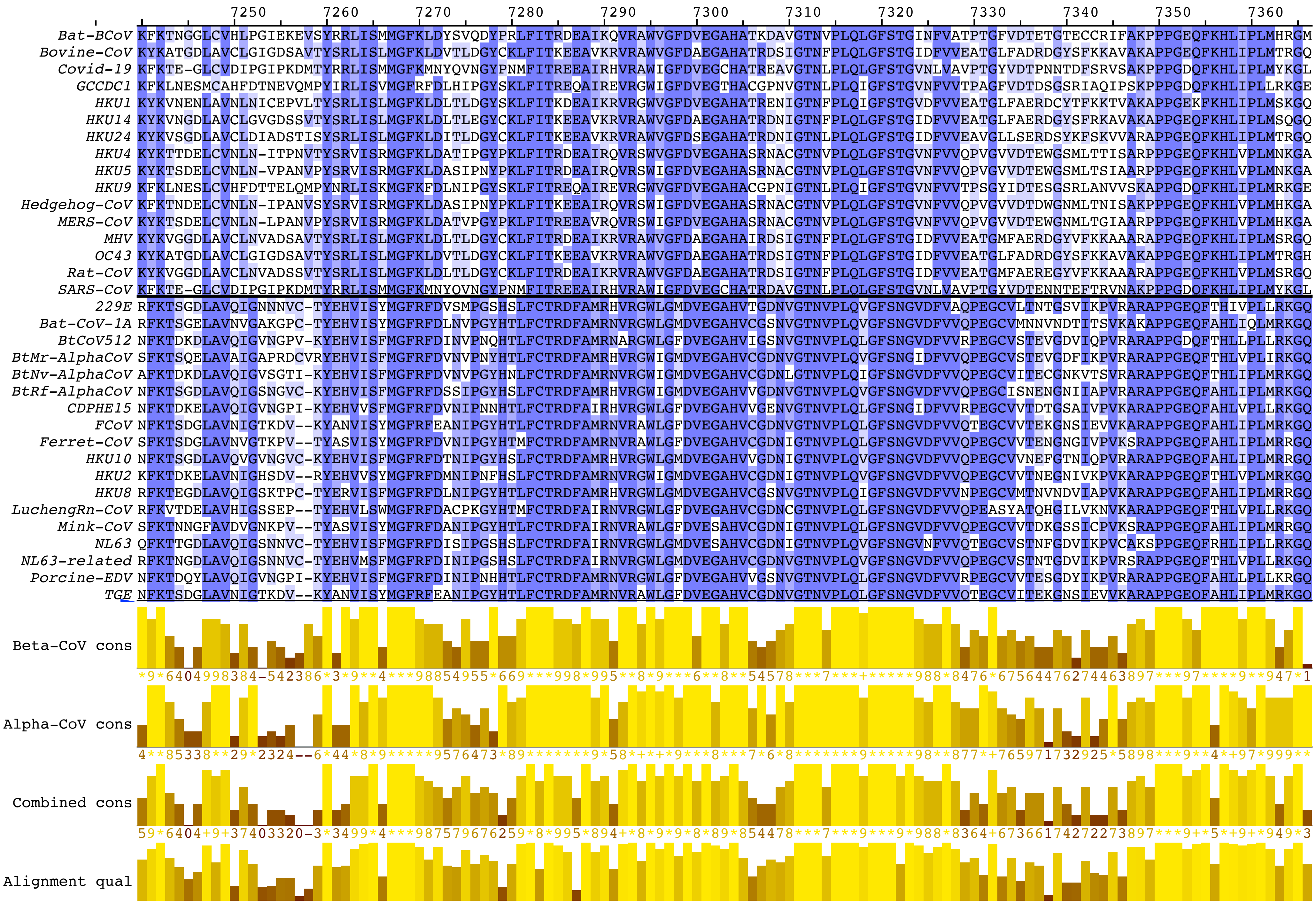

7250
7260
7270
7280
7290
7300
7310
7320
7330
7340
7350
7360
Bat-BCoV
Bovine-CoV
Covid-19
GCCDC1
HKU1
HKU14
HKU24
HKU4
HKU5
HKU9
Hedgehog-CoV
MERS-CoV
MHV
OC43
Rat-CoV
SARS-CoV
229E
Bat-CoV-1A
BtCoV512
BtMr-AlphaCoV
BtNv-AlphaCoV
BtRf-AlphaCoV
CDPHE15
FCoV
Ferret-CoV
HKU10
HKU2
HKU8
LuchengRn-CoV
Mink-CoV
NL63
NL63-related
Porcine-EDV
TGE
Beta-CoV cons
Alpha-CoV cons
Combined cons
Alignment qual

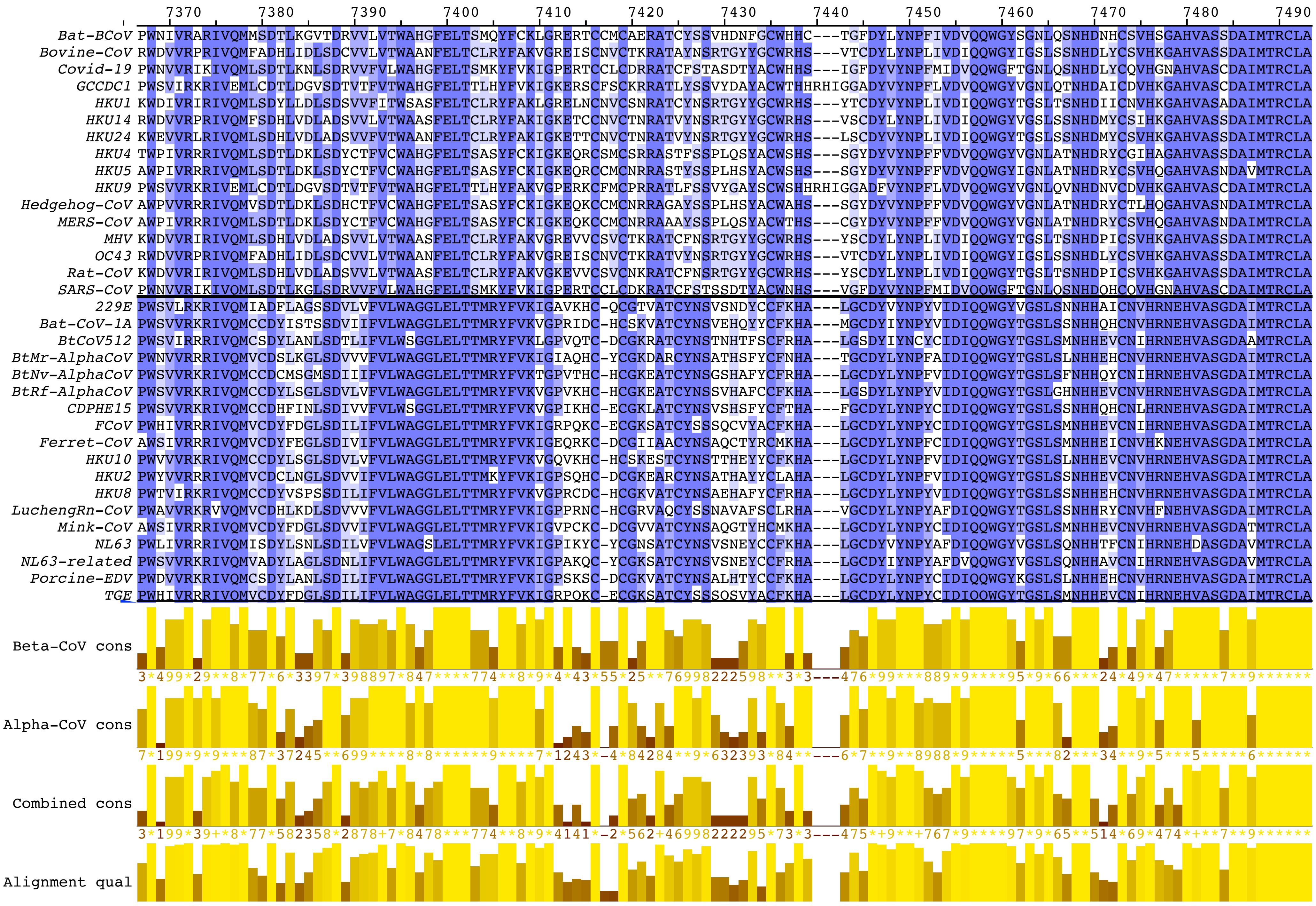
7370 7380 7390 7400 7410 7420 7430 7440 7450 7460 7470 7480 7490
Bat-BCoV
Bovine-CoV
Covid-19
GCCDC1
HKU1
HKU14
HKU24
HKU4
HKU5
HKU9
Hedgehog-CoV
MERS-CoV
MHV
OC43
Rat-CoV
SARS-CoV
229E
Bat-CoV-1A
BtCoV512
BtMr-AlphaCoV
BtNv-AlphaCoV
BtRf-AlphaCoV
CDPHE15
FCoV
Ferret-CoV
HKU10
HKU2
HKU8
LuchengRn-CoV
Mink-CoV
NL63
NL63-related
Porcine-EDV
TGE
Beta-CoV cons
Alpha-CoV cons
Combined cons
Alignment qual

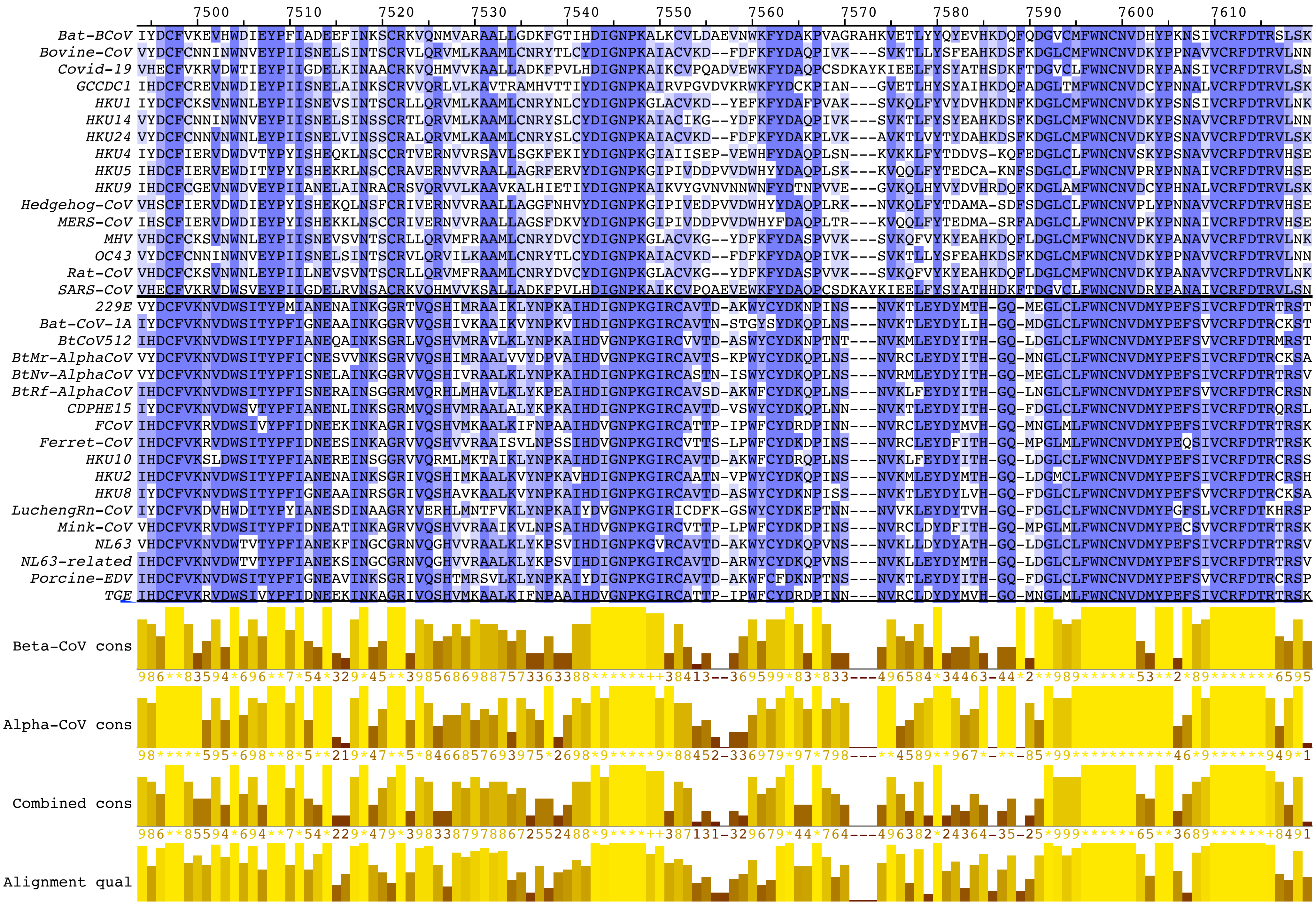
7500 7510 7520 7530 7540 7550 7560 7570 7580 7590 7600 7610
Bat-BCoV IYDCFVKEVHWDIEYPFIADEEFINKSCRKVQNMVARAALLGDKFGTIHDIGNPKALKCVLDAEVNWKFYDAKPVAGRAHKVETLYYQYEVHKDQFQDGVCMFWNCNVDHYPKNSIVCRFDTRSLSK
Bovine-CoV VYDCFCNNINWNVEYPIISNELSINTSCRVLQRVMLKAAMLCNRYTLCYDIGNPKAIACVKD--FDFKFYDAQPIVK---SVKTLLYSFEAHKDSFKDGLCMFWNCNVDKYPPNAVVCRFDTRVLNN
Covid-19 VHECFVKRVDWTIEYPIIGDELKINAACRKVQHMVVKAALLADKFPVLHDIGNPKAIKCVPQADVEWKFYDAQPCSDKAYKIEELFYSYATHSDKFTDGVCLFWNCNVDRYPANSIVCRFDTRVLSN
GCCDC1 IHDCFCREVNWDIEYPIISNELAINKSCRVVQRLVLKAVTRAMHVTTIYDIGNPKAIRVPGVDVKRWKFYDCKPIAN---GVETLHYSYAIHKDQFADGLTMFWNCNVDCYPNNALVCRFDTRVLSK
HKU1 IYDCFCKSVNWNLEYPIISNEVSINTSCRLLQRVMLKAAMLCNRYNLCYDIGNPKGLACVKD--YEFKFYDAFPVAK---SVKQLFYVYDVHKDNFKDGLCMFWNCNVDKYPSNSIVCRFDTRVLNK
HKU14 VYDCFCNNINWNVEYPIISNELSINSSCRTLQRVMLKAAMLCNRYSLCYDIGNPKAIACIKG--YDFKFYDAQPIVK---SVKTLFYSYEAHKDSFKDGLCMFWNCNVDKYPSNAVVCRFDTRVLNN
HKU24 VYDCFCNNVNWNLEYPIISNELVINSSCRALQRVMLKAAMLCNRYSLCYDIGNPKAIACVKD--FDFKFYDAKPLVK---AVKTLVYTYDAHKDSFKDGLCMFWNCNVDKYPSNAVVCRFDTRVLSK
HKU4 IYDCFIERVDWDVTYPYISHEQKLNSCCRTVERNVVRSAVLSGKFEKIYDIGNPKGIAIISEP-VEWHFYDAQPLSN---KVKKLFYTDDVS-KQFEDGLCLFWNCNVSKYPSNAVVCRFDTRVHSE
HKU5 IHDCFIERVEWDITYPYISHEKRLNSCCRAVERNVVRAALLAGRFERVYDIGNPKGIPIVDDPVVDWHYYDAQPLSK---KVQQLFYTEDCA-KNFSDGLCLFWNCNVPRYPNNAIVCRFDTRVHSE
HKU9 IHDCFCGEVNWDVEYPIIANELAINRACRSVQRVVLKAAVKALHIETIYDIGNPKAIKVYGVNVNNWNFYDTNPVVE---GVKQLHYVYDVHRDQFKDGLAMFWNCNVDCYPHNALVCRFDTRVLSK
Hedgehog-CoV VHSCFIERVDWDIEYPYISHEKQLNSFCRIVERNVVRAALLAGGFNHVYDIGNPKGIPIVEDPVVDWHYYDAQPLRK---NVKQLFYTDAMA-SDFSDGLCLFWNCNVPLYPNNAVVCRFDTRVHSE
MERS-CoV IHSCFIERVDWDIEYPYISHEKKLNSCCRIVERNVVRAALLAGSFDKVYDIGNPKGIPIVDDPVVDWHYFDAQPLTR---KVQQLFYTEDMA-SRFADGLCLFWNCNVPKYPNNAIVCRFDTRVHSE
MHV VHDCFCKSVNWNLEYPIISNEVSVNTSCRLLQRVMFRAAMLCNRYDVCYDIGNPKGLACVKG--YDFKFYDASPVVK---SVKQFVYKYEAHKDQFLDGLCMFWNCNVDKYPANAVVCRFDTRVLNK
OC43 VYDCFCNNINWNVEYPIISNELSINTSCRVLQRVILKAAMLCNRYTLCYDIGNPKAIACVKD--FDFKFYDAQPIVK---SVKTLLYSFEAHKDSFKDGLCMFWNCNVDKYPPNAVVCRFDTRVLNN
Rat-CoV VHDCFCKSVNWNLEYPIILNEVSVNTSCRLLQRVMFRAAMLCNRYDVCYDIGNPKGLACVKG--YDFKFYDASPVVK---SVKQFVYKYEAHKDQFLDGLCMFWNCNVDKYPANAVVCRFDTRVLNK
SARS-CoV VHECFVKRVDWSVEYPIIGDELRVNSACRKVQHMVVKSALLADKFPVLHDIGNPKAIKCVPQAEVEWKFYDAQPCSDKAYKIEELFYSYATHHDKFTDGVCLFWNCNVDRYPANAIVCRFDTRVLSN
229E VYDCFVKNVDWSITYPMIANENAINKGGRTVQSHIMRAAIKLYNPKAIHDIGNPKGIRCAVTD-AKWYCYDKNPINS---NVKTLEYDYMTH-GQ-MEGLCLFWNCNVDMYPEFSIVCRFDTRTRST
Bat-CoV-1A IYDCFVKNVDWSITYPFIGNEAAINKGGRVVQSHIVKAAIKVYNPKVIHDIGNPKGIRCAVTN-STGYSYDKQPLNS---NVKTLEYDYLIH-GQ-MDGLCLFWNCNVDMYPEFSVVCRFDTRCKST
BtCoV512 IHDCFVKNVDWSITYPFIANEQAINKSGRLVQSHVMRAVLKLYNPKAIHDVGNPKGIRCVVTD-ASWYCYDKNPTNT---NVKMLEYDYITH-GQ-LDGLCLFWNCNVDMYPEFSVVCRFDTRMRST
BtMr-AlphaCoV VYDCFVKNVDWSITYPFICNESVVNKSGRVVQSHIMRAALVVYDPVAIHDIGNPKGIRCAVTS-KPWYCYDKQPLNS---NVRCLEYDYITH-GQ-MNGLCLFWNCNVDMYPEFSIVCRFDTRCKSA
BtNv-AlphaCoV VYDCFVKNVDWSITYPFISNELAINKGGRVVQSHIVRAALKLYNPKAIHDIGNPKGIRCASTN-ISWYCYDKQPLNS---NVRMLEYDYITH-GQ-MEGLCLFWNCNVDMYPEFSIVCRFDTRTRSV
BtRf-AlphaCoV IHDCFVKNVDWSITYPFISNERAINSGGRMVQRHLMHAVLKLYKPEAIHDIGNPKGIRCAVSD-AKWFCYDKQPLNS---NVKLFEYDYITH-GQ-LNGLCLFWNCNVDMYPEFSVVCRFDTRCRSN
CDPHE15 IYDCFVKNVDWSVTYPFIANENLINKSGRMVQSHVMRAALALYKPKAIHDIGNPKGIRCAVTD-VSWYCYDKQPLNN---NVKTLEYDYITH-GQ-FDGLCLFWNCNVDMYPEFSIVCRFDTRQRSL
FCoV IHDCFVKRVDWSIVYPFIDNEEKINKAGRIVQSHVMKAALKIFNPAAIHDVGNPKGIRCATTP-IPWFCYDRDPINN---NVRCLEYDYMVH-GQ-MNGLMLFWNCNVDMYPEFSIVCRFDTRTRSK
Ferret-CoV IHDCFVKRVDWSITYPFIDNEESINKAGRVVQSHVVRAAISVLNPSSIHDVGNPKGIRCVTTS-LPWFCYDKDPINS---NVRCLEYDFITH-GQ-MPGLMLFWNCNVDMYPEQSIVCRFDTRTRSK
HKU10 IHDCFVKSLDWSITYPFIANEREINSGGRVVQRMLMKTAIKLYNPKAIHDIGNPKGIRCAVTD-AKWFCYDRQPLNS---NVKLFEYDYITH-GQ-LDGLCLFWNCNVDMYPEFSIVCRFDTRCRSS
HKU2 IHDCFVKNVDWSITYPFIANENAINKSGRIVQSHIMKAALKVYNPKAVHDIGNPKGIRCAATN-VPWYCYDKQPINS---NVKMLEYDYMTH-GQ-LDGMCLFWNCNVDMYPEFSIVCRFDTRCRSH
HKU8 IYDCFVKNVDWSITYPFIGNEAAINRSGRIVQSHAVKAALKVYNPKAIHDIGNPKGIRCAVTD-ASWYCYDKNPISS---NVKTLEYDYLVH-GQ-FDGLCLFWNCNVDMYPEFSVVCRFDTRCKSA
LuchengRn-CoV IYDCFVKDVHWDITYPYIANESDINAAGRYVERHLMNTFVKLYNPKAIYDVGNPKGIRICDFK-GSWYCYDKEPTNN---NVVKLEYDYTVH-GQ-FDGLCLFWNCNVDMYPGFSLVCRFDTKHRSP
Mink-CoV VHDCFVKRVDWSITYPFIDNEATINKAGRVVQSHVVRAAIKVLNPSAIHDIGNPKGIRCVTTP-LPWFCYDKDPINS---NVRCLDYDFITH-GQ-MPGLMLFWNCNVDMYPECSVVCRFDTRTRSK
NL63 VHDCFVKNVDWTVTYPFIANEKFINGCGRNVQGHVVRAALKLYKPSVIHDIGNPKGVRCAVTD-AKWYCYDKQPVNS---NVKLLDYDYATH-GQ-LDGLCLFWNCNVDMYPEFSIVCRFDTRTRSV
NL63-related IHDCFVKNVDWTVTYPFIANEKSINGCGRNVQGHVVRAALKLYKPSVIHDIGNPKGIRCAVTD-ARWYCYDKQPVNS---NVKLLEYDYMTH-GQ-LDGLCLFWNCNVDMYPEFSIVCRFDTRTRSV
Porcine-EDV IHDCFVKNVDWSITYPFIGNEAVINKSGRIVQSHTMRSVLKLYNPKAIYDIGNPKGIRCAVTD-AKWFCFDKNPTNS---NVKTLEYDYITH-GQ-FDGLCLFWNCNVDMYPEFSVVCRFDTRCRSP
TGE IHDCFVKRVDWSIVYPFIDNEEKINKAGRIVQSHVMKAALKIFNPAAIHDVGNPKGIRCATTP-IPWFCYDRDPINN---NVRCLDYDYMVH-GQ-MNGLMLFWNCNVDMYPEFSIVCRFDTRTRSK
Beta-CoV cons
986**83594*696**7*54*329*45**3985686988757336338 8*****++38413--369599*83*833---496584*34463-44*2**989*****53**2*89******6595
Alpha-CoV cons
98*****595*698**8*5**219*47**5*8466857693975*2698*9*****9*88452-336979*97*798---**4589**967*-**-85*99**********46*9******949*1
Combined cons
986**85594*694**7*54*229*479*3983387978867255248 8*9****++387131-329679*44*764---496382*24364-35-25*999*****65**3689******+8491
Alignment qual

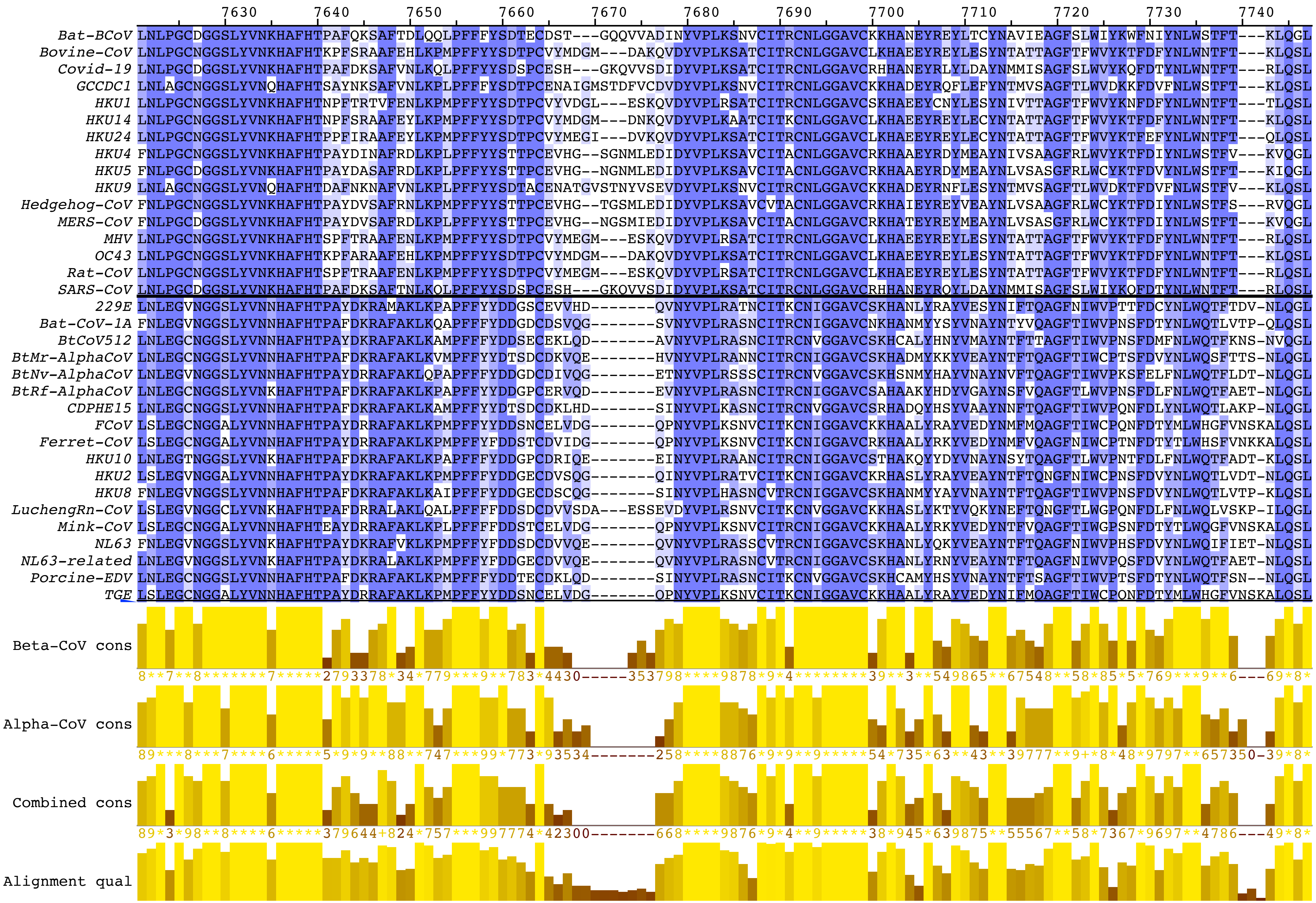
7630
7640
7650
7660
7670
7680
7690
7700
7710
7720
7730
7740
Bat-BCoV
Bovine-CoV
Covid-19
GCCDC1
HKU1
HKU14
HKU24
HKU4
HKU5
HKU9
Hedgehog-CoV
MERS-CoV
MHV
OC43
Rat-CoV
SARS-CoV
229E
Bat-CoV-1A
BtCoV512
BtMr-AlphaCoV
BtNv-AlphaCoV
BtRf-AlphaCoV
CDPHE15
FCoV
Ferret-CoV
HKU10
HKU2
HKU8
LuchengRn-CoV
Mink-CoV
NL63
NL63-related
Porcine-EDV
TGE
Beta-CoV cons
Alpha-CoV cons
Combined cons
Alignment qual

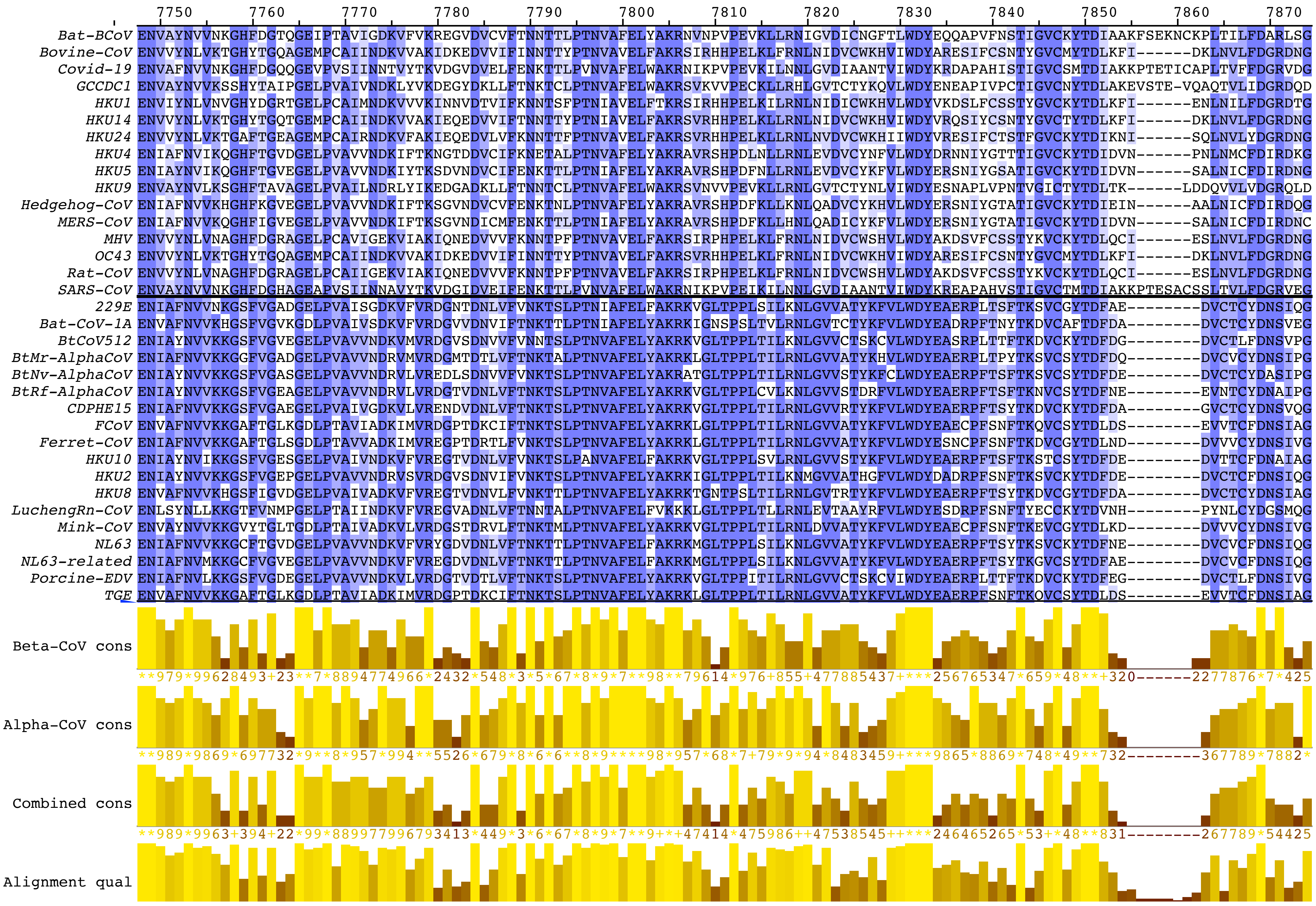

7750 7760 7770 7780 7790 7800 7810 7820 7830 7840 7850 7860 7870
Bat-BCoV ENVAYNVVNKGHFDGTQGEIPTAVIGDKVFVKREGVDVCVFTNNTTLPTNVAFELYAKRNVNPVPEVKLLRNIGVDICNGFTLWDYEQQAPVFNSTIGVCKYTDIAAKFSEKNCKPLTILFDARLSG
Bovine-CoV ENVVYNLVKTGHYTGQAGEMPCAIINDKVVAKIDKEDVVIFINNTTYPTNVAVELFAKRSIRHHPELKLFRNLNIDVCWKHVIWDYARESIFCSNTYGVCMYTDLKFI------DKLNVLFDGRDNG
Covid-19 ENVAFNVVNKGHFDGQQGEVPVSIINNTVYTKVDGVDVELFENKTTLPVNVAFELWAKRNIKPVPEVKILNNLGVDIAANTVIWDYKRDAPAHISTIGVCSMTDIAKKPTETICAPLTVFFDGRVDG
GCCDC1 ENVAYNVVKSSHYTAIPGELPVAIVNDKLYVKDEGYDKLLFTNKTCLPTNVAFELWAKRSVKVVPECKLLRHLGVTCTYKQVLWDYENEAPIVPCTIGVCNYTDLAKEVSTE-VQAQTVLIDGRDQD
HKU1 ENVIYNLVNVGHYDGRTGELPCAIMNDKVVVKINNVDTVIFKNNTSFPTNIAVELFTKRSIRHHPELKILRNLNIDICWKHVLWDYVKDSLFCSSTYGVCKYTDLKFI------ENLNILFDGRDTG
HKU14 ENVVYNLVKTGHYTGQTGEMPCAIINDKVVAKIEQEDVVIFTNNTTYPTNIAVELFAKRSVRHHPELKLLRNLNIDVCWKHVIWDYVRQSIYCSNTYGVCTYTDLKFI------DKLNVLFDGRDNG
HKU24 ENVVYNLVKTGAFTGEAGEMPCAIRNDKVFAKIEQEDVLVFKNNTTFPTNVAVELFAKRSVRHHPELKLLRNLNVDVCWKHIIWDYVRESIFCTSTFGVCKYTDIKNI------SQLNVLYDGRDNG
HKU4 ENIAFNVIKQGHFTGVDGELPVAVVNDKIFTKNGTDDVCIFKNETALPTNVAFELYAKRAVRSHPELNLLRNLEVDVCYNFVLWDYDRNNIYGTTTIGVCKYTDIDVN------PNLNMCFDIRDKG
HKU5 ENIAYNVIKQGHFTGVEGELPVAVVNDKIYTKSDVNDVCIFENKTTLPTNIAFELYAKRAVRSHPDFNLLRNLEVDVCYKFVLWDYERSNIYGSATIGVCKYTDIDVN------SALNICFDIRDNG
HKU9 ENVAYNVLKSGHFTAVAGELPVAILNDRLYIKEDGADKLLFTNNTCLPTNVAFELWAKRSVNVVPEVKLLRNLGVTCTYNLVIWDYESNAPLVPNTVGICTYTDLTK------LDDQVVLVDGRQLD
Hedgehog-CoV ENIAFNVVKHGHFKGVEGELPVAVVNDKIFTKSGVNDVCVFENKTNLPTNVAFELYAKRAVRSHPDFKLLKNLQADVCYKHVLWDYERSNIYGTATIGVCKYTDIEIN------AALNICFDIRDQG
MERS-CoV ENIAFNVVKQGHFIGVEGELPVAVVNDKIFTKSGVNDICMFENKTTLPTNIAFELYAKRAVRSHPDFKLLHNLQADICYKFVLWDYERSNIYGTATIGVCKYTDIDVN------SALNICFDIRDNC
MHV ENVVYNLVNAGHFDGRAGELPCAVIGEKVIAKIQNEDVVVFKNNTPFPTNVAVELFAKRSIRPHPELKLFRNLNIDVCWSHVLWDYAKDSVFCSSTYKVCKYTDLQCI------ESLNVLFDGRDNG
OC43 ENVVYNLVKTGHYTGQAGEMPCAIINDKVVAKIDKEDVVIFINNTTYPTNVAVELFAKRSVRHHPELKLFRNLNIDVCWKHVIWDYARESIFCSNTYGVCMYTDLKFI------DKLNVLFDGRDNG
Rat-CoV ENVVYNLVNAGHFDGRAGELPCAIIGEKVIAKIQNEDVVVFKNNTPFPTNVAVELFAKRSIRPHPELKLFRNLNIDVCWSHVLWDYAKDSVFCSSTYKVCKYTDLQCI------ESLNVLFDGRDNG
SARS-CoV ENVAYNVVNKGHFDGHAGEAPVSIINNAVYTKVDGIDVEIFENKTTLPVNVAFELWAKRNIKPVPEIKILNNLGVDIAANTVIWDYKREAPAHVSTIGVCTMTDIAKKPTESACSSLTVLFDGRVEG
229E ENIAFNVVNKGSFVGADGELPVAISGDKVFVRDGNTDNLVFVNKTSLPTNIAFELFAKRKVGLTPPLSILKNLGVVATYKFVLWDYEAERPLTSFTKSVCGYTDFAE--------DVCTCYDNSIQG
Bat-CoV-1A ENVAFNVVKHGSFVGVKGDLPVAIVSDKVFVRDGVVDNVIFVNKTTLPTNIAFELYAKRKIGNSPSLTVLRNLGVTCTYKFVLWDYEADRPFTNYTKDVCAFTDFDA--------DVCTCYDNSVEG
BtCoV512 ENIAYNVVKKGSFVGVEGELPVAVVNDKVMVRDGVSDNVVFVNNTSLPTNVAFELYAKRKVGLTPPLTILKNLGVVCTSKCVLWDYEASRPLTTFTKDVCKYTDFDG--------DVCTLFDNSVPG
BtMr-AlphaCoV ENIAFNVVKKGGFVGADGELPVAVVNDRVMVRDGMTDTLVFTNKTALPTNVAFELYAKRKLGLTPPLTILRNLGVVATYKHVLWDYEAERPLTPYTKSVCSYTDFDQ--------DVCVCYDNSIPG
BtNv-AlphaCoV ENIAYNVVKKGSFVGASGELPVAVVNDRVLVREDLSDNVVFVNKTSLPTNVAFELYAKRATGLTPPLTILRNLGVVSTYKFCLWDYEAERPFTSFTKSVCSYTDFDE--------DVCTCYDASIPG
BtRf-AlphaCoV ENIAYNVVKKGSFVGEAGELPVAVVNDRVLVRDGTVDNLVFTNKTSLPTNVAFELYAKRKVGLTPPLCVLKNLGVVSTDRFVLWDYEAERPFTSFTKNVCSYTDFNE--------EVNTCYDNAIPG
CDPHE15 ENIAFNVVKKGSFVGAEGELPVAIVGDKVLVRENDVDNLVFTNKTSLPTNVAFELYAKRKVGLTPPLTILRNLGVVRTYKFVLWDYEAERPFTSYTKDVCKYTDFDA--------GVCTCYDNSVQG
FCoV ENVAFNVVKKGAFTGLKGDLPTAVIADKIMVRDGPTDKCIFTNKTSLPTNVAFELYAKRKLGLTPPLTILRNLGVVATYKFVLWDYEAECPFSNFTKQVCSYTDLDS--------EVVTCFDNSIAG
Ferret-CoV ENIAFNVVKKGAFTGLSGDLPTAVVADKIMVREGPTDRTLFVNKTSLPTNVAFELYAKRKLGLTPPLTILRNLGVVATYKFVLWDYESNCPFSNFTKDVCGYTDLND--------DVVVCYDNSIVG
HKU10 ENIAYNVIKKGSFVGESGELPVAIVNDKVFVREGTVDNLVFVNKTSLPANVAFELFAKRKVGLTPPLSVLRNLGVVSTYKFVLWDYEAERPFTSFTKSTCSYTDFDE--------DVTTCFDNAIAG
HKU2 ENIAYNVVKKGSFVGEPGELPVAVVNDRVSVRDGVSDNVIFVNKTSLPTNVAFELYAKRKIGLTPPLTILKNMGVVATHGFVLWDYDADRPFSNFTKSVCKYTDFDE--------DVCTCFDNSIQG
HKU8 ENVAFNVVKHGSFIGVDGELPVAIVADKVFVREGTVDNVLFVNKTTLPTNVAFELYAKRKTGNTPSLTILRNLGVTRTYKFVLWDYEAERPFTSYTKDVCGYTDFDA--------DVCTCYDNSIAG
LuchengRn-CoV ENLSYNLLKKGTFVNMPGELPTAIINDKVFVREGVADNLVFTNNTALPTNVAFELFVKKLGLTPPLTILRNLEVTAAYRFVLWDYESDRPFSNFTYECCKYTDVNH--------PYNLCYDGSMQG
Mink-CoV ENVAYNVVKKGVYTGLTGDLPTAIVADKVLVRDGSTDRVLFTNKTMLPTNVAFELYAKRKVGLTPPLTILRNLDVVATYKFVLWDYEAECPFSNFTKEVCGYTDLKD--------DVVVCYDNSIVG
NL63 ENIAFNVVKKGCFTGVDGELPVAVVNDKVFVRYGDVDNLVFTNKTTLPTNVAFELFAKRKMGLTPPLSILKNLGVVATYKFVLWDYEAERPFTSYTKSVCKYTDFNE--------DVCVCFDNSIQG
NL63-related ENIAFNVMKKGCFVGVEGELPVAVVNDKVFVREGDVDNLVFTNKTTLPTNVAFELFAKRKMGLTPPLSILKNLGVVATYKFVLWDYEAERPFTSYTKGVCSYTDFAE--------DVCVCFDNSIQG
Porcine-EDV ENIAFNVLKKGSFVGDEGELPVAVVNDKVLVRDGTVDTLVFTNKTSLPTNVAFELYAKRKVGLTPPITILRNLGVVCTSKCVIWDYEAERPLTTFTKDVCKYTDFEG--------DVCTLFDNSIVG
TGE ENVAFNVVKKGAFTGLKGDLPTAVIADKIMVRDGPTDKCIFTNKTSLPTNVAFELYAKRKLGLTPPLTILRNLGVVATYKFVLWDYEAERPFSNFTKQVCSYTDLDS--------EVVTCFDNSIAG
Beta-CoV cons
979*99628493+237*8894774966*2432*548*3*5*67*8*9*7**98**79614*976*855+477885437+***256765347*659*48**+320------2277876*7*425
Alpha-CoV cons
989*9869*697732*98*957*994**5526*679*8*6*6**8*9****98*957*68*7+79*9*94*8483459+***9865*8869*748*49**732--------367789*7882*
Combined cons
989*9963+394+22*99*88997799679341 3*449*3*6*67*8*9*79+*+47414*475986++47538545++***246465265*53+*48**831--------267789*54425
Alignment qual

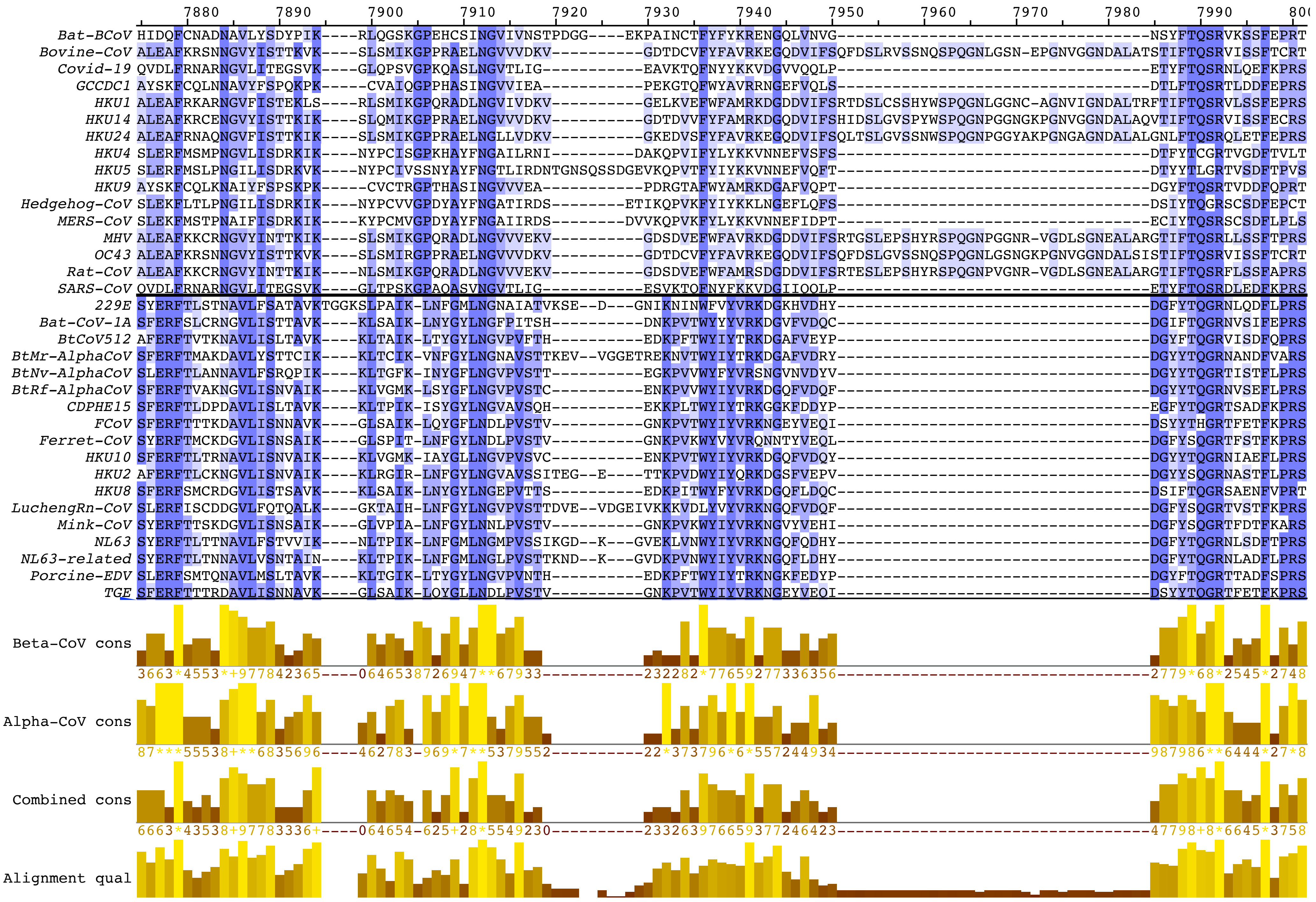

7880
7890
7900
7910
7920
7930
7940
7950
7960
7970
7980
7990
Bat-BCoV
Bovine-CoV
Covid-19
GCCDC1
HKU1
HKU14
HKU24
HKU4
HKU5
HKU9
Hedgehog-CoV
MERS-CoV
MHV
OC43
Rat-CoV
SARS-CoV
229E
Bat-CoV-1A
BtCoV512
BtMr-AlphaCoV
BtNv-AlphaCoV
BtRf-AlphaCoV
CDPHE15
FCoV
Ferret-CoV
HKU10
HKU2
HKU8
LuchengRn-CoV
Mink-CoV
NL63
NL63-related
Porcine-EDV
TGE
Beta-CoV cons
Alpha-CoV cons
Combined cons
Alignment qual

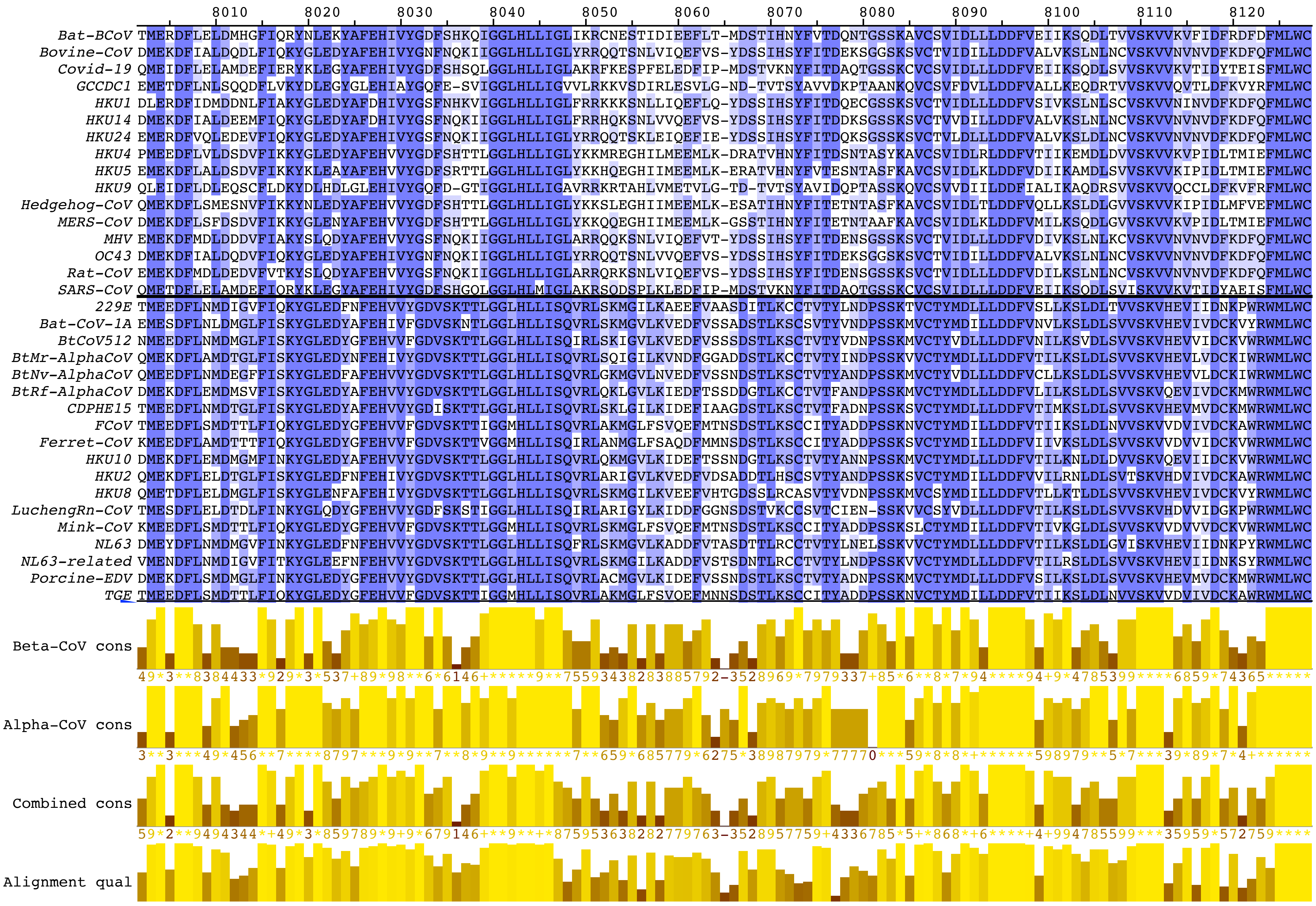

8010
8020
8030
8040
8050
8060
8070
8080
8090
8100
8110
8120
Bat-BCoV
Bovine-CoV
Covid-19
GCCDC1
HKU1
HKU14
HKU24
HKU4
HKU5
HKU9
Hedgehog-CoV
MERS-CoV
MHV
OC43
Rat-CoV
SARS-CoV
229E
Bat-CoV-1A
BtCoV512
BtMr-AlphaCoV
BtNv-AlphaCoV
BtRf-AlphaCoV
CDPHE15
FCoV
Ferret-CoV
HKU10
HKU2
HKU8
LuchengRn-CoV
Mink-CoV
NL63
NL63-related
Porcine-EDV
TGE
Beta-CoV cons
Alpha-CoV cons
Combined cons
Alignment qual

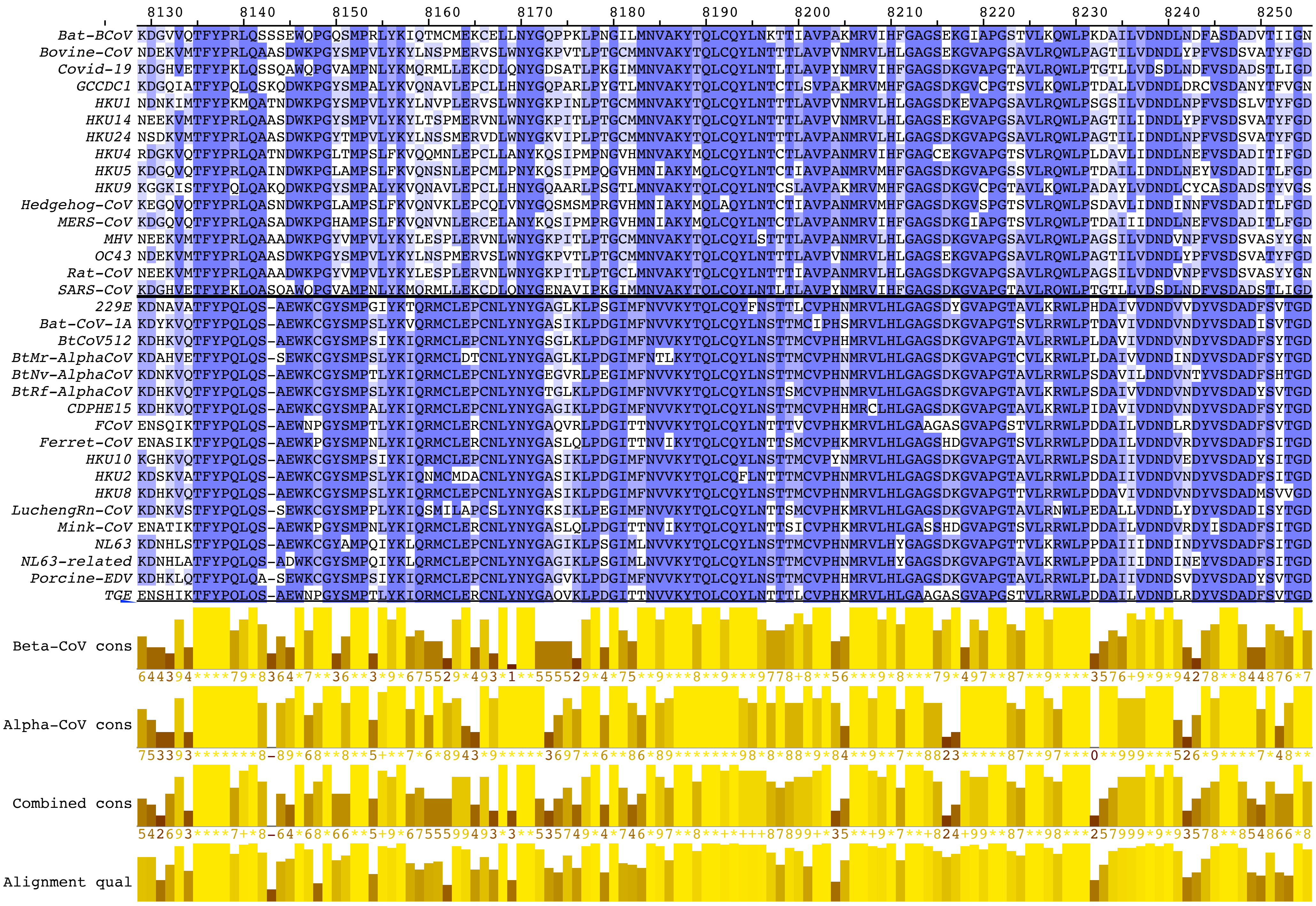
8130 8140 8150 8160 8170 8180 8190 8200 8210 8220 8230 8240 8250
Bat-BCoV KDGVVQTFYPRLQSSSEWQPGQSMPRLYKIQTMCMEKCELLNYGQPPKLPNGILMNVAKYTQLCQYLNKTTIAVPAKMRVIHFGAGSEKGIAPGSTVLKQWLPKDAILVDNDLNDFASDADVTIIGN
Bovine-CoV NDEKVMTFYPRLQAASDWKPGYSMPVLYKYLNSPMERVSLWNYGKPVTLPTGCMMNVAKYTQLCQYLNTTTLAVPVNMRVLHLGAGSEKGVAPGSAVLRQWLPAGTILVDNDLYPFVSDSVATYFGD
Covid-19 KDGHVETFYPKLQSSQAWQPGVAMPNLYKMQRMLLEKCDLQNYGDSATLPKGIMMNVAKYTQLCQYLNTLTLAVPYNMRVIHFGAGSDKGVAPGTAVLRQWLPTGTLLVDSDLNDFVSDADSTLIGD
GCCDC1 KDGQIATFYPQLQSKQDWKPGYSMPALYKVQNAVLEPCLLHNYGQPARLPYGTLMNVAKYTQLCQYLNTCTLSVPAKMRVMHFGAGSDKGVCPGTSVLKQWLPTDALLVDNDLDRCVSDANYTFVGN
HKU1 NDNKIMTFYPKMQATNDWKPGYSMPVLYKYLNVPLERVSLWNYGKPINLPTGCMMNVAKYTQLCQYLNTTTLAVPVNMRVLHLGAGSDKEVAPGSAVLRQWLPSGSILVDNDLNPFVSDSLVTYFGD
HKU14 NEEKVMTFYPRLQAASDWKPGYSMPVLYKYLTSPMERVNLWNYGKPITLPTGCMMNVAKYTQLCQYLNTTTLAVPVNMRVLHLGAGSEKGVAPGSAVLRQWLPAGTILIDNDLYPFVSDSVATYFGD
HKU24 NSDKVMTFYPRLQAASDWKPGYTMPVLYKYLNSSMERVDLWNYGKVIPLPTGCMMNVAKYTQLCQYLNTTTLAVPANMRVLHLGAGSDKGVAPGSAVLRQWLPAGTILIDNDLNPFVSDSVATYFGD
HKU4 RDGKVQTFYPRLQATNDWKPGLTMPSLFKVQQMNLEPCLLANYKQSIPMPNGVHMNVAKYMQLCQYLNTCTLAVPANMRVIHFGAGCEKGVAPGTSVLRQWLPLDAVLIDNDLNEFVSDADITIFGD
HKU5 KDGQVQTFYPRLQAINDWKPGLAMPSLFKVQNSNLEPCMLPNYKQSIPMPQGVHMNIAKYMQLCQYLNTCTIAVPANMRVMHFGAGSDKGVAPGSSVLRQWLPTDAILIDNDLNEYVSDADITLFGD
HKU9 KGGKISTFYPQLQAKQDWKPGYSMPALYKVQNAVLEPCLLHNYGQAARLPSGTLMNVAKYTQLCQYLNTCSLAVPAKMRVMHFGAGSDKGVCPGTAVLKQWLPADAYLVDNDLCYCASDADSTYVGS
Hedgehog-CoV KEGQVQTFYPRLQASNDWKPGLAMPSLFKVQNVKLEPCQLVNYGQSMSMPRGVHMNIAKYMQLAQYLNTCTIAVPANMRVMHFGAGSDKGVSPGTSVLRQWLPSDAVLIDNDINNFVSDADITLFGD
MERS-CoV KDGQVQTFYPRLQASADWKPGHAMPSLFKVQNVNLERCELANYKQSIPMPRGVHMNIAKYMQLCQYLNTCTLAVPANMRVIHFGAGSDKGIAPGTSVLRQWLPTDAIIIDNDLNEFVSDADITLFGD
MHV NEEKVMTFYPRLQAAADWKPGYVMPVLYKYLESPLERVNLWNYGKPITLPTGCMMNVAKYTQLCQYLSTTTLAVPANMRVLHLGAGSDKGVAPGSAVLRQWLPAGSILVDNDVNPFVSDSVASYYGN
OC43 NDEKVMTFYPRLQAASDWKPGYSMPVLYKYLNSPMERVSLWNYGKPVTLPTGCMMNVAKYTQLCQYLNTTTLAVPVNMRVLHLGAGSEKGVAPGSAVLRQWLPAGTILVDNDLYPFVSDSVATYFGD
Rat-CoV NEEKVMTFYPRLQAAADWKPGYVMPVLYKYLESPLERVNLWNYGKPITLPTGCLMNVAKYTQLCQYLNTTTIAVPANMRVLHLGAGSDKGVAPGSAVLRQWLPAGSILVDNDVNPFVSDSVASYYGN
SARS-CoV KDGHVETFYPKLQASQAWQPGVAMPNLYKMQRMLLEKCDLQNYGENAVIPKGIMMNVAKYTQLCQYLNTLTLAVPYNMRVIHFGAGSDKGVAPGTAVLRQWLPTGTLLVDSDLNDFVSDADSTLIGD
229E KDNAVATFYPQLQS-AEWKCGYSMPGIYKTQRMCLEPCNLYNYGAGLKLPSGIMFNVVKYTQLCQYFNSTTLCVPHNMRVLHLGAGSDYGVAPGTAVLKRWLPHDAIVVDNDVVDYVSDADFSVTGD
Bat-CoV-1A KDYKVQTFYPQLQS-AEWKCGYSMPSIYKVQRMCLEPCNLYNYGASIKLPDGIMFNVVKYTQLCQYLNSTTMCIPHSMRVLHLGAGSDKGVAPGTSVLRRWLPTDAVIVDNDVNDYVSDADISVTGD
BtCoV512 KDHKVQTFYPQLQS-AEWKCGYSMPSIYKIQRMCLEPCNLYNYGSGLKLPDGIMFNVVKYTQLCQYLNSTTMCVPHHMRVLHLGAGSDKGVAPGTAVLRRWLPLDAVIVDNDVNDYVSDADFSYTGD
BtMr-AlphaCoV KDAHVETFYPQLQS-SEWKCGYSMPSLYKIQRMCLDTCNLYNYGAGLKLPDGIMFNTLKYTQLCQYLNSTTMCVPHNMRVLHLGAGSDKGVAPGTCVLKRWLPLDAIVVDNDINDYVSDADFSYTGD
BtNv-AlphaCoV KDNKVQTFYPQLQS-AEWKCGYSMPTLYKIQRMCLEPCNLYNYGEGVRLPEGIMFNVVKYTQLCQYLNSTTMCVPHNMRVLHLGAGSDKGVAPGTAVLRRWLPSDAVILDNDVNTYVSDADFSHTGD
BtRf-AlphaCoV KDHKVQTFYPQLQS-AEWKCGYSMPSLYKIQRMCLEPCNLYNYGTGLKLPDGIMFNVVKYTQLCQYLNSTSMCVPHNMRVLHLGAGSDKGVAPGTAVLKRWLPSDAIVVDNDVNDYVSDADYSVTGD
CDPHE15 KDHKVQTFYPQLQS-AEWKCGYSMPALYKIQRMCLEPCNLYNYGAGIKLPDGIMFNVVKYTQLCQYLNSTTMCVPHHMRCLHLGAGSDKGVAPGTAVLKRWLPIDAVIVDNDVNDYVSDADFSYTGD
FCoV ENSQIKTFYPQLQS-AEWNPGYSMPTLYKIQRMCLERCNLYNYGAQVRLPDGITTNVVKYTQLCQYLNTTTVCVPHKMRVLHLGAAGASGVAPGSTVLRRWLPDDAILVDNDLRDYVSDADFSVTGD
Ferret-CoV ENASIKTFYPQLQS-AEWKPGYSMPNLYKIQRMCLERCNLYNYGASLQLPDGITTNVIKYTQLCQYLNTTSMCVPHKMRVLHLGAGSHDGVAPGTSVLRRWLPDDAILVDNDVRDYVSDADFSITGD
HKU10 KGHKVQTFYPQLQS-AEWKCGYSMPSIYKIQRMCLEPCNLYNYGASIKLPDGIMFNVVKYTQLCQYLNSTTMCVPYNMRVLHLGAGSDKGVAPGTAVLRRWLPSDAIIVDNDVEDYVSDADYSITGD
HKU2 KDSKVATFYPQLQS-AEWKCGYSMPSLYKIQNMCMDACNLYNYGASIKLPDGIMFNVVKYTQLCQFLNTTTMCVPHNMRVLHLGAGSDKGVAPGTAVLRRWLPDDAIIVDNDVNDYVSDADFSITGD
HKU8 KDHKVQTFYPQLQS-AEWKCGYSMPSLYKIQRMCLEPCNLYNYGASIKLPDGIMFNVVKYTQLCQYLNSTTMCVPHNMRVLHLGAGSDKGVAPGTTVLRRWLPDDAVIVDNDVNDYVSDADMSVVGD
LuchengRn-CoV KDNKVSTFYPQLQS-SEWKCGYSMPPLYKIQSMILAPCSLYNYGKSIKLPEGIMFNVVKYTQLCQYLNTTSMCVPHKMRVLHLGAGSDKGVAPGTAVLRNWLPEDALLVDNDLYDYVSDADISYTGD
Mink-CoV ENATIKTFYPQLQS-AEWKPGYSMPNLYKIQRMCLERCNLYNYGASLQLPDGITTNVIKYTQLCQYLNTTSICVPHKMRVLHLGASSHDGVAPGTSVLRRWLPDDAILVDNDVRDYISDADFSITGD
NL63 KDNHLSTFYPQLQS-AEWKCGYAMPQIYKLQRMCLEPCNLYNYGAGIKLPSGIMLNVVKYTQLCQYLNSTTMCVPHNMRVLHYGAGSDKGVAPGTTVLKRWLPPDAIIIDNDINDYVSDADFSITGD
NL63-related KDNHLATFYPQLQS-ADWKCGYSMPQIYKLQRMCLEPCNLYNYGAGIKLPSGIMLNVVKYTQLCQYLNSTTMCVPHNMRVLHYGAGSDKGVAPGTAVLKRWLPPDAIIIDNDINEYVSDADFSITGD
Porcine-EDV KDHKLQTFYPQLQA-SEWKCGYSMPSIYKIQRMCLEPCNLYNYGAGVKLPDGIMFNVVKYTQLCQYLNSTTMCVPHHMRVLHLGAGSDKGVAPGTAVLRRWLPLDAIIVDNDSVDYVSDADYSVTGD
TGE ENSHIKTFYPQLQS-AEWNPGYSMPTLYKIQRMCLERCNLYNYGAQVKLPDGITTNVVKYTQLCQYLNTTTLCVPHKMRVLHLGAAGASGVAPGSTVLRRWLPDDAILVDNDLRDYVSDADFSVTGD
Beta-CoV cons
644394****79*8364*7**36**3*9*67552 9*493*1**55552 9*4*75**9***8**9***9778+8**56***9*8***79*497**87**9****3576+9*9*94278**844876*7
Alpha-CoV cons
753393******8-89*68**8**5+**7*6*8943*9*****3697**6**86*89*******98*8*88*9*84**9**7**8823*****87**97***0**999***526*9****7*48**
Combined cons
542693****7+*8-64*68*66**5+9*67555994 93*3**535749*4*746*97**8**+*+++87899+*35**+9*7**+824+99**87**98***257999*9*93578**854866*8
Alignment qual

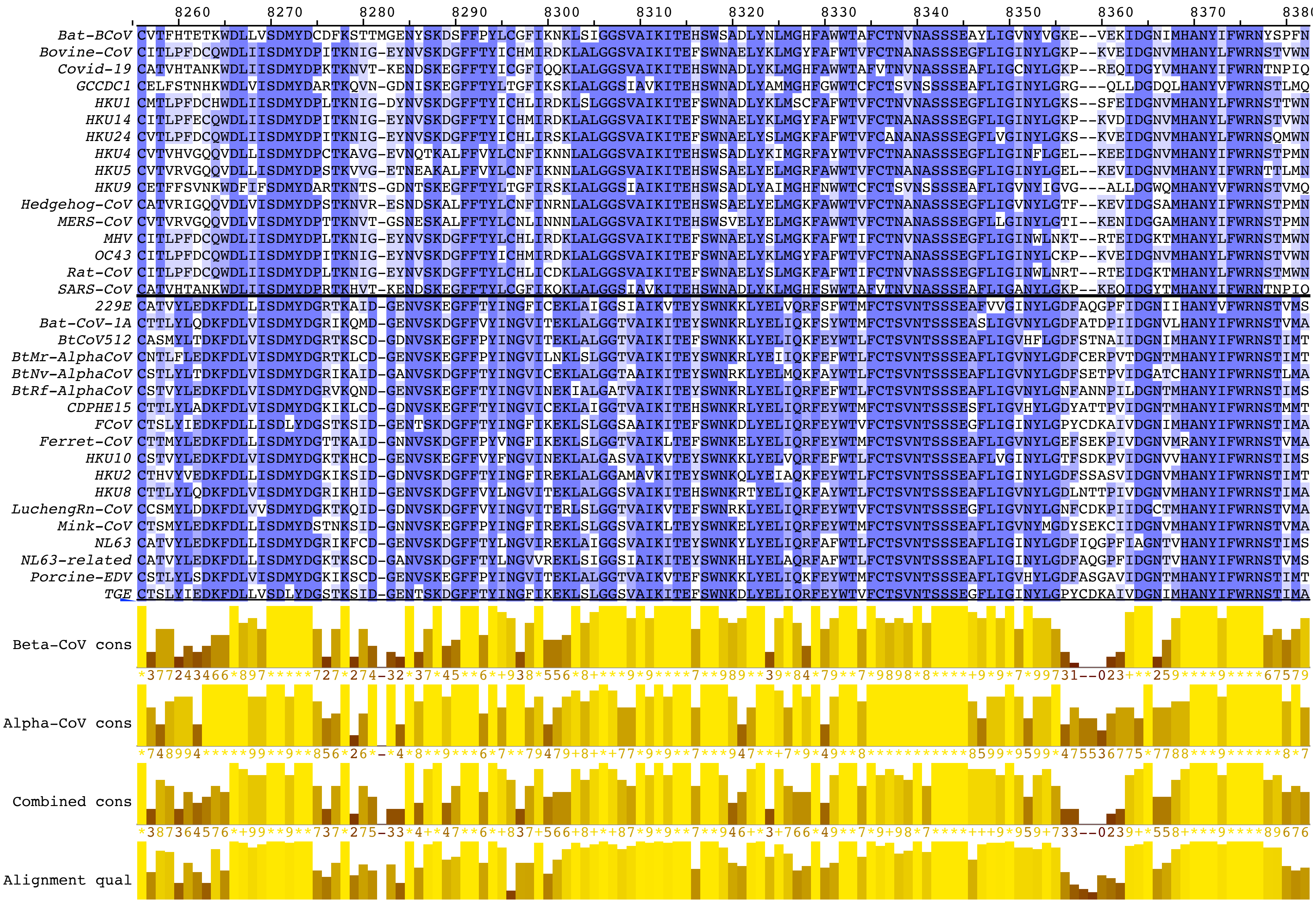

8260
8270
8280
8290
8300
8310
8320
8330
8340
8350
8360
8370
8380
Bat-BCoV
Bovine-CoV
Covid-19
GCCDC1
HKU1
HKU14
HKU24
HKU4
HKU5
HKU9
Hedgehog-CoV
MERS-CoV
MHV
OC43
Rat-CoV
SARS-CoV
229E
Bat-CoV-1A
BtCoV512
BtMr-AlphaCoV
BtNv-AlphaCoV
BtRf-AlphaCoV
CDPHE15
FCoV
Ferret-CoV
HKU10
HKU2
HKU8
LuchengRn-CoV
Mink-CoV
NL63
NL63-related
Porcine-EDV
TGE
Beta-CoV cons
Alpha-CoV cons
Combined cons
Alignment qual

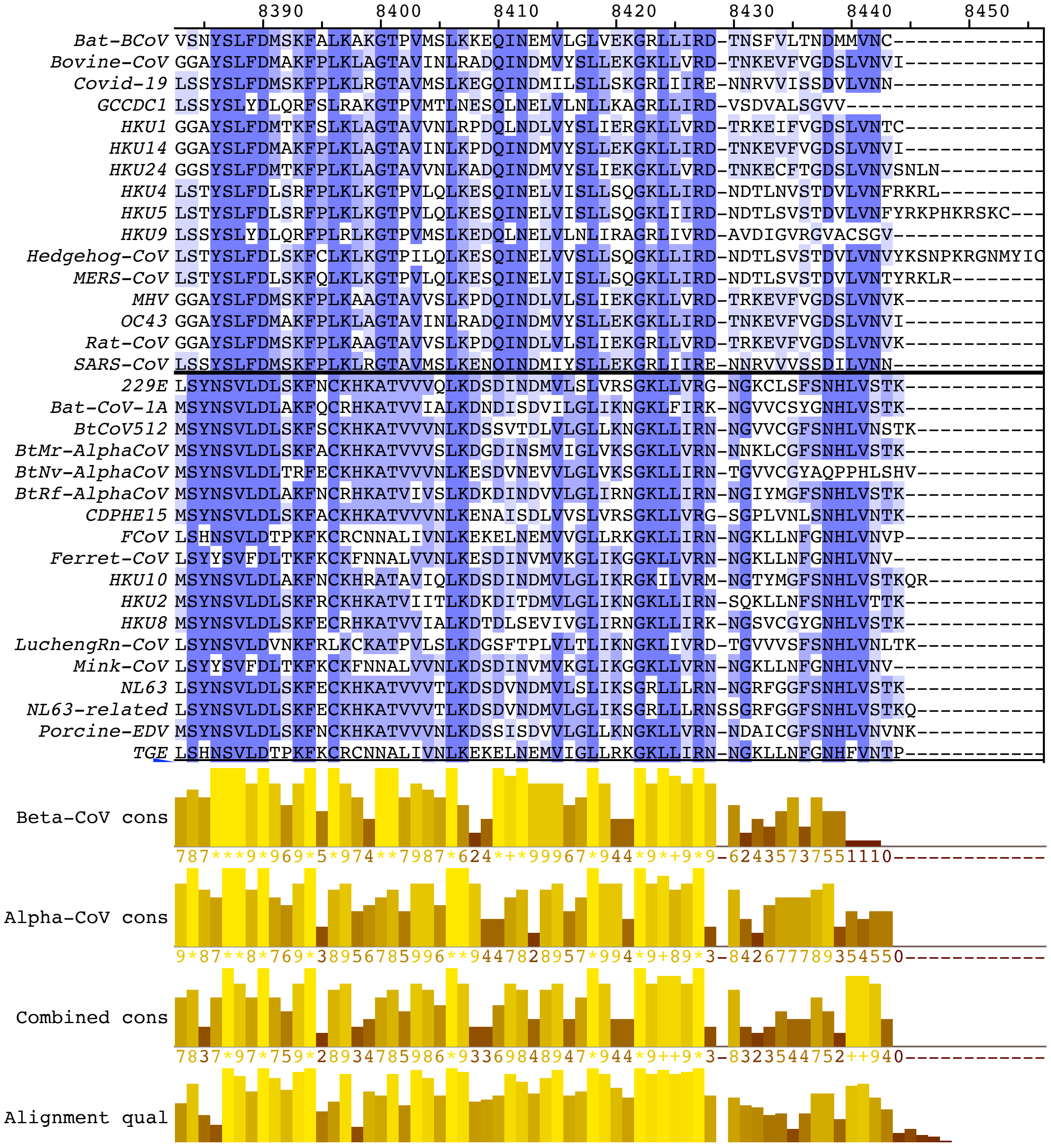
8390 8400 8410 8420 8430 8440 8450
Bat-BCoV VSNYSLFDMSKFALKAKGTPVMSLKKEQINEMVLGLVEKGRLLIRD-TNSFVLTNDMMVNC-------------
Bovine-CoV GGAYSLFDMAKFPLKLAGTAVINLRADQINDMVYSLLEKGKLLVRD-TNKEVFVGDSLVNVI------------
Covid-19 LSSYSLFDMSKFPLKLRGTAVMSLKEGQINDMILSLLSKGRLIIRE-NNRVVISSDVLVNN-------------
GCCDC1 LSSYSLYDLQRFSLRAKGTPVMTLNESQLNELVLNLLKAGRLLIRD-VSDVALSGVV----------------
HKU1 GGAYSLFDMTKFSLKLAGTAVVNLRPDQLNDLVYSLIERGKLLVRD-TRKEIFVGDSLVNTC------------
HKU14 GGAYSLFDMAKFPLKLAGTAVINLKPDQINDMVYSLLEKGKLLIRD-TNKEVFVGDSLVNVI------------
HKU24 GGSYSLFDMTKFPLKLAGTAVVNLKADQINDMVYSLIEKGKLLVRD-TNKECFTGDSLVNVSNLN---------
HKU4 LSTYSLFDLSRFPLKLKGTPVLQLKESQINELVISLLSQGKLLIRD-NDTLNVSTDVLVNFRKRL---------
HKU5 LSTYSLFDLSRFPLKLKGTPVLQLKESQINELVISLLSQGKLIIRD-NDTLSVSTDVLVNFYRKPHKRSKC---
HKU9 LSSYSLYDLQRFPLRLKGTPVMSLKEDQLNELVLNLIRAGRLIVRD-AVDIGVRGVACSGV-------------
Hedgehog-CoV LSTYSLFDLSKFCLKLKGTPILQLKESQINELVVSLLSQGKLLIRD-NDTLSVSTDVLVNVYKSNPKRGNMYIC
MERS-CoV LSTYSLFDLSKFQLKLKGTPVLQLKESQINELVISLLSQGKLLIRD-NDTLSVSTDVLVNTYRKLR--------
MHV GGAYSLFDMSKFPLKAAGTAVVSLKPDQINDLVLSLIEKGKLLVRD-TRKEVFVGDSLVNVK------------
OC43 GGAYSLFDMAKFPLKLAGTAVINLRADQINDMVYSLLEKGKLLIRD-TNKEVFVGDSLVNVI------------
Rat-CoV GGAYSLFDMSKFPLKAAGTAVVSLKPDQINDLVLSLIEKGRLLVRD-TRKEVFVGDSLVNVK------------
SARS-CoV LSSYSLFDMSKFPLKLRGTAVMSLKENQINDMIYSLLEKGRLIIRE-NNRVVVSSDILVNN-------------
229E LSYNSVLDLSKFNCKHKATVVVQLKDSDINDMVLSLVRSGKLLVRG-NGKCLSFSNHLVSTK------------
Bat-CoV-1A MSYNSVLDLAKFQCRHKATVVIALKDNDISDVILGLIKNGKLFIRK-NGVVCSYGNHLVSTK------------
BtCoV512 MSYNSVLDLSKFSCKHKATVVVNLKDSSVTDLVLGLLKNGKLLIRN-NGVVCGFSNHLVNSTK-----------
BtMr-AlphaCoV MSYNSVLDLSKFACKHKATVVVSLKDGDINSMVIGLVKSGKLLVRN-NNKLCGFSNHLVSTK------------
BtNv-AlphaCoV MSYNSVLDLTRFECKHKATVVVNLKESDVNEVVLGLVKSGKLLIRN-TGVVCGYAQPPHLSHV-----------
BtRf-AlphaCoV MSYNSVLDLAKFNCRHKATVIVSLKDKDINDVVLGLIRNGKLLIRN-NGIYMGFSNHLVSTK------------
CDPHE15 MSYNSVLDLSKFACKHKATVVVNLKENAISDLVVSLVRSGKLLVRG-SGPLVNLSNHLVNTK------------
FCoV LSHNSVLDTPKFKCRCNNALIVNLKEKELNEMVVGLLRKGKLLIRN-NGKLLNFGNHLVNVP------------
Ferret-CoV LSYYSVFDLTKFKCKFNNALVVNLKESDINVMVKGLIKGGKLLVRN-NGKLLNFGNHLVNV-------------
HKU10 MSYNSVLDLAKFNCKHRATAVIQLKDSDINDMVLGLIKRGKILVRM-NGTYMGFSNHLVSTKQR----------
HKU2 MSYNSVLDLSKFRCKHKATVIITLKDKDITDMVLGLIKNGKLLIRN-SQKLLNFSNHLVTTK------------
HKU8 MSYNSVLDLSKFECRHKATVVIALKDTDLSEVIVGLIRNGKLLIRK-NGSVCGYGNHLVSTK------------
LuchengRn-CoV LSYNSVLDVNKFRLKCKATPVLSLKDGSFTPLVLTLIKNGKLIVRD-TGVVVSFSNHLVNLTK-----------
Mink-CoV LSYYSVFDLTKFKCKFNNALVVNLKDSDINVMVKGLIKGGKLLVRN-NGKLLNFGNHLVNV-------------
NL63 LSYNSVLDLSKFECKHKATVVVTLKDSDVNDMVLSLIKSGRLLLRN-NGRFGGFSNHLVSTK------------
NL63-related LSYNSVLDLSKFECKHKATVVVTLKDSDVNDMVLGLIKSGRLLLRNSSGRFGGFSNHLVSTKQ-----------
Porcine-EDV MSYNSVLDLSKFNCKHKATVVVNLKDSSISDVVLGLLKNGKLLVRN-NDAICGFSNHLVNVNK-----------
TGE LSHNSVLDTPKFKCRCNNALIVNLKEKELNEMVIGLLRKGKLLIRN-NGKLLNFGNHFVNTP------------
Beta-CoV cons
787***9*969*5*974**7987*624*+*99967*944*9*+9*9-624357375511110-------------
Alpha-CoV cons
9*87**8*769*38956785996**944782895 7*994*9+89*3-842677789354550------------
Combined cons
7837*97*759*28934785986*9336984894 7*944*9++9*3-8323544752++940------------
Alignment qual

Spike (S) Protein

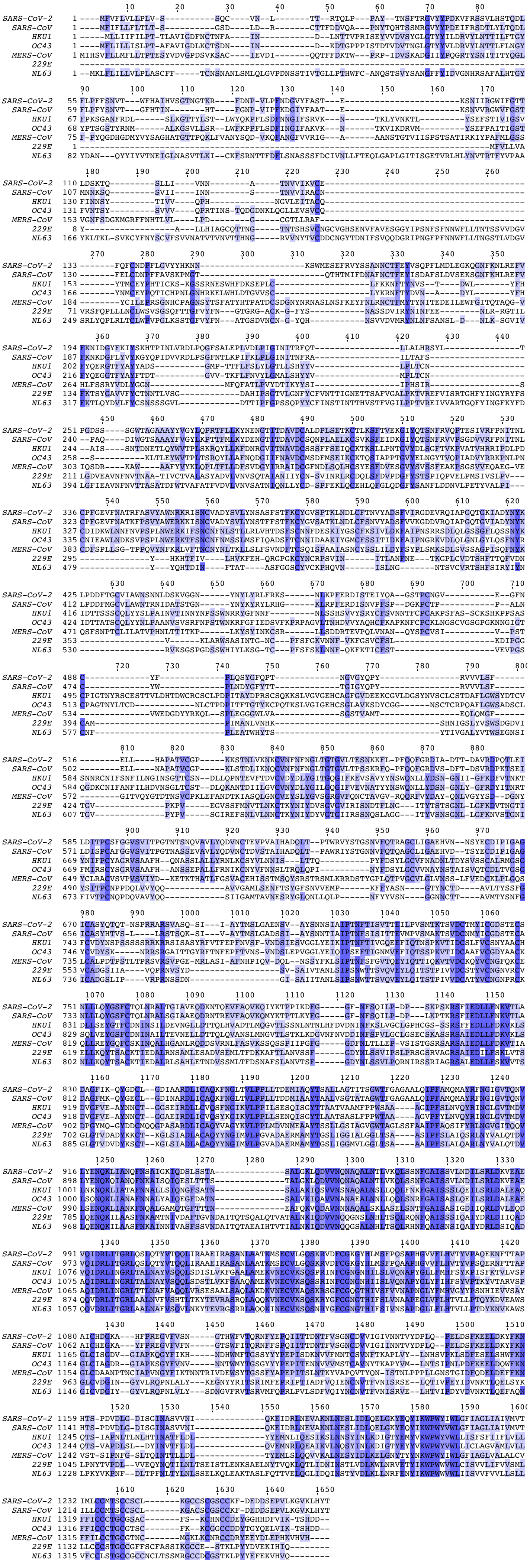

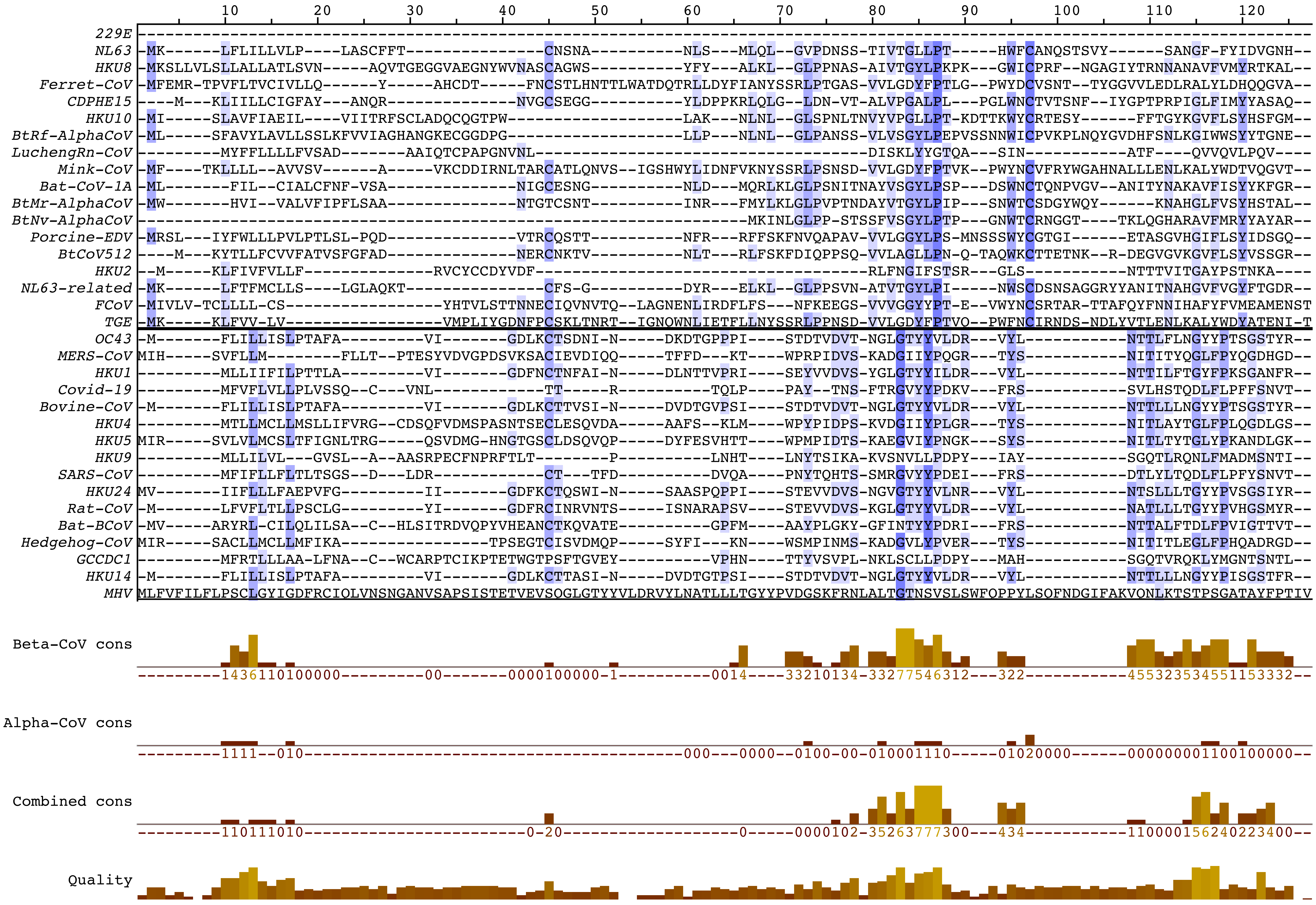

10 20 30 40 50 60 70 80 90 100 110 120
229E
NL63
HKU8
Ferret-CoV
CDPHE15
HKU10
BtRf-AlphaCoV
LuchengRn-CoV
Mink-CoV
Bat-CoV-1A
BtMr-AlphaCoV
BtNv-AlphaCoV
Porcine-EDV
BtCoV512
HKU2
NL63-related
FCoV
TGE
OC43
MERS-CoV
HKU1
Covid-19
Bovine-CoV
HKU4
HKU5
HKU9
SARS-CoV
HKU24
Rat-CoV
Bat-BCoV
Hedgehog-CoV
GCCDC1
HKU14
MHV
Beta-CoV cons
Alpha-CoV cons
Combined cons
Quality

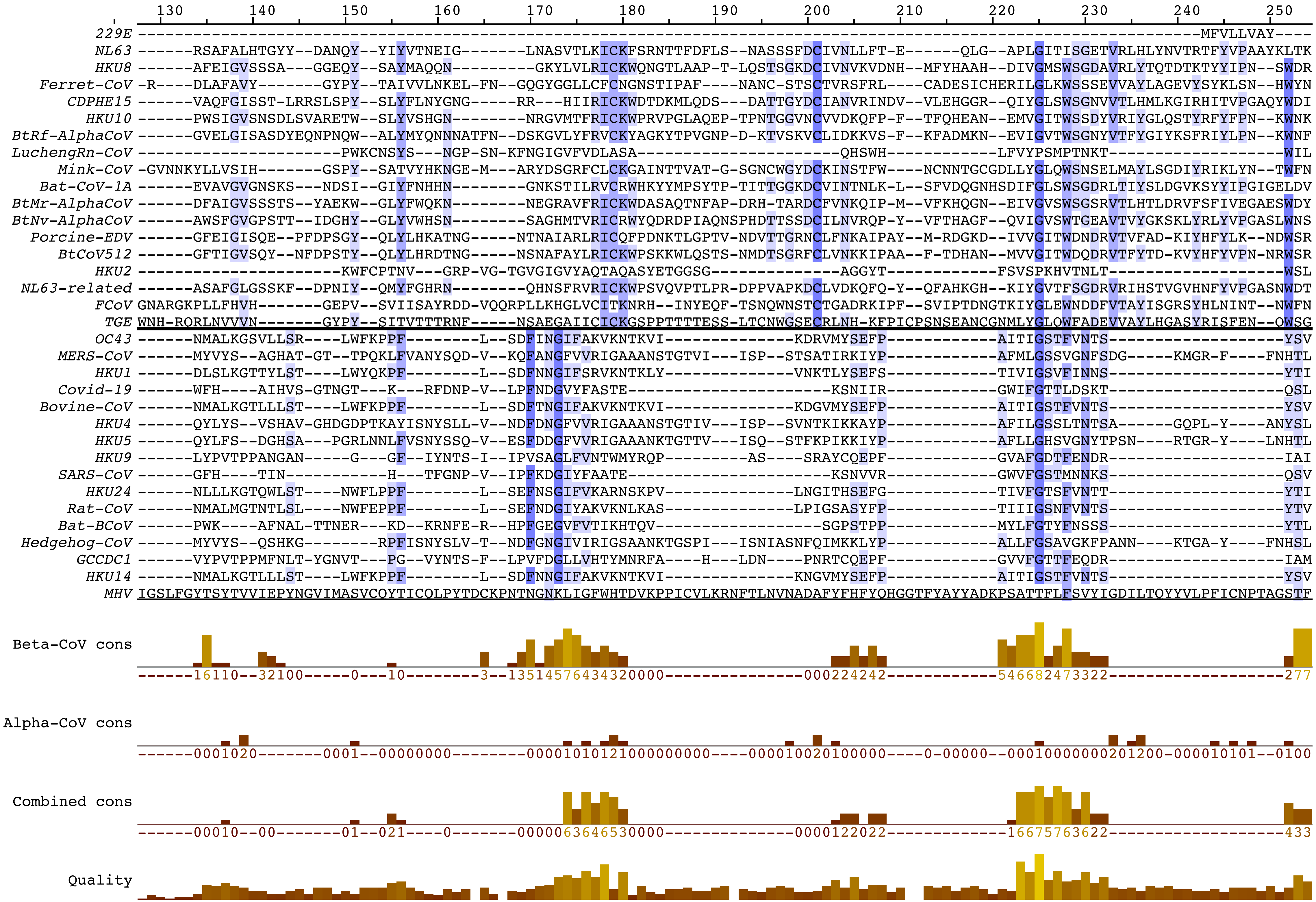
130
140
150
160
170
180
190
200
210
220
230
240
250
229E
NL63
HKU8
Ferret-CoV
CDPHE15
HKU10
BtRf-AlphaCoV
LuchengRn-CoV
Mink-CoV
Bat-CoV-1A
BtMr-AlphaCoV
BtNv-AlphaCoV
Porcine-EDV
BtCoV512
HKU2
NL63-related
FCoV
TGE
OC43
MERS-CoV
HKU1
Covid-19
Bovine-CoV
HKU4
HKU5
HKU9
SARS-CoV
HKU24
Rat-CoV
Bat-BCoV
Hedgehog-CoV
GCCDC1
HKU14
MHV
Beta-CoV cons
Alpha-CoV cons
Combined cons
Quality

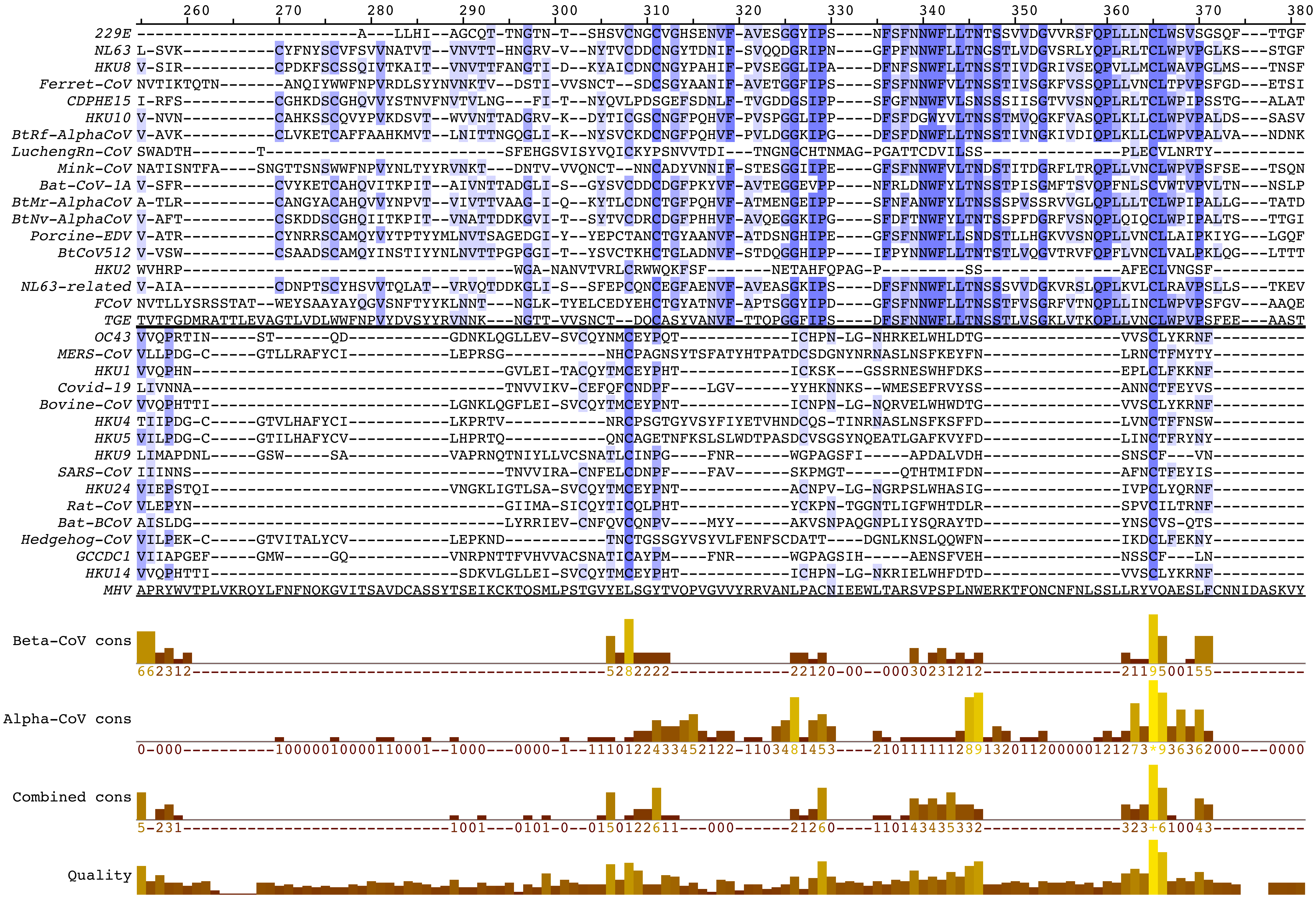
260
270
280
290
300
310
320
330
340
350
360
370
380
229E
NL63
HKU8
Ferret-CoV
CDPHE15
HKU10
BtRf-AlphaCoV
LuchengRn-CoV
Mink-CoV
Bat-CoV-1A
BtMr-AlphaCoV
BtNv-AlphaCoV
Porcine-EDV
BtCoV512
HKU2
NL63-related
FCoV
TGE
OC43
MERS-CoV
HKU1
Covid-19
Bovine-CoV
HKU4
HKU5
HKU9
SARS-CoV
HKU24
Rat-CoV
Bat-BCoV
Hedgehog-CoV
GCCDC1
HKU14
MHV
Beta-CoV cons
Alpha-CoV cons
Combined cons
Quality

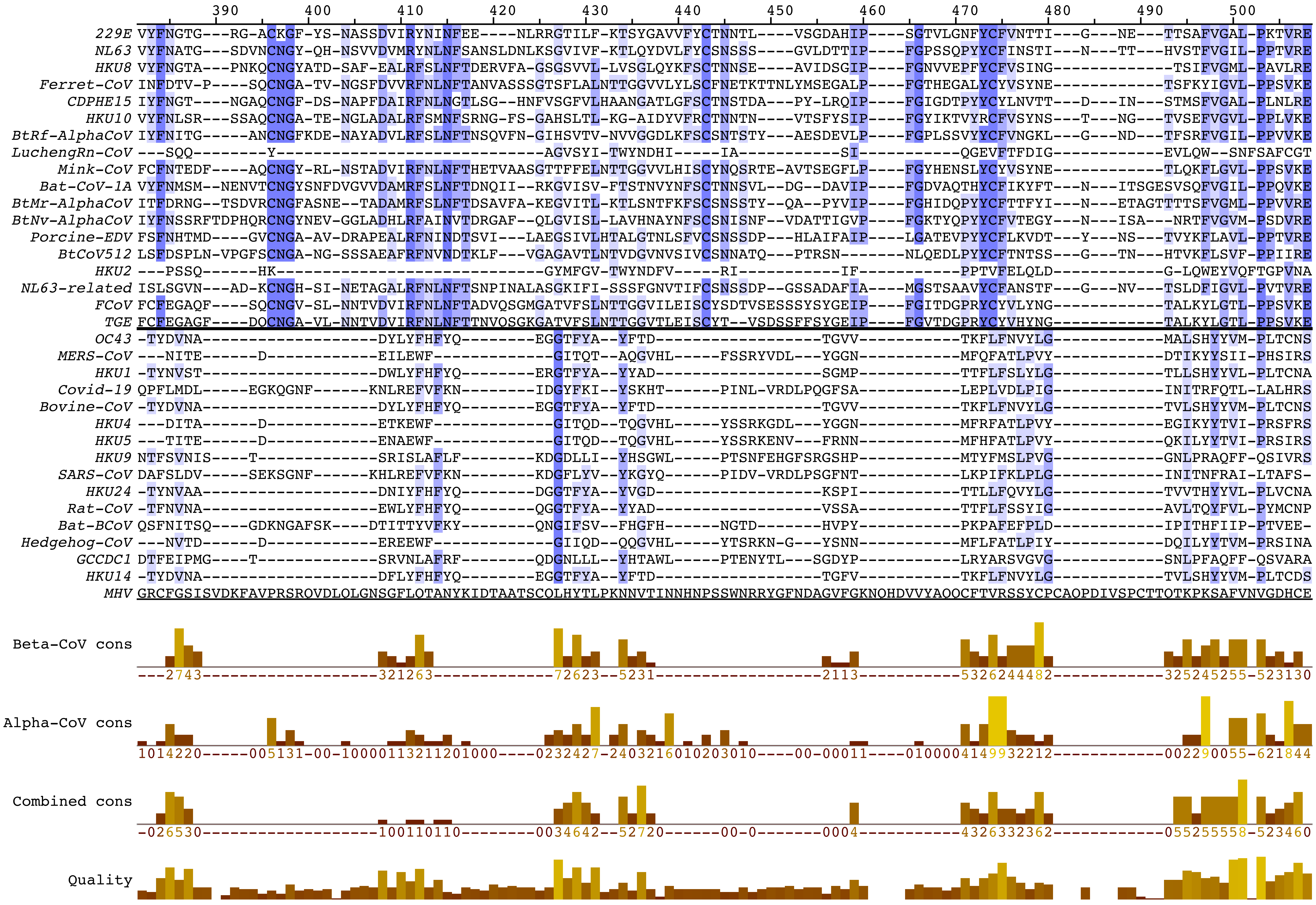

390 400 410 420 430 440 450 460 470 480 490 500
229E VYFNGTG---RG-ACKGF-YS-NASSDVIRYNINFEE----NLRRGTILF-KTSYGAVVFYCTNNTL----VSGDAHIP----SGTVLGNFYCFVNTTI---G---NE---TTSAFVGAL-PKTVRE
NL63 VYFNATG---SDVNCNGY-QH-NSVVDVMRYNLNFSANSLDNLKSGVIVF-KTLQYDVLFYCSNSSS----GVLDTTIP----FGPSSQPYYCFINSTI---N---TT---HVSTFVGIL-PPTVRE
HKU8 VYFNGTA---PNKQCNGYATD-SAF-EALRFSLNFTDERVFA-GSGSVVL-LVSGLQYKFSCTNNSE----AVIDSGIP----FGNVVEPFYCFVSING-------------TSIFVGML-PAVLRE
Ferret-CoV INFDTV-P----SQCNGA-TV-NGSFDVVRFNLNFTANVASSSGTSFLALNTTGGVVLYLSCFNETKTTNLYMSEGALP----FGTHEGALYCYVSYNE-------------TSFKYIGVL-PPSVKE
CDPHE15 IYFNGT----NGAQCNGF-DS-NAPFDAIRFNLNGTLSG--HNFVSGFVLHAANGATLGFSCTNSTDA---PY-LRQIP----FGIGDTPYYCYLNVTT---D---IN---STMSFVGVL-PLNLRE
HKU10 VYFNLSR---SSAQCNGA-TE-NGLADALRFSMNFSRNG-FS-GAHSLTL-KG-AIDYVFRCTNNTN----VTSFYSIP----FGYIKTVYRCFVSYNS---T---NG---TVSEFVGVL-PPLVKE
BtRf-AlphaCoV IYFNITG----ANCNGFKDE-NAYADVLRFSLNFTNSQVFN-GIHSVTV-NVVGGDLKFSCSNTSTY---AESDEVLP----FGPLSSVYYCFVNGKL---G---ND---TFSRFVGIL-PPVVKE
LuchengRn-CoV ---SQQ--------Y-------------------------------AGVSYI-TWYNDHI-----IA----------SI-----------QGEVFTFDIG-------------EVLQW--SNFSAFCGT
Mink-CoV FCFNTEDF----AQCNGY-RL-NSTADVIRFNLNFTHETVAASGTTFFELNTTGGVVLHISCYNQSRTE-AVTSEGFLP----FGYHENSLYCYVSYNE-------------TLQKFLGVL-PPSVKE
Bat-CoV-1A VYFNMSM--NENVTCNGYSNFDVGVVDAMRFSLNFTDNQII--RKGVISV-FTSTNVYNFSCTNNSVL--DG--DAVIP----FGDVAQTHYCFIKYFT---N---ITSGESVSQFVGIL-PPQVKE
BtMr-AlphaCoV ITFDRNG--TSDVRCNGFASNE--TADAMRFSLNFTDSAVFA-KEGVITL-KTLSNTFKFSCSNSSTY--QA--PYVIP----FGHIDQPYYCFTTFYI---N---ETAGTTTSFVGML-PPVVRE
BtNv-AlphaCoV IYFNSSRFTDPHQRCNGYNEV-GGLADHLRFAINVTDRGAF--QLGVISL-LAVHNAYNFSCSNISNF--VDATTIGVP----FGKTYQPYYCFVTEGY---N---ISA---NRTFVGVM-PSDVRE
Porcine-EDV FSFNHTMD----GVCNGA-AV-DRAPEALRFNINDTSVI---LAEGSIVLHTALGTNLSFVCSNSSDP---HLAIFAIP----LGATEVPYYCFLKVDT---Y---NS---TVYKFLAVL-PPTVRE
BtCoV512 LSFDSPLN-VPGFSCNGA-NG-SSSAEAFRFNVNDTKLF---VGAGAVTLNTVDGVNVSIVCSNNATQ---PTRSN--------NLQEDLPYYCFTNTSS---G---TN---HTVKFLSVF-PPIIRE
HKU2 ---PSSQ------HK------------------------------GYMFGV-TWYNDFV-----RI----------IF-----------PPTVFELQLD-------------G-LQWEYVQFTGPVNA
NL63-related ISLSGVN---AD-KCNGH-SI-NETAGALRFNLNFTSNPINALASGKIFI-SSSFGNVTIFCSNSSDP--GSSADAFIA----MGSTSAAVYCFANSTF---G---NV---TSLDFIGVL-PVTVRE
FCoV FCFEGAQF----SQCNGV-SL-NNTVDVIRFNLNFTADVQSGMGATVFSLNTTGGVILEISCYSDTVSESSSYSYGEIP----FGITDGPRYCYVLYNG-------------TALKYLGTL-PPSVKE
TGE FCFEGAGF----DQCNGA-VL-NNTVDVIRFNLNFTTNVQSGKGATVFSLNTTGGVTLEISCYT--VSDSSFFSYGEIP----FGVTDGPRYCYVHYNG-------------TALKYLGTL-PPSVKE
OC43 -TYDVNA-------------------DYLYFHFYQ---------EGGTFYA--YFTD------------------TGVV------------TKFLFNVYLG-------------MALSHYYVM-PLTCNS
MERS-CoV ---NITE------D-------------EILEWF-----------------GITQT--AQGVHL-----FSSRYVDL---YGGN------------MFQFATLPVY-------------DTIKYYSII-PHSIRS
HKU1 -TYNVST-------------------DWLYFHFYQ---------ERGTFYA--YYAD------------------SGMP------------TTFLFSLYLG-------------TLLSHYYVL-PLTCNA
Covid-19 QPFLMDL-----EGKQGNF------KNLREFVFKN---------IDGYFKI--YSKHT------PINL-VRDLPQGFSA------------LEPLVDLPIG-------------INITRFQTL-LALHRS
Bovine-CoV -TYDVNA-------------------DYLYFHFYQ---------EGGTFYA--YFTD------------------TGVV------------TKFLFNVYLG-------------TVLSHYYVM-PLTCNS
HKU4 ---DITA------D-------------ETKEWF-----------------GITQD--TQGVHL-----YSSRKGDL---YGGN------------MFRFATLPVY-------------EGIKYYTVI-PRSFRS
HKU5 ---TITE------D-------------ENAEWF-----------------GITQD--TQGVHL-----YSSRKENV---FRNN------------MFHFATLPVY-------------QKILYYTVI-PRSIRS
HKU9 NTFSVNIS----T---------------SRISLAFLF---------KDGDLLI--YHSGWL-----PTSNFEHGFSRGSHP------------MTYFMSLPVG-------------GNLPRAQFF-QSIVRS
SARS-CoV DAFSLDV-----SEKSGNF------KHLREFVFKN---------KDGFLYV--YKGYQ------PIDV-VRDLPSGFNT------------LKPIFKLPLG-------------INITNFRAI-LTAFS-
HKU24 -TYNVAA-------------------DNIYFHFYQ---------DGGTFYA--YVGD------------------KSPI------------TTLLFQVYLG-------------TVVTHYYVL-PLVCNA
Rat-CoV -TFNVNA-------------------EWLYFHFYQ---------QGGTFYA--YYAD------------------VSSA------------TTFLFSSYIG-------------AVLTQYFVL-PYMCNP
Bat-BCoV QSFNITSQ----GDKNGAFSK----DTITTYVFKY---------QNGIFSV--FHGFH------NGTD-------HVPY------------PKPAFEFPLD-------------IPITHFIIP-PTVEE-
Hedgehog-CoV ---NVTD------D-------------EREEWF-----------------GIIQD--QQGVHL-----YTSRKN-G---YSNN------------MFLFATLPIY-------------DQILYYTVM-PRSINA
GCCDC1 DTFEIPMG----T---------------SRVNLAFRF---------QDGNLLL--YHTAWL-----PTENYTL---SGDYP------------LRYARSVGVG-------------SNLPFAQFF-QSVARA
HKU14 -TYDVNA-------------------DFLYFHFYQ---------EGGTFYA--YFTD------------------TGFV------------TKFLFNVYLG-------------TVLSHYYVM-PLTCDS
MHV GRCFGSISVDKFAVPRSRQVDLQLGNSGFLQTANYKIDTAATSCQLHYTLPKNNVTINNHNPSSWNRRYGFNDAGVFGKNQHDVVYAQQCFTVRSSYCPCAQPDIVSPCTTQTKPKSAFVNVGDHCE
Beta-CoV cons
---2743-------------------321263--------------72623--5231------------------2113------------5326244482-------------325245255-523130
Alpha-CoV cons
1014220-----005131-00-10000113211201000----0232427-2403216010203010----00-00011----0100004149932212-------------002290055-621844
Combined cons
-026530-------------------100110110---------0034642--52720------00-0--------0004------------4326332362-------------055255558-523460
Quality

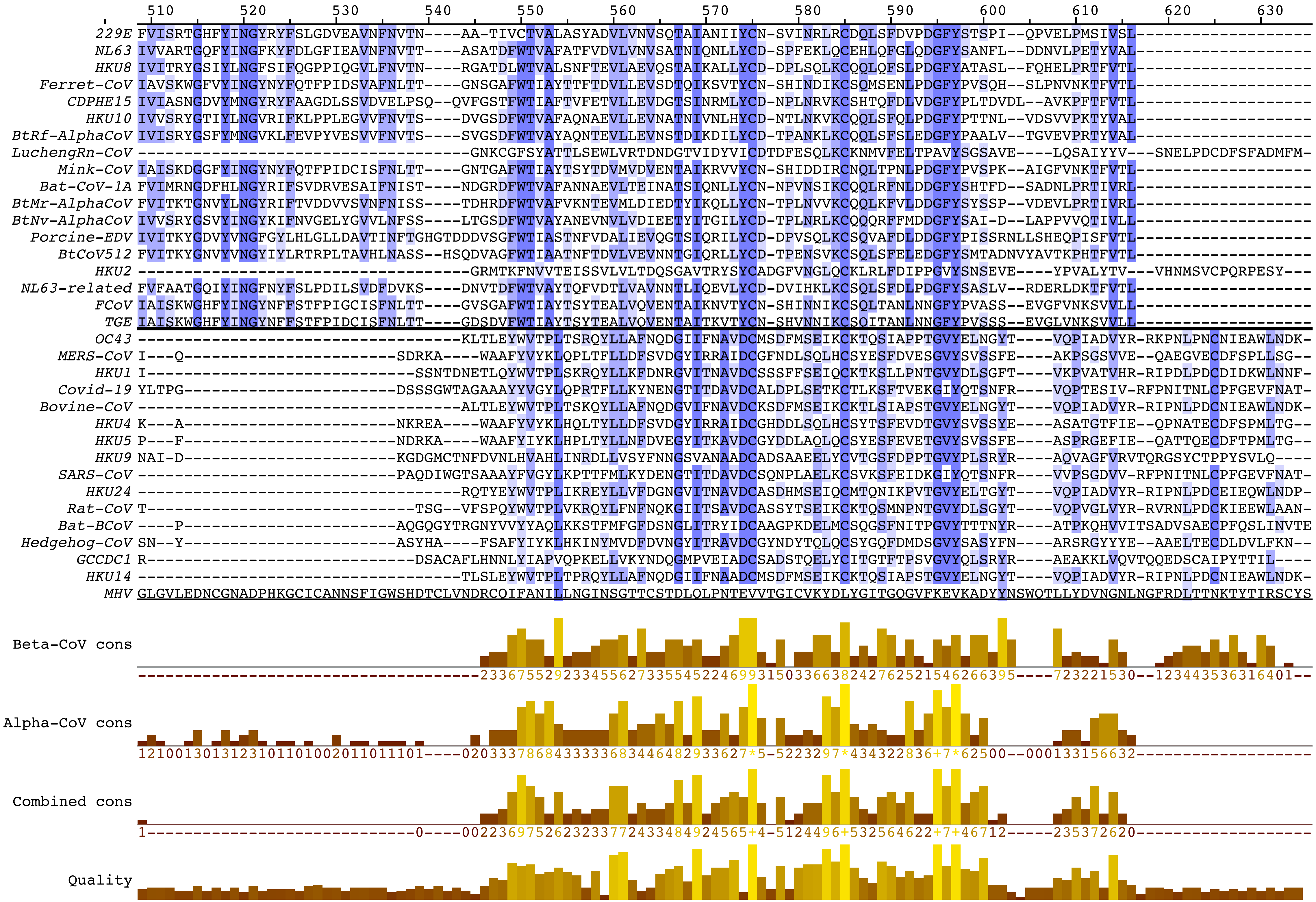

510 520 530 540 550 560 570 580 590 600 610 620 630
229E
NL63
HKU8
Ferret-CoV
CDPHE15
HKU10
BtRf-AlphaCoV
LuchengRn-CoV
Mink-CoV
Bat-CoV-1A
BtMr-AlphaCoV
BtNv-AlphaCoV
Porcine-EDV
BtCoV512
HKU2
NL63-related
FCoV
TGE
OC43
MERS-CoV
HKU1
Covid-19
Bovine-CoV
HKU4
HKU5
HKU9
SARS-CoV
HKU24
Rat-CoV
Bat-BCoV
Hedgehog-CoV
GCCDC1
HKU14
MHV
Beta-CoV cons
Alpha-CoV cons
Combined cons
Quality

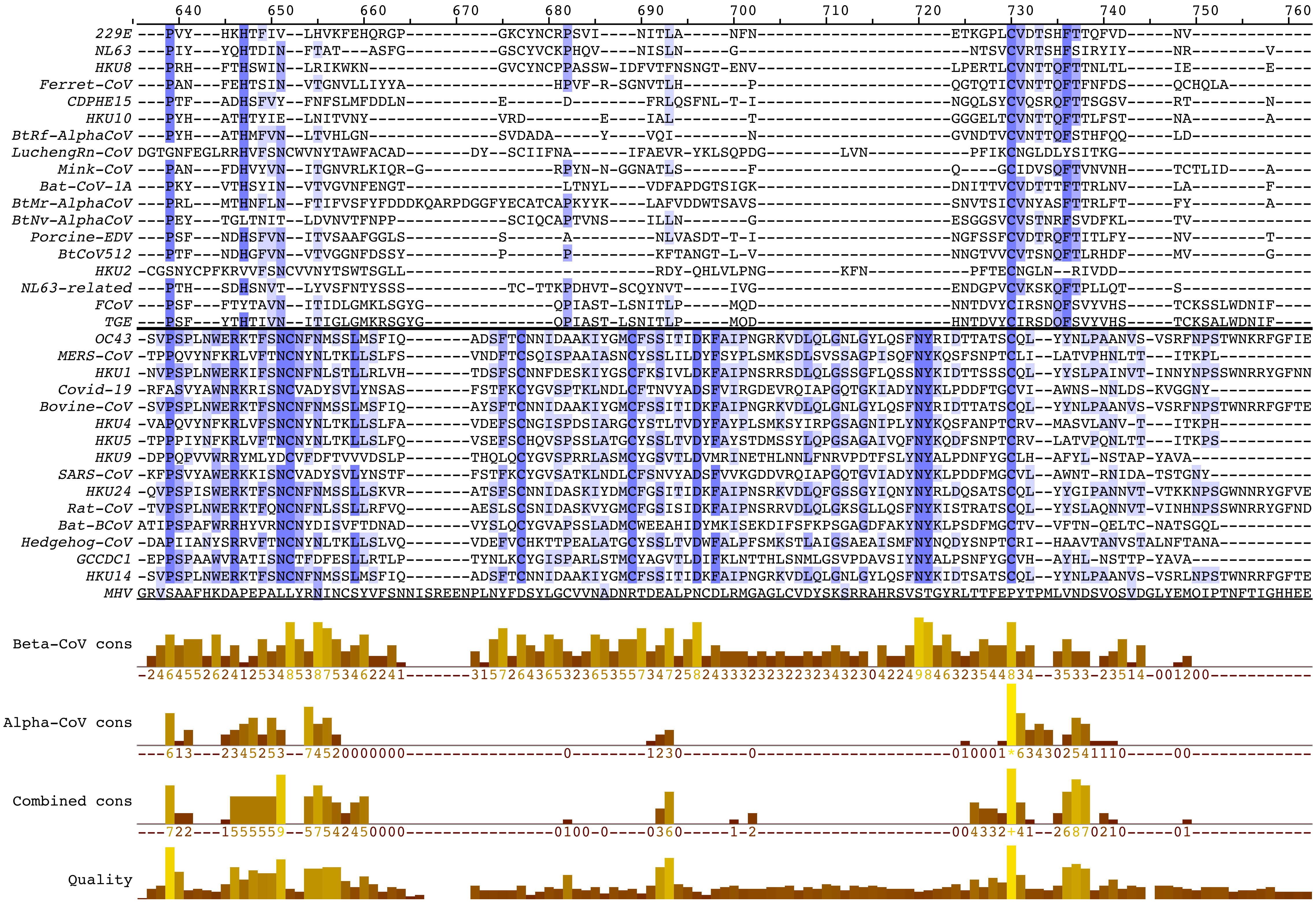
640 650 660 670 680 690 700 710 720 730 740 750 760
229E ---PVY---HKHTFIV--LHVKFEHQRGP----------GKCYNCRPSVI----NITLA-----NFN---------------------ETKGPLCVDTSHFTTQFVD-----NV-------------
NL63 ---PIY---YQHTDIN--FTAT---ASFG----------GSCYVCKPHQV----NISLN-----G-------------------------NTSVCVRTSHFSIRYIY-----NR--------V----
HKU8 ---PRH---FTHSWIN--LRIKWKN--------------GVCYNCPPASSW-IDFVTFNSNGT-ENV---------------------LPERTLCVNTTQFTTNLTL-----IE--------E----
Ferret-CoV ---PAN---FEHTSIN--VTGNVLLIYYA----------------HPVF-R-SGNVTLH-------P---------------------QGTQTICVNTTQFTFNFDS-----QCHQLA---------
CDPHE15 ---PTF---ADHSFVY--FNFSLMFDDLN----------E------D--------FRLQSFNL-T-I---------------------NGQLSYCVQSRQFTTSGSV-----RT--------N----
HKU10 ---PYH---ATHTYIE--LNITVNY--------------VRD--------E----IAL--------T---------------------GGGELTCVNTTQFTTLFST-----NA--------A----
BtRf-AlphaCoV ---PYH---ATHMFVN--LTVHLGN--------------SVDADA-----Y----VQI--------N---------------------GVNDTVCVNTTQFSTHFQQ-----LD-------------
LuchengRn-CoV DGTGNFEGLRRHVFSNCWVNYTAWFACAD-------DY--SCIIFNA------IFAEVR-YKLSQPDG--------LVN-----------PFIKCNGLDLYSITKG---------------------
Mink-CoV ---PAN---FDHVYVN--ITGNVRLKIQR-G--------------RPYN-N-GGNATLS-------F---------------------Q----GCIDVSQFTVNVNH-----TCTLID----A----
Bat-CoV-1A ---PKY---VTHSYIN--VTVGVNFENGT----------------LTNYL----VDFAPDGTSIGK----------------------DNITTVCVDTTTFTTRLNV-----LA--------F----
BtMr-AlphaCoV ---PRL---MTHNFLN--FTIFVSFYFDDDKQARPDGGFYECATCAPKYYK----LAFVDDWTSAVS---------------------SNVTSICVNYASFTTRLFT-----FY--------A----
BtNv-AlphaCoV ---PEY---TGLTNIT--LDVNVTFNPP------------SCIQCAPTVNS----ILLN-------G---------------------ESGGSVCVSTNRFSVDFKL-----TV-------------
Porcine-EDV ---PSF---NDHSFVN--ITVSAAFGGLS----------S------A---------NLVASDT-T-I---------------------NGFSSFCVDTRQFTITLFY-----NV--------T----
BtCoV512 ---PTF---NDHGFVN--VTVGGNFDSSY----------P------P---------KFTANGT-L-V---------------------NNGTVVCVTSNQFTLRHDF-----MV--------G----
HKU2 -CGSNYCPFKRVVFSNCVVNYTSWTSGLL---------------------------RDY-QHLVLPNG--------KFN-----------PFTECNGLN--RIVDD---------------------
NL63-related ---PTH---SDHSNVT--LYVSFNTYSSS-----------TC-TTKPDHVT-SCQYNVT-----IVG---------------------ENDGPVCVKSKQFTPLLQT-----S--------------
FCoV ---PSF---FTYTAVN--ITIDLGMKLSGYG--------------QPIAST-LSNITLP-----MQD---------------------NNTDVYCIRSNQFSVYVHS-----TCKSSLWDNIF----
TGE ---PSF---YTHTIVN--ITIGLGMKRSGYG--------------QPIAST-LSNITLP-----MQD---------------------HNTDVYCIRSDQFSVYVHS-----TCKSALWDNIF----
OC43 -SVPSPLNWERKTFSNCNFNMSSLMSFIQ-------ADSFTCNNIDAAKIYGMCFSSITIDKFAIPNGRKVDLQLGNLGYLQSFNYRIDTTATSCQL--YYNLPAANVS-VSRFNPSTWNKRFGFIE
MERS-CoV -TPPQVYNFKRLVFTNCNYNLTKLLSLFS-------VNDFTCSQISPAAIASNCYSSLILDYFSYPLSMKSDLSVSSAGPISQFNYKQSFSNPTCLI--LATVPHNLTT---ITKPL----------
HKU1 -NVPSPLNWERKIFSNCNFNLSTLLRLVH-------TDSFSCNNFDESKIYGSCFKSIVLDKFAIPNSRRSDLQLGSSGFLQSSNYKIDTTSSSCQL--YYSLPAINVT-INNYNPSSWNRRYGFNN
Covid-19 -RFASVYAWNRKRISNCVADYSVLYNSAS-------FSTFKCYGVSPTKLNDLCFTNVYADSFVIRGDEVRQIAPGQTGKIADYNYKLPDDFTGCVI--AWNS-NNLDS-KVGGNY-----------
Bovine-CoV -SVPSPLNWERKTFSNCNFNMSSLMSFIQ-------AYSFTCNNIDAAKIYGMCFSSITIDKFAIPNGRKVDLQLGNLGYLQSFNYRIDTTATSCQL--YYNLPAANVS-VSRFNPSTWNRRFGFTE
HKU4 -VAPQVYNFKRLVFSNCNYNLTKLLSLFA-------VDEFSCNGISPDSIARGCYSTLTVDYFAYPLSMKSYIRPGSAGNIPLYNYKQSFANPTCRV--MASVLANV-T---ITKPH----------
HKU5 -TPPPIYNFKRLVFTNCNYNLTKLLSLFQ-------VSEFSCHQVSPSSLATGCYSSLTVDYFAYSTDMSSYLQPGSAGAIVQFNYKQDFSNPTCRV--LATVPQNLTT---ITKPS----------
HKU9 -DPPQPVVWRRYMLYDCVFDFTVVVDSLP-------THQLQCYGVSPRRLASMCYGSVTLDVMRINETHLNNLFNRVPDTFSLYNYALPDNFYGCLH--AFYL-NSTAP-YAVA-------------
SARS-CoV -KFPSVYAWERKKISNCVADYSVLYNSTF-------FSTFKCYGVSATKLNDLCFSNVYADSFVVKGDDVRQIAPGQTGVIADYNYKLPDDFMGCVL--AWNT-RNIDA-TSTGNY-----------
HKU24 -QVPSPISWERKTFSNCNFNMSSLLSKVR-------ATSFSCNNIDASKIYDMCFGSITIDKFAIPNSRKVDLQFGSSGYIQNYNYRLDQSATSCQL--YYGIPANNVT-VTKKNPSGWNNRYGFVE
Rat-CoV -TVPSPLNWERKTFQNCNFNLSSLLRFVQ-------AESLSCSNIDASKVYGMCFGSISIDKFAIPNSRRVDLQLGKSGLLQSFNYKISTRATSCQL--YYSLAQNNVT-VINHNPSSWNRRYGFND
Bat-BCoV ATIPSPAFWRRHYVRNCNYDISVFTDNAD-------VYSLQCYGVAPSSLADMCWEEAHIDYMKISEKDIFSFKPSGAGDFAKYNYKLPSDFMGCTV--VFTN-QELTC-NATSGQL----------
Hedgehog-CoV -DAPIIANYSRRVFTNCNYNLTKLLSLVQ-------VDEFVCHKTTPEALATGCYSSLTVDWFALPFSMKSTLAIGSAEAISMFNYNQDYSNPTCRI--HAAVTANVSTALNFTANA----------
GCCDC1 -EPPSPAAWVRATISNCTFDFESLLRTLP-------TYNLKCYGISPARLSTMCYAGVTLDIFKLNTTHLSNMLGSVPDAVSIYNYALPSNFYGCVH--AYHL-NSTTP-YAVA-------------
HKU14 -SVPSPLNWERKTFSNCNFNMSSLMSFIQ-------ADSFTCNNIDAAKIYGMCFSSITIDKFAIPNGRKVDLQLGNLGYLQSFNYKIDTSATSCQL--YYNLPAANVS-VSRLNPSTWNRRFGFTE
MHV GRVSAAFHKDAPEPALLYRNINCSYVFSNNISREENPLNYFDSYLGCVVNADNRTDEALPNCDLRMGAGLCVDYSKSRRAHRSVSTGYRLTTFEPYTPMLVNDSVQSVDGLYEMQIPTNFTIGHHEE
Beta-CoV cons
-2464552624125348538753462241-------3157264365323653557347258243332323223234323042249846323544834--3533-23514-001200-----------
Alpha-CoV cons
---613---2345253--74520000000-----------------0--------1230-------0---------------------010001*634302541110-----00-------------
Combined cons
---722---1555559--57542450000----------------0100-0----0360-----1-2---------------------004332+41--26870210-----01-------------
Quality

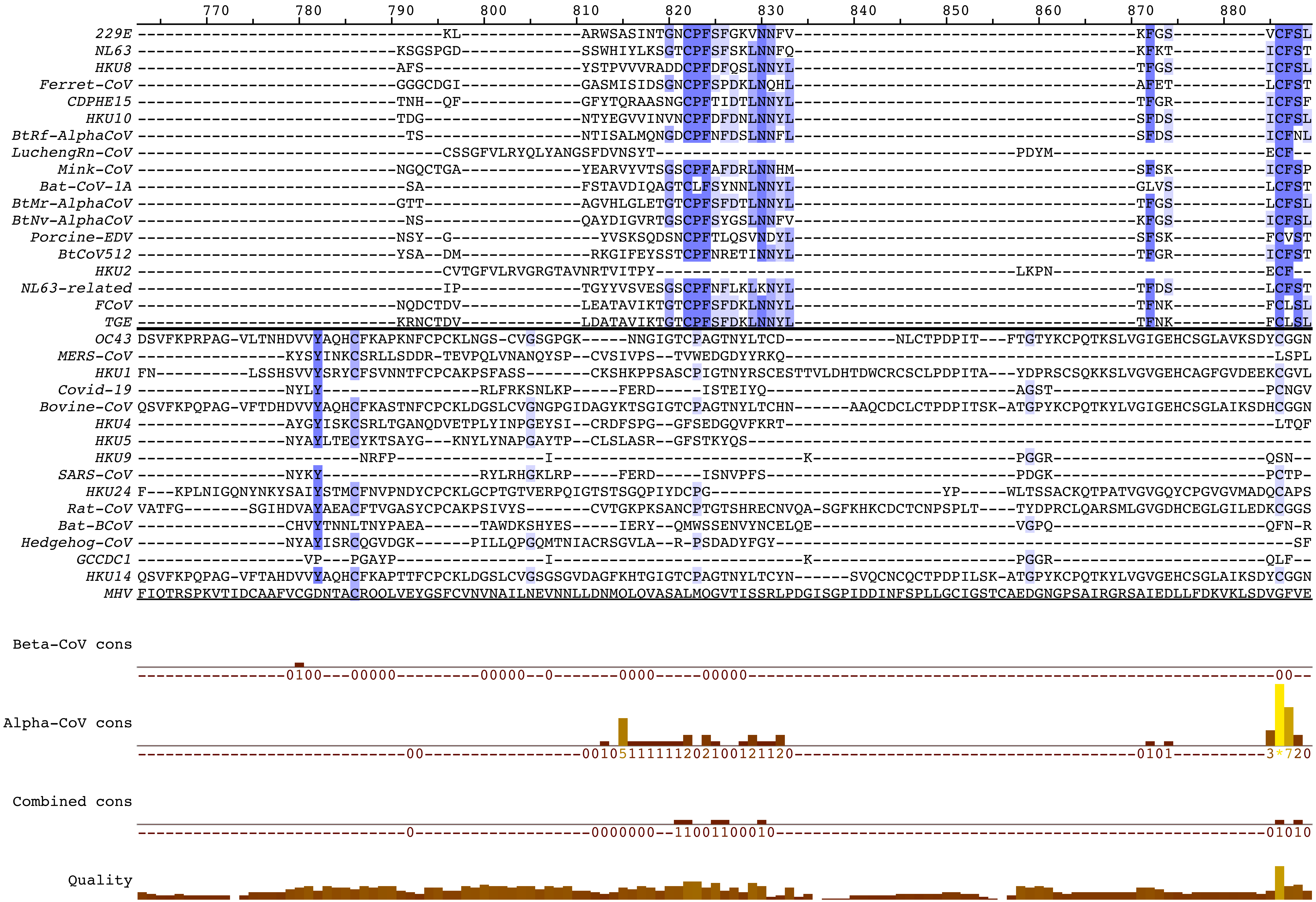
770 780 790 800 810 820 830 840 850 860 870 880
229E ---------------------------------KL-------------ARWSASINTGNCPFSFGKVNNFV-------------------------------------KFGS-----------VCFSL
NL63 ----------------------------KSGSPGD-------------SSWHIYLKSGTCPFSFSKLNNFQ-------------------------------------KFKT-----------ICFST
HKU8 ----------------------------AFS-----------------YSTPVVVRADDCPFDFQSLNNYL-------------------------------------TFGS-----------ICFSL
Ferret-CoV ----------------------------GGGCDGI-------------GASMISIDSGNCPFSPDKLNQHL-------------------------------------AFET-----------LCFST
CDPHE15 ----------------------------TNH--QF-------------GFYTQRAASNGCPFTIDTLNNYL-------------------------------------TFGR-----------ICFSF
HKU10 ----------------------------TDG-----------------NTYEGVVINVNCPFDFDNLNNYL-------------------------------------TFDS-----------ICFSL
BtRf-AlphaCoV ----------------------------TS------------------NTISALMQNGDCPFNFDSLNNFL-------------------------------------SFDS-----------ICFNL
LuchengRn-CoV ---------------------------------CSSGFVLRYQLYANGSFDVNSYT-------------------------------------PDYM----------------------ECF--
Mink-CoV ----------------------------NGQCTGA-------------YEARVYVTSGSCPFAFDRLNNHM-------------------------------------SFSK-----------ICFSP
Bat-CoV-1A ----------------------------SA------------------FSTAVDIQAGTCLFSYNNLNNYL-------------------------------------GLVS-----------LCFST
BtMr-AlphaCoV ----------------------------GTT-----------------AGVHLGLETGTCPFSFDTLNNYL-------------------------------------TFGS-----------LCFSL
BtNv-AlphaCoV ----------------------------NS------------------QAYDIGVRTGSCPFSYGSLNNFV-------------------------------------KFGS-----------ICFSL
Porcine-EDV ----------------------------NSY--G----------------YVSKSQDSNCPFTLQSVNDYL-------------------------------------SFSK-----------FCVST
BtCoV512 ----------------------------YSA--DM--------------RKGIFEYSSTCPFNRETINNYL-------------------------------------TFGR-----------ICFST
HKU2 ---------------------------------CVTGFVLRVGRGTAVNRTVITPY-------------------------------------LKPN----------------------ECF--
NL63-related ---------------------------------IP--------------TGYYVSVESGSCPFNFLKLKNYL-------------------------------------TFDS-----------LCFST
FCoV ----------------------------NQDCTDV-------------LEATAVIKTGTCPFSFDKLNNYL-------------------------------------TFNK-----------FCLSL
TGE ----------------------------KRNCTDV-------------LDATAVIKTGTCPFSFDKLNNYL-------------------------------------TFNK-----------FCLSL
OC43 DSVFKPRPAG-VLTNHDVVYAQHCFKAPKNFCPCKLNGS-CVGSGPGK-----NNGIGTCPAGTNYLTCD------------NLCTPDPIT---FTGTYKCPQTKSLVGIGEHCSGLAVKSDYCGGN
MERS-CoV ----------------KYSYINKCSRLLSDDR-TEVPQLVNANQYSP--CVSIVPS--TVWEDGDYYRKQ--LSPL
HKU1 FN----------LSSHSVVYSRYCFSVNNTFCPCAKPSFASS-------CKSHKPPSASCPIGTNYRSCESTTVLDHTDWCRCSCLPDPITA---YDPRSCSQKKSLVGVGEHCAGFGVDEEKCGVL
Covid-19 ----------------NYLY-----------------RLFRKSNLKP-----FERD-----ISTEIYQ---------------------------------AGST--------------------PCNGV
Bovine-CoV QSVFKPQPAG-VFTDHDVVYAQHCFKASTNFCPCKLDGSLCVGNGPGIDAGYKTSGIGTCPAGTNYLTCHN------AAQCDCLCTPDPITSK-ATGPYKCPQTKYLVGIGEHCSGLAIKSDHCGGN
HKU4 ----------------AYGYISKCSRLTGANQDVETPLYINPGEYSI--CRDFSPG--GFSEDGQVFKRT--LTQF
HKU5 ----------------NYAYLTECYKTSAYG---KNYLYNAPGAYTP--CLSLASR--GFSTKYQS---
HKU9 ------------------------NRFP-------------I----------------------------K----------------------PGGR--------------------QSN--
SARS-CoV ----------------NYKY-----------------RYLRHGKLRP-----FERD-----ISNVPFS---------------------------------PDGK--------------------PCTP-
HKU24 F---KPLNIGQNYNKYSAIYSTMCFNVPNDYCPCKLGCPTGTVERPQIGTSTSGQPIYDCPG------------------------YP-----WLTSSACKQTPATVGVGQYCPGVGVMADQCAPS
Rat-CoV VATFG-------SGIHDVAYAEACFTVGASYCPCAKPSIVYS-------CVTGKPKSANCPTGTSHRECNVQA-SGFKHKCDCTCNPSPLT----TYDPRCLQARSMLGVGDHCEGLGILEDKCGGS
Bat-BCoV ----------------CHVYTNNLTNYPAEA------TAWDKSHYES-----IERY--QMWSSENVYNCELQE-----------------------VGPQ--------------------QFN-R
Hedgehog-CoV ----------------NYAYISRCQGVDGK------PILLQPGQMTNIACRSGVLA--R-PSDADYFGY--SF
GCCDC1 ----------------VP---PGAYP-------------I----------------------------K----------------------PGGR--------------------QLF--
HKU14 QSVFKPQPAG-VFTAHDVVYAQHCFKAPTTFCPCKLDGSLCVGSGSGVDAGFKHTGIGTCPAGTNYLTCYN------SVQCNCQCTPDPILSK-ATGPYKCPQTKYLVGVGEHCSGLAIKSDYCGGN
MHV FIQTRSPKVTIDCAAFVCGDNTACRQQLVEYGSFCVNVNAILNEVNNLLDNMQLQVASALMQGVTISSRLPDGISGPIDDINFSPLLGCIGSTCAEDGNGPSAIRGRSAIEDLLFDKVKLSDVGFVE
Beta-CoV cons
----------------0100---00000---------00000--0-------0000-----00000---00--
Alpha-CoV cons
-----------------------------00-------------------0010511111120210012112O-------------------------------------0101------------3*720
Combined cons
-----------------------------0------------------0000000--11001100010--01010
Quality

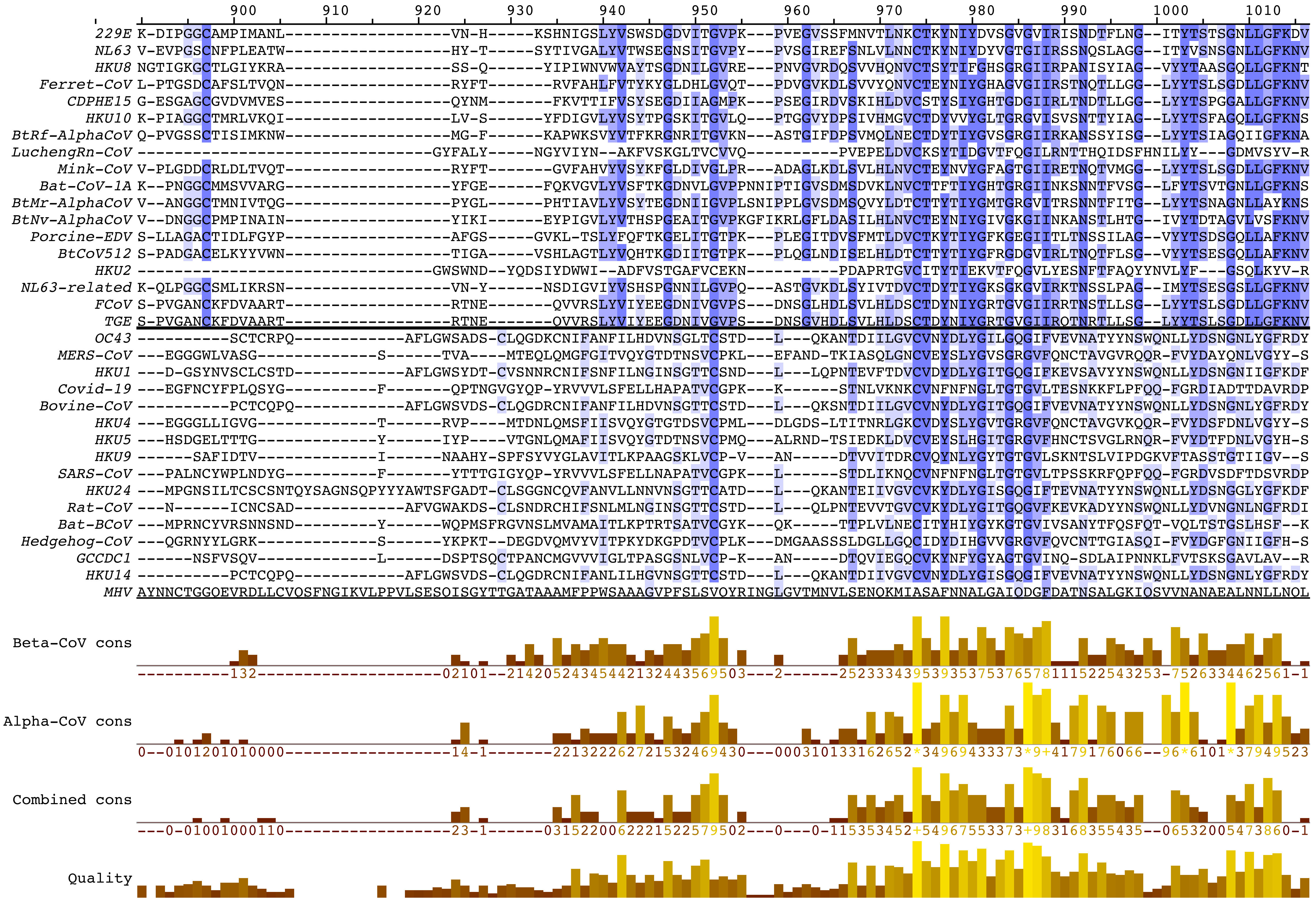
900 910 920 930 940 950 960 970 980 990 1000 1010
229E K-DIPGGCAMPIMANL------------------VN-H-----KSHNIGSLYVSWSDGDVITGVPK---PVEGVSSFMNVTLNKCTKYNIYDVSGVGVIRISNDTFLNG--ITYTSTSGNLLGFKDV
NL63 V-EVPGSCNFPLEATW------------------HY-T-----SYTIVGALYVTWSEGNSITGVPY---PVSGIREFSNLVLNNCTKYNIYDYVGTGIIRSSNQSLAGG--ITYVSNSGNLLGFKNV
HKU8 NGTIGKGCTLGIYKRA------------------SS-Q------YIPIWNVWVAYTSGDNILGVRE---PNVGVRDQSVVHQNVCTSYTIFGHSGRGIIRPANISYIAG--VYYTAASGQLLGFKNT
Ferret-CoV L-PTGSDCAFSLTVQN------------------RYFT-------RVFAHLFVTYKYGLDHLGVQT---PDVGVKDLSVVYQNVCTEYNIYGHAGVGIIRSTNQTLLGG--LYYTSLSGDLLGFKNV
CDPHE15 G-ESGAGCGVDVMVES------------------QYNM-------FKVTTIFVSYSEGDIIAGMPK---PSEGIRDVSKIHLDVCSTYSIYGHTGDGIIRLTNDTLLGG--LYYTSLSGDLLGFKNV
HKU10 K-PIAGGCTMRLVKQI------------------LV-S------YFDIGVLYVSYTPGSKITGVLQ---PTGGVYDPSIVHMGVCTDYVVYGLTGRGIIRLTNDTLLGG--LYYTSPGGALLGFKNS
BtRf-AlphaCoV Q-PVGSSCTISIMKNW------------------MG-F------KAPWKSVYVTFKRGNRITGVKN---ASTGIFDPSVMQLNECTDYTIYGVSGRGIIRKANSSYISG--LYYTSIAGQIIGFKNA
LuchengRn-CoV ------------------------------GYFALY------NGYVIYN--AKFVSKGLTVCVVQ---------PVEPELDVCKSYTIDGVTFQGILRNTTHQIDSFHNILYY---GDMVSYV-R
Mink-CoV V-PLGDDCRLDLTVQT------------------RYFT-------GVFAHVYVSYKFGLDIVGLPR---ADAGLKDLSVLHLNVCTEYNVYGFAGTGIIRETNQTVMGG--LYYTSLSGDLLGFKNV
Bat-CoV-1A K--PNGGCMMSVVARG------------------YFGE-------FQKVGVLYVSFTKGDNVLGVPPNNIPTIGVSDMSDVKLNVCTTFTIYGHTGRGIINKSNNTFVSG--LFYTSVTGNLLGFKNS
BtMr-AlphaCoV V--ANGGCTMNIVTQG------------------PYGL------PHTIAVLYVSYTEGDNIIGVPLSNIPPLGVSDMSQVYLDTCTTYTIYGMTGRGVITRSNNTFITG--LYYTSNAGNLLAYKNS
BtNv-AlphaCoV V--DNGGCPMPINAIN------------------YIKI------EYPIGVLYVTHSPGEAITGVPKGFIKRLGFLDASILHLNVCTEYNIYGIVGKGIINKANSTLHTG--IVYTDTAGVLVSFKNV
Porcine-EDV S-LLAGACTIDLFGYP------------------AFGS-----GVKL-TSLYFQFTKGELITGTPK---PLEGITDVSFMTLDVCTKYTIYGFKGEGIITLTNSSILAG--VYYTSDSGQLLAFKNV
BtCoV512 S-PADGACELKYYVWN------------------TIGA-----VSHLAGTLYVQHTKGDIITGTPK---PLQGLNDISELHLDTCTTYTIYGFRGDGVIRLTNQTFLSG--VYYTSESGQLLAFKNV
HKU2 ------------------------------GWSWND--YQDSIYDWWI--ADFVSTGAFVCEKN---------PDAPRTGVCITYTIEKVTFQGVLYESNFTFAQYYNVLYF---GSQLKYV-R
NL63-related K-QLPGGCSMLIKRSN------------------VN-Y------NSDIGVIYVSHSPGNNILGVPQ---ASTGVKDLSYIVTDVCTDYTIYGKSGKGVIRKTNSSLPAG--IMYTSESGSLLGFKNV
FCoV S-PVGANCKFDVAART------------------RTNE-------QVVRSLYVIYEEGDNIVGVPS---DNSGLHDLSVLHLDSCTDYNIYGRTGVGIIRRTNSTLLSG--LYYTSLSGDLLGFKNV
TGE S-PVGANCKFDVAART------------------RTNE-------QVVRSLYVIYEEGDNIVGVPS---DNSGVHDLSVLHLDSCTDYNIYGRTGVGIIRQTNRTLLSG--LYYTSLSGDLLGFKNV
OC43 ----------SCTCRPQ-------------AFLGWSADS-CLQGDKCNIFANFILHDVNSGLTCSTD---L---QKANTDIILGVCVNYDLYGILGQGIFVEVNATYYNSWQNLLYDSNGNLYGFRDY
MERS-CoV ---EGGGWLVASG-------------S------TVA----MTEQLQMGFGITVQYGTDTNSVCPKL---EFAND-TKIASQLGNCVEYSLYGVSGRGVFQNCTAVGVRQQR-FVYDAYQNLVGYY-S
HKU1 ---D-GSYNVSCLCSTD-------------AFLGWSYDT-CVSNNRCNIFSNFILNGINSGTTCSND---L---LQPNTEVFTDVCVDYDLYGITGQGIFKEVSAVYYNSWQNLLYDSNGNIIGFKDF
Covid-19 ---EGFNCYFPLQSYG-----------F-------QPTNGVGYQP-YRVVVLSFELLHAPATVCGPK---K------STNLVKNKCVNFNFNGLTGTGVLTESNKKFLPFQQ-FGRDIADTTDAVRDP
Bovine-CoV ----------PCTCQPQ-------------AFLGWSVDS-CLQGDRCNIFANFILHDVNSGTTCSTD---L---QKSNTDIILGVCVNYDLYGITGQGIFVEVNATYYNSWQNLLYDSNGNLYGFRDY
HKU4 ---EGGGLLIGVG-------------T------RVP----MTDNLQMSFIISVQYGTGTDSVCPML---DLGDS-LTITNRLGKCVDYSLYGVTGRGVFQNCTAVGVKQQR-FVYDSFDNLVGYY-S
HKU5 ---HSDGELTTTG-------------Y------IYP----VTGNLQMAFIISVQYGTDTNSVCPMQ---ALRND-TSIEDKLDVCVEYSLHGITGRGVFHNCTSVGLRNQR-FVYDTFDNLVGYH-S
HKU9 ------SAFIDTV-------------I------NAAHY-SPFSYVYGLAVITLKPAAGSKLVCP-V---AN-----DTVVITDRCVQYNLYGYTGTGVLSKNTSLVIPDGKVFTASSTGTIIGV--S
SARS-CoV ---PALNCYWPLNDYG-----------F-------YTTTGIGYQP-YRVVVLSFELLNAPATVCGPK---L------STDLIKNQCVNFNFNGLTGTGVLTPSSKRFQPFQQ-FGRDVSDFTDSVRDP
HKU24 ---MPGNSILTCSCSNTQYSAGNSQPYYYAWTSFGADT-CLSGGNCQVFANVLLNNVNSGTTCATD---L---QKANTEIIVGVCVKYDLYGISGQGIFTEVNATYYNSWQNLLYDSNGGLYGFKDF
Rat-CoV ---N------ICNCSAD-------------AFVGWAKDS-CLSNDRCHIFSNLMLNGINSGTTCSTD---L---QLPNTEVVTGVCVKYDLYGITGQGVFKEVKADYYNSWQNLLYDVNGNLNGFRDI
Bat-BCoV ---MPRNCYVRSNNSND-----------Y------WQPMSFRGVNSLMVAMAITLKPTRTSATVCGYK---QK-----TTPLVLNECITYHIYGYKGTGVIVSANYTFQSFQT-VQLTSTGSLHSF--K
Hedgehog-CoV ---QGRNYYLGRK-------------S------YKPKT--DEGDVQMVYVITPKYDKGPDTVCPLK---DMGAASSSLDGLLGQCIDYDIHGVVGRGVFQVCNTTGIASQI-FVYDGFGNIIGFH-S
GCCDC1 ------NSFVSQV-------------L------DSPTSQCTPANCMGVVVIGLTPASGSNLVCP-K---AN-----DTQVIEGQCVKYNFYGYAGTGVINQ-SDLAIPNNKLFVTSKSGAVLAV--R
HKU14 ----------PCTCQPQ-------------AFLGWSVDS-CLQGDRCNIFANLILHGVNSGTTCSTD---L---QKANTDIIVGVCVNYDLYGISGQGIFVEVNATYYNSWQNLLYDSNGNLYGFRDY
MHV AYNNCTGGQEVRDLLCVQSFNGIKVLPPVLSESQISGYTTGATAAAMFPPWSAAAGVPFSLSVQYRINGLGVTMNVLSENQKMIASAFNNALGAIQDGFDATNSALGKIQSVVNANAEALNNLLNQL
Beta-CoV cons
----------132-------------------02101--21420524345442132443569503---2------2523334395393537537657811152254325З-7526334462561-1
Alpha-CoV cons
0--0101201010000-------------------14-1-------221322262721532469430---000310133162652*34969433373*9+41791760 66--96*6101*37949523
Combined cons
---0-01001000110-------------------23-1------031522006222152257950 2---0---0-115353452+549675533 73+983168355435--0653200547386 0-1
Quality

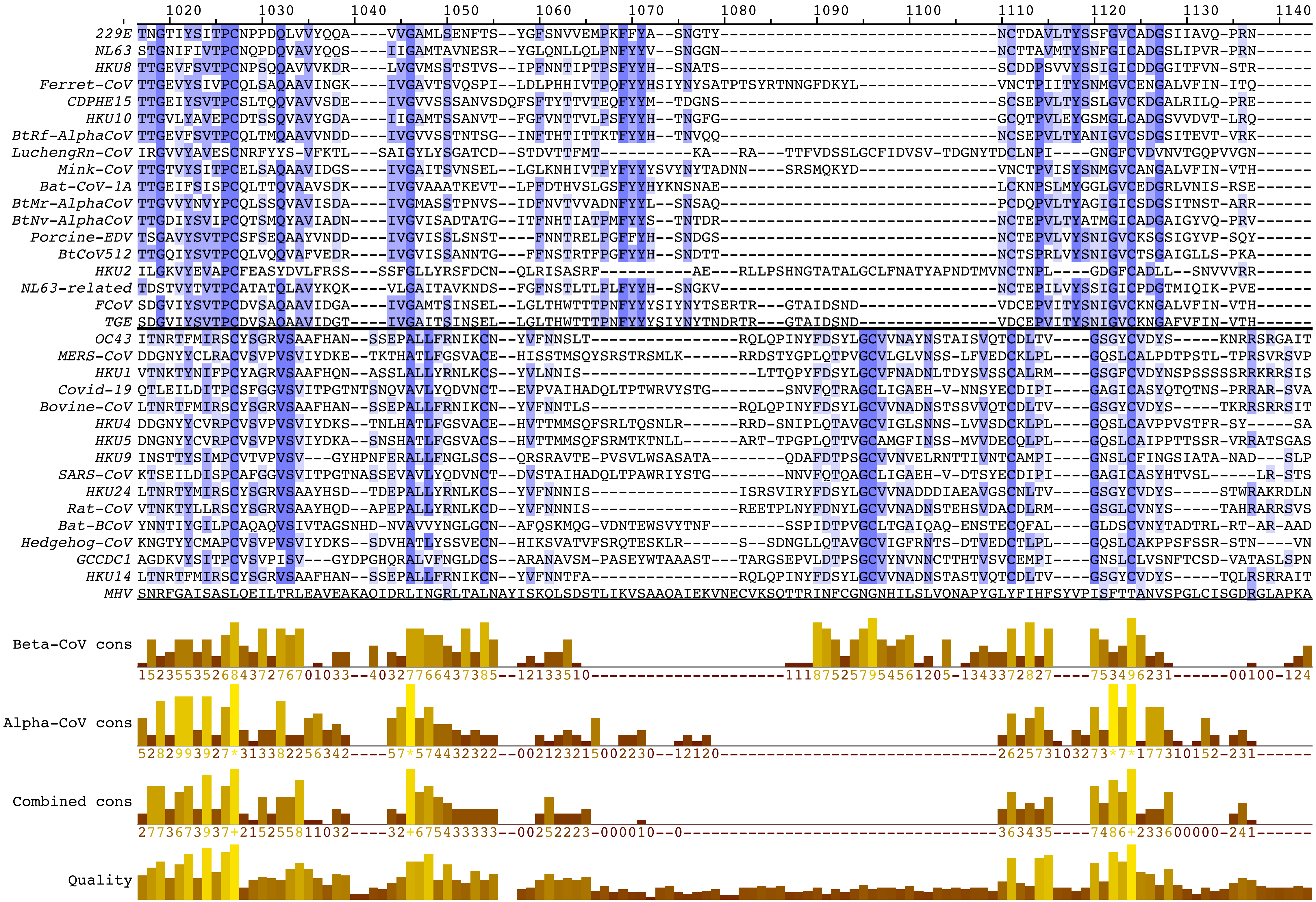
1020 1030 1040 1050 1060 1070 1080 1090 1100 1110 1120 1130 1140
229E TNGTIYSITPCNPPDQLVVYQQA----VVGAMLSENFTS--YGFSNVVEMPKFFYA--SNGTY------------------------------NCTDAVLTYSSFGVCADGSIIAVQ-PRN------
NL63 STGNIFIVTPCNQPDQVAVYQQS----IIGAMTAVNESR--YGLQNLLQLPNFYYV--SNGGN------------------------------NCTTAVMTYSNFGICADGSLIPVR-PRN------
HKU8 TTGEVFSVTPCNPSQQAVVVKDR----LVGVMSSTSTVS--IPFNNTIPTPSFYYH--SNATS------------------------------SCDDPSVVYSSIGICDDGGITFVN-STR------
Ferret-CoV TTGEVYSIVPCQLSAQAAVINGK----IVGAVTSVQSPI--LDLPHHIVTPQFYYHSIYNYSATPTSYRTNNGFDKYL--------------VNCTPIITYSNMGVCENGALVFIN-ITQ------
CDPHE15 TTGEIYSVTPCSLTQQVAVVSDE----IVGVVSSSANVSDQFSFTYTTVTEQFYYM--TDGNS------------------------------SCSEPVLTYSSLGVCKDGALRILQ-PRE------
HKU10 TTGVLYAVEPCDTSSQVAVYGDA----IIGAMTSSANVT--FGFVNTTVLPSFYYH--TNGFG------------------------------GCQTPVLEYGSMGLCADGSVVDVT-LRQ------
BtRf-AlphaCoV TTGEVFSVTPCQLTMQAAVVNDD----IVGVVSSTNTSG--INFTHTITTKTFYYH--TNVQQ------------------------------NCSEPVLTYANIGVCSDGSITEVT-VRK------
LuchengRn-CoV IRGVVYAVESCNRFYYS-VFKTL---SAIGYLYSGATCD--STDVTTFMT----------KA---RA---TTFVDSSLGCFIDVSV-TDGNYTDCLNPI----GNGFCVDVNVTGQPVVGN------
Mink-CoV TTGTVYSITPCELSAQAAVIDGS----IVGAITSVNSEL--LGLKNHIVTPYFYYYSVYNYTADNN----SRSMQKYD--------------VNCTPVISYSNMGVCANGALVFIN-VTH------
Bat-CoV-1A TTGEIFSISPCQLTTQVAAVSDK----IVGVAAATKEVT--LPFDTHVSLGSFYYHYKNSNAE------------------------------LCKNPSLMYGGLGVCEDGRLVNIS-RSE------
BtMr-AlphaCoV TTGVVYNVYPCQLSSQVAVISDA----IVGMASSTPNVS--IDFNVTVVADNFYYL--SNSAQ------------------------------PCDQPVLTYAGIGICSDGSITNST-ARR------
BtNv-AlphaCoV TTGDIYSVIPCQTSMQYAVIADN----IVGVISADTATG--ITFNHTIATPMFYYS--TNTDR------------------------------NCTEPVLTYATMGICADGAIGYVQ-PRV------
Porcine-EDV TSGAVYSVTPCSFSEQAAYVNDD----IVGVISSLSNST----FNNTRELPGFFYH--SNDGS------------------------------NCTEPVLVYSNIGVCKSGSIGYVP-SQY------
BtCoV512 TTGQIYSVTPCQLVQQVAFVEDR----IVGVISSANNTG---FFNSTRTFPGFYYH--SNDTT------------------------------NCTSPRLVYSNIGVCTSGAIGLLS-PKA------
HKU2 ILGKVYEVAPCFEASYDVLFRSS---SSFGLLYRSFDCN--QLRISASRF----------AE---RLLPSHNGTATALGCLFNATYAPNDTMVNCTNPL----GDGFCADLL--SNVVVRR------
NL63-related TDSTVYTVTPCATATQLAVYKQK----VLGAITAVKNDS--FGFNSTLTLPLFYYH--SNGKV------------------------------NCTEPILVYSSIGICPDGTMIQIK-PVE------
FCoV SDGVIYSVTPCDVSAQAAVIDGA----IVGAMTSINSEL--LGLTHWTTTPNFYYYSIYNYTSERTR---GTAIDSND--------------VDCEPVITYSNIGVCKNGALVFIN-VTH------
TGE SDGVIYSVTPCDVSAQAAVIDGT----IVGAITSINSEL--LGLTHWTTTPNFYYYSIYNYTNDRTR---GTAIDSND--------------VDCEPVITYSNIGVCKNGAFVFIN-VTH------
OC43 ITNRTFMIRSCYSGRVSAAFHAN--SSEPALLFRNIKCN--YVFNNSLT----------------RQLQPINYFDSYLGCVVNAYNSTAISVQTCDLTV----GSGYCVDYS-----KNRRSRGAIT
MERS-CoV DDGNYYCLRACVSVPVSVIYDKE--TKTHATLFGSVACE--HISSTMSQYSRSTRSMLK------RRDSTYGPLQTPVGCVLGLVNSS-LFVEDCKLPL----GQSLCALPDTPSTL-TPRSVRSVP
HKU1 VTNKTYNIFPCYAGRVSAAFHQN--ASSLALLYRNLKCS--YVLNNIS-------------------LTTQPYFDSYLGCVFNADNLTDYSVSSCALRM----GSGFCVDYNSPSSSSSRRKRRSIS
Covid-19 QTLEILDITPCSFGGVSVITPGTNTSNQVAVLYQDVNCT--EVPVAIHADQLTPTWRVYSTG--------SNVFQTRAGCLIGAEH-V-NNSYECDIPI----GAGICASYQTQTNS-PRRAR-SVA
Bovine-CoV LTNRTFMIRSCYSGRVSAAFHAN--SSEPALLFRNIKCN--YVFNNTLS----------------RQLQPINYFDSYLGCVVNADNSTSSVVQTCDLTV----GSGYCVDYS-----TKRRSRRSIT
HKU4 DDGNYYCVRPCVSVPVSVIYDKS--TNLHATLFGSVACE--HVTTMMSQFSRLTQSNLR------RRD-SNIPLQTAVGCVIGLSNNS-LVVSDCKLPL----GQSLCAVPPVSTFR-SY-----SA
HKU5 DNGNYYCVRPCVSVPVSVIYDKA--SNSHATLFGSVACS--HVTTMMSQFSRMTKTNLL------ART-TPGPLQTTVGCAMGFINSS-MVVDECQLPL----GQSLCAIPPTTSSR-VRRATSGAS
HKU9 INSTTYSIMPCVTVPVSV---GYHPNFERALLFNGLSCS--QRSRAVTE-PVSVLWSASATA--------QDAFDTPSGCVVNVELRNTTIVNTCAMPI----GNSLCFINGSIATA-NAD---SLP
SARS-CoV KTSEILDISPCAFGGVSVITPGTNASSEVAVLYQDVNCT--DVSTAIHADQLTPAWRIYSTG--------NNVFQTQAGCLIGAEH-V-DTSYECDIPI----GAGICASYHTVSL-----LR-STS
HKU24 LTNRTYMIRSCYSGRVSAAYHSD--TDEPALLYRNLKCS--YVFNNNIS----------------ISRSVIRYFDSYLGCVVNADDDIAEAVGSCNLTV----GSGYCVDYS-----STWRAKRDLN
Rat-CoV VTNKTYLLRSCYSGRVSAAYHQD--APEPALLYRNLKCD--YVFNNNIS----------------REETPLNYFDNYLGCVVNADNSTEHSVDACDLRM----GSGLCVNYS-----TAHRARRSVS
Bat-BCoV YNNTIYGILPCAQAQVSIVTAGSNHD-NVAVVYNGLGCN--AFQSKMQG-VDNTEWSVYTNF--------SSPIDTPVGCLTGAIQAQ-ENSTECQFAL----GLDSCVNYTADTRL-RT-AR-AAD
Hedgehog-CoV KNGTYYCMAPCVSVPVSVIYDKS--SDVHATLYSSVECN--HIKSVATVFSRQTESKLR------S--SDNGLLQTAVGCVIGFRNTS-DTVEDCTLPL----GQSLCAKPPSFSSR-STN----VN
GCCDC1 AGDKVYSITPCVSVPISV---GYDPGHQRALVFNGLDCS--ARANAVSM-PASEYWTAAAST---TARGSEPVLDTPSGCVYNVNNCTTHTVSVCEMPI----GNSLCLVSNFTCSD-VATASLSPN
HKU14 LTNRTFMIRSCYSGRVSAAFHAN--SSEPALLFRNIKCN--YVFNNTFA----------------RQLQPINYFDSYLGCVVNADNSTASTVQTCDLTV----GSGYCVDYS-----TQLRSRRAIT
MHV SNRFGAISASLQEILTRLEAVEAKAQIDRLINGRLTALNAYISKQLSDSTLIKVSAAQAIEKVNECVKSQTTRINFCGNGNHILSLVQNAPYGLYFIHFSYVPISFTTANVSPGLCISGDRGLAPKA
Beta-CoV cons
15235535268437276701033--40327766437385--12133510---------------------111875257954561205-1343372827----753496231------00100-124
Alpha-CoV cons
5282993927*313382256342----57*574432322--002123215002230--12120------------------------------262573103273*7*177310152-231------
Combined cons
2773673937+215255811032----32+675433333--00252223-000010--0----------------------------------363435----7486+233600000-241------
Quality

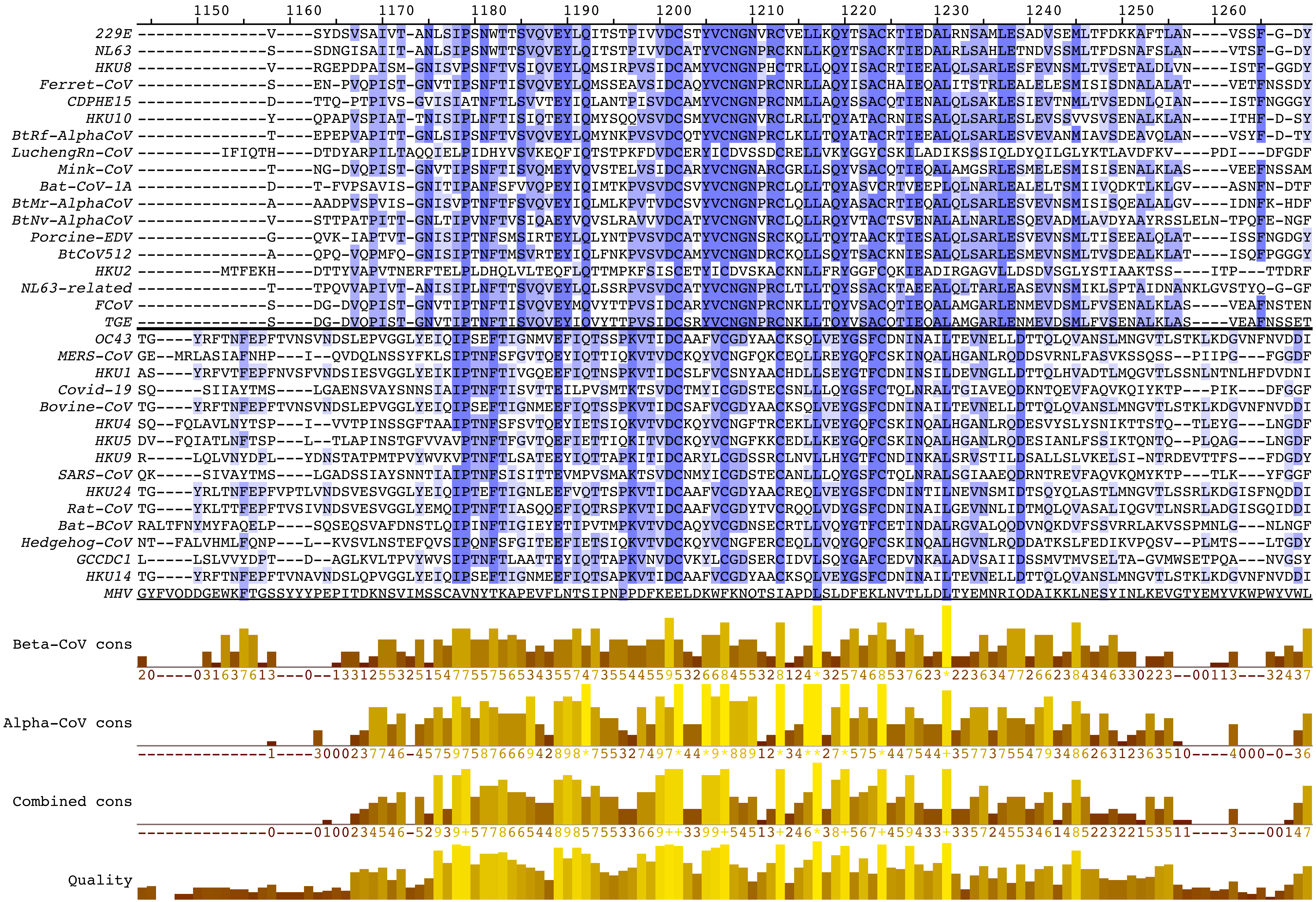
1150 1160 1170 1180 1190 1200 1210 1220 1230 1240 1250 1260
229E --------------V----SYDSVSAIVT-ANLSIPSNWTTSVQVEYLQITSTPIVVDCSTYVCNGNVRCVELLKQYTSACKTIEDALRNSAMLESADVSEMLTFDKKAFTLAN----VSSF-G-DY
NL63 --------------S----SDNGISAIIT-ANLSIPSNWTTSVQVEYLQITSTPIVVDCATYVCNGNPRCKNLLKQYTSACKTIEDALRLSAHLETNDVSSMLTFDSNAFSLAN----VTSF-G-DY
HKU8 --------------V----RGEPDPAISM-GNISVPSNFTVSIQVEYLQMSIRPVSIDCAMYVCNGNPHCTRLLQQYISACRTIEEALQLSARLESFEVNSMLTVSETALDLVN----ISTF-GGDY
Ferret-CoV --------------S----EN-PVQPIST-GNVTIPSNFTISVQVEYLQMSSEAVSIDCAQYVCNGNPRCNRLLAQYISACHAIEQALITSTRLEALELESMISISDNALALAT----VETFNSSDY
CDPHE15 --------------D----TTQ-PTPIVS-GVISIATNFTLSVVTEYIQLANTPISVDCAMYVCNGNPRCNMLLAQYSSACQTIENALQLSAKLESIEVTNMLTVSEDNLQIAN----ISTFNGGGY
HKU10 --------------Y----QPAPVSPIAT-TNISIPLNFTISIQTEYIQMYSQQVSVDCSMYVCNGNVRCLRLLTQYATACRNIESALQLSARLESLEVSSVVSVSENALKLAN----ITHF-D-SY
BtRf-AlphaCoV --------------T----EPEPVAPITT-GNLSIPSNFTVSVQVEYLQMYNKPVSVDCQTYVCNGNPRCLKLLTQYATACRTIEEALQLSARLESVEVANMIAVSDEAVQLAN----VSYF-D-TY
LuchengRn-CoV ---------IFIQTH----DTDYARPILTAQQIELPIDHYVSVKEQFIQTSTPKFDVDCERYICDVSSDCRELLVKYGGYCSKILADIKSSSIQLDYQILGLYKTLAVDFKV----PDI---DFGDF
Mink-CoV --------------T----NG-DVQPIST-GNVTIPSNFTISVQMEYVQVSTELVSIDCARYVCNGNARCGRLLSQYVSACQTIEQALAMGSRLESMELESMISISENALKLAS----VEEFNSSAM
Bat-CoV-1A --------------D----T-FVPSAVIS-GNITIPANFSFVVQPEYIQIMTKPVSVDCSVYVCNGNPRCLQLLTQYASVCRTVEEPLQLNARLEALELTSMIIVQDKTLKLGV----ASNFN-DTF
BtMr-AlphaCoV --------------A----AADPVSPVIS-GNISVPTNFTFSVQVEYIQLMLKPVTVDCSVYVCNGNPRCLQLLAQYASACRTIEQALQLSARLESVEVNSMISISQEALALGV----IDNFK-HDF
BtNv-AlphaCoV --------------V----STTPATPITT-GNLTIPVNFTVSIQAEYVQVSLRAVVVDCATYVCNGNVRCLQLLRQYVTACTSVENALALNARLESQEVADMLAVDYAAYRSSLELN-TPQFE-NGF
Porcine-EDV --------------G----QVK-IAPTVT-GNISIPTNFSMSIRTEYLQLYNTPVSVDCATYVCNGNSRCKQLLTQYTAACKTIESALQLSARLESVEVNSMLTISEEALQLAT----ISSFNGDGY
BtCoV512 --------------A----QPQ-VQPMFQ-GNISIPTNFTMSVRTEYIQLFNKPVSVDCAMYVCNGNDRCKQLLSQYTSACKNIESALQLSARLESMEVNSMLTVSDEALKLAT----ISQFPGGGY
HKU2 ---------MTFEKH----DTTYVAPVTNERFTELPLDHQLVLTEQFLQTTMPKFSISCETYICDVSKACKNLLFRYGGFCQKIEADIRGAGVLLDSDVSGLYSTIAAKTSS----ITP---TTDRF
NL63-related --------------T----TPQVVAPIVT-ANISIPLNFTTSVQVEYLQLSSRPVSVDCATYVCNGNPRCLTLLTQYSSACKTAEEALQLTARLEASEVNSMIKLSPTAIDNANKLGVSTYQ-G-GF
FCoV --------------S----DG-DVQPIST-GNVTIPTNFTISVQVEYMQVYTTPVSIDCARYVCNGNPRCNKLLTQYVSACQTIEQALAMGARLENMEVDSMLFVSENALKLAS----VEAFNSTEN
TGE --------------S----DG-DVQPIST-GNVTIPTNFTISVQVEYIQVYTTPVSIDCSRYVCNGNPRCNKLLTQYVSACQTIEQALAMGARLENMEVDSMLFVSENALKLAS----VEAFNSSET
OC43 TG----YRFTNFEPFTVNSVNDSLEPVGGLYEIQIPSEFTIGNMVEFIQTSSPKVTIDCAAFVCGDYAACKSQLVEYGSFCDNINAILTEVNELLDTTQLQVANSLMNGVTLSTKLKDGVNFNVDDI
MERS-CoV GE--MRLASIAFNHP---I--QVDQLNSSYFKLSIPTNFSFGVTQEYIQTTIQKVTVDCKQYVCNGFQKCEQLLREYGQFCSKINQALHGANLRQDDSVRNLFASVKSSQSS--PIIPG---FGGDF
HKU1 AS----YRFVTFEPFNVSFVNDSIESVGGLYEIKIPTNFTIVGQEEFIQTNSPKVTIDCSLFVCSNYAACHDLLSEYGTFCDNINSILDEVNGLLDTTQLHVADTLMQGVTLSSNLNTNLHFDVDNI
Covid-19 SQ-----SIIAYTMS----LGAENSVAYSNNSIAIPTNFTISVTTEILPVSMTKTSVDCTMYICGDSTECSNLLLQYGSFCTQLNRALTGIAVEQDKNTQEVFAQVKQIYKTP---PIK---DFGGF
Bovine-CoV TG----YRFTNFEPFTVNSVNDSLEPVGGLYEIQIPSEFTIGNMEEFIQTSSPKVTIDCSAFVCGDYAACKSQLVEYGSFCDNINAILTEVNELLDTTQLQVANSLMNGVTLSTKLKDGVNFNVDDI
HKU4 SQ--FQLAVLNYTSP---I--VVTPINSSGFTAAIPTNFSFSVTQEYIETSIQKVTVDCKQYVCNGFTRCEKLLVEYGQFCSKINQALHGANLRQDESVYSLYSNIKTTSTQ--TLEYG---LNGDF
HKU5 DV--FQIATLNFTSP---L--TLAPINSTGFVVAVPTNFTFGVTQEFIETTIQKITVDCKQYVCNGFKKCEDLLKEYGQFCSKINQALHGANLRQDESIANLFSSIKTQNTQ--PLQAG---LNGDF
HKU9 R-----LQLVNYDPL---YDNSTATPMTPVYWVKVPTNFTLSATEEYIQTTAPKITIDCARYLCGDSSRCLNVLLHYGTFCNDINKALSRVSTILDSALLSLVKELSI-NTRDEVTTFS---FDGDY
SARS-CoV QK-----SIVAYTMS----LGADSSIAYSNNTIAIPTNFSISITTEVMPVSMAKTSVDCNMYICGDSTECANLLLQYGSFCTQLNRALSGIAAEQDRNTREVFAQVKQMYKTP---TLK---YFGGF
HKU24 TG----YRLTNFEPFVPTLVNDSVESVGGLYEIQIPTEFTIGNLEEFVQTTSPKVTIDCAAFVCGDYAACREQLVEYGSFCDNINTILNEVNSMIDTSQYQLASTLMNGVTLSSRLKDGISFNQDDI
Rat-CoV TG----YKLTTFEPFTVSIVNDSVESVGGLYEMQIPTNFTIASQQEFIQTRSPKVTIDCAAFVCGDYTVCRQQLVDYGSFCDNINAILGEVNNLIDTMQLQVASALIQGVTLNSRLADGISGQIDDI
Bat-BCoV RALTFNYMYFAQELP----SQSEQSVAFDNSTLQIPINFTIGIEYETIPVTMPKVTVDCAQYVCGDNSECRTLLVQYGTFCETINDALRGVALQQDVNQKDVFSSVRRLAKVSSPMNLG---NLNGF
Hedgehog-CoV NT--FALVHMLFQNP---L--KVSVLNSTEFQVSIPQNFSFGITEEFIETSIQKVTVDCKQYVCNGFERCEQLLVEYGQFCSKINQALHGVNLRQDDATKSLFEDIKVPQSV--PLMTS---LTGDY
GCCDC1 L-----LSLVVYDPT---D--AGLKVLTPVYWVSIPTNFTLAATTEYIQTTAPKVNVDCVKYLCGDSERCIDVLSQYGAFCEDVNKALADVSAIIDSSMVTMVSELTA-GVMWSETPQA---NVGSY
HKU14 TG----YRFTNFEPFTVNAVNDSLQPVGGLYEIQIPSEFTIGNMEEFIQTSAPKVTIDCAAFVCGDYAACKSQLVEYGSFCDNINAILTEVNELLDTTQLQVANSLMNGVTLSTKLKDGVNFNVDDI
MHV GYFVQDDGEWKFTGSSYYYPEPITDKNSVIMSSCAVNYTKAPEVFLNTSIPNPPDFKEELDKWFKNQTSIAPDLSLDFEKLNVTLLDLTYEMNRIQDAIKKLNESYINLKEVGTYEMYVKWPWYVWL
Beta-CoV cons
20----031637613---0--133125532515477557565343557473554455953266845532812 4*3257468537623*22363477266238434633022 3--00113---32437
Alpha-CoV cons
--------------1----3000237746-4575975876669 42898*755327497*44*9*88912*34**27*575*447544+35773755479348626312363510----4000-0-36
Combined cons
--------------0----0100234546-52939+577866544898575533669++3399+54513+246*38+567+459433+33572455346148522322153511----3---00147
Quality

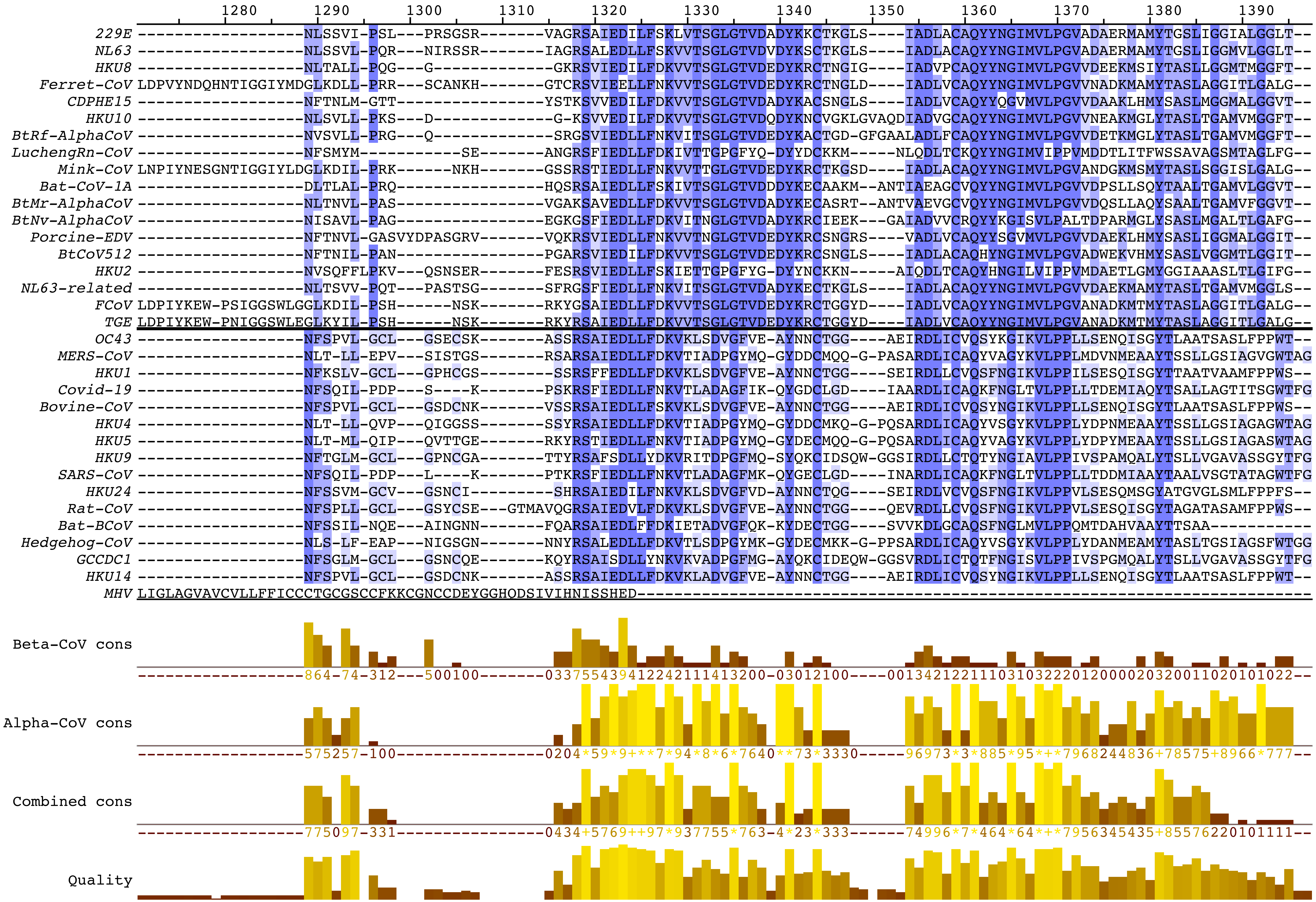
1280
1290
1300
1310
1320
1330
1340
1350
1360
1370
1380
1390
229E
NL63
HKU8
Ferret-CoV
CDPHE15
HKU10
BtRf-AlphaCoV
LuchengRn-CoV
Mink-CoV
Bat-CoV-1A
BtMr-AlphaCoV
BtNv-AlphaCoV
Porcine-EDV
BtCoV512
HKU2
NL63-related
FCoV
TGE
OC43
MERS-CoV
HKU1
Covid-19
Bovine-CoV
HKU4
HKU5
HKU9
SARS-CoV
HKU24
Rat-CoV
Bat-BCoV
Hedgehog-CoV
GCCDC1
HKU14
MHV
Beta-CoV cons
Alpha-CoV cons
Combined cons
Quality

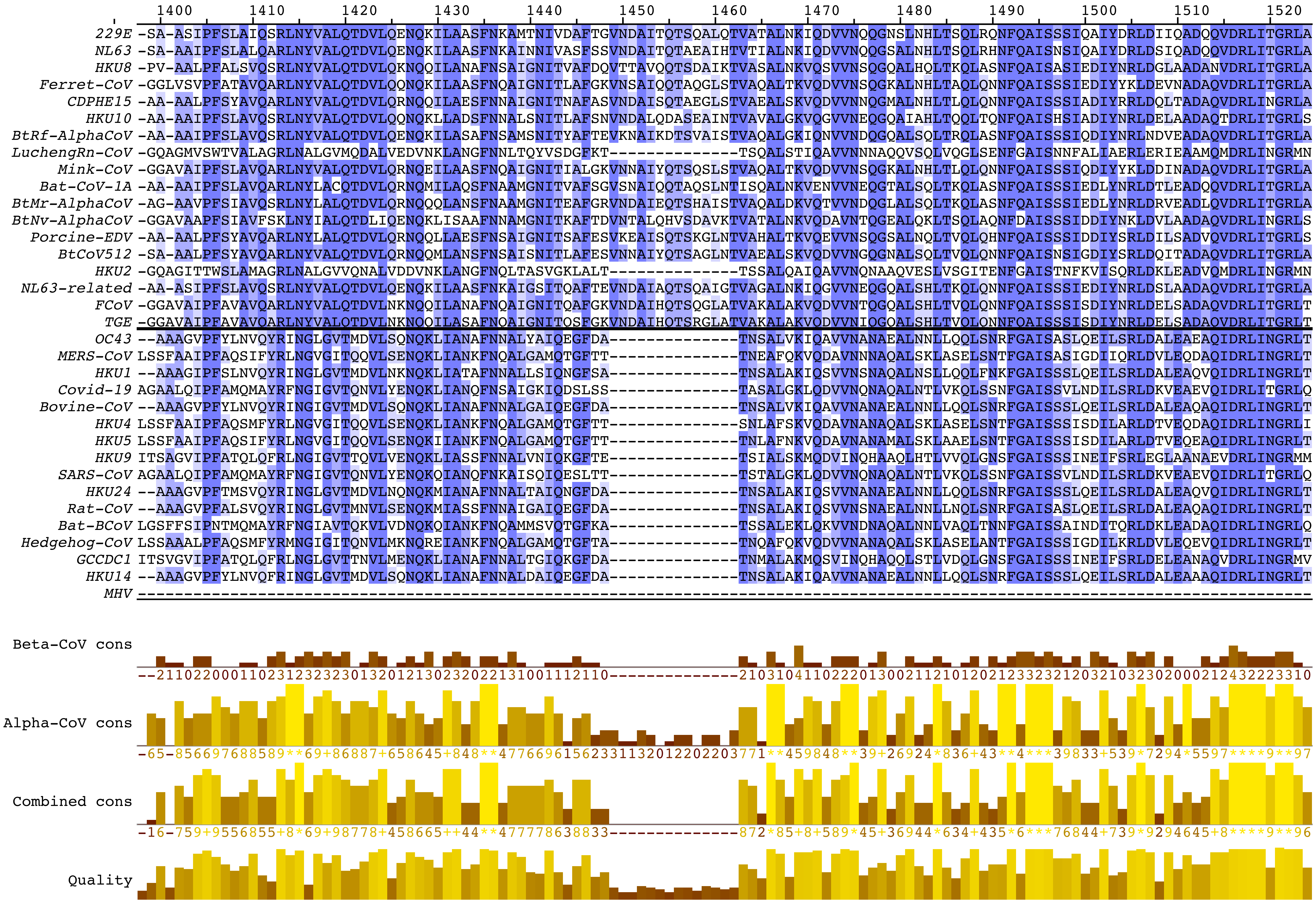

1400
1410
1420
1430
1440
1450
1460
1470
1480
1490
1500
1510
1520
229E
NL63
HKU8
Ferret-CoV
CDPHE15
HKU10
BtRf-AlphaCoV
LuchengRn-CoV
Mink-CoV
Bat-CoV-1A
BtMr-AlphaCoV
BtNv-AlphaCoV
Porcine-EDV
BtCoV512
HKU2
NL63-related
FCoV
TGE
OC43
MERS-CoV
HKU1
Covid-19
Bovine-CoV
HKU4
HKU5
HKU9
SARS-CoV
HKU24
Rat-CoV
Bat-BCoV
Hedgehog-CoV
GCCDC1
HKU14
MHV
Beta-CoV cons
Alpha-CoV cons
Combined cons
Quality

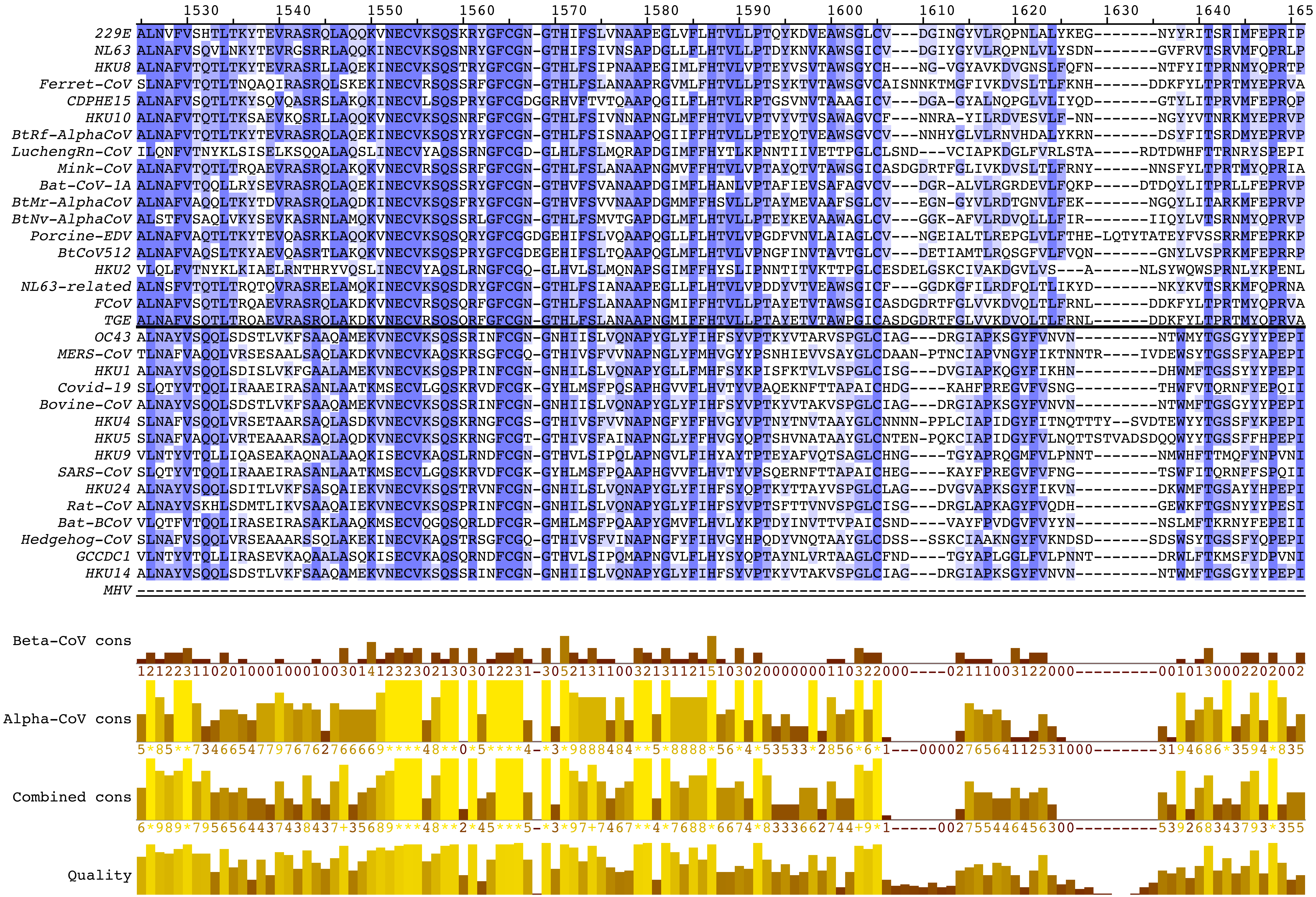
1530 1540 1550 1560 1570 1580 1590 1600 1610 1620 1630 1640 165
229E ALNVFVSHTLTKYTEVRASRQLAQQKVNECVKSQSKRYGFCGN-GTHIFSLVNAAPEGLVFLHTVLLPTQYKDVEAWSGLCV---DGINGYVLRQPNLALYKEG-------NYYRITSRIMFEPRIP
NL63 ALNAFVSQVLNKYTEVRGSRRLAQQKINECVKSQSNRYGFCGN-GTHIFSIVNSAPDGLLFLHTVLLPTDYKNVKAWSGICV---DGIYGYVLRQPNLVLYSDN-------GVFRVTSRVMFQPRLP
HKU8 ALNAFVTQTLTKYTEVRASRLLAQEKINECVKSQSTRYGFCGN-GTHLFSIPNAAPEGIMLFHTVLVPTEYVSVTAWSGYCH---NG-VGYAVKDVGNSLFQFN-------NTFYITPRNMYQPRTP
Ferret-CoV SLNAFVTQTLTNQAQIRASRQLSKEKINECVRSQSSRFGFCGN-GTHLFSLANAAPRGVMLFHTVLLPTSYKTVTAWSGVCAISNNKTMGFIVKDVSLTLFKNH------DDKFYLTPRTMYEPRVA
CDPHE15 ALNAFVSQTLTKYSQVQASRSLAKQKINECVLSQSPRYGFCGDGGRHVFTVTQAAPQGILFLHTVLRPTGSVNVTAAGICV---DGA-GYALNQPGLVLIYQD-------GTYLITPRVMFEPRQP
HKU10 ALNAFVTQTLTKSAEVKQSRLLAQQKVNECVKSQSNRFGFCGN-GTHLFSIVNNAPNGLMFFHTVLVPTVYVTVSAWAGVCF---NNRA-YILRDVESVLF-NN-------NGYYVTNRKMYEPRVP
BtRf-AlphaCoV ALNAFVTQTLTKYTEVRASRQLAQEKINECVKSQSYRYGFCGN-GTHLFSISNAAPQGIIFFHTVLLPTEYQTVEAWSGVCV---NNHYGLVLRNVHDALYKRN-------DSYFITSRDMYEPRVP
LuchengRn-CoV ILQNFVTNYKLSISELKSQQALAQSLINECVYAQSSRNGFCGD-GLHLFSLMQRAPDGIMFFHYTLKPNNTIIVETTPGLCLSND---VCIAPKDGLFVRLSTA-----RDTDWHFTTRNRYSPEPI
Mink-CoV ALNAFVTQTLTRQAEVRASRQLAKQKVNECVRSQSSRFGFCGN-GTHLFSLANAAPNGMVFFHTVLVPTAYQTVTAWSGICASDGDRTFGLIVKDVSLTLFRNY------NNSFYLTPRTMYQPRIA
Bat-CoV-1A ALNAFVTQQLLRYSEVRASRQLAQEKINECVKSQSSRYGFCGN-GTHVFSVANAAPDGIMFLHANLVPTAFIEVSAFAGVCV---DGR-ALVLRGRDEVLFQKP-----DTDQYLITPRLLFEPRVP
BtMr-AlphaCoV ALNAFVAQQLTKYTDVRASRQLAQDKINECVKSQSFRYGFCGN-GTHVFSVVNAAPDGMMFFHSVLLPTAYMEVAAFSGLCV---EGN-GYVLRDTGNVLFEK-------NGQYLITARKMFEPRVP
BtNv-AlphaCoV ALSTFVSAQLVKYSEVKASRNLAMQKVNECVKSQSSRLGFCGN-GTHLFSMVTGAPDGLMFLHTVLLPTEYKEVAAWAGLCV---GGK-AFVLRDVQLLLFIR-------IIQYLVTSRNMYQPRVP
Porcine-EDV ALNAFVAQTLTKYTEVQASRKLAQQKVNECVKSQSQRYGFCGGDGEHIFSLVQAAPQGLLFLHTVLVPGDFVNVLAIAGLCV---NGEIALTLREPGLVLFTHE-LQTYTATEYFVSSRRMFEPRKP
BtCoV512 ALNAFVAQSLTKYAEVQASRTLAKQKVNECVKSQSPRYGFCGDEGEHIFSLTQAAPQGLMFLHTVLVPNGFINVTAVTGLCV---DETIAMTLRQSGFVLFVQN-------GNYLVSPRKMFEPRRP
HKU2 VLQLFVTNYKLKIAELRNTHRYVQSLINECVYAQSLRNGFCGQ-GLHVLSLMQNAPSGIMFFHYSLIPNNTITVKTTPGLCESDELGSKCIVAKDGVLVS---A-----NLSYWQWSPRNLYKPENL
NL63-related ALNSFVTQTLTRQTQVRASRELAMQKINECVKSQSDRYGFCGN-GTHLFSIANAAPEGLLFLHTVLVPDDYVTVEAWSGICF---GGDKGFILRDFQLTLIKYD-------NKYKVTSRKMFQPRNA
FCoV ALNAFVSQTLTRQAEVRASRQLAKDKVNECVRSQSQRFGFCGN-GTHLFSLANAAPNGMIFFHTVLLPTAYETVTAWSGICASDGDRTFGLVVKDVQLTLFRNL------DDKFYLTPRTMYQPRVA
TGE ALNAFVSQTLTRQAEVRASRQLAKDKVNECVRSQSQRFGFCGN-GTHLFSLANAAPNGMIFFHTVLLPTAYETVTAWPGICASDGDRTFGLVVKDVQLTLFRNL------DDKFYLTPRTMYQPRVA
OC43 ALNAYVSQQLSDSTLVKFSAAQAMEKVNECVKSQSSRINFCGN-GNHIISLVQNAPYGLYFIHFSYVPTKYVTARVSPGLCIAG---DRGIAPKSGYFVNVN---------NTWMYTGSGYYYPEPI
MERS-CoV TLNAFVAQQLVRSESAALSAQLAKDKVNECVKAQSKRSGFCGQ-GTHIVSFVVNAPNGLYFMHVGYYPSNHIEVVSAYGLCDAAN-PTNCIAPVNGYFIKTNNTR----IVDEWSYTGSSFYAPEPI
HKU1 ALNAYVSQQLSDISLVKFGAALAMEKVNECVKSQSPRINFCGN-GNHILSLVQNAPYGLLFMHFSYKPISFKTVLVSPGLCISG---DVGIAPKQGYFIKHN---------DHWMFTGSSYYYPEPI
Covid-19 SLQTYVTQQLIRAAEIRASANLAATKMSECVLGQSKRVDFCGK-GYHLMSFPQSAPHGVVFLHVTYVPAQEKNFTTAPAICHDG----KAHFPREGVFVSNG---------THWFVTQRNFYEPQII
Bovine-CoV ALNAYVSQQLSDSTLVKFSAAQAMEKVNECVKSQSSRINFCGN-GNHIISLVQNAPYGLYFIHFSYVPTKYVTAKVSPGLCIAG---DRGIAPKSGYFVNVN---------NTWMFTGSGYYYPEPI
HKU4 SLNAFVSQQLVRSETAARSAQLASDKVNECVKSQSKRNGFCGS-GTHIVSFVVNAPNGFYFFHVGYVPTNYTNVTAAYGLCNNNN-PPLCIAPIDGYFITNQTTTY--SVDTEWYYTGSSFYKPEPI
HKU5 SLNAFVAQQLVRTEAAARSAQLAQDKVNECVKSQSKRNGFCGT-GTHIVSFAINAPNGLYFFHVGYQPTSHVNATAAYGLCNTEN-PQKCIAPIDGYFVLNQTTSTVADSDQQWYYTGSSFFHPEPI
HKU9 VLNTYVTQLLIQASEAKAQNALAAQKISECVKAQSLRNDFCGN-GTHVLSIPQLAPNGVLFIHYAYTPTEYAFVQTSAGLCHNG----TGYAPRQGMFVLPNNT-------NMWHFTTMQFYNPVNI
SARS-CoV SLQTYVTQQLIRAAEIRASANLAATKMSECVLGQSKRVDFCGK-GYHLMSFPQAAPHGVVFLHVTYVPSQERNFTTAPAICHEG----KAYFPREGVFVFNG---------TSWFITQRNFFSPQII
HKU24 ALNAYVSQQLSDITLVKFSASQAIEKVNECVKSQSTRVNFCGN-GNHILSLVQNAPYGLYFIHFSYQPTKYTTAYVSPGLCLAG---DVGVAPKSGYFIKVN---------DKWMFTGSAYYHPEPI
Rat-CoV ALNAYVSKHLSDMTLIKVSAAQAIEKVNECVKSQSPRINFCGN-GNHILSLVQNAPYGLYFIHFSYVPTSFTTVNVSPGLCISG---DRGLAPKAGYFVQDH---------GEWKFTGSNYYYPESI
Bat-BCoV VLQTFVTQQLIRASEIRASAKLAAQKMSECVQGQSQRLDFCGR-GMHLMSFPQAAPYGMVFLHVLYKPTDYINVTTVPAICSND----VAYFPVDGVFVYYN---------NSLMFTKRNYFEPEII
Hedgehog-CoV SLNAFVSQQLVRSEAAARSSQLAKEKINECVKAQSTRSGFCGQ-GTHIVSFVINAPNGFYFIHVGYHPQDYVNQTAAYGLCDSS-SSKCIAAKNGYFVKNDSD------SDSWSYTGSSFYQPEPI
GCCDC1 VLNTYVTQLLIRASEVKAQAALASQKISECVKAQSQRNDFCGN-GTHVLSIPQMAPNGVLFLHYSYQPTAYNLVRTAAGLCFND----TGYAPLGGLFVLPNNT-------DRWLFTKMSFYDPVNI
HKU14 ALNAYVSQQLSDSTLVKFSAAQAMEKVNECVKSQSSRINFCGN-GNHIISLVQNAPYGLYFIHFSYVPTKYVTAKVSPGLCIAG---DRGIAPKSGYFVNVN---------NTWMFTGSGYYYPEPI
MHV --
Beta-CoV cons
Alpha-CoV cons
Combined cons
Quality

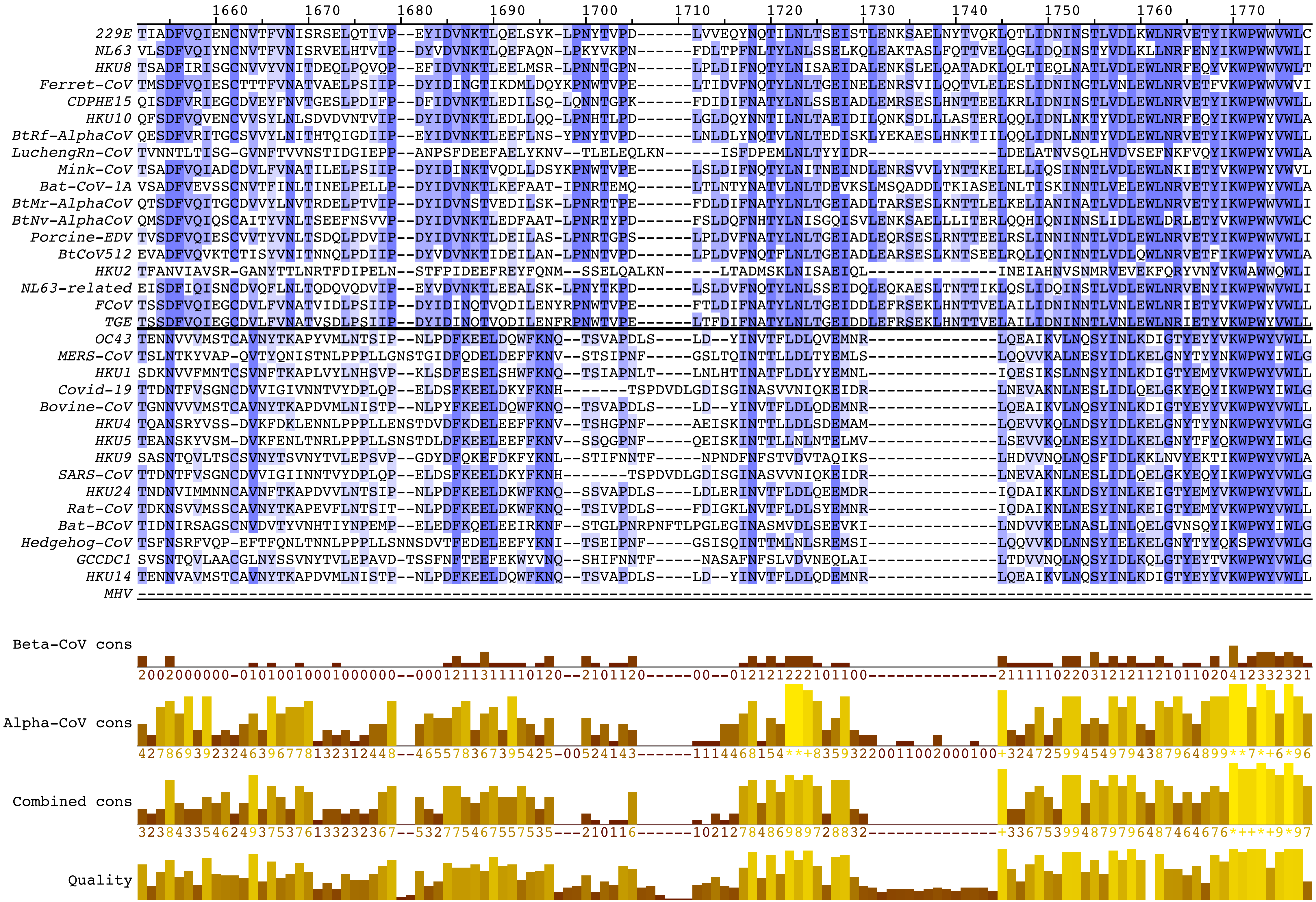
1660 1670 1680 1690 1700 1710 1720 1730 1740 1750 1760 1770
229E TIADFVQIENCNVTFVNISRSELQTIVP--EYIDVNKTLQELSYK-LPNYTVPD------LVVEQYNQTILNLTSEISTLENKSAELNYTVQKLQTLIDNINSTLVDLKWLNRVETYIKWPWWVWLC
NL63 VLSDFVQIYNCNVTFVNISRVELHTVIP--DYVDVNKTLQEFAQN-LPKYVKPN------FDLTPFNLTYLNLSSELKQLEAKTASLFQTTVELQGLIDQINSTYVDLKLLNRFENYIKWPWWVWLI
HKU8 TSADFIRISGCNVVYVNITDEQLPQVQP--EFIDVNKTLEELMSR-LPNNTGPN------LPLDIFNQTYLNISAEIDALENKSLELQATADKLQLTIEQLNATLVDLEWLNRFEQYVKWPWWVWLT
Ferret-CoV TMSDFVQIESCTTTFVNATVAELPSIIP--DYIDINGTIKDMLDQYKPNWTVPE------LTIDVFNQTYLNLTGEINELENRSVILQQTVLELESLIDNINGTLVNLEWLNRVETFVKWPWWVWVI
CDPHE15 QISDFVRIEGCDVEYFNVTGESLPDIFP--DFIDVNKTLEDILSQ-LQNNTGPK------FDIDIFNATYLNLSSEIADLEMRSESLHNTTEELKRLIDNINSTLVDLEWLNRVETYIKWPWWVWLL
HKU10 QFSDFVQVENCVVSYLNLSDVDVNTVIP--DYIDVNKTLEDLLQQ-LPNHTLPD------LGLDQYNNTILNLTAEIDILQNKSDLLASTERLQQLIDNLNNKTYVDLEWLNRFEQYIKWPWYVWLA
BtRf-AlphaCoV QESDFVRITGCSVVYLNITHTQIGDIIP--EYIDVNKTLEEFLNS-YPNYTVPD------LNLDLYNQTVLNLTEDISKLYEKAESLHNKTIILQQLIDNLNNTYVDLEWLNRVETYIKWPWYVWLL
LuchengRn-CoV TVNNTLTISG-GVNFTVVNSTIDGIEPP--ANPSFDEEFAELYKNV--TLELEQLKN------ISFDPEMLNLTYYIDR-------------LDELATNVSQLHVDVSEFNKFVQYIKWPWYVWLA
Mink-CoV TSADFVQIADCDVLFVNATILELPSIIP--DYIDINKTVQDLLDSYKPNWTVPE------LSLDIFNQTYLNITNEINDLENRSVVLYNTTKELELLIQSINNTLVDLEWLNKIETYVKWPWYVWVL
Bat-CoV-1A VSADFVEVSSCNVTFINLTINELPELLP--DYIDVNKTLKEFAAT-IPNRTEMQ------LTLNTYNATVLNLTDEVKSLMSQADDLTKIASELNLTISKINNTLVELEWLNRVETYIKWPWYVWLA
BtMr-AlphaCoV QTSDFVQITGCDVVYLNVTRDELPTVIP--DYIDVNSTVEDILSK-LPNRTTPE------FDLDIFNATYLNLTGEIADLTARSESLKNTTLELKELIANINATLVDLEWLNRVETYIKWPWWVWLI
BtNv-AlphaCoV QMSDFVQIQSCAITYVNLTSEEFNSVVP--DYIDVNKTLEDFAAT-LPNRTYPD------FSLDQFNHTYLNISGQISVLENKSAELLLITERLQQHIQNINNSLIDLEWLDRLETYVKWPWWVWLC
Porcine-EDV TVSDFVQIESCVVTYVNLTSDQLPDVIP--DYIDVNKTLDEILAS-LPNRTGPS------LPLDVFNATYLNLTGEIADLEQRSESLRNTTEELRSLINNINNTLVDLEWLNRVETYIKWPWWVWLI
BtCoV512 EVADFVQVKTCTISYVNITNNQLPDIIP--DYVDVNKTIDEILAN-LPNNTVPD------LPLDVFNQTFLNLTGEIADLEARSESLKNTSEELRQLIQNINNTLVDLQWLNRVETFIKWPWYVWLA
HKU2 TFANVIAVSR-GANYTTLNRTFDIPELN--STFPIDEEFREYFQNM--SSELQALKN------LTADMSKLNISAEIQL-------------INEIAHNVSNMRVEVEKFQRYVNYVKWAWWQWLI
NL63-related EISDFIQISNCDVQFLNLTQDQVQDVIP--EYVDVNKTLEEALSK-LPNYTKPD------LSLDVFNQTYLNLSSEIDQLEQKAESLTNTTIKLQSLIDQINSTLVDLEWLNRVENYIKWPWWVWLI
FCoV TSSDFVQIEGCDVLFVNATVIDLPSIIP--DYIDINQTVQDILENYRPNWTVPE------FTLDIFNATYLNLTGEIDDLEFRSEKLHNTTVELAILIDNINNTLVNLEWLNRIETYVKWPWYVWLL
TGE TSSDFVQIEGCDVLFVNATVSDLPSIIP--DYIDINQTVQDILENFRPNWTVPE------LTFDIFNATYLNLTGEIDDLEFRSEKLHNTTVELAILIDNINNTLVNLEWLNRIETYVKWPWYVWLL
OC43 TENNVVVMSTCAVNYTKAPYVMLNTSIP--NLPDFKEELDQWFKNQ--TSVAPDLS----LD--YINVTFLDLQVEMNR--------------LQEAIKVLNQSYINLKDIGTYEYYVKWPWYVWLL
MERS-CoV TSLNTKYVAP-QVTYQNISTNLPPPLLGNSTGIDFQDELDEFFKNV--STSIPNF-----GSLTQINTTLLDLTYEMLS--------------LQQVVKALNESYIDLKELGNYTYYNKWPWYIWLG
HKU1 SDKNVVFMNTCSVNFTKAPLVYLNHSVP--KLSDFESELSHWFKNQ--TSIAPNLT----LNLHTINATFLDLYYEMNL--------------IQESIKSLNNSYINLKDIGTYEMYVKWPWYVWLL
Covid-19 TTDNTFVSGNCDVVIGIVNNTVYDPLQP--ELDSFKEELDKYFKNH-------TSPDVDLGDISGINASVVNIQKEIDR--------------LNEVAKNLNESLIDLQELGKYEQYIKWPWYIWLG
Bovine-CoV TGNNVVVMSTCAVNYTKAPDVMLNISTP--NLPYFKEELDQWFKNQ--TSVAPDLS----LD--YINVTFLDLQDEMNR--------------LQEAIKVLNQSYINLKDIGTYEYYVKWPWYVWLL
HKU4 TQANSRYVSS-DVKFDKLENNLPPPLLENSTDVDFKDELEEFFKNV--TSHGPNF-----AEISKINTTLLDLSDEMAM--------------LQEVVKQLNDSYIDLKELGNYTYYNKWPWYVWLG
HKU5 TEANSKYVSM-DVKFENLTNRLPPPLLSNSTDLDFKEELEEFFKNV--SSQGPNF-----QEISKINTTLLNLNTELMV--------------LSEVVKQLNESYIDLKELGNYTFYQKWPWYIWLG
HKU9 SASNTQVLTSCSVNYTSVNYTVLEPSVP--GDYDFQKEFDKFYKNL--STIFNNTF-----NPNDFNFSTVDVTAQIKS--------------LHDVVNQLNQSFIDLKKLNVYEKTIKWPWYVWLA
SARS-CoV TTDNTFVSGNCDVVIGIINNTVYDPLQP--ELDSFKEELDKYFKNH-------TSPDVDLGDISGINASVVNIQKEIDR--------------LNEVAKNLNESLIDLQELGKYEQYIKWPWYVWLG
HKU24 TNDNVIMMNNCAVNFTKAPDVVLNTSIP--NLPDFKEELDKWFKNQ--SSVAPDLS----LDLERINVTFLDLQEEMDR--------------IQDAIKKLNDSYINLKEIGTYEMYVKWPWYVWLL
Rat-CoV TDKNSVVMSSCAVNYTKAPEVFLNTSIT--NLPDFKEELDKWFKNQ--TSIVPDLS----FDIGKLNVTFLDLSYEMNR--------------IQDAIKNLNESYINLKEIGTYEMYVKWPWYVWLL
Bat-BCoV TIDNIRSAGSCNVDVTYVNHTIYNPEMP--ELEDFKQELEEIRKNF--STGLPNRPNFTLPGLEGINASMVDLSEEVKI--------------LNDVVKELNASLINLQELGVNSQYIKWPWYIWLG
Hedgehog-CoV TSFNSRFVQP-EFTFQNLTNNLPPPLLSNNSDVTFEDELEEFYKNI--TSEIPNF-----GSISQINTTMLNLSREMSI--------------LQQVVKDLNNSYIELKELGNYTYYQKSPWYVWLG
GCCDC1 SVSNTQVLAACGLNYSSVNYTVLEPAVD-TSSFNFTEEFEKWYVNQ--SHIFNNTF-----NASAFNFSLVDVNEQLAI--------------LTDVVNQLNQSYIDLKQLGTYEYTVKWPWYVWLG
HKU14 TENNVAVMSTCAVNYTKAPDVMLNISTP--NLPDFKEELDQWFKNQ--TSVAPDLS----LD--YINVTFLDLQDEMNR--------------LQEAIKVLNQSYINLKDIGTYEYYVKWPWYVWLL
MHV
Beta-CoV cons
2002000000-0101001000100000--000121131111012 0--2101120-----00--012121222101100--------------21111102203121211210110204123323 21
Alpha-CoV cons
4278693923246396778132312448--4655783673954 25--00524143------1114468154**+835932200110020001 00+32472599454979438796489 9**7*+6*96
Combined cons
3238433546249375376133232367--532775467557535---210116------10212784869897 28832--------------+33675399487979648746467 6*+++*+9*97
Quality

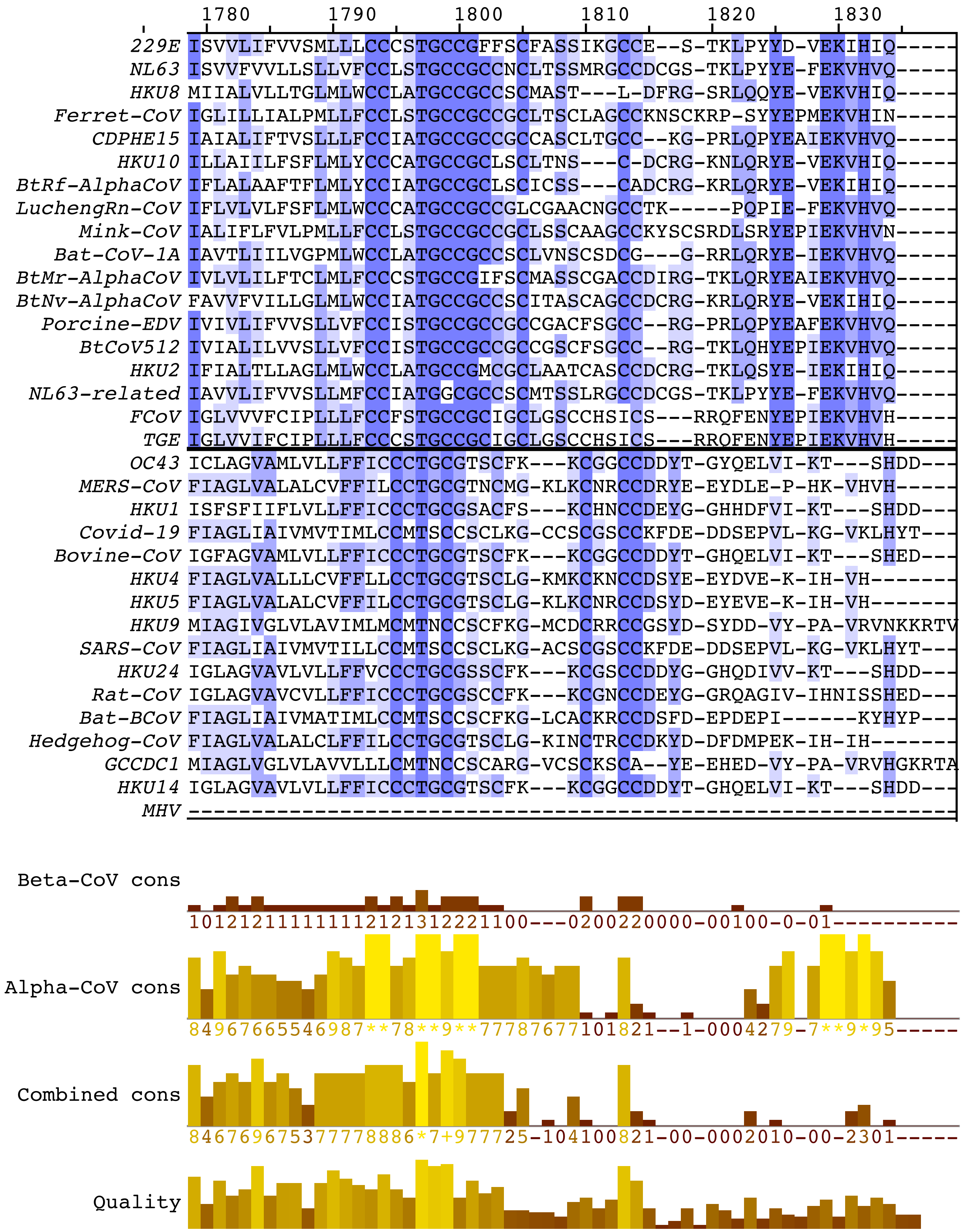

1780 1790 1800 1810 1820 1830
229E ISVVLIFVVSMLLLCCCSTGCCGFFSCFASSIKGCCE--S-TKLPYYD-VEKIHIQ-----
NL63 ISVVFVVLLSLLVFCCLSTGCCGCCNCLTSSMRGCCDCGS-TKLPYYE-FEKVHVQ-----
HKU8 MIIALVLLTGLMLWCCLATGCCGCCSCMAST---L-DFRG-SRLQQYE-VEKVHIQ-----
Ferret-CoV IGLILLIALPMLLFCCLSTGCCGCCGCLTSCLAGCCKNSCKRP-SYYEPMEKVHIN-----
CDPHE15 IAIALIFTVSLLLFCCIATGCCGCCGCCASCLTGCC--KG-PRLQPYEAIEKVHVQ-----
HKU10 ILLAIILFSFLMLYCCCATGCCGCLSCLTNS---C-DCRG-KNLQRYE-VEKVHIQ-----
BtRf-AlphaCoV IFLALAAFTFLMLYCCIATGCCGCLSCICSS---CADCRG-KRLQRYE-VEKIHIQ-----
LuchengRn-CoV IFLVLVLFSFLMLWCCCATGCCGCCGLCGAACNGCCTK-----PQPIE-FEKVHVQ-----
Mink-CoV IALIFLFVLPMLLFCCLSTGCCGCCGCLSSCAAGCCKYSCSRDLSRYEPIEKVHVN-----
Bat-CoV-1A IAVTLIILVGPMLWCCLATGCCGCCSCLVNSCSDCG---G-RRLQRYE-IEKVHVQ-----
BtMr-AlphaCoV IVLVLILFTCLMLFCCCSTGCCGIFSCMASSCGACCDIRG-TKLQRYEAIEKVHVQ-----
BtNv-AlphaCoV FAVVFVILLGLMLWCCIATGCCGCCSCITASCAGCCDCRG-KRLQRYE-VEKIHIQ-----
Porcine-EDV IVIVLIFVVSLLVFCCISTGCCGCCGCCGACFSGCC--RG-PRLQPYEAFEKVHVQ-----
BtCoV512 IVIALILVVSLLVFCCISTGCCGCCGCCGSCFSGCC--RG-TKLQHYEPIEKVHVQ-----
HKU2 IFIALTLLAGLMLWCCLATGCCGMCGCLAATCASCCDCRG-TKLQSYE-IEKIHIQ-----
NL63-related IAVVLIFVVSLLMFCCIATGGCGCCSCMTSSLRGCCDCGS-TKLPYYE-FEKVHVQ-----
FCoV IGLVVVFCIPLLLFCCFSTGCCGCIGCLGSCCHSICS---RRQFENYEPIEKVHVH-----
TGE IGLVVIFCIPLLLFCCCSTGCCGCIGCLGSCCHSICS---RRQFENYEPIEKVHVH-----
OC43 ICLAGVAMLVLLFFICCCTGCGTSCFK---KCGGCCDDYT-GYQELVI-KT---SHDD---
MERS-CoV FIAGLVALALCVFFILCCTGCGTNCMG-KLKCNRCCDRYE-EYDLE-P-HK-VHVH-----
HKU1 ISFSFIIFLVLLFFICCCTGCGSACFS---KCHNCCDEYG-GHHDFVI-KT---SHDD---
Covid-19 FIAGLIAIVMVTIMLCCMTSCCSCLKG-CCSCGSCCKFDE-DDSEPVL-KG-VKLHYT---
Bovine-CoV IGFAGVAMLVLLFFICCCTGCGTSCFK---KCGGCCDDYT-GHQELVI-KT---SHED---
HKU4 FIAGLVALLLCVFFLLCCTGCGTSCLG-KMKCKNCCDSYE-EYDVE-K-IH-VH-------
HKU5 FIAGLVALALCVFFILCCTGCGTSCLG-KLKCNRCCDSYD-EYEVE-K-IH-VH-------
HKU9 MIAGIVGLVLAVIMLMCMTNCCSCFKG-MCDCRRCCGSYD-SYDD-VY-PA-VRVNKKRTV
SARS-CoV FIAGLIAIVMVTILLCCMTSCCSCLKG-ACSCGSCCKFDE-DDSEPVL-KG-VKLHYT---
HKU24 IGLAGVAVLVLLFFVCCCTGCGSSCFK---KCGSCCDDYG-GHQDIVV-KT---SHDD---
Rat-CoV IGLAGVAVCVLLFFICCCTGCGSCCFK---KCGNCCDEYG-GRQAGIV-IHNISSHED---
Bat-BCoV FIAGLIAIVMATIMLCCMTSCCSCFKG-LCACKRCCDSFD-EPDEPI------KYHYP---
Hedgehog-CoV FIAGLVALALCLFFILCCTGCGTSCLG-KINCTRCCDKYD-DFDMPEK-IH-IH-------
GCCDC1 MIAGLVGLVLAVVLLLCMTNCCSCARG-VCSCKSCA--YE-EHED-VY-PA-VRVHGKRTA
HKU14 IGLAGVAVLVLLFFICCCTGCGTSCFK---KCGGCCDDYT-GHQELVI-KT---SHDD---
MHV ---
Beta-CoV cons
10121211111111212131222110---0200220000-00100-0-01----------
Alpha-CoV cons
84967665546987**78**9**77787677101821--1-0004279-7**9*95-----
Combined cons
846769675377778886*7+977725-104100821-00-0002010-00-2301-----
Quality

Envelope (E) protein

```
                       10        20        30        40        50        60        70        80
SARS-CoV-2   1 --MYSFVSEETGTLIVNSVLLFLAFVVFLLVTLAILTALRLCAYCCNIVNVSLVKPSFYVYSRVKNLNSS--R-VPD----LLV-
SARS-CoV     1 --MYSFVSEETGTLIVNSVLLFLAFVVFLLVTLAILTALRLCAYCCNIVNVSLVKPTVYVYSRVKNLNSS--EGVPD----LLV-
HKU1         1 --MVDLFFNDTAWYIGQILVLVLFCLISLIFVVAFLATIKLCMQLCGFCNFFIISPSAYVYKRGMQLYKSYSEQVIPPTSDYLI-
OC43         1 MFMADAYLADTVWYVGQIIFIVAICLLVTIVVVAFLATFKLCIQLCGMCNTLVLSPSIYVFNRGRQFYEFYNDVKP-PVLDVDDV
MERS-CoV     1 --MLPFVQERIGLFIVNFFIFTVVCAITLLVCMAFLTATRLCVQCMTGFNTLLVQPALYLYNTGRSVYVKFQDSKPPLPPDEWV-
229E         1 --MFLKLV-DDHALIVNVLLWCVVLIVILLVCITIIKLIKLCFTCHMFCNRTVYGPIKNVYHI----YQSYMHIDPFP-KRVIDF
NL63         1 --MFLRLI-DDNGIVLNSILWLLVMIFFFVLAMTFIKLIQLCFTCHYFFSRTLYQPVYKIFLA----YQDYMQIAPVP-AEVLNV
```

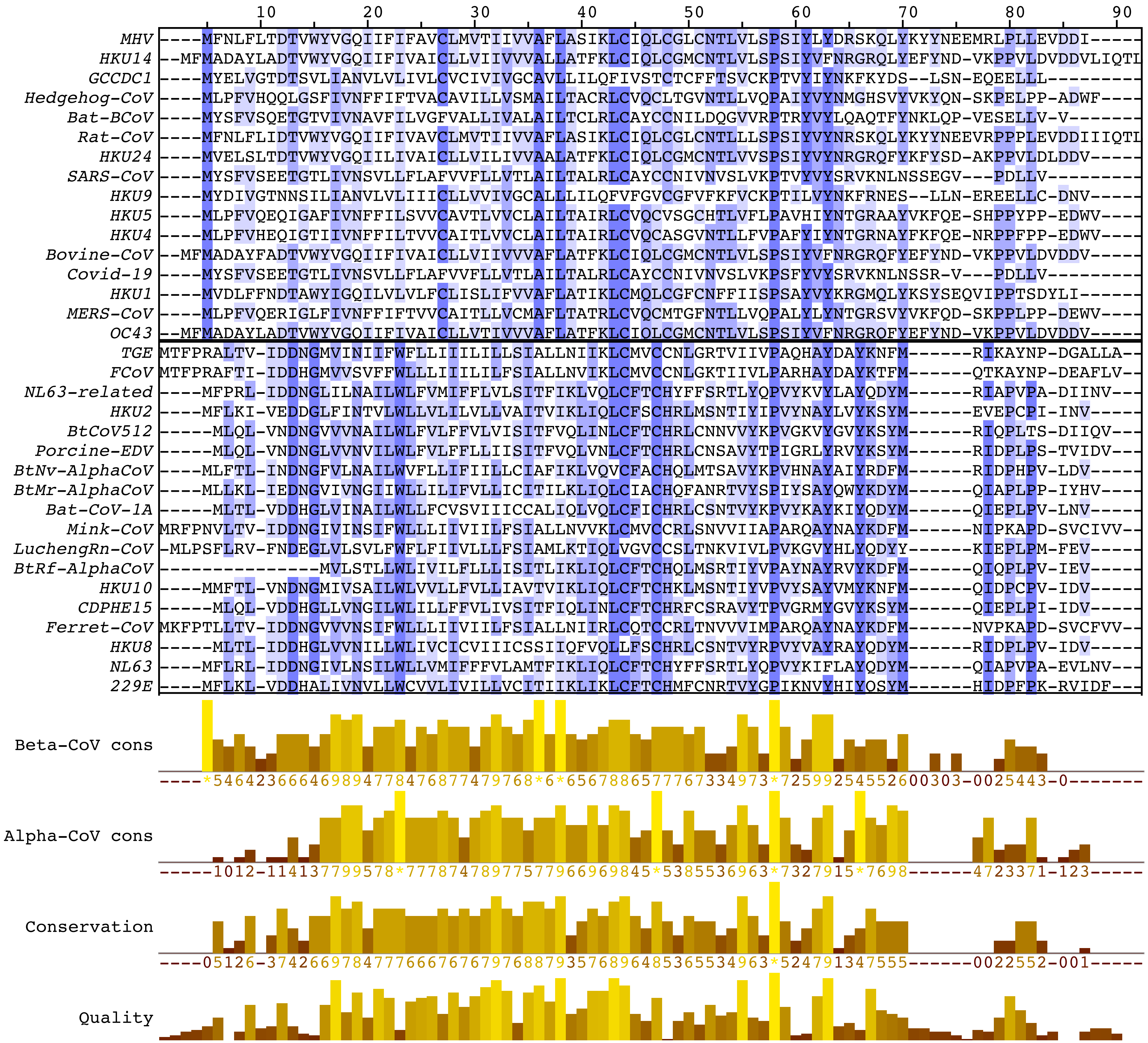

10 20 30 40 50 60 70 80 90
MHV ----MFNLFLTDTVWYVGQIIFIFAVCLMVTIIVVAFLASIKLCIQLCGLCNTLVLSPSIYLYDRSKQLYKYYNEEMRLPLLEVDDI-----
HKU14 --MFMADAYLADTVWYVGQIIFIVAICLLVIIVVVALLATFKLCIQLCGMCNTLVLSPSIYVFNRGRQLYEFYND-VKPPVLDVDDVLIQTL
GCCDC1 ----MYELVGTDTSVLIANVLVLIVLCVCIVIVGCAVLLILQFIVSTCTCFFTSVCKPTVYIYNKFKYDS--LSN-EQEELLL---------
Hedgehog-CoV ----MLPFVHQQLGSFIVNFFIFTVACAVILLVSMAILTACRLCVQCLTGVNTLLVQPAIYVYNMGHSVYVKYQN-SKPELPP-ADWF----
Bat-BCoV ----MYSFVSQETGTVIVNAVFILVGFVALLIVALAILTCLRLCAYCCNILDQGVVRPTRYVYLQAQTFYNKLQP-VESELLV-V-------
Rat-CoV ----MFNLFLIDTVWYVGQIIFIVAVCLMVTIIVVAFLASIKLCIQLCGLCNTLLLSPSIYVYNRSKQLYKYYNEEVRPPPLEVDDIIIQTL
HKU24 ----MVELSLTDTVWYVGQIILIVAICLLVILIVVAALATFKLCIQLCGMCNTLVVSPSIYVYNRGRQFYKFYSD-AKPPVLDLDDV-----
SARS-CoV ----MYSFVSEETGTLIVNSVLLFLAFVVFLLVTLAILTALRLCAYCCNIVNVSLVKPTVYVYSRVKNLNSSEGV---PDLLV---------
HKU9 ----MYDIVGTNNSILIANVLVLIIICLLVVIVGCALLLILQFVFGVCGFVFKFVCKPTILVYNKFRNES--LLN-EREELLC-DNV-----
HKU5 ----MLPFVQEQIGAFIVNFFILSVVCAVTLVVCLAILTAIRLCVQCVSGCHTLVFLPAVHIYNTGRAAYVKFQE-SHPPYPP-EDWV----
HKU4 ----MLPFVHEQIGTIIVNFFILTVVCAITLVVCLAILTAIRLCVQCASGVNTLLFVPAFYIYNTGRNAYFKFQE-NRPPFPP-EDWV----
Bovine-CoV --MFMADAYFADTVWYVGQIIFIVAICLLVIIVVVAFLATFKLCIQLCGMCNTLVLSPSIYVFNRGRQFYEFYND-VKPPVLDVDDV-----
Covid-19 ----MYSFVSEETGTLIVNSVLLFLAFVVFLLVTLAILTALRLCAYCCNIVNVSLVKPSFYVYSRVKNLNSSR-V---PDLLV---------
HKU1 ----MVDLFFNDTAWYIGQILVLVLFCLISLIFVVAFLATIKLCMQLCGFCNFFIISPSAYVYKRGMQLYKSYSEQVIPPTSDYLI------
MERS-CoV ----MLPFVQERIGLFIVNFFIFTVVCAITLLVCMAFLTATRLCVQCMTGFNTLLVQPALYLYNTGRSVYVKFQD-SKPPLPP-DEWV----
OC43 --MFMADAYLADTVWYVGQIIFIVAICLLVTIVVVAFLATFKLCIQLCGMCNTLVLSPSIYVFNRGRQFYEFYND-VKPPVLDVDDV-----
TGE MTFPRALTV-IDDNGMVINIIFWFLLIIILILLSIALLNIIKLCMVCCNLGRTVIIVPAQHAYDAYKNFM------RIKAYNP-DGALLA--
FCoV MTFPRAFTI-IDDHGMVVSVFFWLLLIIILILFSIALLNVIKLCMVCCNLGKTIIVLPARHAYDAYKTFM------QTKAYNP-DEAFLV--
NL63-related ----MFPRL-IDDNGLILNAILWLFVMIFFLVLSITFIKLVQLCFTCHYFFSRTLYQPVYKVYLAYQDYM------RIAPVPA-DIINV---
HKU2 ----MFLKI-VEDDGLFINTVLWLLVLILVLLVAITVIKLIQLCFSCHRLMSNTIYIPVYNAYLVYKSYM------EVEPCPI-INV-----
BtCoV512 -----MLQL-VNDNGVVVNAILWLFVLFFVLVISITFVQLINLCFTCHRLCNNVVYKPVGKVYGVYKSYM------RIQPLTS-DIIQV---
Porcine-EDV -----MLQL-VNDNGLVVNVILWLFVLFFLLIISITFVQLVNLCFTCHRLCNSAVYTPIGRLYRVYKSYM------RIDPLPS-TVIDV---
BtNv-AlphaCoV ----MLFTL-INDNGFVLNAILWVFLLIFIILLCIAFIKLVQVCFACHQLMTSAVYKPVHNAYAIYRDFM------RIDPHPV-LDV-----
BtMr-AlphaCoV ----MLLKL-IEDNGVIVNGIIWLLILIFVLLICITILKLIQLCIACHQFANRTVYSPIYSAYQWYKDYM------QIAPLPP-IYHV----
Bat-CoV-1A -----MLTL-VDDHGLVINAILWLLFCVSVIIICCALIQLVQLCFICHRLCSNTVYKPVYKAYKIYQDYM------QIEPLPV-LNV-----
Mink-CoV MRFPNVLTV-IDDNGIVINSIFWLLLIIVIILFSIALLNVVKLCMVCCRLSNVVIIAPARQAYNAYKDFM------NIPKAPD-SVCIVV--
LuchengRn-CoV -MLPSFLRV-FNDEGLVLSVLFWFLFIIVLLLFSIAMLKTIQLVGVCCSLTNKVIVLPVKGVYHLYQDYY------KIEPLPM-FEV-----
BtRf-AlphaCoV ---------------MVLSTLLWLIVILFLLLISITLIKLIQLCFTCHQLMSRTIYVPAYNAYRVYKDFM------QIQPLPV-IEV-----
HKU10 ----MMFTL-VNDNGMIVSAILWLVVLLFVLLIAVTVIKLIQLCFTCHKLMSNTIYVPVYSAYVMYKNFM------QIDPCPV-IDV-----
CDPHE15 ----MLQL-VDDHGLLVNGILWLILLFFVLLIVSITFIQLINLCFTCHRFCSRAVYTPVGRMYGVYKSYM------QIEPLPI-IDV-----
Ferret-CoV MKFPTLLTV-IDDNGVVVNSIFWLLLIIVIILFSIALLNIIRLCQTCCRLTNVVVIMPARQAYNAYKDFM------NVPKAPD-SVCFVV--
HKU8 -----MLTL-IDDHGLVVNILLWLIVCICVIIICSSIIQFVQLLFSCHRLCSNTVYRPVYVAYRAYQDYM------RIDPLPV-IDV-----
NL63 ----MFLRL-IDDNGIVLNSILWLLVMIFFFVLAMTFIKLIQLCFTCHYFFSRTLYQPVYKIFLAYQDYM------QIAPVPA-EVLNV---
229E ----MFLKL-VDDHALIVNVLLWCVVLIVILLVCITIIKLIKLCFTCHMFCNRTVYGPIKNVYHIYQSYM------HIDPFPK-RVIDF---
Beta-CoV cons
----*546423666469894778476877479768*6*6567886577767334973*72599254552600303-0025443-0-------
Alpha-CoV cons
-----1012-114137799578*77787478977577966969845*5385536963*7327915*7698------4723371-123------
Conservation
----05126-3742669784777666767679768879357689648536553496 3*524791347555------0022552-001------
Quality

Membrane (M) Protein

```
                          10        20        30        40        50        60        70        80        90
SARS-CoV-2   1 -----MADSNGTITVEELKKLLEQWNLVIGFLFLTWICLLQFAYANRNRFLYIIKLIFLWLLWPVTLACFVLAAV--YRINWITGGIAIAM
SARS-CoV     1 ------MADNGTITVEELKQLLEQWNLVIGFLFLAWIMLLQFAYSNRNRFLYIIKLVFLWLLWPVTLACFVLAAV--YRINWVTGGIAIAM
HKU1         1 ----MNKSFLPQFTSDQAVTFLKEWNFSLGVILLFITIILQFGYTSRSMFVYLIKMIILWLMWPLTITLTIFNCF--YALNNAFLAFSIVF
OC43         1 MSSKTTPAPVYIWTADEAIKFLKEWNFSLGIILLFITIILQFGYTSRSMFVYVIKMIILWLMWPLTIILTIFNCV--YALNNVYLGLSIVF
MERS-CoV     1 ------MSNMTQLTEAQIIAIIKDWNFAWSLIFLLITIVLQYGYPSRSMTVYVFKMFVLWLLWPSSMALSIFSAV--YPIDLASQIISGIV
229E         1 -------MSND-NCTGDIVTHLKNWNFGWNVILTIFIVILQFGHYKYSRLFYGLKMLVLWLLWPLVLALSIFDTWANWDSNWAFVAFSFLM
NL63         1 -------MSNSSVPLLEVYVHLRNWNFSWNLILTLFIVVLQYGHYKYSRLLYGLKMSVLWCLWPLVLALSIFDCFVNFNVDWVFFGFSILM

                         100       110       120       130       140       150       160       170       180
SARS-CoV-2  85 ACLVGLMWLSYFIASFRLFARTRSMWSFNPETNILLNVPLHGTILTRPLLESELVIGAVILRGHLRIAGHHLGRC-DIKDLPKEITVATSR
SARS-CoV    84 ACIVGLMWLSYFVASFRLFARTRSMWSFNPETNILLNVPLRGTIVTRPLMESELVIGAVIIRGHLRMAGHSLGRC-DIKDLPKEITVATSR
HKU1        86 TIISIVIWILYFVNSIRLFIRTGSWWSFNPETNNLMCIDMKGKMFVRPVIEDYHTLTATVIRGHLYIQGVKLGTGYTLSDLPVYVTVAKVQ
OC43        90 TIVAIIMWIVYFVNSIRLFIRTGSFWSFNPETNNLMCIDMKGTMYVRPIIEDYHTLTVTIIRGHLYIQGIKLGTGYSLADLPAYMTVAKVT
MERS-CoV    84 AAVSAMMWISYFVQSIRLFMRTGSWWSFNPETNCLLNVPFGGTTVVRPLVEDSTSVTAVVTNGHLKMAGMHFGAC-DYDRLPNEVTVAKPN
229E        84 AVSTLVMWVMYFANSFRLFRRARTFWAWNPEVNAITVTTVLGQTYYQPIQQAPTGITVTLLSGVLYVDGHRLASGVQVHNLPEYMTVAVPS
NL63        85 SIITLCLWVMYFVNSFRLWRRVKTFWAFNPETNAIISLQVYGHNYYLPVMAAPTGVTLTLLSGVLLVDGHKIATRVQVGQLPKYVIVATPS

                        190       200       210       220       230
SARS-CoV-2 175 TLSYYKLG-ASQRVAGDSGFAAYSRYRIGNYKLNTDHSSSSDNIALLVQ--
SARS-CoV   174 TLSYYKLG-ASQRVGTDSGFAAYNRYRIGNYKLNTDHAGSNDNIALLVQ--
HKU1       177 VLCTYKRA-FLDKLDVNSGFAVFVKSKVGNYRLPSS-KPSGMDTALLRA--
OC43       181 HLCTYKRG-FLDRISDTSGFAVYVKSKVGNYRLPSTQKGSGMDTALLRNNI
MERS-CoV   174 VLIALKMV-KRQSYGTNSGVAIYHRYKAGNYRSPPITA--DIELALLRA--
229E       175 TTIIYSRVGRSVNSQNSTGWVFYVRVKHGDFSAVSSPMSNMTENERLLHFF
NL63       176 TTIVCDRVGRSVNETSQTGWAFYVRAKHGDFSGVASQEGVLSEREKLLHLI
```

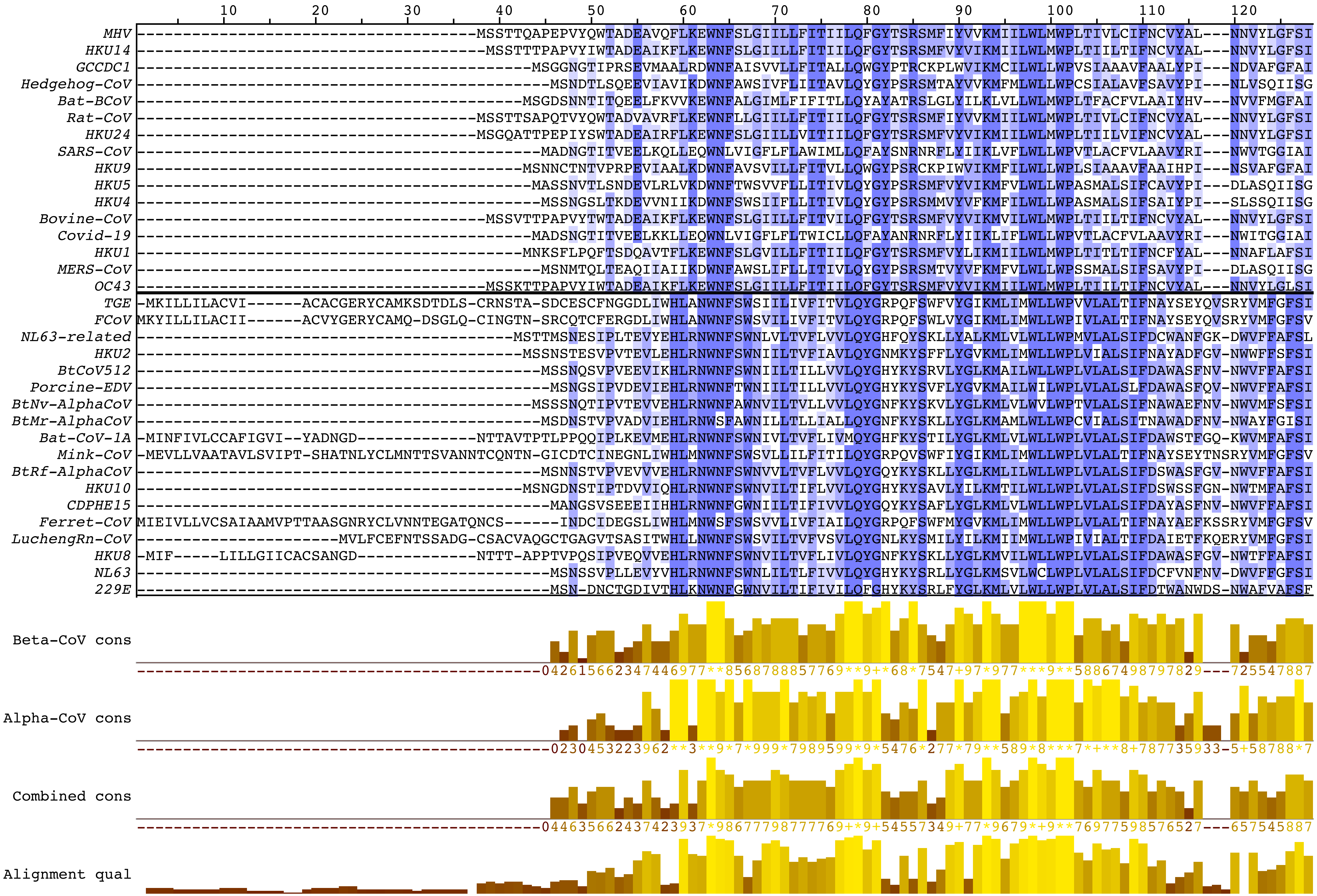
10
20
30
40
50
60
70
80
90
100
110
120
MHV
HKU14
GCCDC1
Hedgehog-CoV
Bat-BCoV
Rat-CoV
HKU24
SARS-CoV
HKU9
HKU5
HKU4
Bovine-CoV
Covid-19
HKU1
MERS-CoV
OC43
TGE
FCoV
NL63-related
HKU2
BtCoV512
Porcine-EDV
BtNv-AlphaCoV
BtMr-AlphaCoV
Bat-CoV-1A
Mink-CoV
BtRf-AlphaCoV
HKU10
CDPHE15
Ferret-CoV
LuchengRn-CoV
HKU8
NL63
229E
Beta-CoV cons
Alpha-CoV cons
Combined cons
Alignment qual

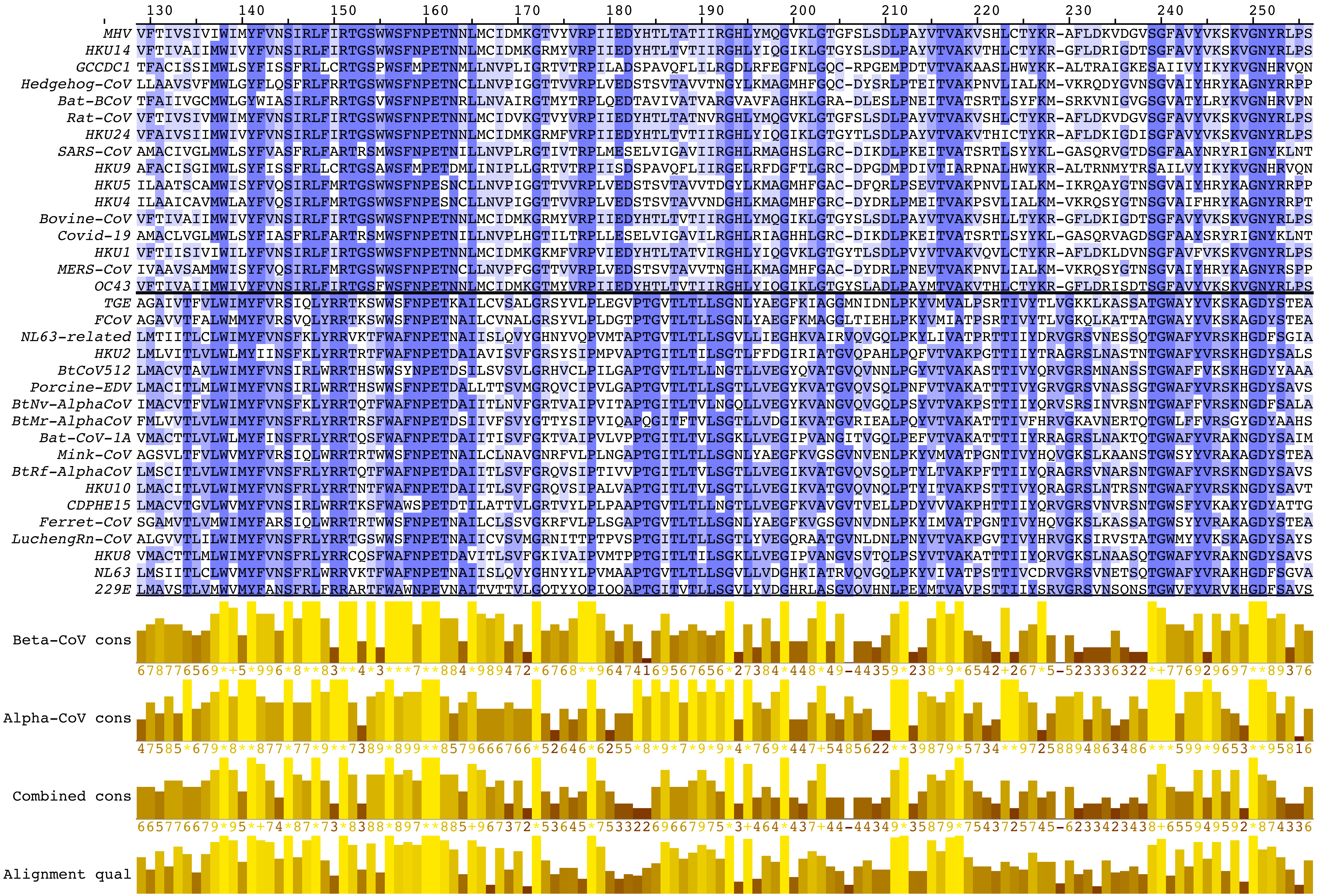

130 140 150 160 170 180 190 200 210 220 230 240 250
MHV VFTIVSIVIWIMYFVNSIRLFIRTGSWWSFNPETNNLMCIDMKGTVYVRPIIEDYHTLTATIIRGHLYMQGVKLGTGFSLSDLPAYVTVAKVSHLCTYKR-AFLDKVDGVSGFAVYVKSKVGNYRLPS
HKU14 VFTIVAIIMWIVYFVNSIRLFIRTGSWWSFNPETNNLMCIDMKGRMYVRPIIEDYHTLTVTIIRGHLYIQGIKLGTGYSLSDLPAYVTVAKVTHLCTYKR-GFLDRIGDTSGFAVYVKSKVGNYRLPS
GCCDC1 TFACISSIMWLSYFISSFRLLCRTGSPWSFMPETNMLLNVPLIGRTVTRPILADSPAVQFLILRGDLRFEGFNLGQC-RPGEMPDTVTVAKAASLHWYKK-ALTRAIGKESAIIVYIKYKVGNHRVQN
Hedgehog-CoV LLAAVSVFMWLGYFLQSFRLFRRTGSWWSFNPETNCLLNVPIGGTTVVRPLVEDSTSVTAVVTNGYLKMAGMHFGQC-DYSRLPTEITVAKPNVLIALKM-VKRQDYGVNSGVAIYHRYKAGNYRRPP
Bat-BCoV TFAIIVGCMWLGYWIASIRLFRRTGSVWSFNPETNRLLNVAIRGTMYTRPLQEDTAVIVATVARGVAVFAGHKLGRA-DLESLPNEITVATSRTLSYFKM-SRKVNIGVGSGVATYLRYKVGNHRVPN
Rat-CoV VFTIVSIVMWIMYFVNSIRLFIRTGSWWSFNPETNNLMCIDVKGTVYVRPIIEDYHTLTATNVRGHLYMQGVKLGTGFSLSDLPAYVTVAKVSHLCTYKR-AFLDKVDGVSGFAVYVKSKVGNYRLPS
HKU24 VFAIVSIIMWIVYFVNSIRLFIRTGSWWSFNPETNNLMCIDMKGRMFVRPIIEDYHTLTVTIIRGHLYIQGIKLGTGYTLSDLPAYVTVAKVTHICTYKR-AFLDKIGDISGFAVYVKSKVGNYRLPS
SARS-CoV AMACIVGLMWLSYFVASFRLFARTRSMWSFNPETNILLNVPLRGTIVTRPLMESELVIGAVIIRGHLRMAGHSLGRC-DIKDLPKEITVATSRTLSYYKL-GASQRVGTDSGFAAYNRYRIGNYKLNT
HKU9 AFACISGIMWLSYFISSFRLLCRTGSAWSFMPETDMLINIPLLGRTVTRPIISDSPAVQFLIIRGELRFDGFTLGRC-DPGDMPDIVTIARPNALHWYKR-ALTRNMYTRSAILVYIKYKVGNHRVQN
HKU5 ILAATSCAMWISYFVQSIRLFMRTGSWWSFNPESNCLLNVPIGGTTVVRPLVEDSTSVTAVVTDGYLKMAGMHFGAC-DFQRLPSEVTVAKPNVLIALKM-IKRQAYGTNSGVAIYHRYKAGNYRRPP
HKU4 ILAAICAVMWLAYFVQSIRLFMRTGSWWSFNPESNCLLNVPIGGTTVVRPLVEDSTSVTAVVNDGHLKMAGMHFGRC-DYDRLPMEITVAKPSVLIALKM-VKRQSYGTNSGVAIFHRYKAGNYRRPT
Bovine-CoV VFTIVAIIMWIVYFVNSIRLFIRTGSWWSFNPETNNLMCIDMKGRMYVRPIIEDYHTLTVTIIRGHLYMQGIKLGTGYSLSDLPAYVTVAKVSHLLTYKR-GFLDKIGDTSGFAVYVKSKVGNYRLPS
Covid-19 AMACLVGLMWLSYFIASFRLFARTRSMWSFNPETNILLNVPLHGTILTRPLLESELVIGAVILRGHLRIAGHHLGRC-DIKDLPKEITVATSRTLSYYKL-GASQRVAGDSGFAAYSRYRIGNYKLNT
HKU1 VFTIISIVIWILYFVNSIRLFIRTGSWWSFNPETNNLMCIDMKGKMFVRPVIEDYHTLTATVIRGHLYIQGVKLGTGYTLSDLPVYVTVAKVQVLCTYKR-AFLDKLDVNSGFAVFVKSKVGNYRLPS
MERS-CoV IVAAVSAMMWISYFVQSIRLFMRTGSWWSFNPETNCLLNVPFGGTTVVRPLVEDSTSVTAVVTNGHLKMAGMHFGAC-DYDRLPNEVTVAKPNVLIALKM-VKRQSYGTNSGVAIYHRYKAGNYRSPP
OC43 VFTIVAIIMWIVYFVNSIRLFIRTGSFWSFNPETNNLMCIDMKGTMYVRPIIEDYHTLTVTIIRGHLYIQGIKLGTGYSLADLPAYMTVAKVTHLCTYKR-GFLDRISDTSGFAVYVKSKVGNYRLPS
TGE AGAIVTFVLWIMYFVRSIQLYRRTKSWWSFNPETKAILCVSALGRSYVLPLEGVPTGVTLTLLSGNLYAEGFKIAGGMNIDNLPKYVMVALPSRTIVYTLVGKKLKASSATGWAYYVKSKAGDYSTEA
FCoV AGAVVTFALWMMYFVRSVQLYRRTKSWWSFNPETNAILCVNALGRSYVLPLDGTPTGVTLTLLSGNLYAEGFKMAGGLTIEHLPKYVMIATPSRTIVYTLVGKQLKATTATGWAYYVKSKAGDYSTEA
NL63-related LMTIITLCLWIMYFVNSFKLYRRVKTFWAFNPETNAIISLQVYGHNYVQPVMTAPTGVTLTLLSGVLLIEGHKVAIRVQVGQLPKYLIVATPRTTIIYDRVGRSVNESSQTGWAFYVRSKHGDFSGIA
HKU2 LMLVITLVLWLMYIINSFKLYRRTRTFWAFNPETDAIAVISVFGRSYSIPMPVAPTGITLTILSGTLFFDGIRIATGVQPAHLPQFVTVAKPGTTIIYTRAGRSLNASTNTGWAFYVRSKHGDYSALS
BtCoV512 LMACVTAVLWIMYFVNSIRLWRRTHSWWSYNPETDSILSVSVLGRHVCLPILGAPTGVTLTLLNGTLLVEGYQVATGVQVNNLPGYVTVAKASTTIVYQRVGRSMNANSSTGWAFFVKSKHGDYYAAA
Porcine-EDV LMACITLMLWIMYFVNSIRLWRRTHSWWSFNPETDALLTTSVMGRQVCIPVLGAPTGVTLTLLSGTLLVEGYKVATGVQVSQLPNFVTVAKATTTIVYGRVGRSVNASSGTGWAFYVRSKHGDYSAVS
BtNv-AlphaCoV IMACVTFVLWIMYFVNSFKLYRRTQTFWAFNPETDAIITLNVFGRTVAIPVITAPTGITLTVLNGQLLVEGYKVANGVQVGQLPSYVTVAKPSTTIIYQRVSRSINVRSNTGWAFFVRSKNGDFSALA
BtMr-AlphaCoV FMLVVTLVLWIMYFVNSFRLYRRTRSFWAFNPETDSIIVFSVYGTTYSIPVIQAPQGITFTVLSGTLLVDGIKVATGVRIEALPQYVTVAKATTTIVFHRVGKAVNERTQTGWLFFVRSGYGDYAAHS
Bat-CoV-1A VMACTTLVLWLMYFINSFRLYRRTQSFWAFNPETDAIITISVFGKTVAIPVLVPPTGITLTVLSGKLLVEGIPVANGITVGQLPEFVTVAKATTTIIYRRAGRSLNAKTQTGWAFYVRAKNGDYSAIM
Mink-CoV AGSVLTFVLWVMYFVRSIQLWRRTRTWWSFNPETNAILCLNAVGNRFVLPLNGAPTGVTLTLLSGNLYAEGFKVGSGVNVENLPKYVMVATPGNTIVYHQVGKSLKAANSTGWSYYVRAKAGDYSTEA
BtRf-AlphaCoV LMSCITLVLWIMYFVNSFRLYRRTQTFWAFNPETDAIITLSVFGRQVSIPTIVVPTGITLTVLSGTLLVEGIKVATGVQVSQLPTYLTVAKPFTTIIYQRAGRSVNARSNTGWAFYVRSKNGDYSAVS
HKU10 LMACITLVLWIMYFVNSFRLYRRTNTFWAFNPETDAIITLSVFGRQVSIPALVAPTGITLTVLSGTLLVEGIKVATGVQVNQLPTYITVAKPSTTIVYQRAGRSLNTRSNTGWAFYVRSKNGDYSAVT
CDPHE15 LMACVTGVLWVMYFVNSIRLWRRTKSFWAWSPETDTILATTVLGRTVYLPLPAAPTGVTLTLLNGTLLVEGFKVAVGVTVELLPDYVVVAKPHTTIIYQRVGRSVNVRSNTGWSFYVKAKYGDYATTG
Ferret-CoV SGAMVTLVMWIMYFARSIQLWRRTRTWWSFNPETNAILCLSSVGKRFVLPLSGAPTGVTLTLLSGNLYAEGFKVGSGVNVDNLPKYIMVATPGNTIVYHQVGKSLKASSATGWSYYVRAKAGDYSTEA
LuchengRn-CoV ALGVVTLILWIMYFVNSFRLYRRTRTGSWWSFNPETNAIICVSVMGRNITTPTPVSPTGITLTLLSGTLYVEGQRAATGVNLDNLPNYVTVAKPGVTIVYHRVGKSIRVSTATGWMYYVKSKAGDYSAYS
HKU8 VMACTTLMLWIMYFVNSFRLYRRCQSFWAFNPETDAVITLSVFGKIVAIPVMTPPTGITLTILSGKLLVEGIPVANGVSVTQLPSYVTVAKATTTIIYQRVGKSLNAASQTGWAFYVRAKNGDYSAVS
NL63 LMSIITLCLWVMYFVNSFRLWRRVKTFWAFNPETNAIISLQVYGHNYYLPVMAAPTGVTLTLLSGVLLVDGHKIATRVQVGQLPKYVIVATPSTTIVCDRVGRSVNETSQTGWAFYVRAKHGDFSGVA
229E LMAVSTLVMWVMYFANSFRLFRRARTFWAWNPEVNAITVTTVLGQTYYQPIQQAPTGITVTLLSGVLYVDGHRLASGVQVHNLPEYMTVAVPSTTIIYSRVGRSVNSQNSTGWVFYVRVKHGDFSAVS
Beta-CoV cons
678776569*+5*996*8**83**4*3***7**884*989472*6768**964741695676 56*27384*448*49-44359*238*9*6542+267*5-523336322*+7769 2969 7**89376
Alpha-CoV cons
47585*679*8**877*77*9**7389*899**857966676 6*52646*6255*8*9*7*9*9*4*769*447+5485622**39879*5734**9725889486 3486***599*9653**95816
Combined cons
665776679*95*+74*87*73*8388*897**885+967372*53645*753322696679 75*3+464*437+44-44349*35879*7543725745-623342343 8+655949592*874336
Alignment qual

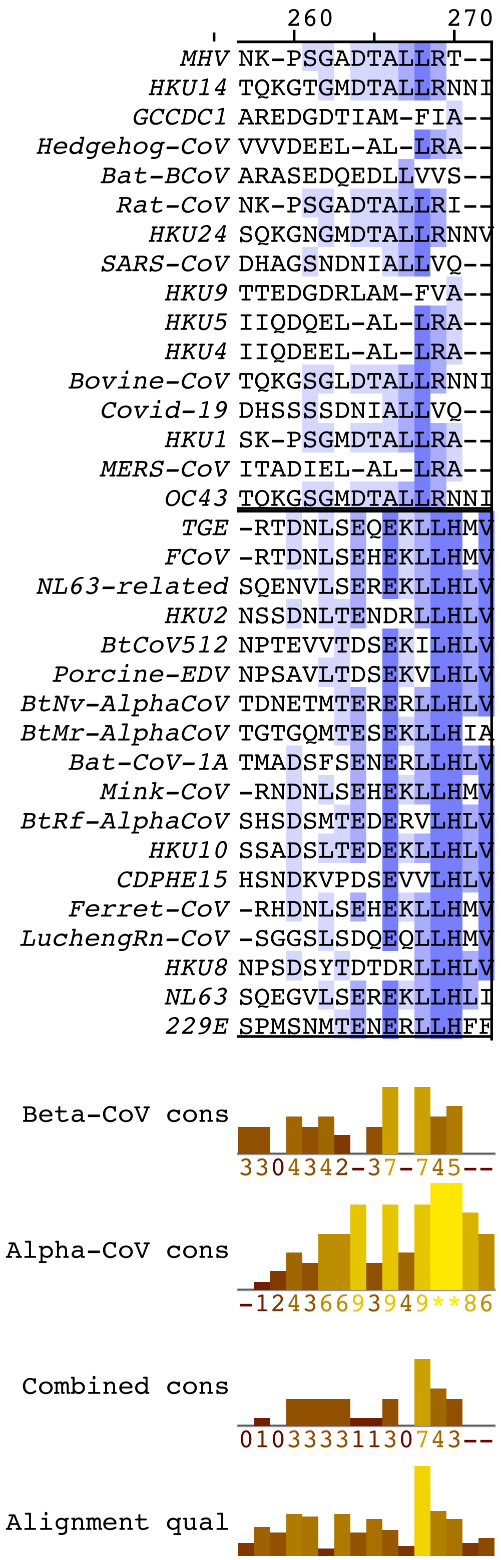
260
270
MHV NK-PSGADTALLRT--
HKU14 TQKGTGMDTALLRNNI
GCCDC1 AREDGDTIAM-FIA--
Hedgehog-CoV VVVDEEL-AL-LRA--
Bat-BCoV ARASEDQEDLLVVS--
Rat-CoV NK-PSGADTALLRI--
HKU24 SQKGNGMDTALLRNNV
SARS-CoV DHAGSNDNIALLVQ--
HKU9 TTEDGDRLAM-FVA--
HKU5 IIQDQEL-AL-LRA--
HKU4 IIQDEEL-AL-LRA--
Bovine-CoV TQKGSGLDTALLRNNI
Covid-19 DHSSSSDNIALLVQ--
HKU1 SK-PSGMDTALLRA--
MERS-CoV ITADIEL-AL-LRA--
OC43 TQKGSGMDTALLRNNI
TGE -RTDNLSEQEKLLHMV
FCoV -RTDNLSEHEKLLHMV
NL63-related SQENVLSEREKLLHLV
HKU2 NSSDNLTENDRLLHLV
BtCoV512 NPTEVVTDSEKILHLV
Porcine-EDV NPSAVLTDSEKVLHLV
BtNv-AlphaCoV TDNETMTERERLLHLV
BtMr-AlphaCoV TGTGQMTESEKLLHIA
Bat-CoV-1A TMADSFSENERLLHLV
Mink-CoV -RNDNLSEHEKLLHMV
BtRf-AlphaCoV SHSDSMTEDERVLHLV
HKU10 SSADSLTEDEKLLHLV
CDPHE15 HSNDKVPDSEVVLHLV
Ferret-CoV -RHDNLSEHEKLLHMV
LuchengRn-CoV -SGGSLSDQEQLLHMV
HKU8 NPSDSYTDTDRLLHLV
NL63 SQEGVLSEREKLLHLI
229E SPMSNMTENERLLHFF
Beta-CoV cons
3304342-37-745--
Alpha-CoV cons
-12436693949**86
Combined cons
010333311307 43--
Alignment qual

Nucleocapsid (N) protein

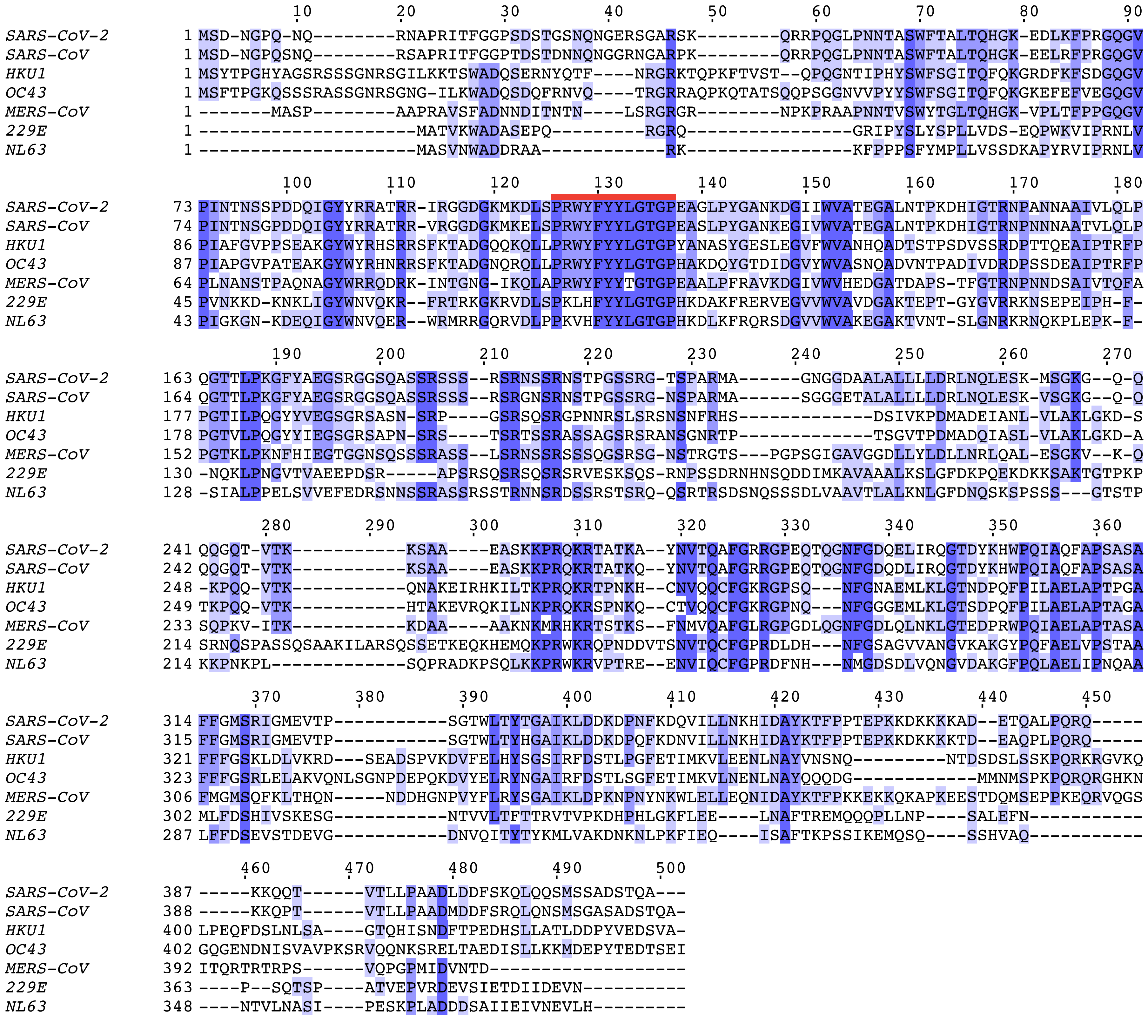

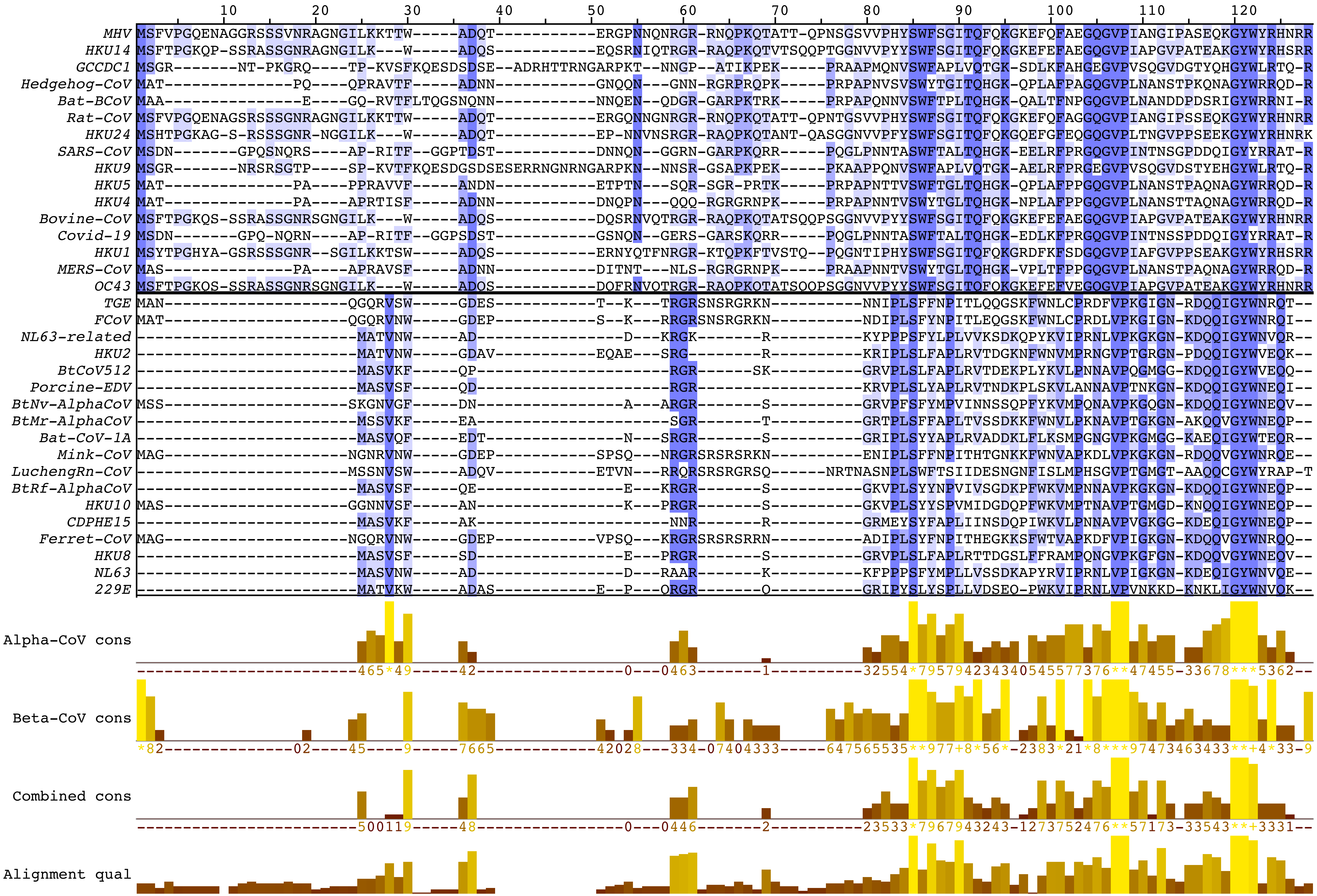
10
20
30
40
50
60
70
80
90
100
110
120
MHV
HKU14
GCCDC1
Hedgehog-CoV
Bat-BCoV
Rat-CoV
HKU24
SARS-CoV
HKU9
HKU5
HKU4
Bovine-CoV
Covid-19
HKU1
MERS-CoV
OC43
TGE
FCoV
NL63-related
HKU2
BtCoV512
Porcine-EDV
BtNv-AlphaCoV
BtMr-AlphaCoV
Bat-CoV-1A
Mink-CoV
LuchengRn-CoV
BtRf-AlphaCoV
HKU10
CDPHE15
Ferret-CoV
HKU8
NL63
229E
Alpha-CoV cons
Beta-CoV cons
Combined cons
Alignment qual

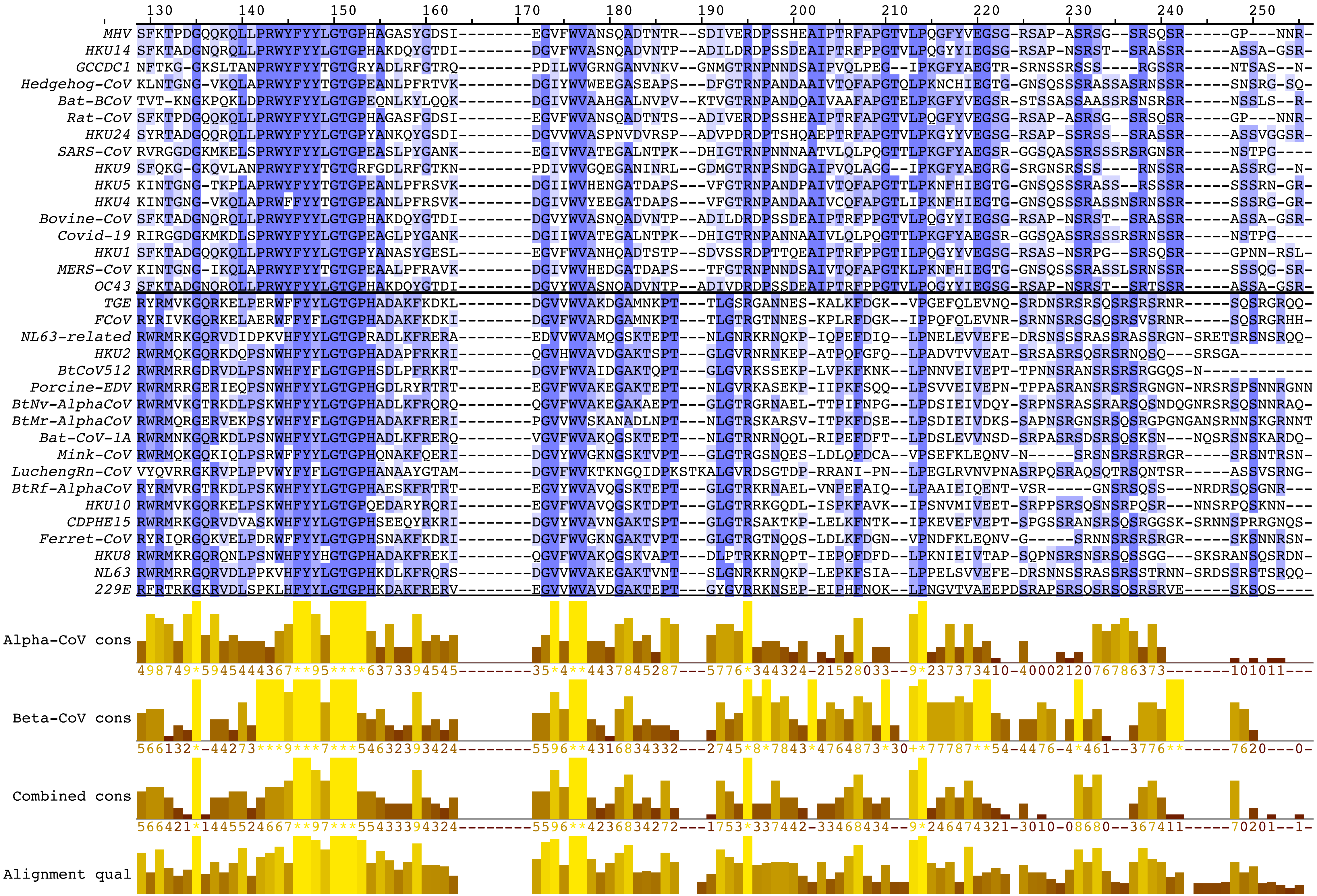
130 140 150 160 170 180 190 200 210 220 230 240 250
MHV SFKTPDGQQKQLLPRWYFYYLGTGPHAGASYGDSI--------EGVFWVANSQADTNTR--SDIVERDPSSHEAIPTRFAPGTVLPQGFYVEGSG-RSAP-ASRSG--SRSQSR-----GP---NNR-
HKU14 SFKTADGNQRQLLPRWYFYYLGTGPHAKDQYGTDI--------DGVFWVASNQADVNTP--ADILDRDPSSDEAIPTRFPPGTVLPQGYYIEGSG-RSAP-NSRST--SRASSR-----ASSA-GSR-
GCCDC1 NFTKG-GKSLTANPRWYFYYTGTGRYADLRFGTRQ--------PDILWVGRNGANVNKV--GNMGTRNPNNDSAIPVQLPEG--IPKGFYAEGTR-SRNSSRSSS----RGSSR-----NTSAS--N-
Hedgehog-CoV KLNTGNG-VKQLAPRWYFYYTGTGPEANLPFRTVK--------DGIYWVWEEGASEAPS---DFGTRNPANDAAIVTQFAPGTQLPKNCHIEGTG-GNSQSSSRASSASRNSSR-----SNSRG-SQ-
Bat-BCoV TVT-KNGKPQKLDPRWYFYYLGTGPEQNLKYLQQK--------DGIVWVAAHGALNVPV--KTVGTRNPANDQAIVAAFAPGTELPKGFYVEGSR-STSSASSAASSRSNSRSR-----NSSLS--R-
Rat-CoV SFKTPDGQQKQLLPRWYFYYLGTGPHAGASFGDSI--------EGVFWVANSQADTNTS--ADIVERDPSSHEAIPTRFAPGTVLPQGFYVEGSG-RSAP-ASRSG--SRSQSR-----GP---NNR-
HKU24 SYRTADGQQRQLLPRWYFYYLGTGPYANKQYGSDI--------DGVVWVASPNVDVRSP--ADVPDRDPTSHQAEPTRFAPGTVLPKGYYVEGSG-RSAP-SSRSS--SRASSR-----ASSVGGSR-
SARS-CoV RVRGGDGKMKELSPRWYFYYLGTGPEASLPYGANK--------EGIVWVATEGALNTPK--DHIGTRNPNNNAATVLQLPQGTTLPKGFYAEGSR-GGSQASSRSSSRSRGNSR-----NSTPG----
HKU9 SFQKG-GKQVLANPRWYFYYTGTGRFGDLRFGTKN--------PDIVWVGQEGANINRL--GDMGTRNPSNDGAIPVQLAGG--IPKGFYAEGRG-SRGNSRSSS----RNSSR-----ASSRG--N-
HKU5 KINTGNG-TKPLAPRWYFYYTGTGPEANLPFRSVK--------DGIIWVHENGATDAPS---VFGTRNPANDPAIVTQFAPGTTLPKNFHIEGTG-GNSQSSSRASS--RSSSR-----SSSRN-GR-
HKU4 KINTGNG-VKQLAPRWFFYYTGTGPEANLPFRSVK--------DGIVWVYEEGATDAPS---VFGTRNPANDAAIVCQFAPGTLIPKNFHIEGTG-GNSQSSSRASSNSRNSSR-----SSSRG-GR-
Bovine-CoV SFKTADGNQRQLLPRWYFYYLGTGPHAKDQYGTDI--------DGVYWVASNQADVNTP--ADILDRDPSSDEAIPTRFPPGTVLPQGYYIEGSG-RSAP-NSRST--SRASSR-----ASSA-GSR-
Covid-19 RIRGGDGKMKDLSPRWYFYYLGTGPEAGLPYGANK--------DGIIWVATEGALNTPK--DHIGTRNPANNAAIVLQLPQGTTLPKGFYAEGSR-GGSQASSRSSSRSRNSSR-----NSTPG----
HKU1 SFKTADGQQKQLLPRWYFYYLGTGPYANASYGESL--------EGVFWVANHQADTSTP--SDVSSRDPTTQEAIPTRFPPGTILPQGYYVEGSG-RSAS-NSRPG--SRSQSR-----GPNN-RSL-
MERS-CoV KINTGNG-IKQLAPRWYFYYTGTGPEAALPFRAVK--------DGIVWVHEDGATDAPS---TFGTRNPNNDSAIVTQFAPGTKLPKNFHIEGTG-GNSQSSSRASSLSRNSSR-----SSSQG-SR-
OC43 SFKTADGNQRQLLPRWYFYYLGTGPHAKDQYGTDI--------DGVYWVASNQADVNTP--ADIVDRDPSSDEAIPTRFPPGTVLPQGYYIEGSG-RSAP-NSRST--SRTSSR-----ASSA-GSR-
TGE RYRMVKGQRKELPERWFFYYLGTGPHADAKFKDKL--------DGVVWVAKDGAMNKPT---TLGSRGANNES-KALKFDGK--VPGEFQLEVNQ-SRDNSRSRSQSRSRSRNR-----SQSRGRQQ-
FCoV RYRIVKGQRKELAERWFFYFLGTGPHADAKFKDKI--------DGVFWVARDGAMNKPT---TLGTRGTNNES-KPLRFDGK--IPPQFQLEVNR-SRNNSRSGSQSRSVSRNR-----SQSRGRHH-
NL63-related RWRMRKGQRVDIDPKVHFYYLGTGPRADLKFRERA--------EDVVWVAMQGSKTEPT---NLGNRKRNQKP-IQPEFDIQ--LPNELEVVEFE-DRSNSSSRASSRASSRGN-SRETSRSNSRQQ-
HKU2 RWRMQKGQRKDQPSNWHFYYLGTGPHADAPFRKRI--------QGVHWVAVDGAKTSPT---GLGVRNRNKEP-ATPQFGFQ--LPADVTVVEAT-SRSASRSQSRSRNQSQ---SRSGA--------
BtCoV512 RWRMRRGDRVDLPSNWHFYFLGTGPHSDLPFRKRT--------DGVFWVAIDGAKTQPT---GLGVRKSSEKP-LVPKFKNK--LPNNVEIVEPT-TPNNSRANSRSRSRGGQS-N------------
Porcine-EDV RWRMRRGERIEQPSNWHFYYLGTGPHGDLRYRTRT--------EGVFWVAKEGAKTEPT---NLGVRKASEKP-IIPKFSQQ--LPSVVEIVEPN-TPPASRANSRSRSRGNGN-NRSRSPSNNRGNN
BtNv-AlphaCoV RWRMVKGTRKDLPSKWHFYYLGTGPHADLKFRQRQ--------QGVFWVAKEGAKAEPT---GLGTRGRNAEL-TTPIFNPG--LPDSIEIVDQY-SRPNSRASSRARSQSNDQGNRSRSQSNNRAQ-
BtMr-AlphaCoV RWRMQRGERVEKPSYWHFYFLGTGPHADAKFRERI--------PGVVWVSKANADLNPT---NLGTRSKARSV-ITPKFDSE--LPSDIEIVDKS-SAPNSRGNSRSQSRGPGNGANSRNNSKGRNNT
Bat-CoV-1A RWRMNKGQRKDLPSNWHFYYLGTGPHADLKFRERQ--------VGVFWVAKQGSKTEPT---NLGTRNRNQQL-RIPEFDFT--LPDSLEVVNSD-SRPASRSDSRSQSKSN---NQSRSNSKARDQ-
Mink-CoV RWRMQKGQKIQLPSRWFFYYLGTGPHQNAKFQERI--------DGVYWVGKNGSKTVPT---GLGTRGSNQES-LDLQFDCA--VPSEFKLEQNV-N-----SRSNSRSRSRGR-----SRSNTRSN-
LuchengRn-CoV VYQVRRGKRVPLPPVWYFYFLGTGPHANAAYGTAM--------DGVFWVKTKNGQIDPKSTKALGVRDSGTDP-RRANI-PN--LPEGLRVNVPNASRPQSRAQSQTRSQNTSR-----ASSVSRNG-
BtRf-AlphaCoV RYRMVRGTRKDLPSKWHFYYLGTGPHAESKFRTRT--------EGVYWVAVQGSKTEPT---GLGTRKRNAEL-VNPEFAIQ--LPAAIEIQENT-VSR-----GNSRSQSS---NRDRSQSGNR---
HKU10 RWRMVKGQRKELPSKWHFYYLGTGPQEDARYRQRI--------EGVFWVAVQGSKTDPT---GLGTRRKGQDL-ISPKFAVK--IPSNVMIVEET-SRPPSRSQSNSRPQSR---NNSRPQSKNN---
CDPHE15 RWRMRKGQRVDVASKWHFYYLGTGPHSEEQYRKRI--------DGVYWVAVNGAKTSPT---GLGTRSAKTKP-LELKFNTK--IPKEVEFVEPT-SPGSSRANSRSQSRGGSK-SRNNSPRRGNQS-
Ferret-CoV RYRIQRGQKVELPDRWFFYYLGTGPHSNAKFKDRI--------DGVFWVGKNGAKTVPT---GLGTRGTNQQS-LDLKFDGN--VPNDFKLEQNV-G-----SRNNSRSRSRGR-----SKSNNRSN-
HKU8 RWRMKRGQRQNLPSNWHFYYHGTGPHADAKFREKI--------QGVFWVAKQGSKVAPT---DLPTRKRNQPT-IEPQFDFD--LPKNIEIVTAP-SQPNSRSNSRSQSSGG---SKSRANSQSRDN-
NL63 RWRMRRGQRVDLPPKVHFYYLGTGPHKDLKFRQRS--------DGVVWVAKEGAKTVNT---SLGNRKRNQKP-LEPKFSIA--LPPELSVVEFE-DRSNNSSRASSRSSTRNN-SRDSSRSTSRQQ-
229E RFRTRKGKRVDLSPKLHFYYLGTGPHKDAKFRERV--------EGVVWVAVDGAKTEPT---GYGVRRKNSEP-EIPHFNQK--LPNGVTVAEEPDSRAPSRSQSRSQSRSRVE-----SKSQS----
Alpha-CoV cons
498749*5945444367**95****6373394545--------35*4**4437845287---5776*344324-21528033--9*237373410-4000212076786373--------101011---
Beta-CoV cons
566132*-44273***9***7***54632393424--------5596**4316834332---2745*8*7843*4764873*30+*77787**54-4476-4*461--3776**------7620---0-
Combined cons
566421*1445524667**97***55433394324--------5596**4236834272---1753*337442-33468434--9*246474321-3010-08680--367411------70201--1-
Alignment qual

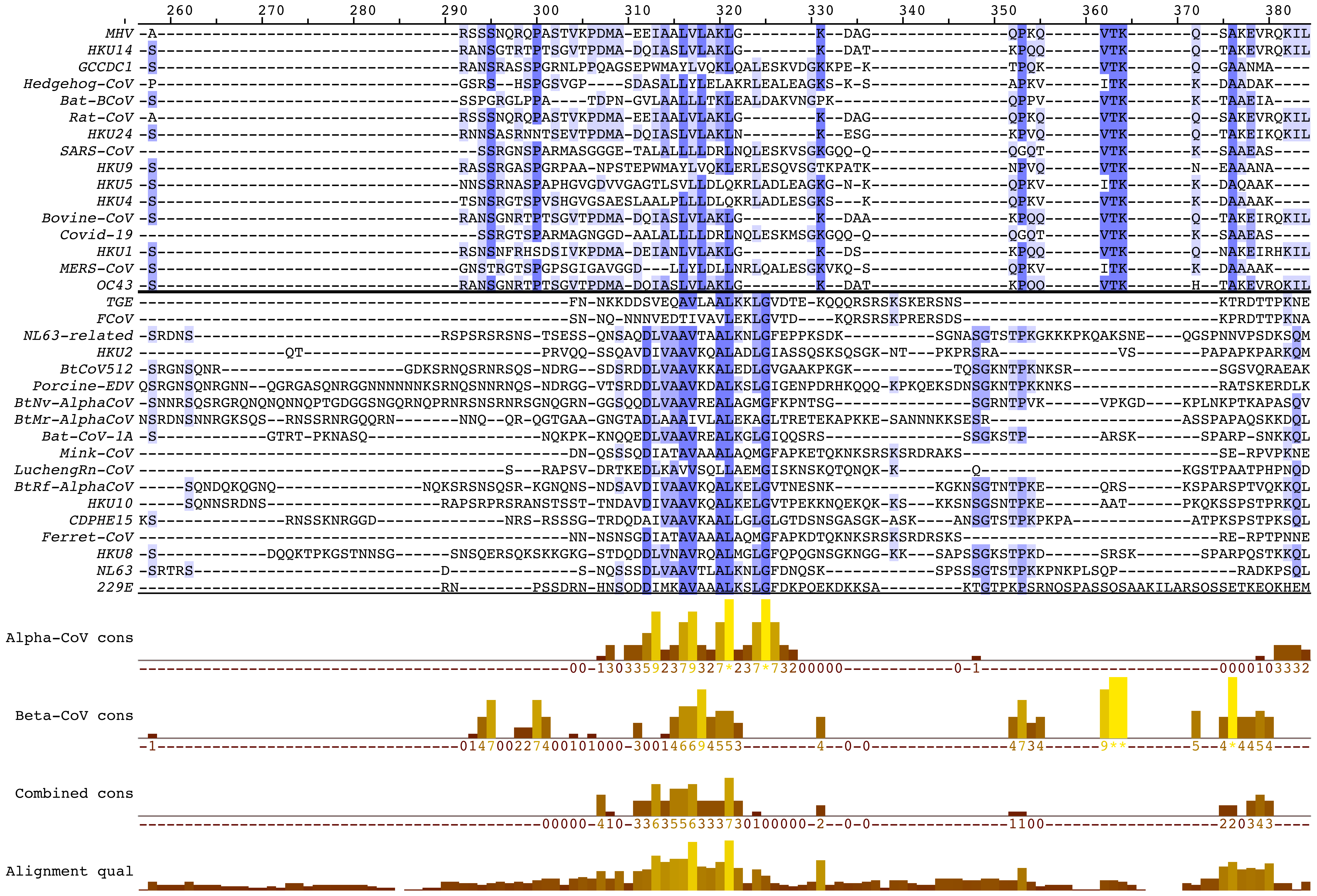
260 270 280 290 300 310 320 330 340 350 360 370 380
MHV
HKU14
GCCDC1
Hedgehog-CoV
Bat-BCoV
Rat-CoV
HKU24
SARS-CoV
HKU9
HKU5
HKU4
Bovine-CoV
Covid-19
HKU1
MERS-CoV
OC43
TGE
FCoV
NL63-related
HKU2
BtCoV512
Porcine-EDV
BtNv-AlphaCoV
BtMr-AlphaCoV
Bat-CoV-1A
Mink-CoV
LuchengRn-CoV
BtRf-AlphaCoV
HKU10
CDPHE15
Ferret-CoV
HKU8
NL63
229E
Alpha-CoV cons
Beta-CoV cons
Combined cons
Alignment qual

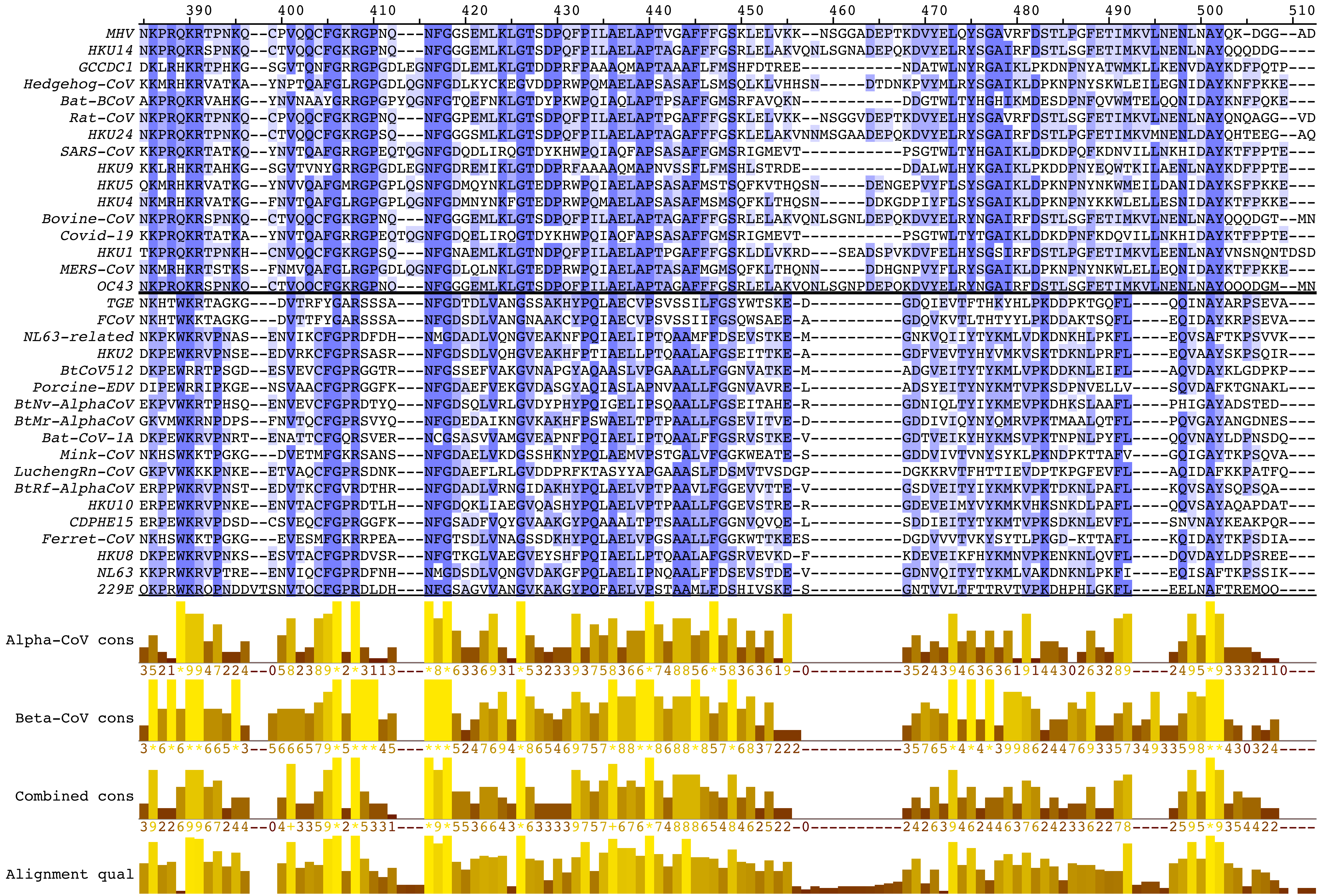

390 400 410 420 430 440 450 460 470 480 490 500 510
MHV
HKU14
GCCDC1
Hedgehog-CoV
Bat-BCoV
Rat-CoV
HKU24
SARS-CoV
HKU9
HKU5
HKU4
Bovine-CoV
Covid-19
HKU1
MERS-CoV
OC43
TGE
FCoV
NL63-related
HKU2
BtCoV512
Porcine-EDV
BtNv-AlphaCoV
BtMr-AlphaCoV
Bat-CoV-1A
Mink-CoV
LuchengRn-CoV
BtRf-AlphaCoV
HKU10
CDPHE15
Ferret-CoV
HKU8
NL63
229E
Alpha-CoV cons
Beta-CoV cons
Combined cons
Alignment qual

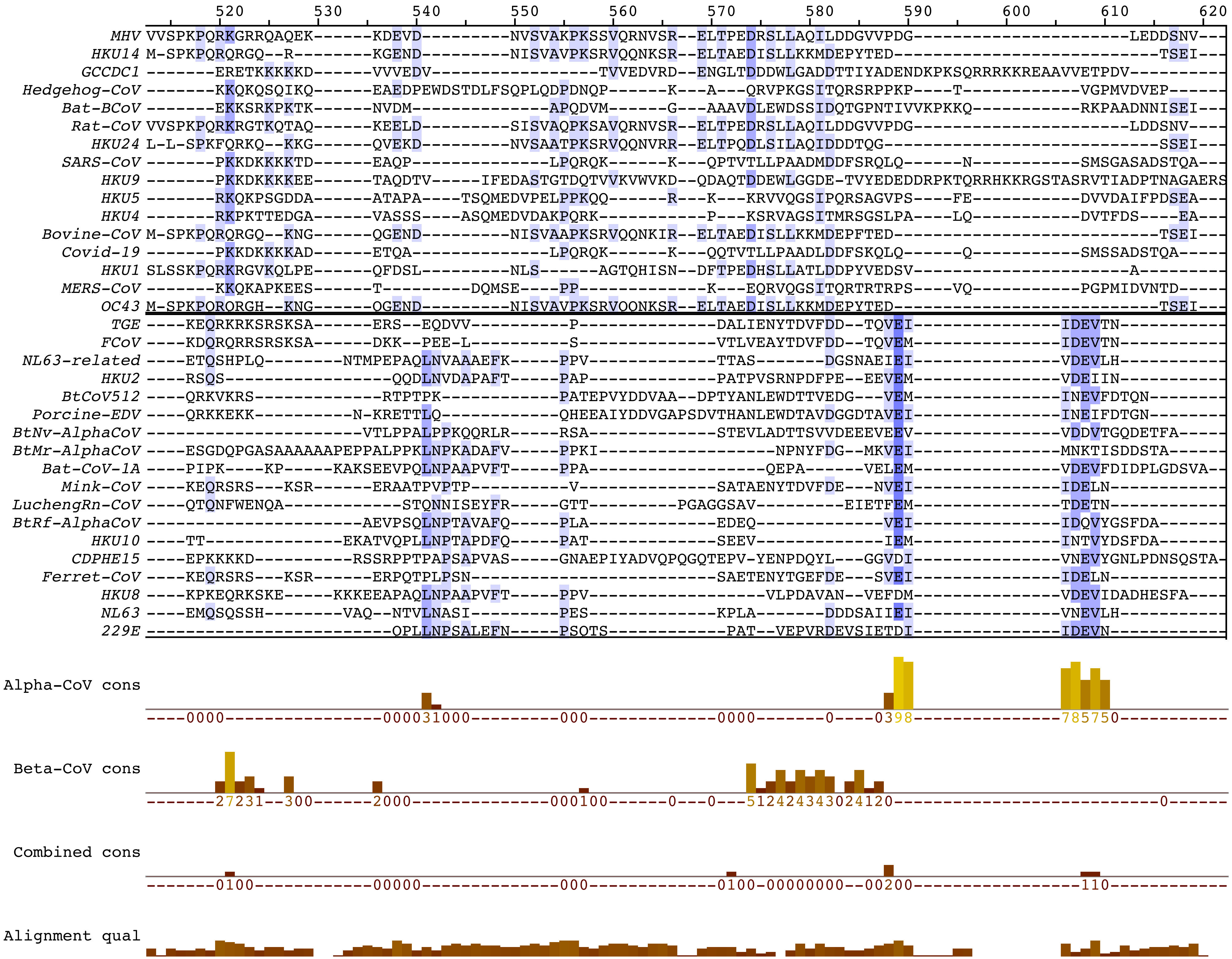

520
530
540
550
560
570
580
590
600
610
620
MHV
HKU14
GCCDC1
Hedgehog-CoV
Bat-BCoV
Rat-CoV
HKU24
SARS-CoV
HKU9
HKU5
HKU4
Bovine-CoV
Covid-19
HKU1
MERS-CoV
OC43
TGE
FCoV
NL63-related
HKU2
BtCoV512
Porcine-EDV
BtNv-AlphaCoV
BtMr-AlphaCoV
Bat-CoV-1A
Mink-CoV
LuchengRn-CoV
BtRf-AlphaCoV
HKU10
CDPHE15
Ferret-CoV
HKU8
NL63
229E
Alpha-CoV cons
Beta-CoV cons
Combined cons
Alignment qual
