# Supplementary material for: Human Leukocyte Antigen Susceptibility Map for Severe Acute Respiratory Syndrome Coronavirus 2
Source: J Virol. 2020 Jun 16;94(13):e00510-20. doi: 10.1128/JVI.00510-20 (PMC7307149; doi:10.1128/JVI.00510-20)
Supplement: Supplemental file 2 [file JVI.00510-20-s0002.pdf]

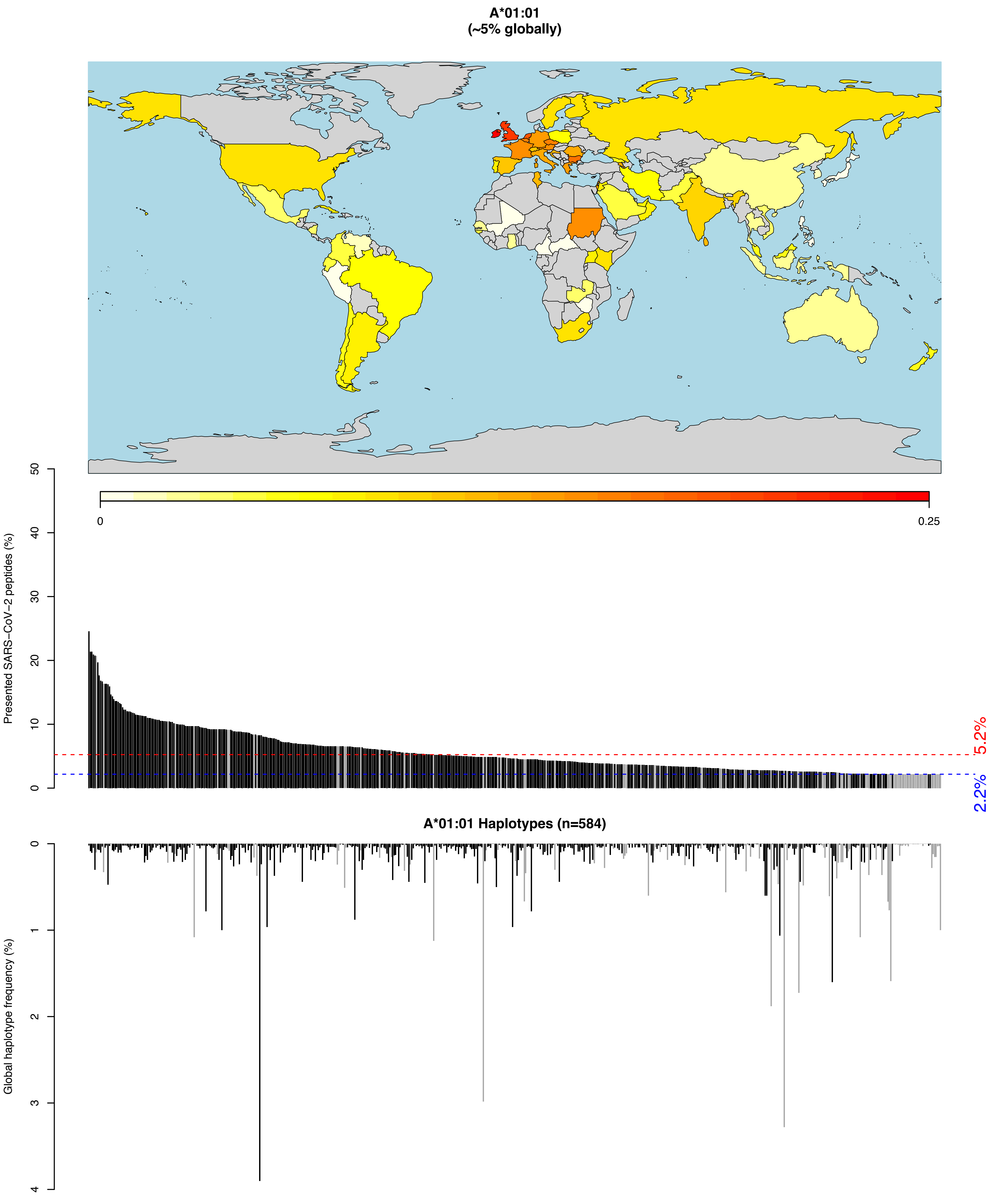

A*01:01
(~5% globally)
0
0.25
Presented SARS−CoV−2 peptides (%)
50
40
30
20
10
0
5.2%
2.2%
A*01:01 Haplotypes (n=584)
Global haplotype frequency (%)
0
1
2
3
4

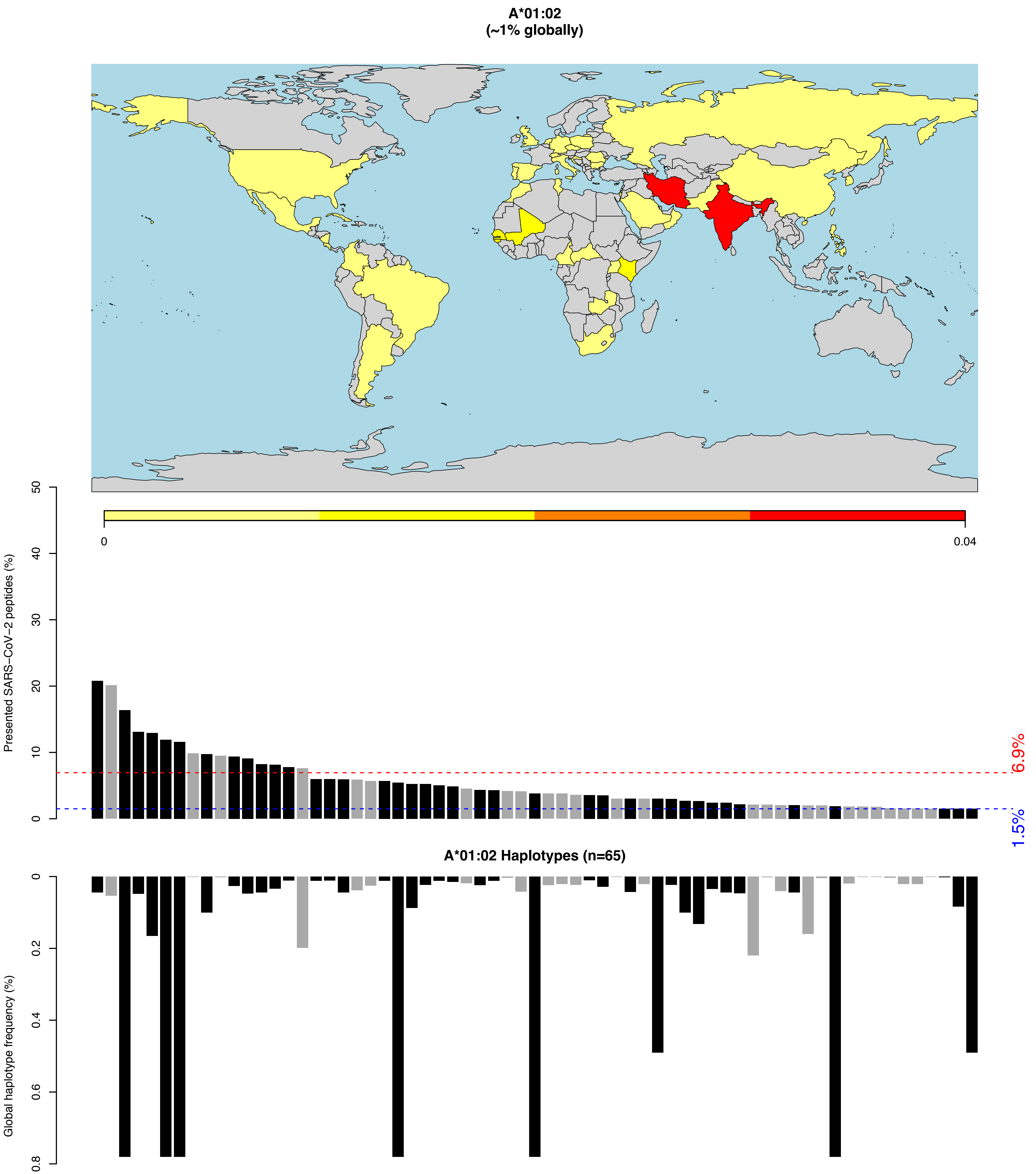

A*01:02
(~1% globally)
0
0.04
Presented SARS−CoV−2 peptides (%)
50
40
30
20
10
0
6.9%
1.5%
A*01:02 Haplotypes (n=65)
Global haplotype frequency (%)
0
0.2
0.4
0.6
0.8

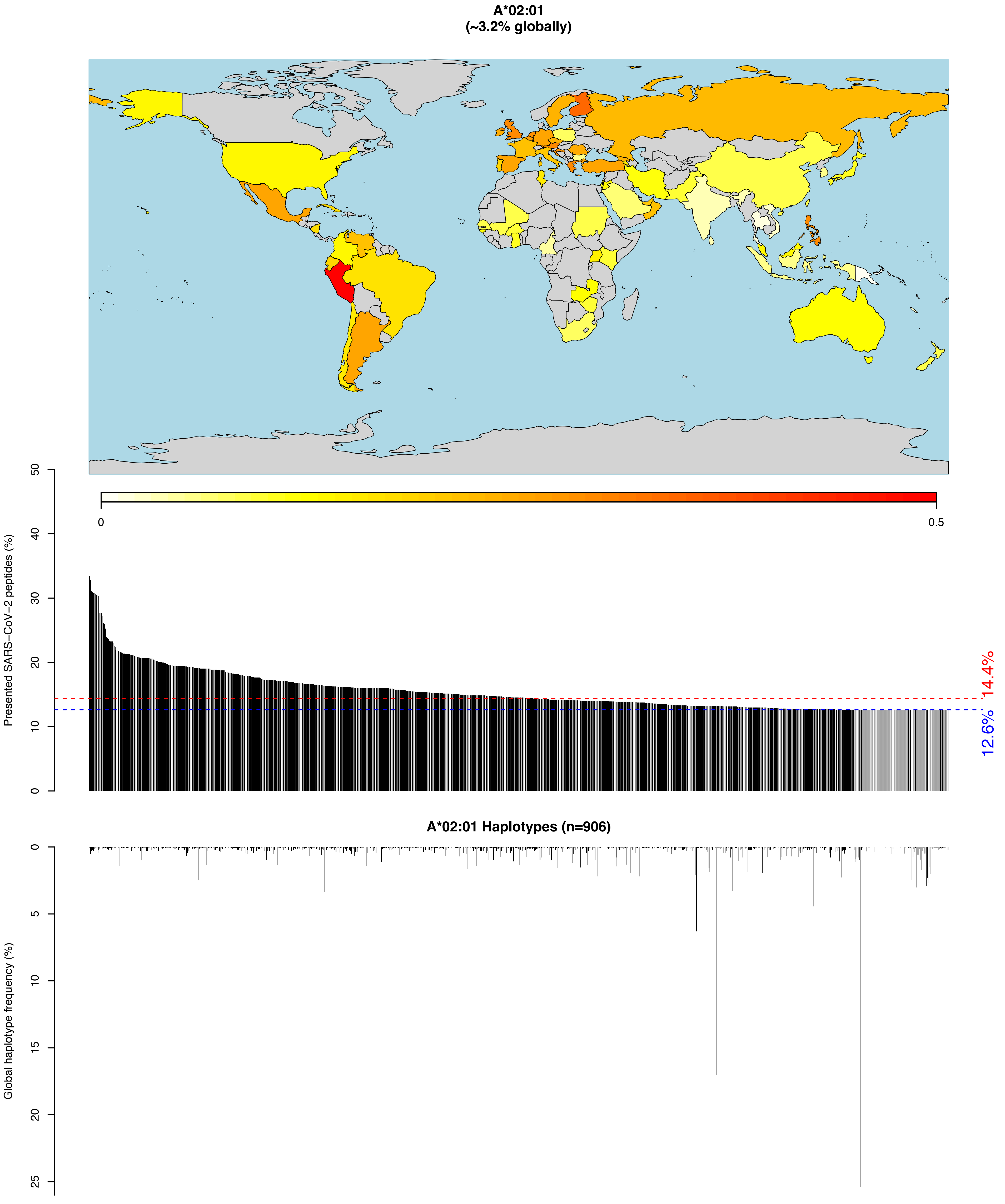
A*02:01
(~3.2% globally)
0
0.5
Presented SARS−CoV−2 peptides (%)
0
10
20
30
40
50
14.4%
12.6%
A*02:01 Haplotypes (n=906)
Global haplotype frequency (%)
0
5
10
15
20
25

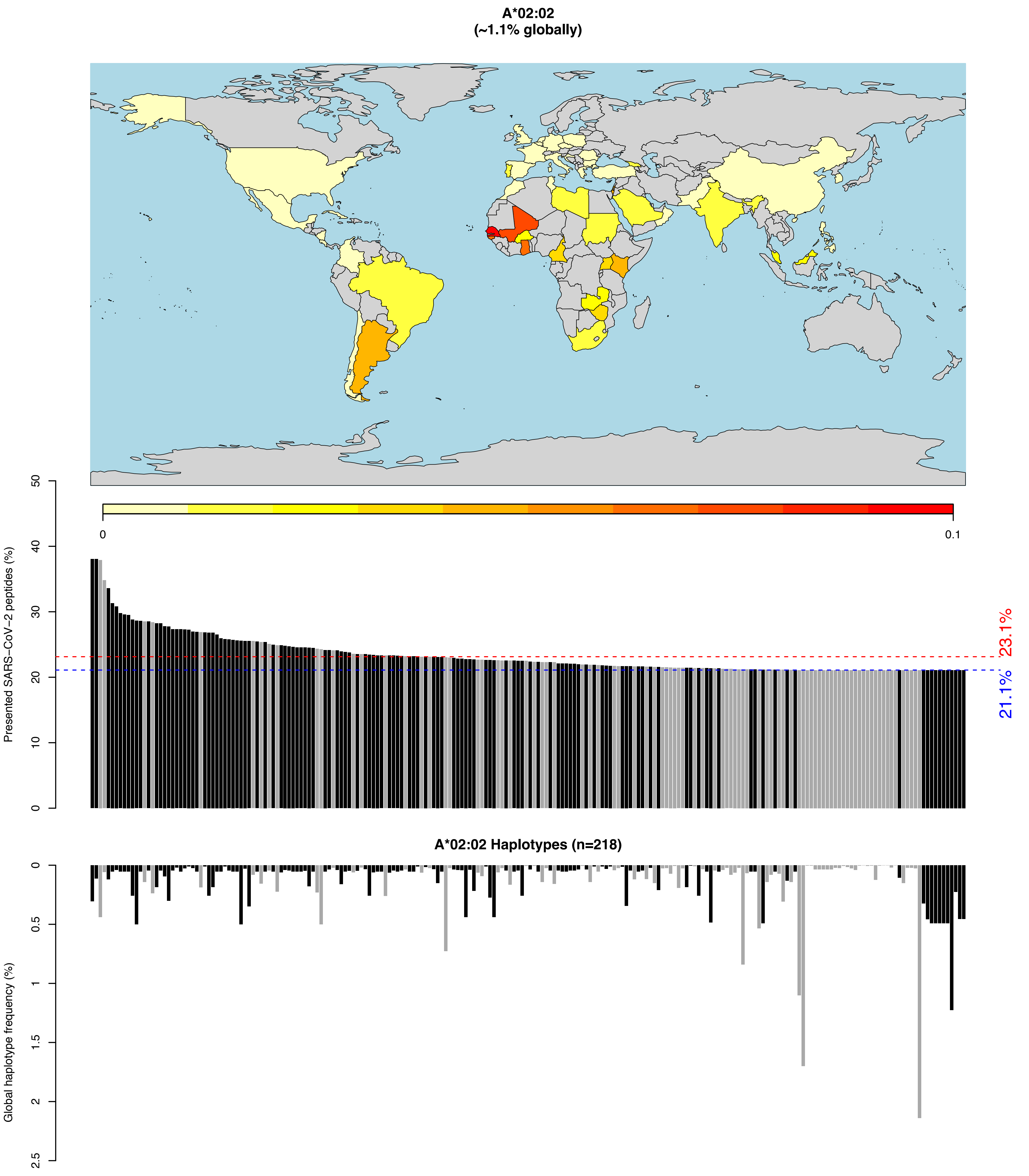
A*02:02
(~1.1% globally)
0
0.1
Presented SARS−CoV−2 peptides (%)
0
10
20
30
40
50
23.1%
21.1%
A*02:02 Haplotypes (n=218)
Global haplotype frequency (%)
0
0.5
1
1.5
2
2.5

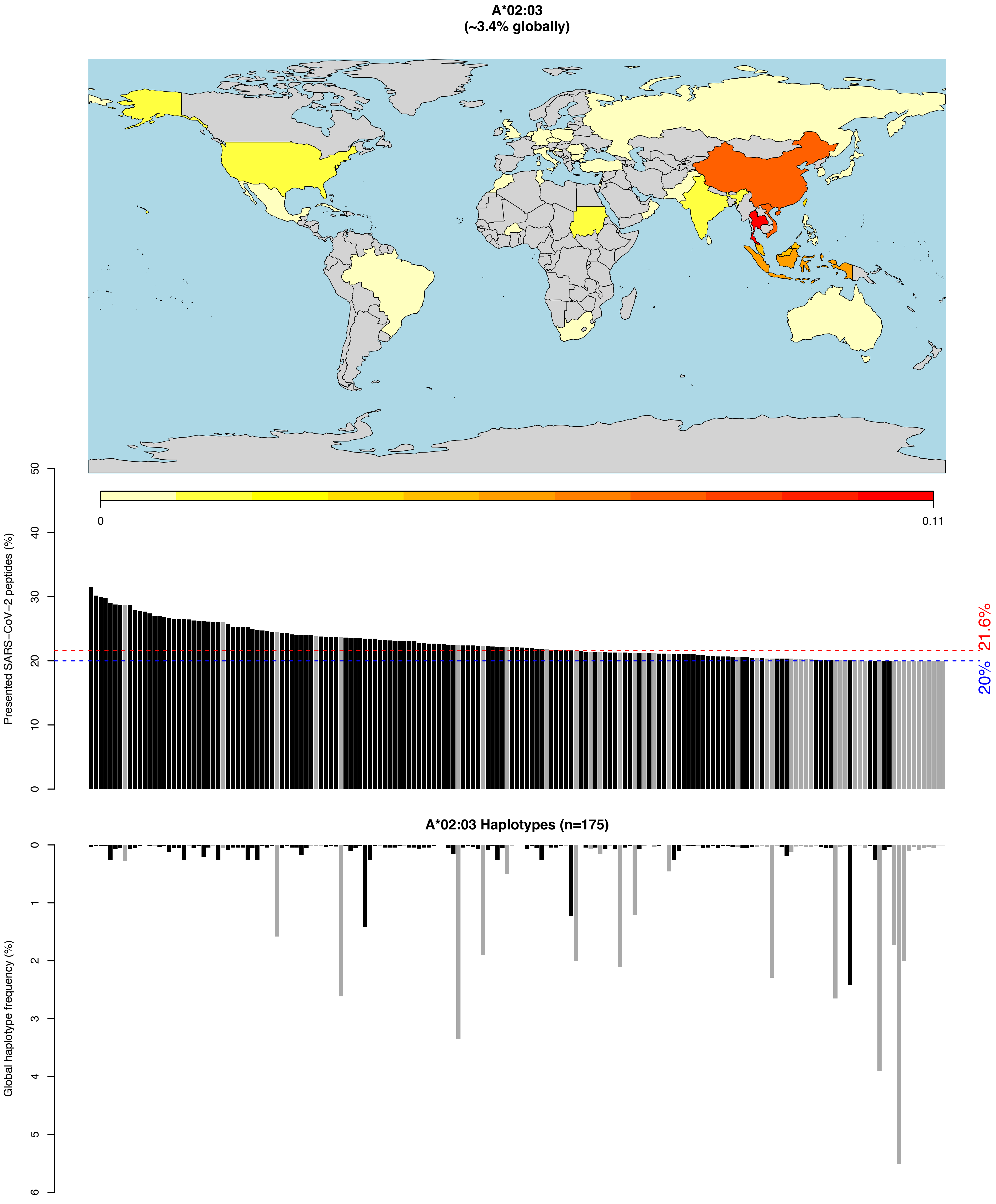

A*02:03
(~3.4% globally)
0
0.11
Presented SARS−CoV−2 peptides (%)
50
40
30
20
10
0
21.6%
20%
A*02:03 Haplotypes (n=175)
Global haplotype frequency (%)
0
1
2
3
4
5
6

A*02:05
(~0.91% globally)

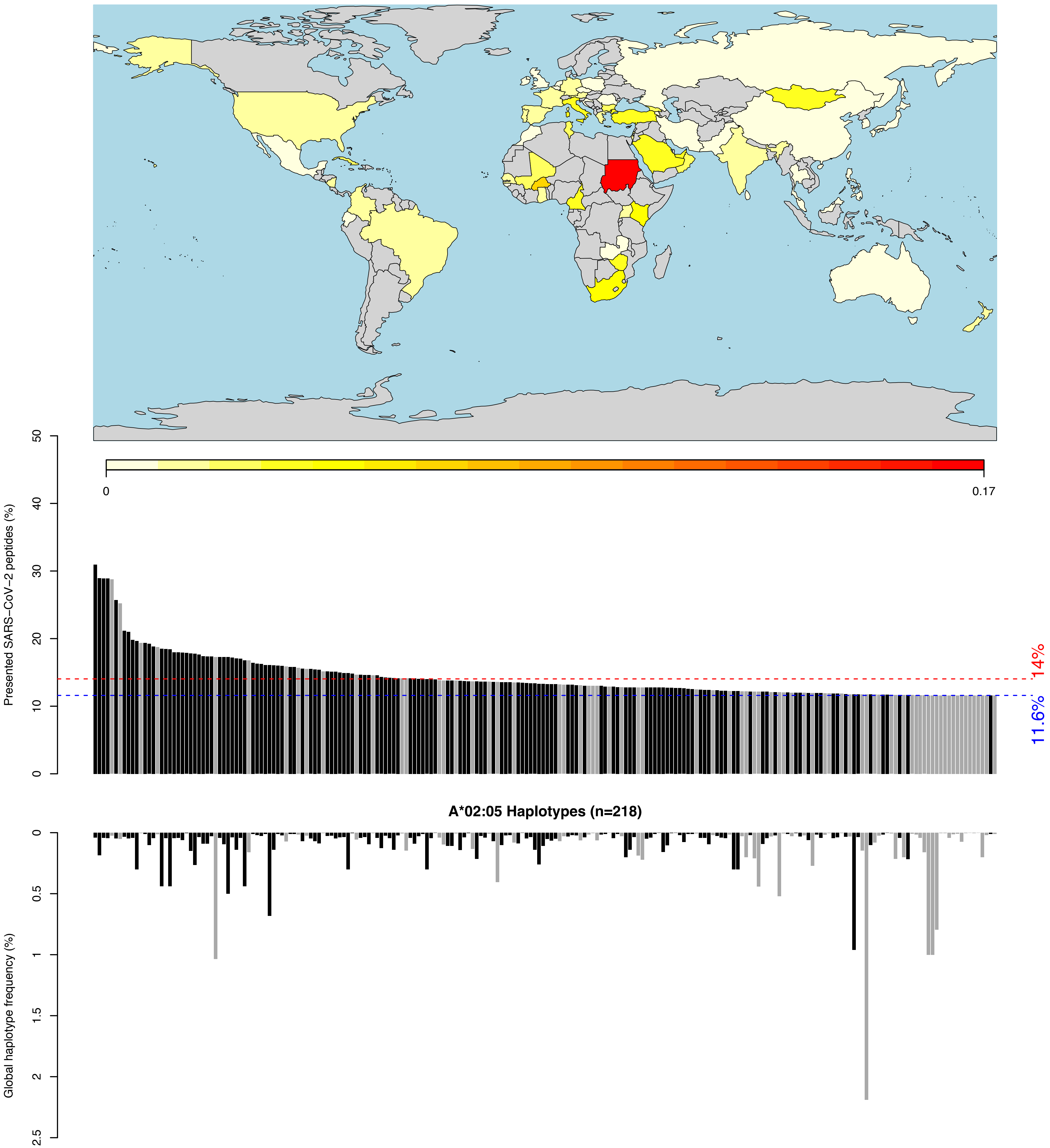

A*02:06
(~2.6% globally)

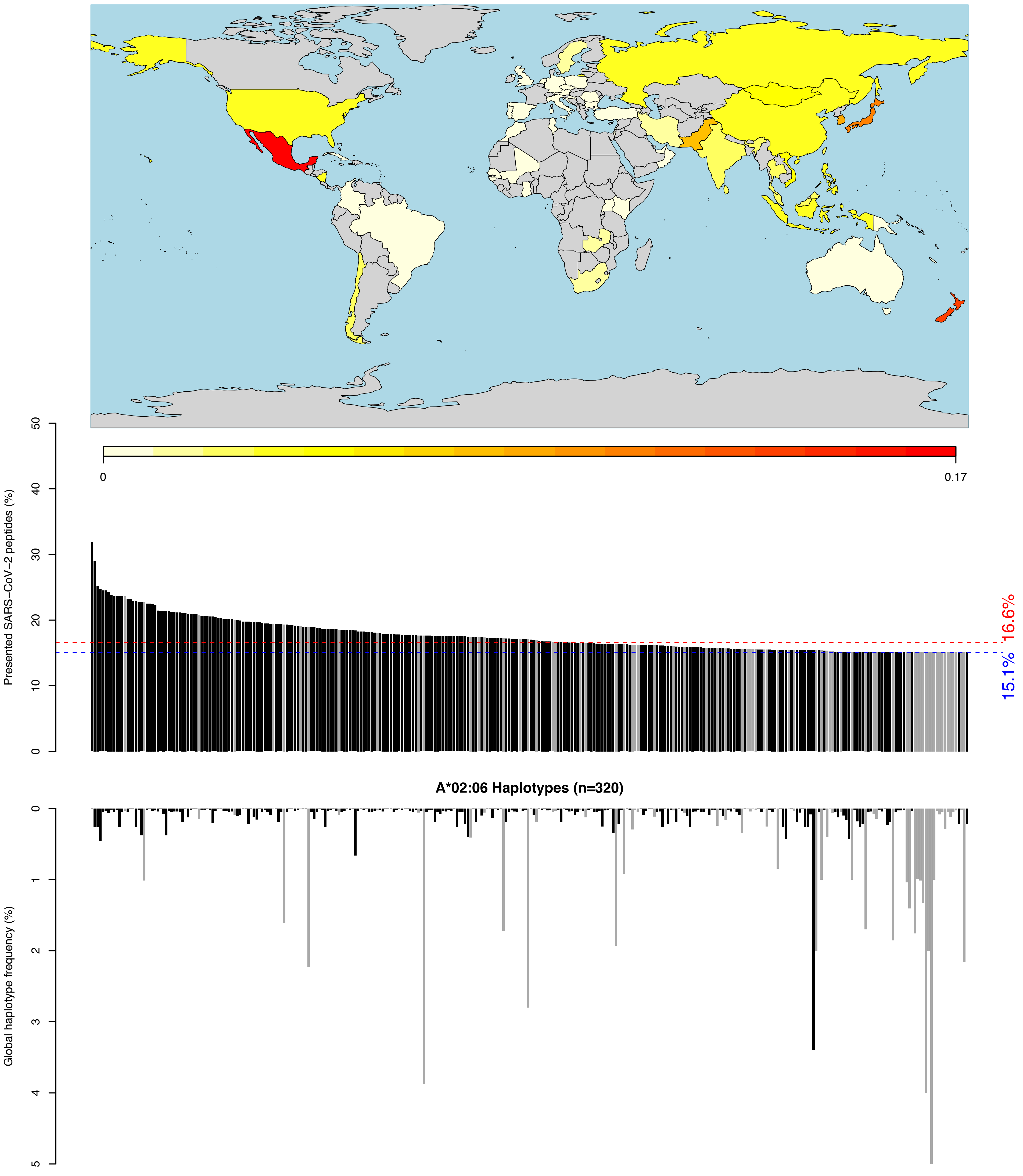

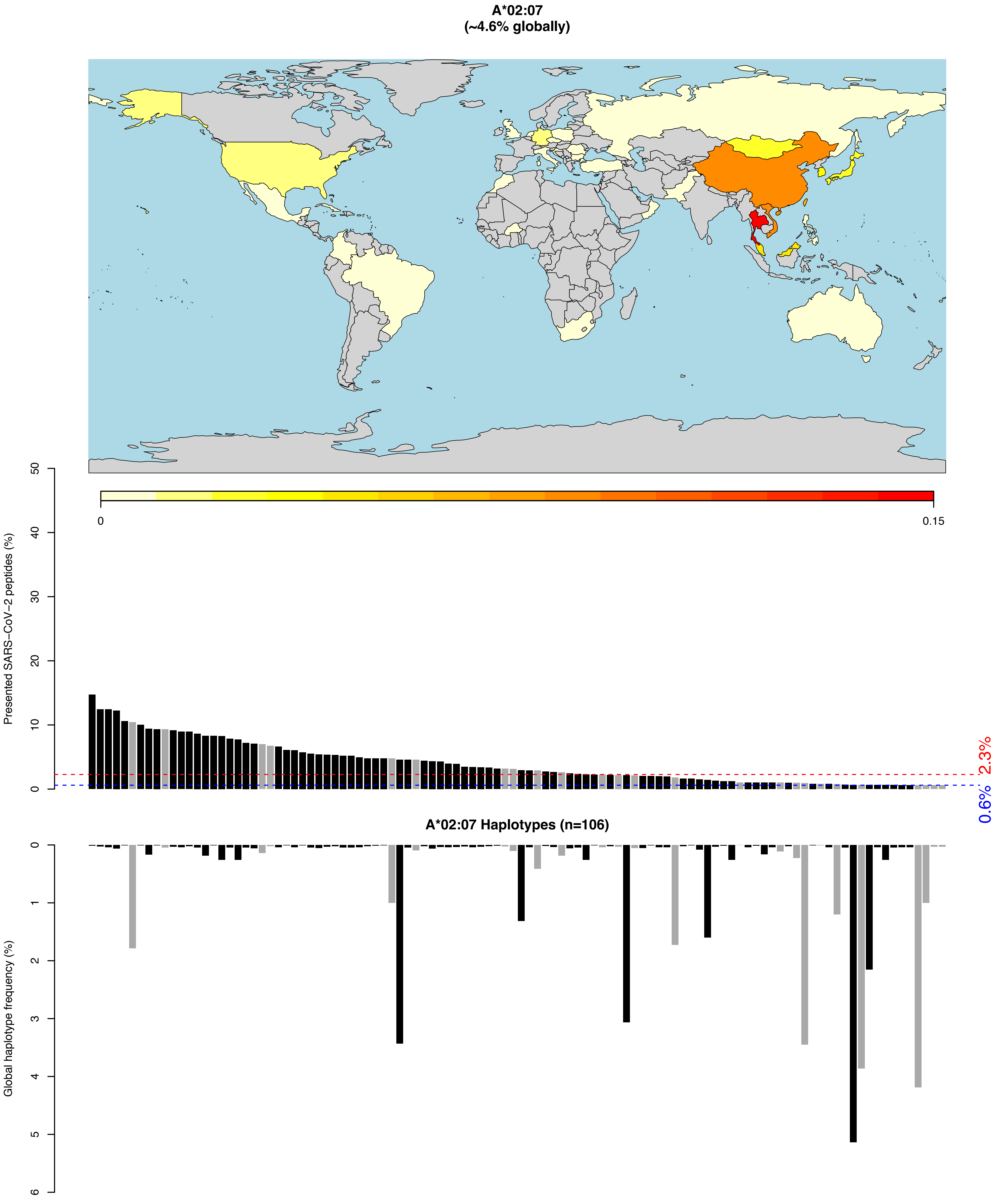

A*02:07
(~4.6% globally)
0
0.15
Presented SARS−CoV−2 peptides (%)
0
10
20
30
40
50
2.3%
0.6%
A*02:07 Haplotypes (n=106)
Global haplotype frequency (%)
0
1
2
3
4
5
6

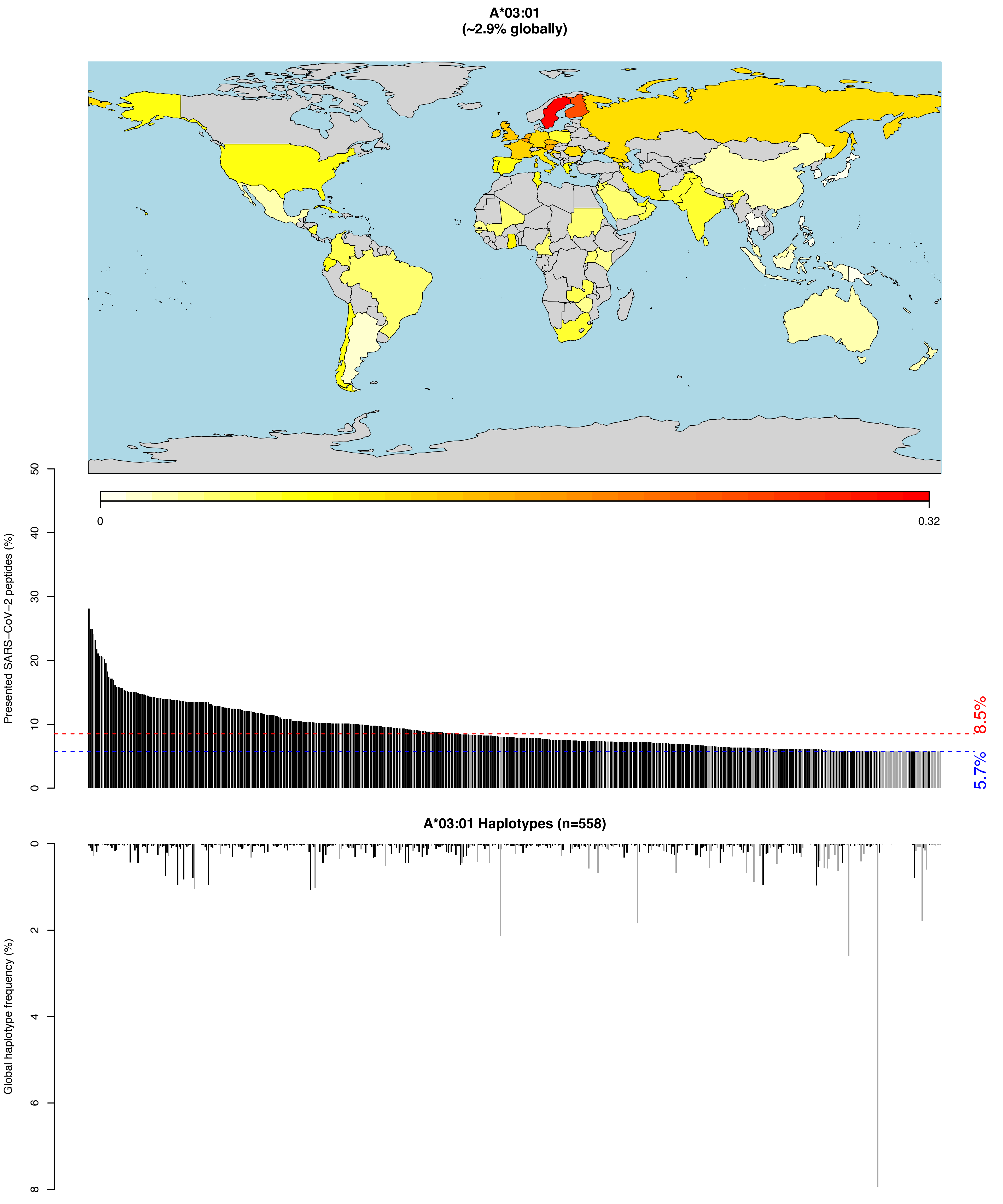
A*03:01
(~2.9% globally)
0
0.32
Presented SARS−CoV−2 peptides (%)
50
40
30
20
10
0
8.5%
5.7%
A*03:01 Haplotypes (n=558)
Global haplotype frequency (%)
0
2
4
6
8

A*03:02
(~0.47% globally)

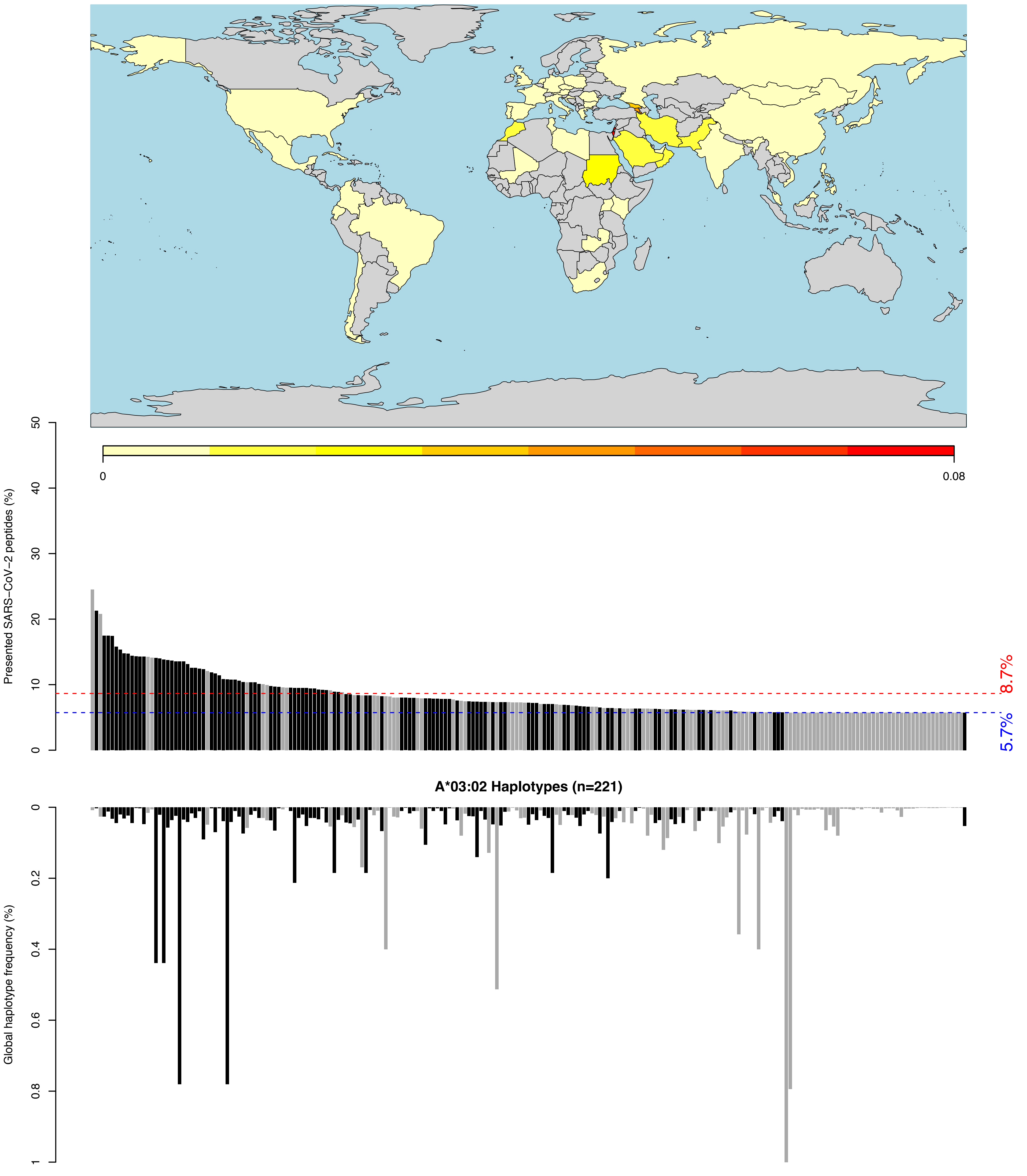

A*11:01
(~14% globally)

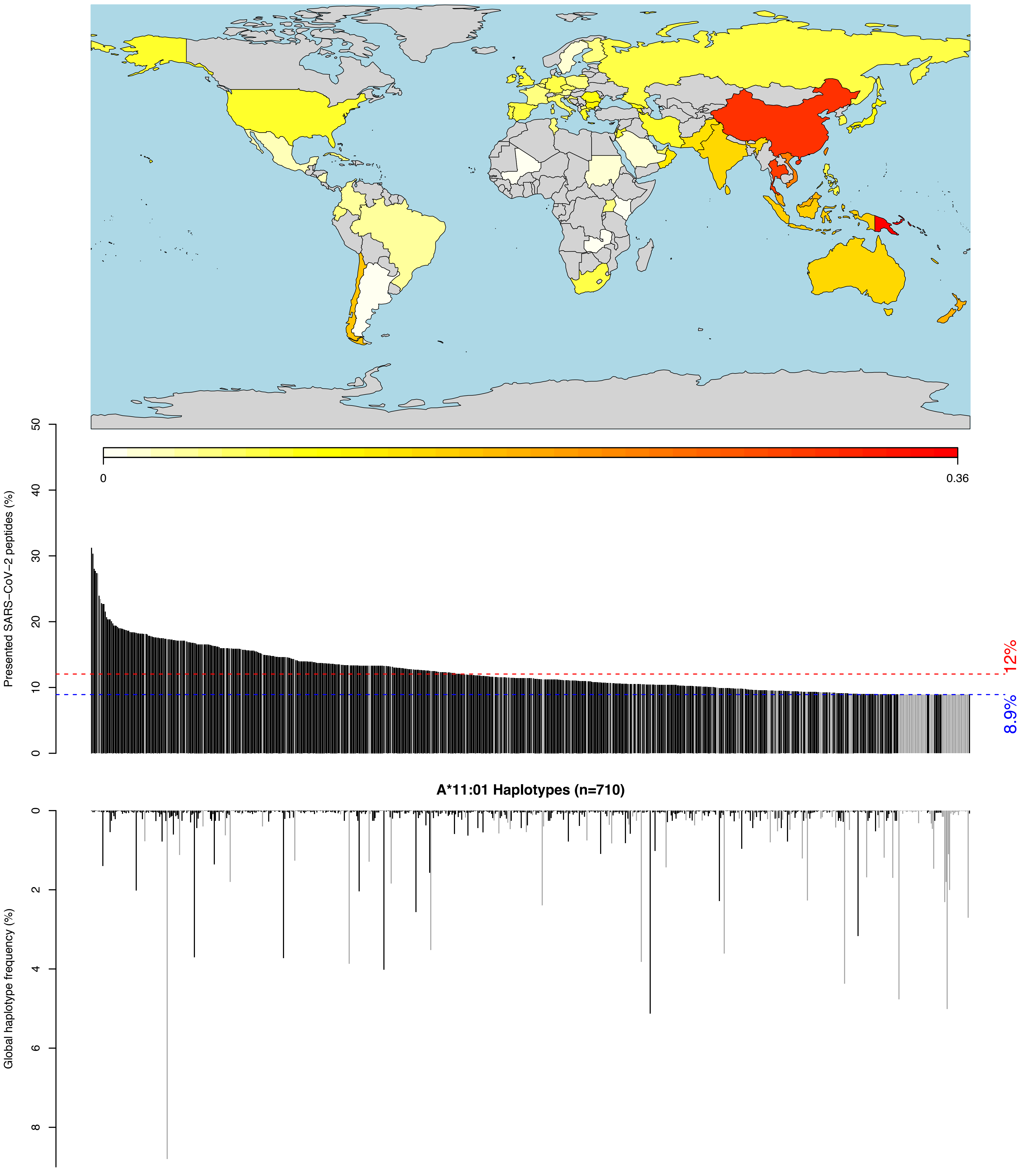

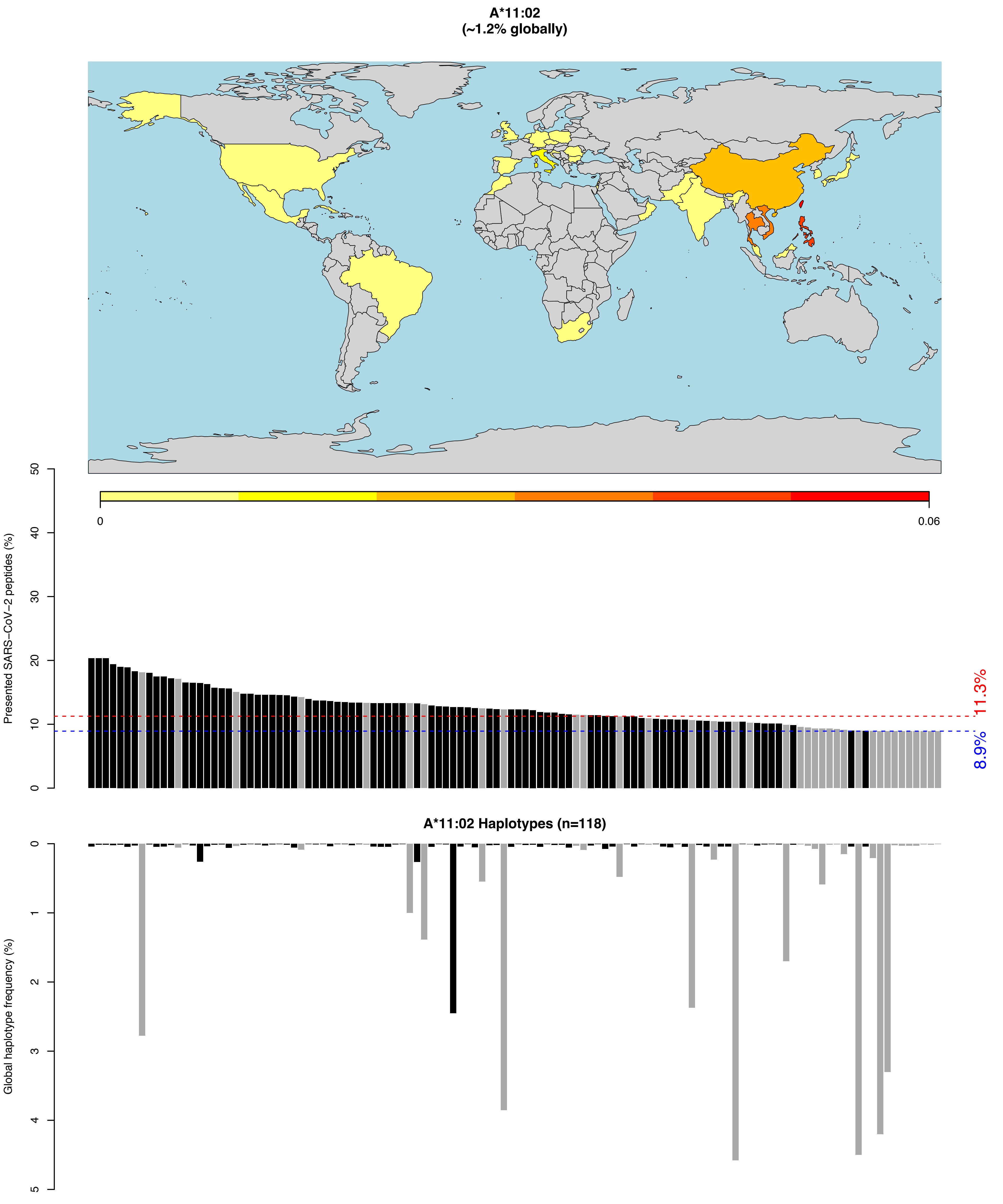
A*11:02
(~1.2% globally)
0
0.06
Presented SARS−CoV−2 peptides (%)
50
40
30
20
10
0
11.3%
8.9%
A*11:02 Haplotypes (n=118)
Global haplotype frequency (%)
0
1
2
3
4
5

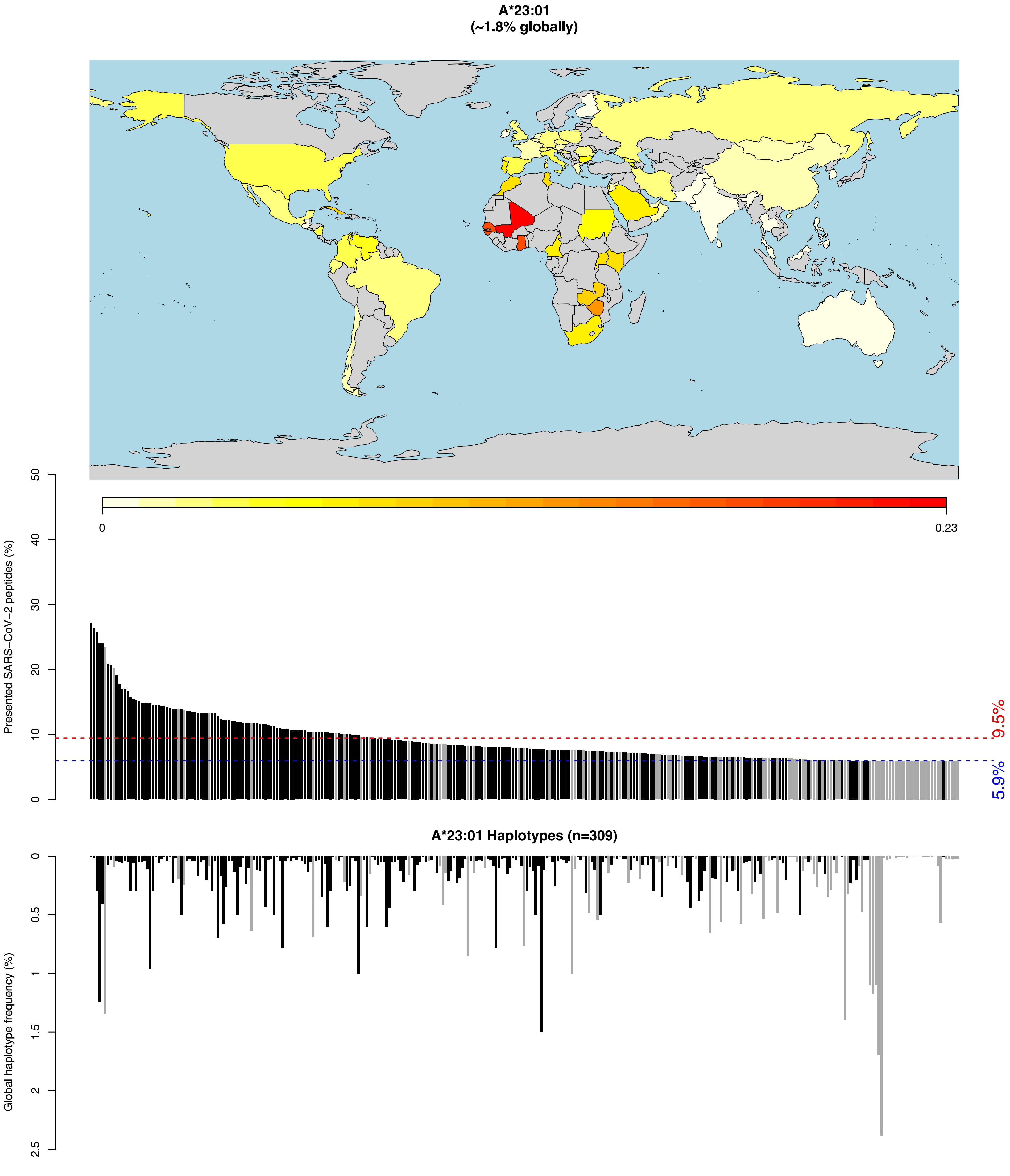
A*23:01
(~1.8% globally)
0
0.23
Presented SARS−CoV−2 peptides (%)
50
40
30
20
10
0
9.5%
5.9%
A*23:01 Haplotypes (n=309)
Global haplotype frequency (%)
0
0.5
1
1.5
2
2.5

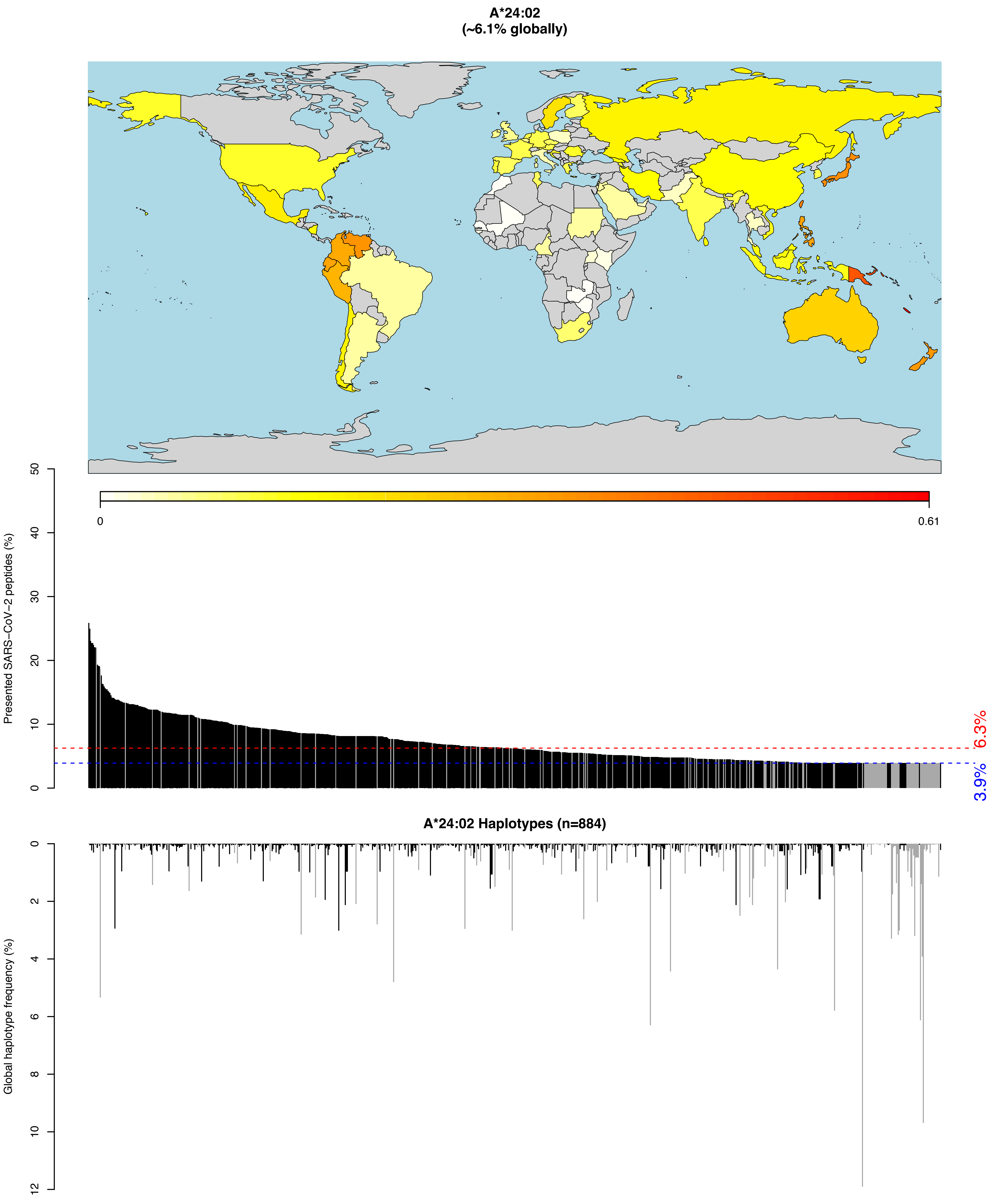

A*24:02
(~6.1% globally)
0
0.61
Presented SARS−CoV−2 peptides (%)
0
10
20
30
40
50
6.3%
3.9%
A*24:02 Haplotypes (n=884)
Global haplotype frequency (%)
0
2
4
6
8
10
12

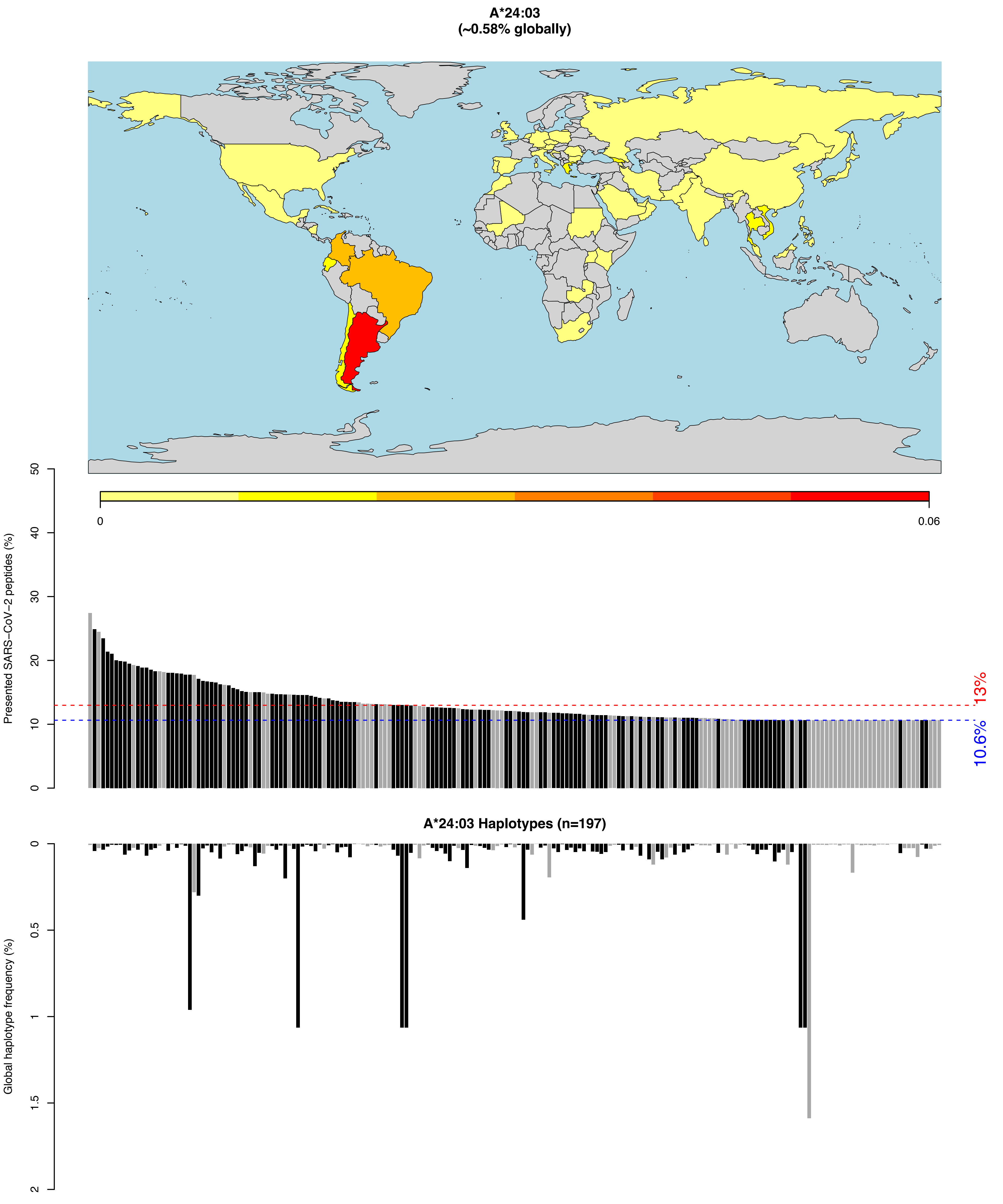

A*24:03
(~0.58% globally)
0
0.06
Presented SARS−CoV−2 peptides (%)
50
40
30
20
10
0
13%
10.6%
A*24:03 Haplotypes (n=197)
Global haplotype frequency (%)
0
0.5
1
1.5
2

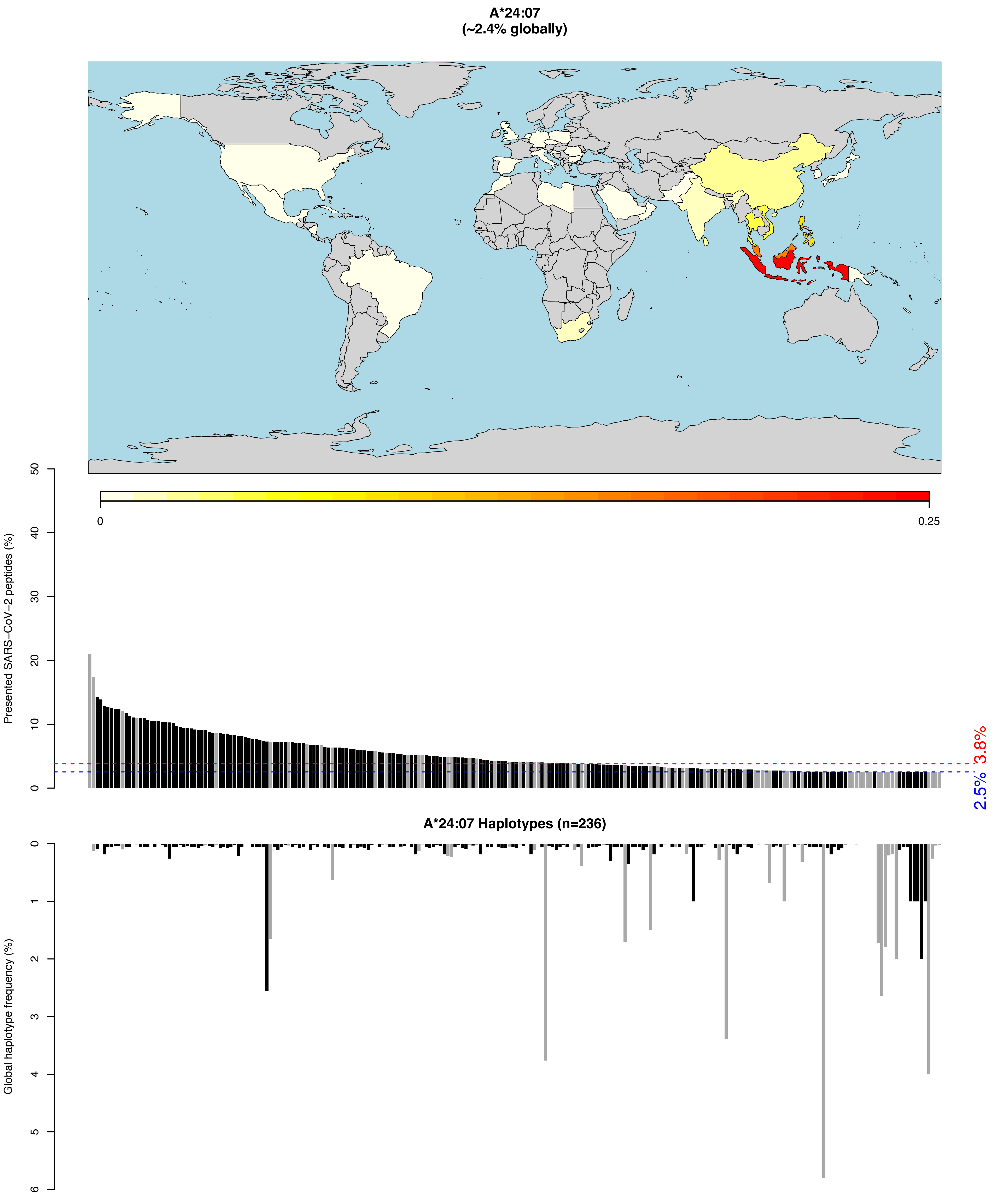

A*24:07
(~2.4% globally)
0
0.25
Presented SARS−CoV−2 peptides (%)
0
10
20
30
40
50
3.8%
2.5%
A*24:07 Haplotypes (n=236)
Global haplotype frequency (%)
0
1
2
3
4
5
6

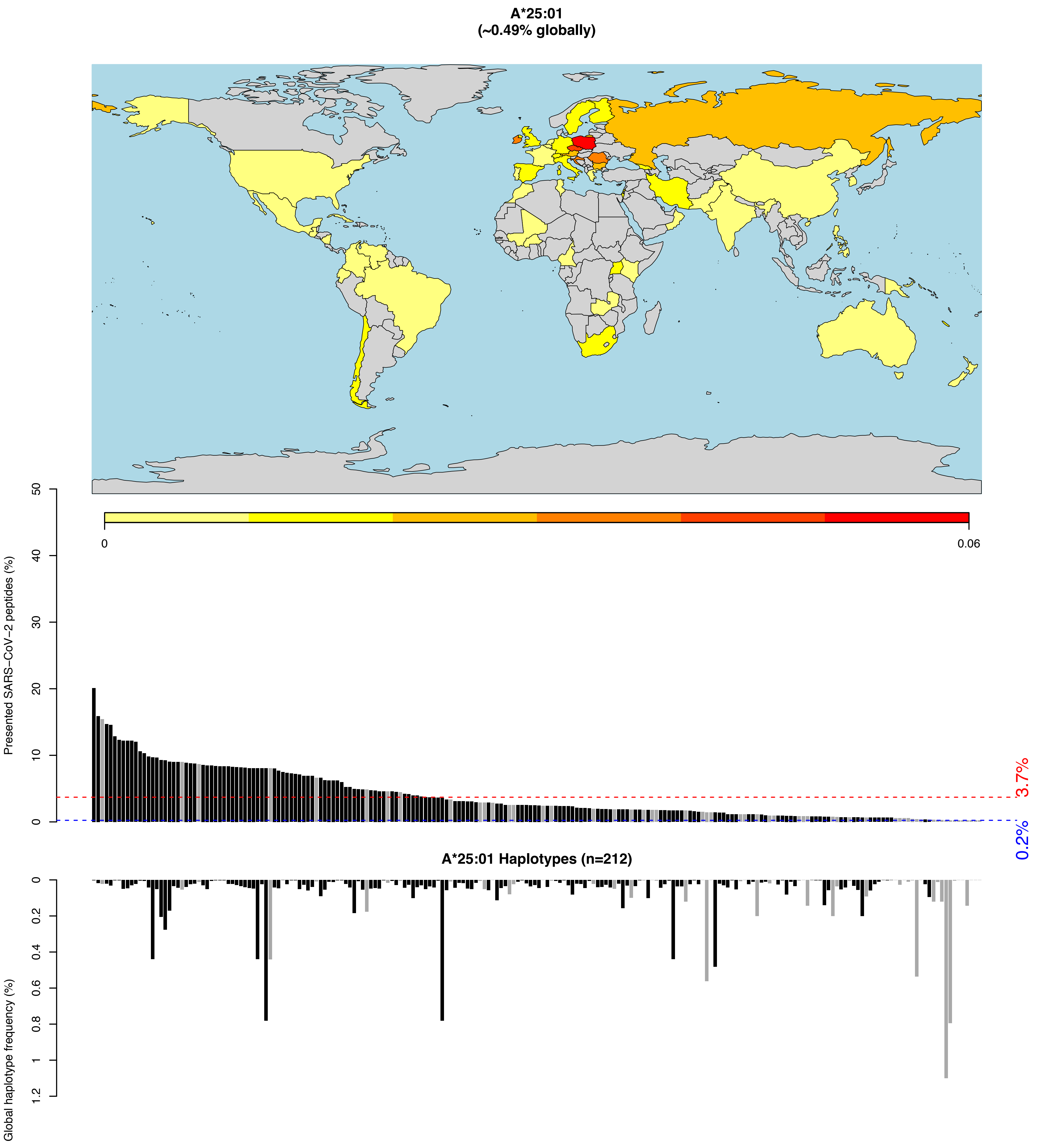

A*25:01
(~0.49% globally)
0
0.06
Presented SARS−CoV−2 peptides (%)
50
40
30
20
10
0
3.7%
0.2%
A*25:01 Haplotypes (n=212)
Global haplotype frequency (%)
0
0.2
0.4
0.6
0.8
1
1.2

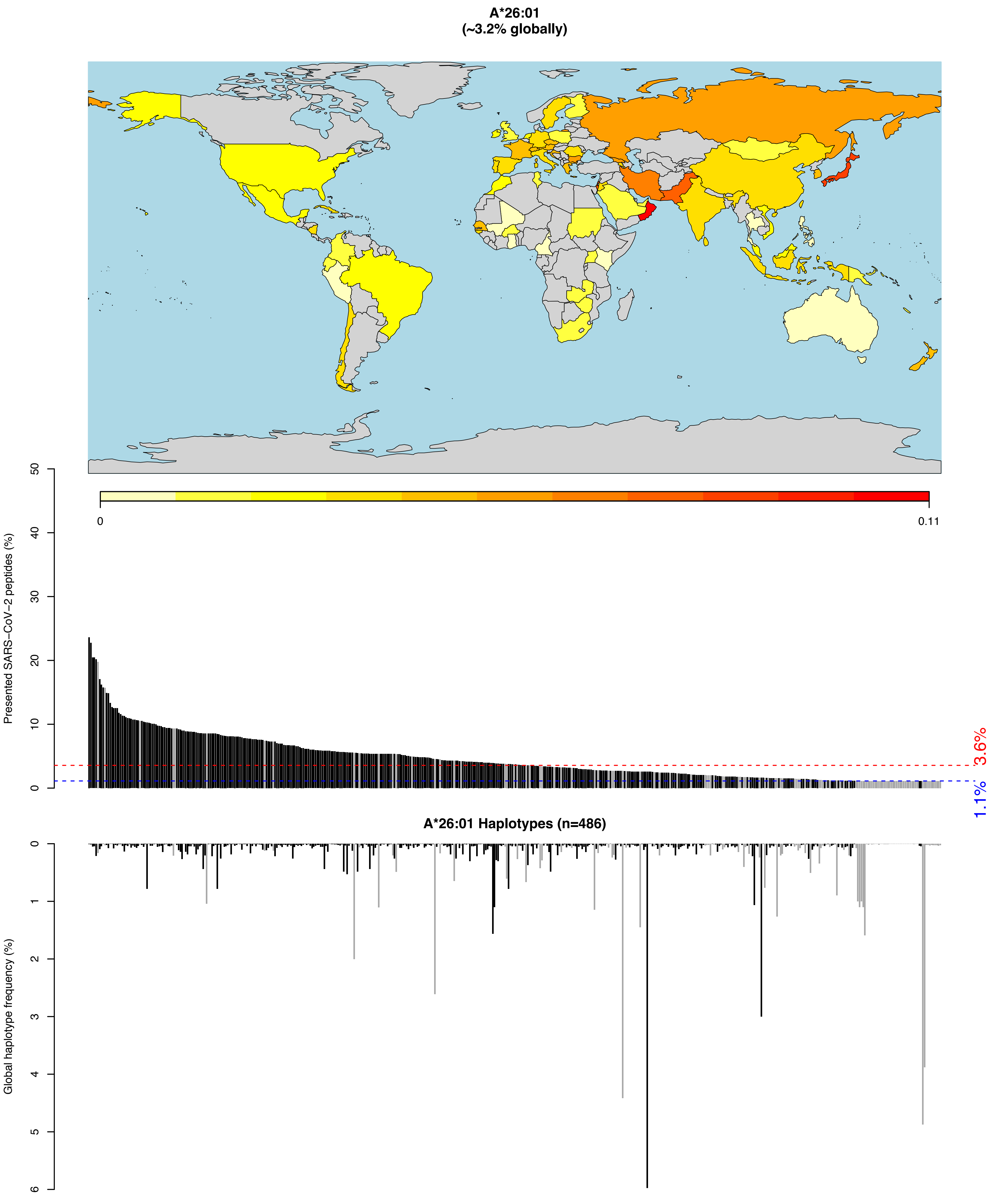

A*26:01
(~3.2% globally)
0
0.11
Presented SARS−CoV−2 peptides (%)
50
40
30
20
10
0
3.6%
1.1%
A*26:01 Haplotypes (n=486)
Global haplotype frequency (%)
0
1
2
3
4
5
6

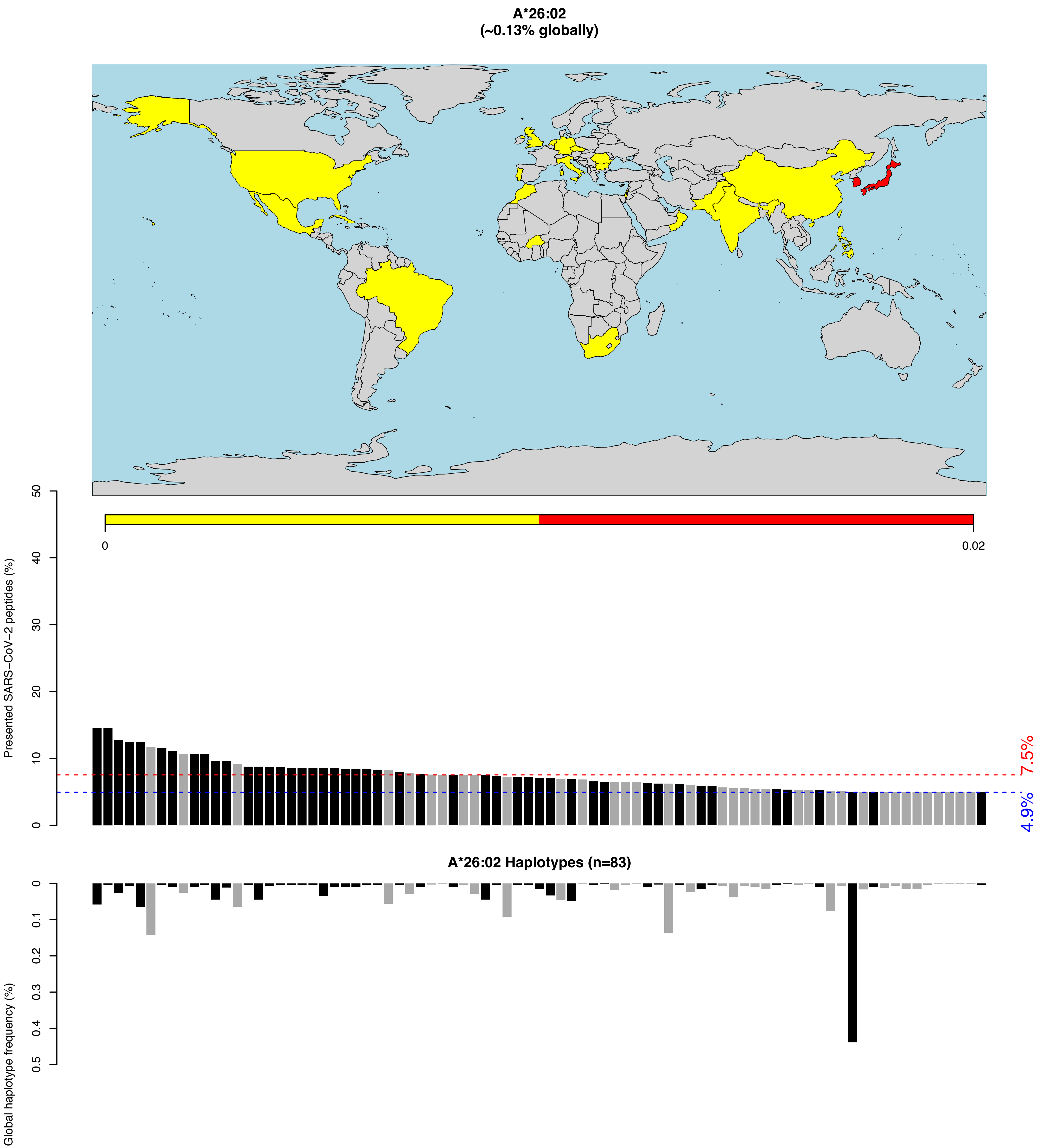
A*26:02
(~0.13% globally)
0
0.02
Presented SARS−CoV−2 peptides (%)
50
40
30
20
10
0
7.5%
4.9%
A*26:02 Haplotypes (n=83)
Global haplotype frequency (%)
0
0.1
0.2
0.3
0.4
0.5

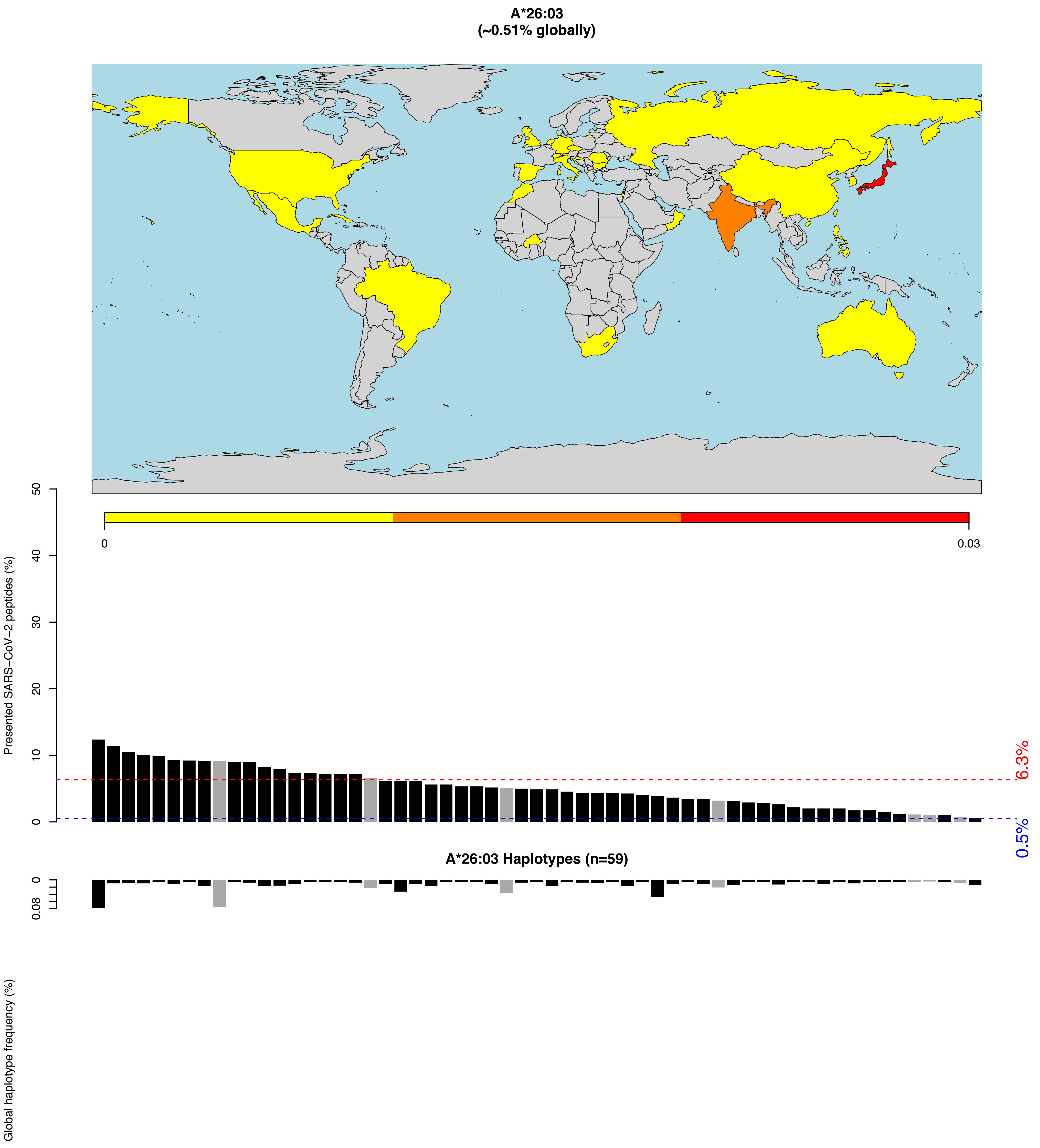
A*26:03
(~0.51% globally)
0
0.03
50
40
30
20
10
0
Presented SARS−CoV−2 peptides (%)
6.3%
0.5%
A*26:03 Haplotypes (n=59)
0
0.08
Global haplotype frequency (%)

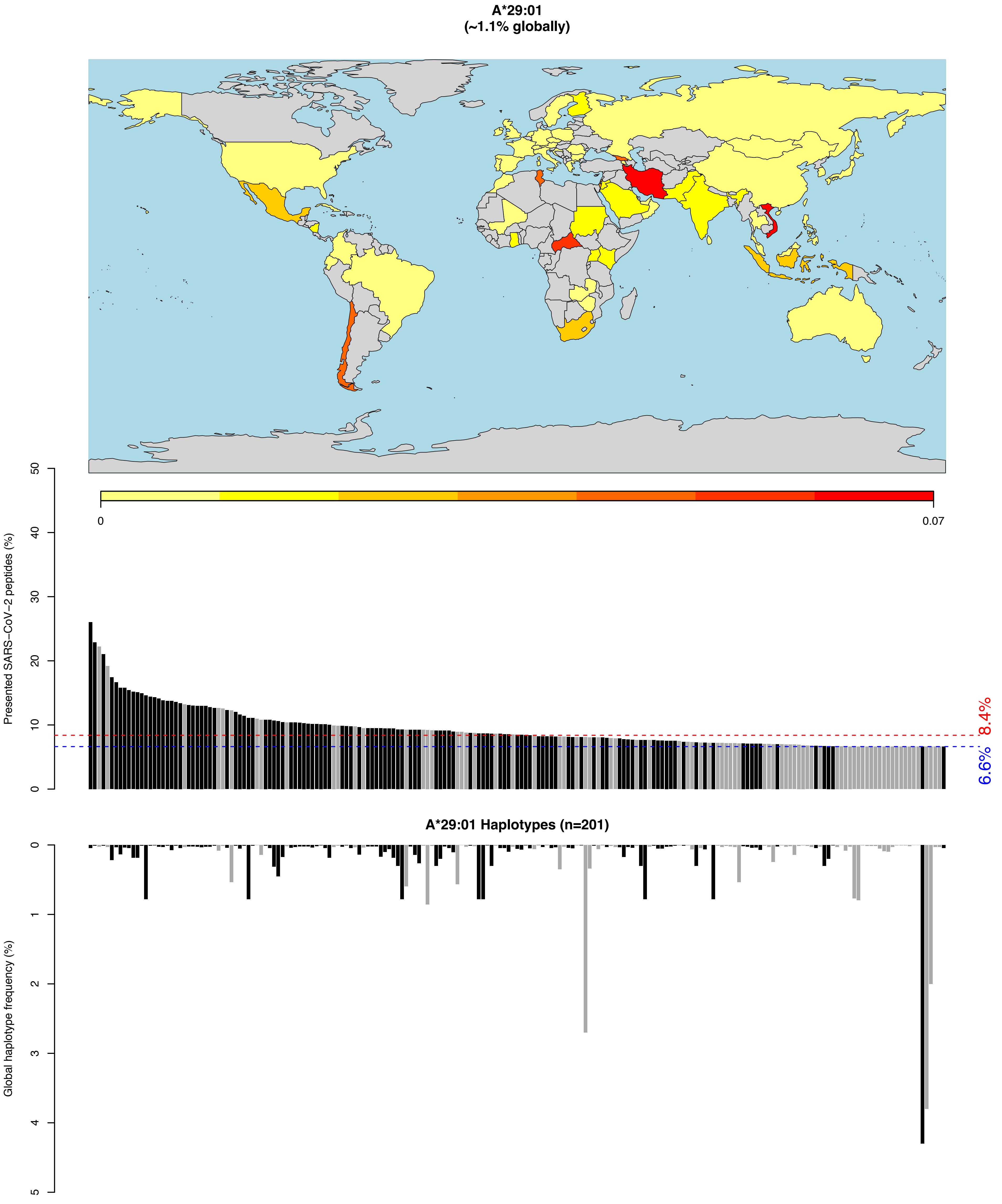
A*29:01
(~1.1% globally)
0
0.07
Presented SARS−CoV−2 peptides (%)
0
10
20
30
40
50
8.4%
6.6%
A*29:01 Haplotypes (n=201)
Global haplotype frequency (%)
0
1
2
3
4
5

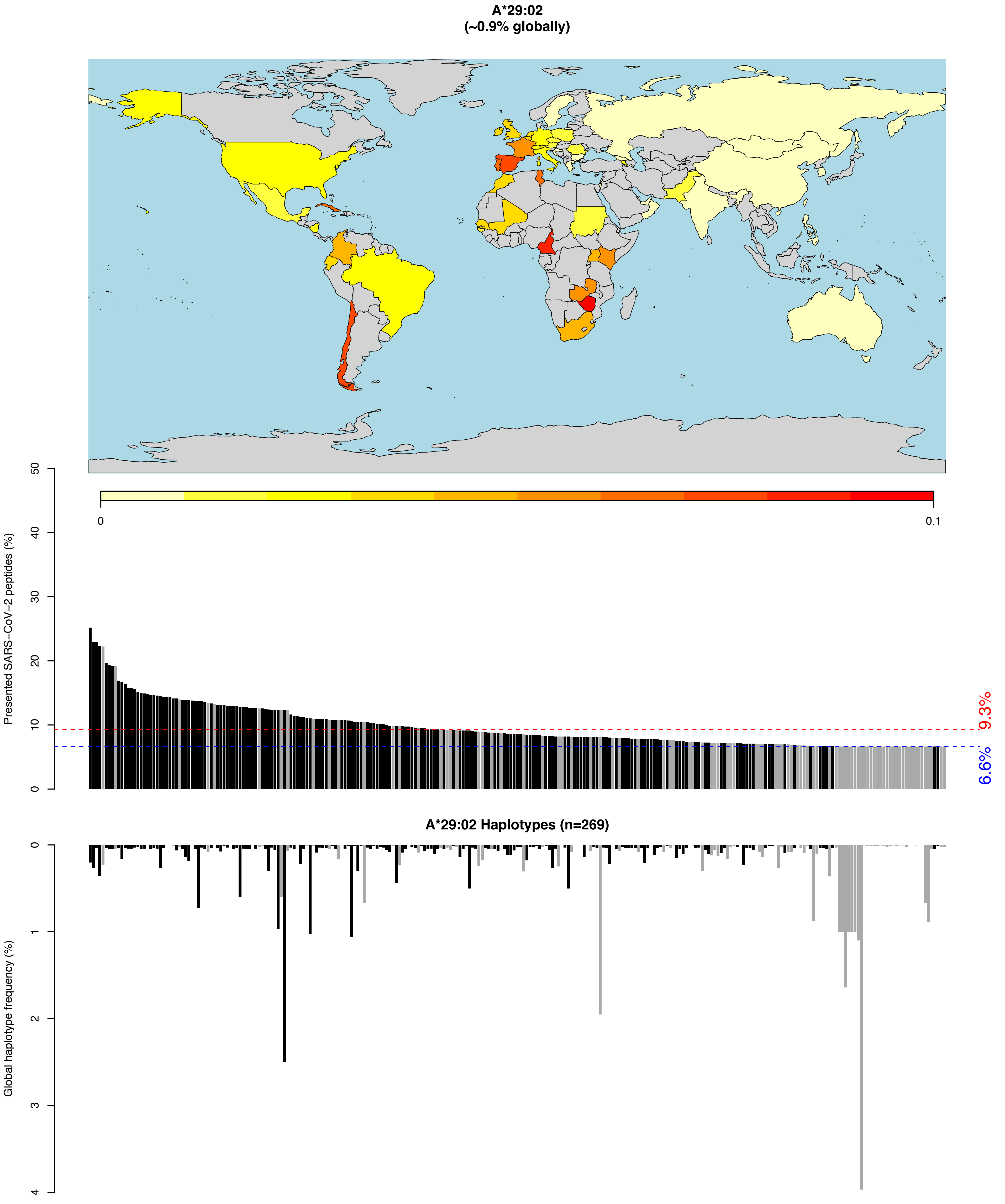
A*29:02
(~0.9% globally)
0
0.1
Presented SARS−CoV−2 peptides (%)
50
40
30
20
10
0
9.3%
6.6%
A*29:02 Haplotypes (n=269)
Global haplotype frequency (%)
0
1
2
3
4

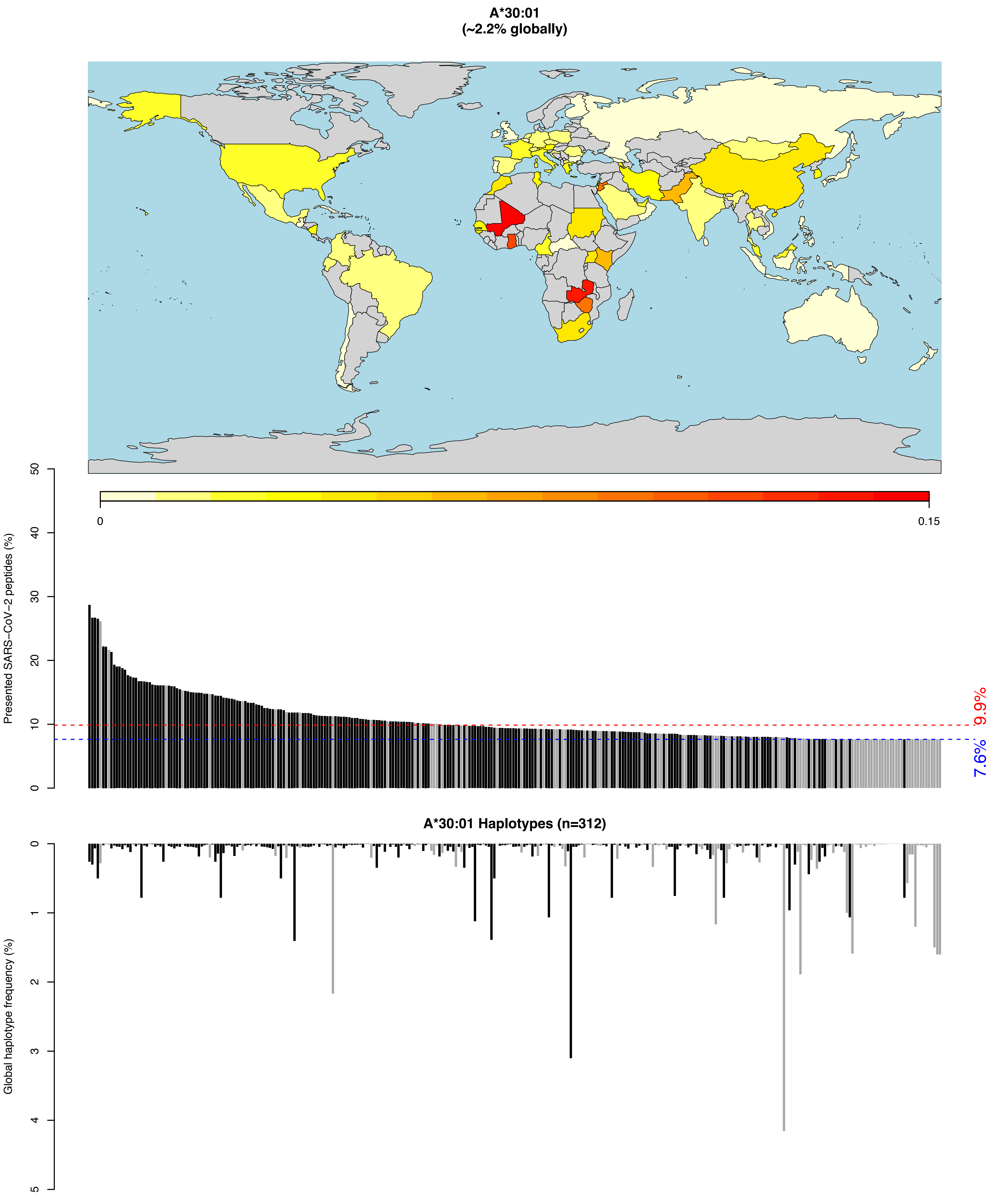

A*30:01
(~2.2% globally)
0
0.15
Presented SARS−CoV−2 peptides (%)
50
40
30
20
10
0
9.9%
7.6%
A*30:01 Haplotypes (n=312)
Global haplotype frequency (%)
0
1
2
3
4
5

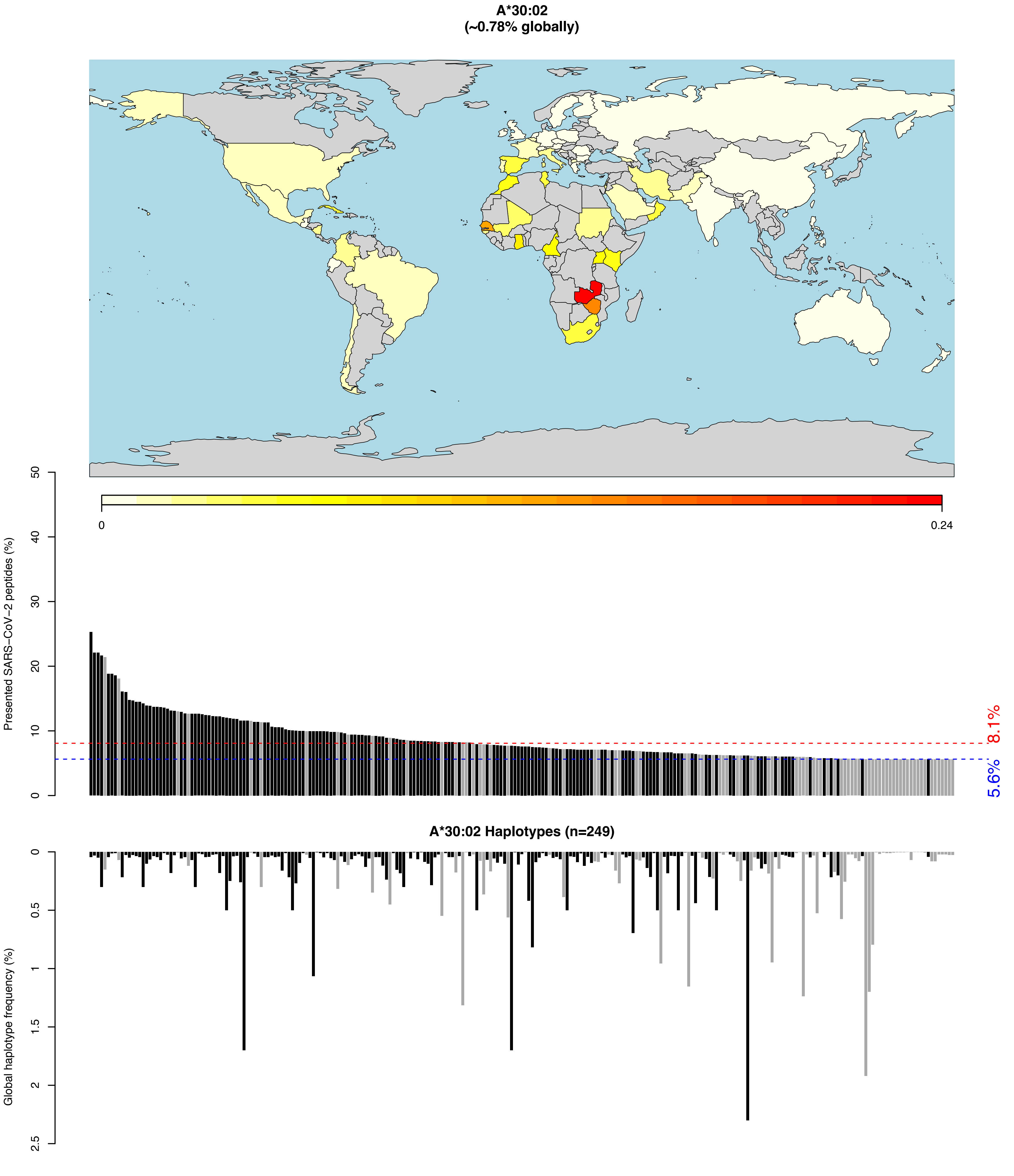

A*30:02
(~0.78% globally)
0
0.24
Presented SARS−CoV−2 peptides (%)
50
40
30
20
10
0
8.1%
5.6%
A*30:02 Haplotypes (n=249)
Global haplotype frequency (%)
0
0.5
1
1.5
2
2.5

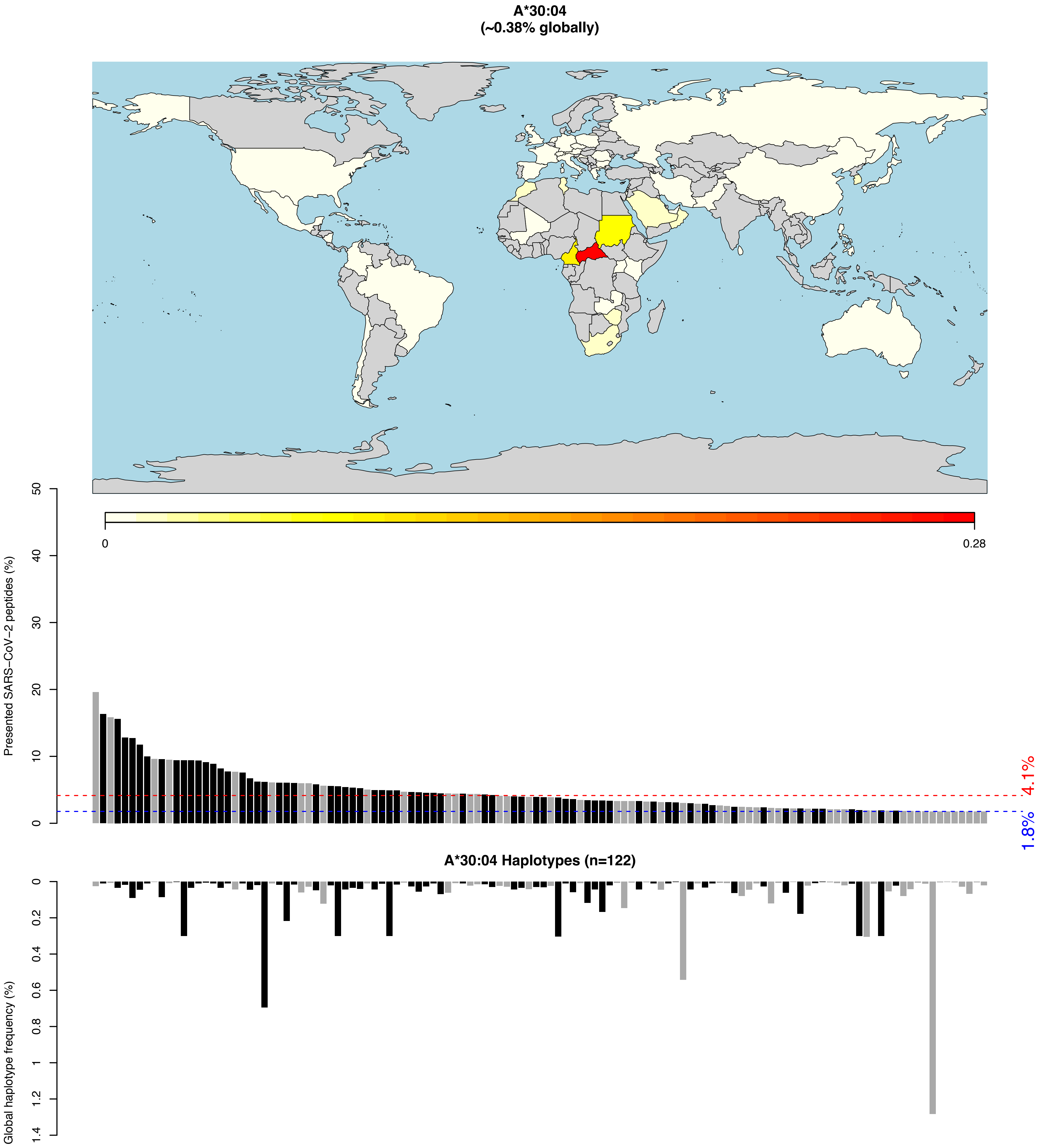
A*30:04
(~0.38% globally)
0
0.28
Presented SARS−CoV−2 peptides (%)
50
40
30
20
10
0
4.1%
1.8%
A*30:04 Haplotypes (n=122)
Global haplotype frequency (%)
0
0.2
0.4
0.6
0.8
1
1.2
1.4

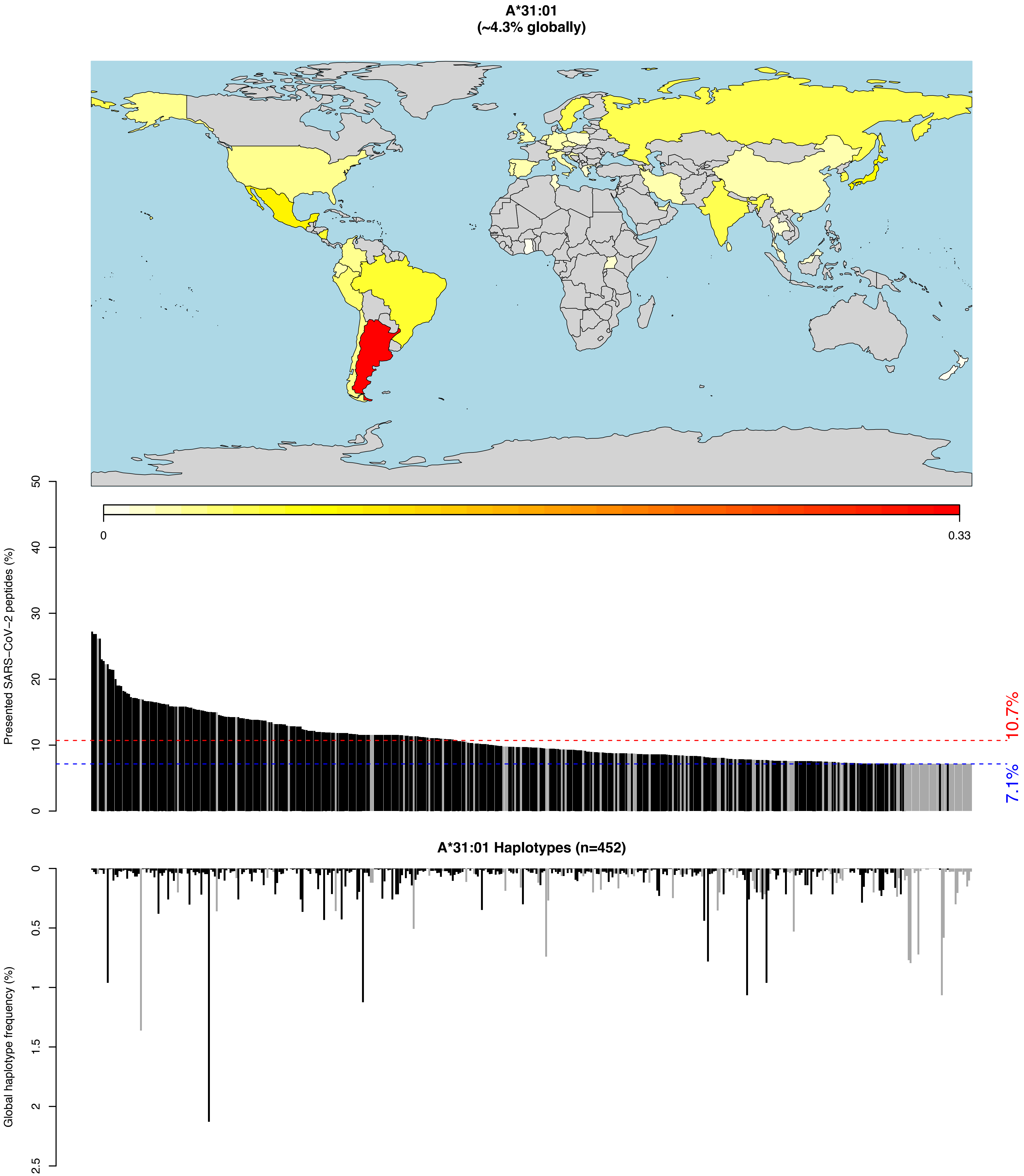

A*31:01
(~4.3% globally)
0
0.33
Presented SARS−CoV−2 peptides (%)
50
40
30
20
10
0
10.7%
7.1%
A*31:01 Haplotypes (n=452)
Global haplotype frequency (%)
0
0.5
1
1.5
2
2.5

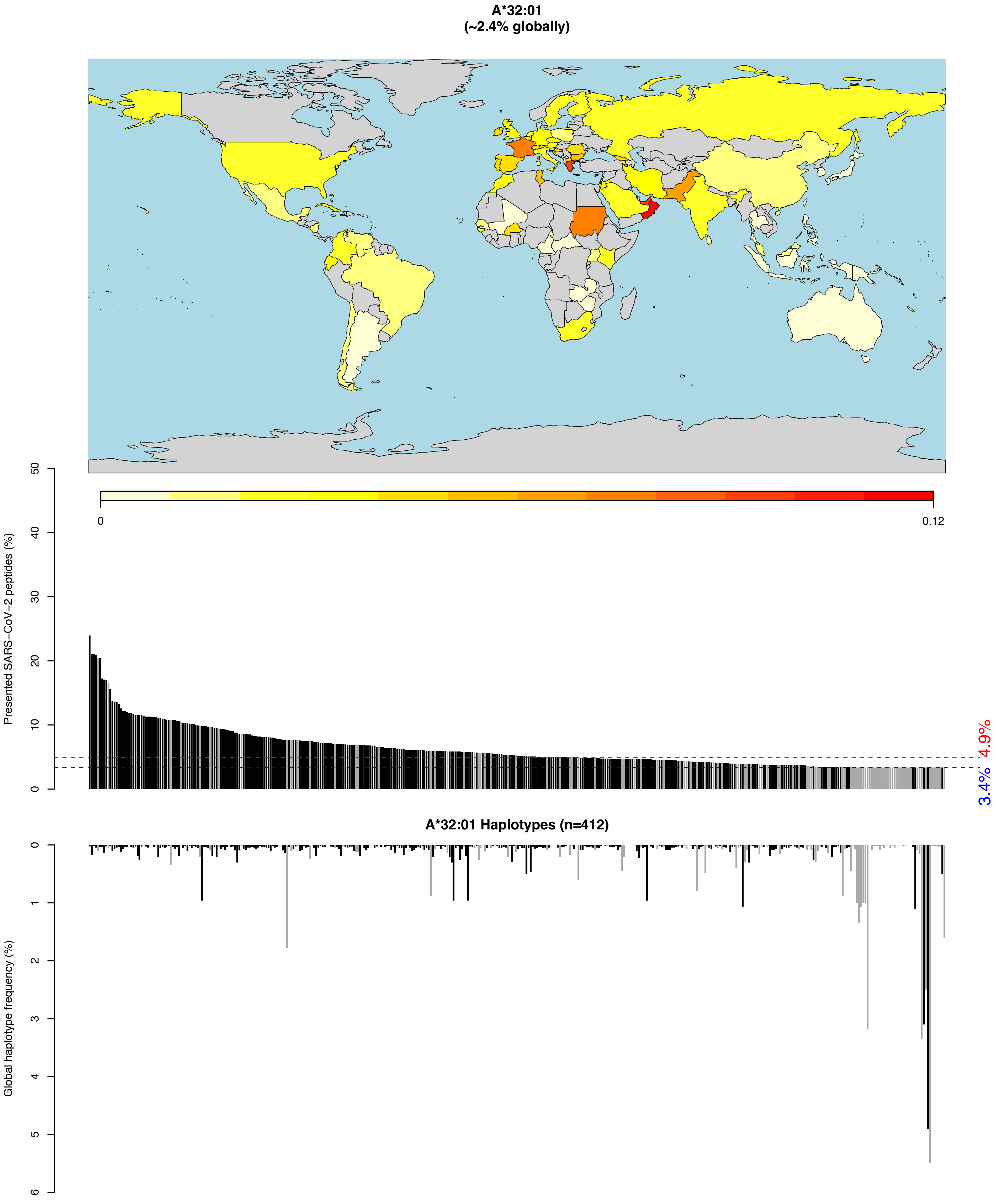
A*32:01
(~2.4% globally)
0
0.12
Presented SARS−CoV−2 peptides (%)
50
40
30
20
10
0
4.9%
3.4%
A*32:01 Haplotypes (n=412)
Global haplotype frequency (%)
0
1
2
3
4
5
6

A*33:01
(~0.99% globally)

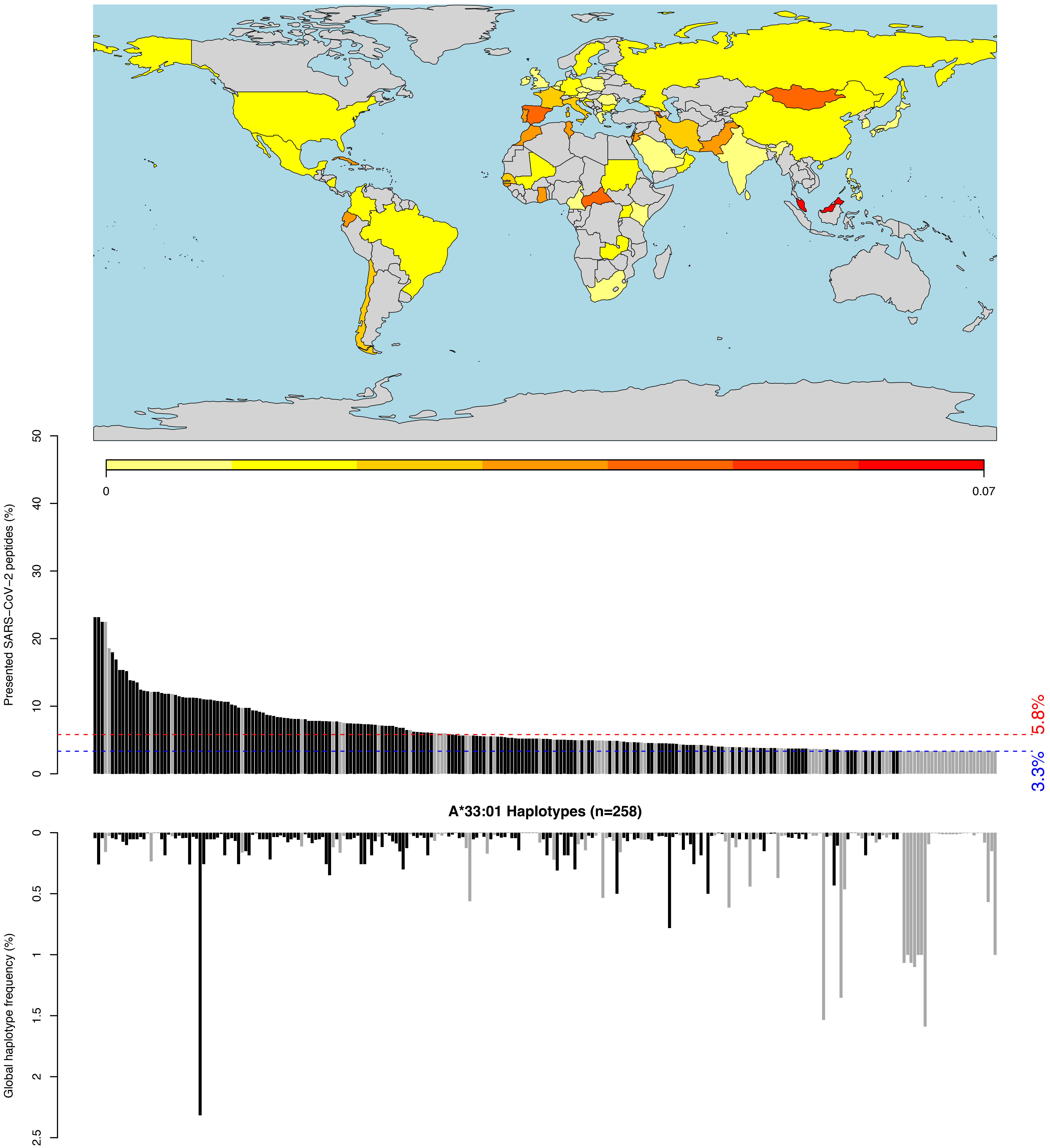

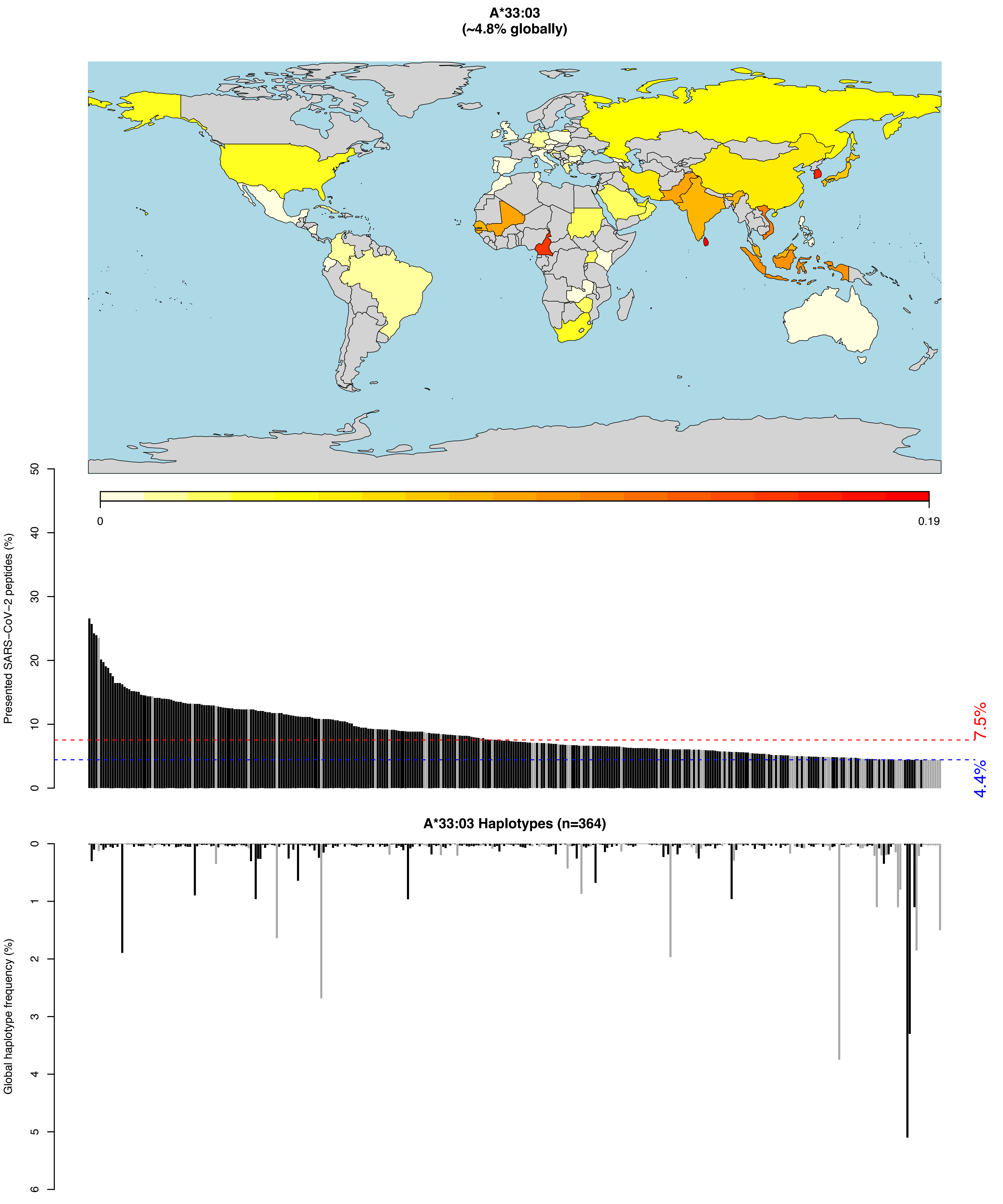

A*33:03
(~4.8% globally)
0
0.19
Presented SARS−CoV−2 peptides (%)
50
40
30
20
10
0
7.5%
4.4%
A*33:03 Haplotypes (n=364)
Global haplotype frequency (%)
0
1
2
3
4
5
6

A*34:01
(~1.3% globally)

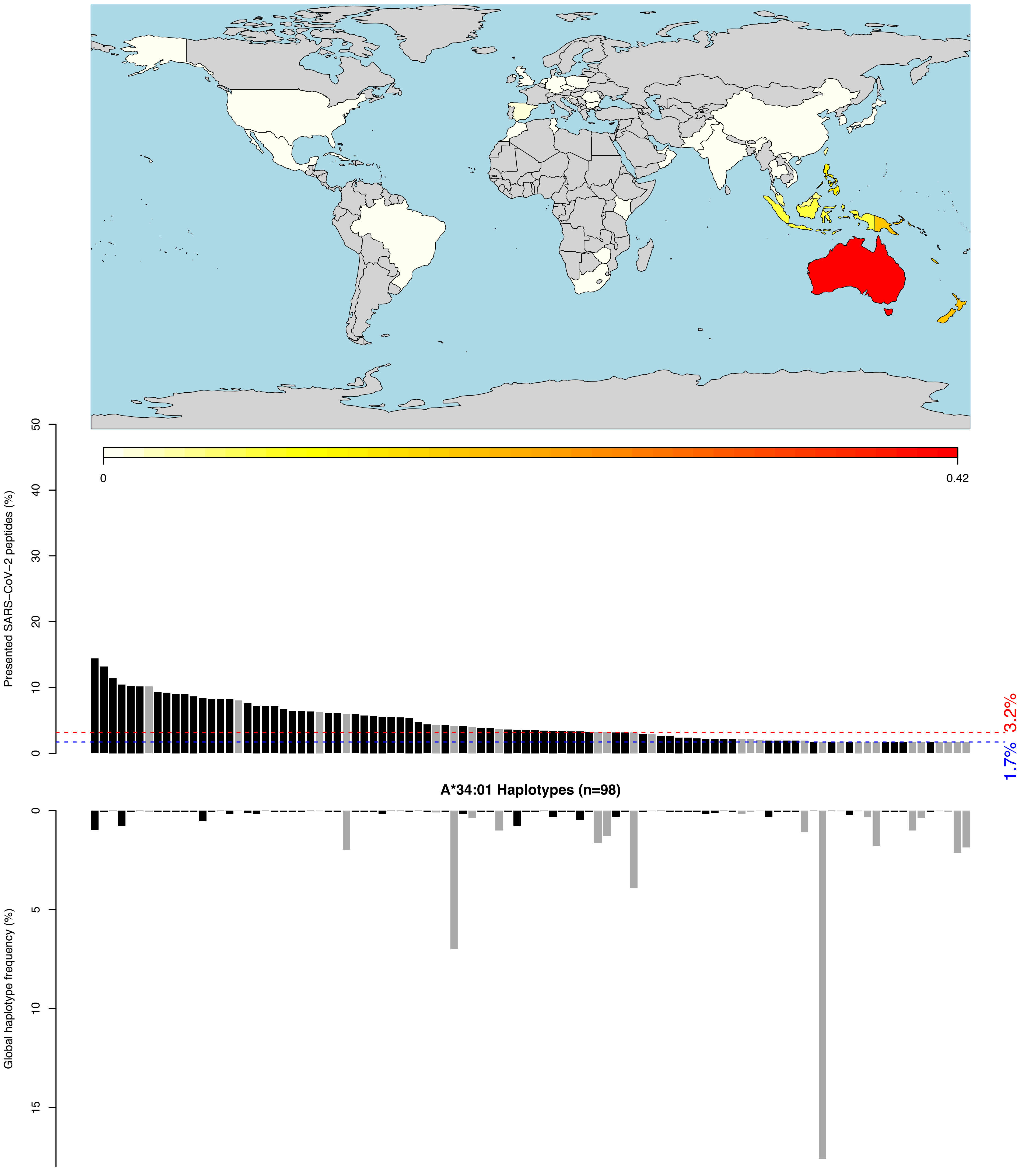

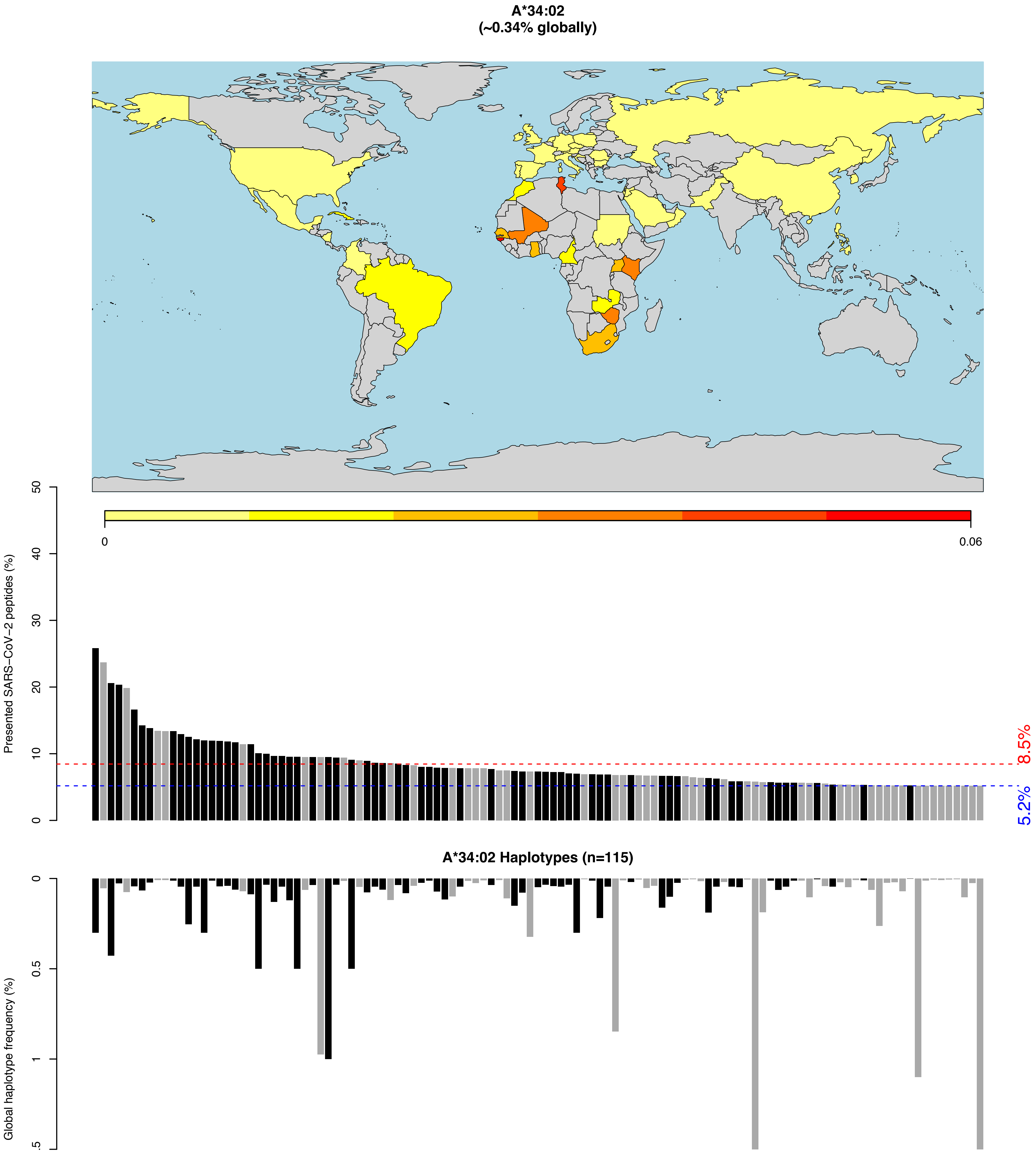
A*34:02
(~0.34% globally)
0
0.06
50
40
30
20
10
0
Presented SARS−CoV−2 peptides (%)
8.5%
5.2%
A*34:02 Haplotypes (n=115)
0
0.5
1
1.5
Global haplotype frequency (%)

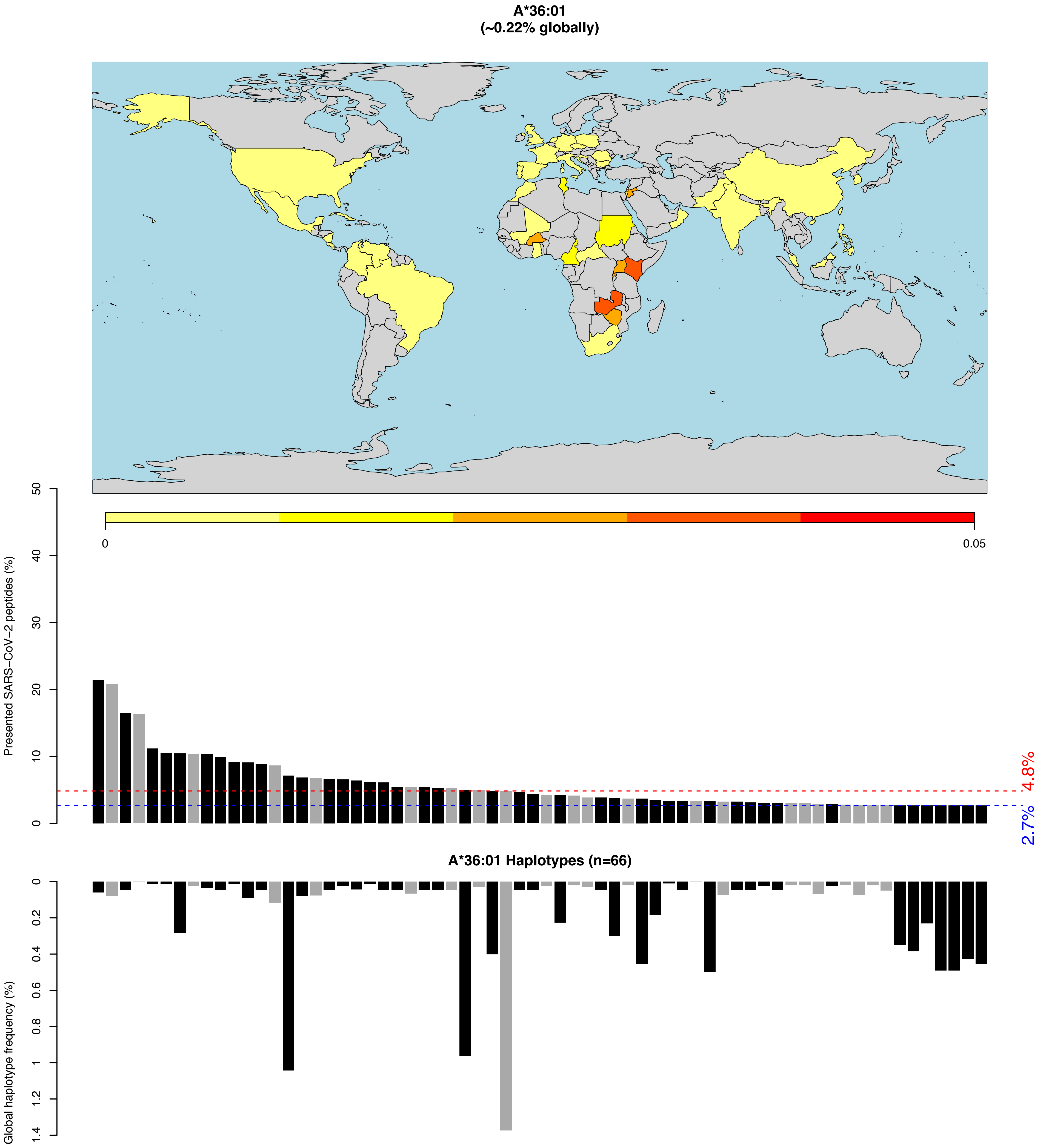
A*36:01
(~0.22% globally)
0
0.05
Presented SARS−CoV−2 peptides (%)
50
40
30
20
10
0
4.8%
2.7%
A*36:01 Haplotypes (n=66)
Global haplotype frequency (%)
0
0.2
0.4
0.6
0.8
1
1.2
1.4

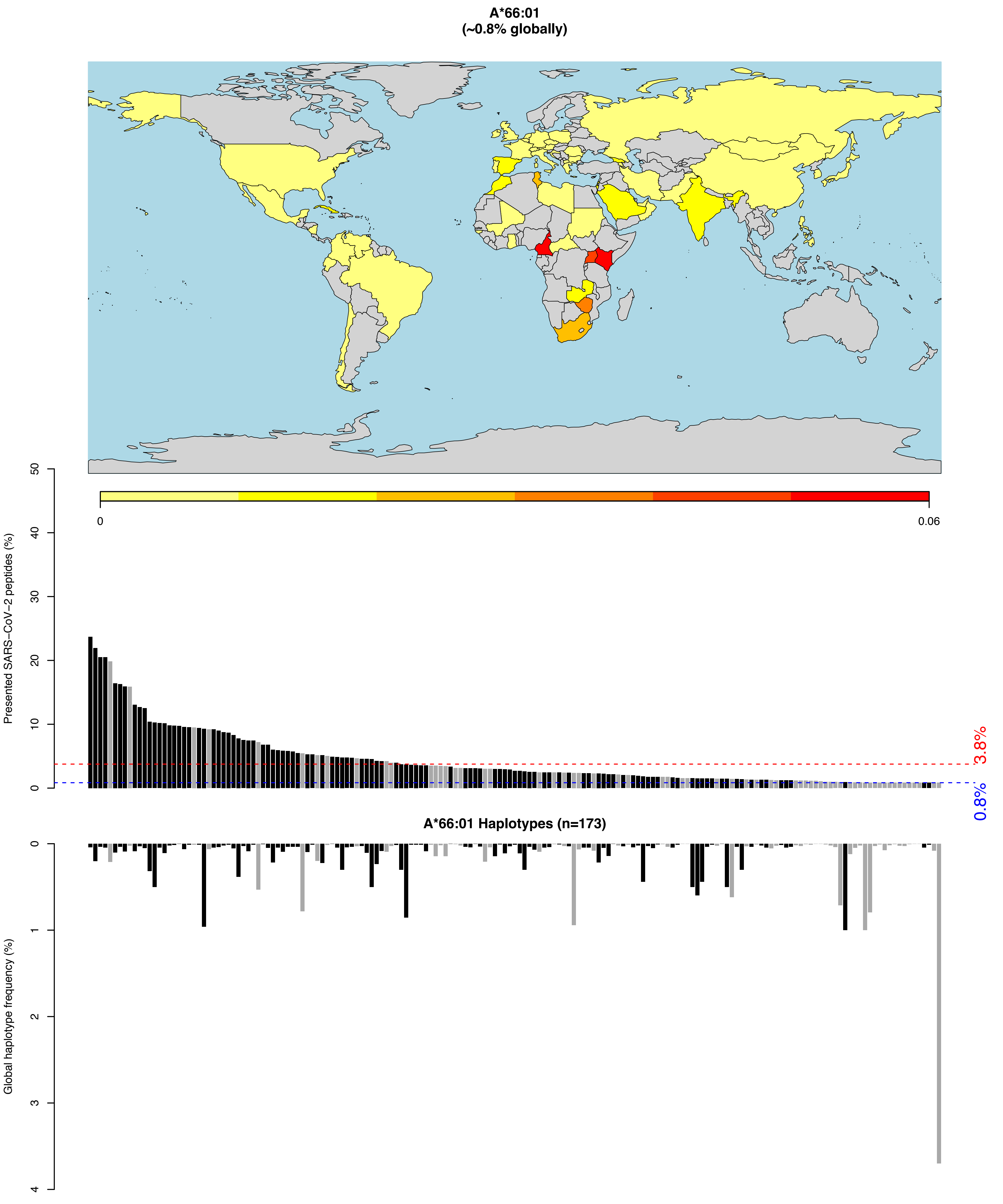

A*66:01
(~0.8% globally)
0
0.06
Presented SARS−CoV−2 peptides (%)
50
40
30
20
10
0
3.8%
0.8%
A*66:01 Haplotypes (n=173)
Global haplotype frequency (%)
0
1
2
3
4

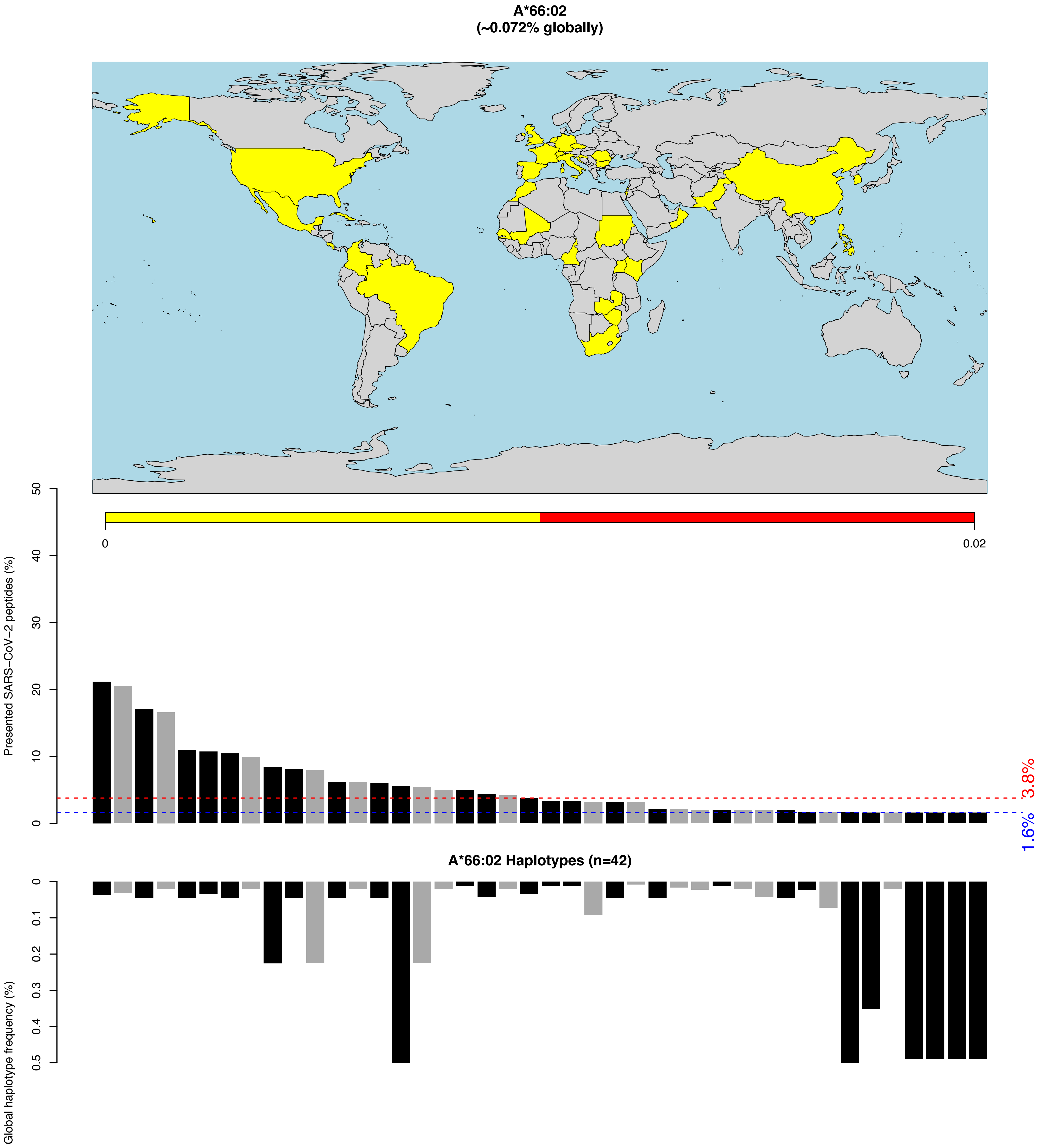

A*66:02
(~0.072% globally)
0
0.02
Presented SARS−CoV−2 peptides (%)
50
40
30
20
10
0
3.8%
1.6%
A*66:02 Haplotypes (n=42)
Global haplotype frequency (%)
0
0.1
0.2
0.3
0.4
0.5

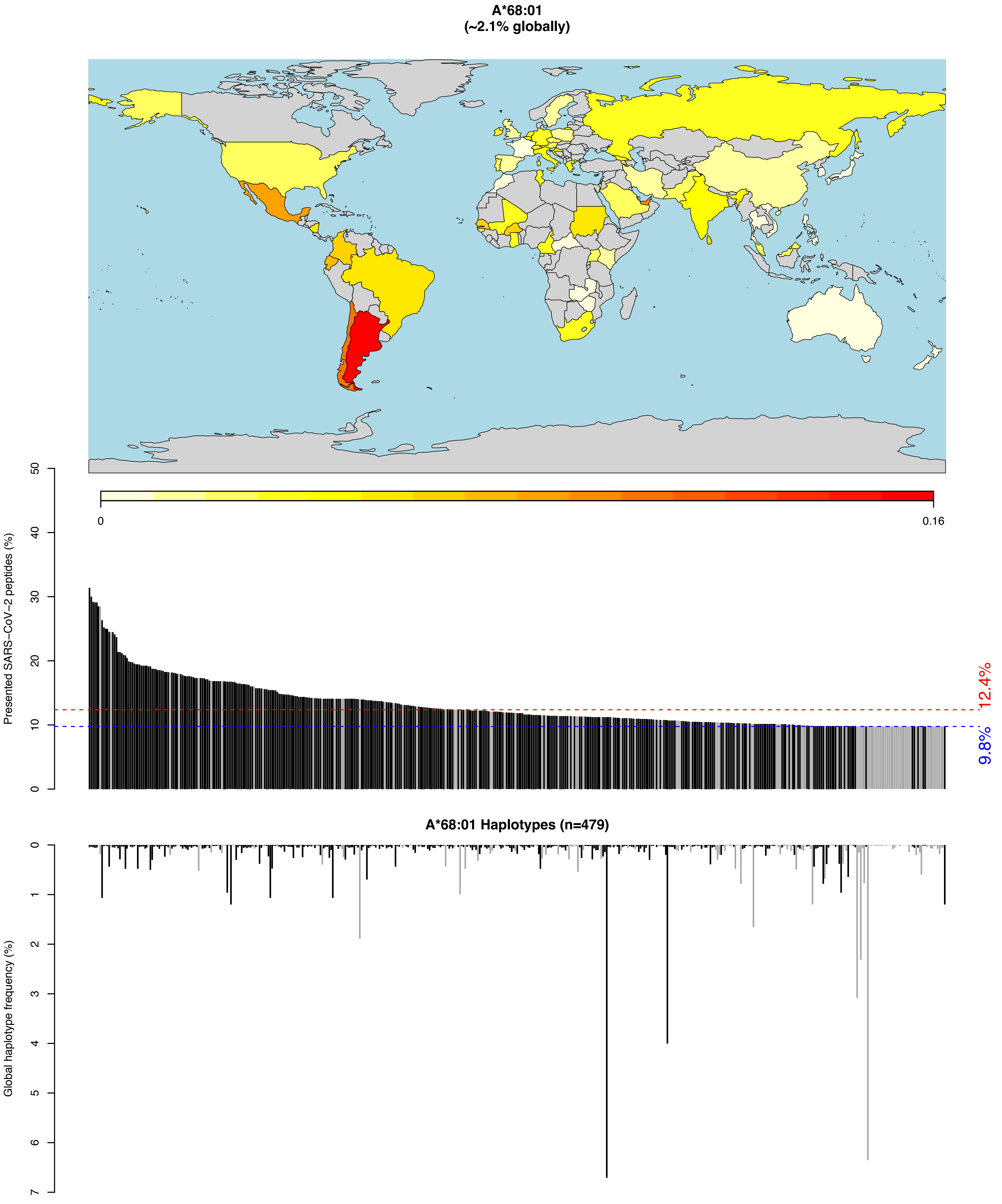
A*68:01
(~2.1% globally)
0
0.16
Presented SARS−CoV−2 peptides (%)
50
40
30
20
10
0
12.4%
9.8%
A*68:01 Haplotypes (n=479)
Global haplotype frequency (%)
0
1
2
3
4
5
6
7

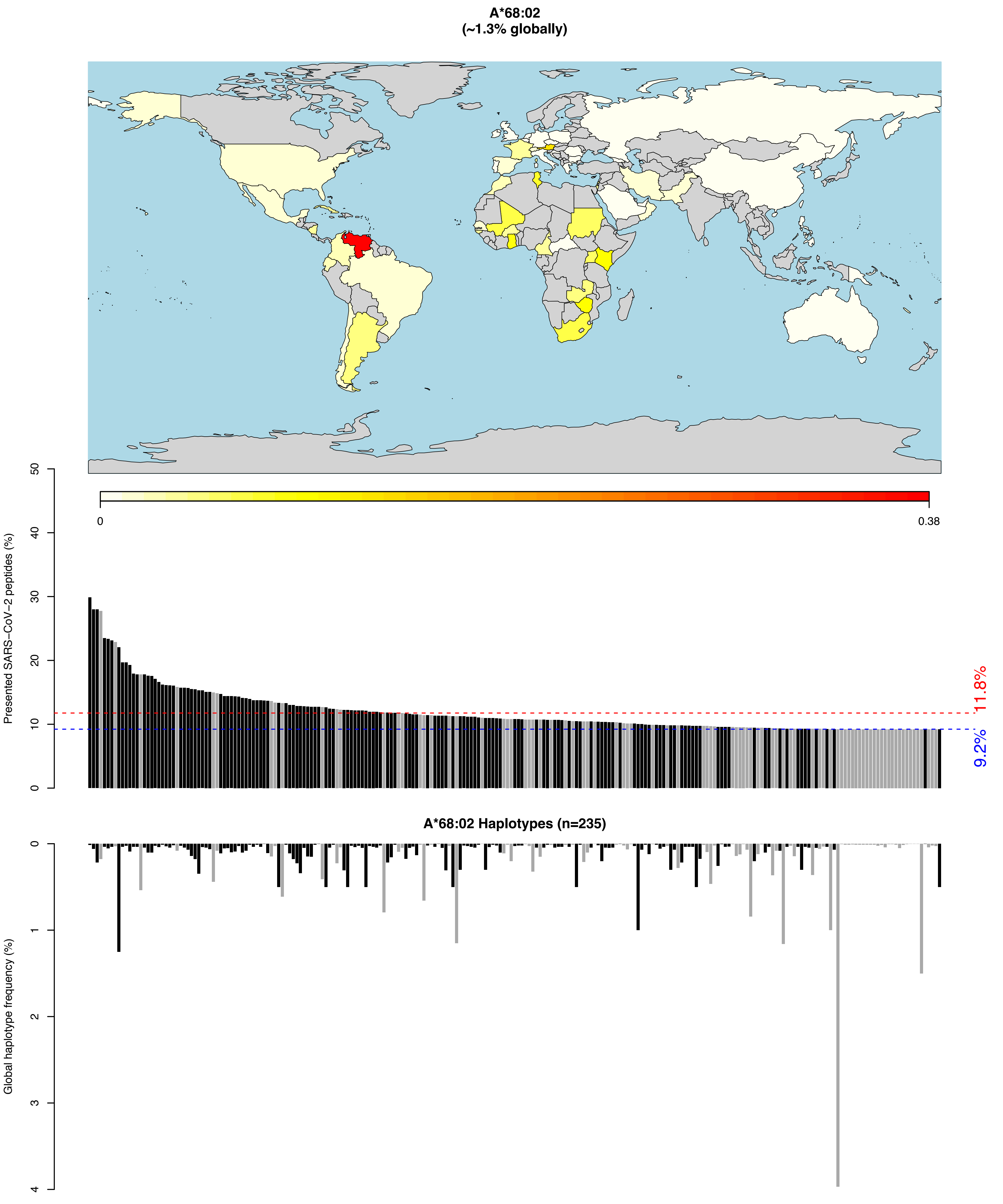
A*68:02
(~1.3% globally)
0
0.38
Presented SARS−CoV−2 peptides (%)
0
10
20
30
40
50
11.8%
9.2%
A*68:02 Haplotypes (n=235)
Global haplotype frequency (%)
0
1
2
3
4

A*74:01
(~0.59% globally)

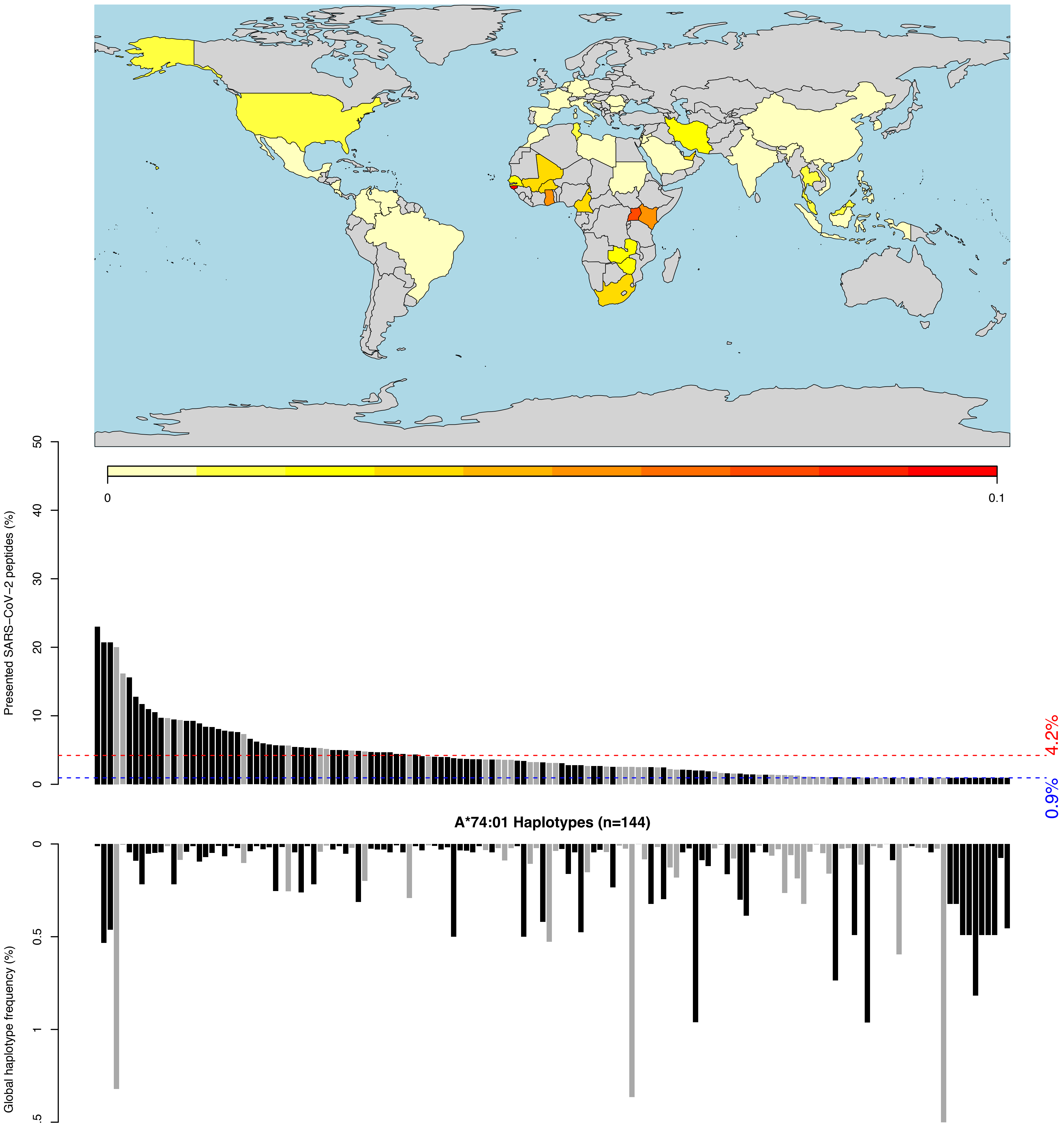

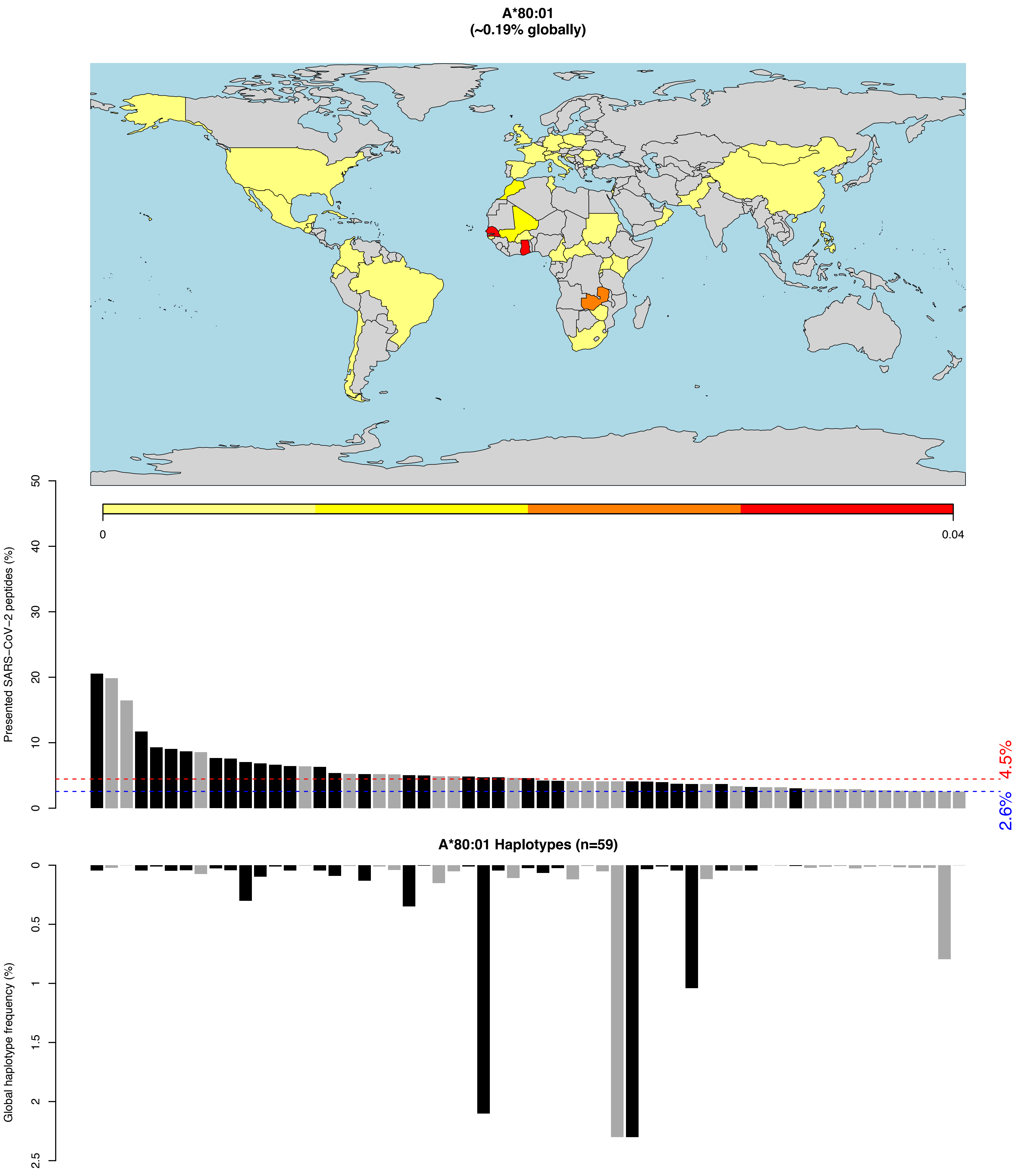

A*80:01
(~0.19% globally)
0
0.04
Presented SARS−CoV−2 peptides (%)
50
40
30
20
10
0
4.5%
2.6%
A*80:01 Haplotypes (n=59)
Global haplotype frequency (%)
0
0.5
1
1.5
2
2.5

B*07:02
(~2.4% globally)

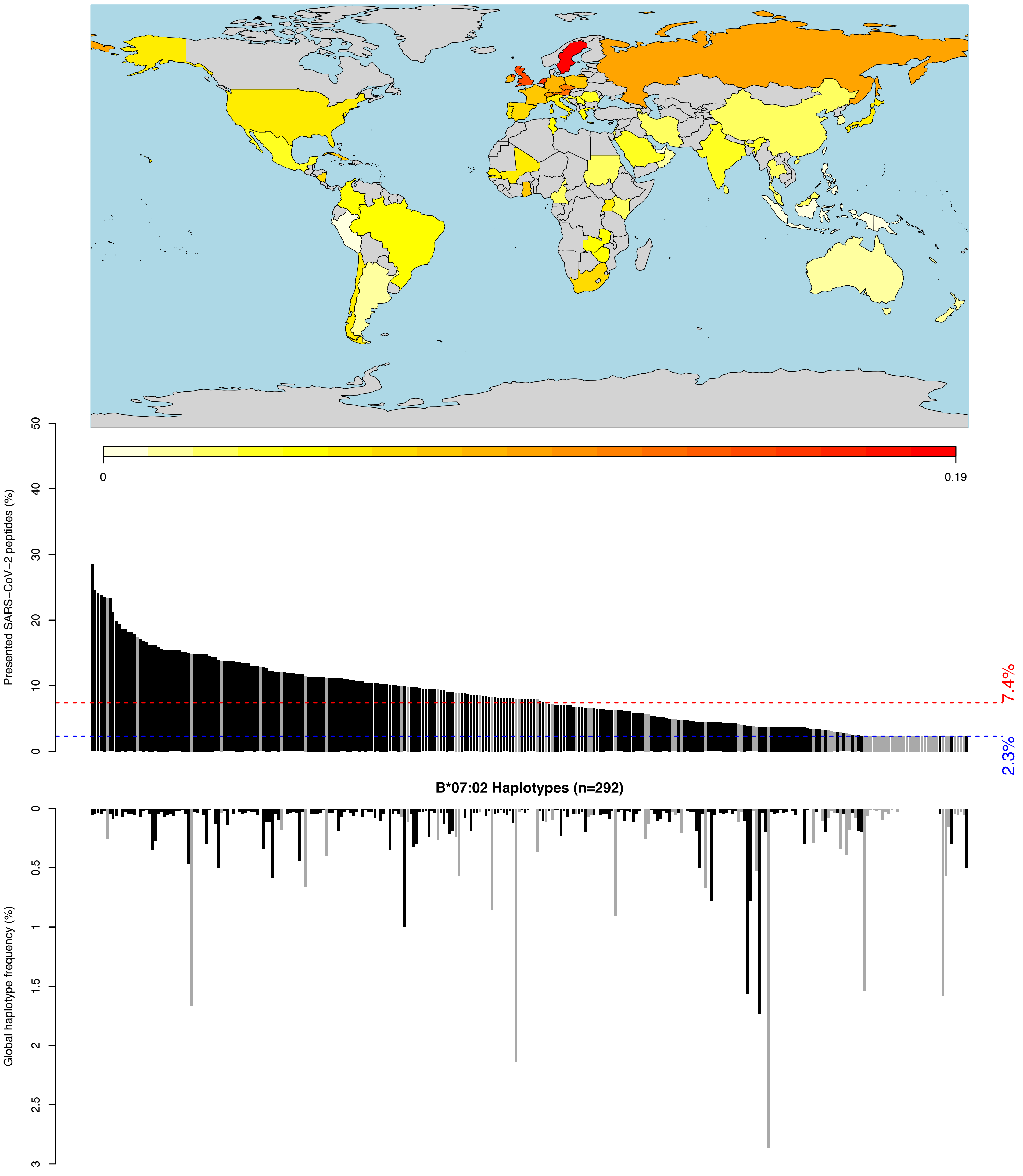

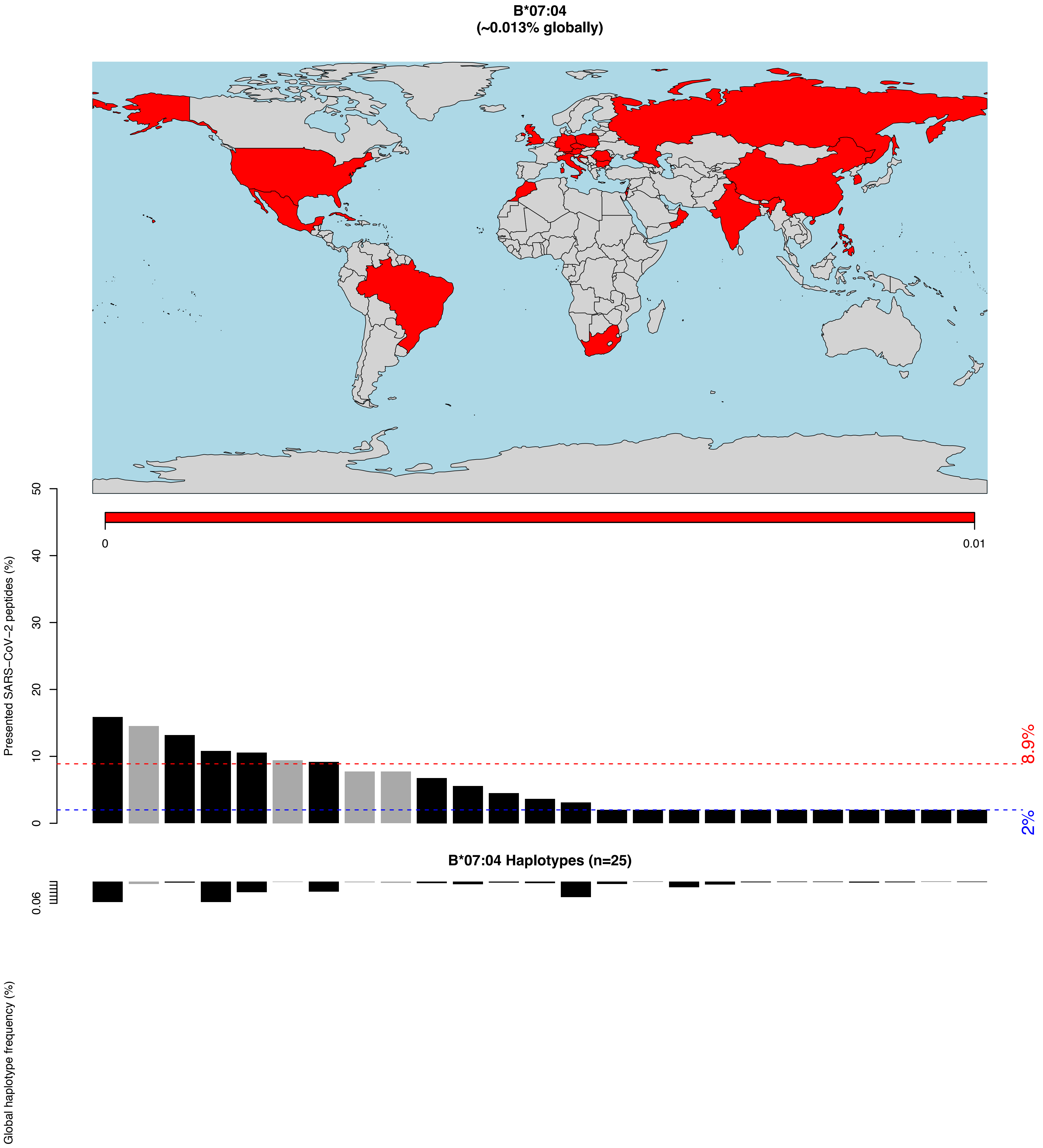
B*07:04
(~0.013% globally)
0
0.01
50
40
30
20
10
0
Presented SARS−CoV−2 peptides (%)
8.9%
2%
B*07:04 Haplotypes (n=25)
0.06
Global haplotype frequency (%)

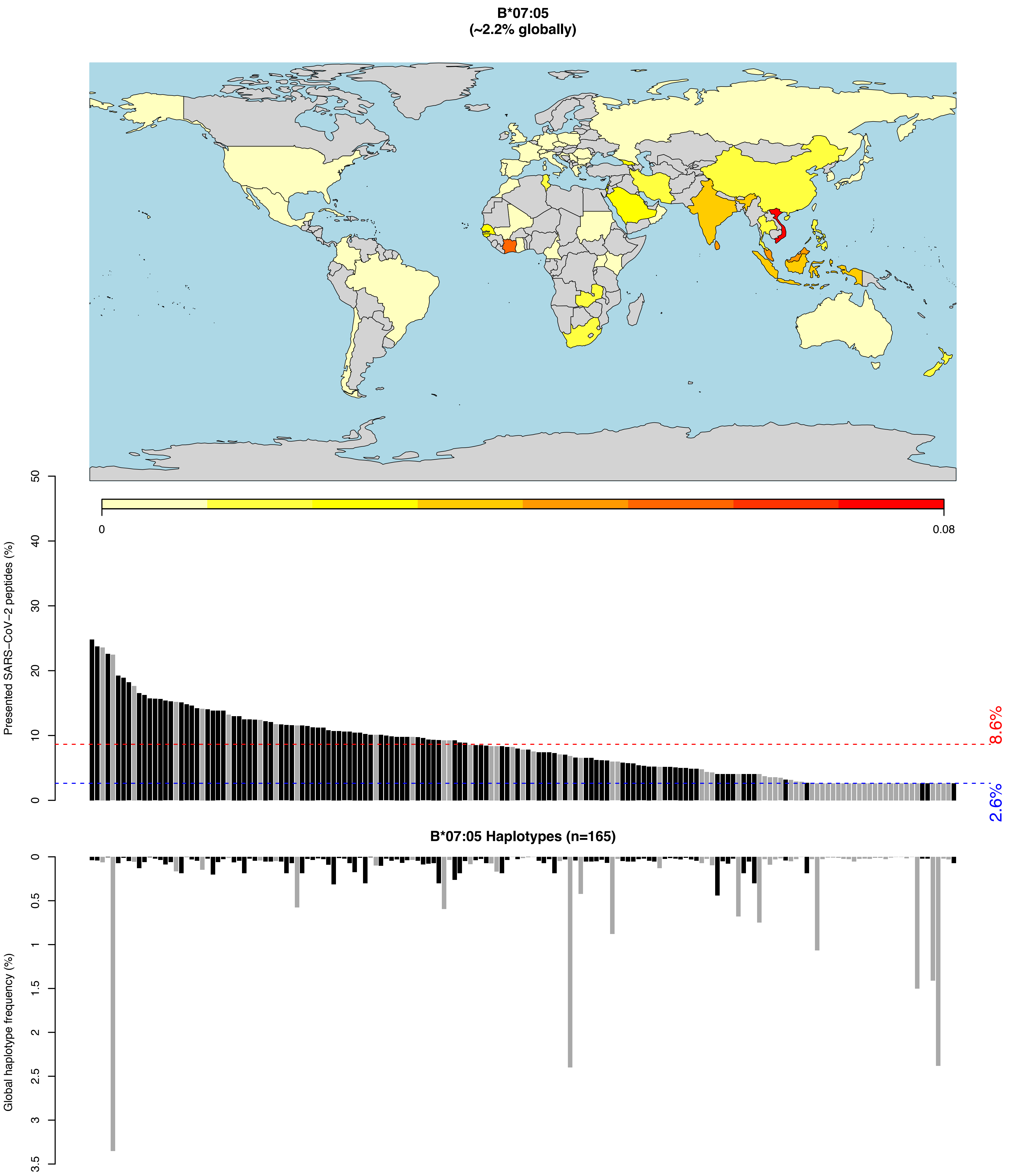

B*07:05
(~2.2% globally)
0
0.08
Presented SARS−CoV−2 peptides (%)
50
40
30
20
10
0
8.6%
2.6%
B*07:05 Haplotypes (n=165)
Global haplotype frequency (%)
0
0.5
1
1.5
2
2.5
3
3.5

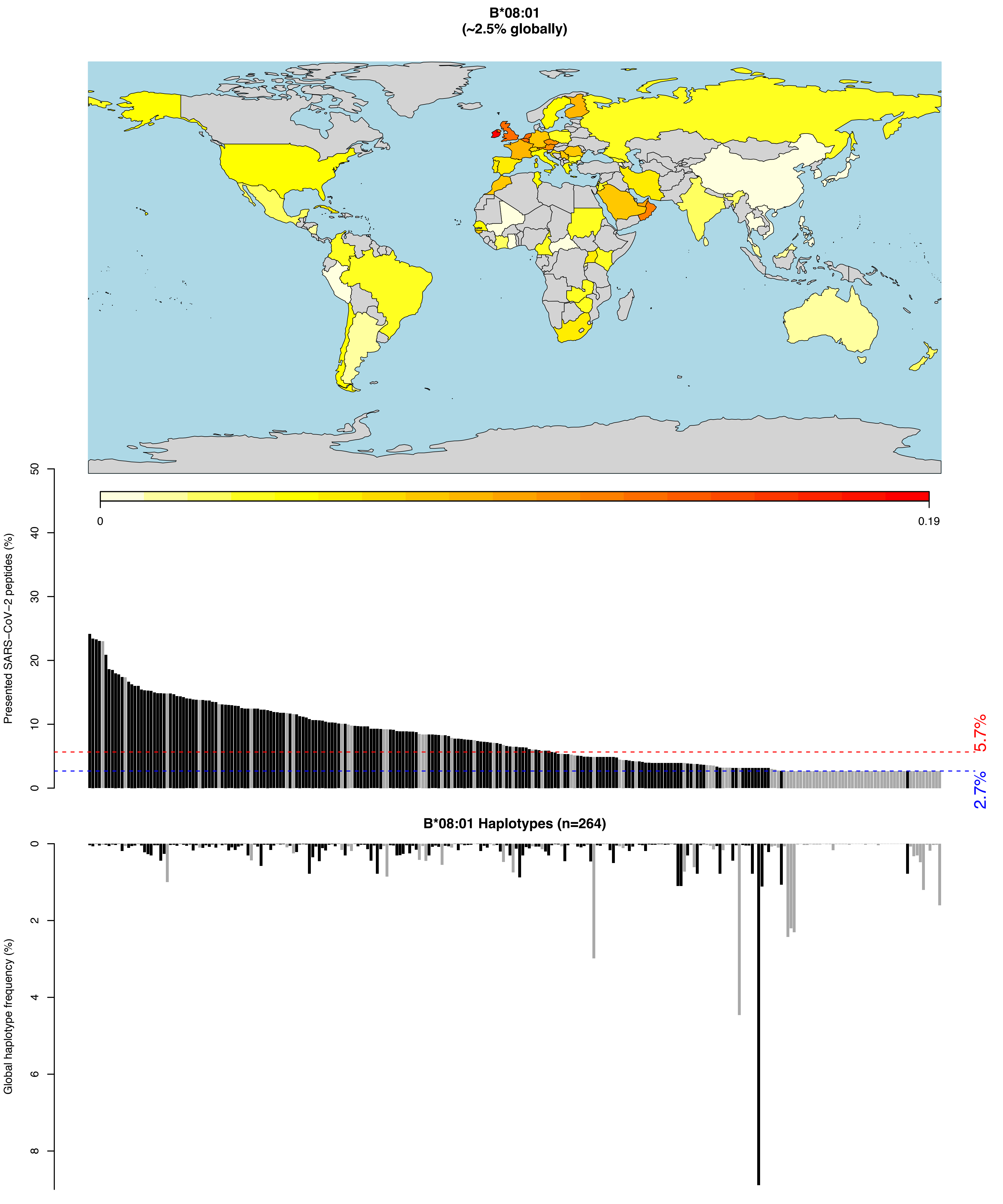

B*08:01
(~2.5% globally)
0
0.19
Presented SARS−CoV−2 peptides (%)
0
10
20
30
40
50
5.7%
2.7%
B*08:01 Haplotypes (n=264)
Global haplotype frequency (%)
0
2
4
6
8

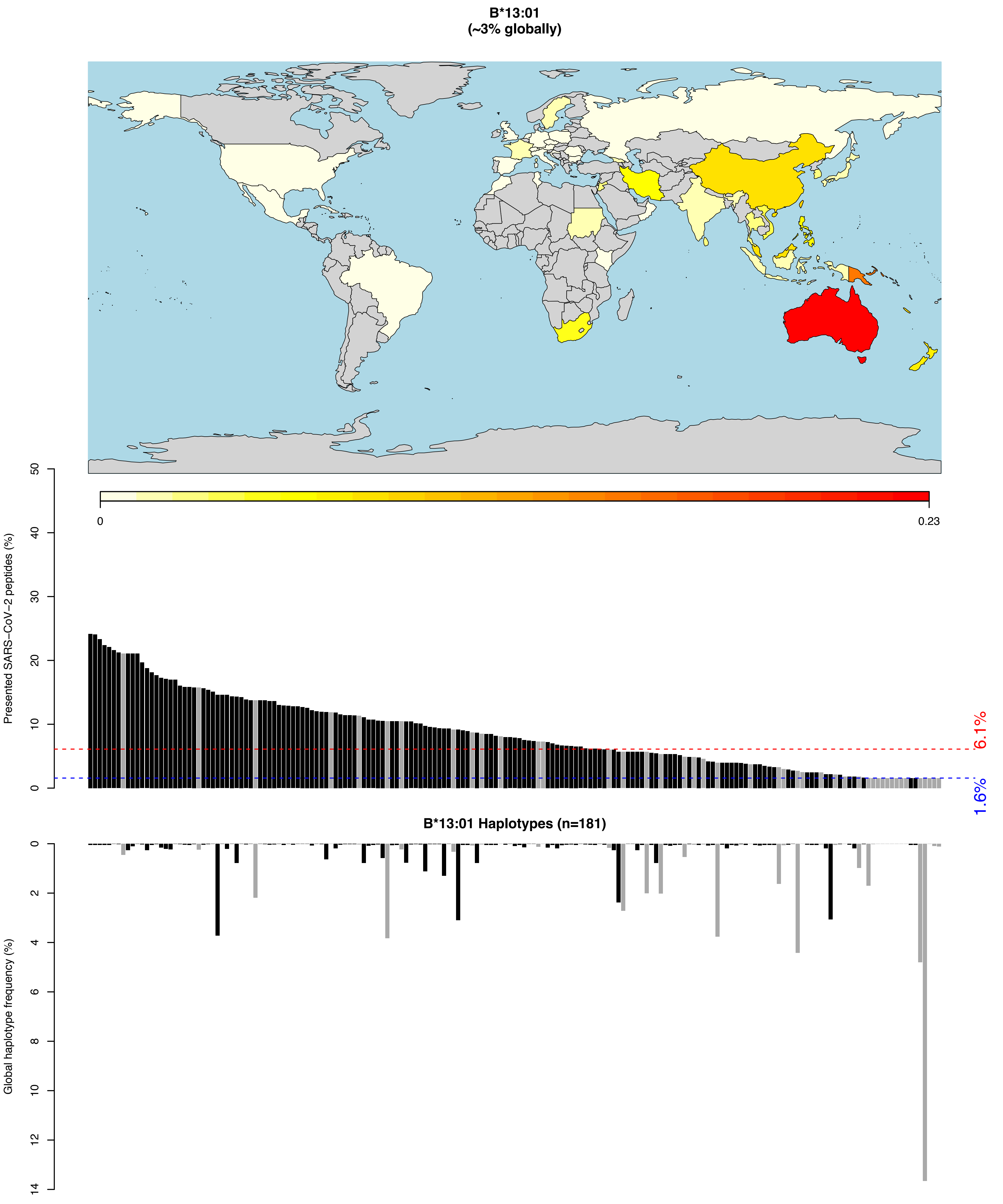
B*13:01
(~3% globally)
0
0.23
Presented SARS−CoV−2 peptides (%)
50
40
30
20
10
0
6.1%
1.6%
B*13:01 Haplotypes (n=181)
Global haplotype frequency (%)
0
2
4
6
8
10
12
14

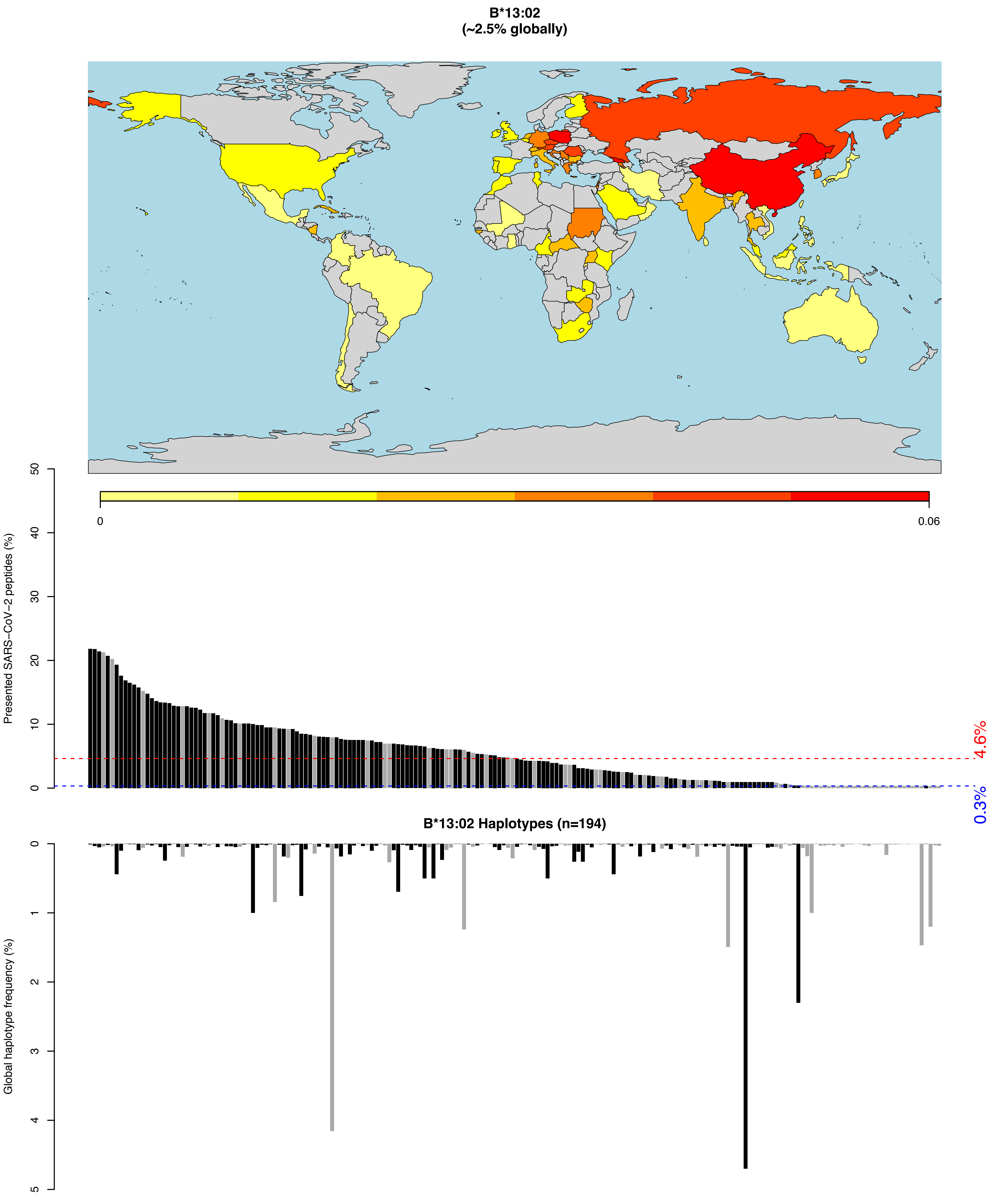

B*13:02
(~2.5% globally)
0
0.06
Presented SARS−CoV−2 peptides (%)
50
40
30
20
10
0
4.6%
0.3%
B*13:02 Haplotypes (n=194)
Global haplotype frequency (%)
0
1
2
3
4
5

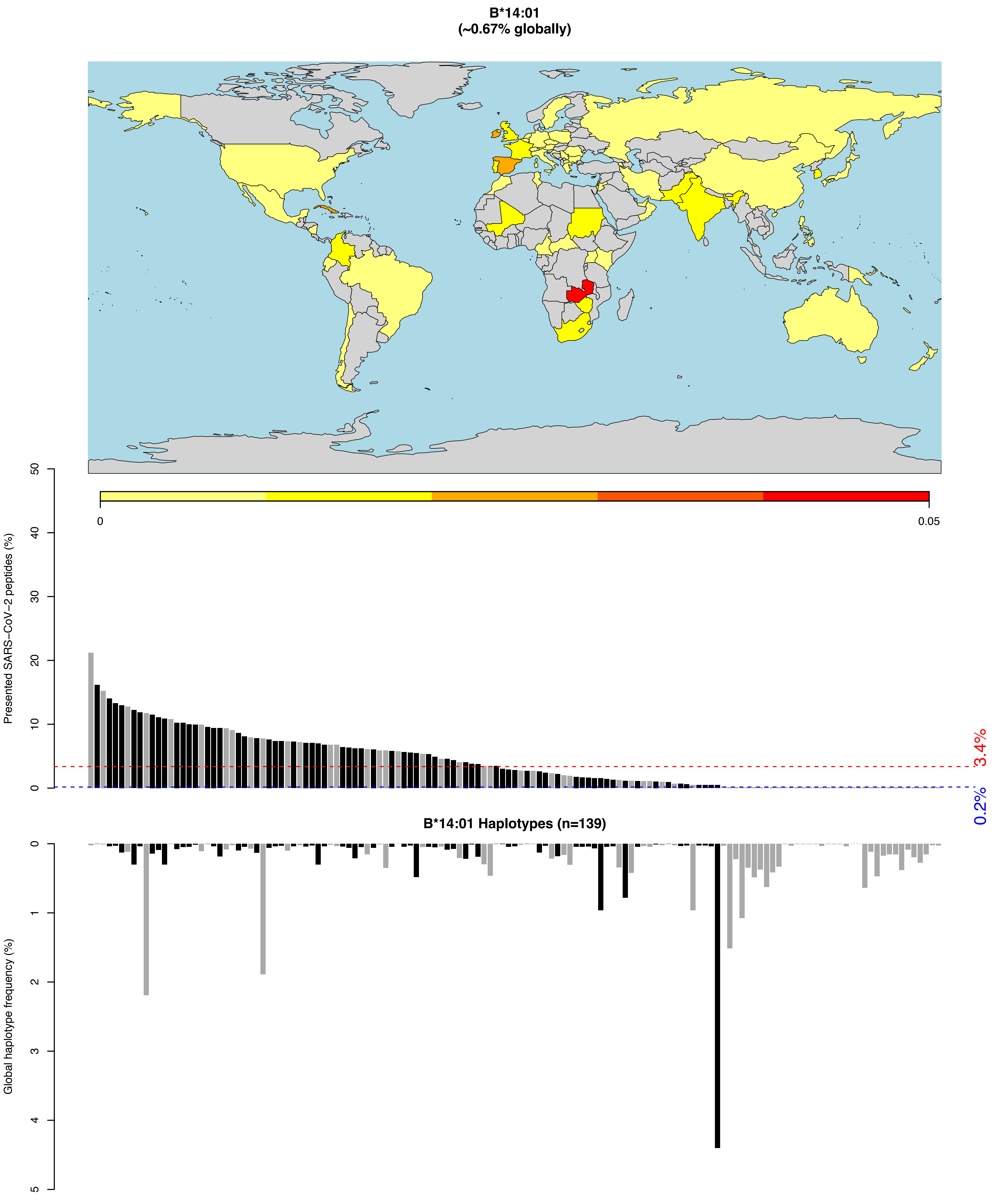

B*14:01
(~0.67% globally)
0
0.05
50
40
30
20
10
0
Presented SARS−CoV−2 peptides (%)
3.4%
0.2%
B*14:01 Haplotypes (n=139)
0
1
2
3
4
5
Global haplotype frequency (%)

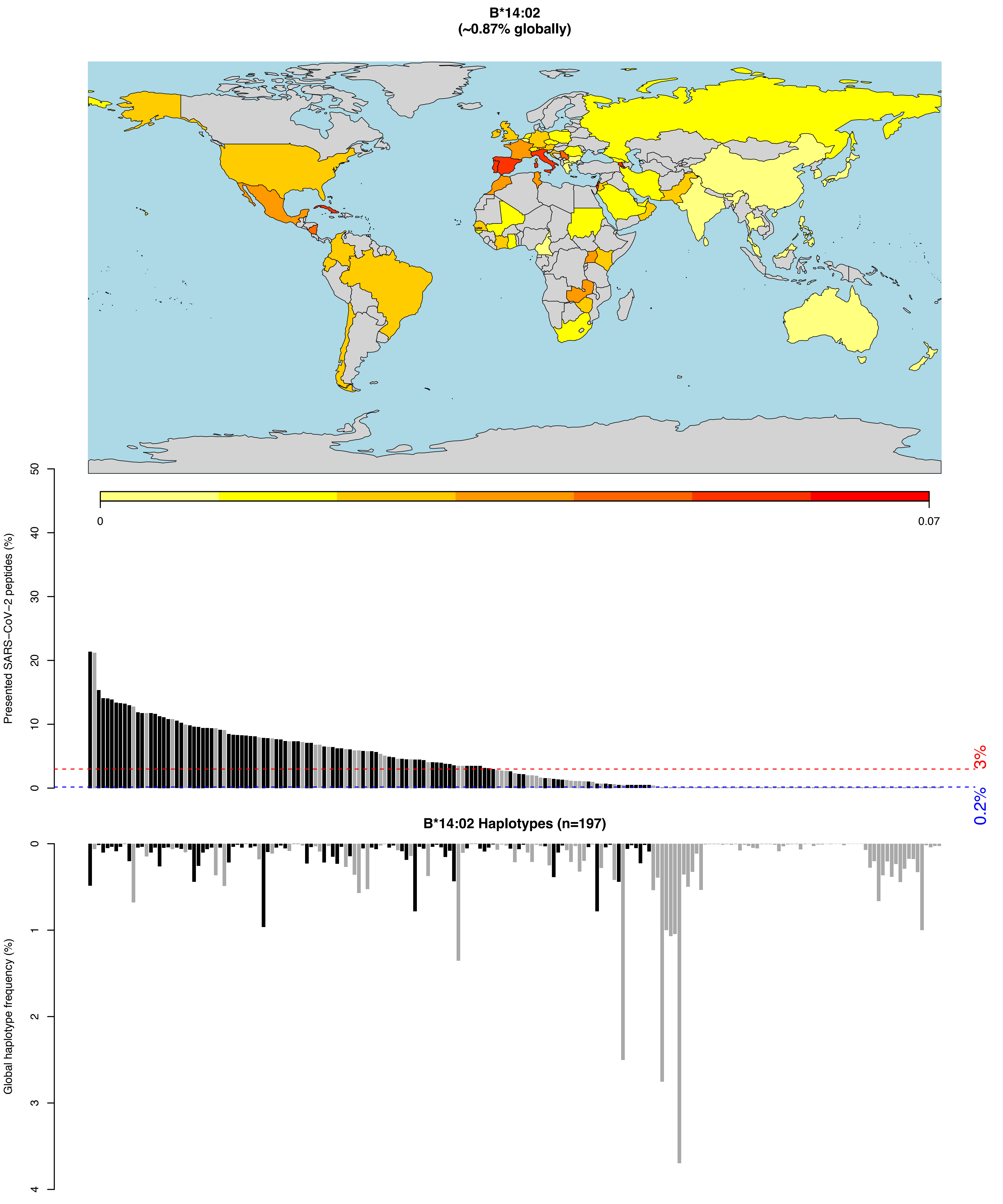

B*14:02
(~0.87% globally)
0
0.07
Presented SARS−CoV−2 peptides (%)
50
40
30
20
10
0
3%
0.2%
B*14:02 Haplotypes (n=197)
Global haplotype frequency (%)
0
1
2
3
4

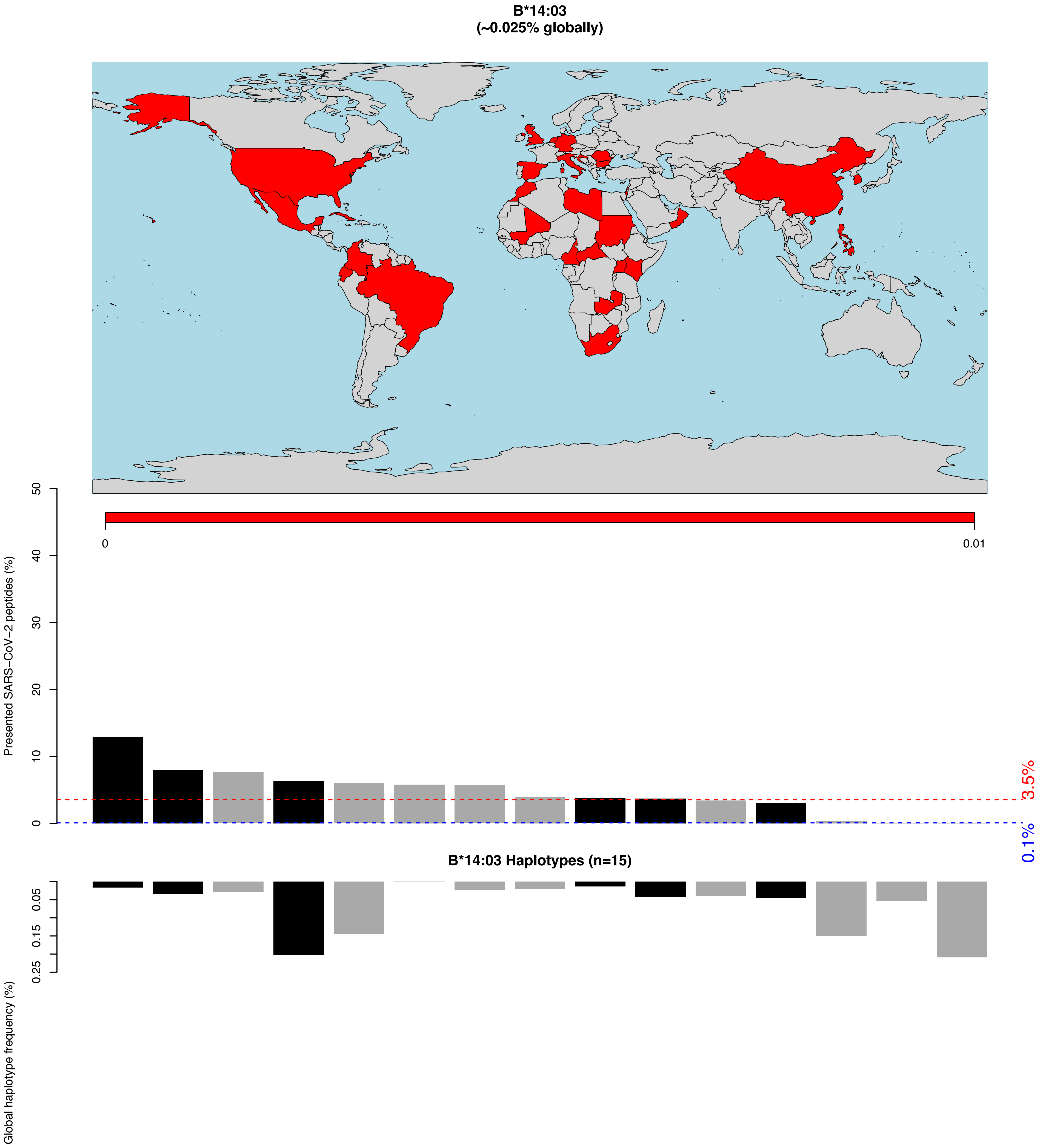

B*14:03
(~0.025% globally)
0
0.01
Presented SARS−CoV−2 peptides (%)
50
40
30
20
10
0
3.5%
0.1%
B*14:03 Haplotypes (n=15)
Global haplotype frequency (%)
0.05
0.15
0.25

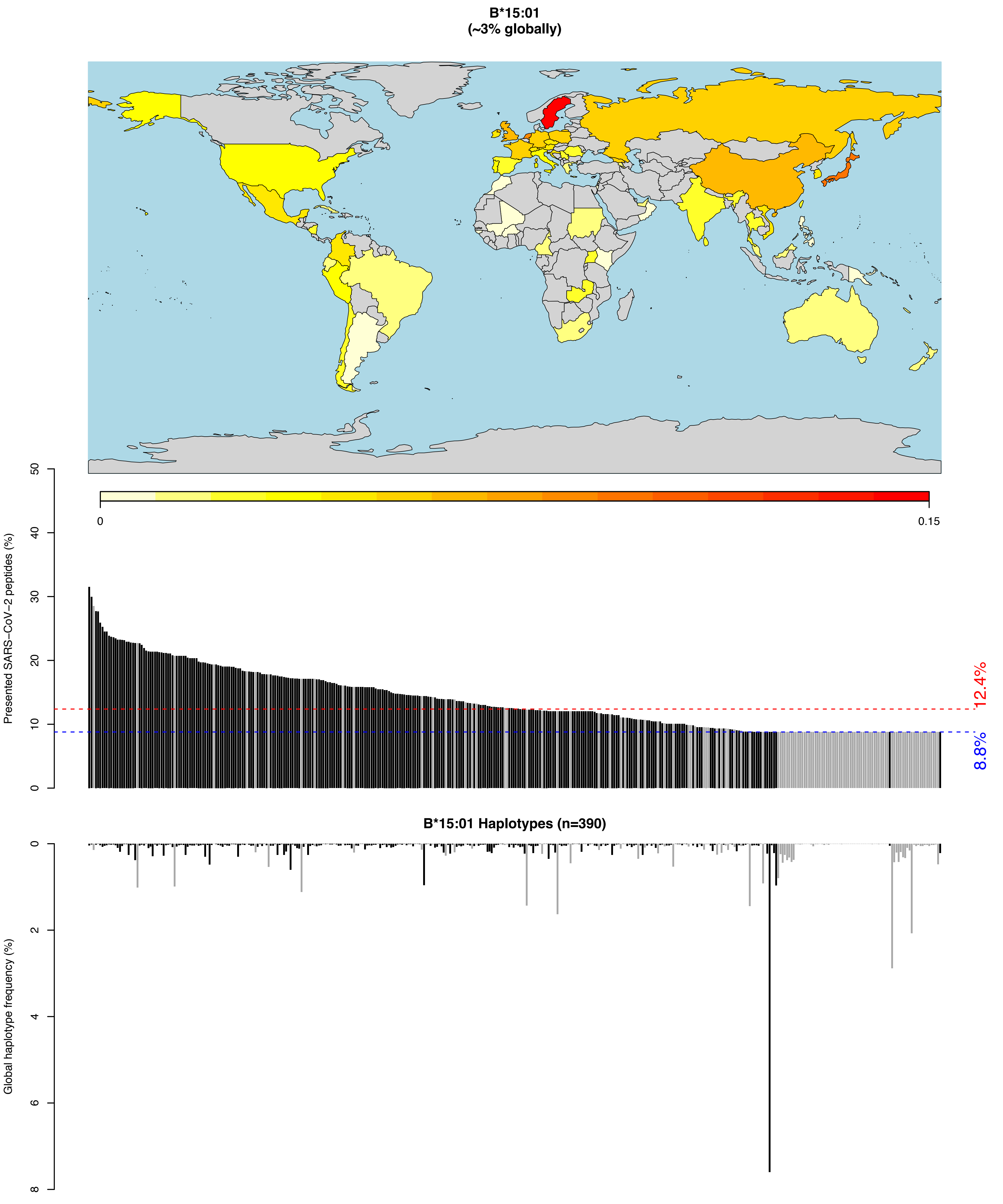

B*15:01
(~3% globally)
0
0.15
Presented SARS−CoV−2 peptides (%)
50
40
30
20
10
0
12.4%
8.8%
B*15:01 Haplotypes (n=390)
Global haplotype frequency (%)
0
2
4
6
8

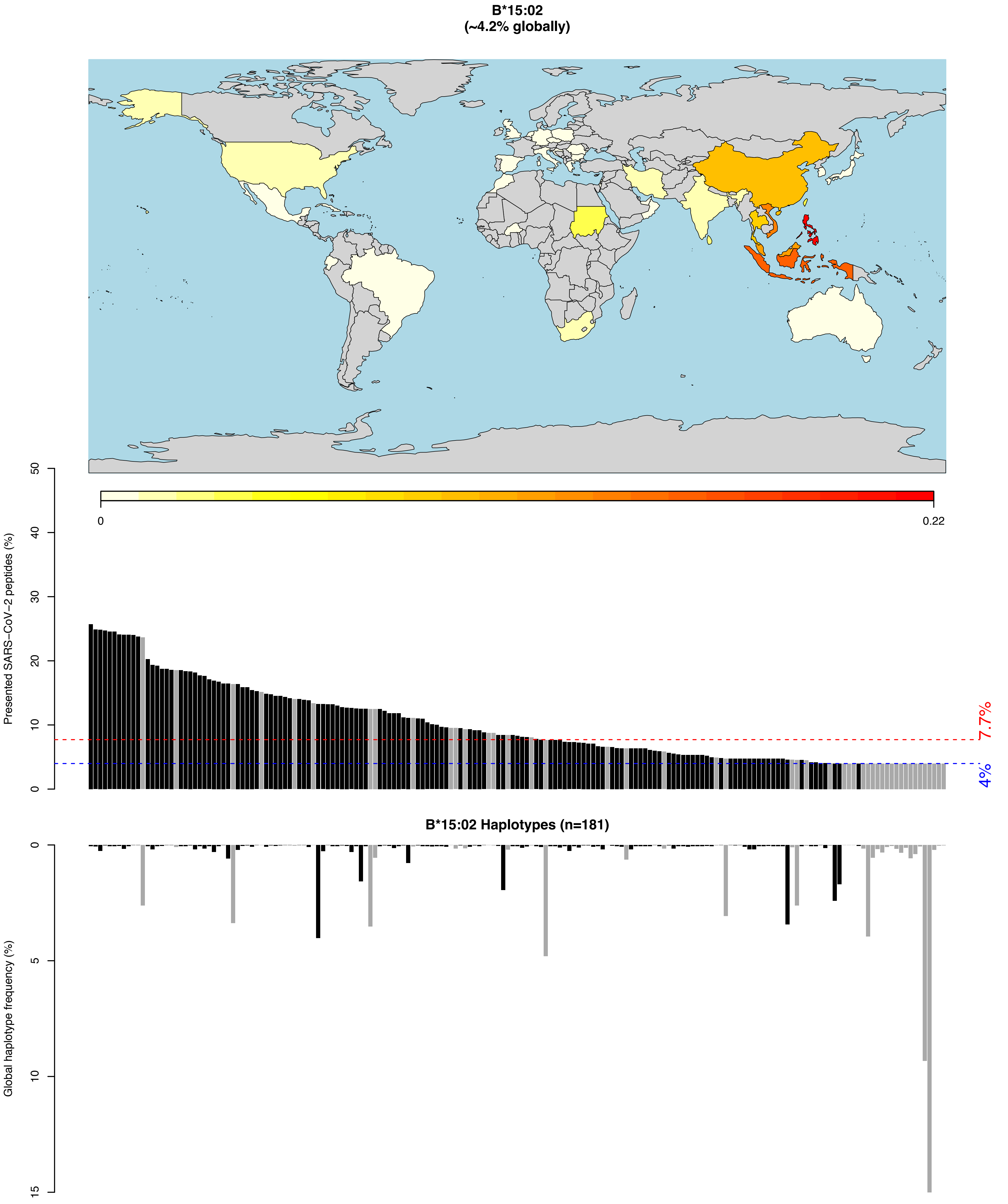

B*15:02
(~4.2% globally)
0
0.22
Presented SARS−CoV−2 peptides (%)
50
40
30
20
10
0
7.7%
4%
B*15:02 Haplotypes (n=181)
Global haplotype frequency (%)
0
5
10
15

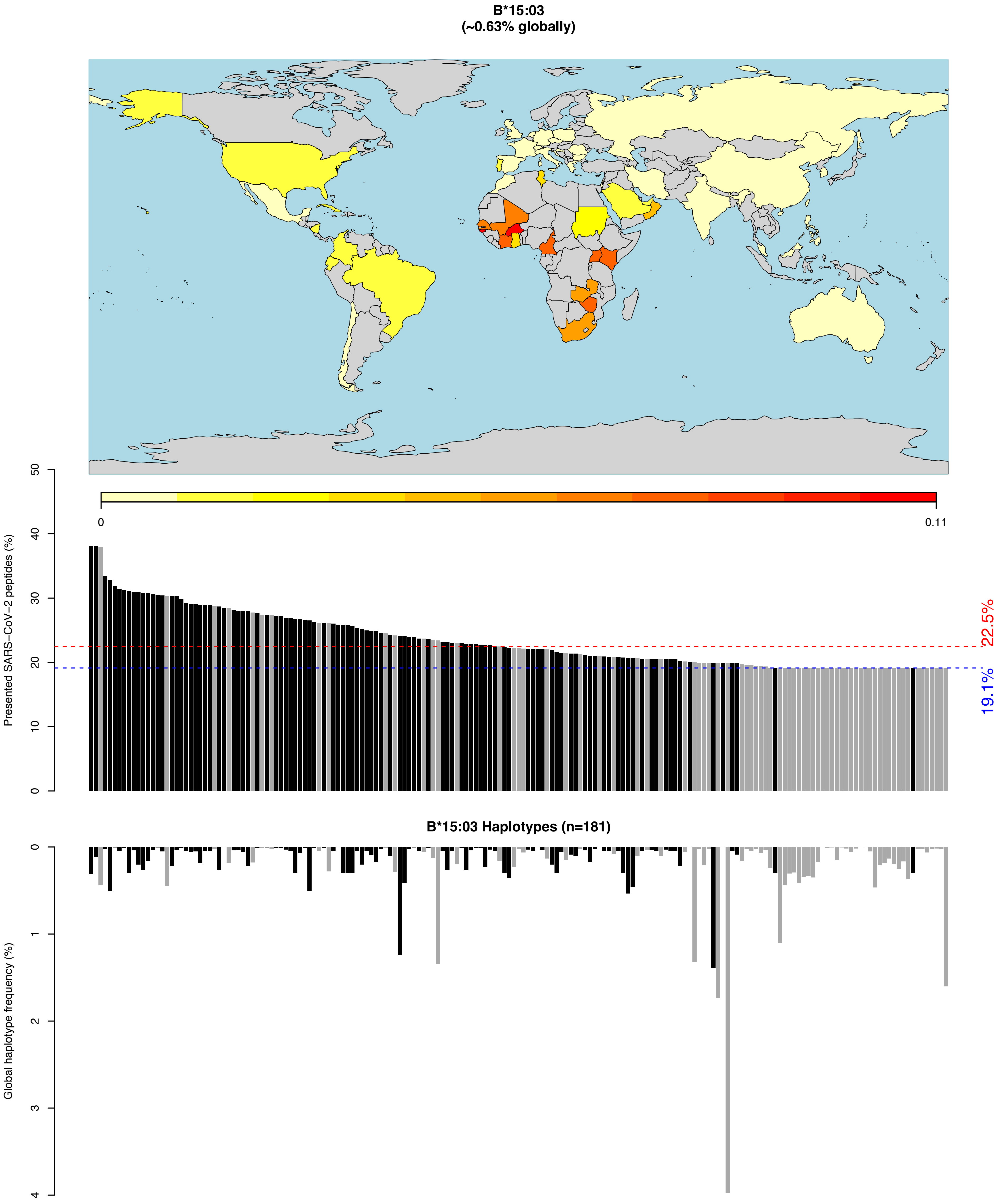
B*15:03
(~0.63% globally)
0
0.11
Presented SARS−CoV−2 peptides (%)
0
10
20
30
40
50
22.5%
19.1%
B*15:03 Haplotypes (n=181)
Global haplotype frequency (%)
0
1
2
3
4

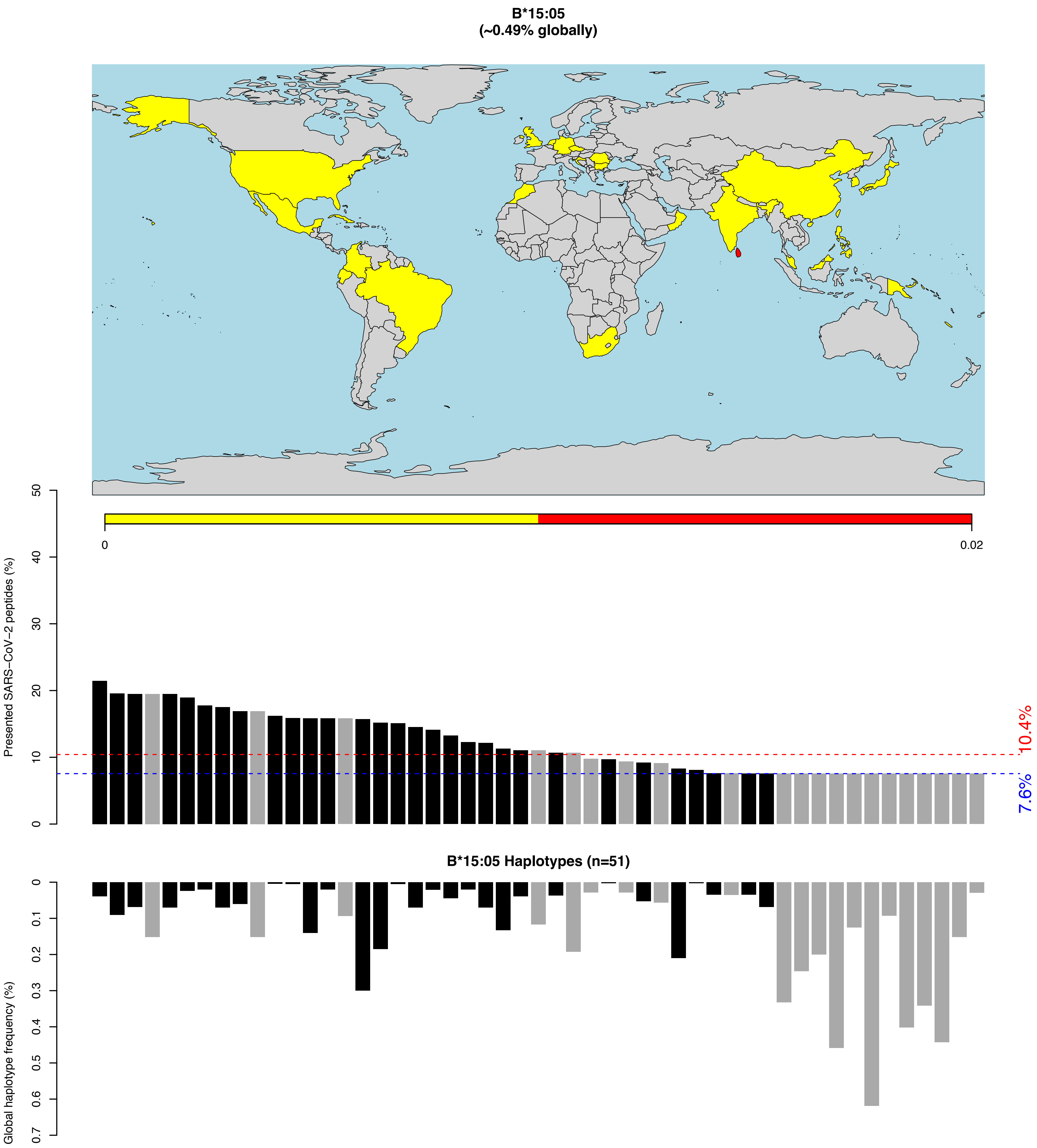

B*15:05
(~0.49% globally)
0
0.02
Presented SARS−CoV−2 peptides (%)
50
40
30
20
10
0
10.4%
7.6%
B*15:05 Haplotypes (n=51)
Global haplotype frequency (%)
0
0.1
0.2
0.3
0.4
0.5
0.6
0.7

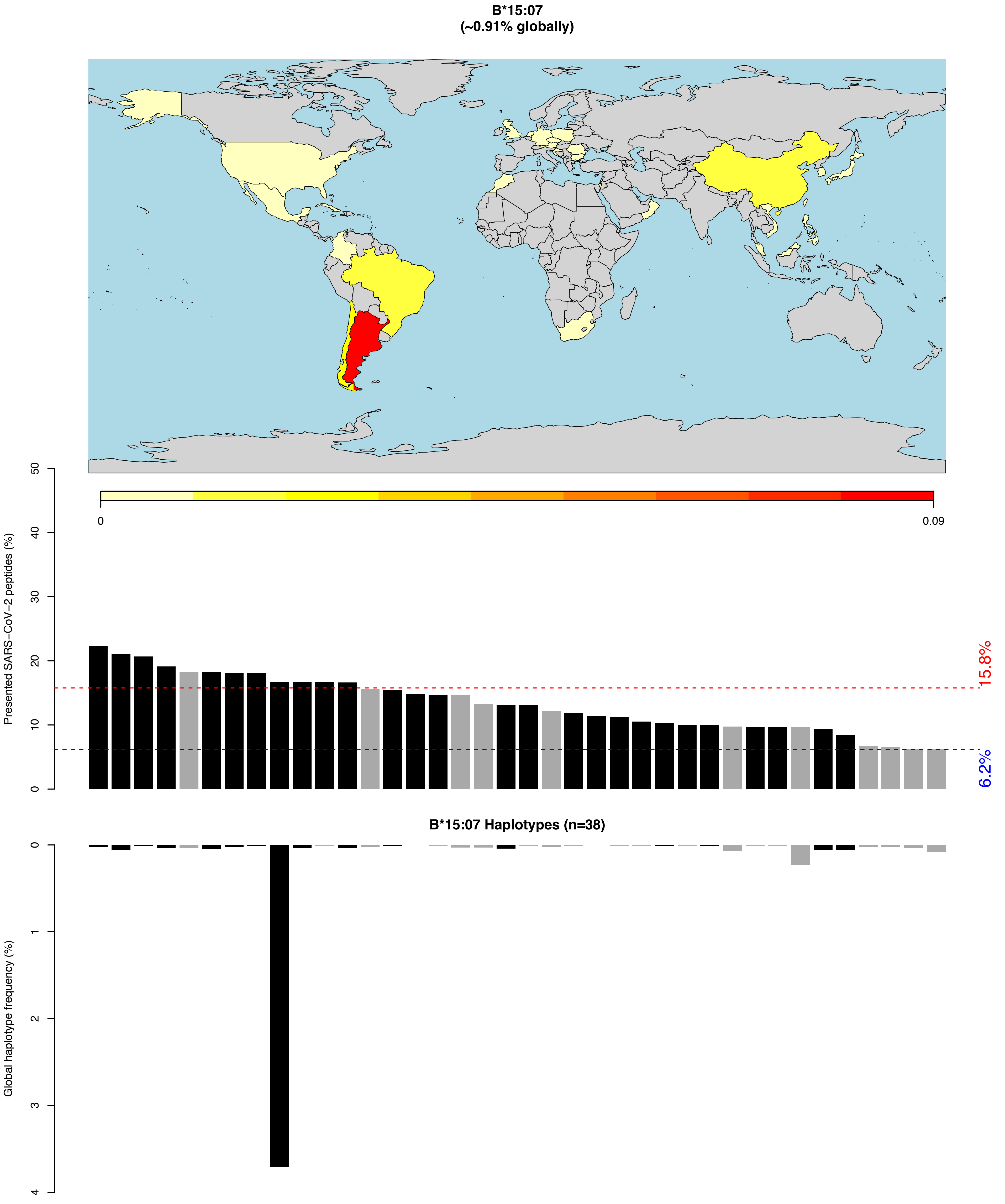

B*15:07
(~0.91% globally)
0
0.09
Presented SARS−CoV−2 peptides (%)
50
40
30
20
10
0
15.8%
6.2%
B*15:07 Haplotypes (n=38)
Global haplotype frequency (%)
0
1
2
3
4

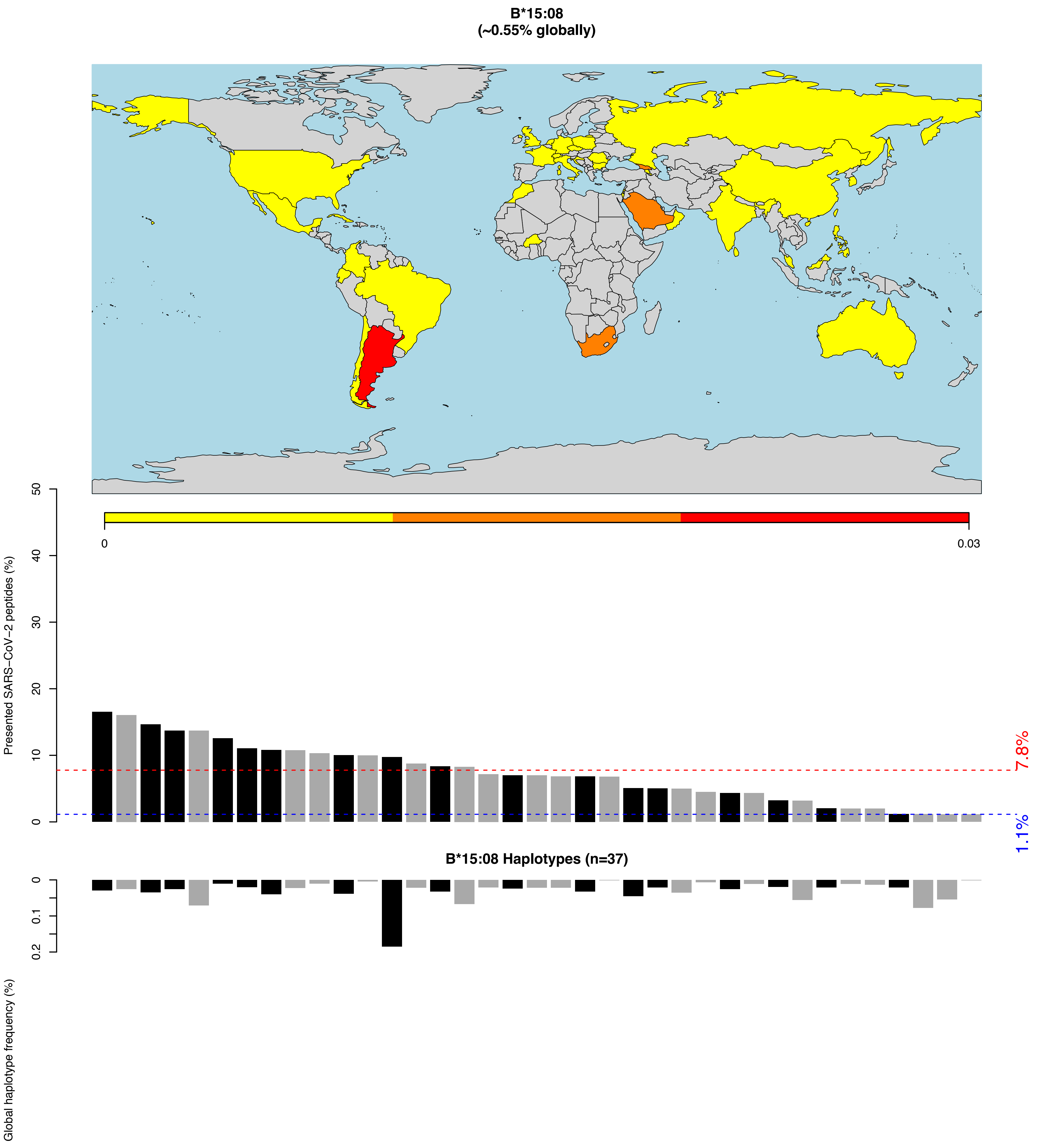
B*15:08
(~0.55% globally)
0
0.03
Presented SARS−CoV−2 peptides (%)
50
40
30
20
10
0
7.8%
1.1%
B*15:08 Haplotypes (n=37)
0
0.1
0.2
Global haplotype frequency (%)

B*15:10
(~0.26% globally)

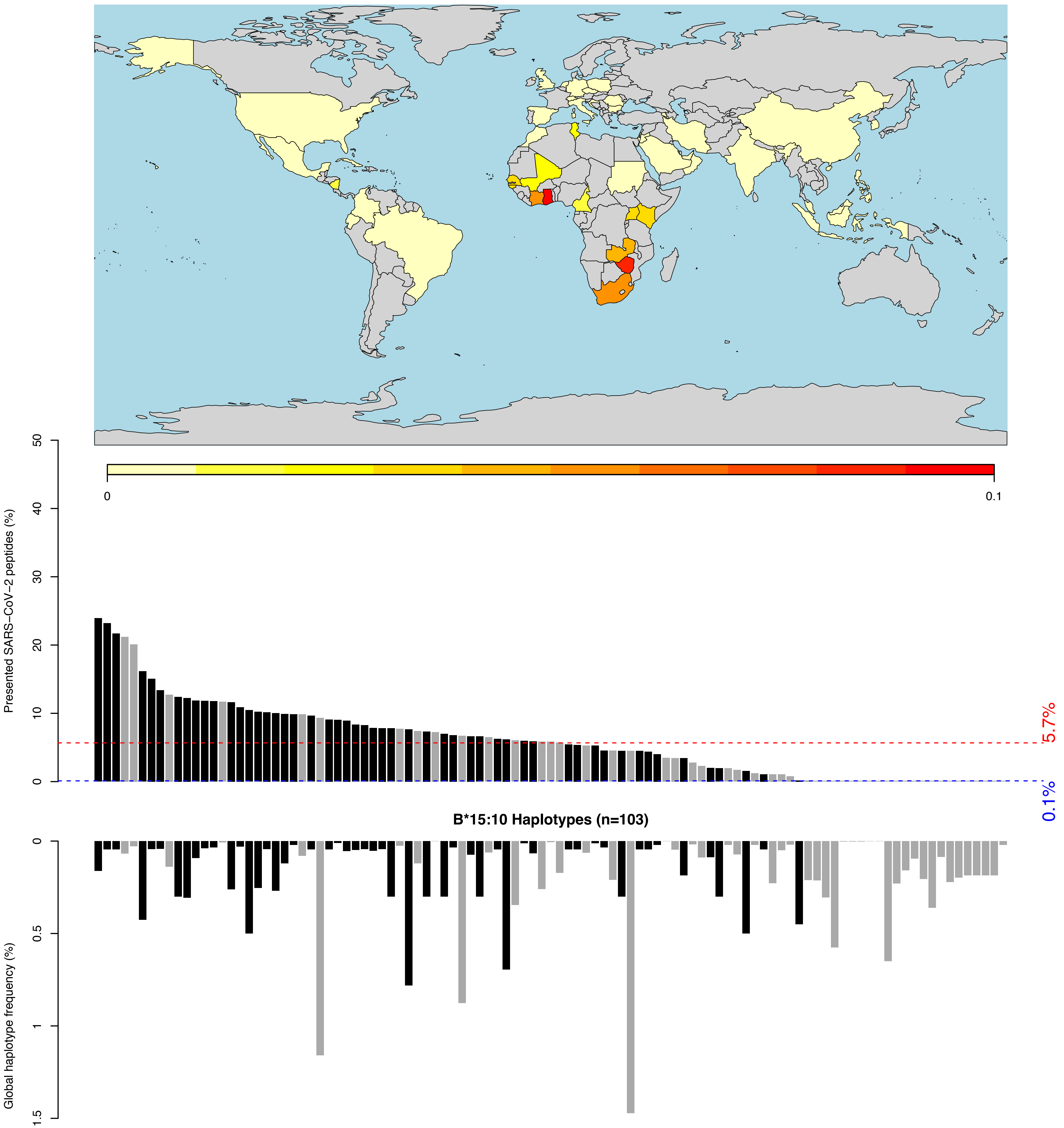

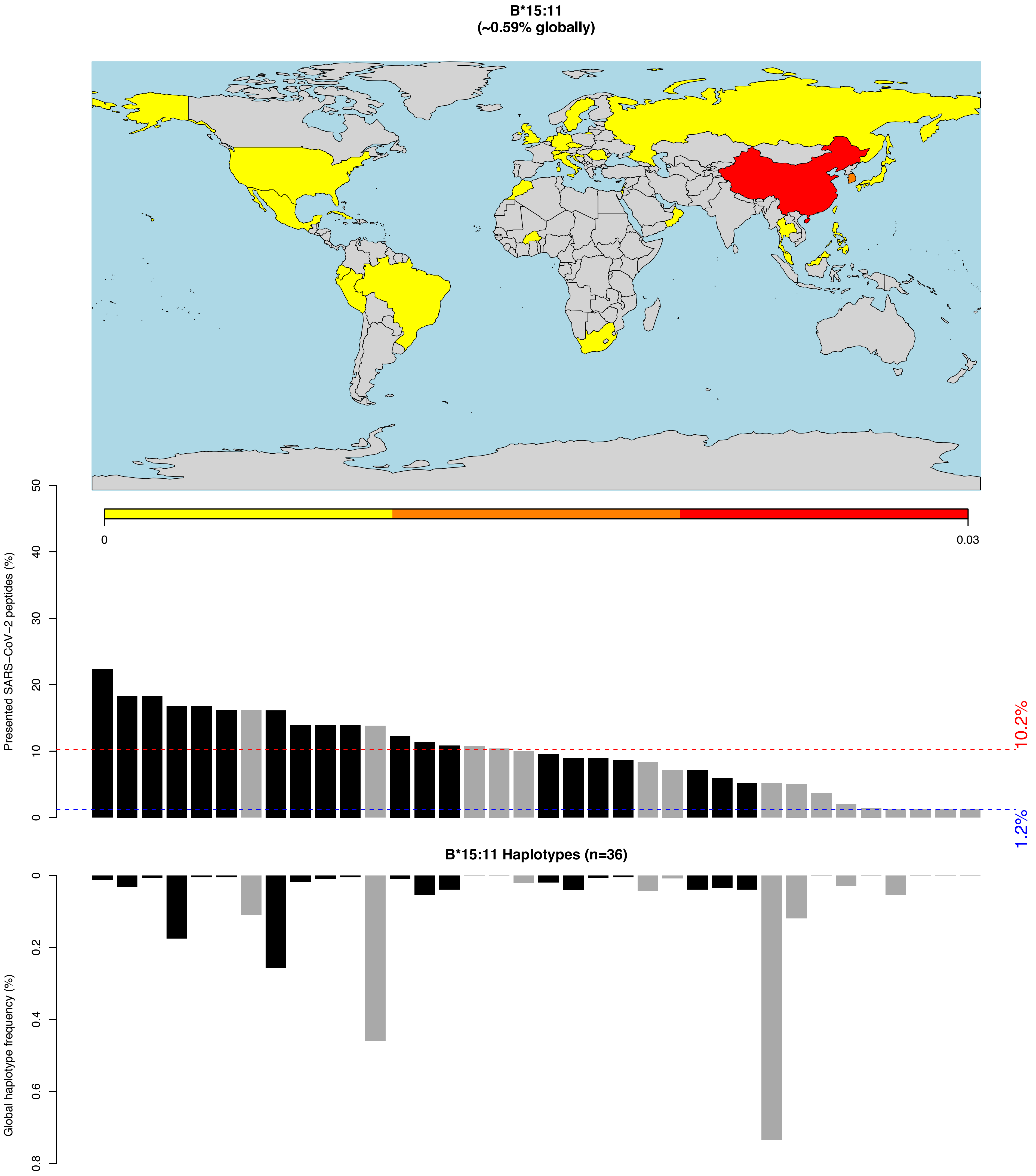

B*15:11
(~0.59% globally)
0
0.03
Presented SARS−CoV−2 peptides (%)
50
40
30
20
10
0
10.2%
1.2%
B*15:11 Haplotypes (n=36)
Global haplotype frequency (%)
0
0.2
0.4
0.6
0.8

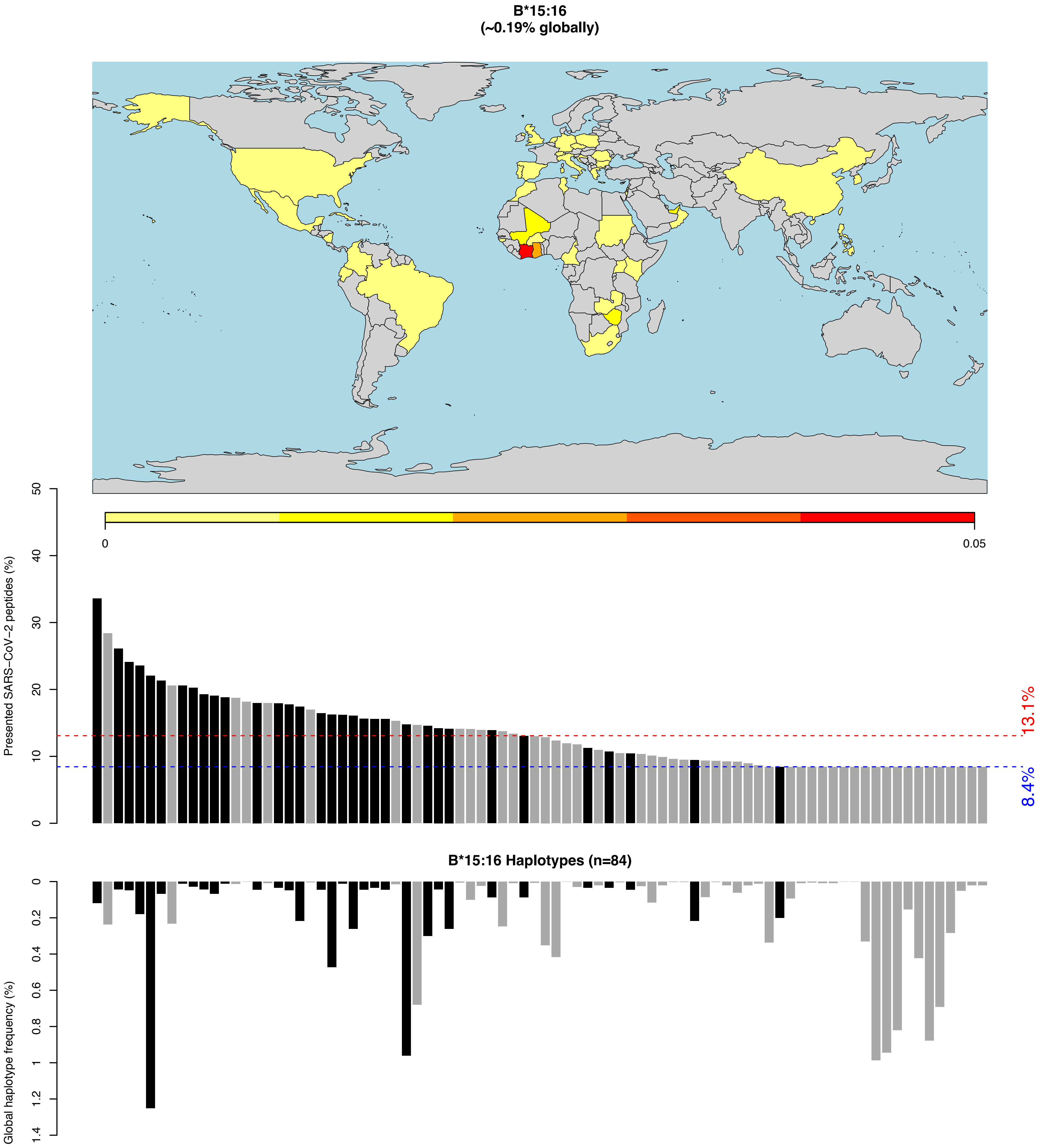

B*15:16
(~0.19% globally)
0
0.05
Presented SARS−CoV−2 peptides (%)
13.1%
8.4%
B*15:16 Haplotypes (n=84)
Global haplotype frequency (%)

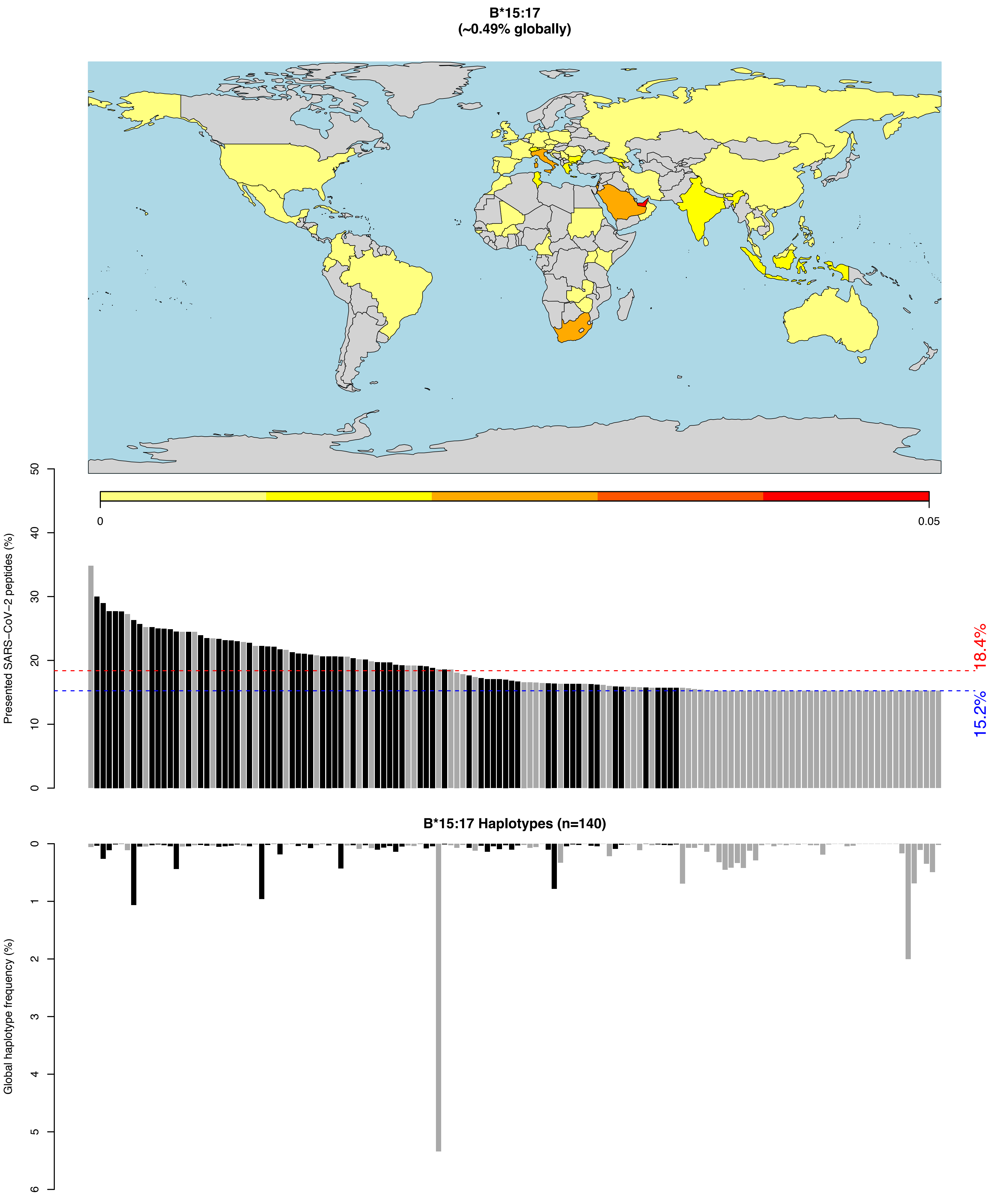
B*15:17
(~0.49% globally)
0
0.05
Presented SARS−CoV−2 peptides (%)
50
40
30
20
10
0
18.4%
15.2%
B*15:17 Haplotypes (n=140)
Global haplotype frequency (%)
0
1
2
3
4
5
6

B*15:18
(~1.3% globally)

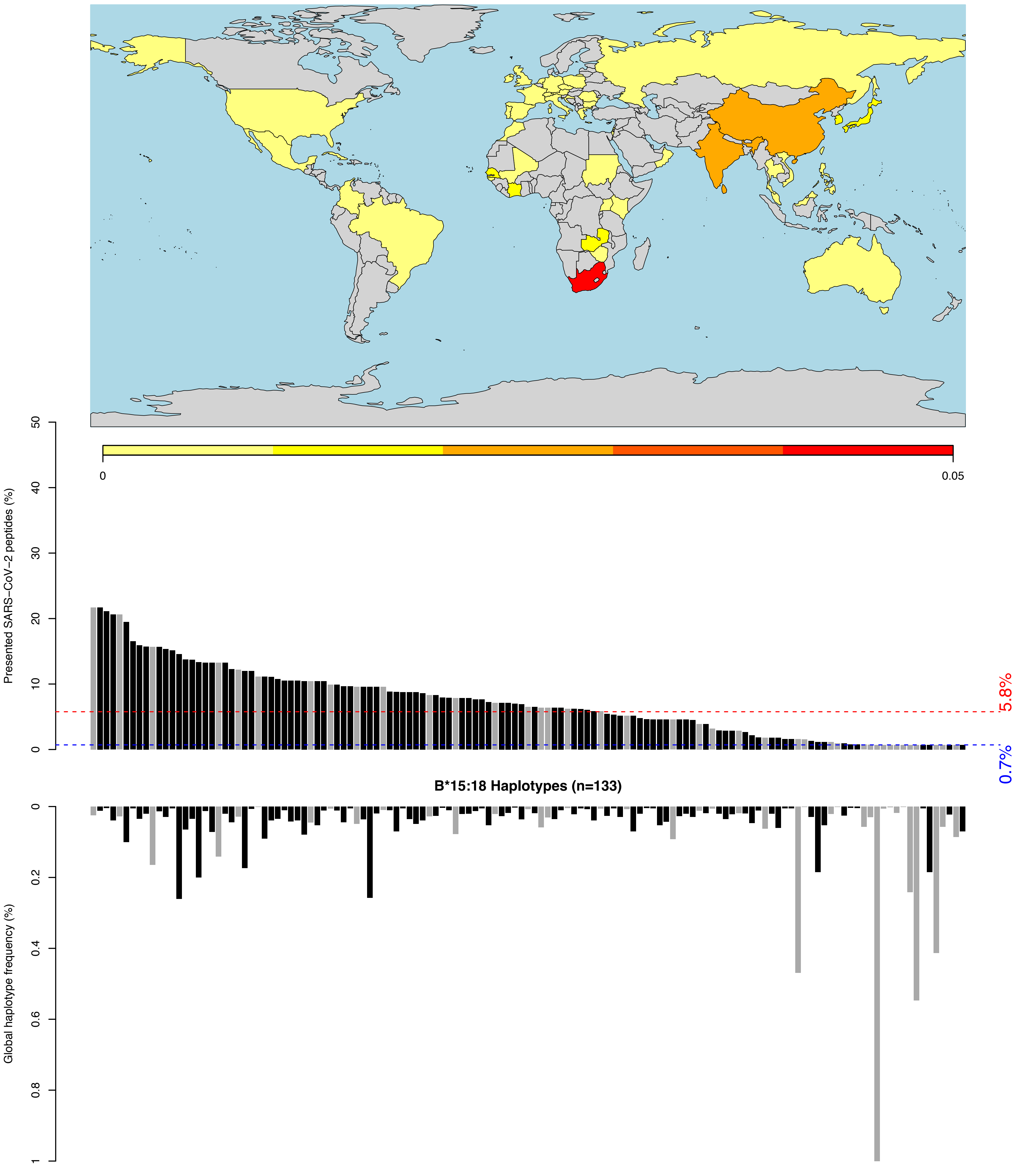

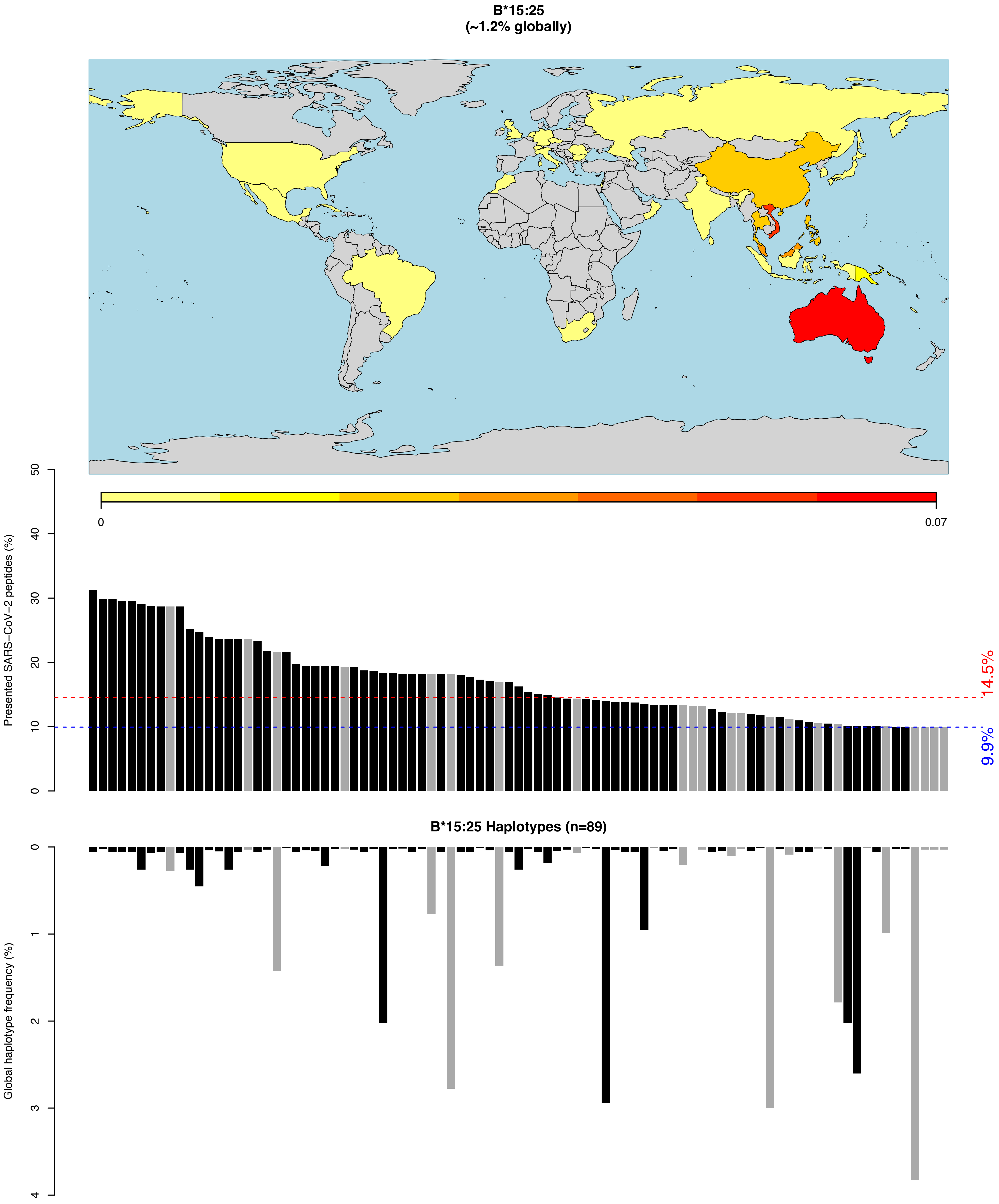

B*15:25
(~1.2% globally)
0
0.07
Presented SARS−CoV−2 peptides (%)
14.5%
9.9%
B*15:25 Haplotypes (n=89)
Global haplotype frequency (%)

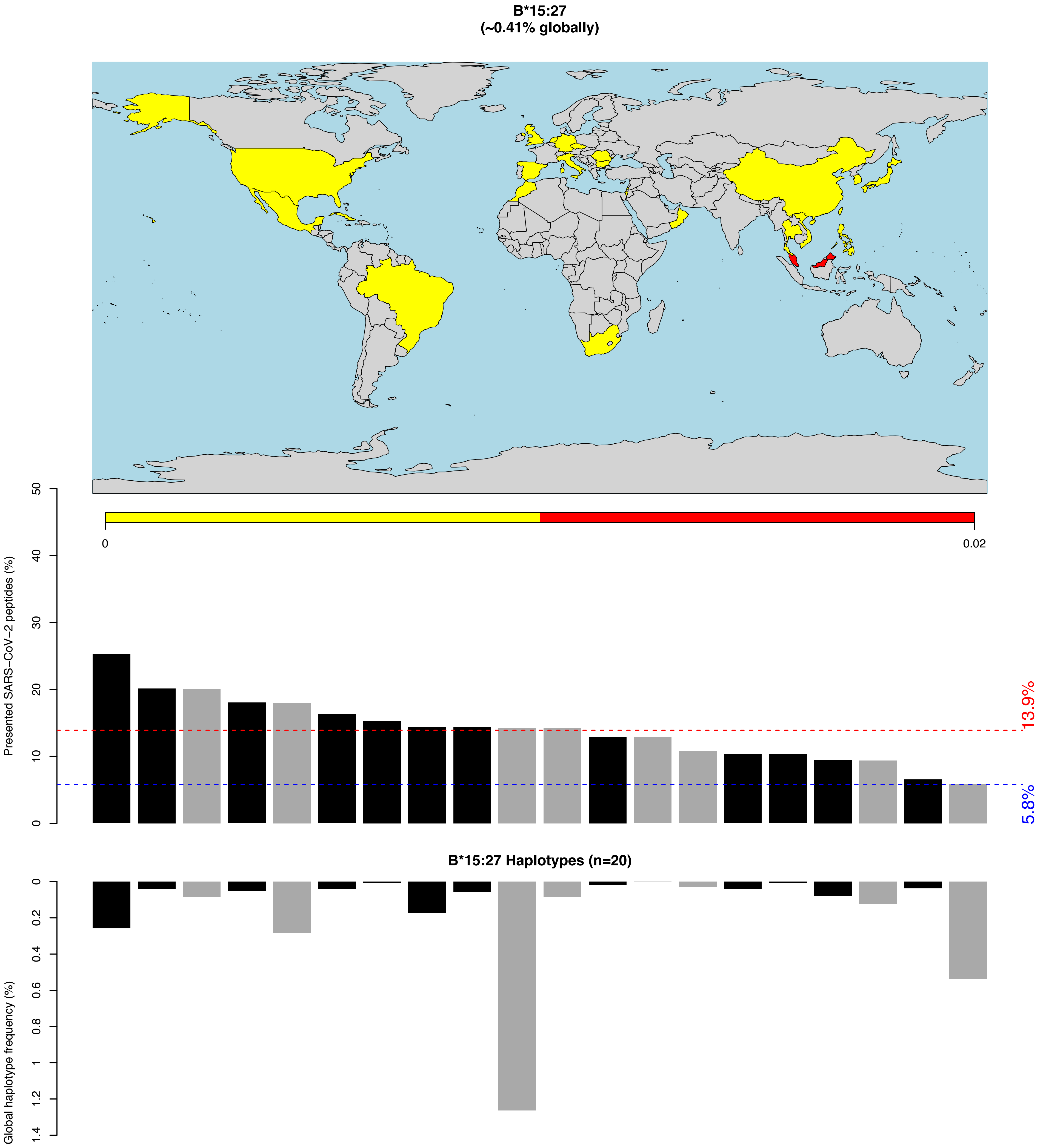
B*15:27
(~0.41% globally)
0
0.02
Presented SARS−CoV−2 peptides (%)
13.9%
5.8%
B*15:27 Haplotypes (n=20)
Global haplotype frequency (%)

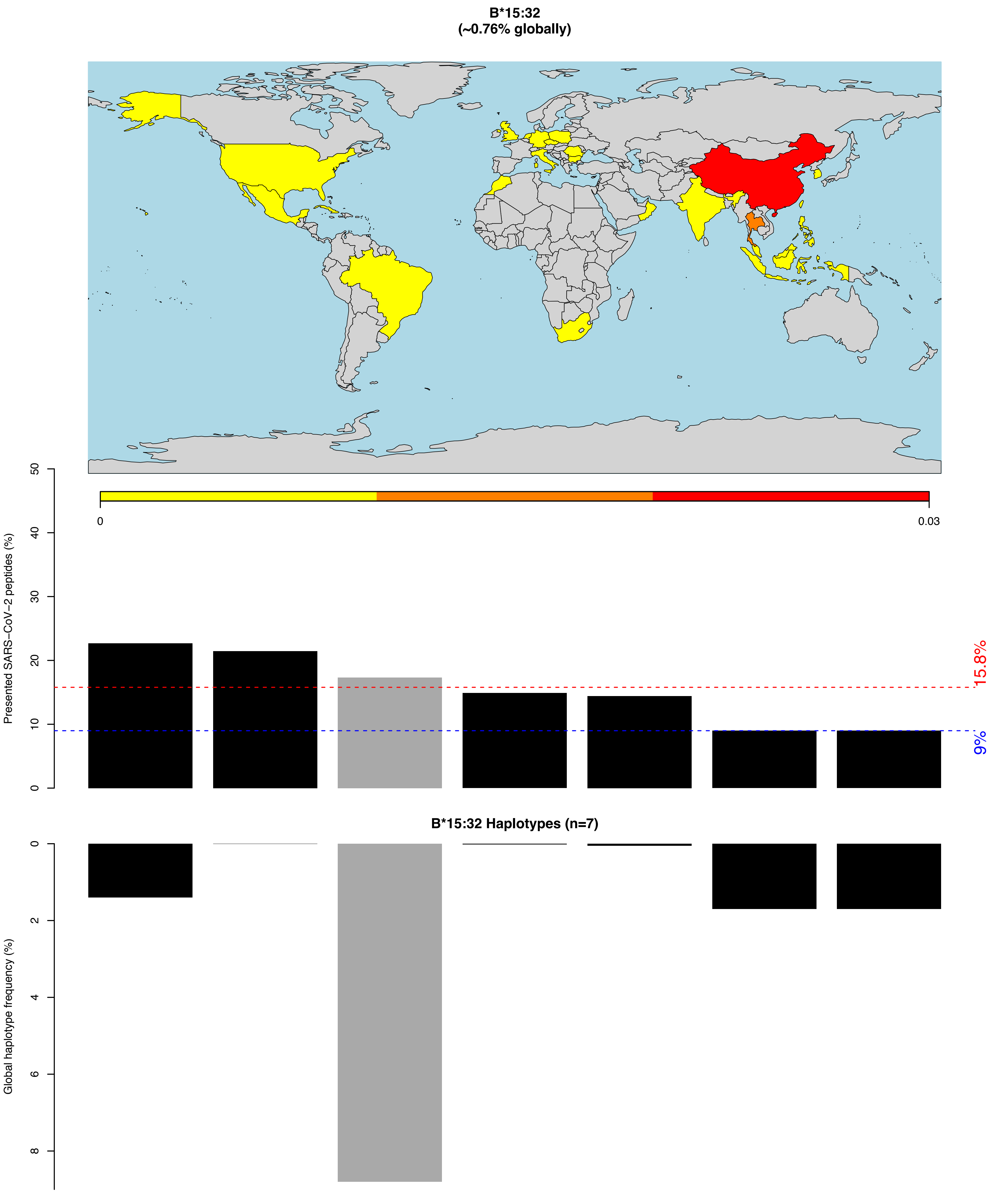
B*15:32
(~0.76% globally)
0
0.03
Presented SARS−CoV−2 peptides (%)
0
10
20
30
40
50
15.8%
9%
B*15:32 Haplotypes (n=7)
Global haplotype frequency (%)
0
2
4
6
8

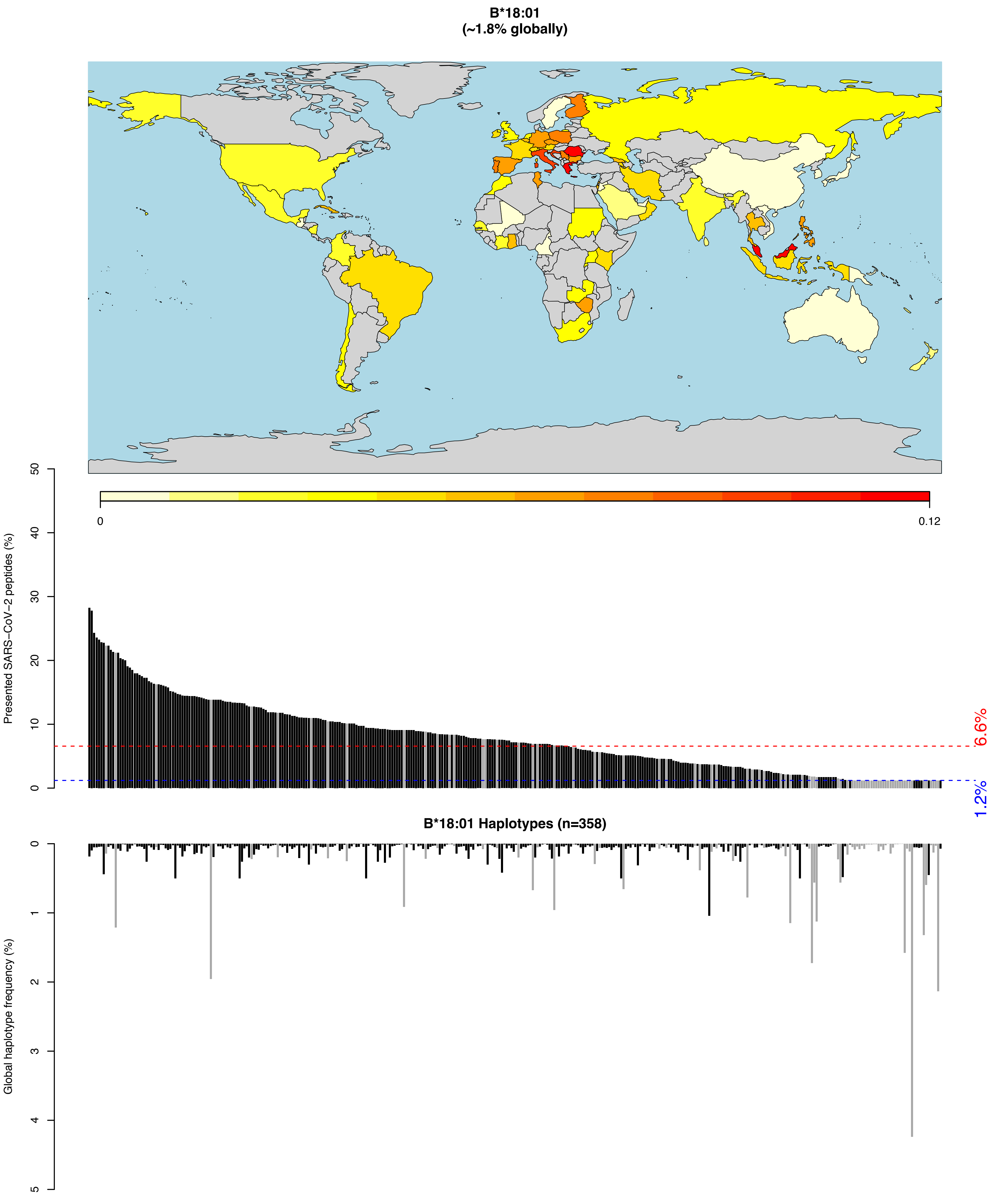

B*18:01
(~1.8% globally)
0
0.12
Presented SARS−CoV−2 peptides (%)
0
10
20
30
40
50
6.6%
1.2%
B*18:01 Haplotypes (n=358)
Global haplotype frequency (%)
0
1
2
3
4
5

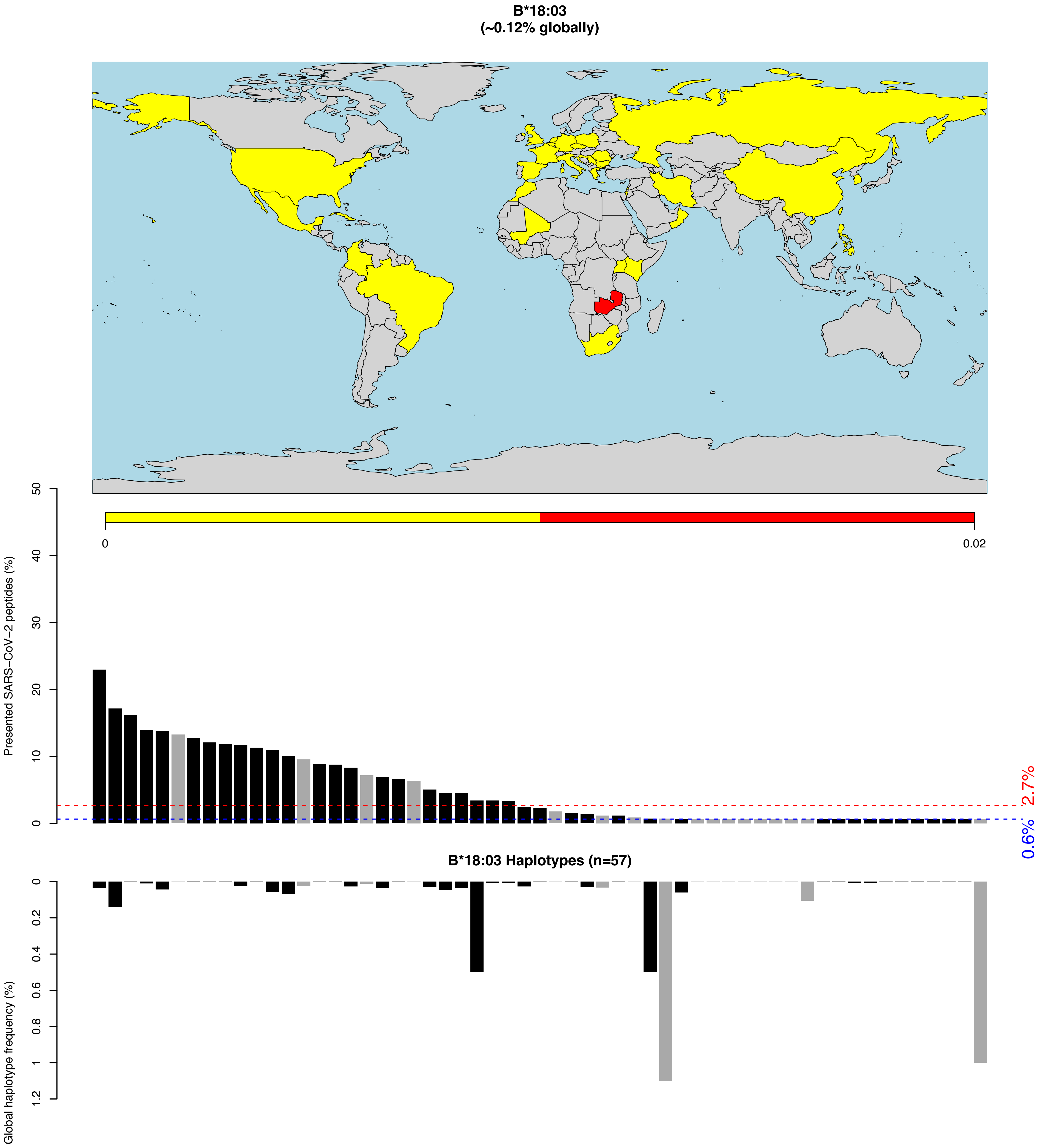

B*18:03
(~0.12% globally)
0
0.02
Presented SARS−CoV−2 peptides (%)
50
40
30
20
10
0
2.7%
0.6%
B*18:03 Haplotypes (n=57)
Global haplotype frequency (%)
0
0.2
0.4
0.6
0.8
1
1.2

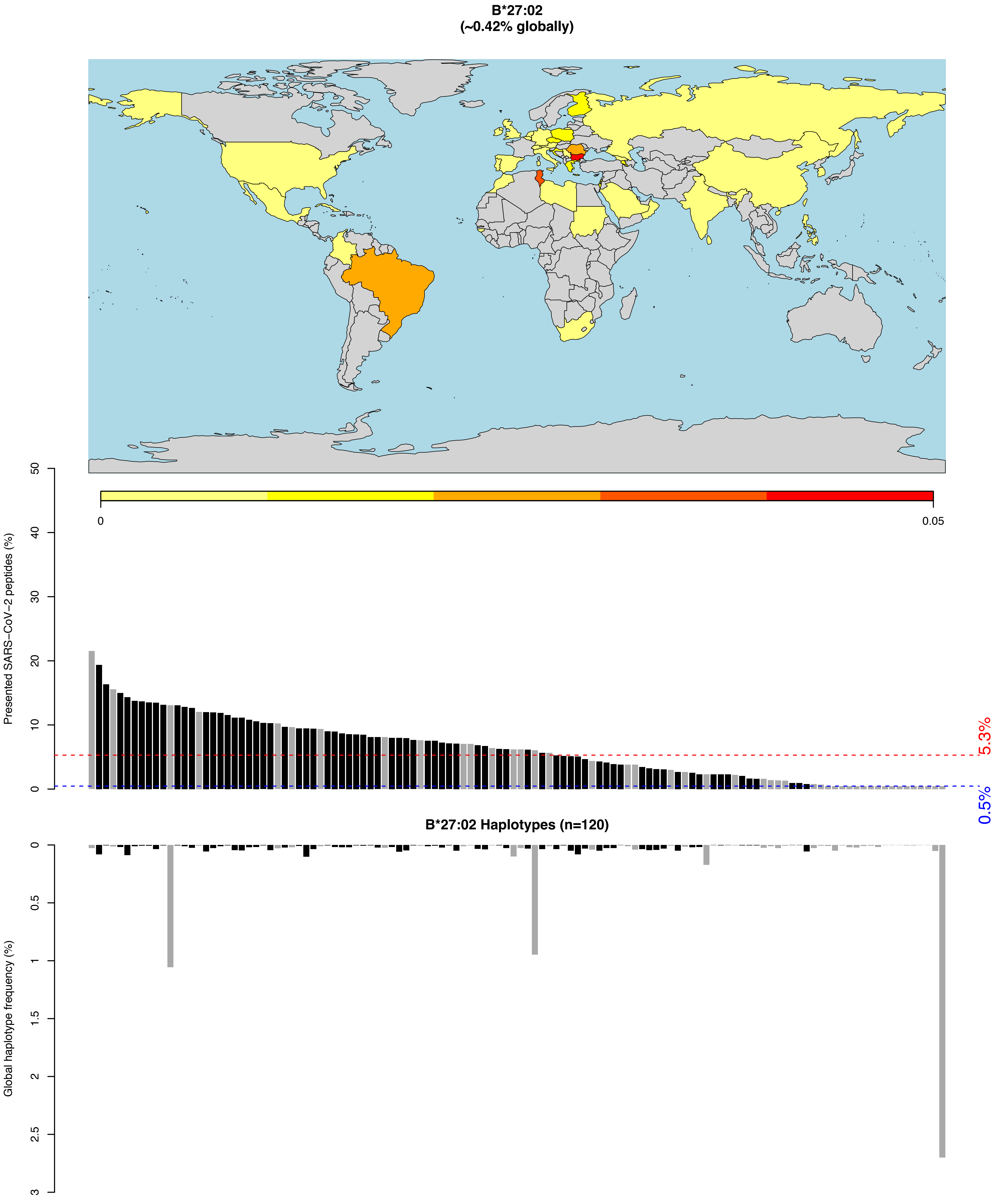

B*27:02
(~0.42% globally)
0
0.05
Presented SARS−CoV−2 peptides (%)
50
40
30
20
10
0
5.3%
0.5%
B*27:02 Haplotypes (n=120)
Global haplotype frequency (%)
0
0.5
1
1.5
2
2.5
3

B*27:03
(~0.49% globally)

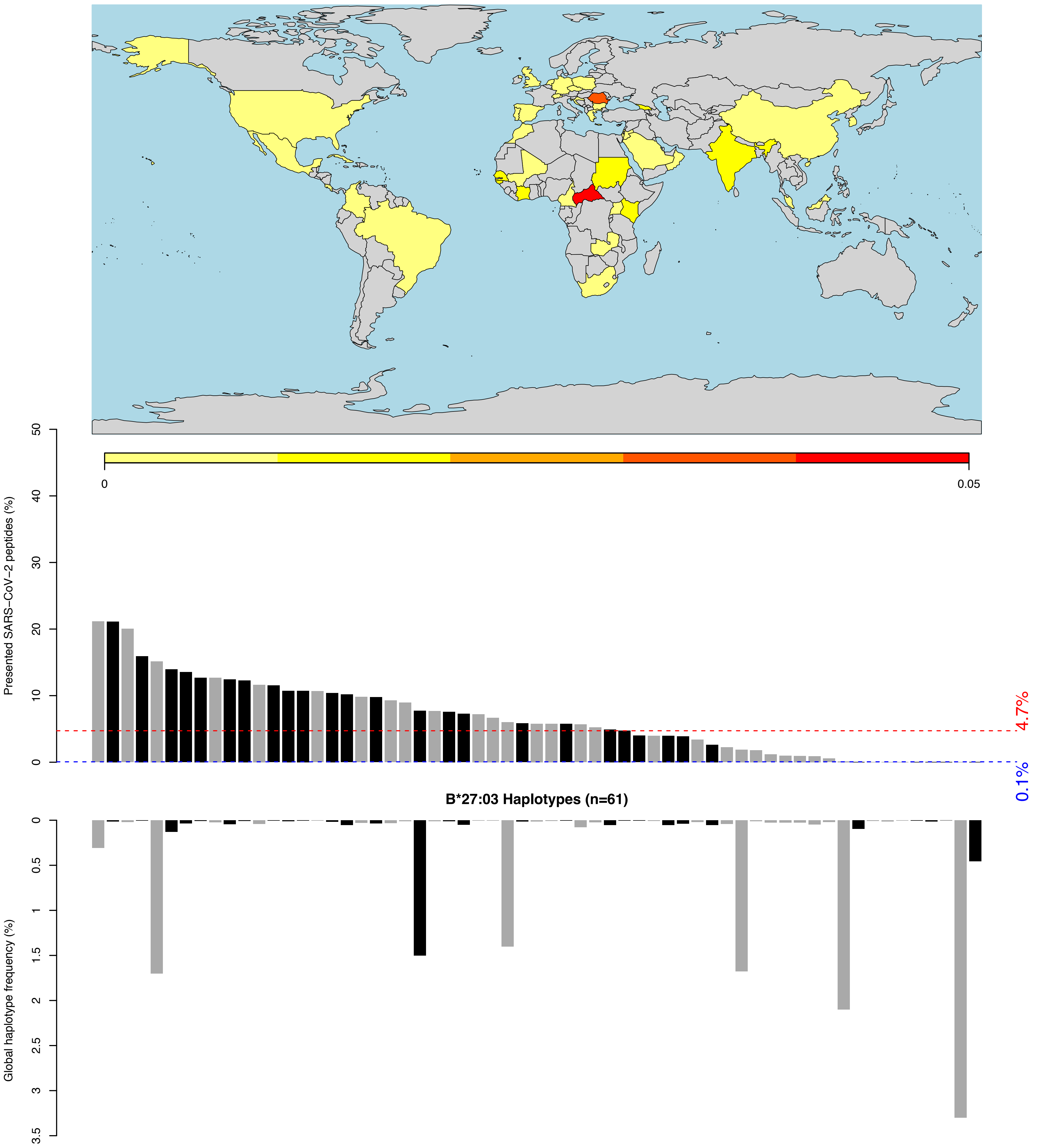

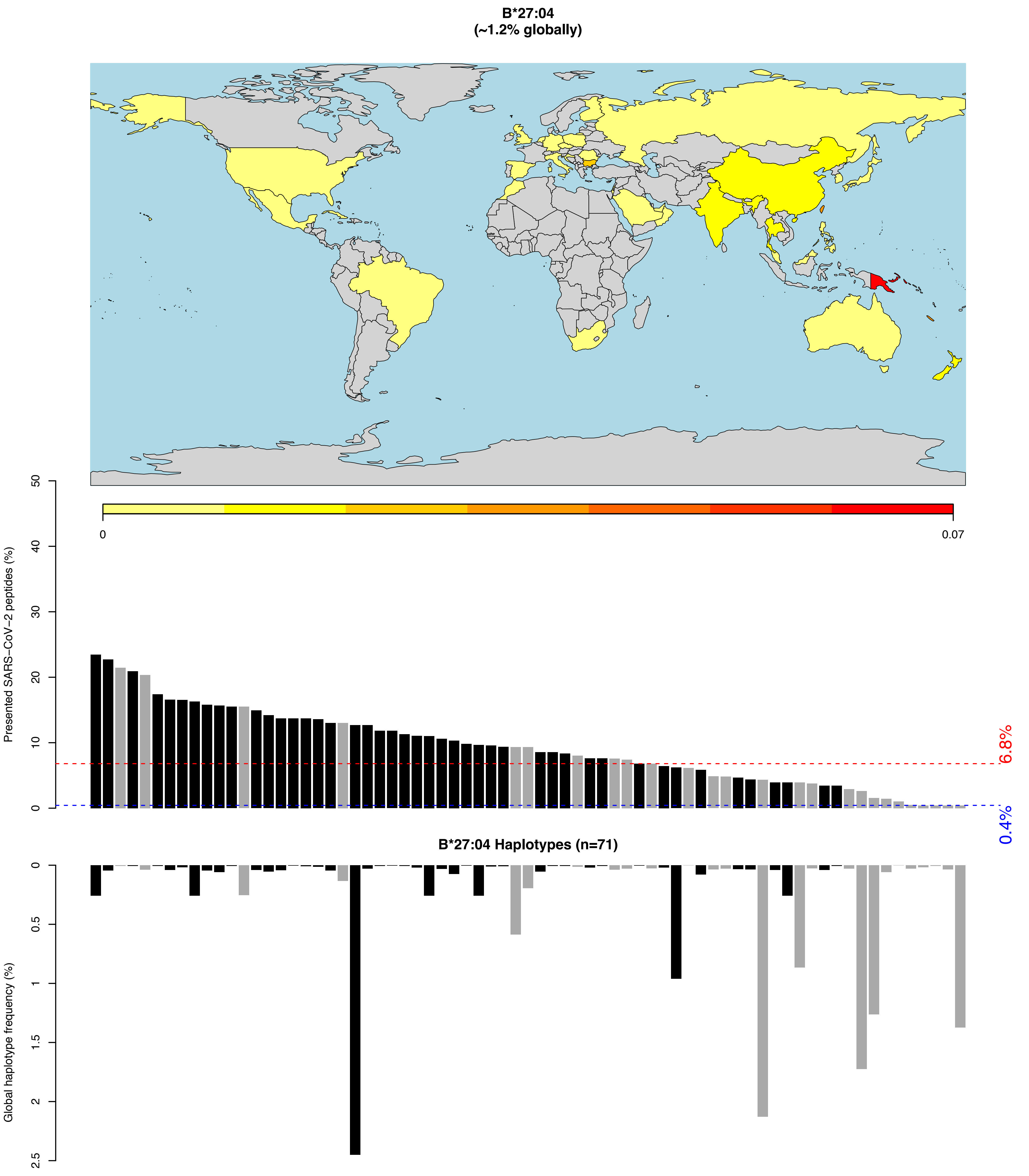
B*27:04
(~1.2% globally)
0
0.07
Presented SARS−CoV−2 peptides (%)
6.8%
0.4%
B*27:04 Haplotypes (n=71)
Global haplotype frequency (%)

B*27:05
(~0.61% globally)

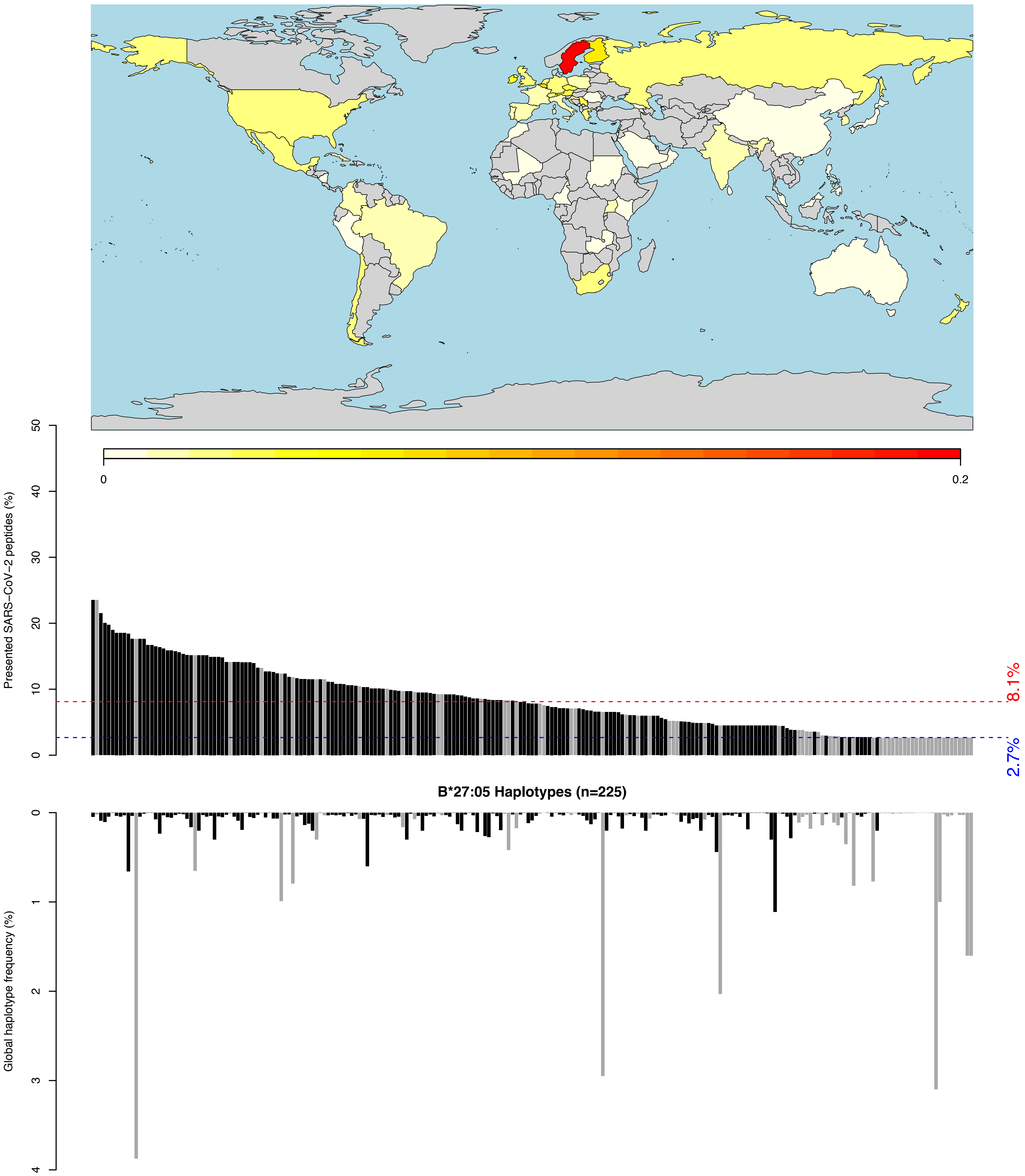

B*35:01
(~2.7% globally)

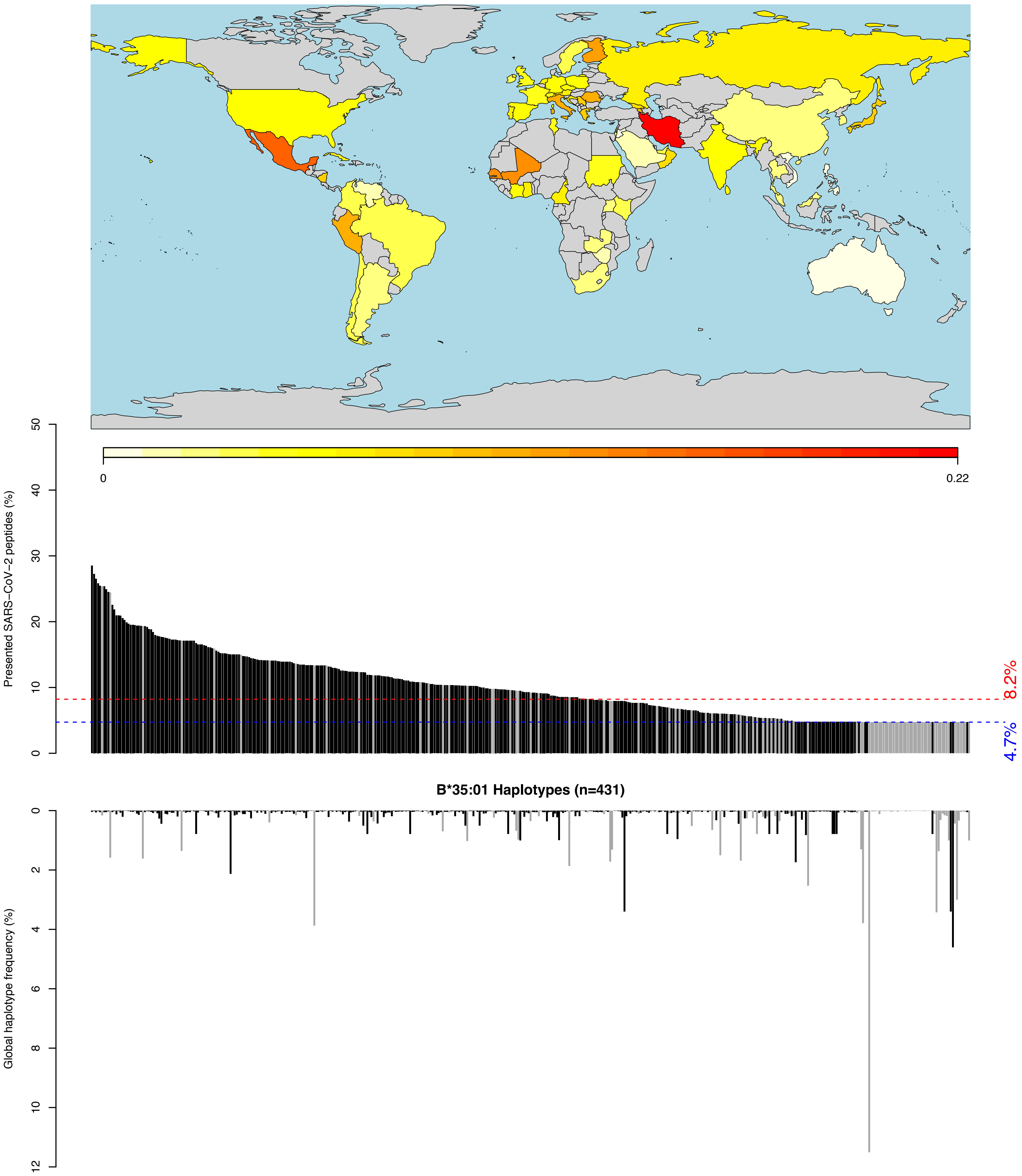

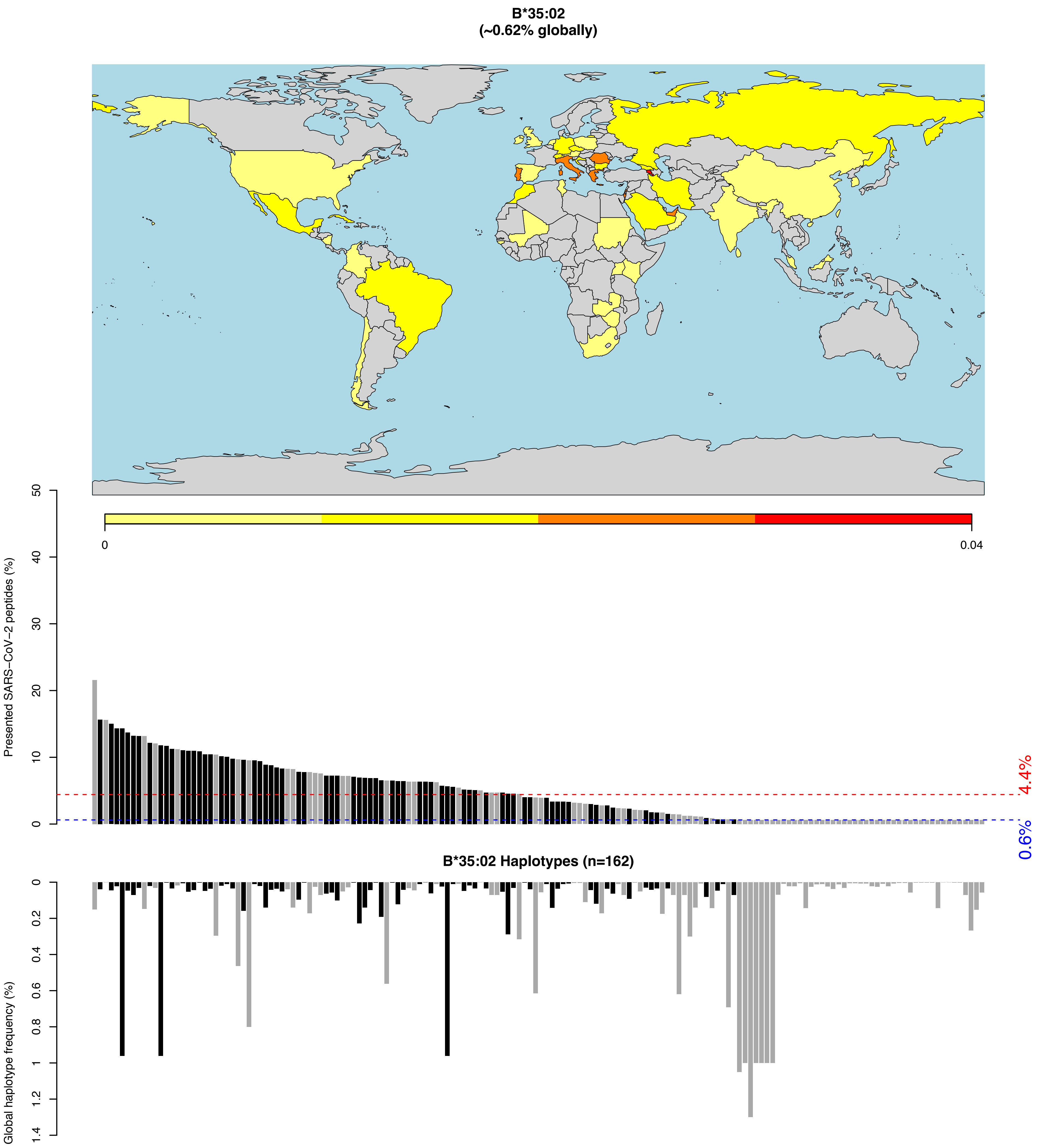

B*35:02
(~0.62% globally)
0
0.04
Presented SARS−CoV−2 peptides (%)
50
40
30
20
10
0
4.4%
0.6%
B*35:02 Haplotypes (n=162)
Global haplotype frequency (%)
0
0.2
0.4
0.6
0.8
1
1.2
1.4

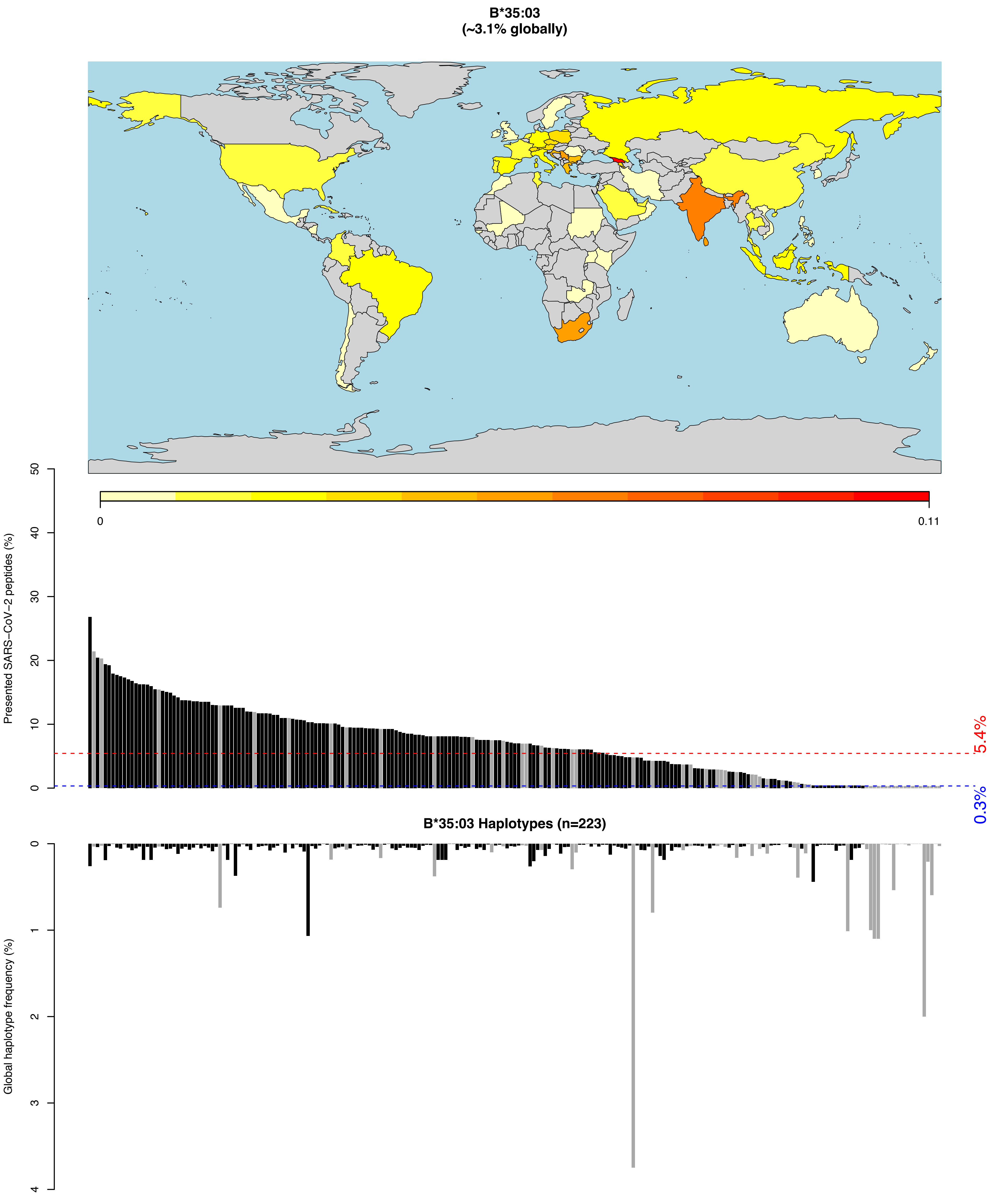

B*35:03
(~3.1% globally)
0
0.11
Presented SARS−CoV−2 peptides (%)
50
40
30
20
10
0
5.4%
0.3%
B*35:03 Haplotypes (n=223)
Global haplotype frequency (%)
0
1
2
3
4

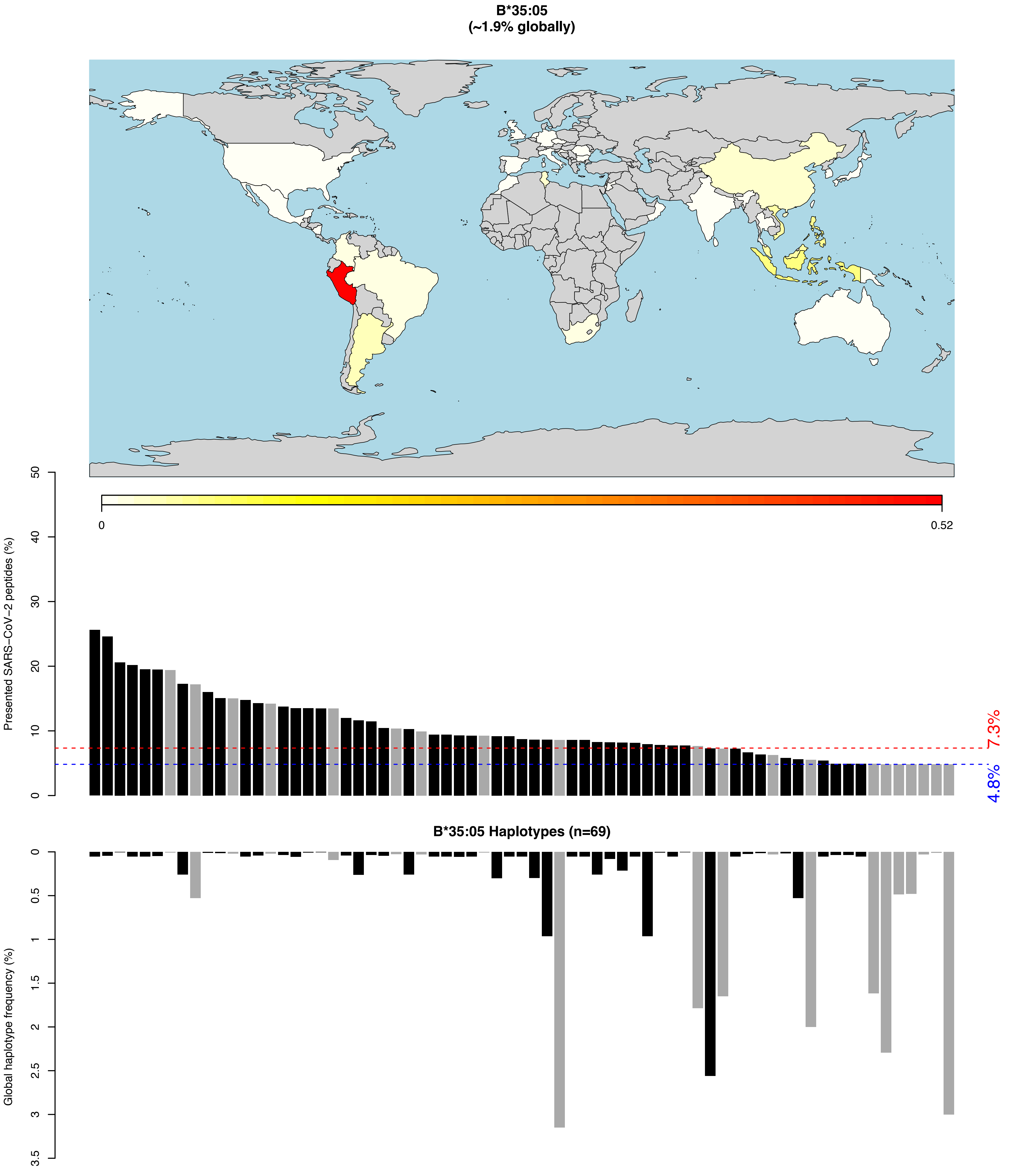

B*35:05
(~1.9% globally)
0
0.52
Presented SARS−CoV−2 peptides (%)
50
40
30
20
10
0
7.3%
4.8%
B*35:05 Haplotypes (n=69)
Global haplotype frequency (%)
0
0.5
1
1.5
2
2.5
3
3.5

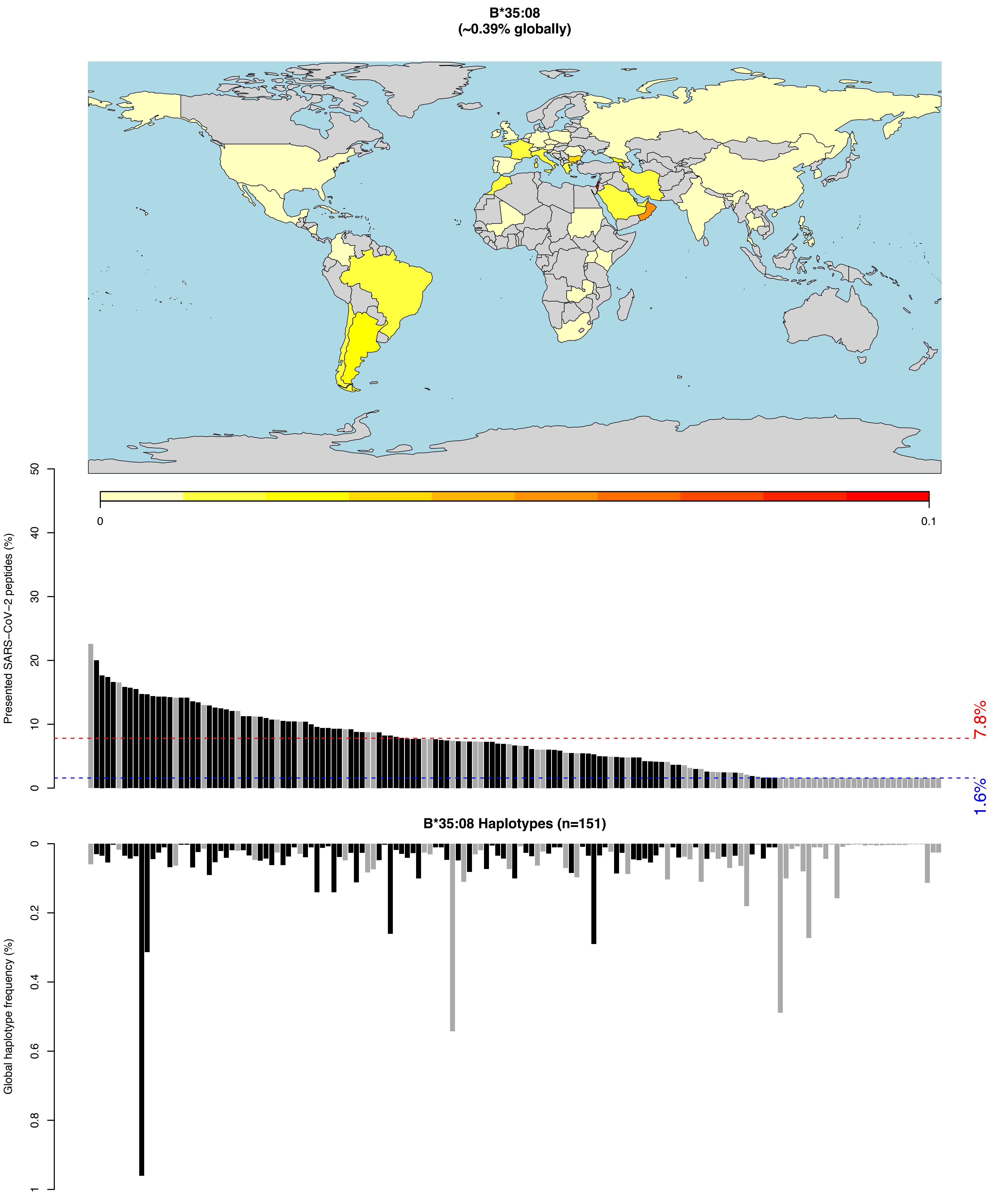
B*35:08
(~0.39% globally)
0
0.1
Presented SARS−CoV−2 peptides (%)
50
40
30
20
10
0
7.8%
1.6%
B*35:08 Haplotypes (n=151)
Global haplotype frequency (%)
0
0.2
0.4
0.6
0.8
1

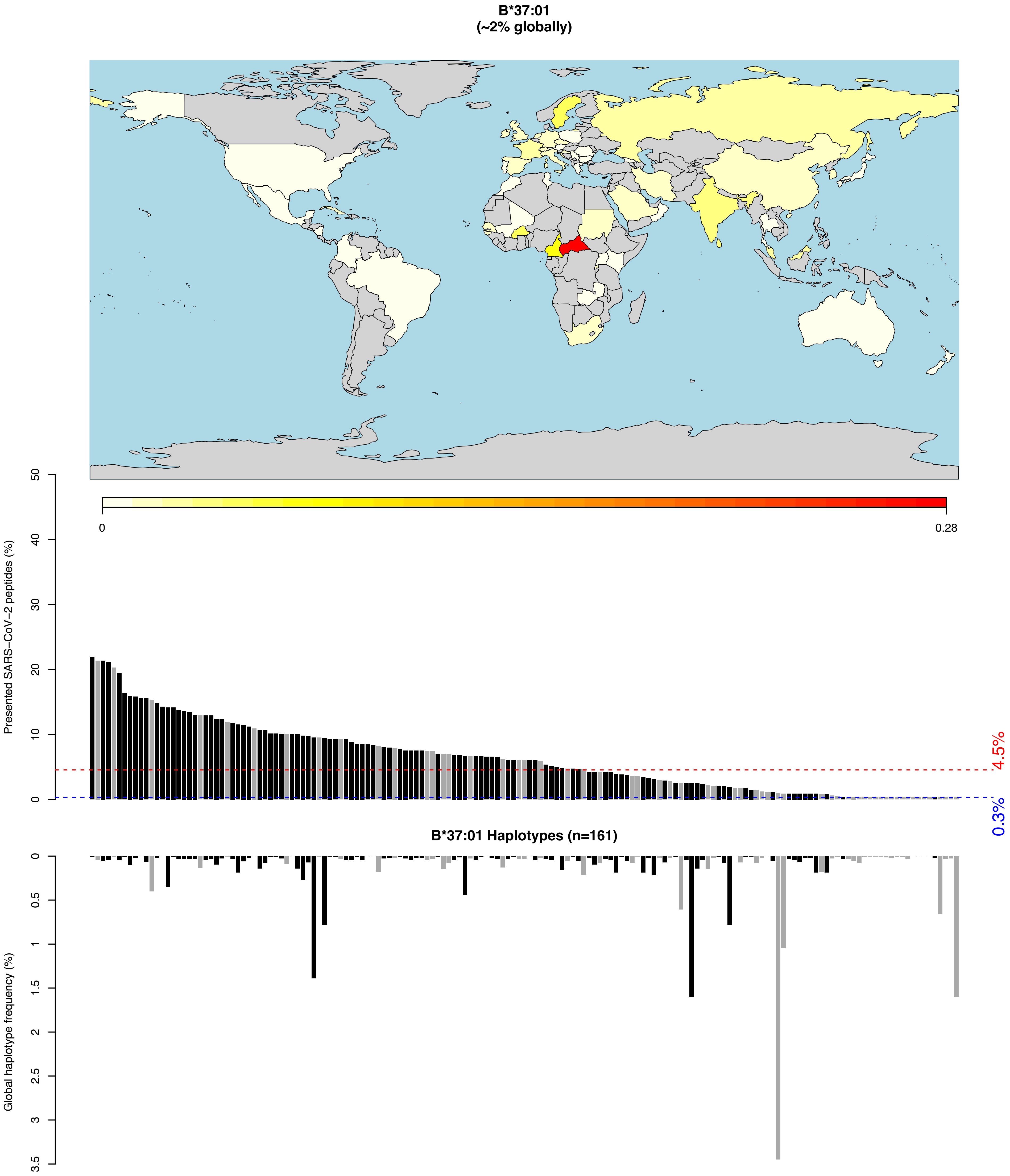

B*37:01
(~2% globally)
0
0.28
Presented SARS−CoV−2 peptides (%)
50
40
30
20
10
0
4.5%
0.3%
B*37:01 Haplotypes (n=161)
Global haplotype frequency (%)
0
0.5
1
1.5
2
2.5
3
3.5

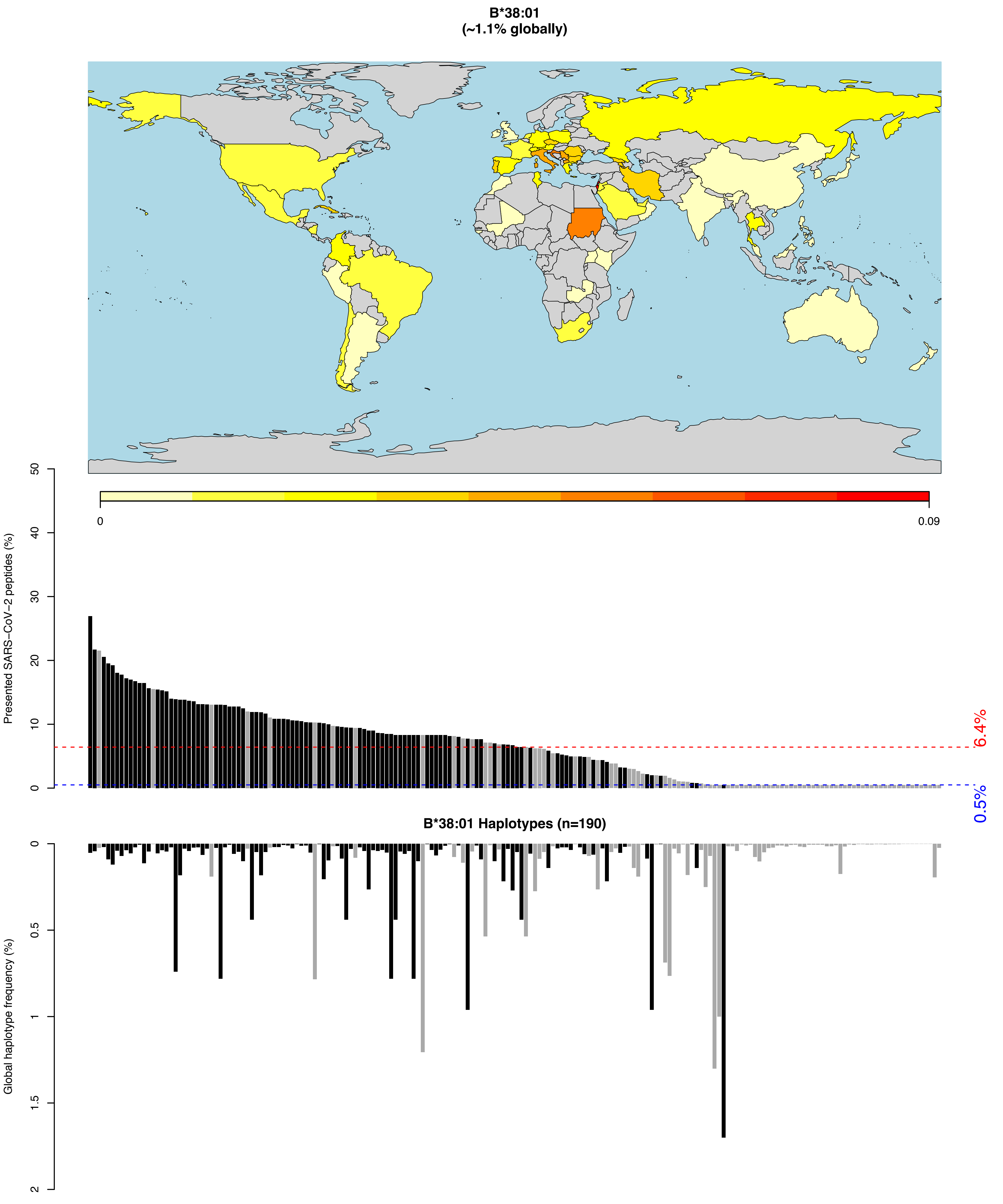
B*38:01
(~1.1% globally)
0
0.09
Presented SARS−CoV−2 peptides (%)
50
40
30
20
10
0
6.4%
0.5%
B*38:01 Haplotypes (n=190)
Global haplotype frequency (%)
0
0.5
1
1.5
2

B*38:02
(~2.1% globally)

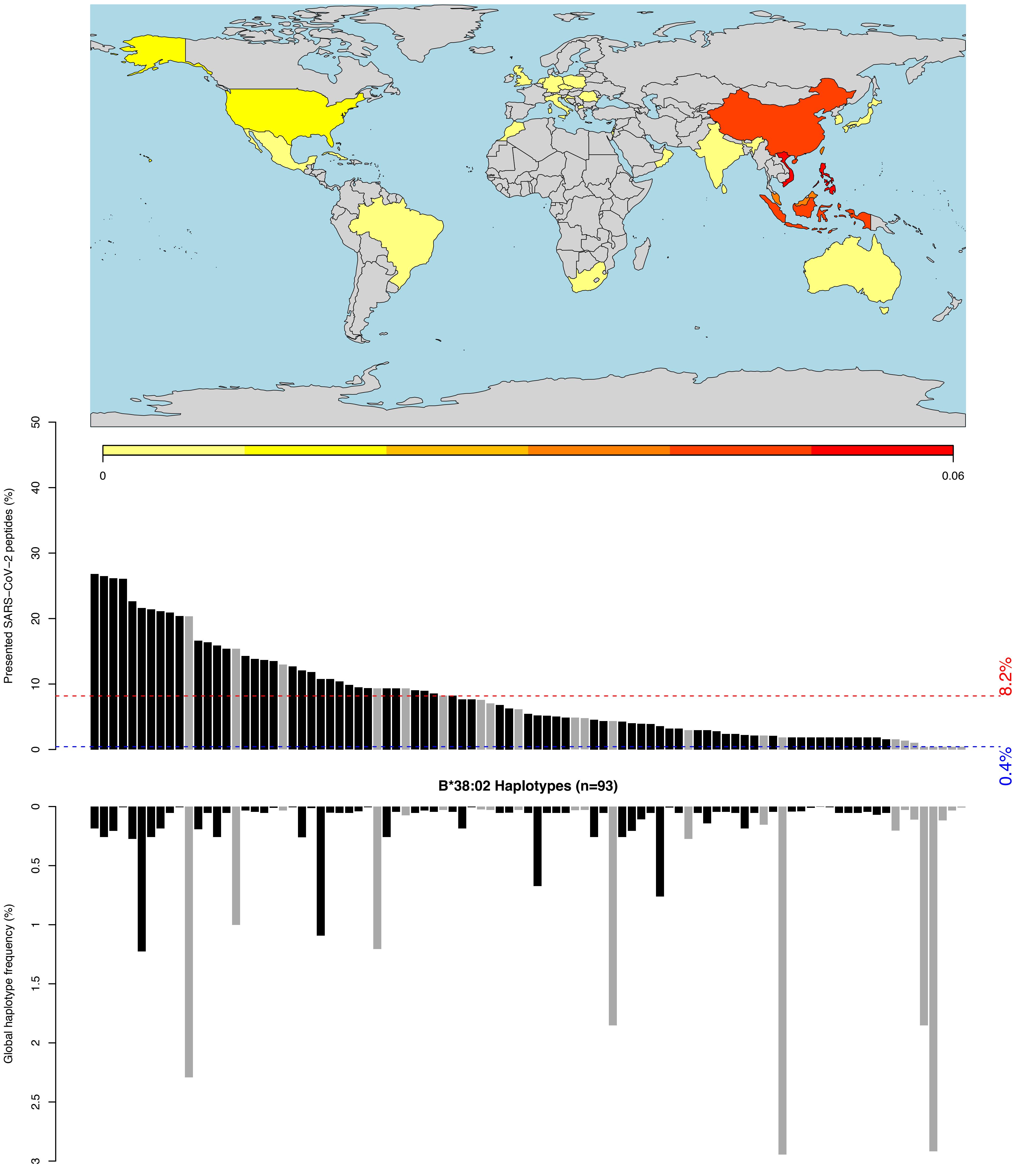

B*39:01
(~1.2% globally)

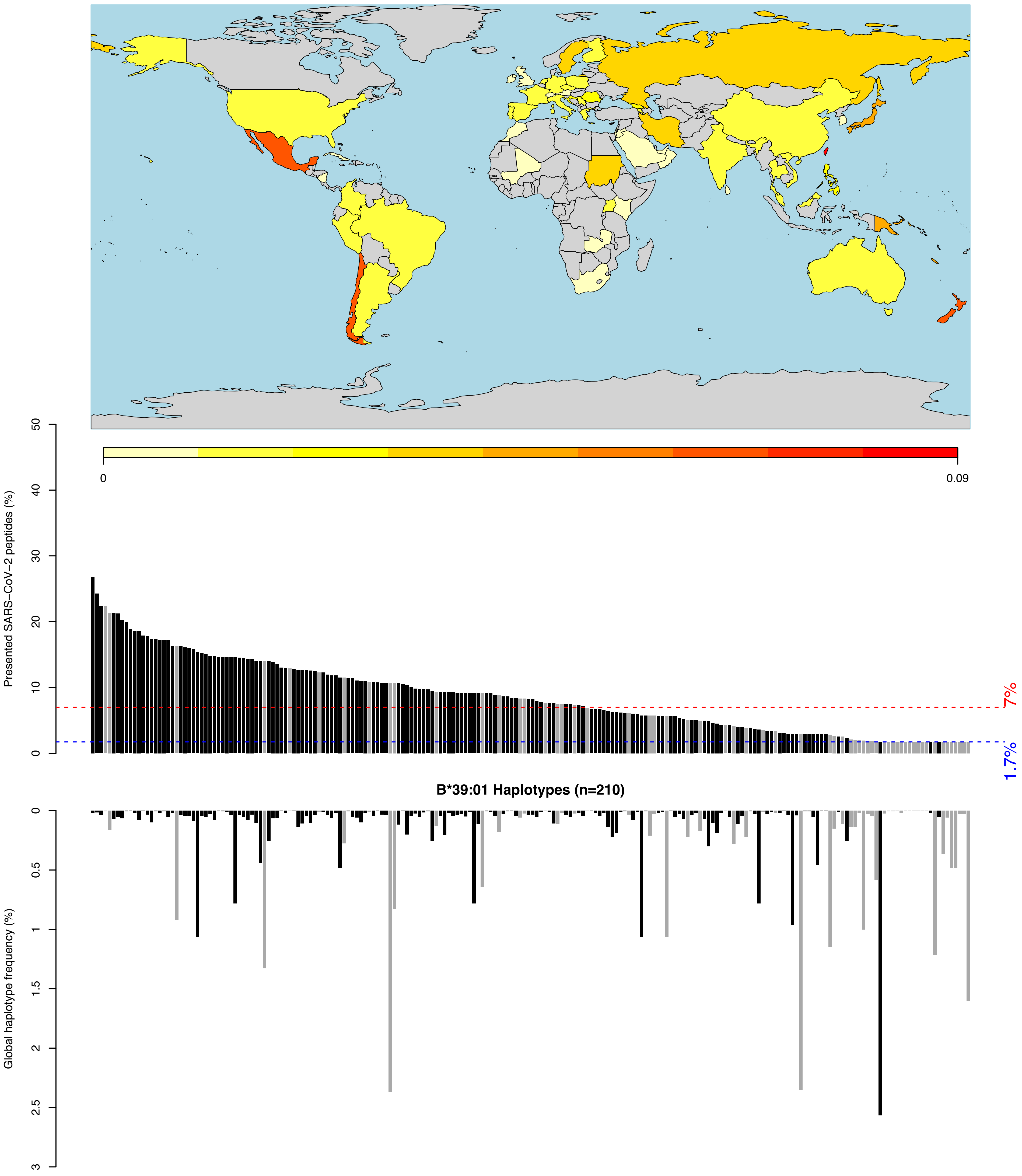

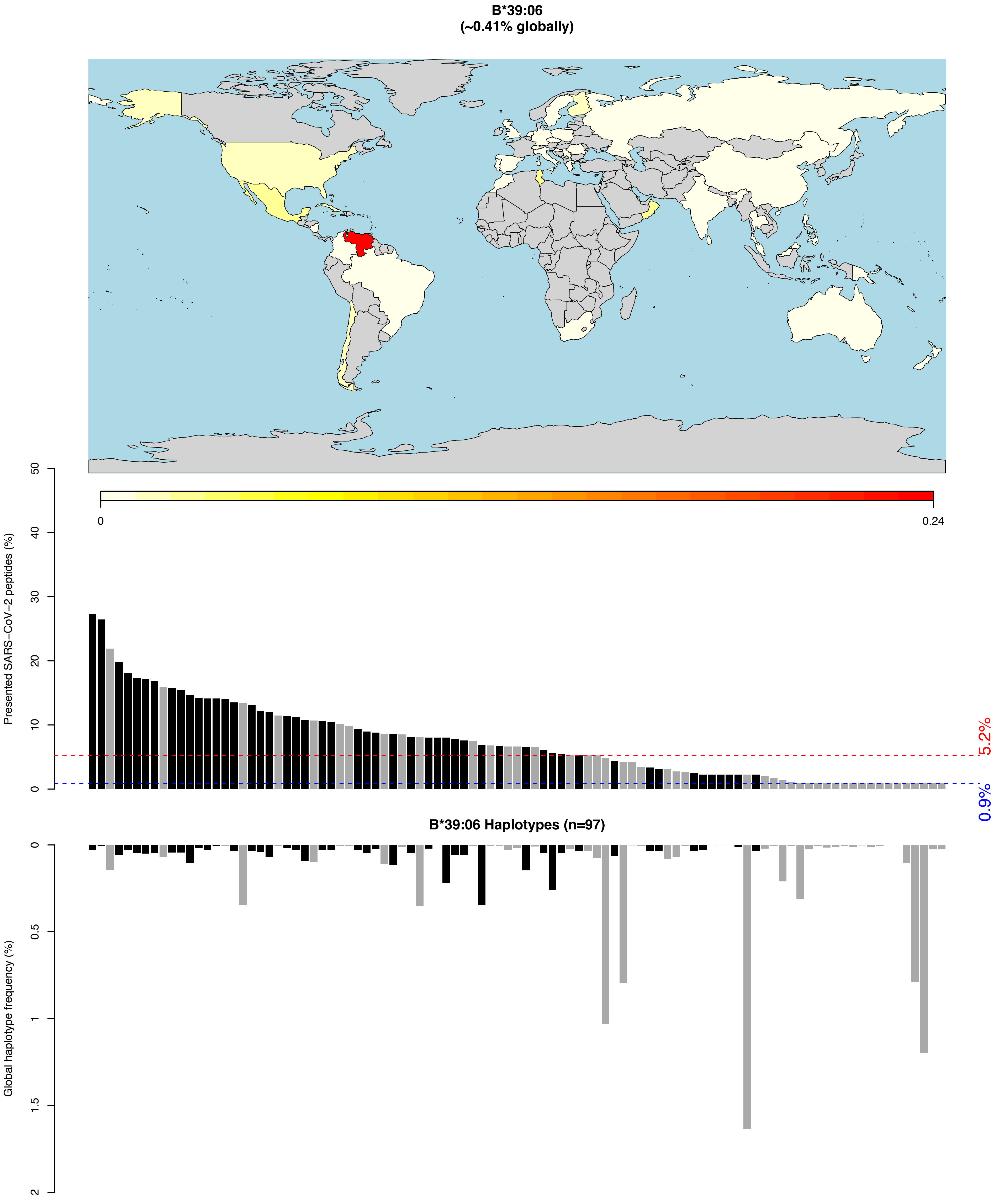

B*39:06
(~0.41% globally)
0
0.24
Presented SARS−CoV−2 peptides (%)
50
40
30
20
10
0
5.2%
0.9%
B*39:06 Haplotypes (n=97)
Global haplotype frequency (%)
0
0.5
1
1.5
2

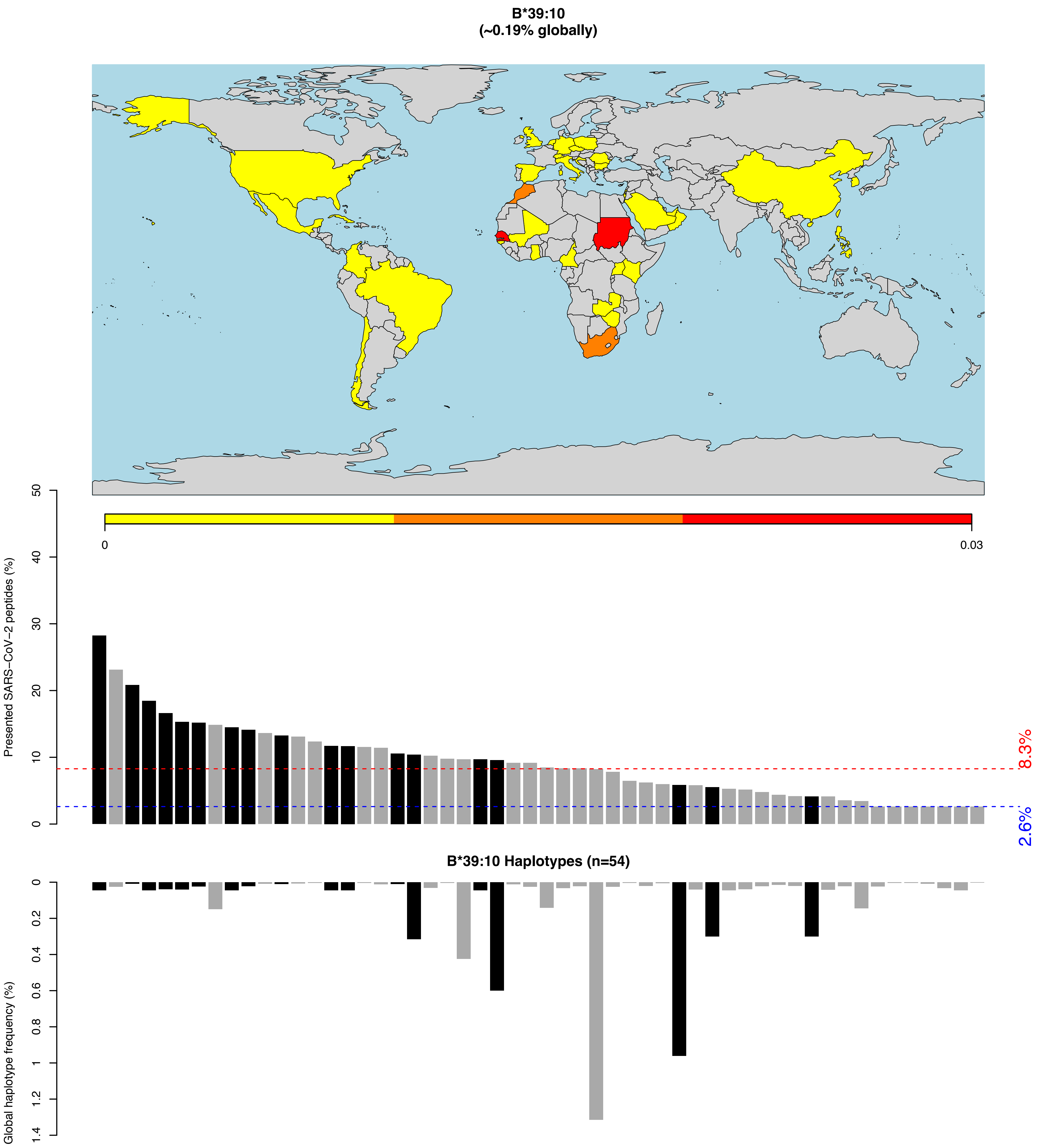

B*39:10
(~0.19% globally)
0
0.03
Presented SARS−CoV−2 peptides (%)
50
40
30
20
10
0
8.3%
2.6%
B*39:10 Haplotypes (n=54)
Global haplotype frequency (%)
0
0.2
0.4
0.6
0.8
1
1.2
1.4

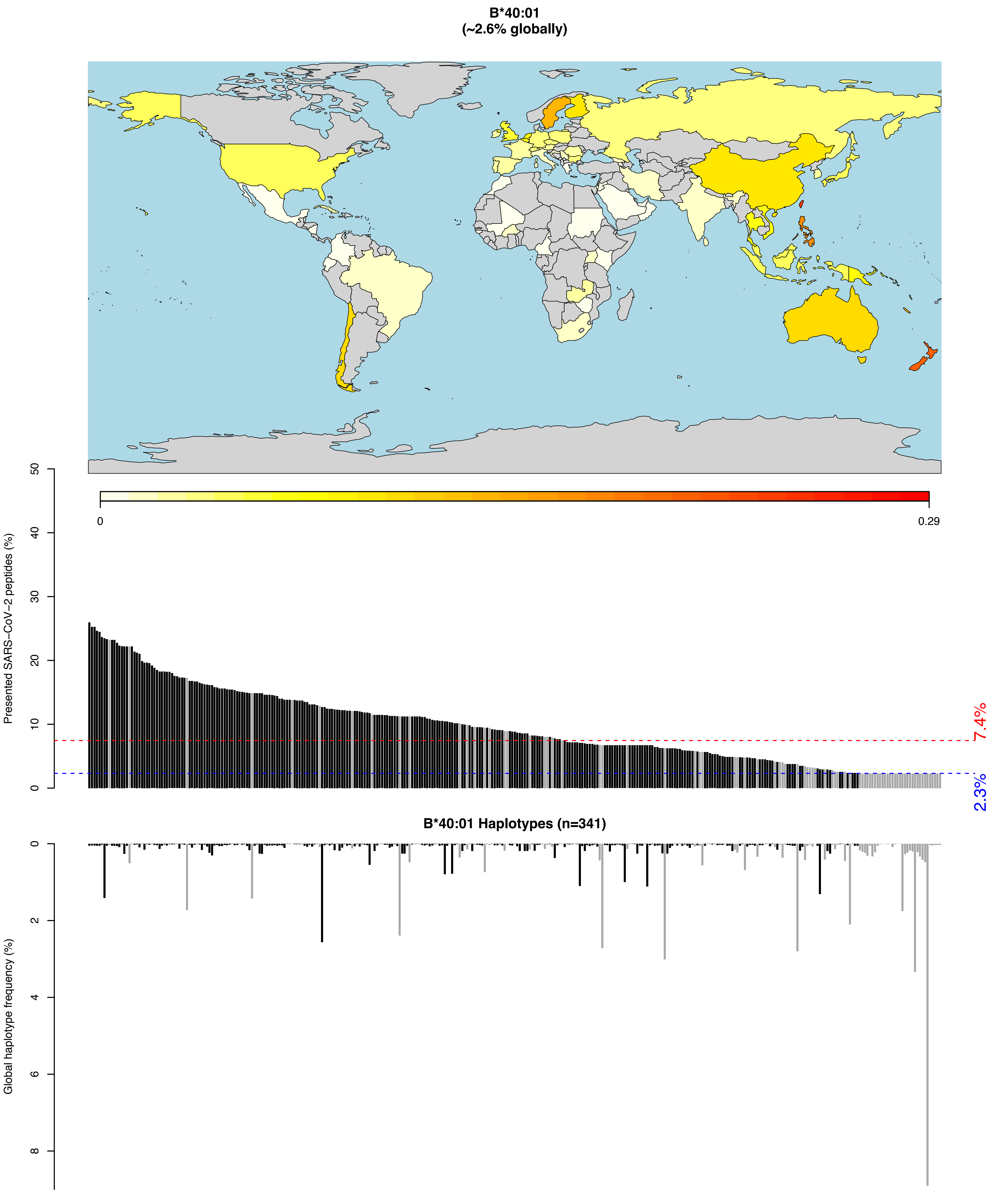
B*40:01
(~2.6% globally)
0
0.29
Presented SARS−CoV−2 peptides (%)
50
40
30
20
10
0
7.4%
2.3%
B*40:01 Haplotypes (n=341)
Global haplotype frequency (%)
0
2
4
6
8

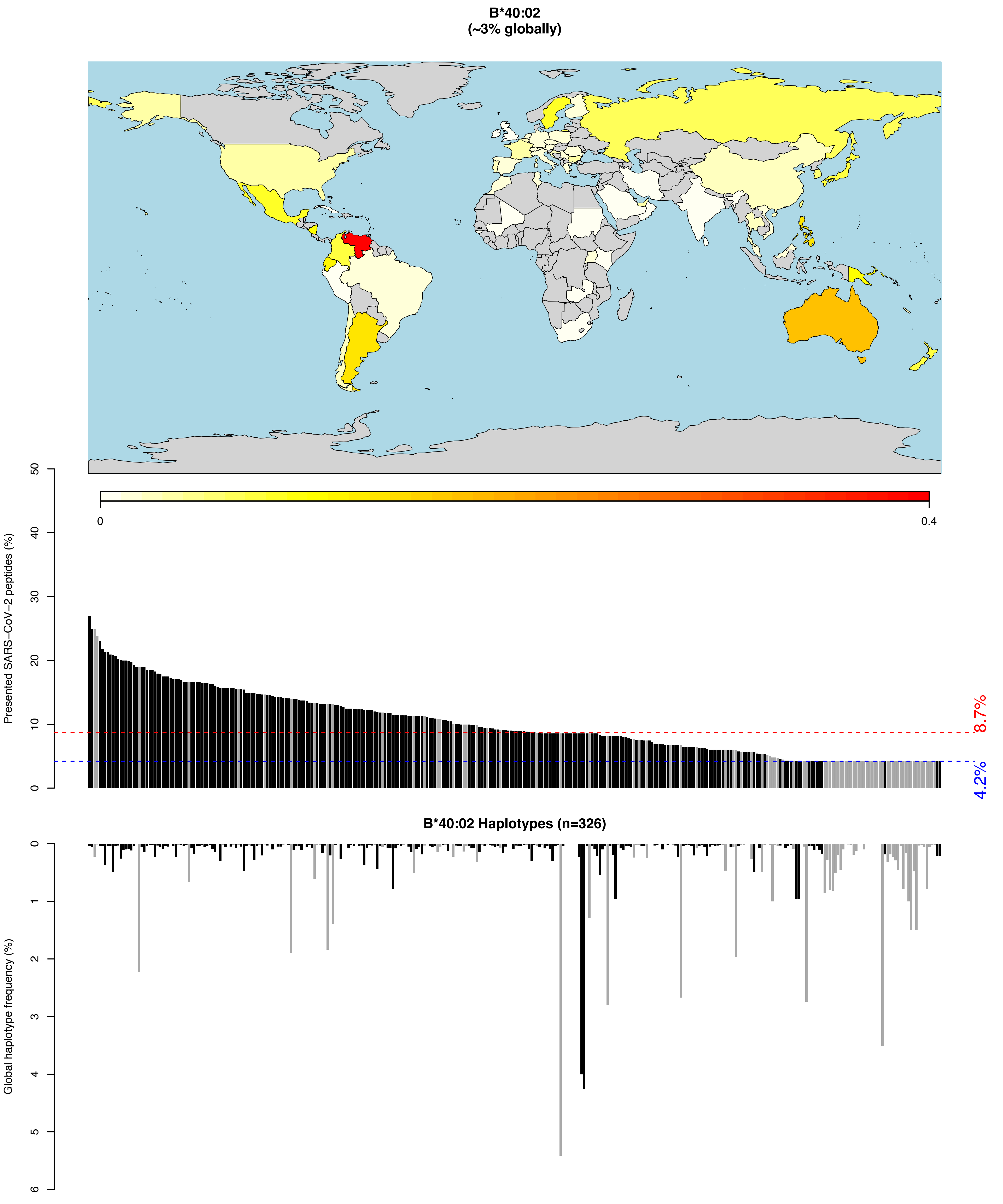
B*40:02
(~3% globally)
0
0.4
Presented SARS−CoV−2 peptides (%)
50
40
30
20
10
0
8.7%
4.2%
B*40:02 Haplotypes (n=326)
Global haplotype frequency (%)
0
1
2
3
4
5
6

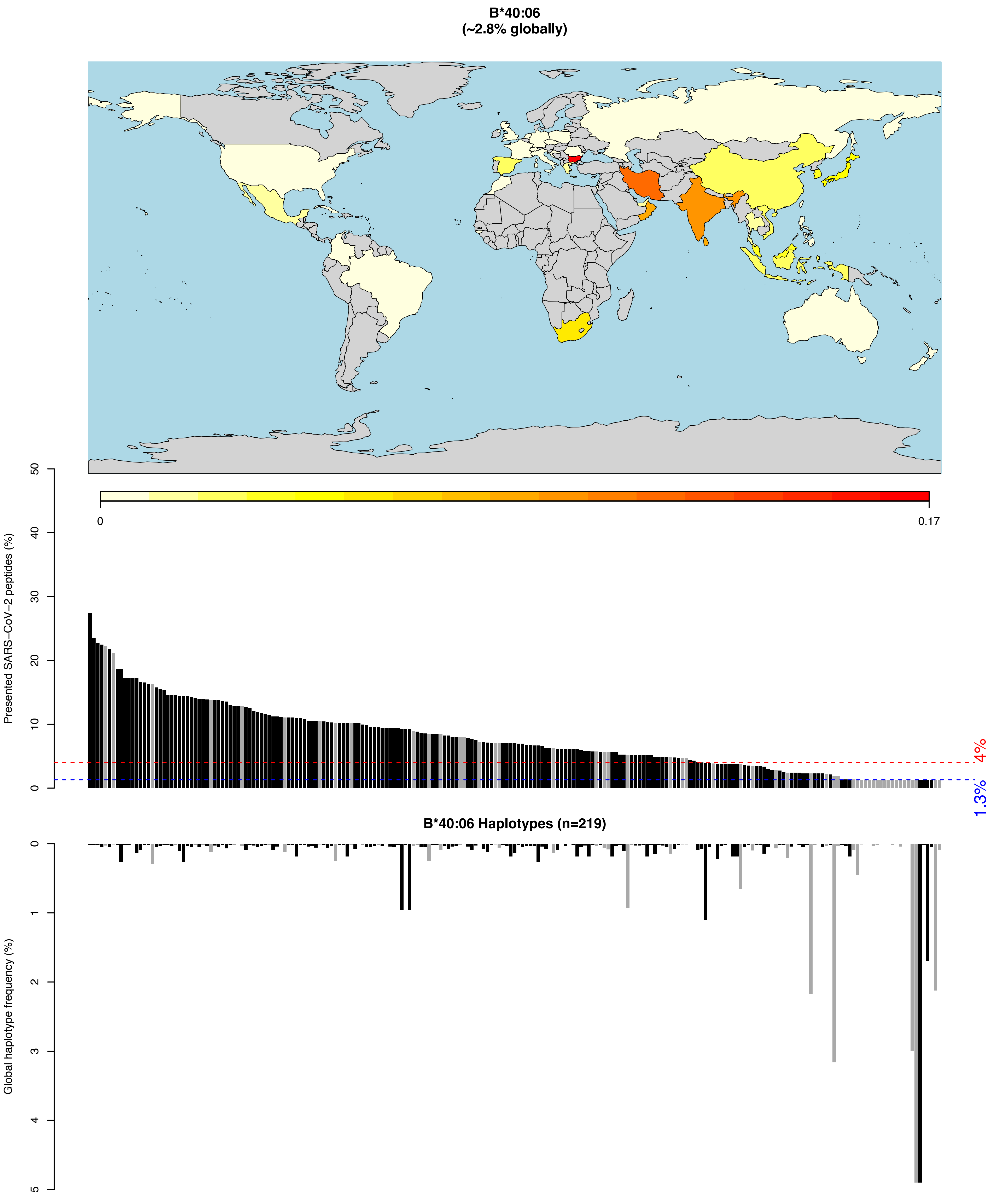
B*40:06
(~2.8% globally)
0
0.17
Presented SARS−CoV−2 peptides (%)
50
40
30
20
10
0
4%
1.3%
B*40:06 Haplotypes (n=219)
Global haplotype frequency (%)
0
1
2
3
4
5

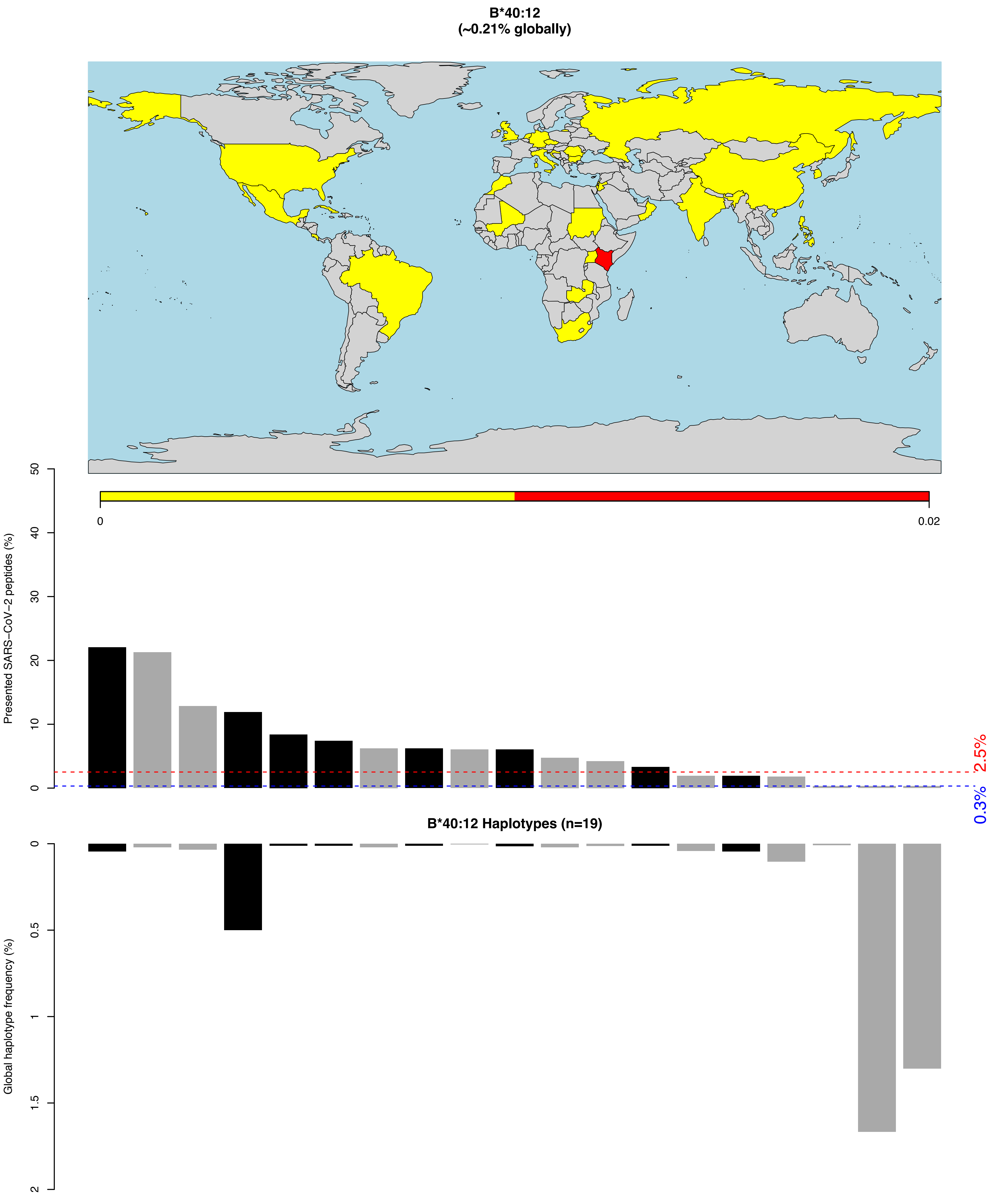

B*40:12
(~0.21% globally)
0
0.02
Presented SARS−CoV−2 peptides (%)
50
40
30
20
10
0
2.5%
0.3%
B*40:12 Haplotypes (n=19)
Global haplotype frequency (%)
0
0.5
1
1.5
2

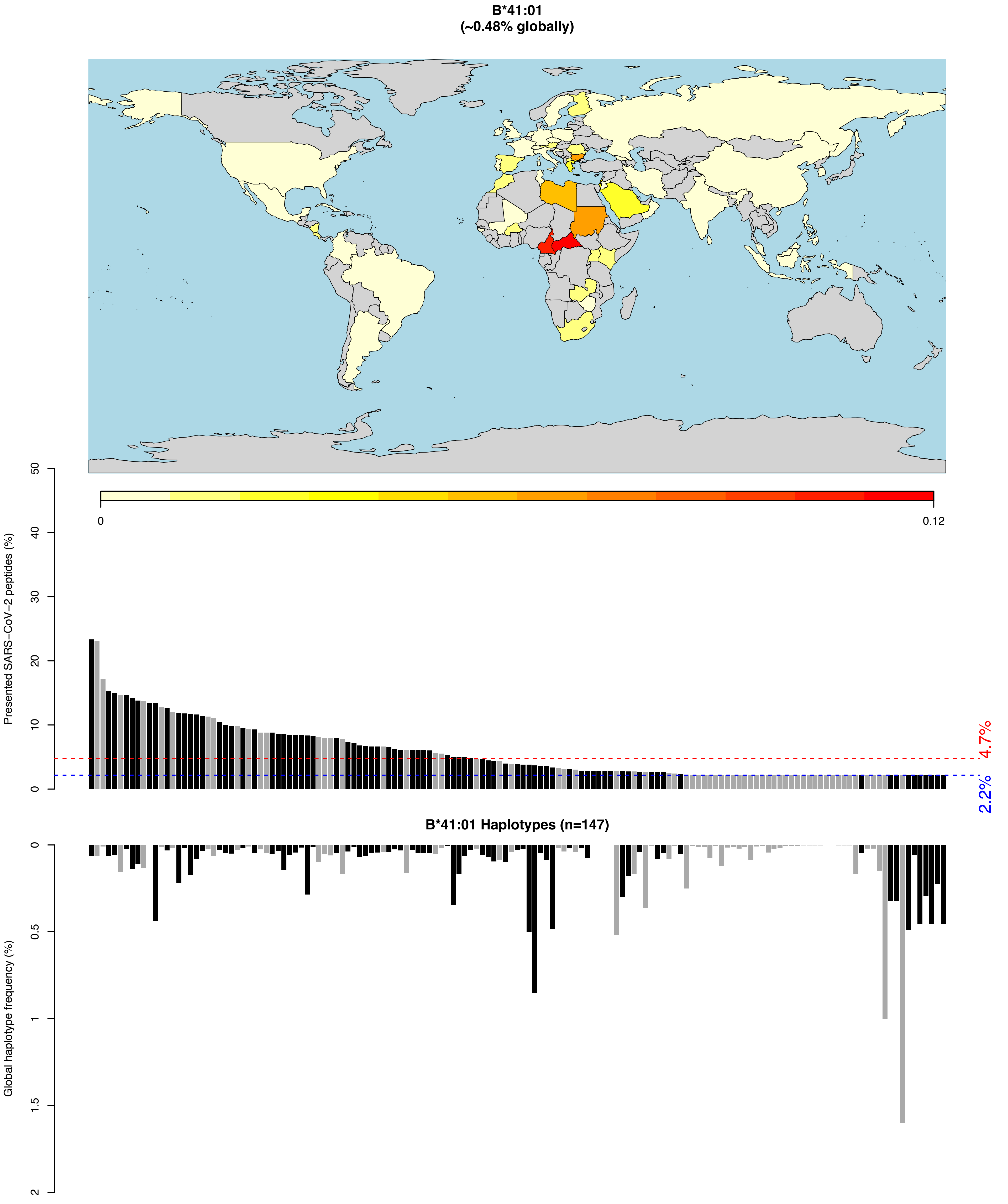

B*41:01
(~0.48% globally)
0
0.12
Presented SARS−CoV−2 peptides (%)
0
10
20
30
40
50
4.7%
2.2%
B*41:01 Haplotypes (n=147)
Global haplotype frequency (%)
0
0.5
1
1.5
2

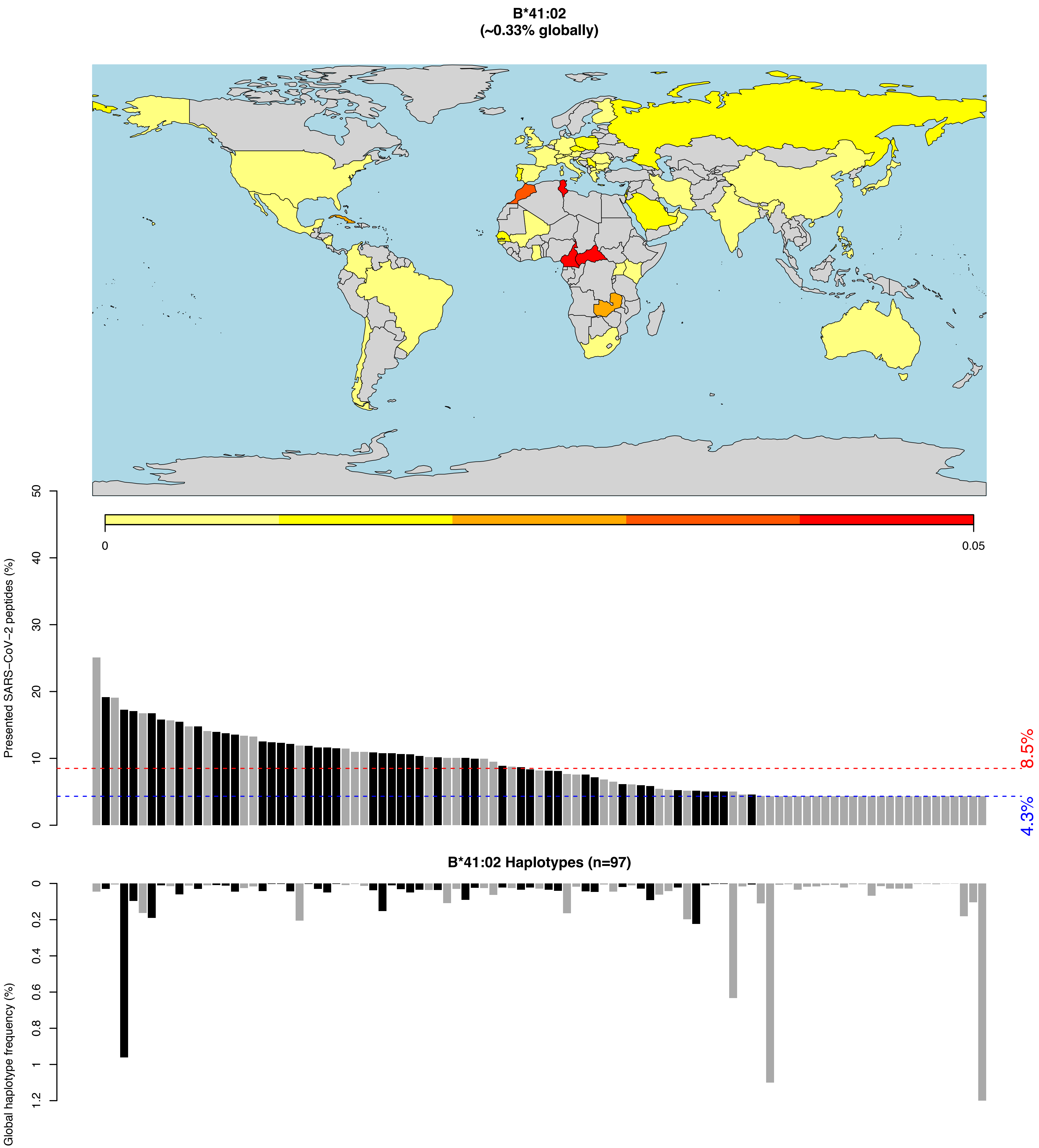

B*41:02
(~0.33% globally)
0
0.05
Presented SARS−CoV−2 peptides (%)
50
40
30
20
10
0
8.5%
4.3%
B*41:02 Haplotypes (n=97)
Global haplotype frequency (%)
0
0.2
0.4
0.6
0.8
1
1.2

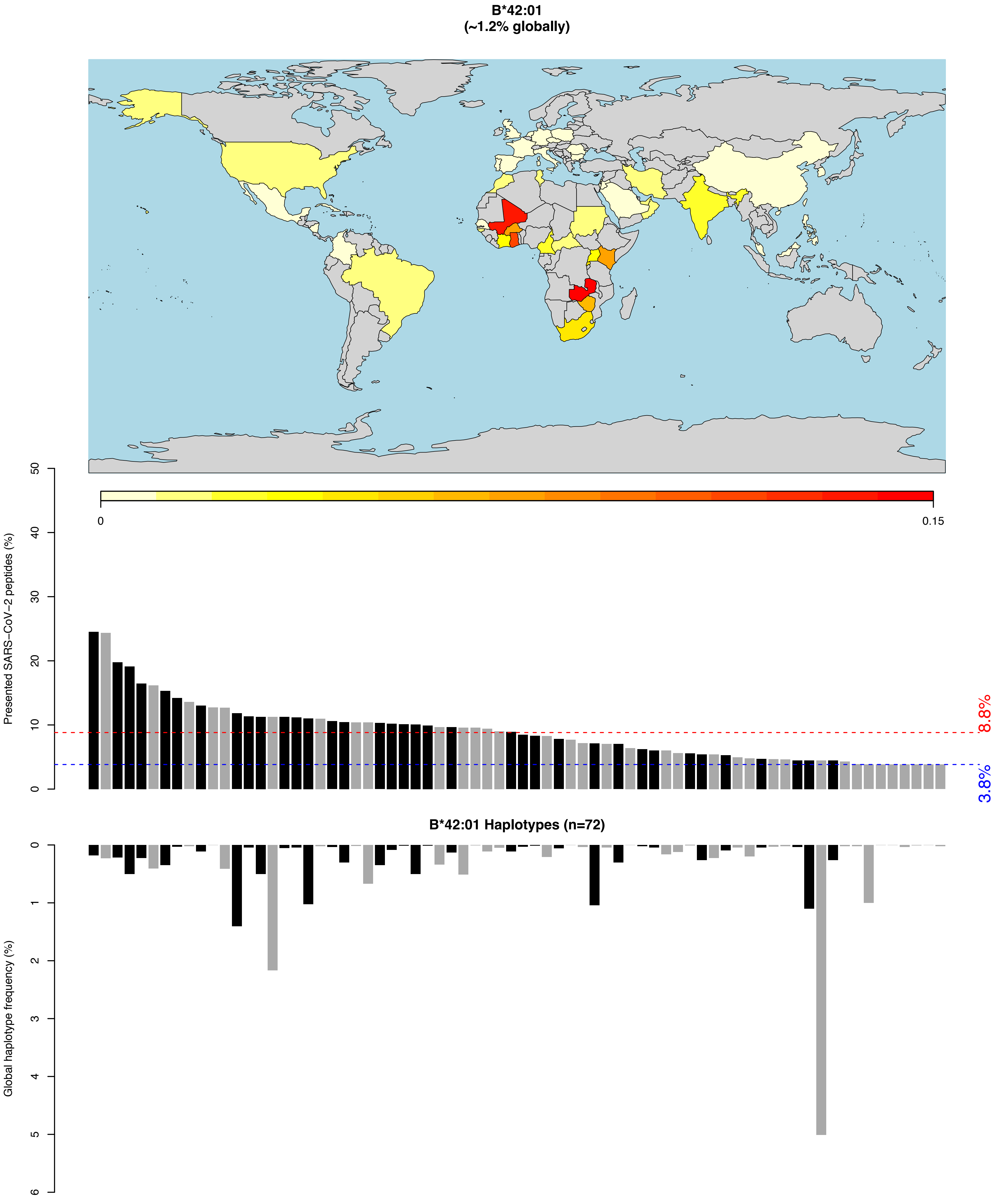

B*42:01
(~1.2% globally)
0
0.15
Presented SARS−CoV−2 peptides (%)
8.8%
3.8%
B*42:01 Haplotypes (n=72)
Global haplotype frequency (%)

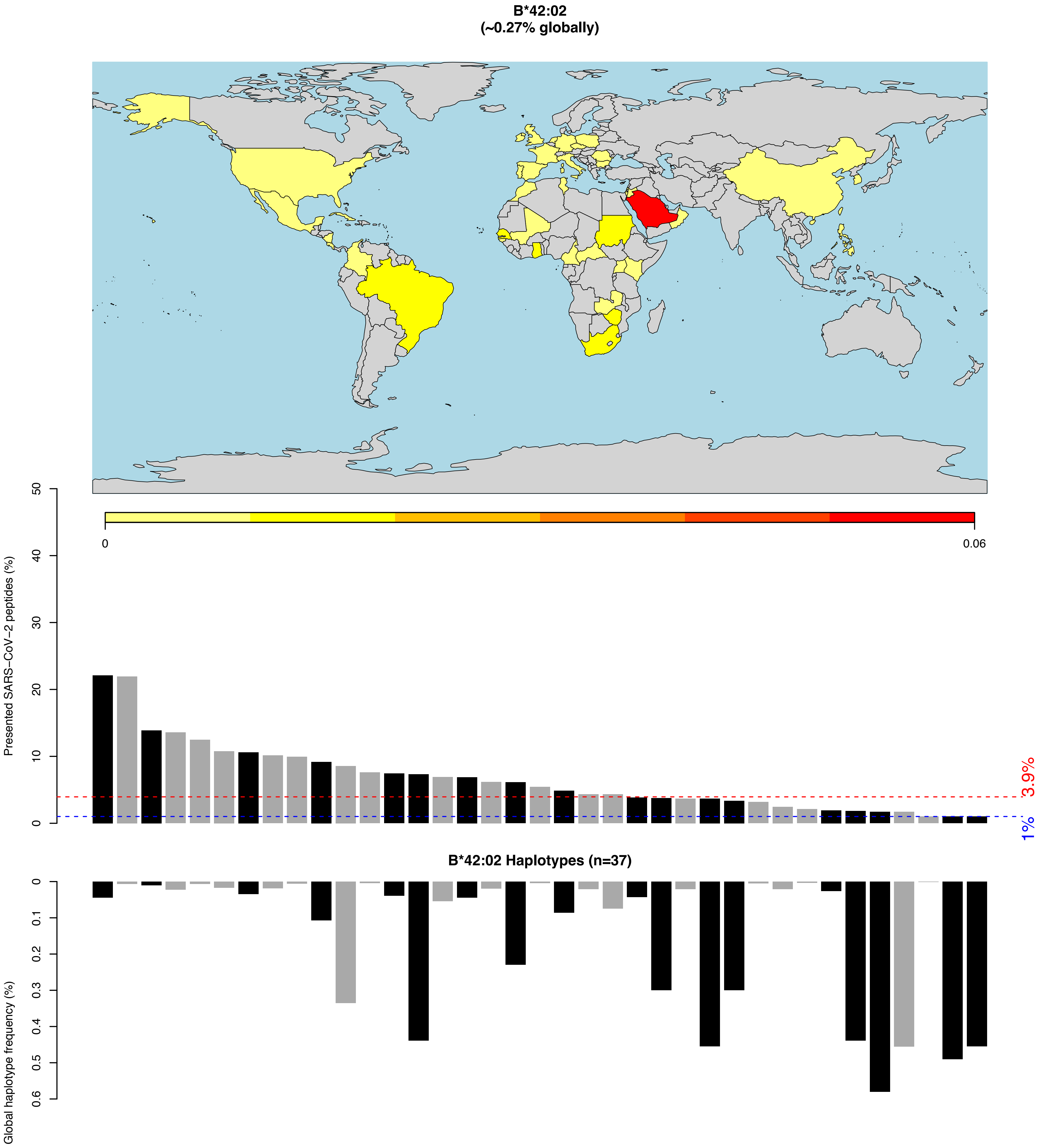

B*42:02
(~0.27% globally)
0
0.06
Presented SARS−CoV−2 peptides (%)
50
40
30
20
10
0
3.9%
1%
B*42:02 Haplotypes (n=37)
Global haplotype frequency (%)
0
0.1
0.2
0.3
0.4
0.5
0.6

B*44:02
(~1.3% globally)

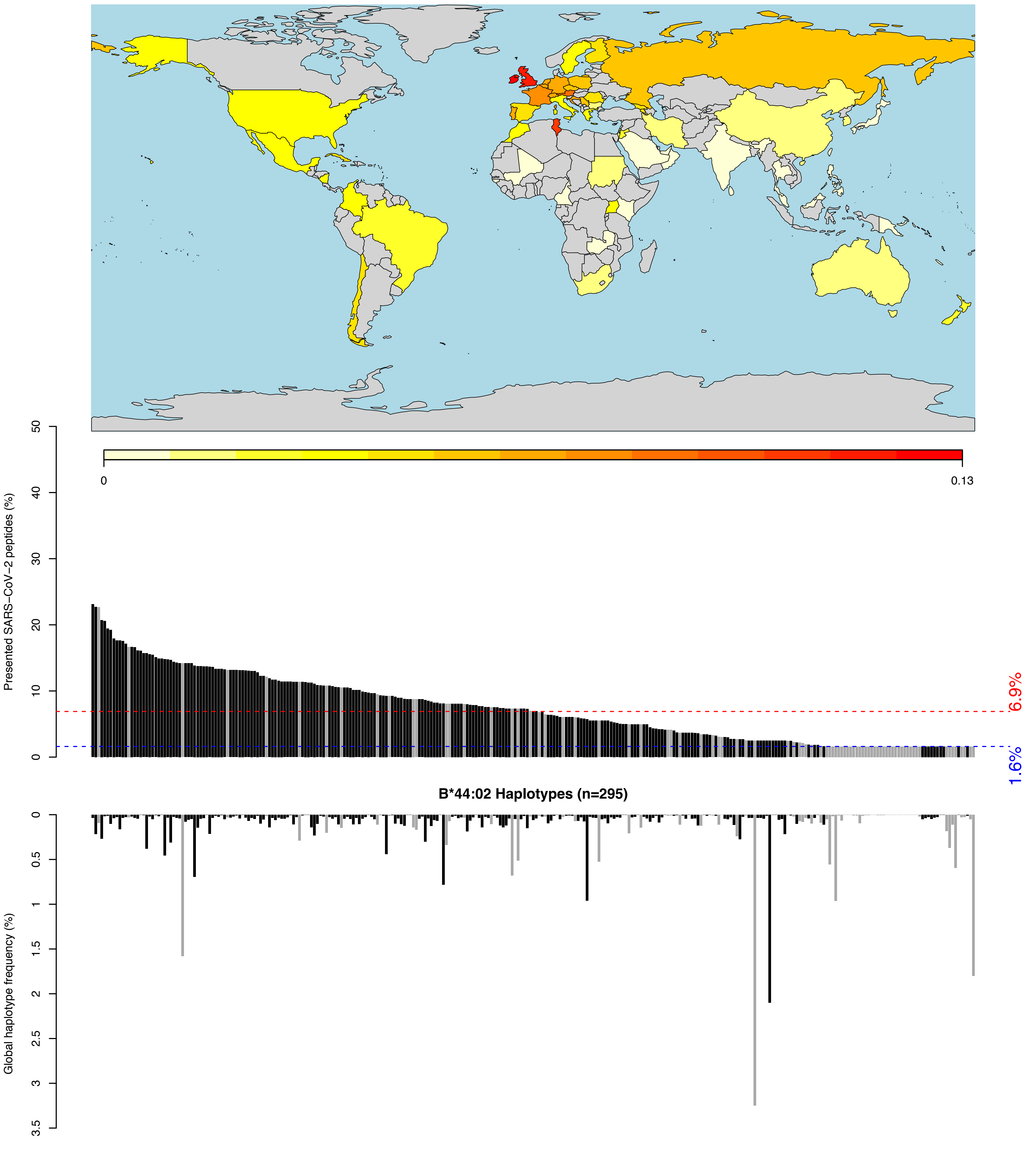

B*44:03
(~2.6% globally)

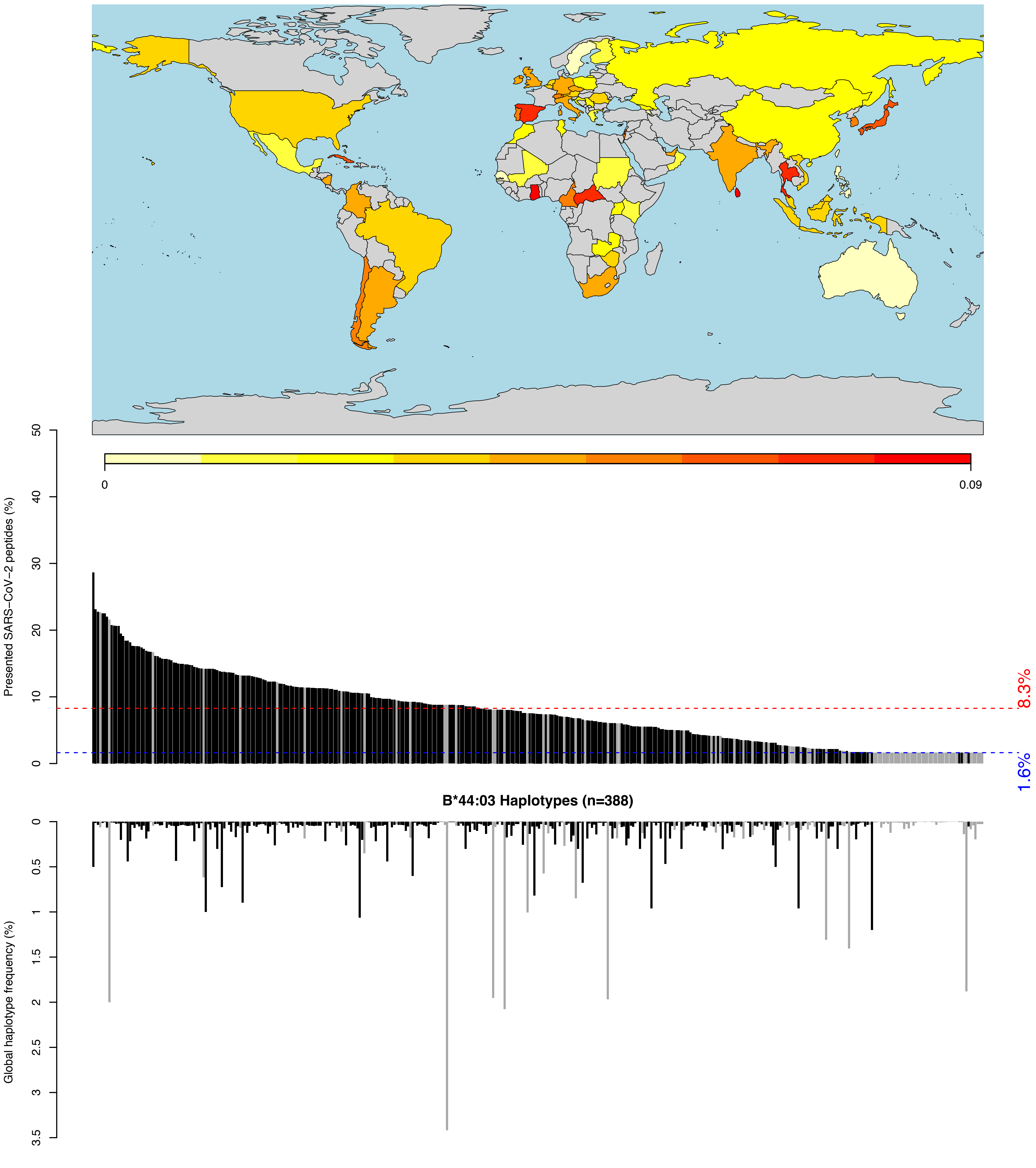

B*44:05
(~0.17% globally)

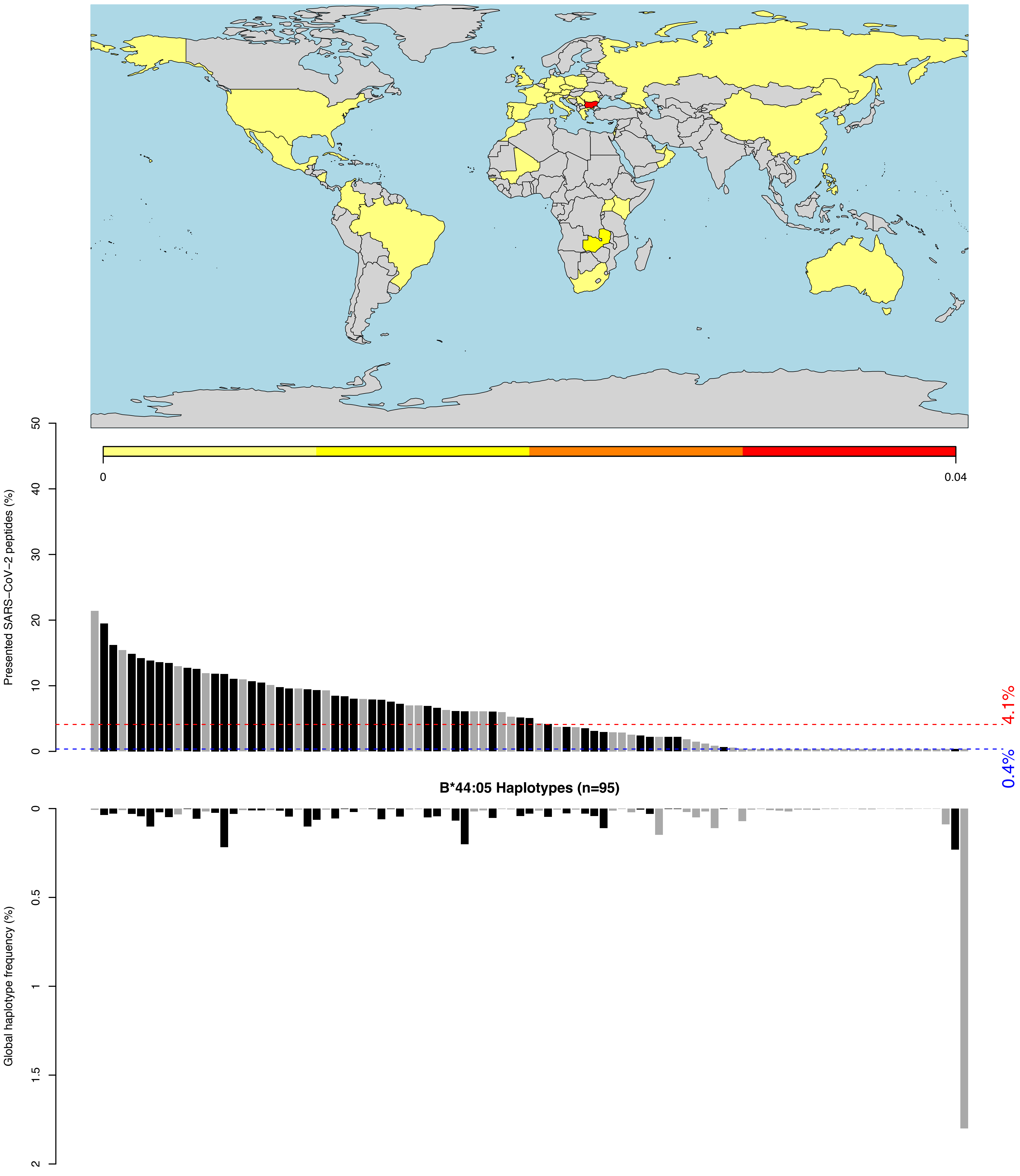

B*44:10
(~0.042% globally)

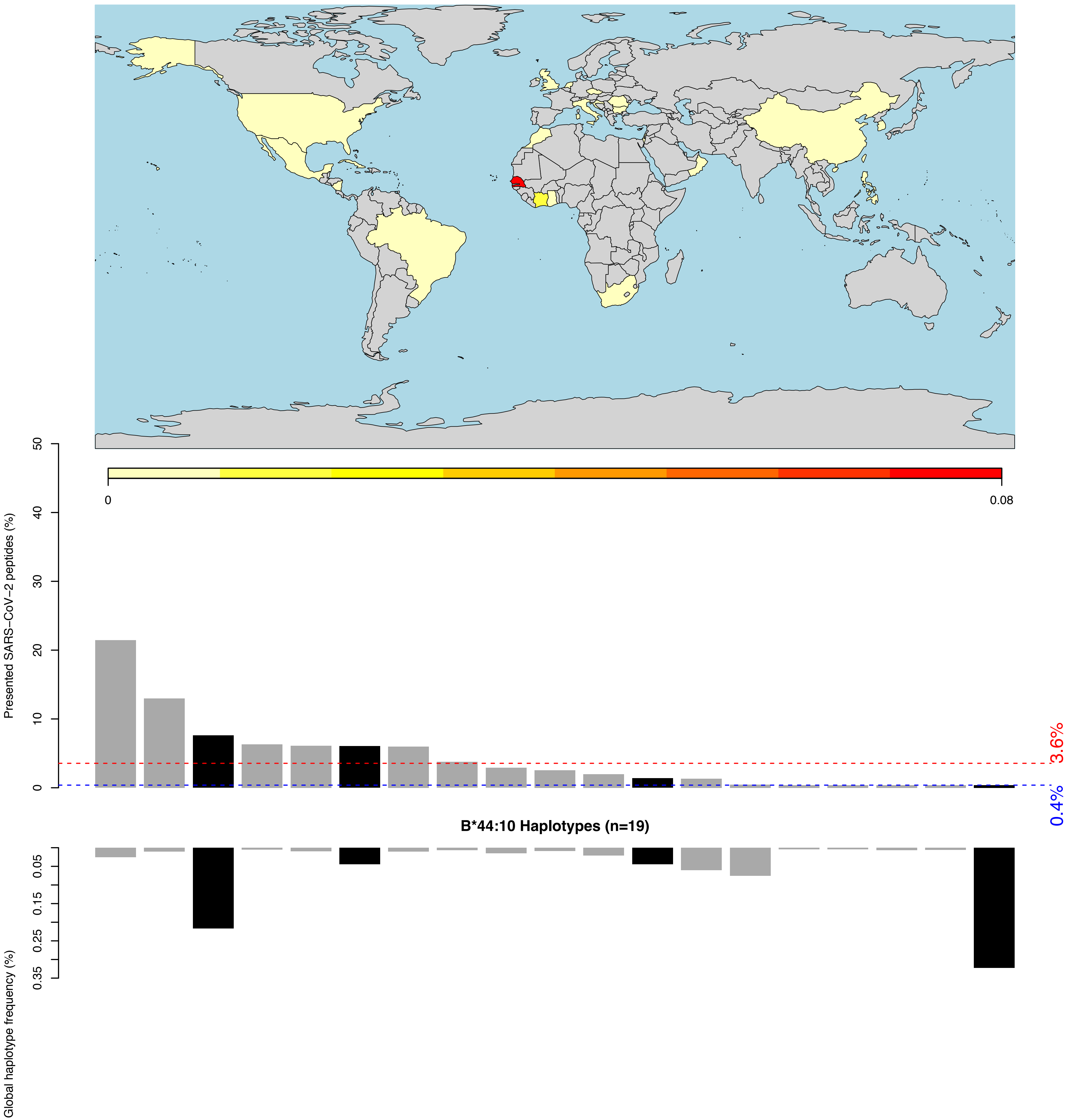

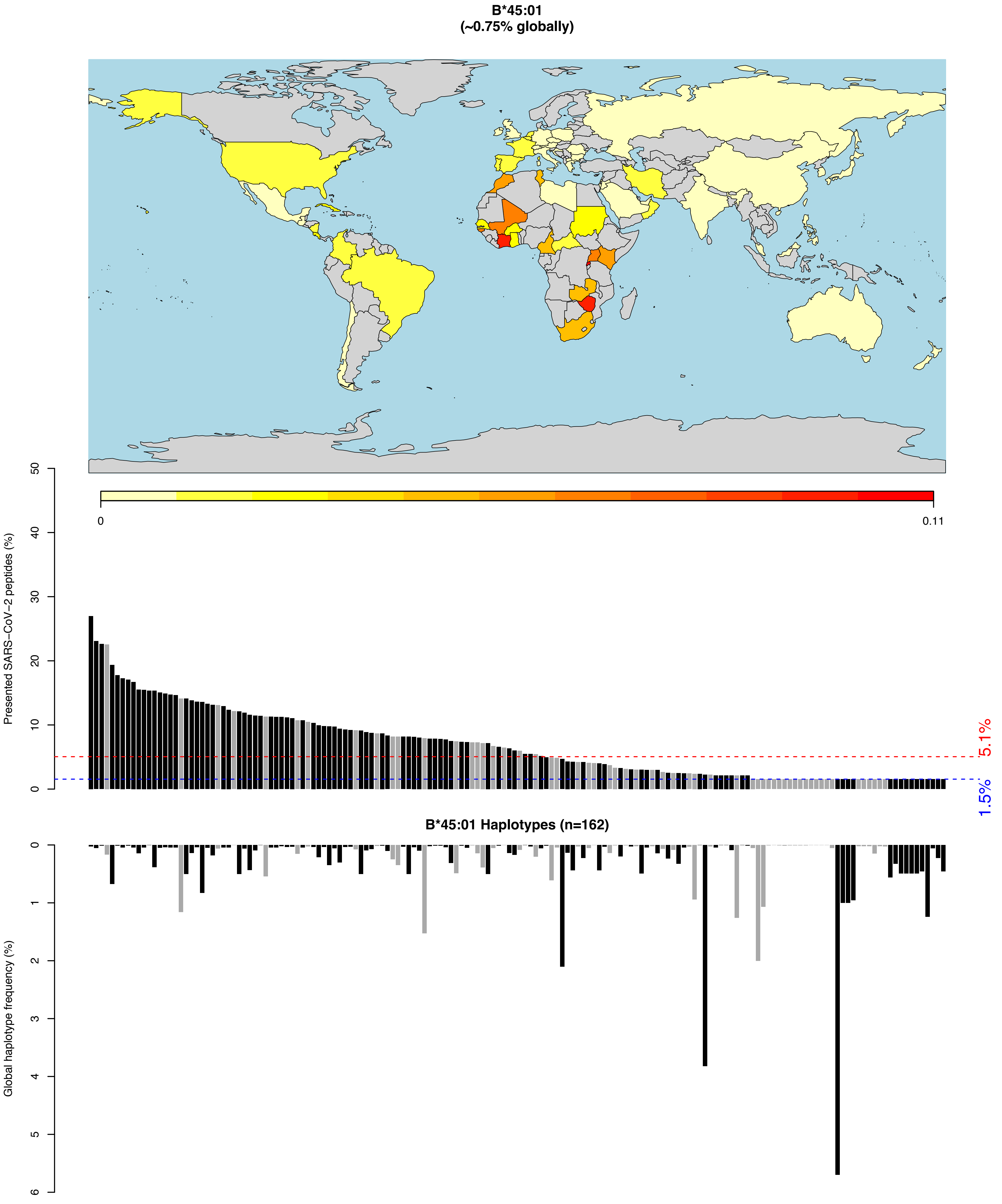

B*45:01
(~0.75% globally)
0
0.11
Presented SARS−CoV−2 peptides (%)
5.1%
1.5%
B*45:01 Haplotypes (n=162)
Global haplotype frequency (%)

B*46:01
(~6.1% globally)

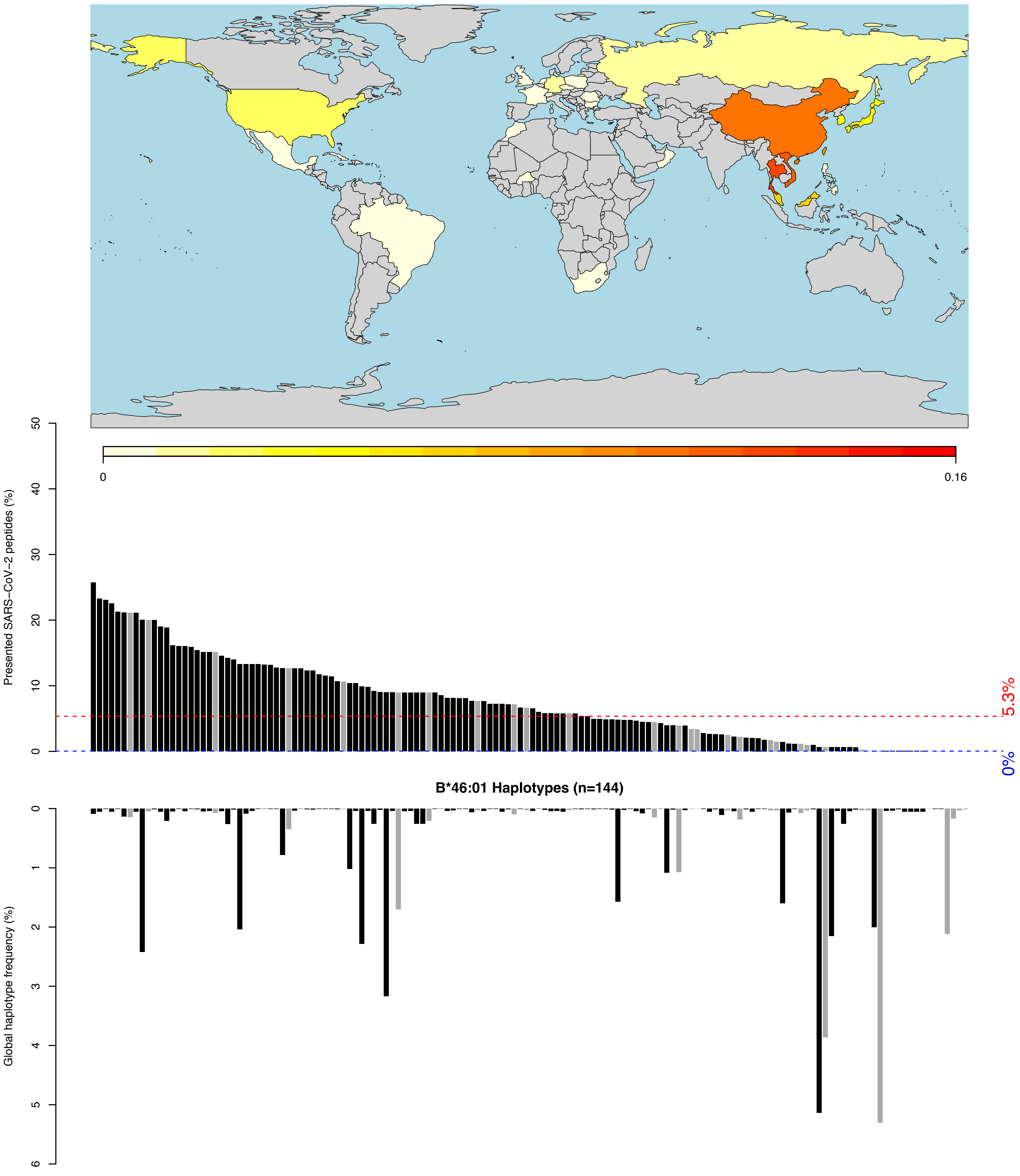

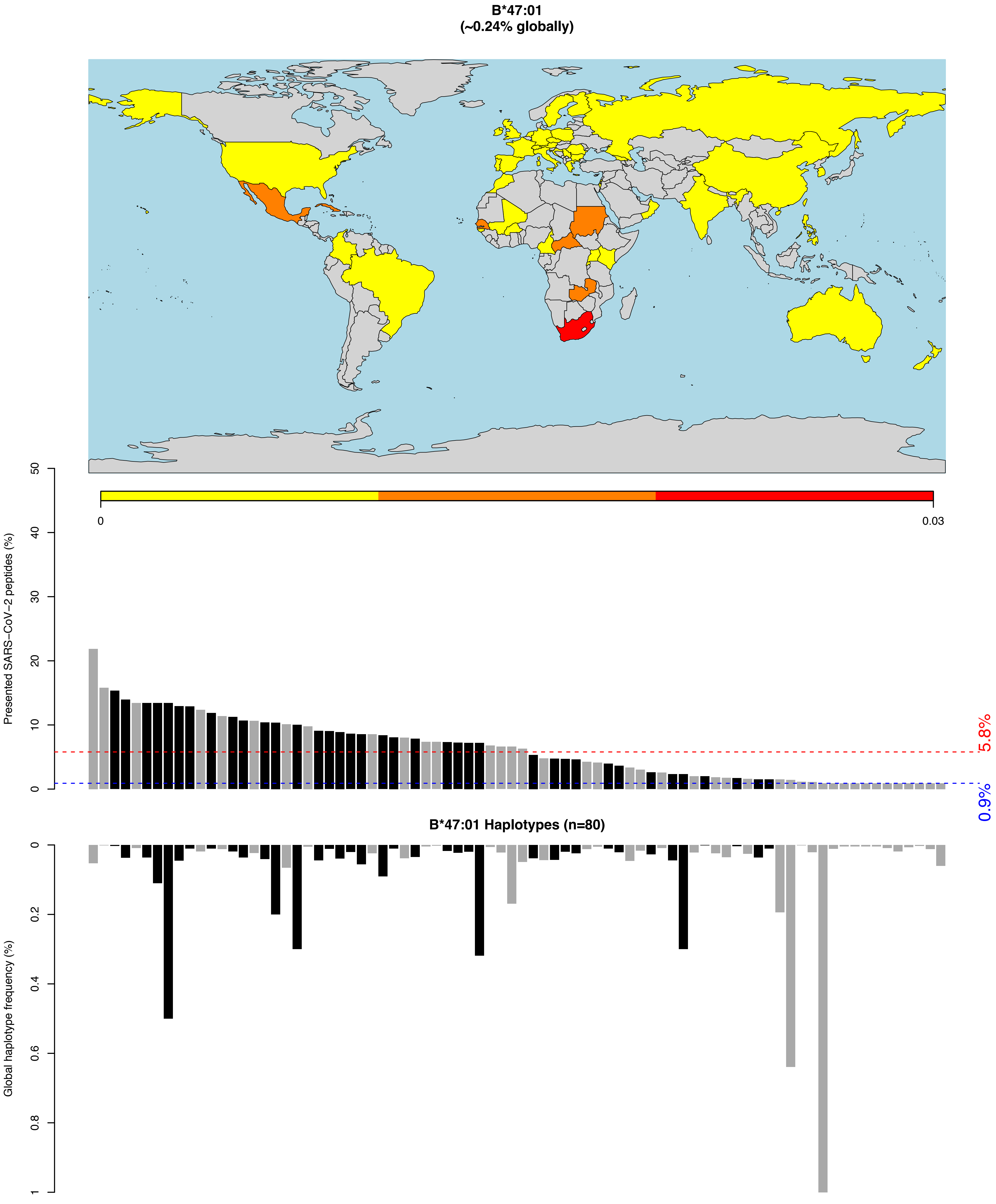
B*47:01
(~0.24% globally)
0
0.03
Presented SARS−CoV−2 peptides (%)
50
40
30
20
10
0
5.8%
0.9%
B*47:01 Haplotypes (n=80)
Global haplotype frequency (%)
0
0.2
0.4
0.6
0.8
1

B*47:03
(~0.069% globally)

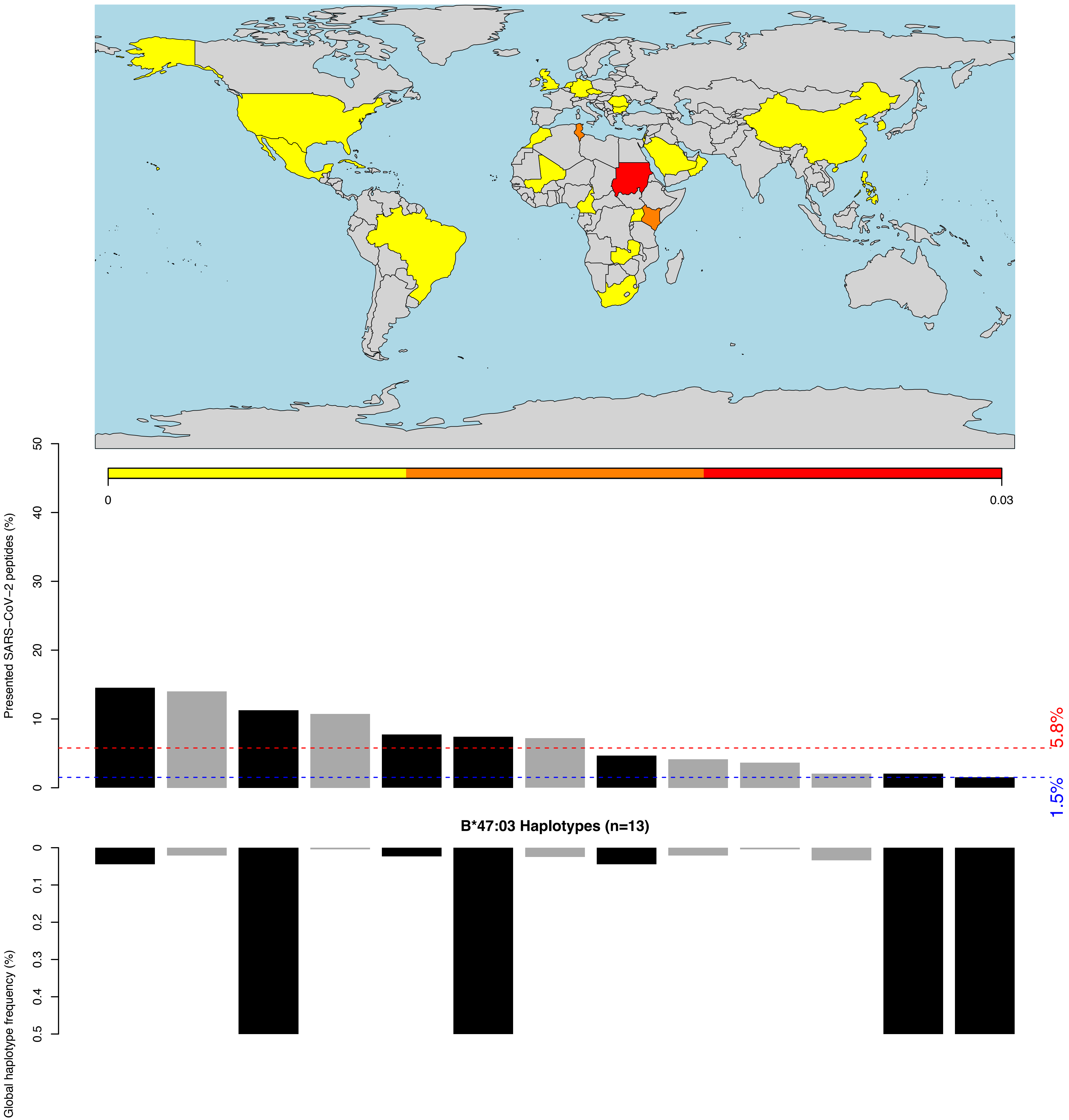

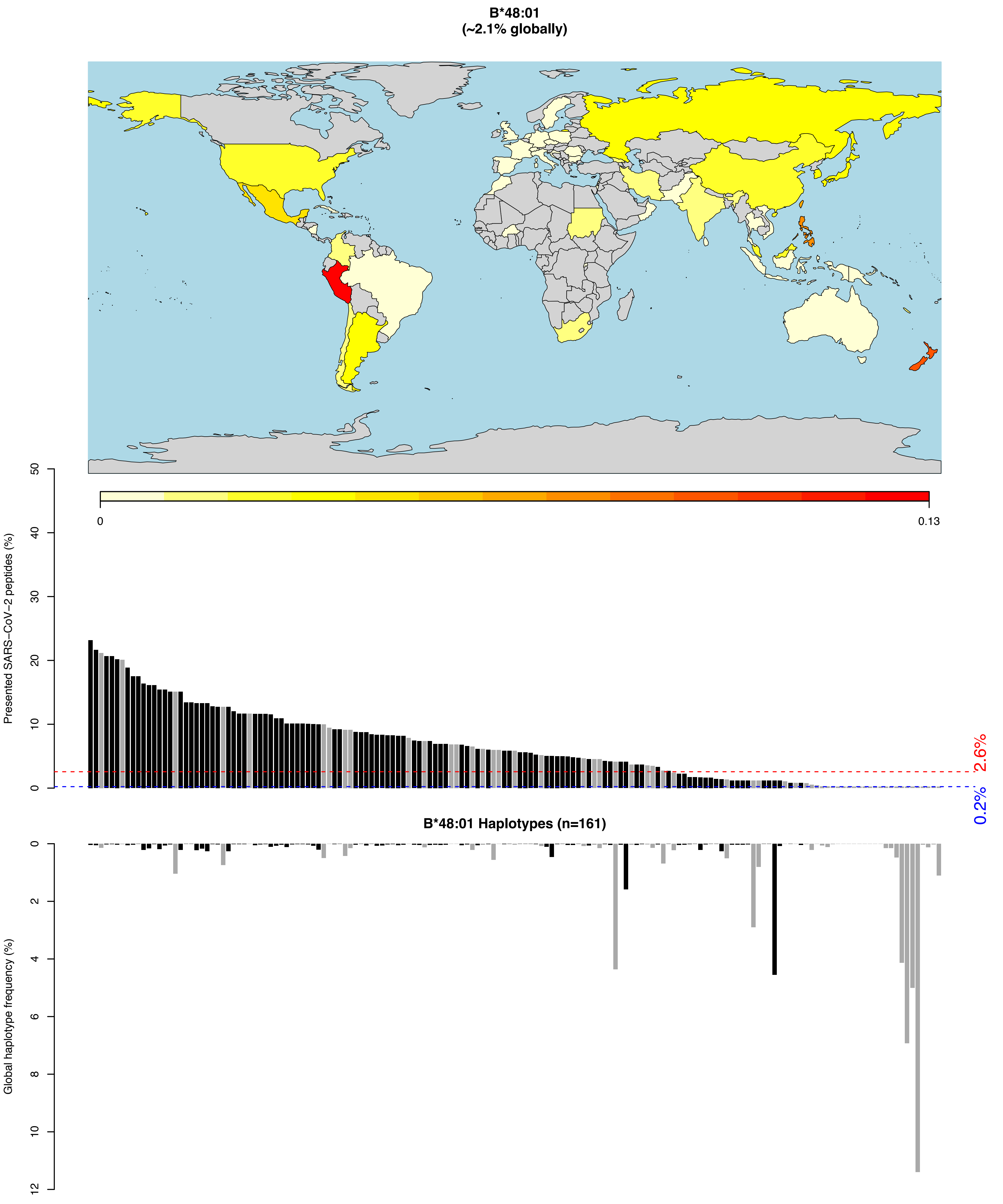

B*48:01
(~2.1% globally)
0
0.13
Presented SARS−CoV−2 peptides (%)
50
40
30
20
10
0
2.6%
0.2%
B*48:01 Haplotypes (n=161)
Global haplotype frequency (%)
0
2
4
6
8
10
12

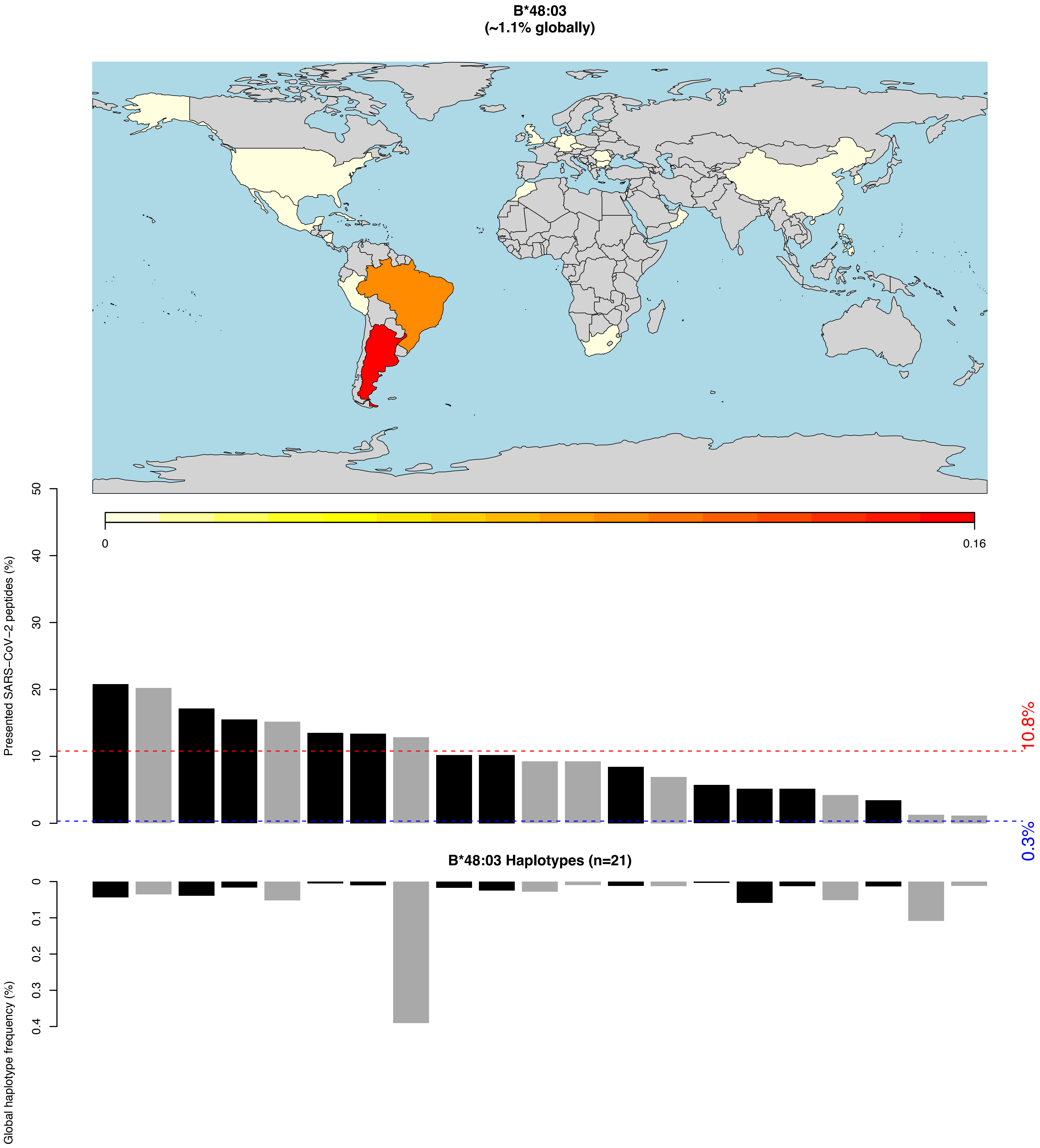
B*48:03
(~1.1% globally)
0
0.16
Presented SARS−CoV−2 peptides (%)
50
40
30
20
10
0
10.8%
0.3%
B*48:03 Haplotypes (n=21)
Global haplotype frequency (%)
0
0.1
0.2
0.3
0.4

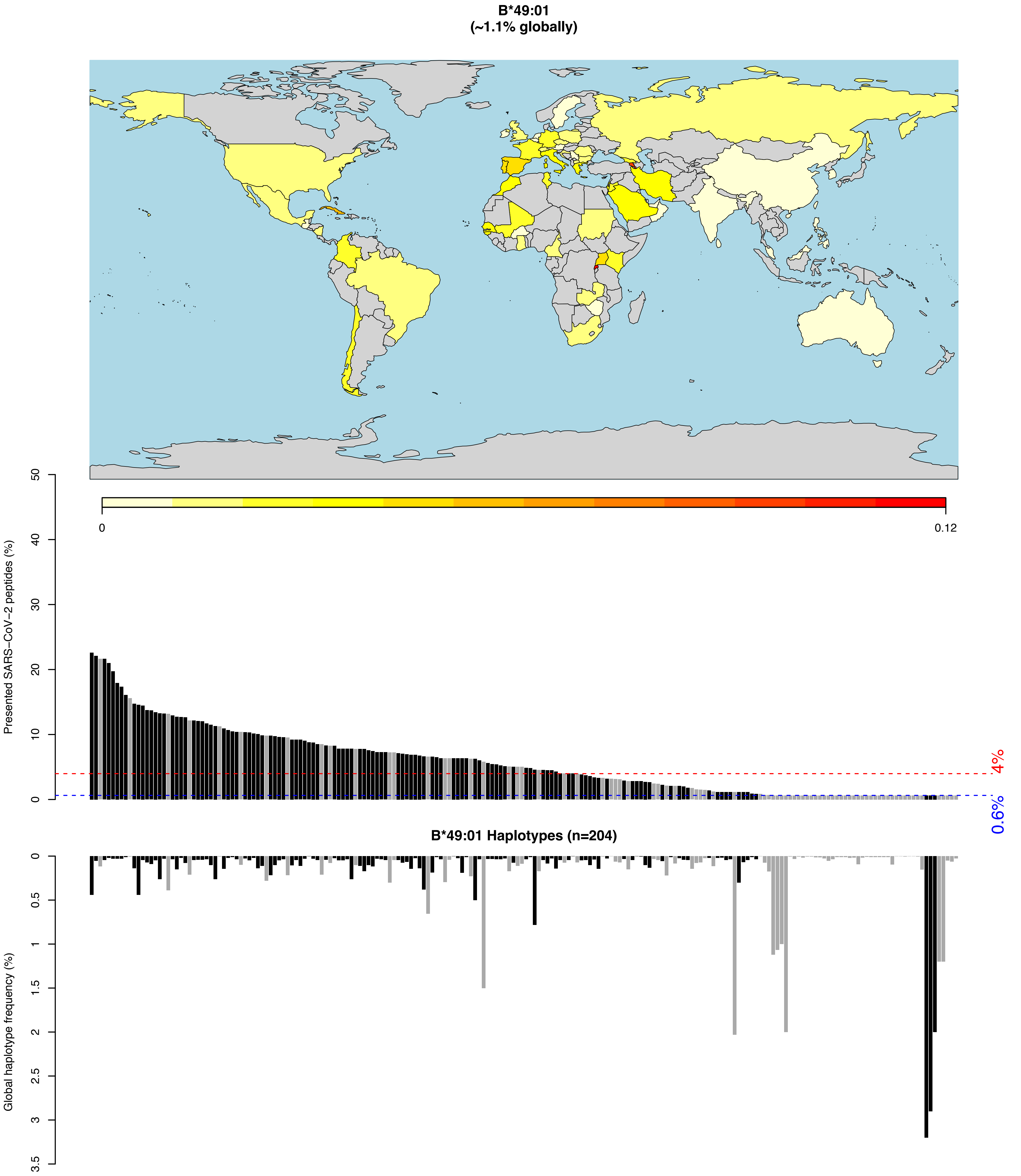

B*49:01
(~1.1% globally)
0
0.12
Presented SARS−CoV−2 peptides (%)
4%
0.6%
B*49:01 Haplotypes (n=204)
Global haplotype frequency (%)

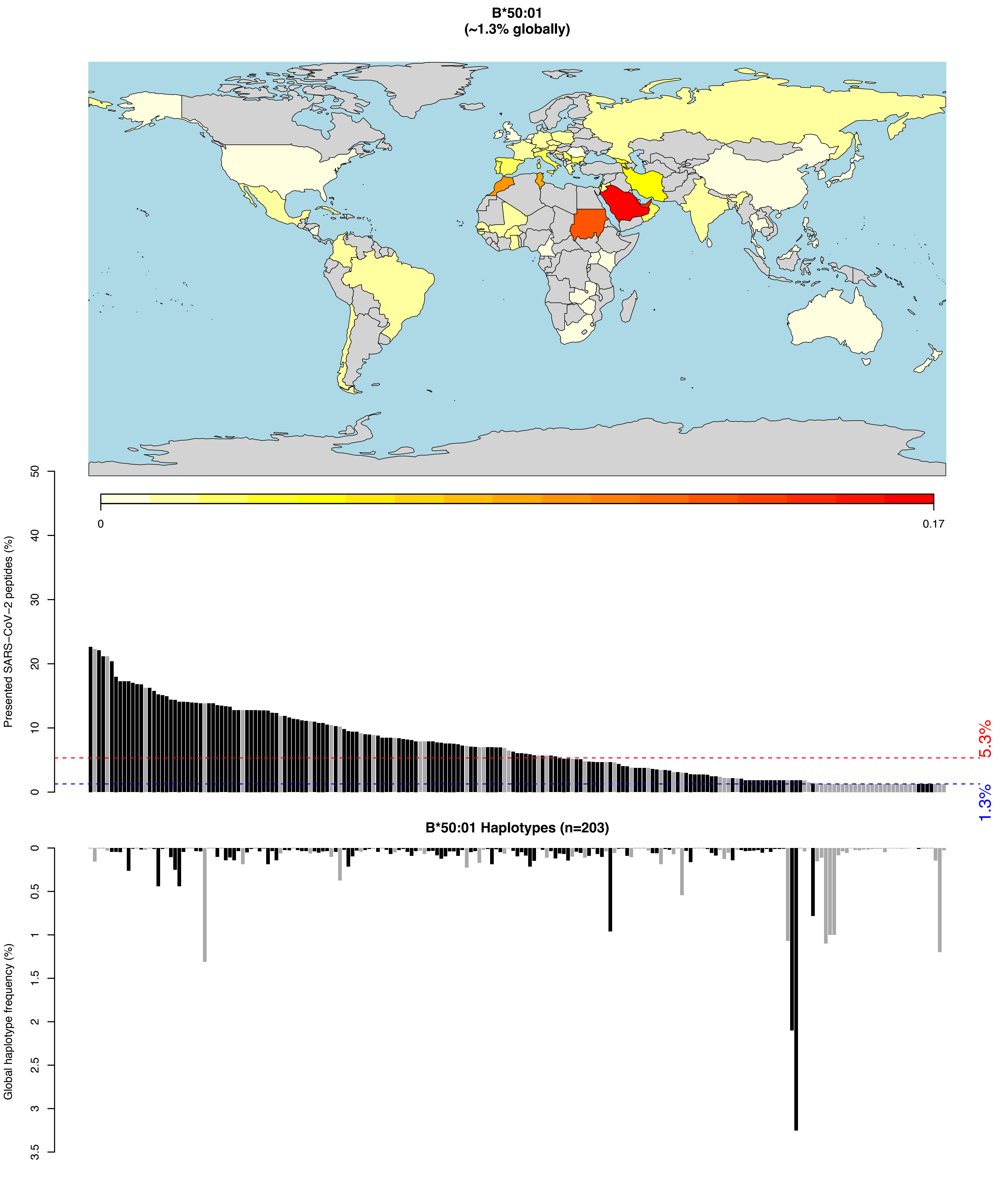

B*50:01
(~1.3% globally)
0
0.17
Presented SARS−CoV−2 peptides (%)
5.3%
1.3%
B*50:01 Haplotypes (n=203)
Global haplotype frequency (%)

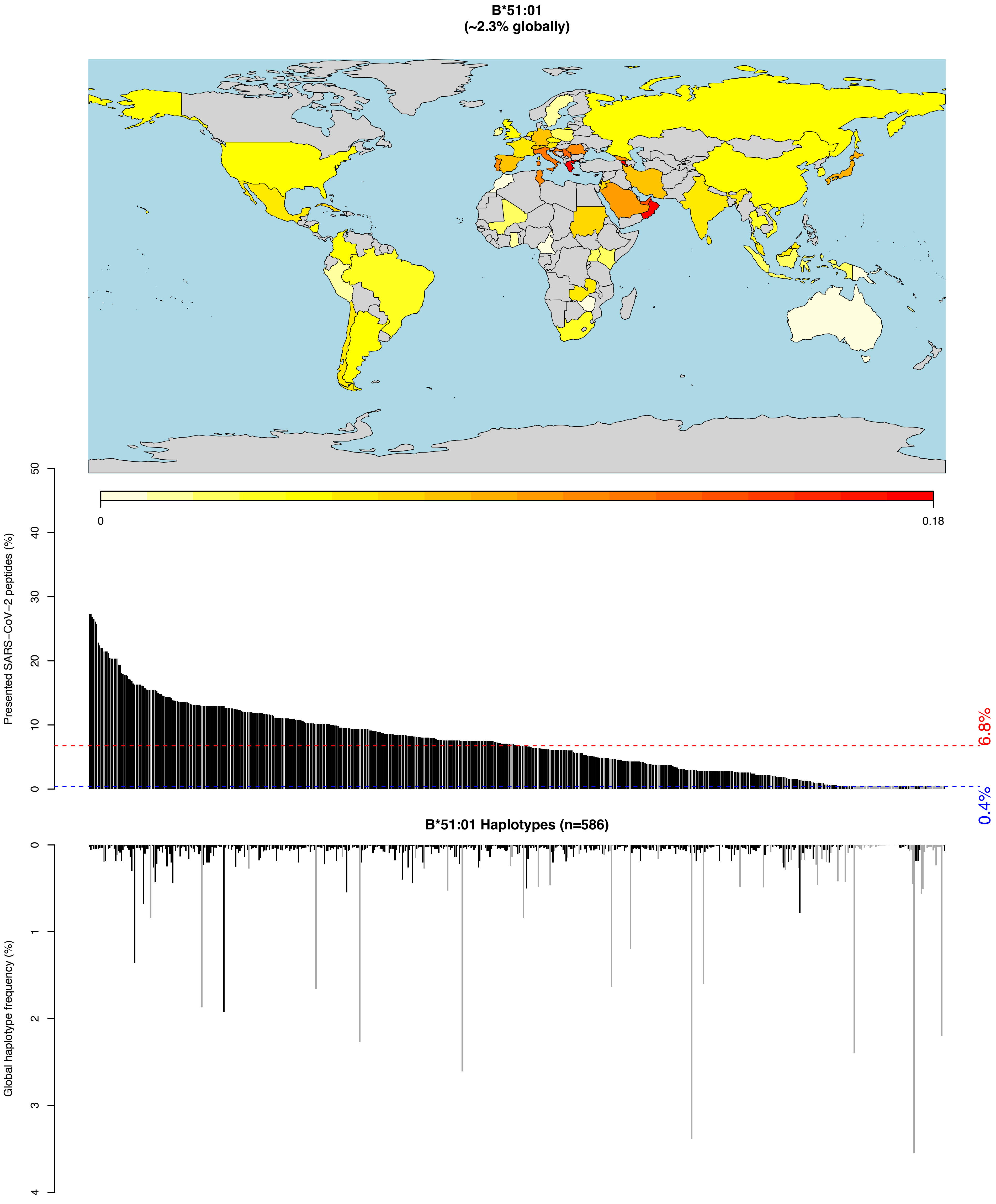
B*51:01
(~2.3% globally)
0
0.18
Presented SARS−CoV−2 peptides (%)
50
40
30
20
10
0
6.8%
0.4%
B*51:01 Haplotypes (n=586)
Global haplotype frequency (%)
0
1
2
3
4

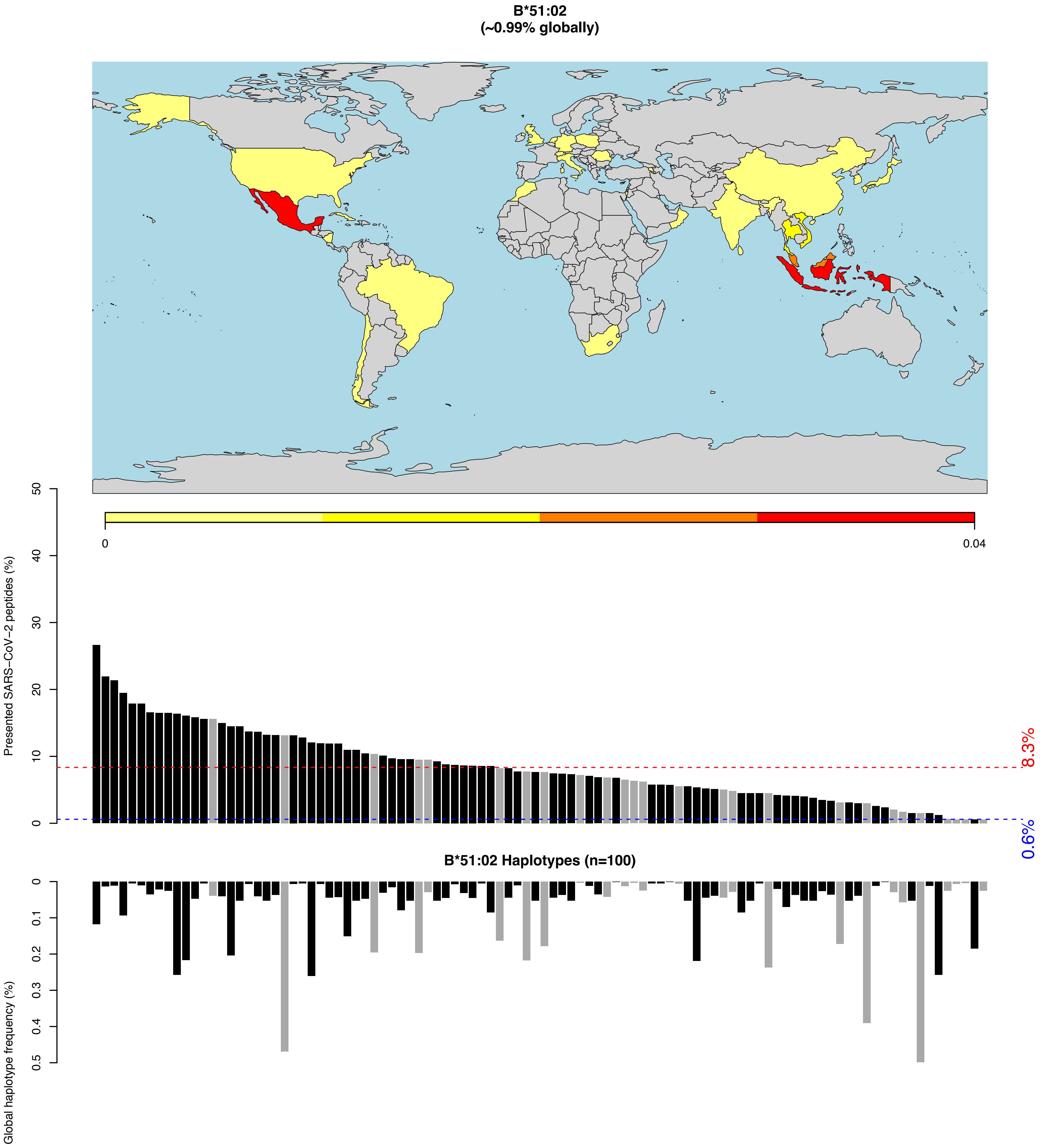
B*51:02
(~0.99% globally)
0
0.04
Presented SARS−CoV−2 peptides (%)
50
40
30
20
10
0
8.3%
0.6%
B*51:02 Haplotypes (n=100)
Global haplotype frequency (%)
0
0.1
0.2
0.3
0.4
0.5

B*51:07
(~0.29% globally)

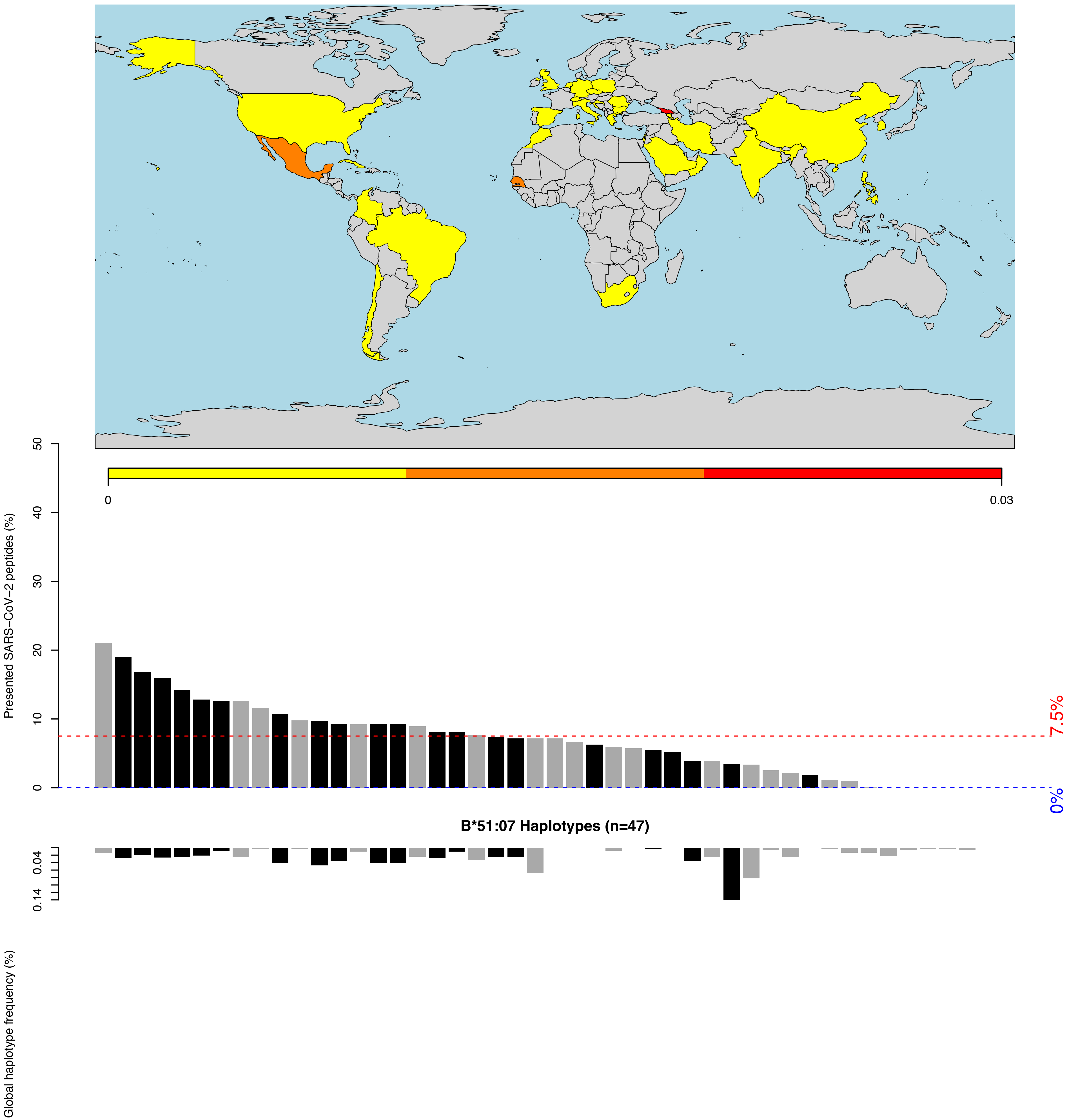

B*51:08
(~0.13% globally)

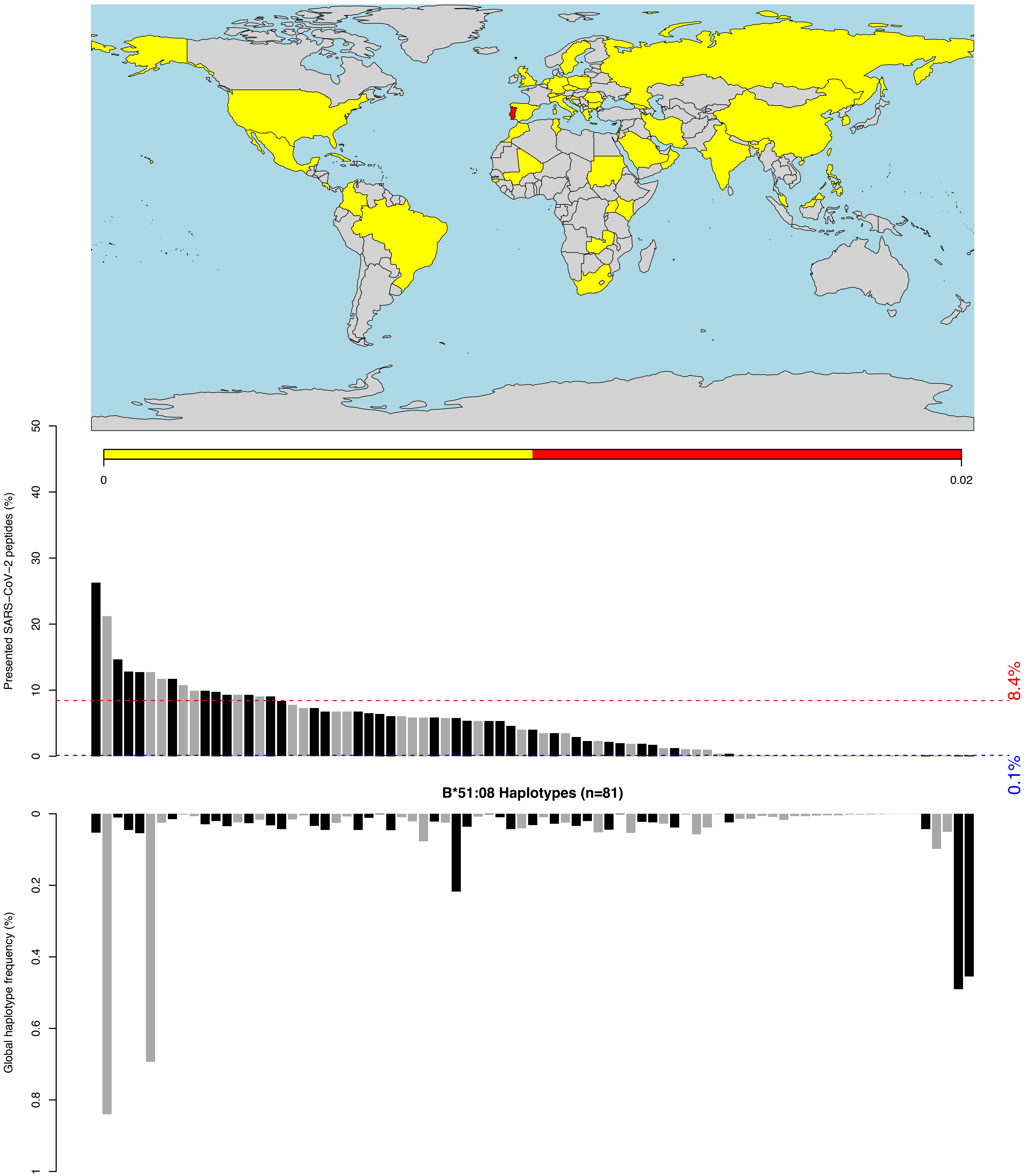

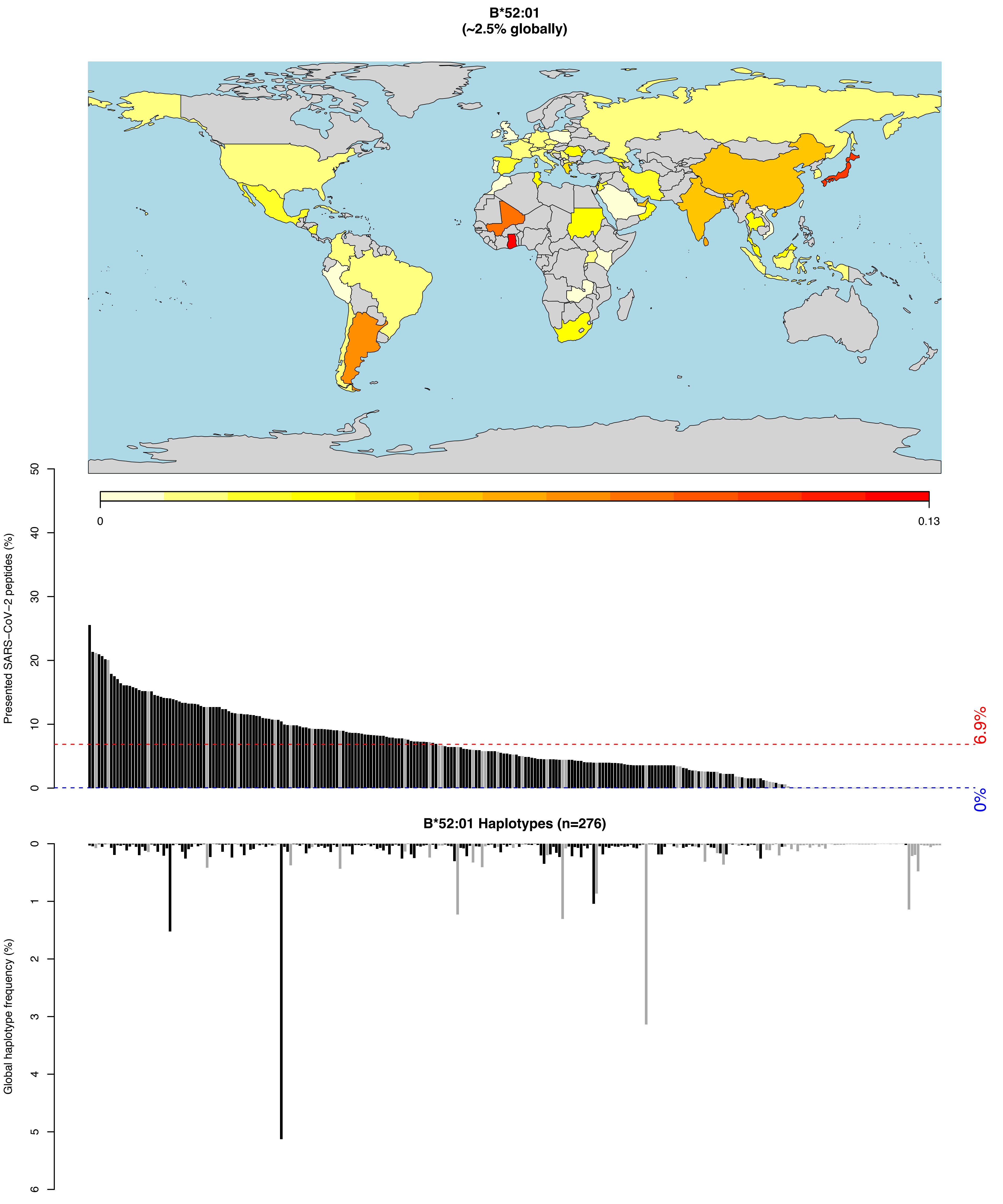

B*52:01
(~2.5% globally)
0
0.13
Presented SARS−CoV−2 peptides (%)
0
10
20
30
40
50
6.9%
0%
B*52:01 Haplotypes (n=276)
Global haplotype frequency (%)
0
1
2
3
4
5
6

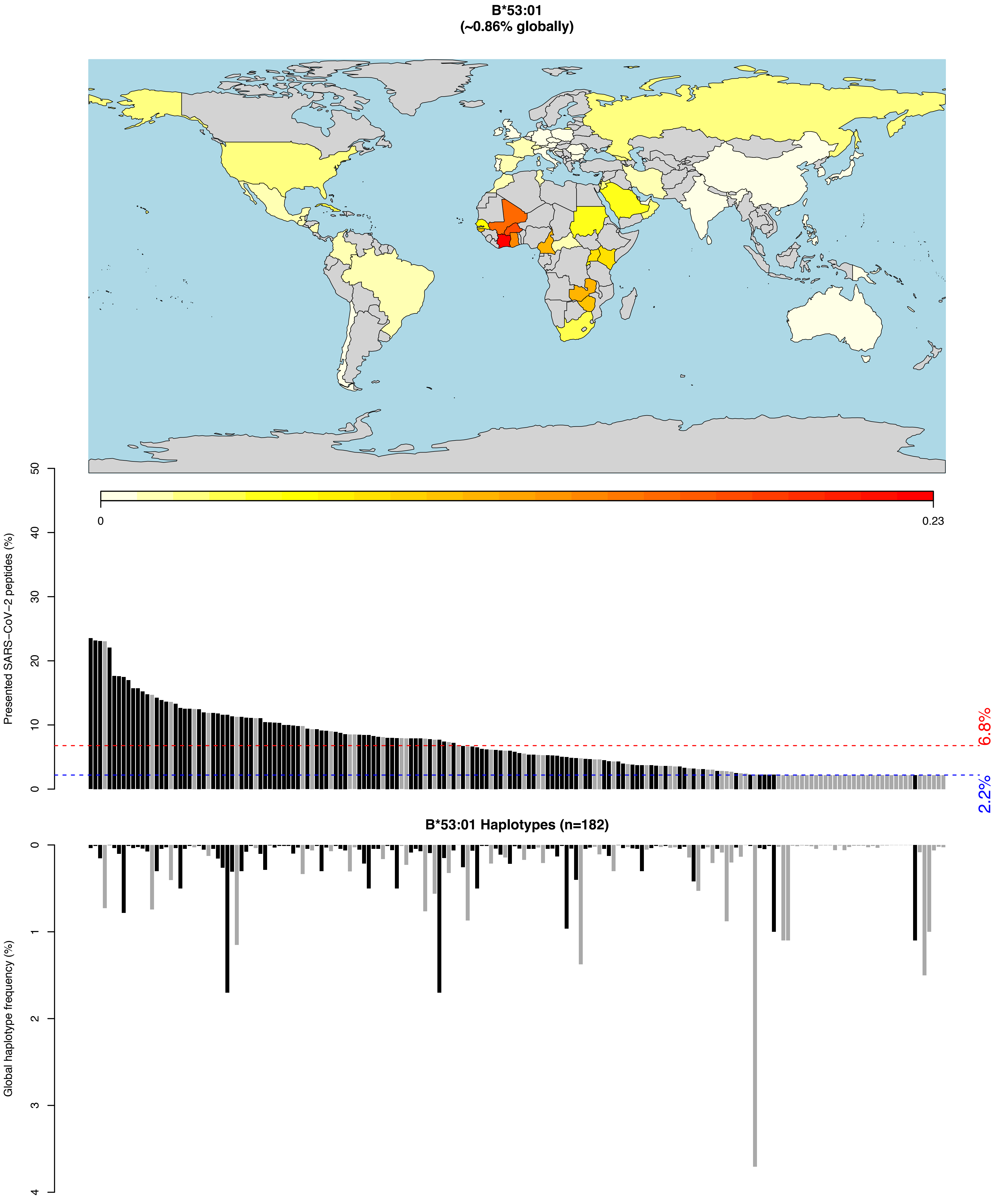
B*53:01
(~0.86% globally)
0
0.23
Presented SARS−CoV−2 peptides (%)
50
40
30
20
10
0
6.8%
2.2%
B*53:01 Haplotypes (n=182)
Global haplotype frequency (%)
0
1
2
3
4

B*54:01
(~1.4% globally)

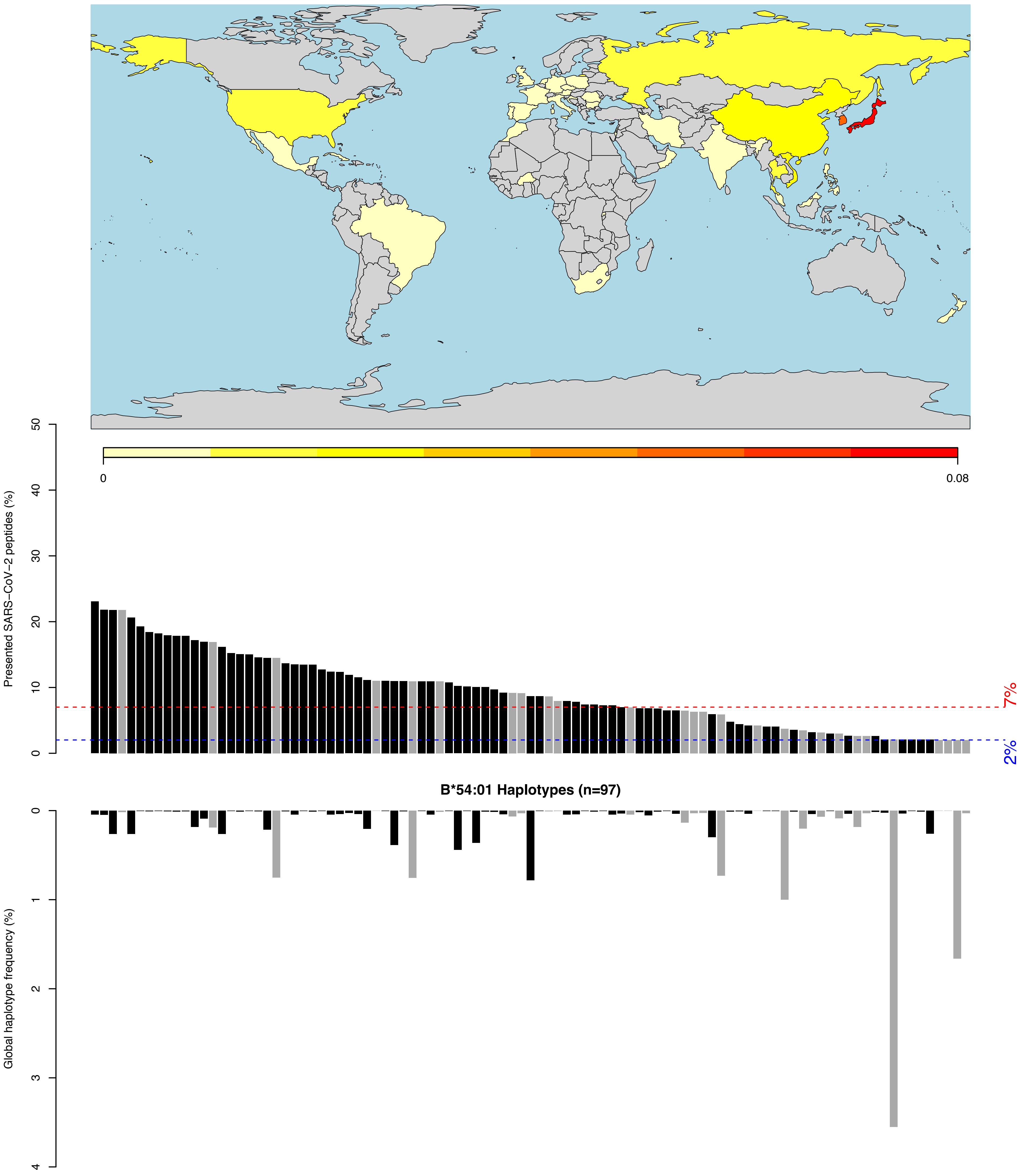

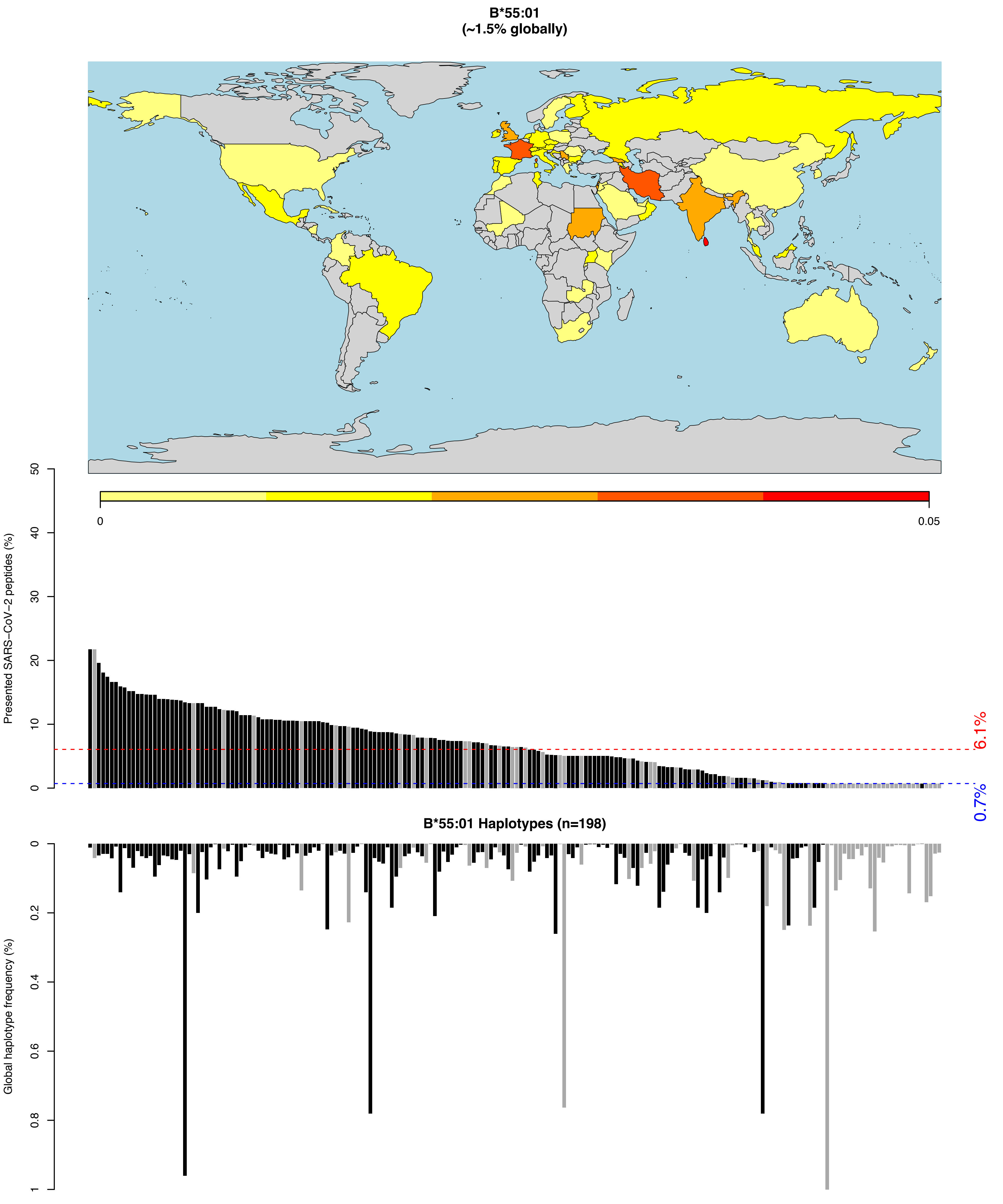

B*55:01
(~1.5% globally)
0
0.05
Presented SARS−CoV−2 peptides (%)
50
40
30
20
10
0
6.1%
0.7%
B*55:01 Haplotypes (n=198)
Global haplotype frequency (%)
0
0.2
0.4
0.6
0.8
1

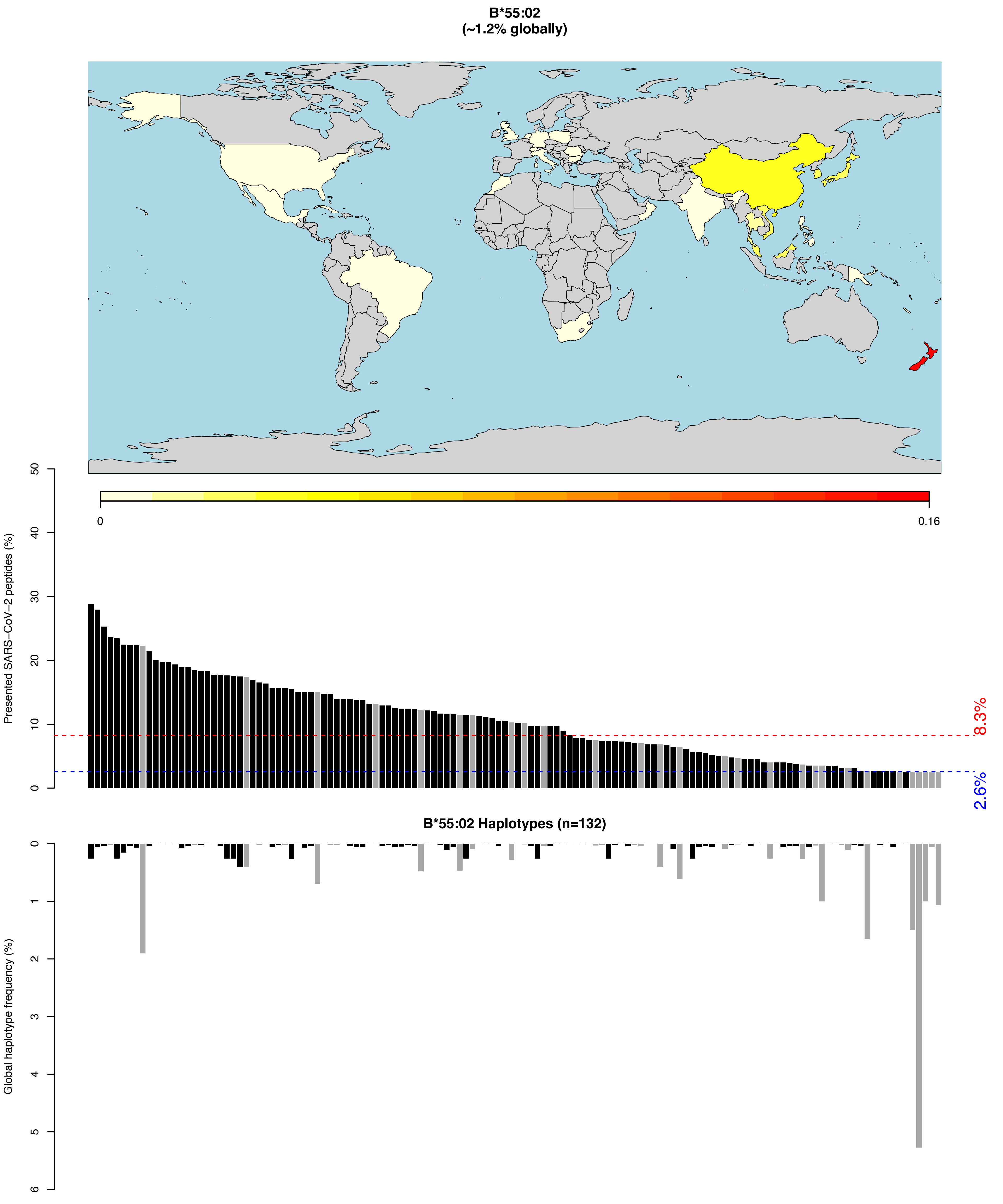

B*55:02
(~1.2% globally)
0
0.16
Presented SARS−CoV−2 peptides (%)
8.3%
2.6%
B*55:02 Haplotypes (n=132)
Global haplotype frequency (%)

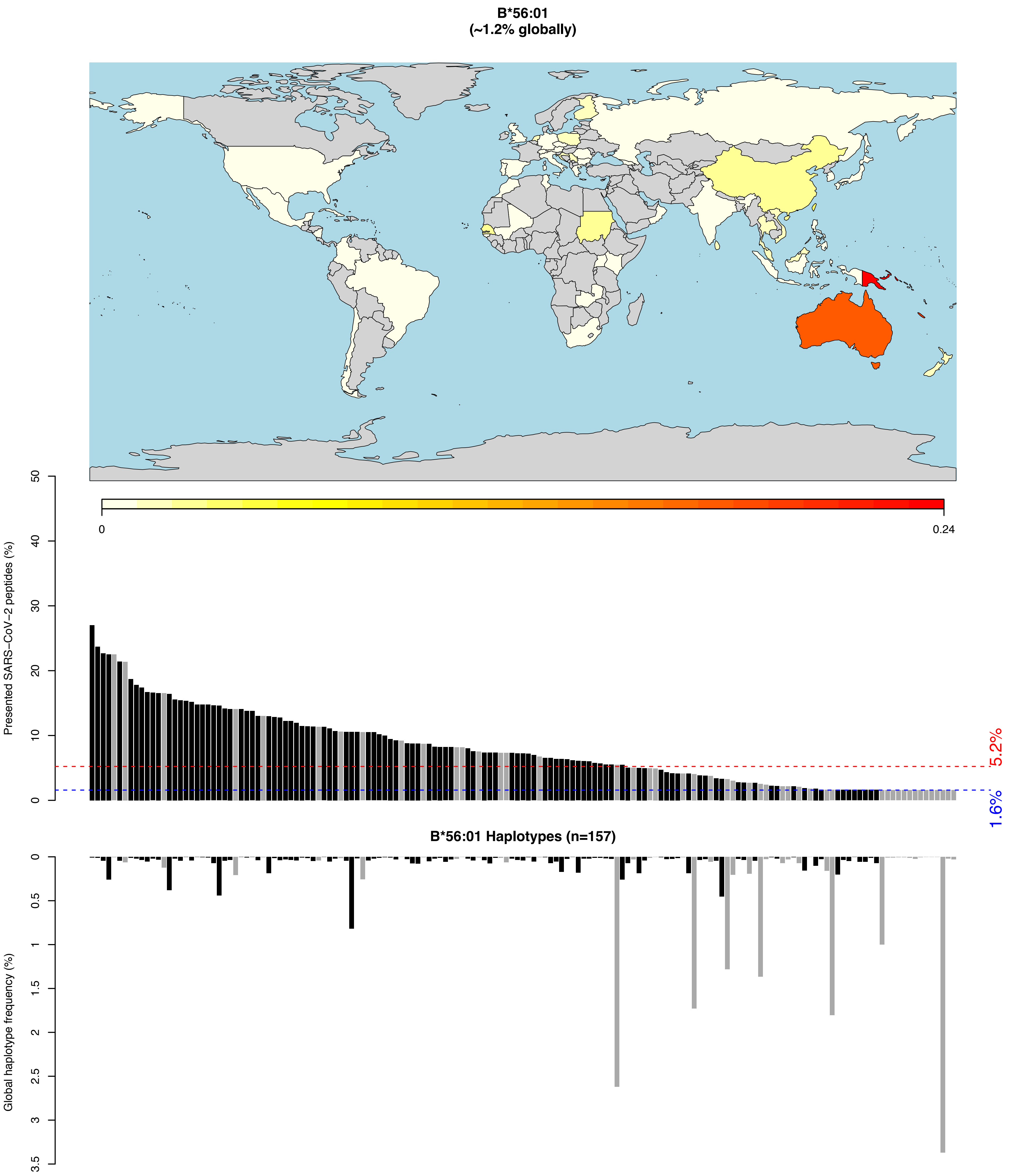

B*56:01
(~1.2% globally)
0
0.24
Presented SARS−CoV−2 peptides (%)
5.2%
1.6%
B*56:01 Haplotypes (n=157)
Global haplotype frequency (%)

B*57:01
(~2.3% globally)

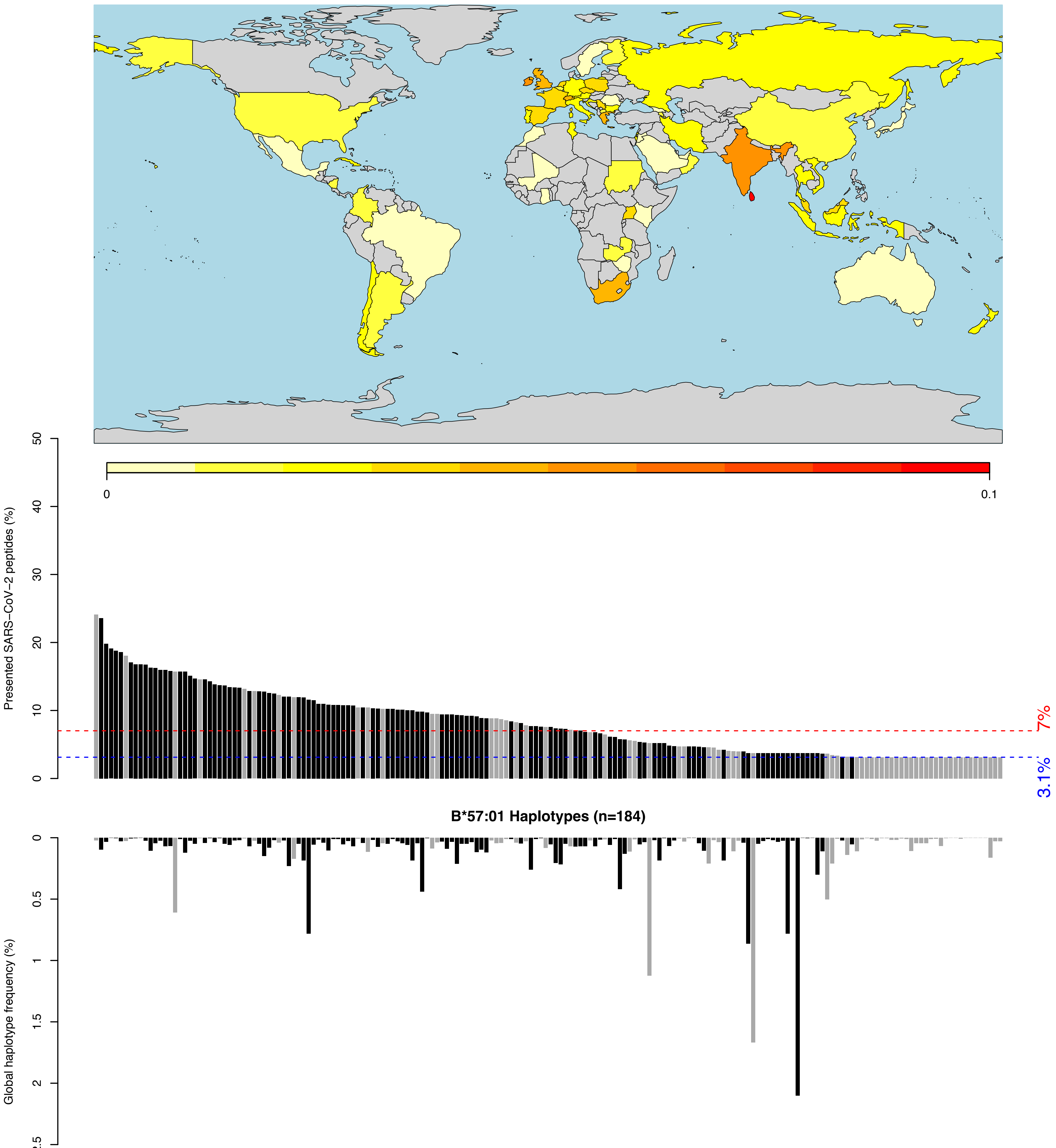

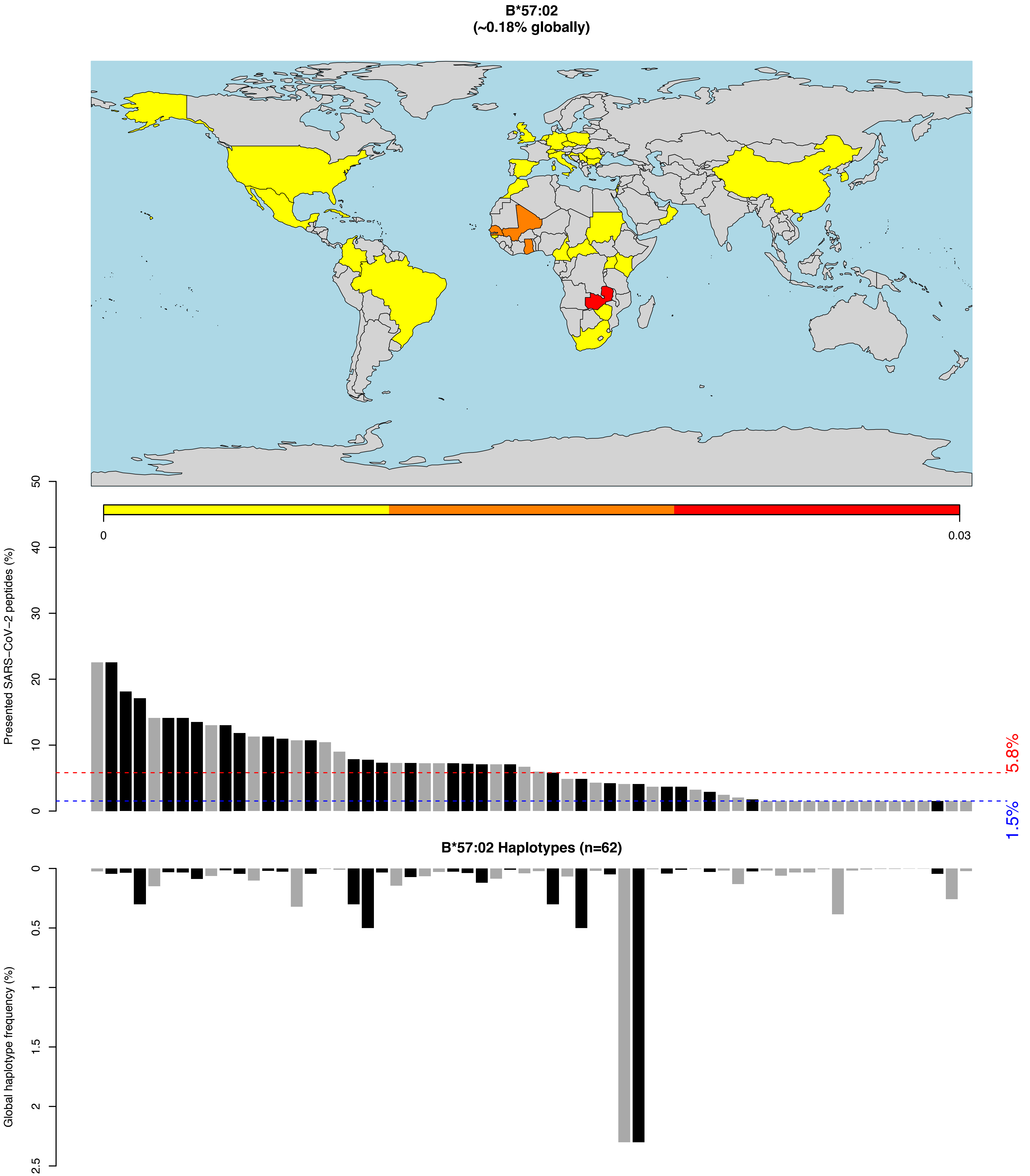

B*57:02
(~0.18% globally)
0
0.03
Presented SARS−CoV−2 peptides (%)
5.8%
1.5%
B*57:02 Haplotypes (n=62)
Global haplotype frequency (%)

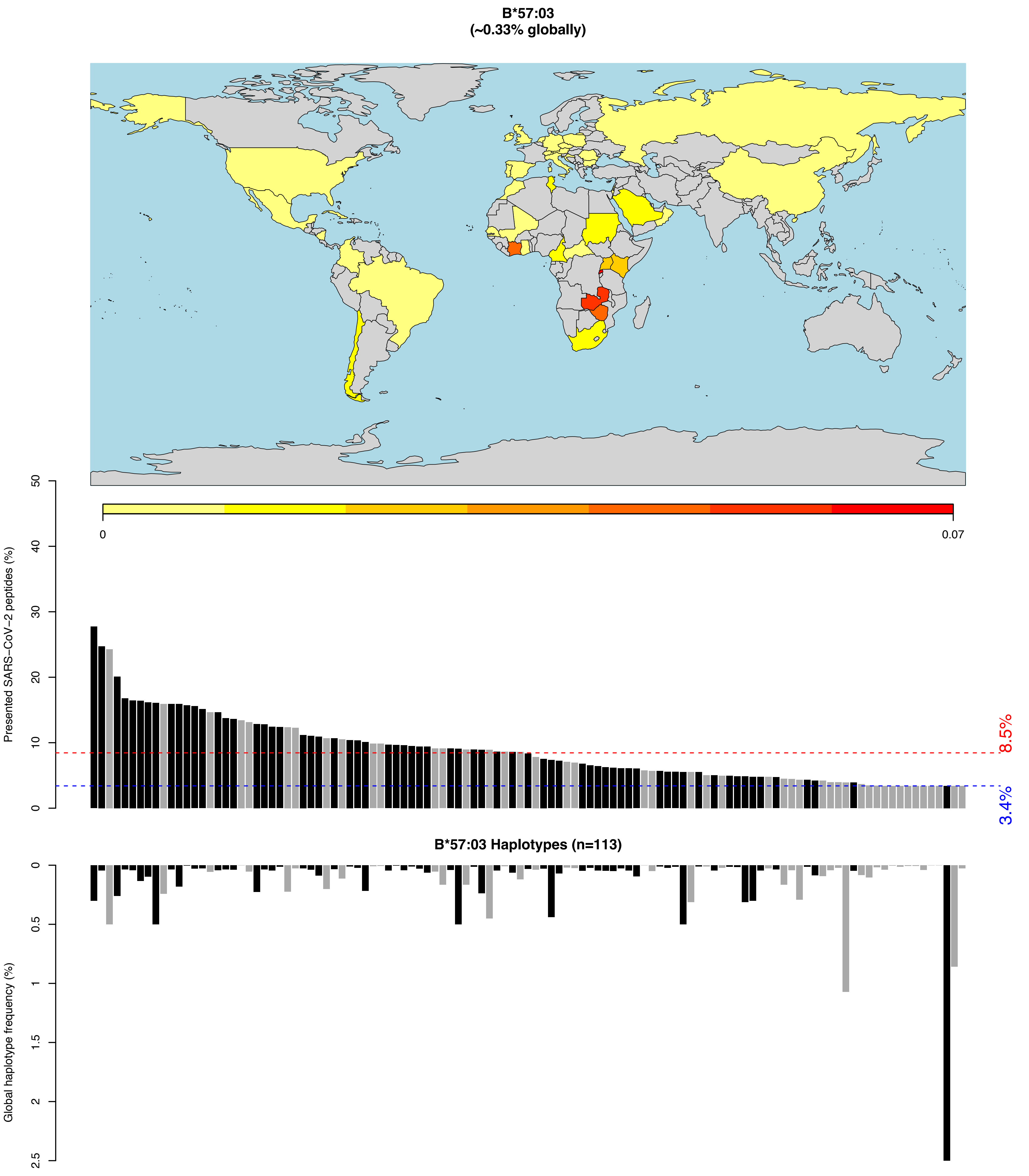

B*57:03
(~0.33% globally)
0
0.07
Presented SARS−CoV−2 peptides (%)
50
40
30
20
10
0
8.5%
3.4%
B*57:03 Haplotypes (n=113)
Global haplotype frequency (%)
0
0.5
1
1.5
2
2.5

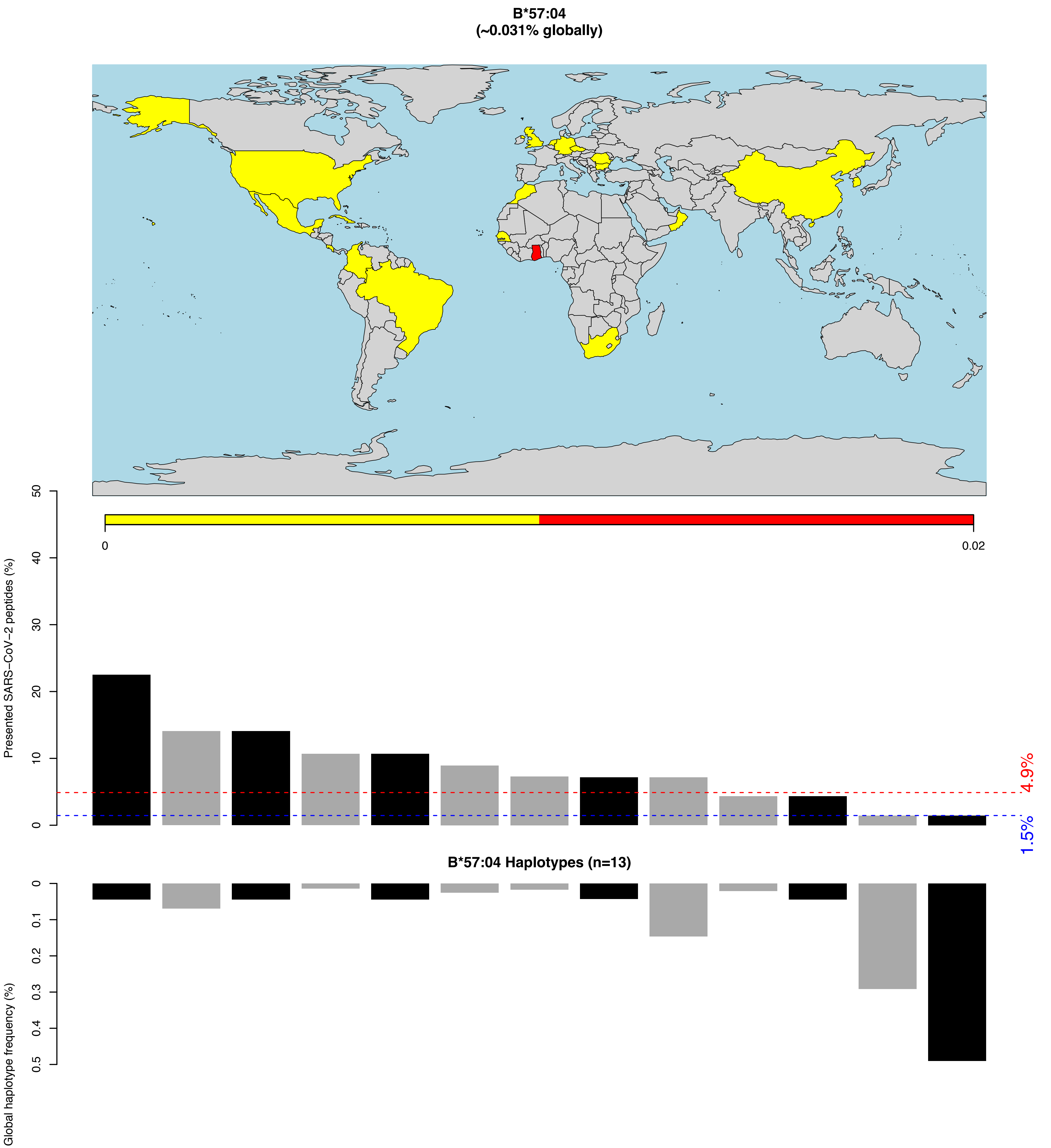
B*57:04
(~0.031% globally)
0
0.02
Presented SARS−CoV−2 peptides (%)
4.9%
1.5%
B*57:04 Haplotypes (n=13)
Global haplotype frequency (%)

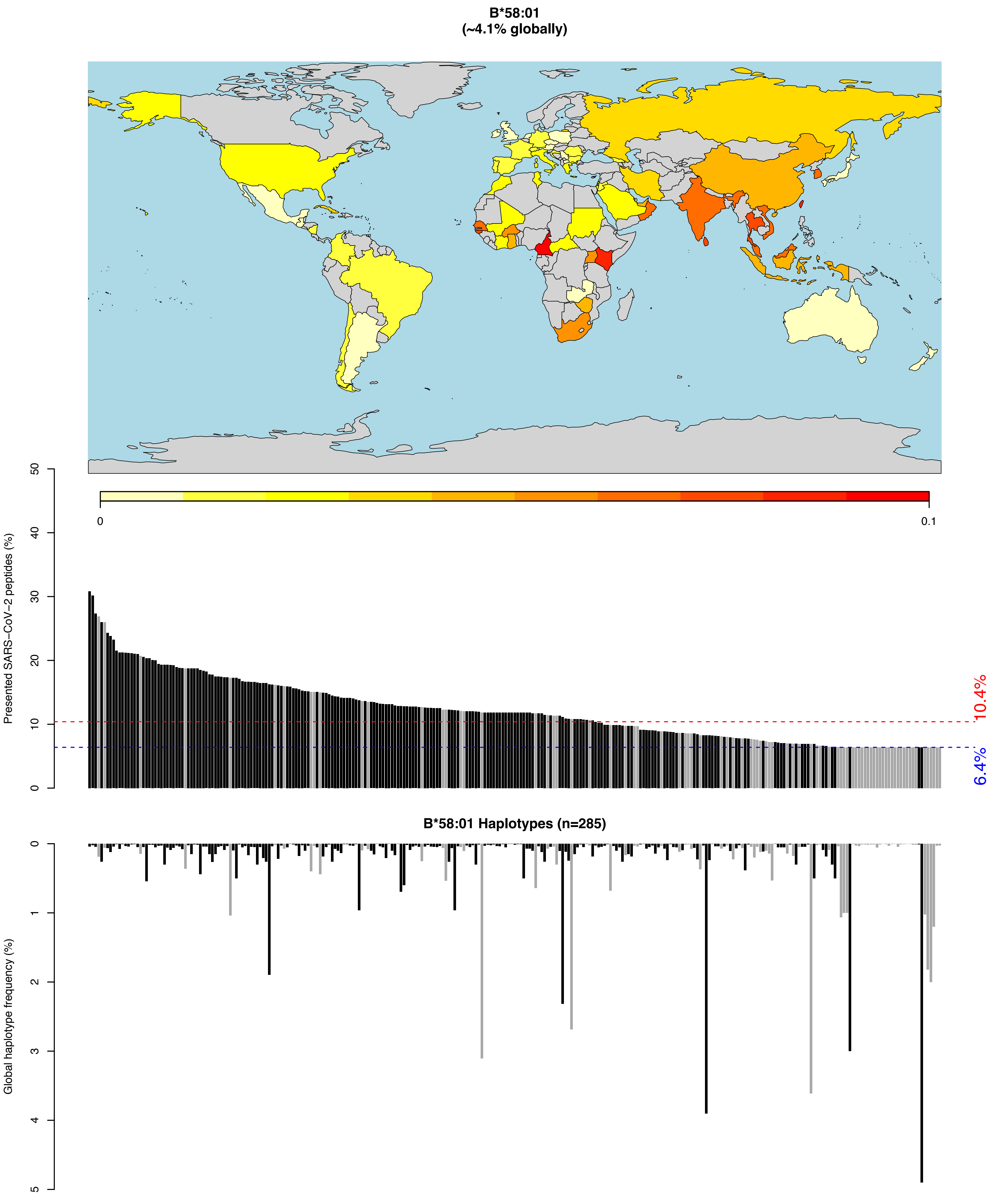

B*58:01
(~4.1% globally)
0
0.1
Presented SARS−CoV−2 peptides (%)
50
40
30
20
10
0
10.4%
6.4%
B*58:01 Haplotypes (n=285)
Global haplotype frequency (%)
0
1
2
3
4
5

B*58:02
(~0.91% globally)

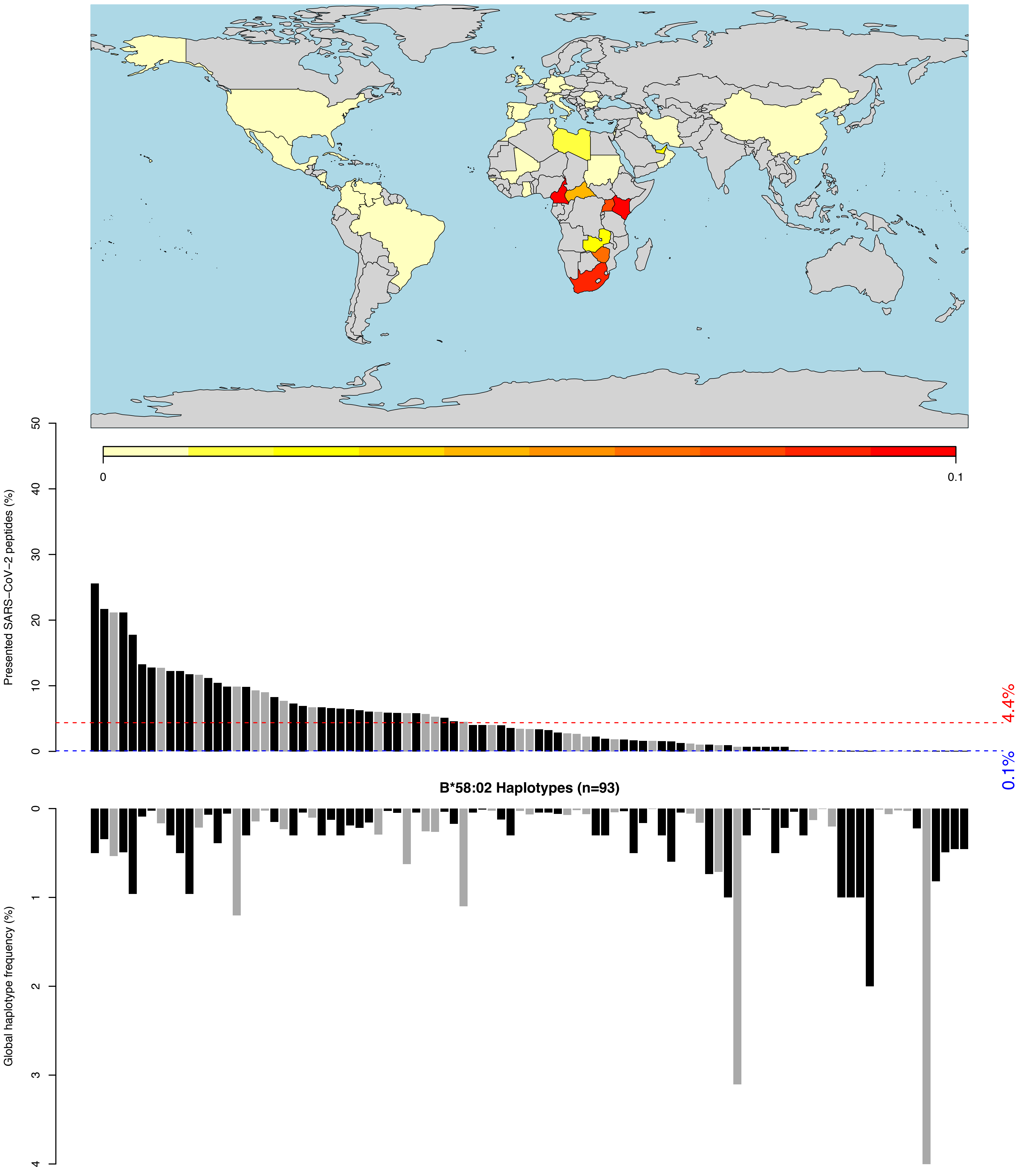

B*59:01
(~0.25% globally)

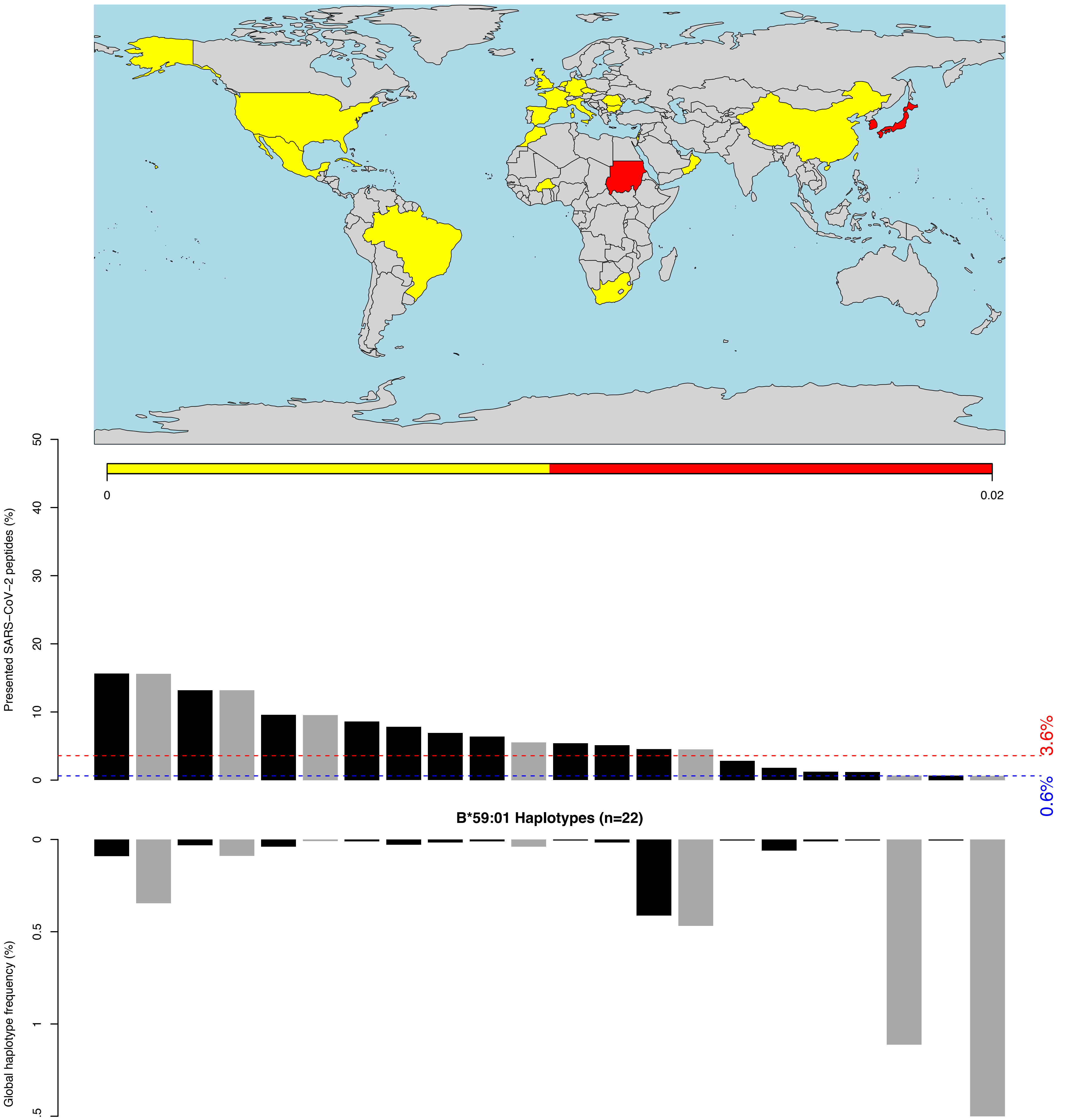

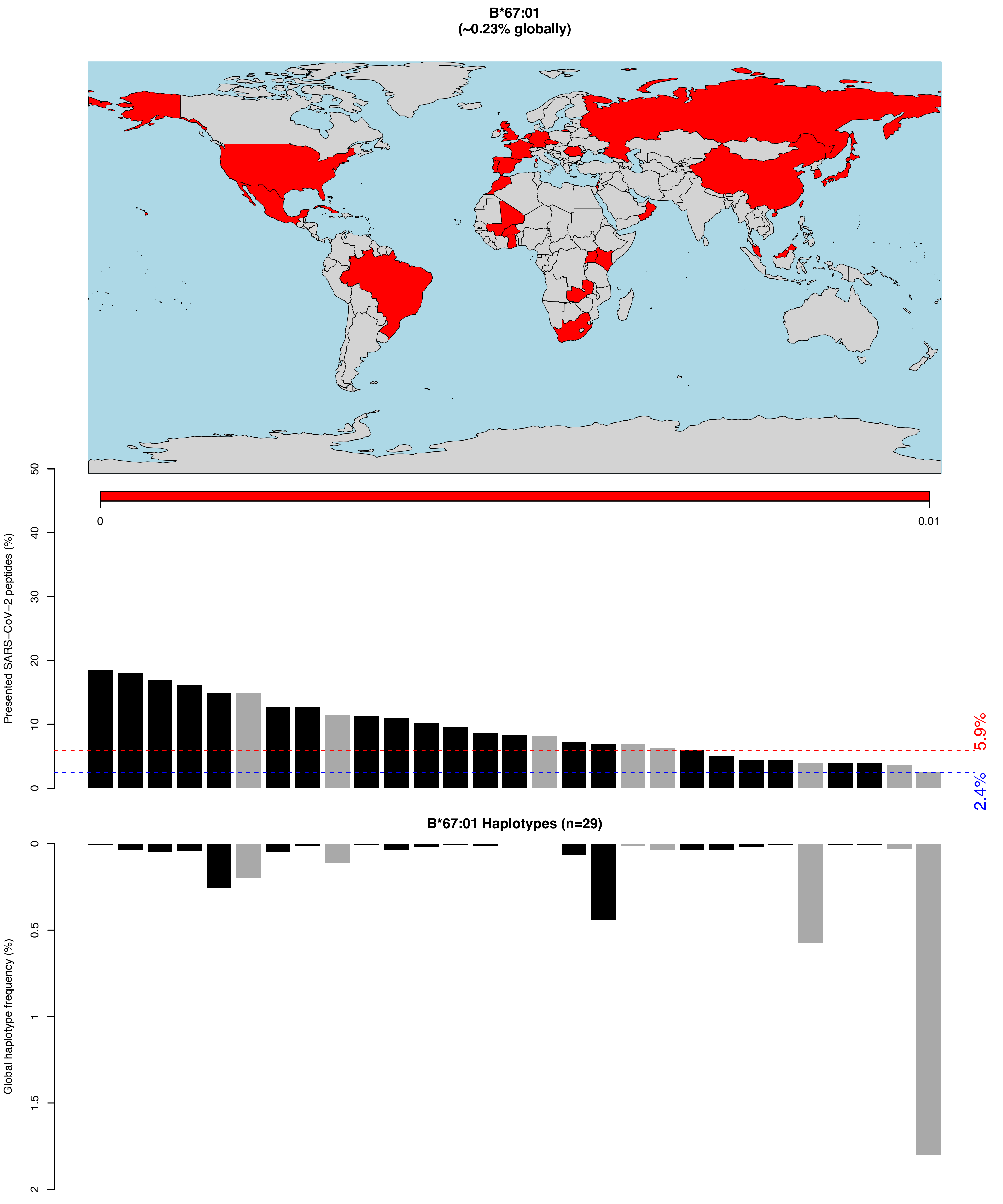

B*67:01
(~0.23% globally)
0
0.01
Presented SARS−CoV−2 peptides (%)
50
40
30
20
10
0
5.9%
2.4%
B*67:01 Haplotypes (n=29)
Global haplotype frequency (%)
0
0.5
1
1.5
2

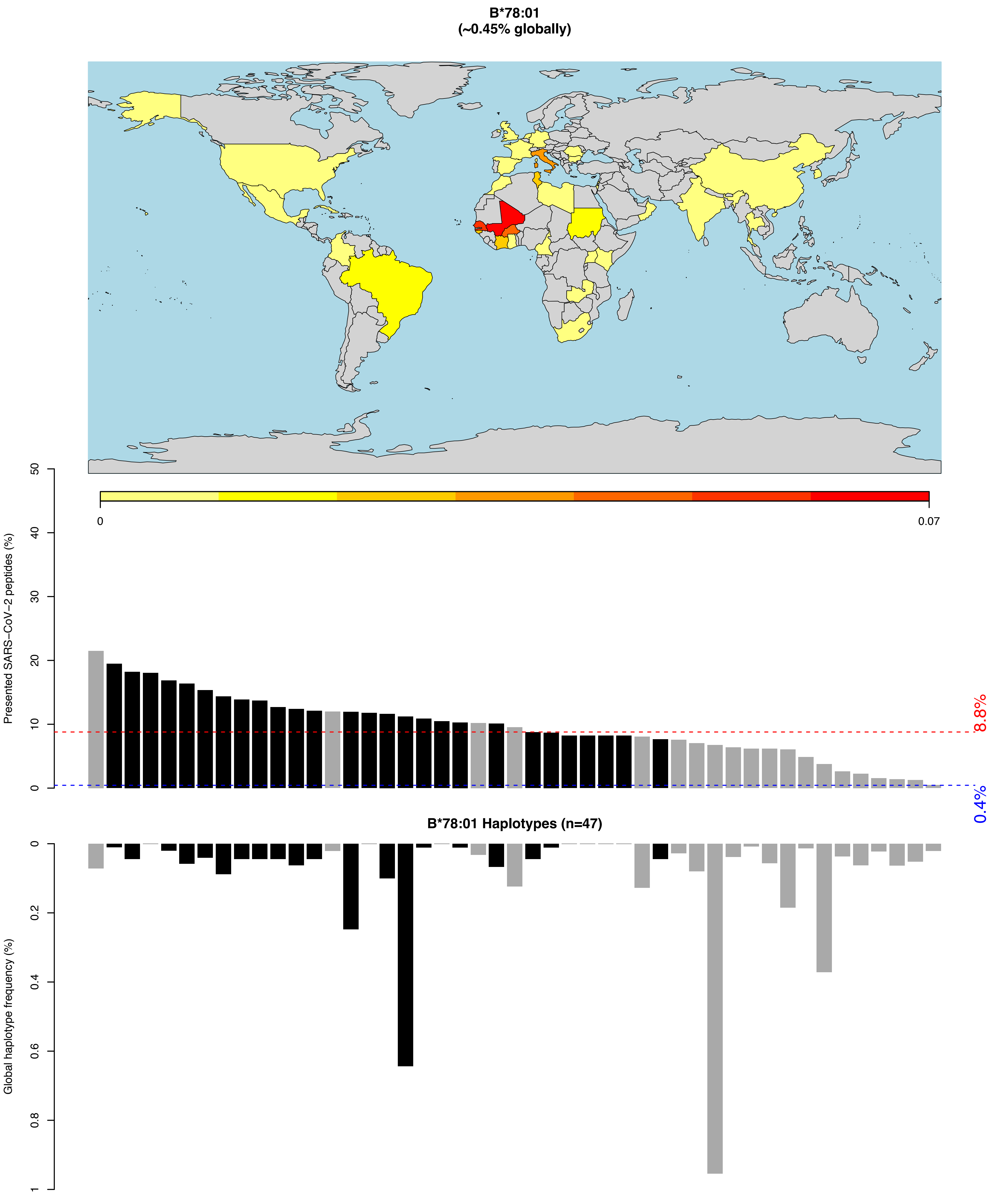

B*78:01
(~0.45% globally)
0
0.07
Presented SARS−CoV−2 peptides (%)
50
40
30
20
10
0
8.8%
0.4%
B*78:01 Haplotypes (n=47)
Global haplotype frequency (%)
0
0.2
0.4
0.6
0.8
1

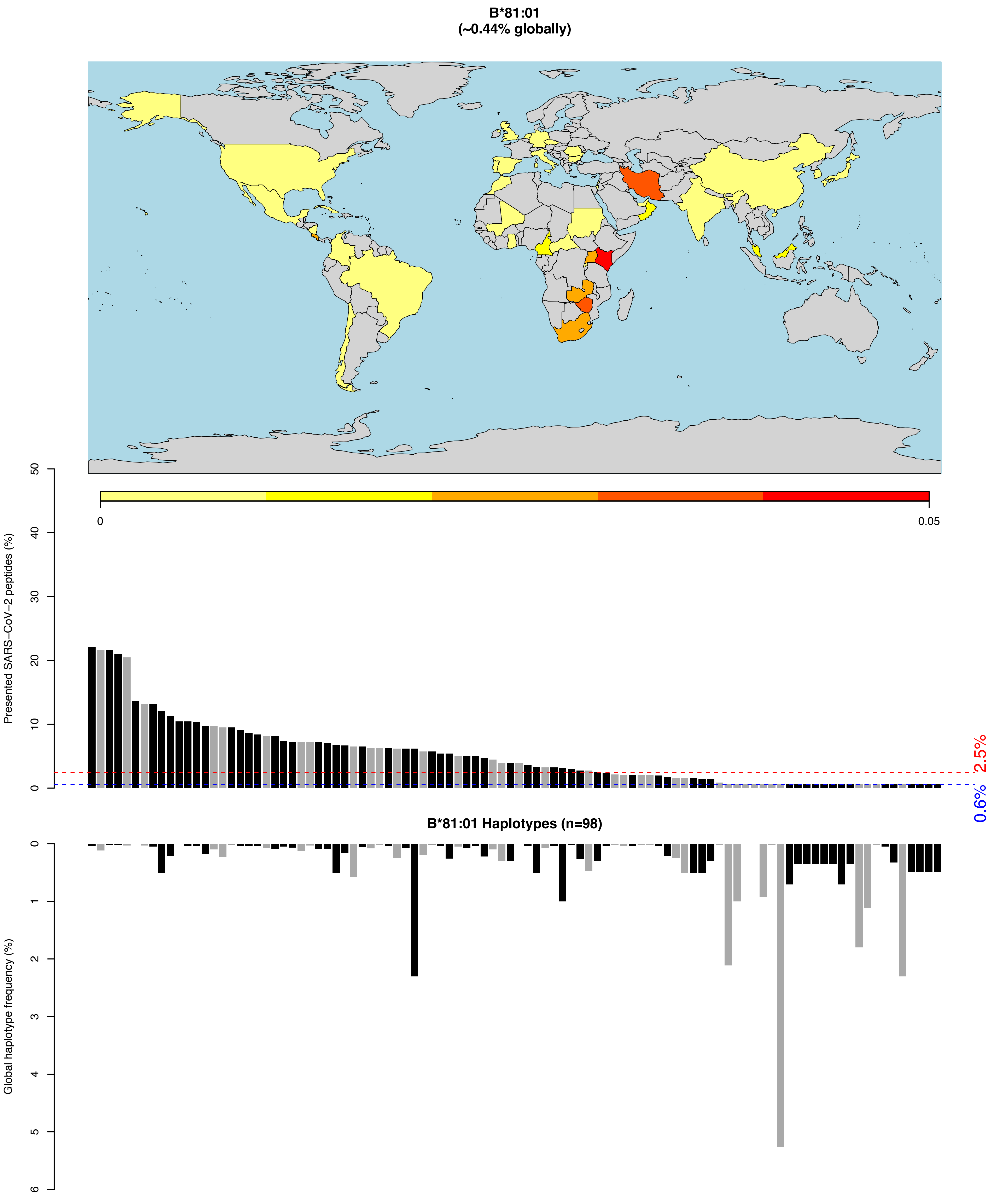
B*81:01
(~0.44% globally)
0
0.05
Presented SARS-CoV-2 peptides (%)
50
40
30
20
10
0
2.5%
0.6%
B*81:01 Haplotypes (n=98)
Global haplotype frequency (%)
0
1
2
3
4
5
6

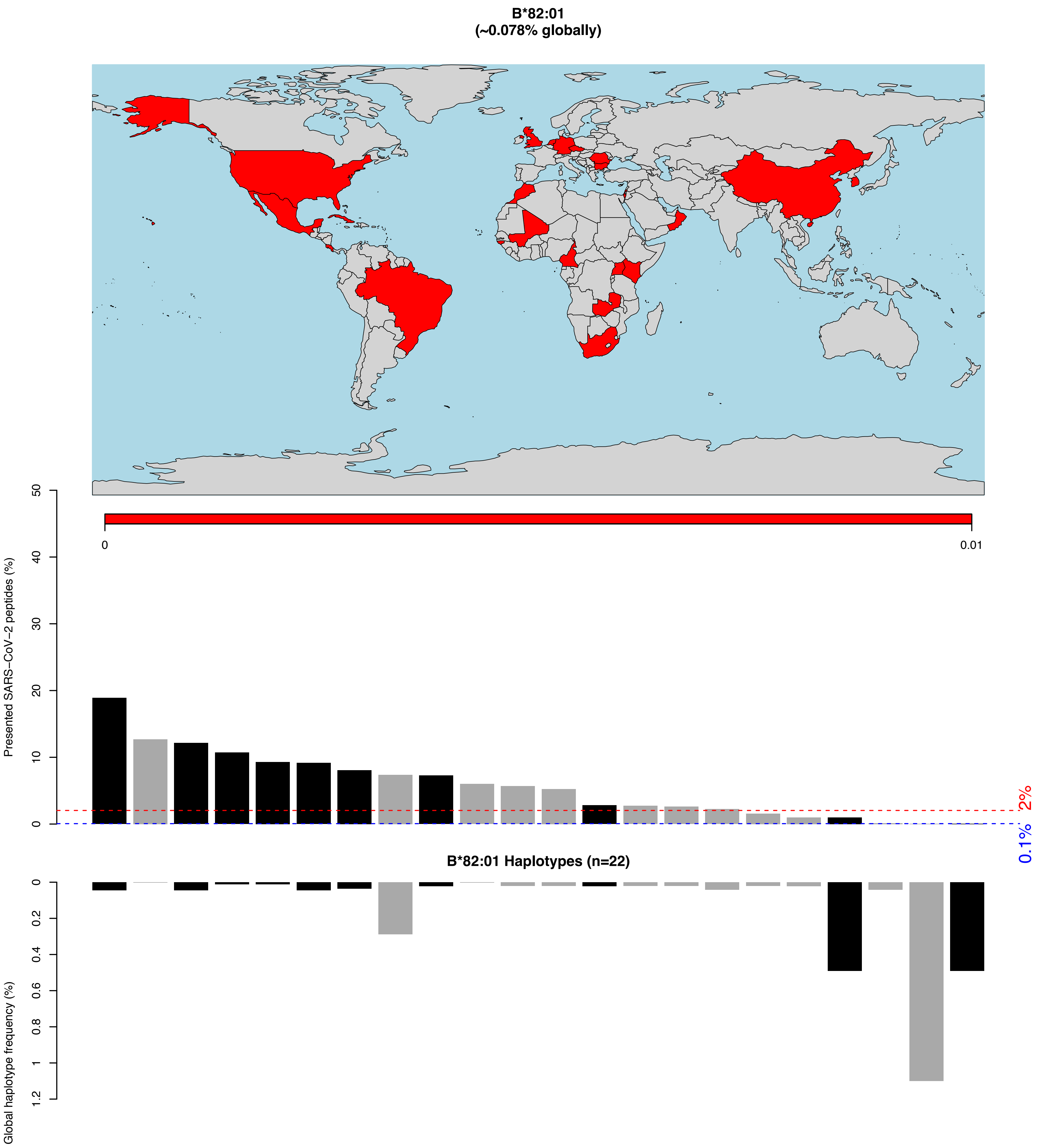
B*82:01
(~0.078% globally)
0
0.01
Presented SARS−CoV−2 peptides (%)
50
40
30
20
10
0
2%
0.1%
B*82:01 Haplotypes (n=22)
Global haplotype frequency (%)
0
0.2
0.4
0.6
0.8
1
1.2

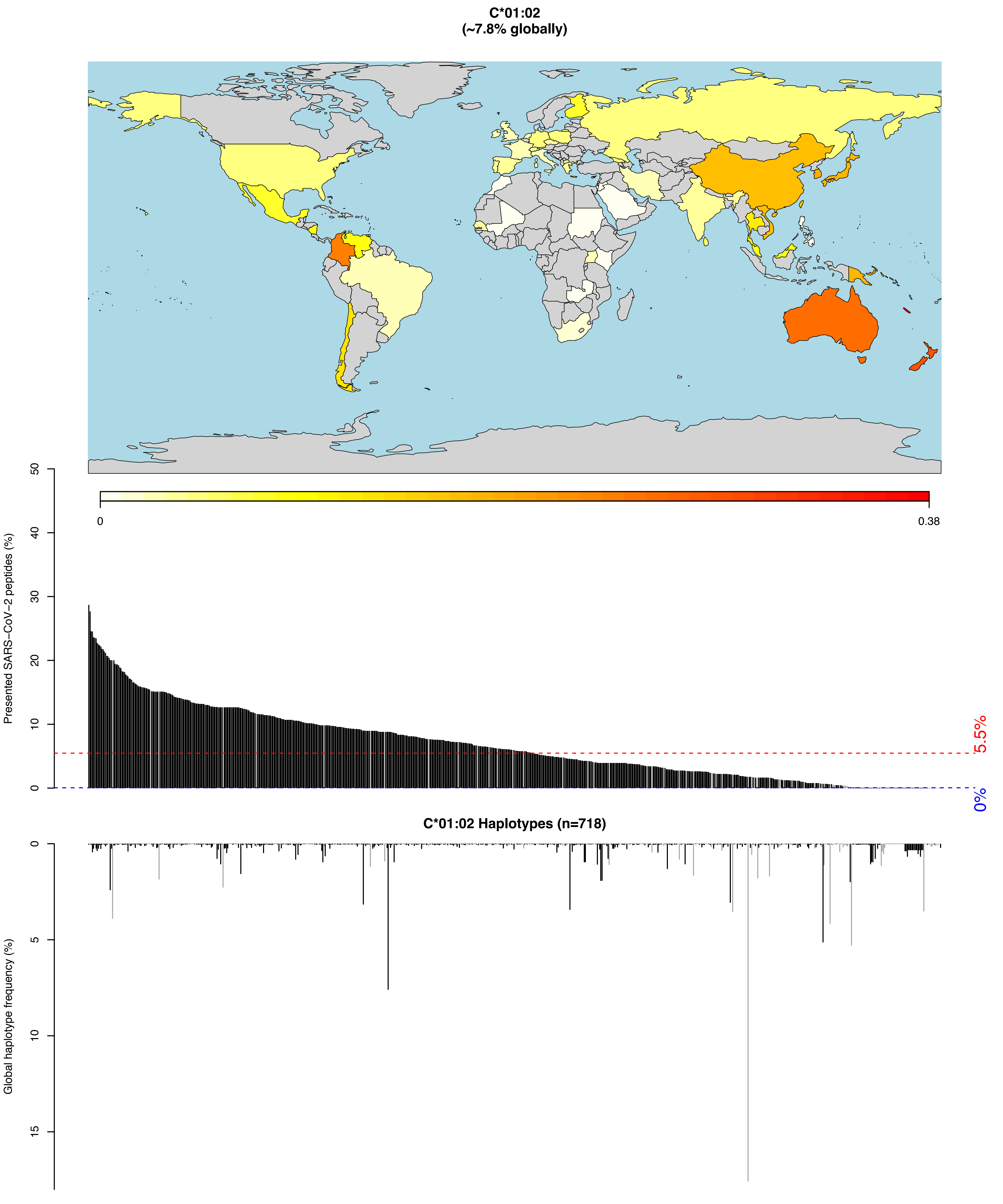

C*01:02
(~7.8% globally)
0
0.38
Presented SARS−CoV−2 peptides (%)
50
40
30
20
10
0
5.5%
0%
C*01:02 Haplotypes (n=718)
Global haplotype frequency (%)
0
5
10
15

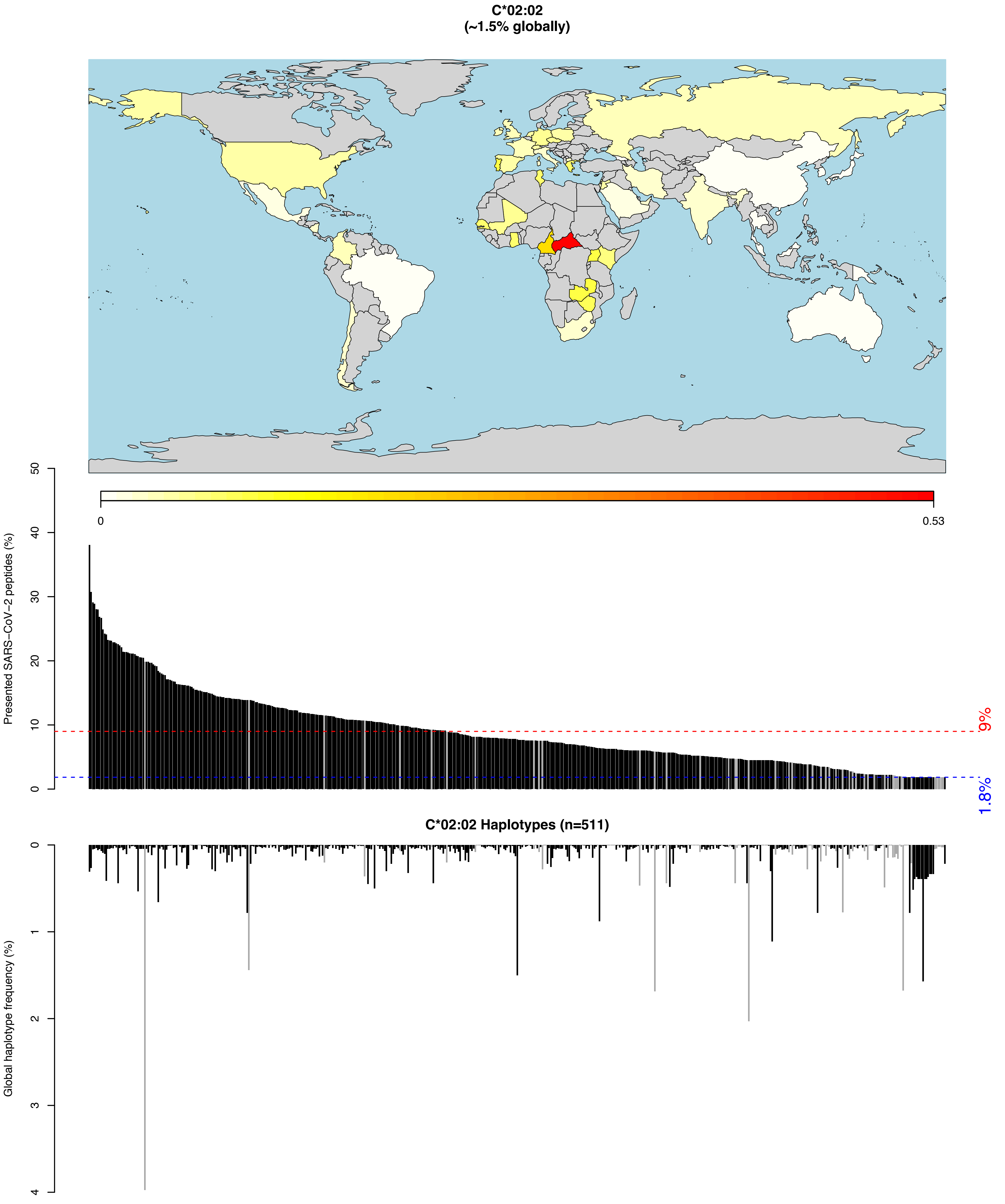
C*02:02
(~1.5% globally)
0
0.53
Presented SARS−CoV−2 peptides (%)
50
40
30
20
10
0
9%
1.8%
C*02:02 Haplotypes (n=511)
Global haplotype frequency (%)
0
1
2
3
4

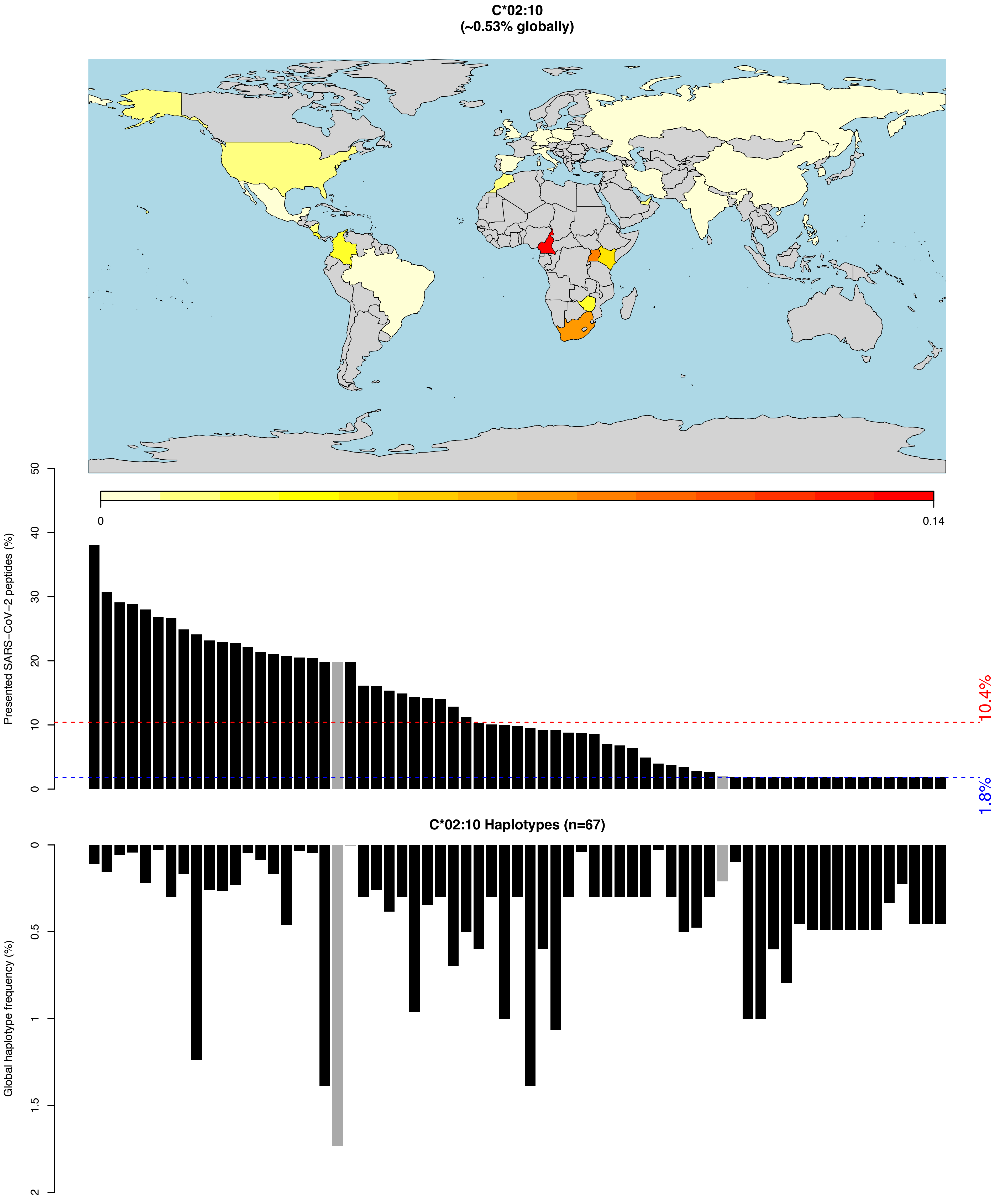

C*02:10
(~0.53% globally)
0
0.14
Presented SARS−CoV−2 peptides (%)
10.4%
1.8%
C*02:10 Haplotypes (n=67)
Global haplotype frequency (%)

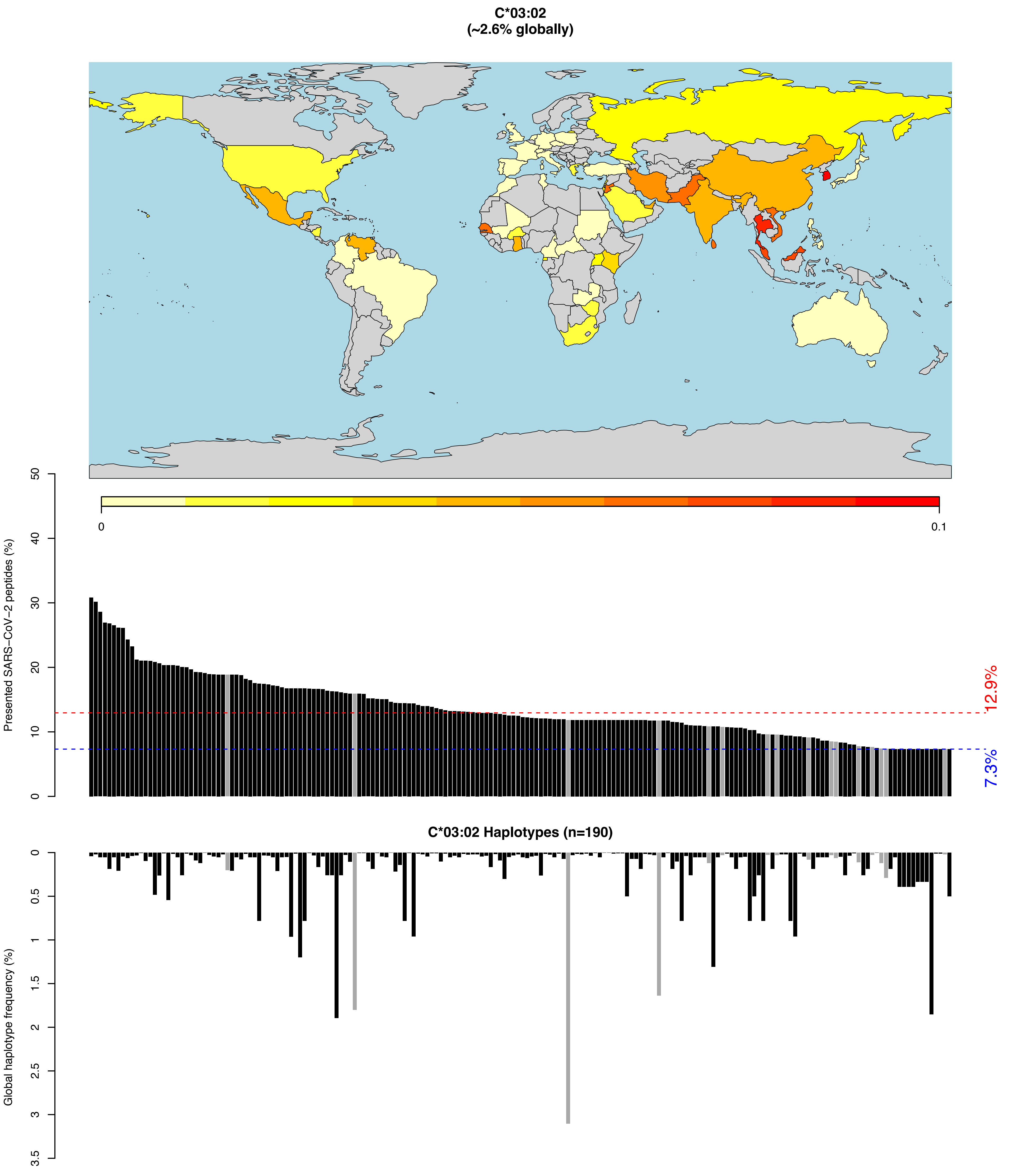
C*03:02
(~2.6% globally)
0
0.1
Presented SARS−CoV−2 peptides (%)
50
40
30
20
10
0
12.9%
7.3%
C*03:02 Haplotypes (n=190)
Global haplotype frequency (%)
0
0.5
1
1.5
2
2.5
3
3.5

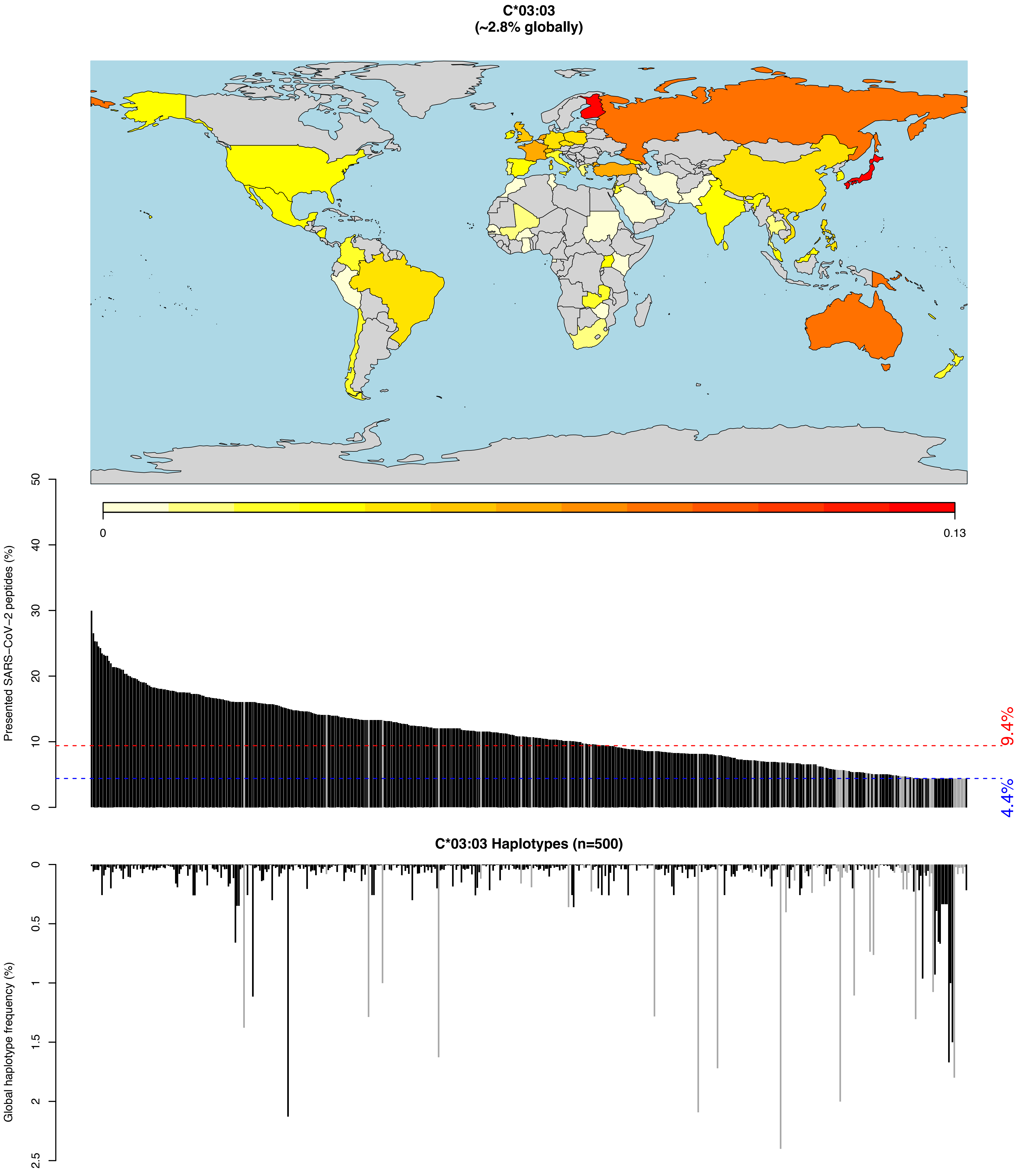
C*03:03
(~2.8% globally)
0
0.13
Presented SARS−CoV−2 peptides (%)
50
40
30
20
10
0
9.4%
4.4%
C*03:03 Haplotypes (n=500)
Global haplotype frequency (%)
0
0.5
1
1.5
2
2.5

C*03:04
(~3.9% globally)

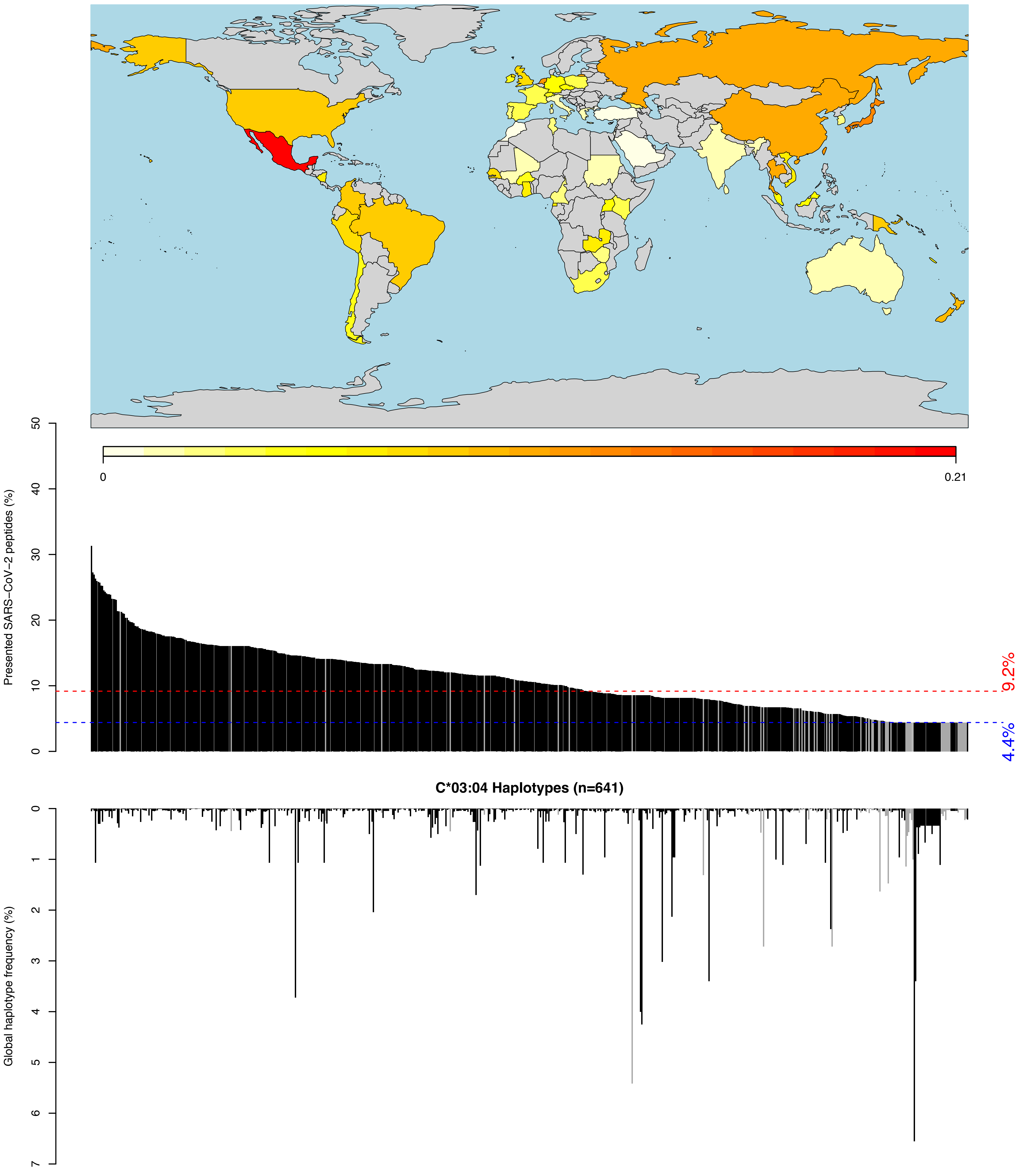

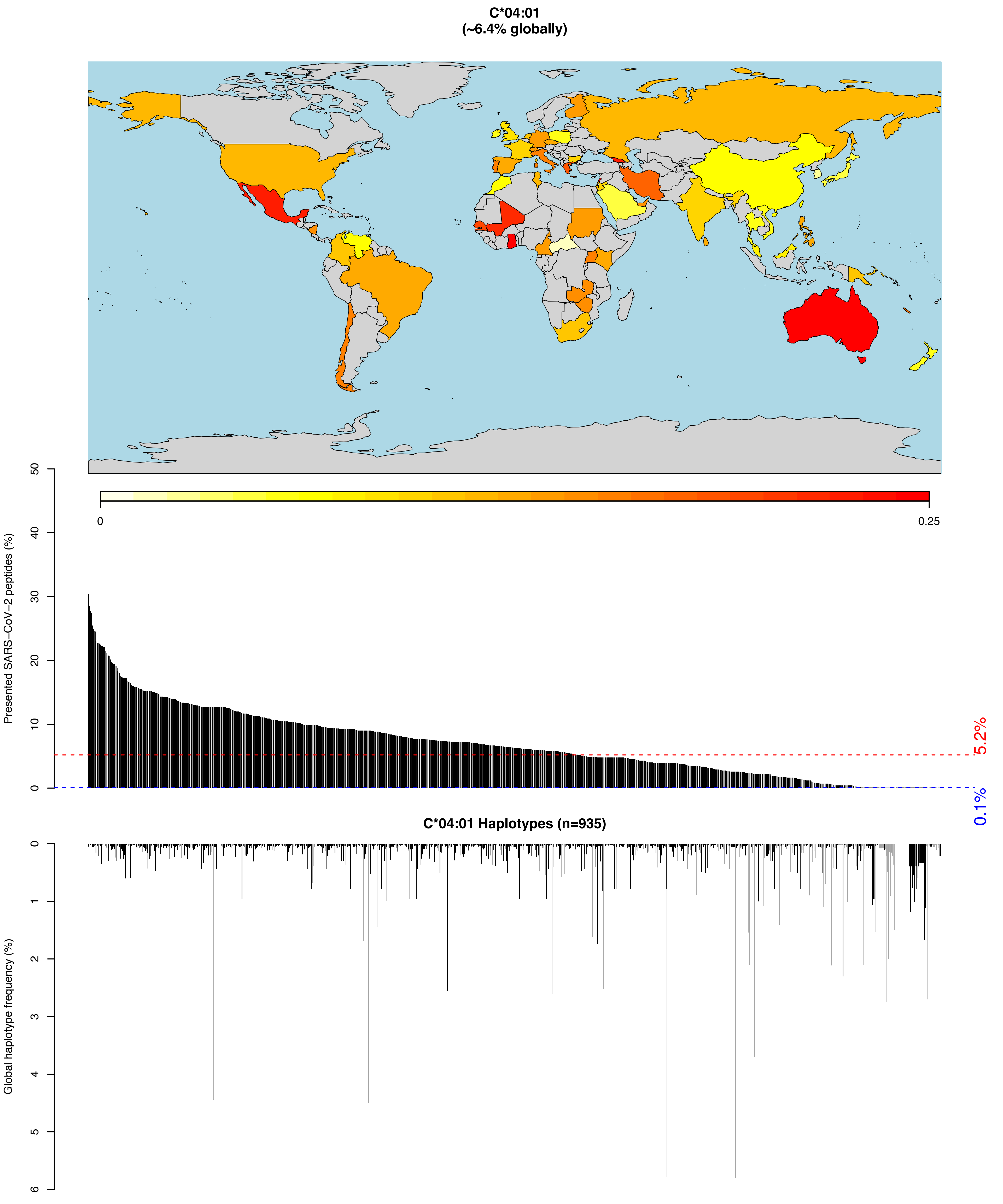

C*04:01
(~6.4% globally)
0
0.25
Presented SARS−CoV−2 peptides (%)
0
10
20
30
40
50
5.2%
0.1%
C*04:01 Haplotypes (n=935)
Global haplotype frequency (%)
0
1
2
3
4
5
6

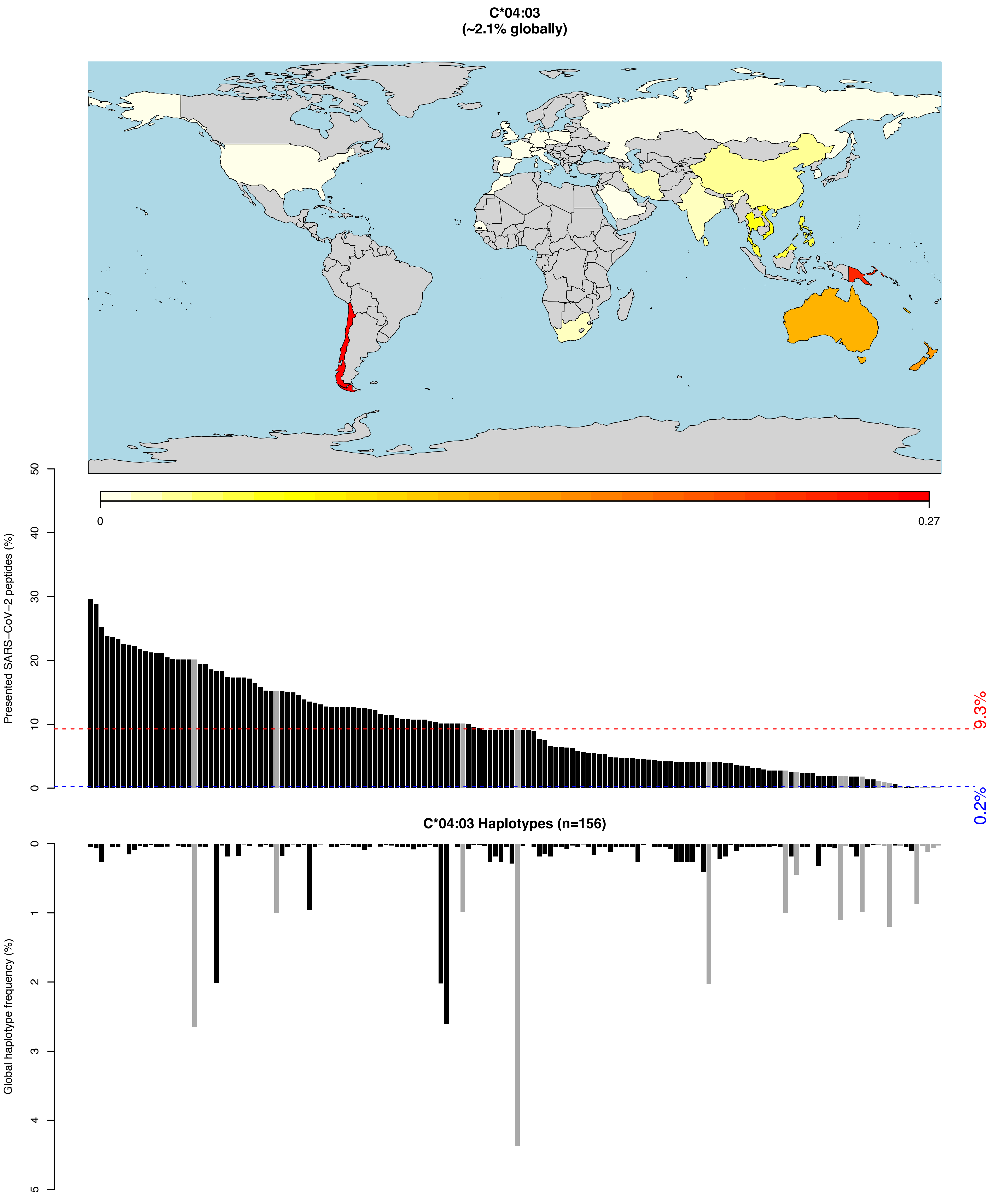

C*04:03
(~2.1% globally)
0
0.27
Presented SARS−CoV−2 peptides (%)
50
40
30
20
10
0
9.3%
0.2%
C*04:03 Haplotypes (n=156)
Global haplotype frequency (%)
0
1
2
3
4
5

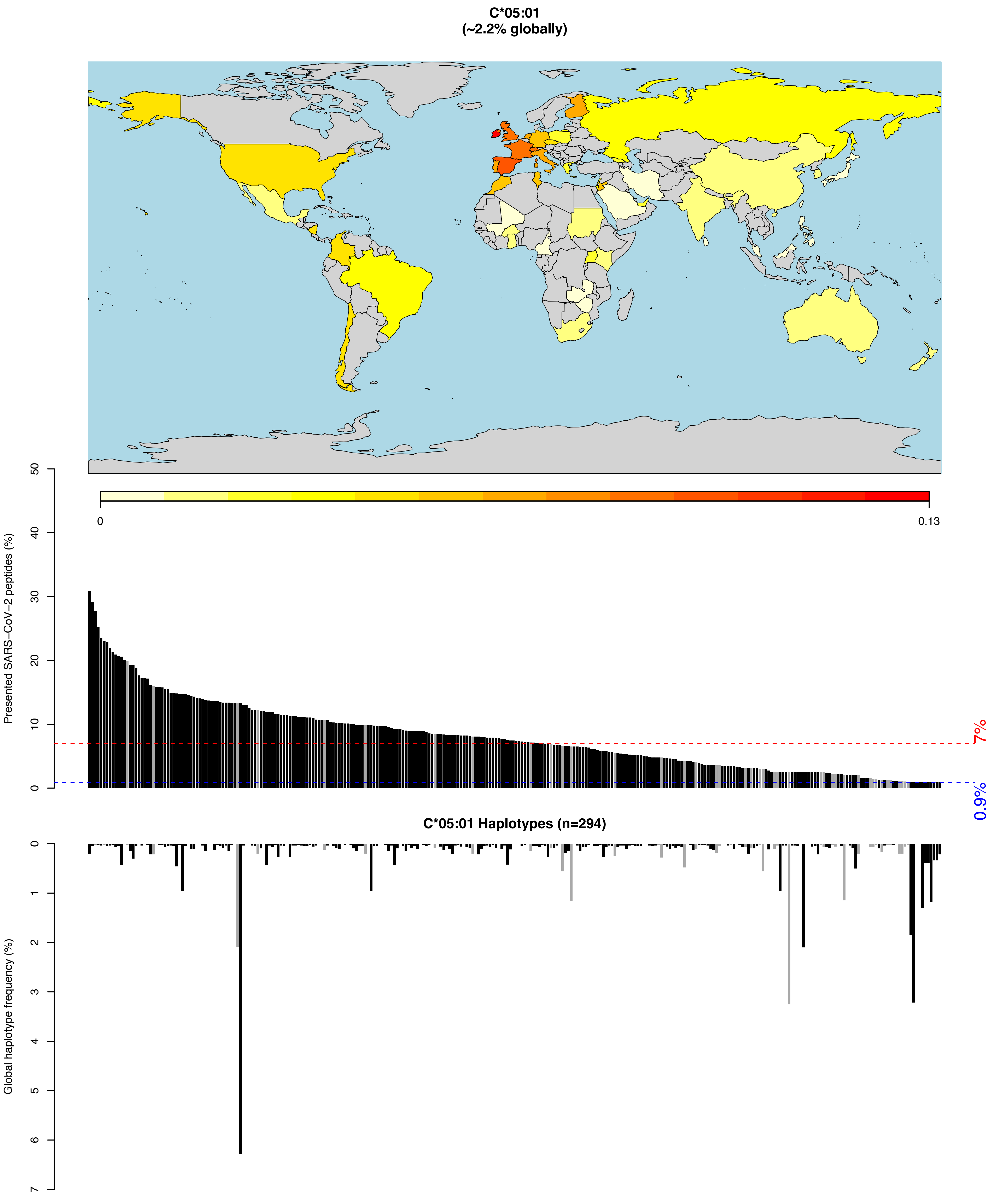
C*05:01
(~2.2% globally)
0
0.13
Presented SARS−CoV−2 peptides (%)
0
10
20
30
40
50
7%
0.9%
C*05:01 Haplotypes (n=294)
Global haplotype frequency (%)
0
1
2
3
4
5
6
7

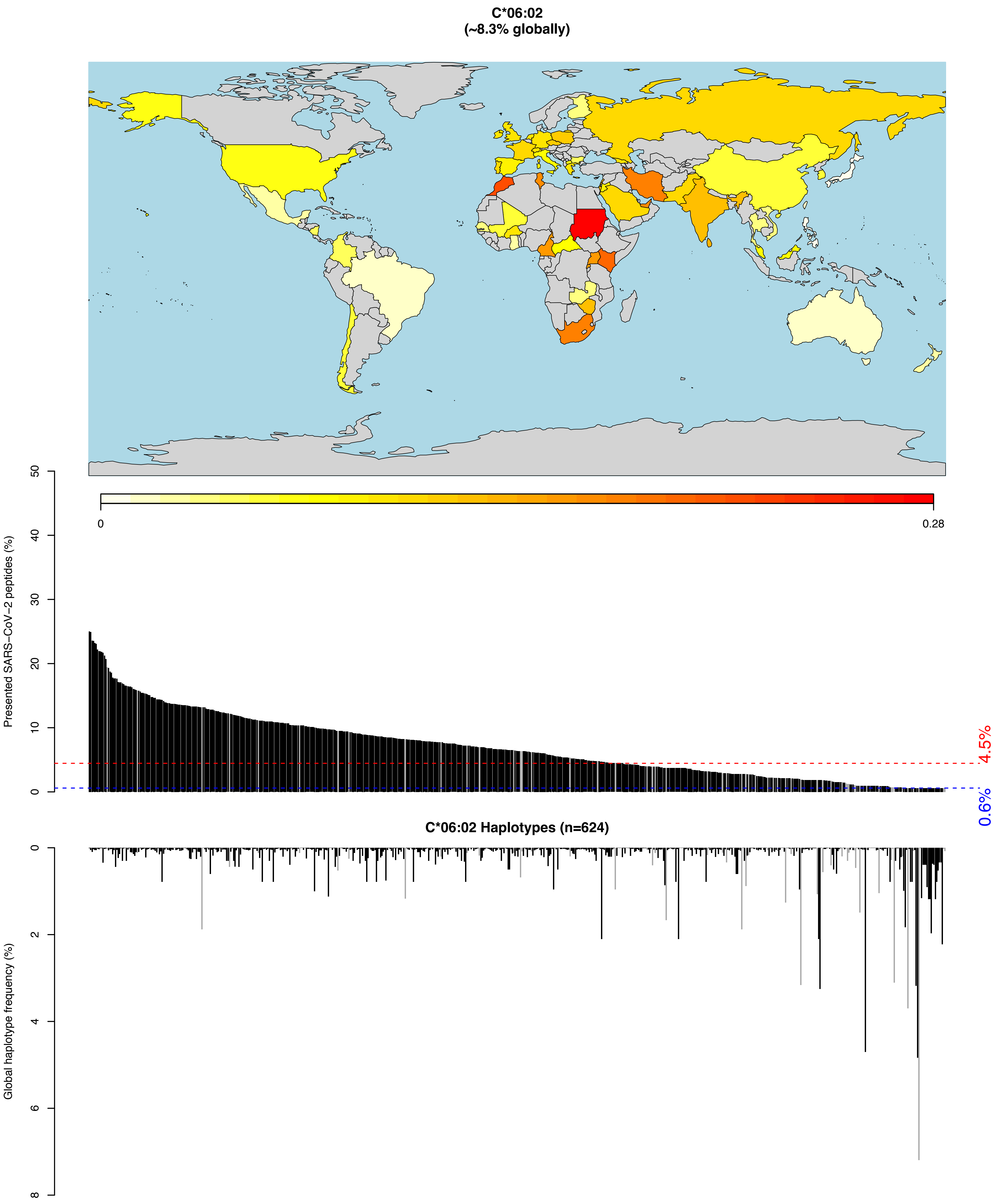
C*06:02
(~8.3% globally)
0
0.28
Presented SARS−CoV−2 peptides (%)
50
40
30
20
10
0
4.5%
0.6%
C*06:02 Haplotypes (n=624)
Global haplotype frequency (%)
0
2
4
6
8

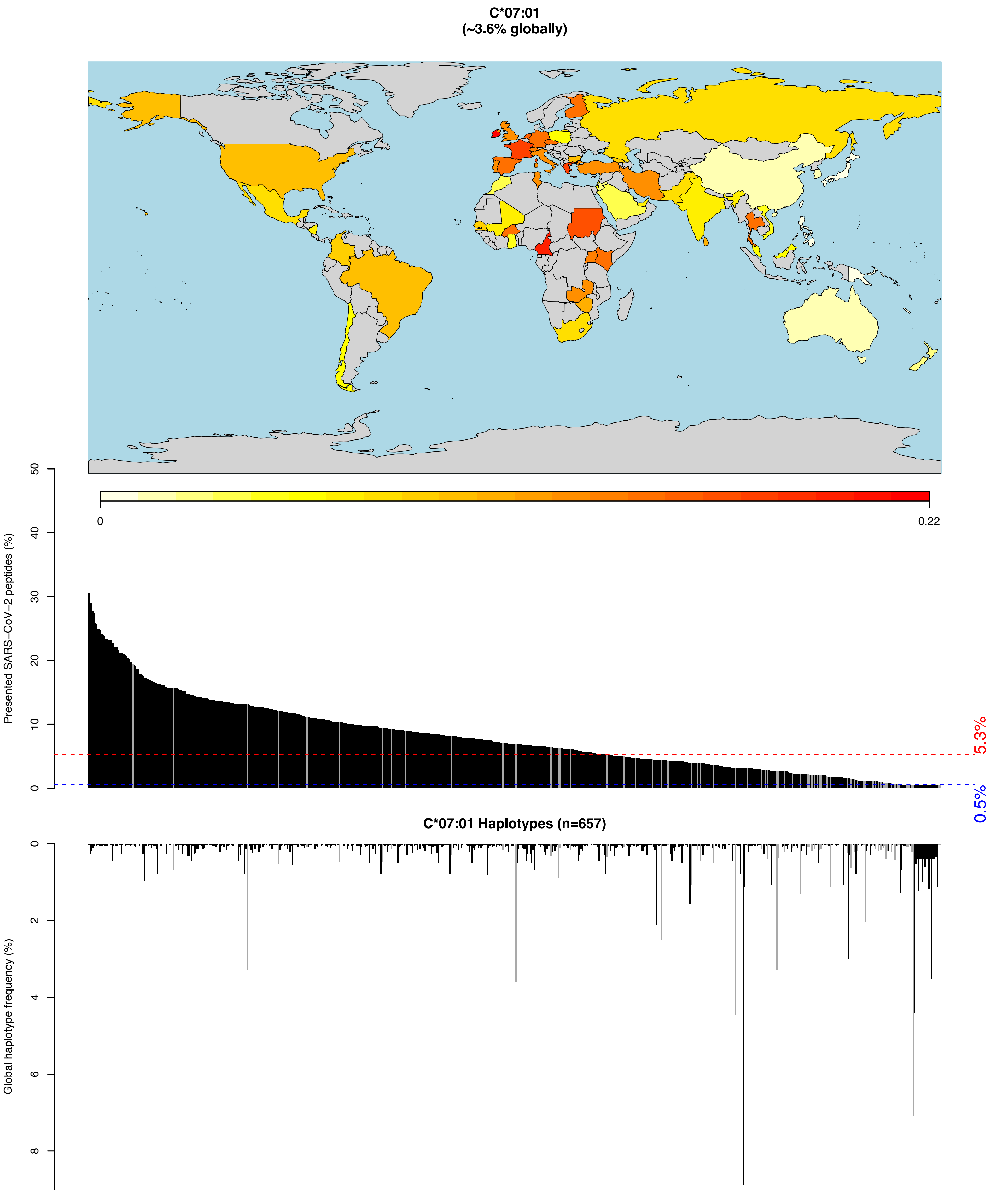

C*07:01
(~3.6% globally)
0
0.22
Presented SARS−CoV−2 peptides (%)
0
10
20
30
40
50
5.3%
0.5%
C*07:01 Haplotypes (n=657)
Global haplotype frequency (%)
0
2
4
6
8

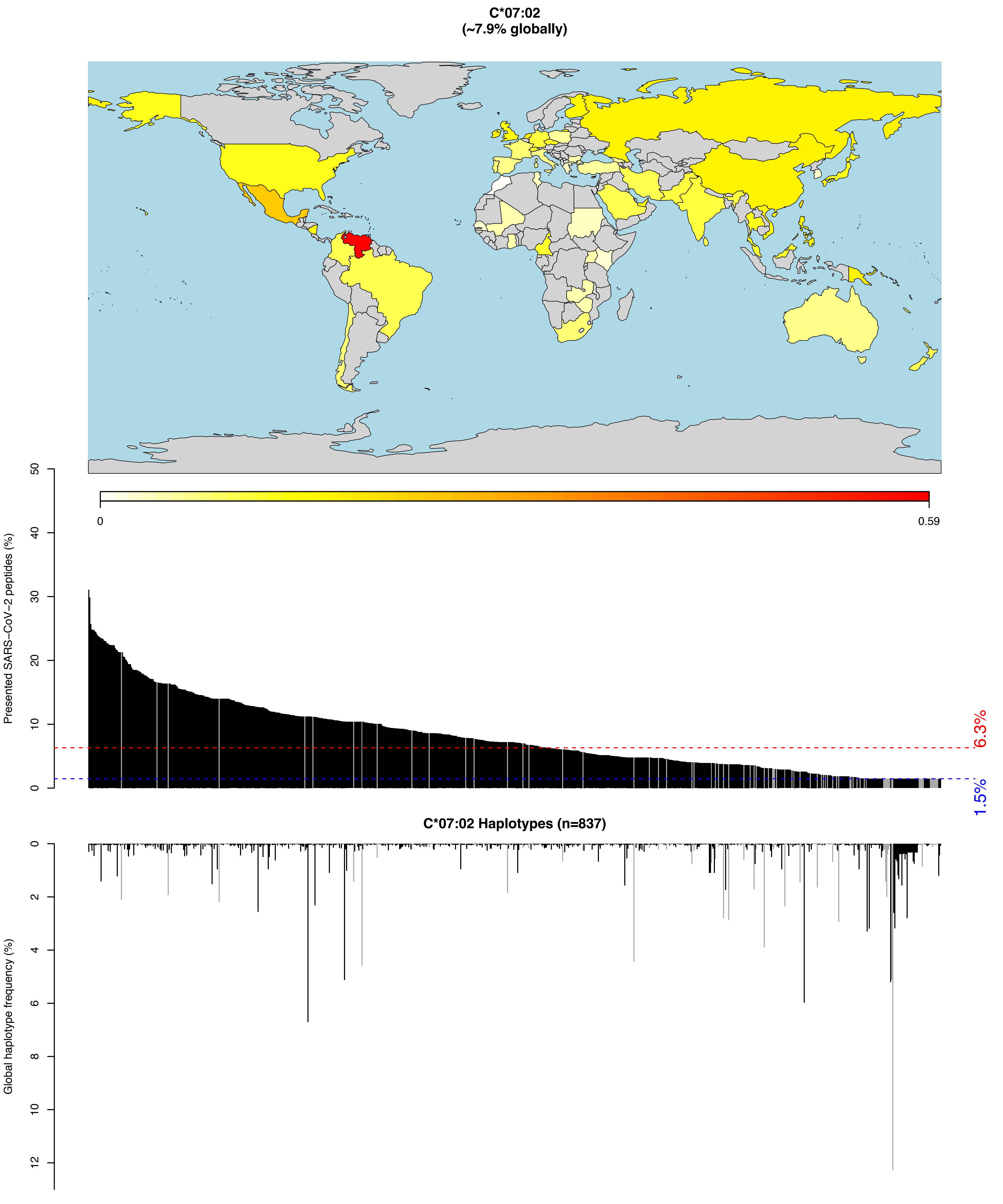

C*07:02
(~7.9% globally)
0
0.59
Presented SARS−CoV−2 peptides (%)
0
10
20
30
40
50
6.3%
1.5%
C*07:02 Haplotypes (n=837)
Global haplotype frequency (%)
0
2
4
6
8
10
12

C*07:04
(~1.8% globally)

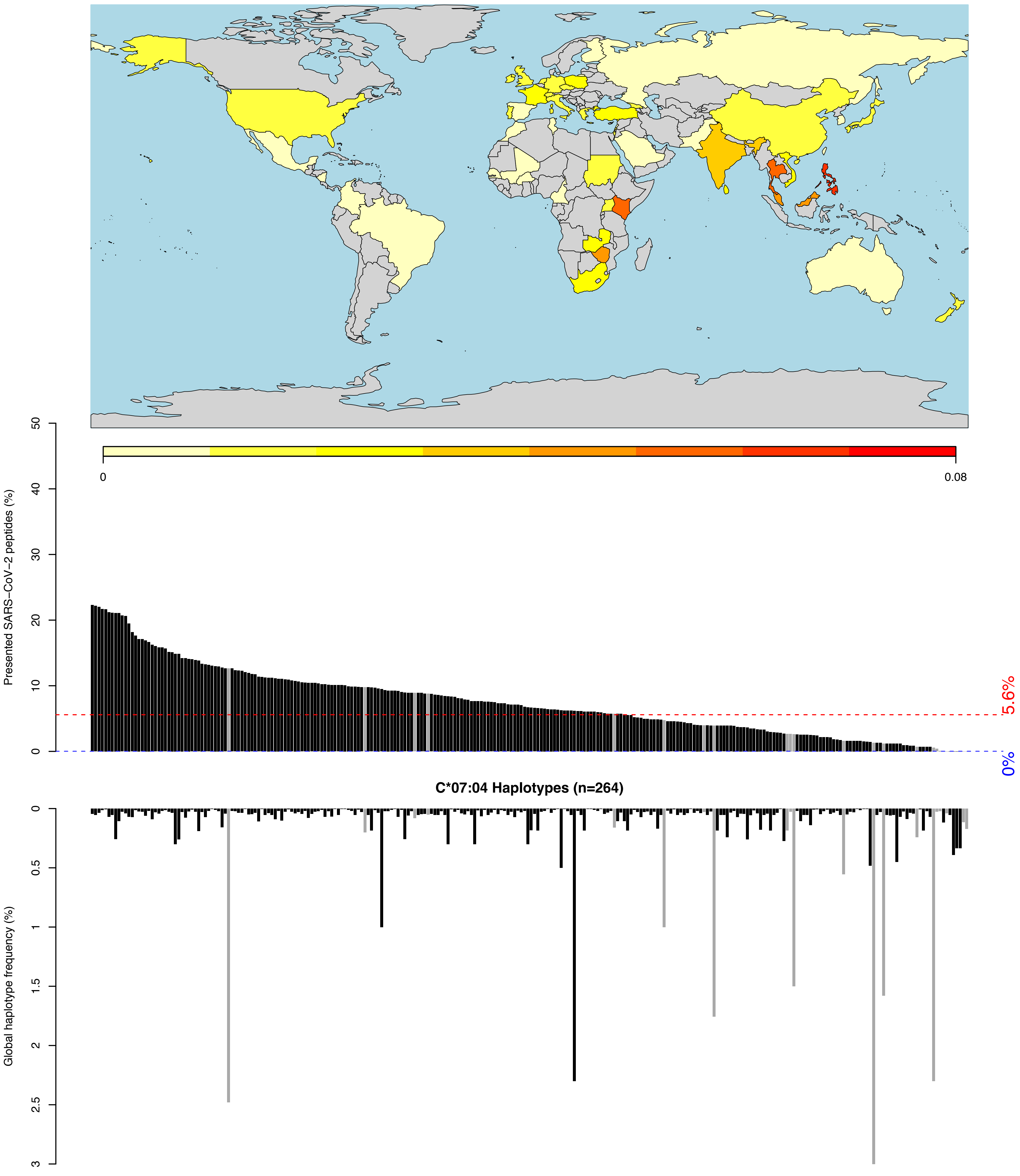

C*08:01
(~5.3% globally)

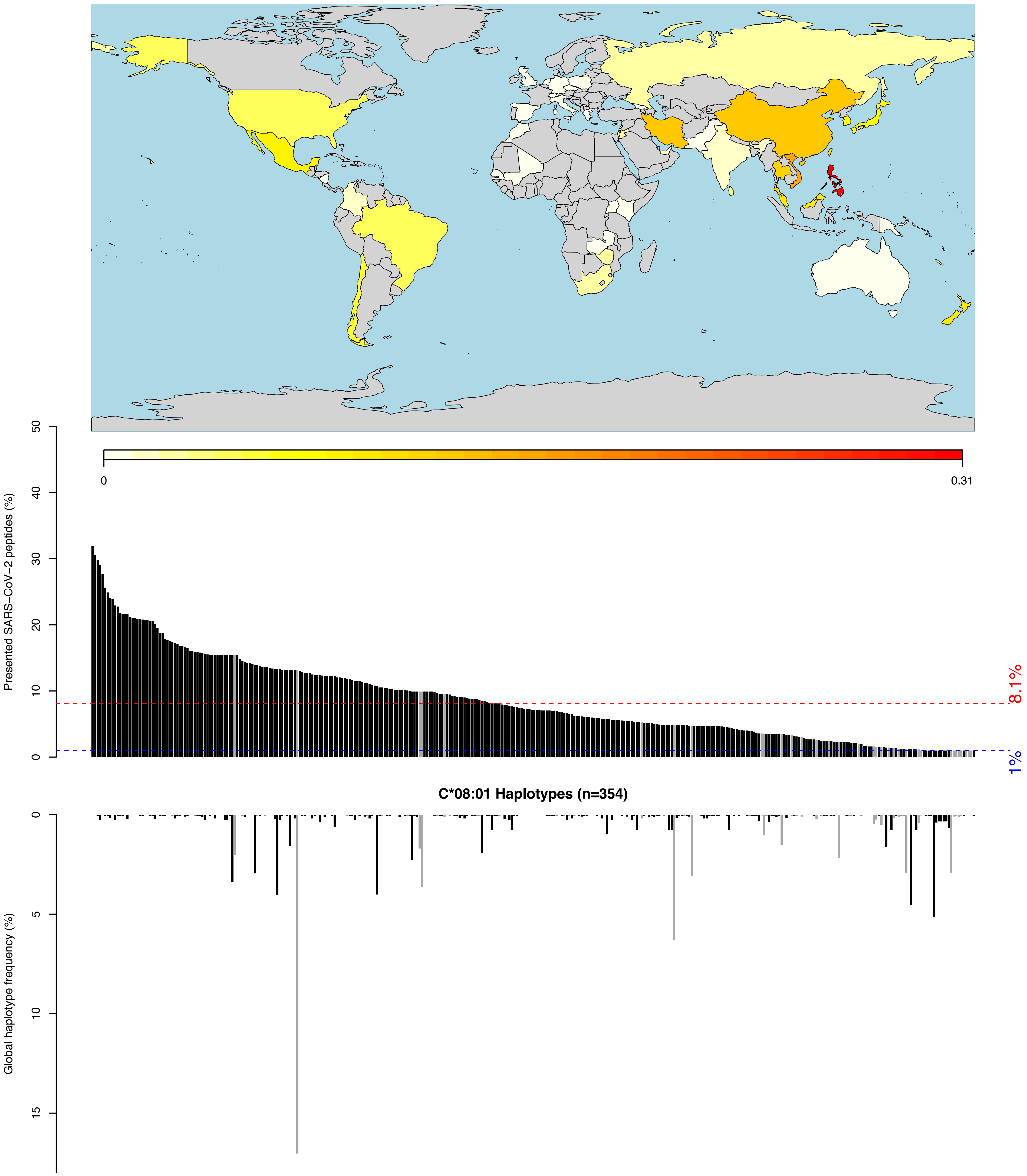

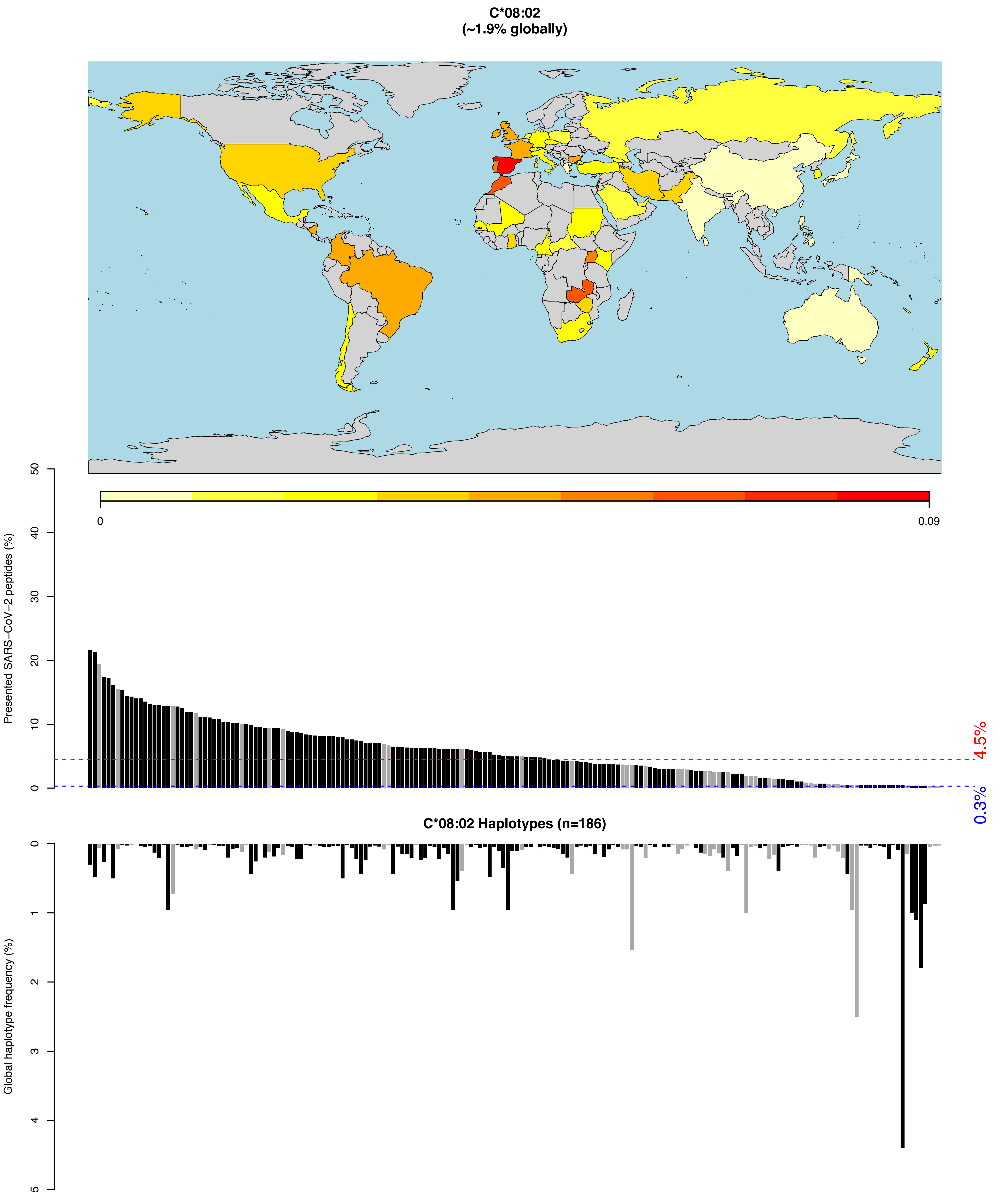
C*08:02
(~1.9% globally)
0
0.09
Presented SARS−CoV−2 peptides (%)
50
40
30
20
10
0
4.5%
0.3%
C*08:02 Haplotypes (n=186)
Global haplotype frequency (%)
0
1
2
3
4
5

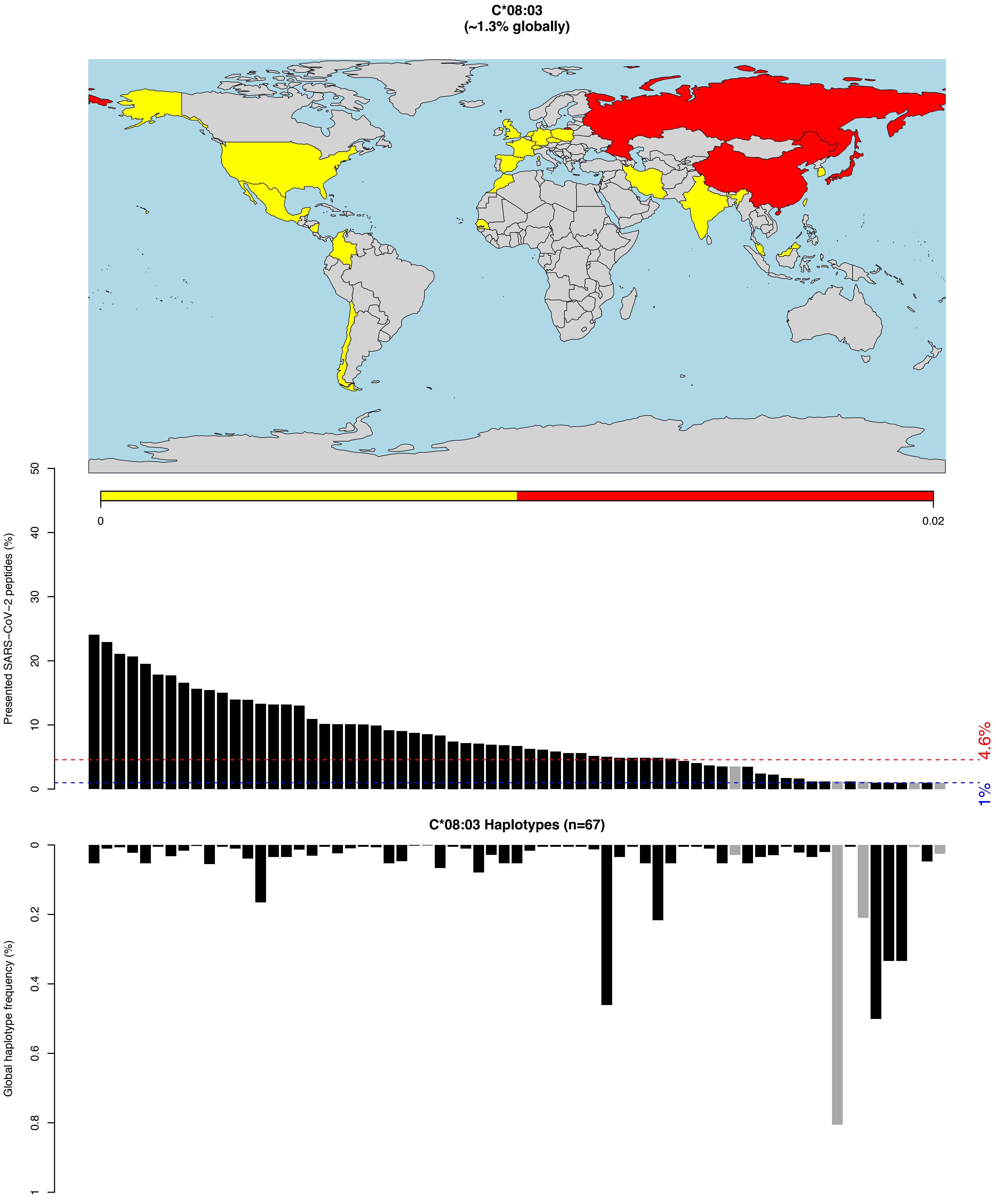

C*08:03
(~1.3% globally)
0
0.02
Presented SARS−CoV−2 peptides (%)
50
40
30
20
10
0
4.6%
1%
C*08:03 Haplotypes (n=67)
Global haplotype frequency (%)
0
0.2
0.4
0.6
0.8
1

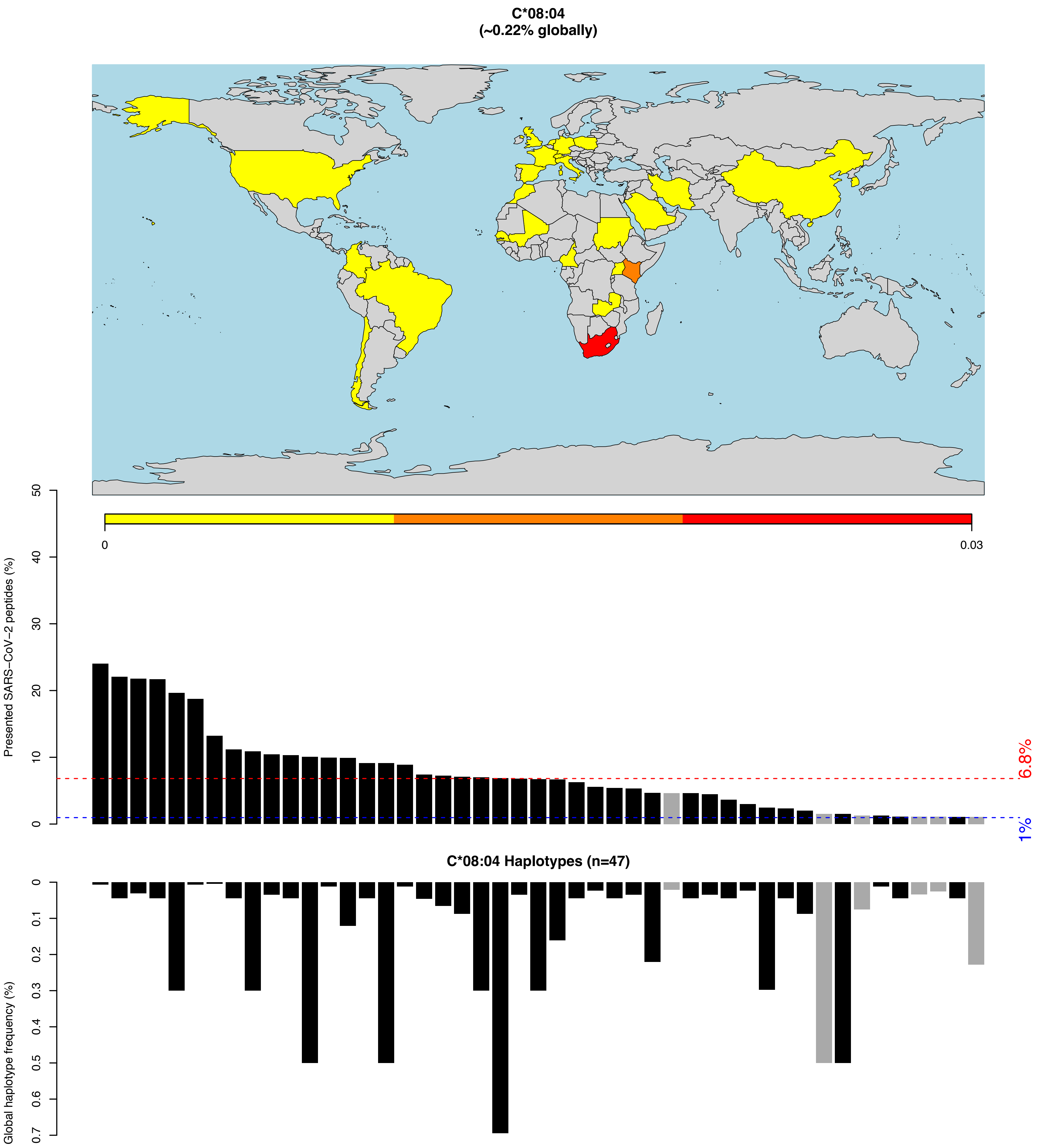
C*08:04
(~0.22% globally)
0
0.03
Presented SARS−CoV−2 peptides (%)
6.8%
1%
C*08:04 Haplotypes (n=47)
Global haplotype frequency (%)

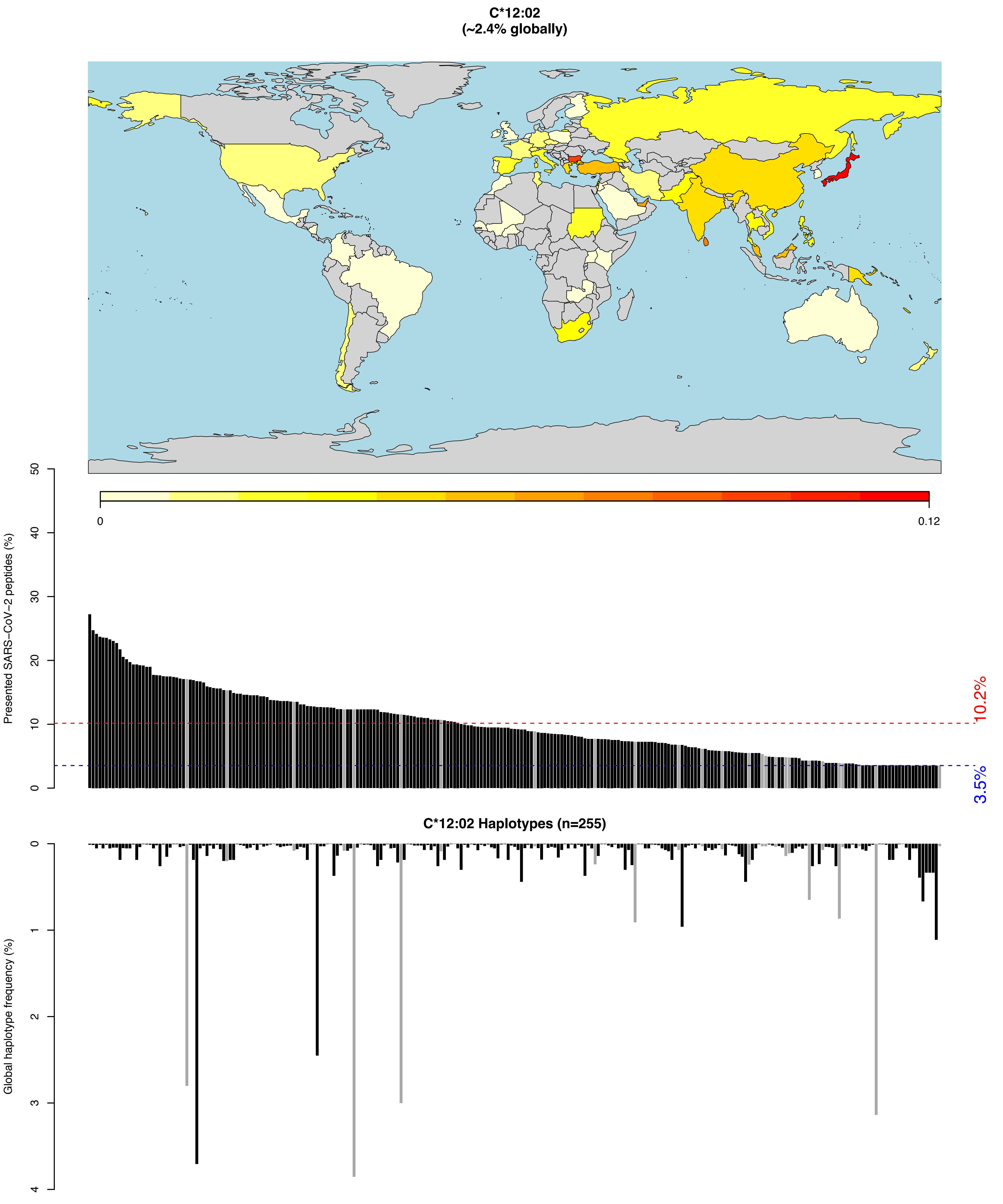

C*12:02
(~2.4% globally)
0
0.12
Presented SARS−CoV−2 peptides (%)
0
10
20
30
40
50
10.2%
3.5%
C*12:02 Haplotypes (n=255)
Global haplotype frequency (%)
0
1
2
3
4

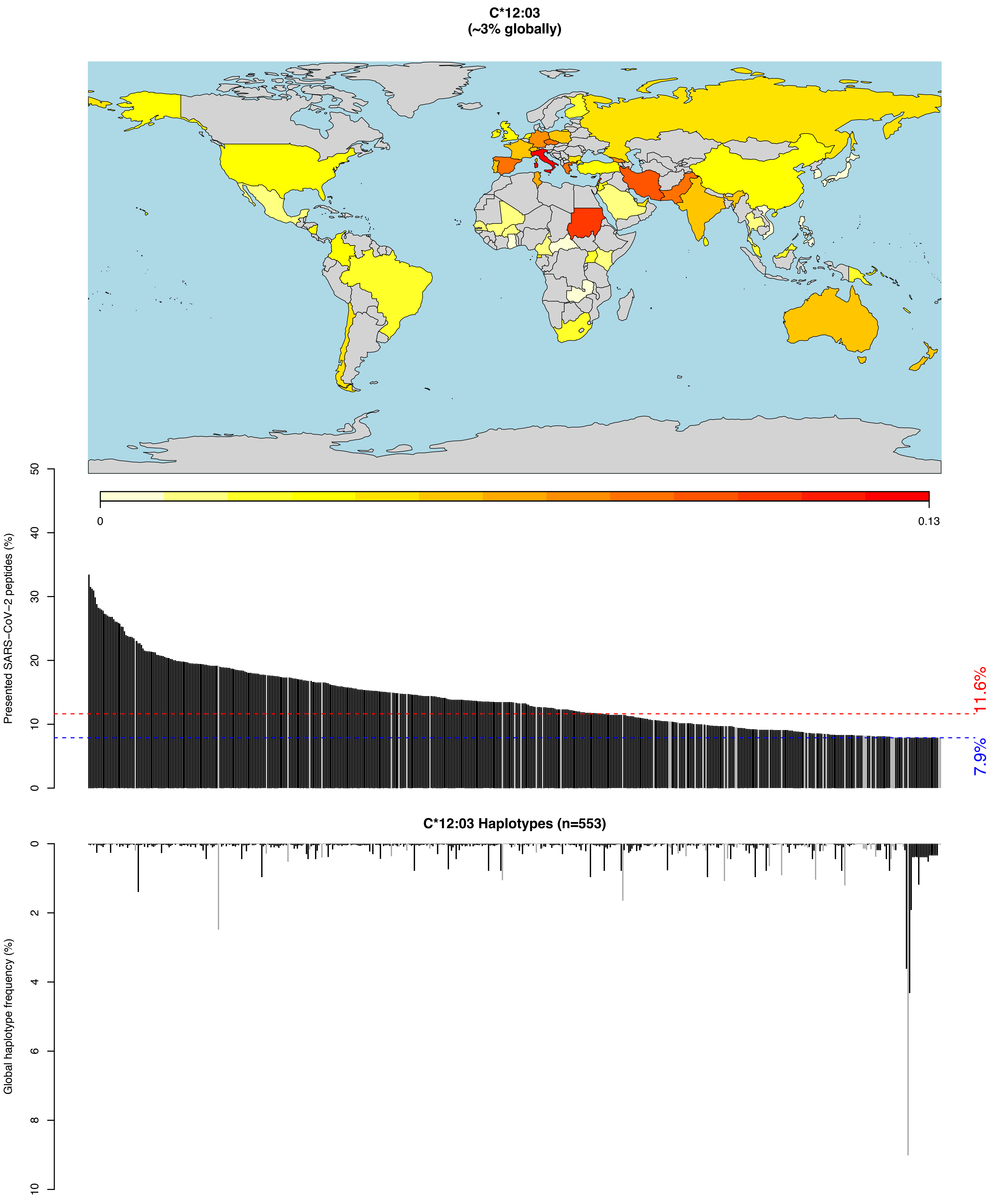
C*12:03
(~3% globally)
0
0.13
Presented SARS−CoV−2 peptides (%)
50
40
30
20
10
0
11.6%
7.9%
C*12:03 Haplotypes (n=553)
Global haplotype frequency (%)
0
2
4
6
8
10

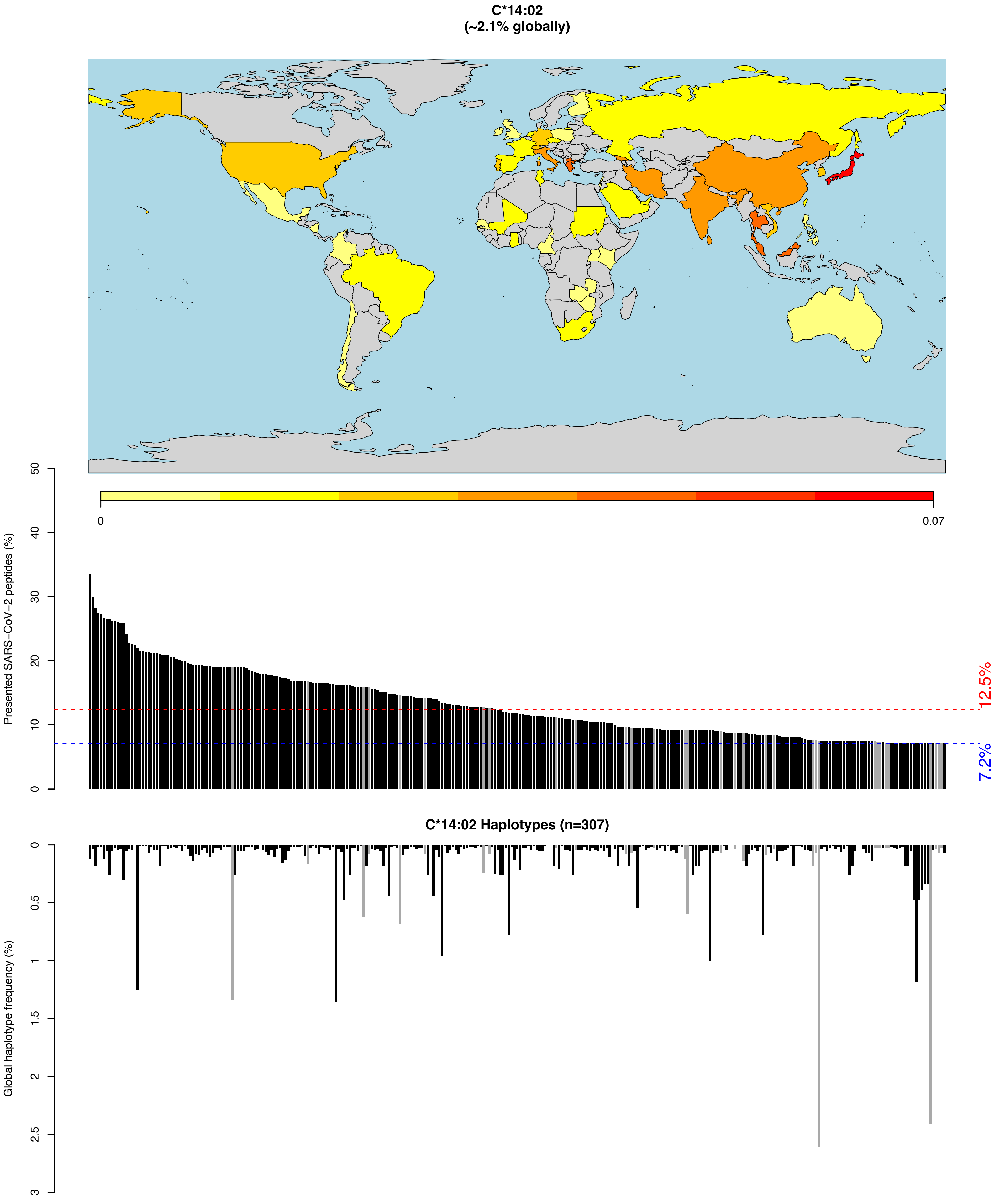
C*14:02
(~2.1% globally)
0
0.07
Presented SARS−CoV−2 peptides (%)
0
10
20
30
40
50
12.5%
7.2%
C*14:02 Haplotypes (n=307)
Global haplotype frequency (%)
0
0.5
1
1.5
2
2.5
3

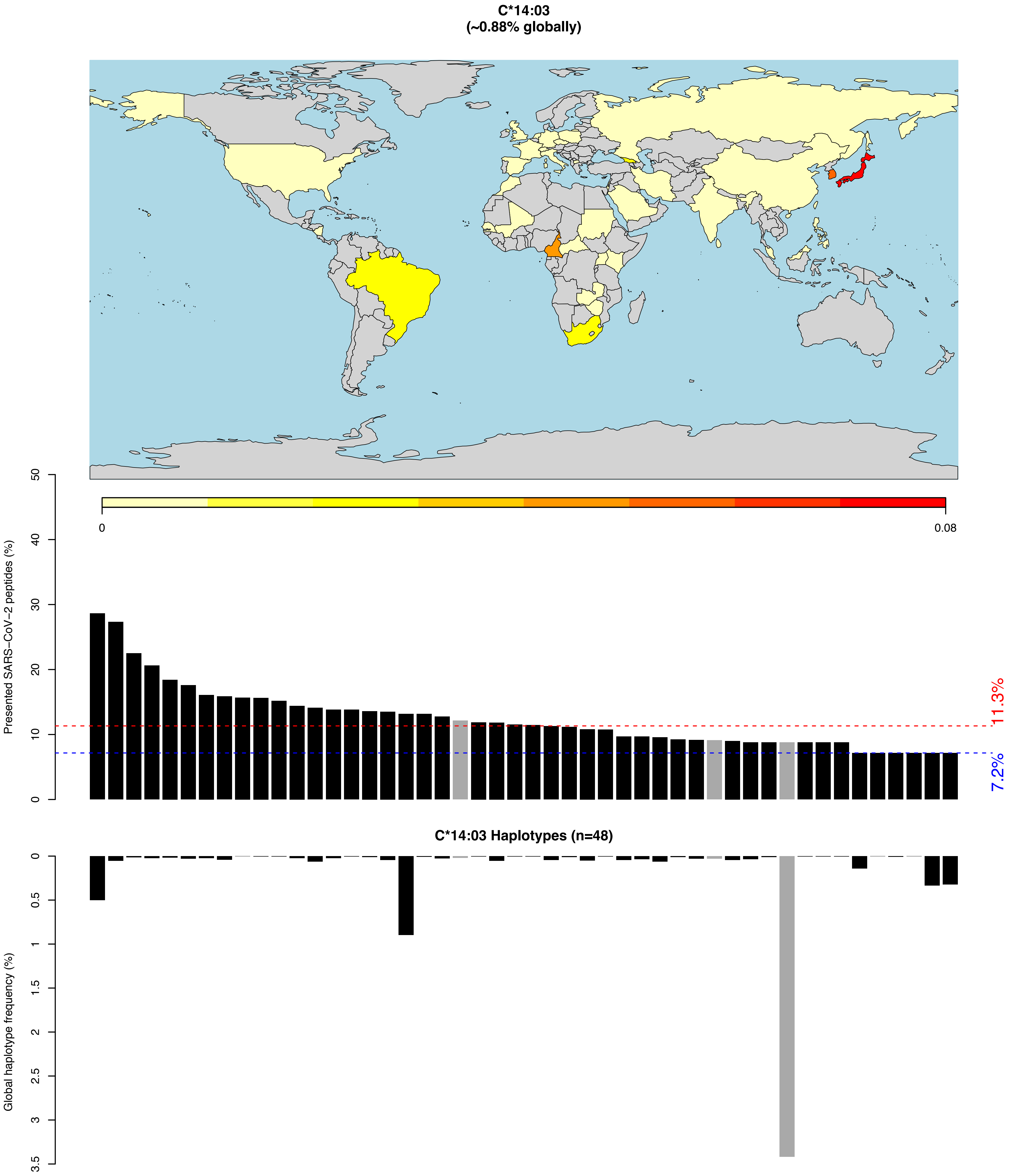

C*14:03
(~0.88% globally)
0
0.08
Presented SARS−CoV−2 peptides (%)
0
10
20
30
40
50
11.3%
7.2%
C*14:03 Haplotypes (n=48)
Global haplotype frequency (%)
0
0.5
1
1.5
2
2.5
3
3.5

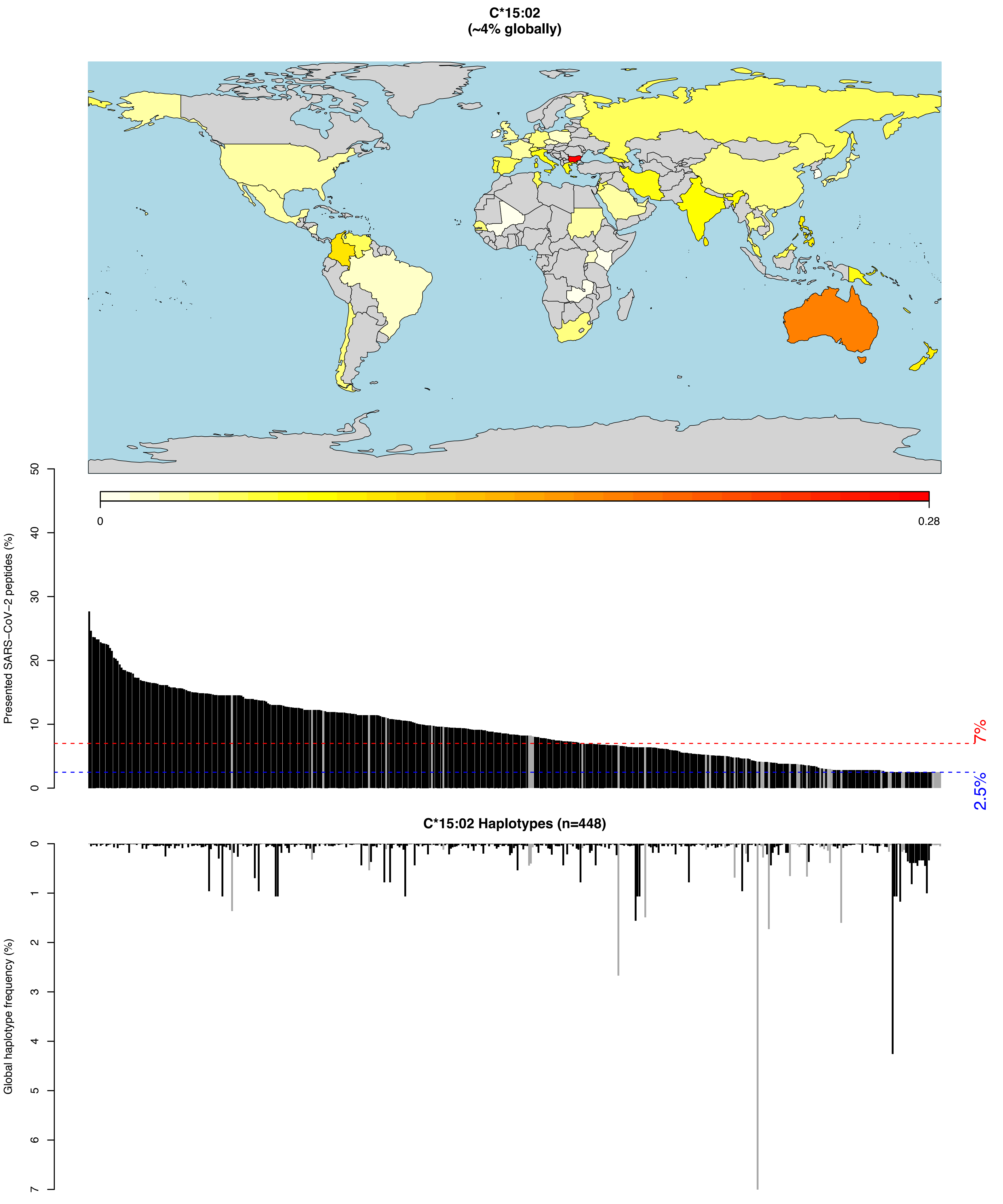
C*15:02
(~4% globally)
0
0.28
Presented SARS−CoV−2 peptides (%)
7%
2.5%
C*15:02 Haplotypes (n=448)
Global haplotype frequency (%)

C*15:05
(~0.56% globally)

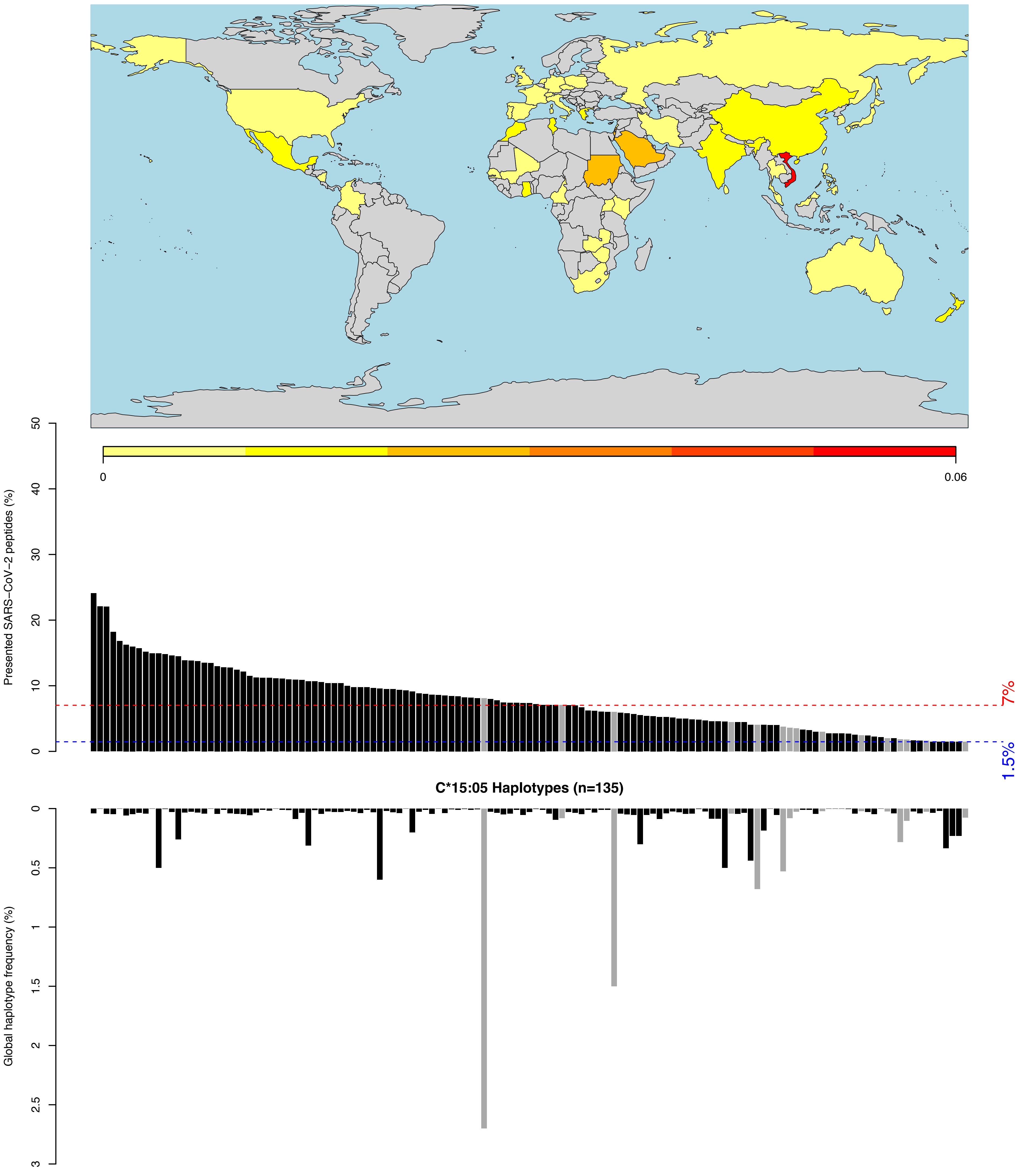

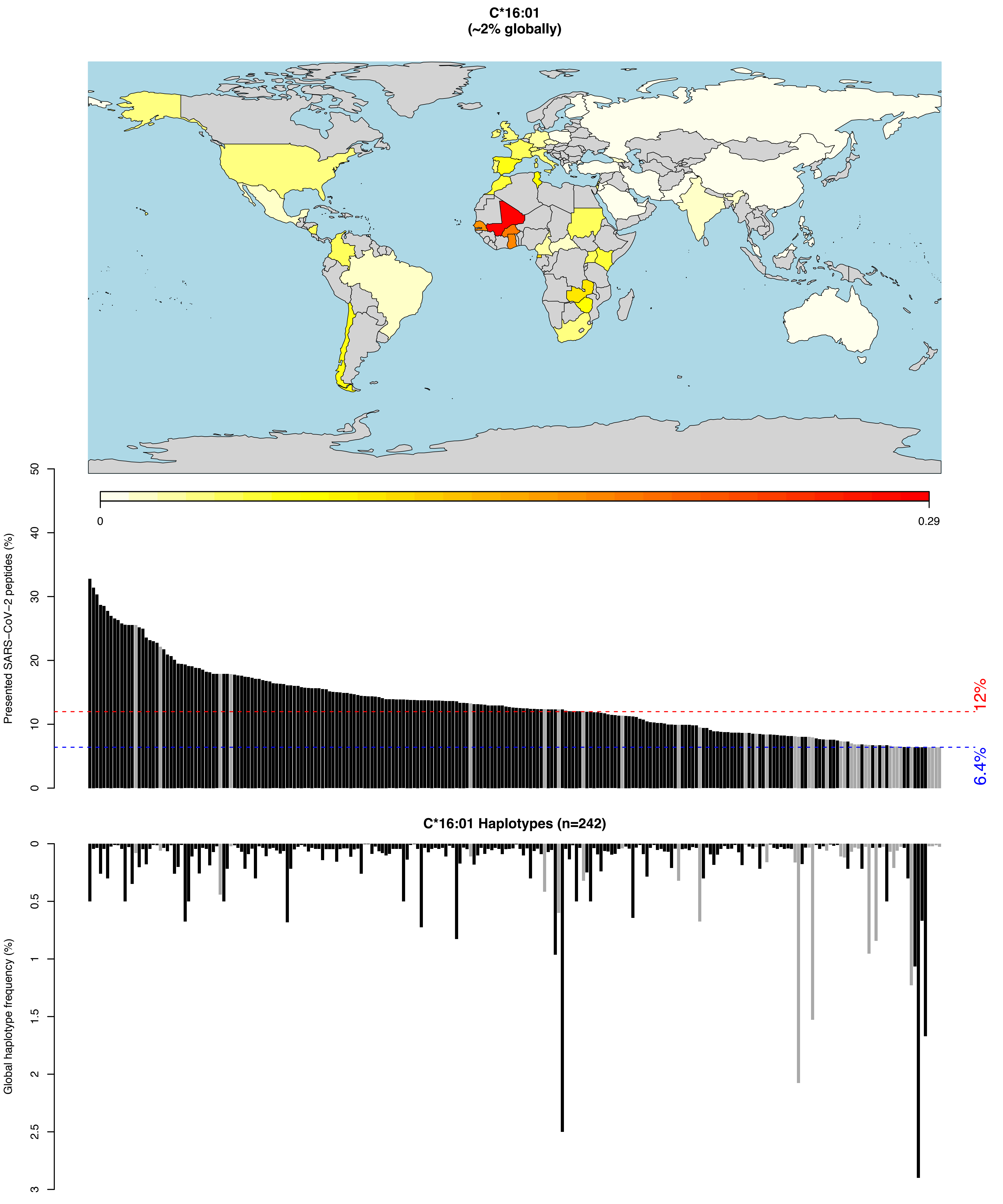

C*16:01
(~2% globally)
0
0.29
Presented SARS−CoV−2 peptides (%)
50
40
30
20
10
0
12%
6.4%
C*16:01 Haplotypes (n=242)
Global haplotype frequency (%)
0
0.5
1
1.5
2
2.5
3

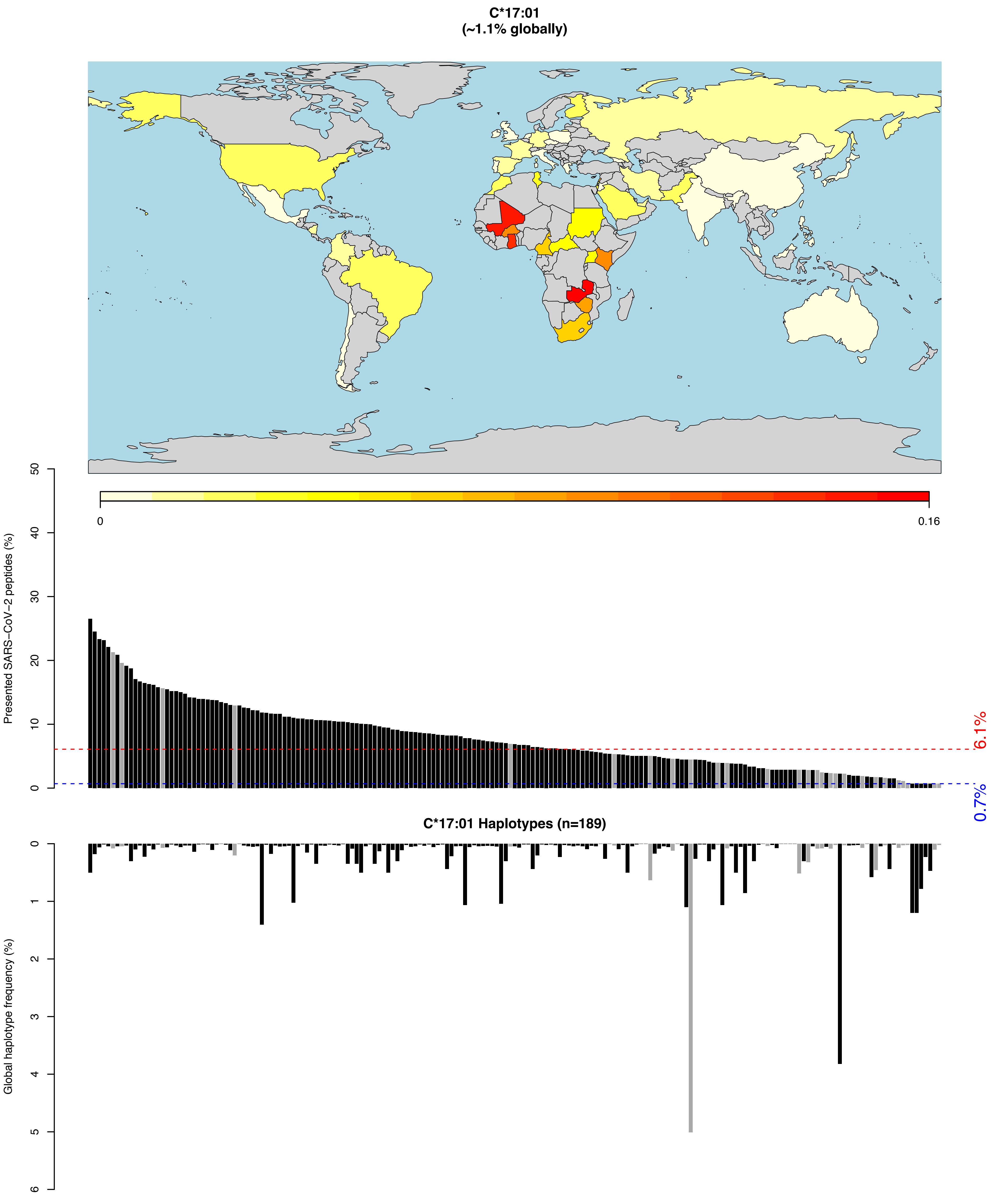
C*17:01
(~1.1% globally)
0
0.16
Presented SARS−CoV−2 peptides (%)
50
40
30
20
10
0
6.1%
0.7%
C*17:01 Haplotypes (n=189)
Global haplotype frequency (%)
0
1
2
3
4
5
6

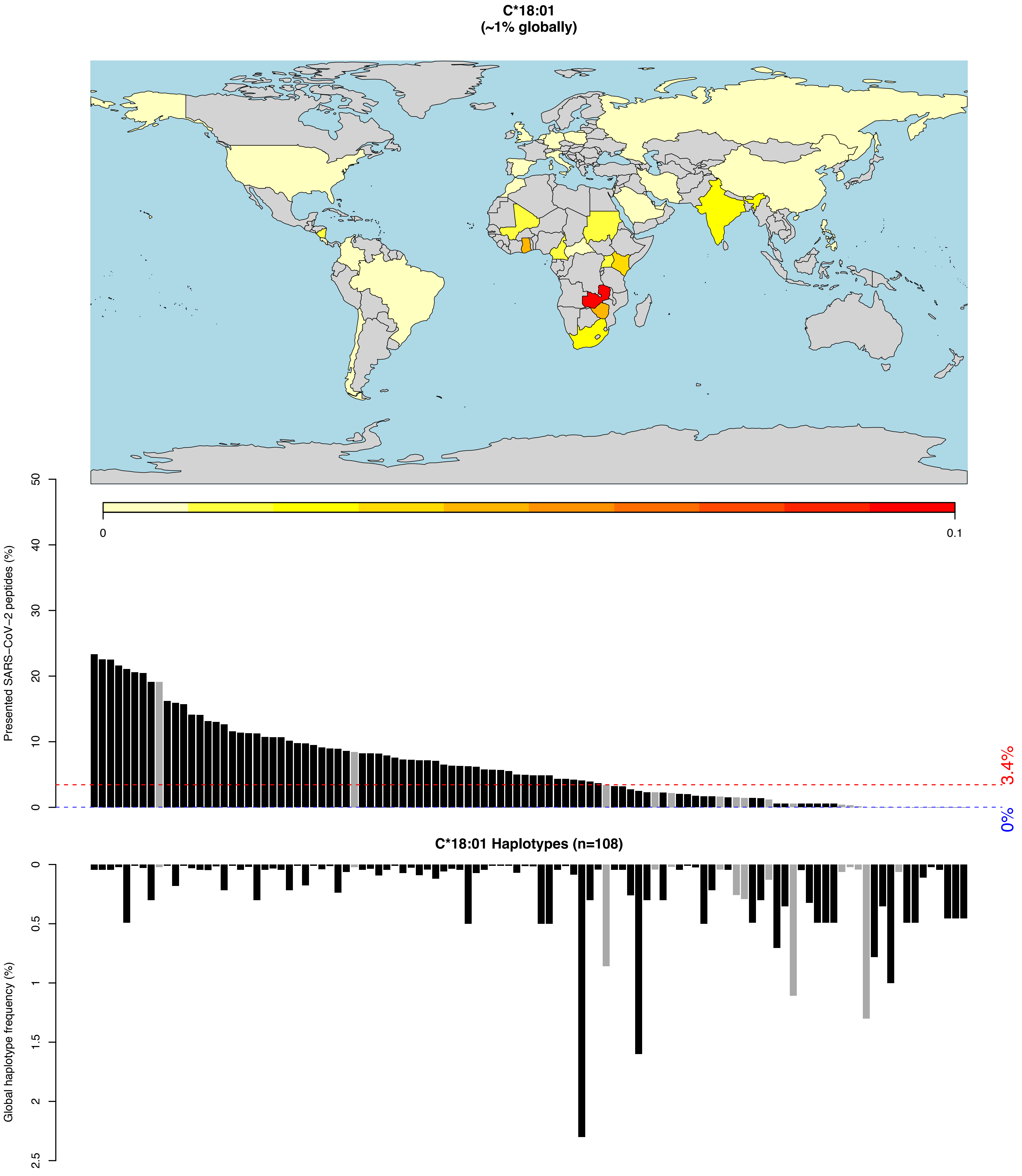

C*18:01
(~1% globally)
0
0.1
Presented SARS−CoV−2 peptides (%)
0
10
20
30
40
50
3.4%
0%
C*18:01 Haplotypes (n=108)
Global haplotype frequency (%)
0
0.5
1
1.5
2
2.5
