# Supplementary material for: Human Leukocyte Antigen Susceptibility Map for Severe Acute Respiratory Syndrome Coronavirus 2
Source: J Virol. 2020 Jun 16;94(13):e00510-20. doi: 10.1128/JVI.00510-20 (PMC7307149; doi:10.1128/JVI.00510-20)
Supplement: Supplemental file 4 [file JVI.00510-20-s0004.pdf]

SUPPLEMENTARY DATA

Supplemental Data 1 contains graphical depictions of sequence alignments for 5 protein sequences (ORF1ab polyprotein, spike, envelope, membrane, and nucleocapsid) across 34 diverse coronavirus proteomes (including all 7 human coronaviruses). Supplemental Data 2 contains a series of world maps displaying the estimated global distributions and frequencies across 145 different HLA alleles, as well as the distribution of predicted SARS-CoV-2 peptide binding and global haplotype frequencies across all haplotypes containing that HLA allele. The full list of SARS-CoV-2 8- to 12-mers with their individual netMHCpan v4.0 predicted binding affinities across all 145 HLA alleles is available at https://github.com/pdxgx/covid19/blob/master/supporting_data/Appendix_4.zip.

SUPPLEMENTARY FIGURES

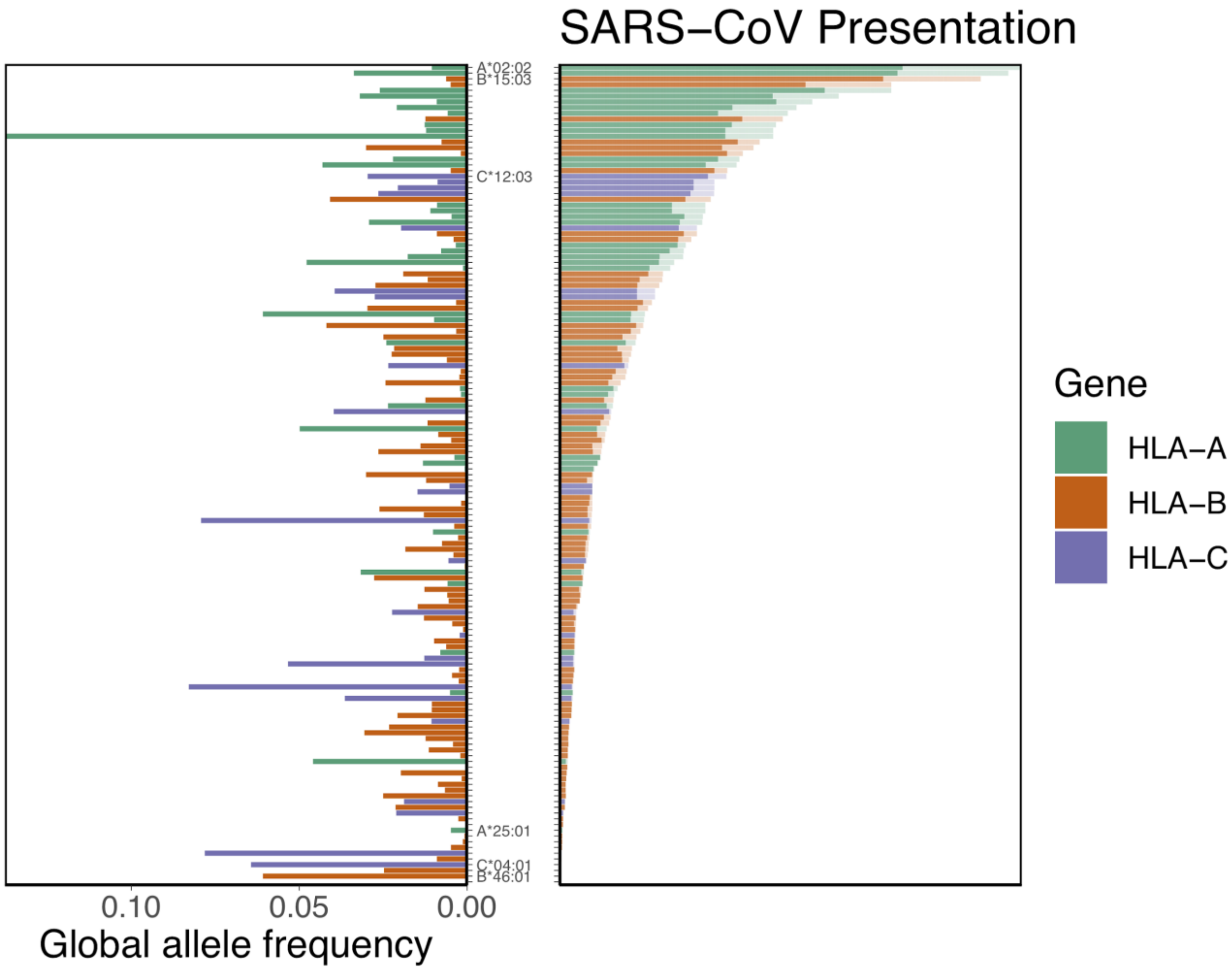

Supplementary Figure S1: Distribution of HLA allelic presentation of 8-12mers from the SARS-CoV proteome (see Supplementary Table S6). At right, the number of peptides that putatively bind to each of 145 HLA alleles is shown as a series of horizontal bars, with dark and light shading indicating the number of tightly (<50nM) and loosely (<500nM) binding peptides respectively, and with green, orange, and purple colors representing HLA-A, -B, and -C alleles, respectively. Alleles are sorted in descending order based on the number of peptides they bind (<500nM). The corresponding estimated allelic frequency in the global population is also shown (to left), with length of horizontal bar indicating absolute frequency in the population.

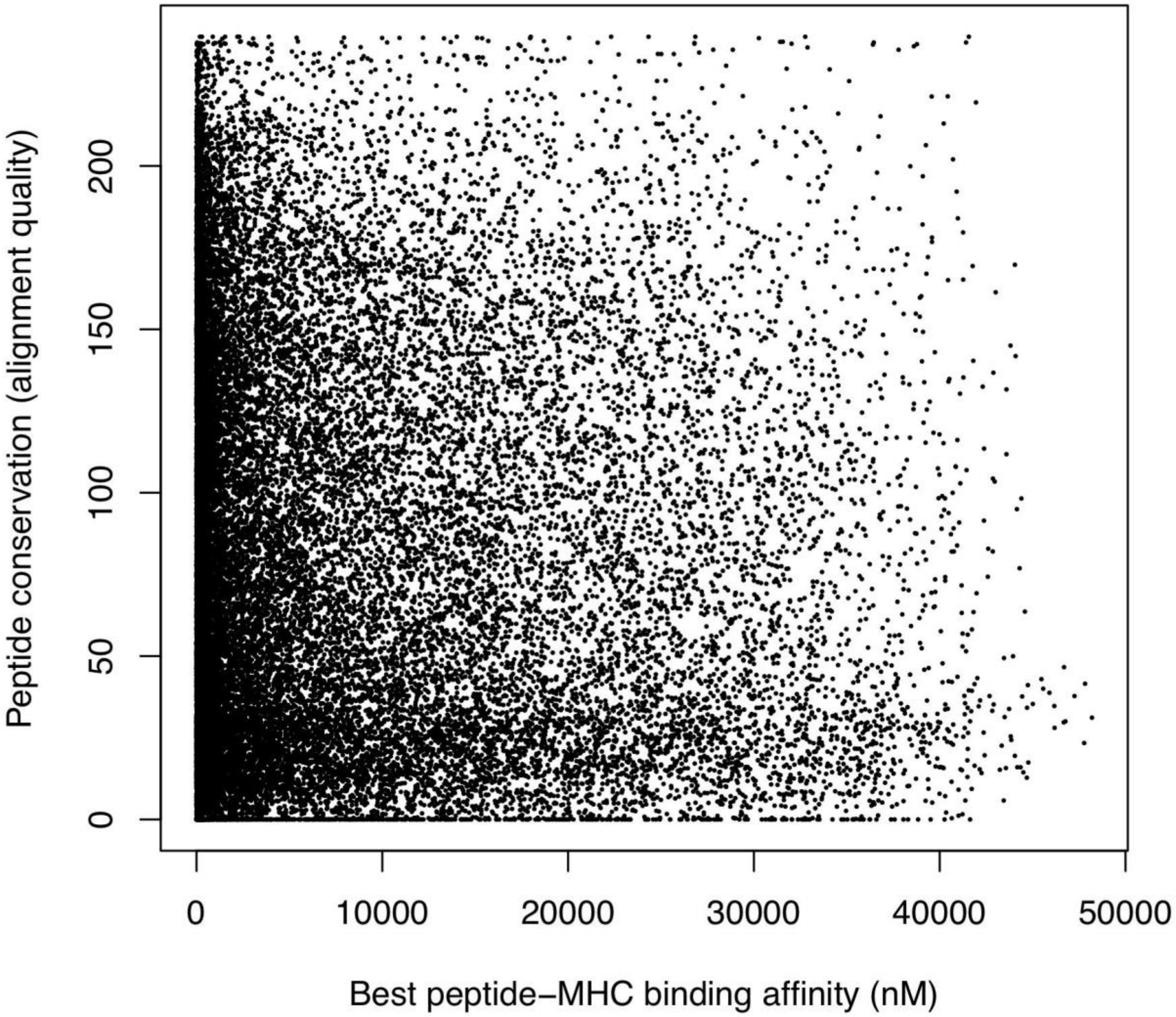

Supplementary Figure S2: Relationship between predicted peptide-MHC binding affinity and peptide conservation across coronaviruses. Every point represents a single unique peptide covering, together, the entirety of the SARS-CoV-2 proteome. The best predicted MHC binding affinity scores across 145 different HLA alleles are shown for each peptide along the x-axis. Sequence conservation (Clustal Omega alignment score) is shown for each peptide along the y-axis.

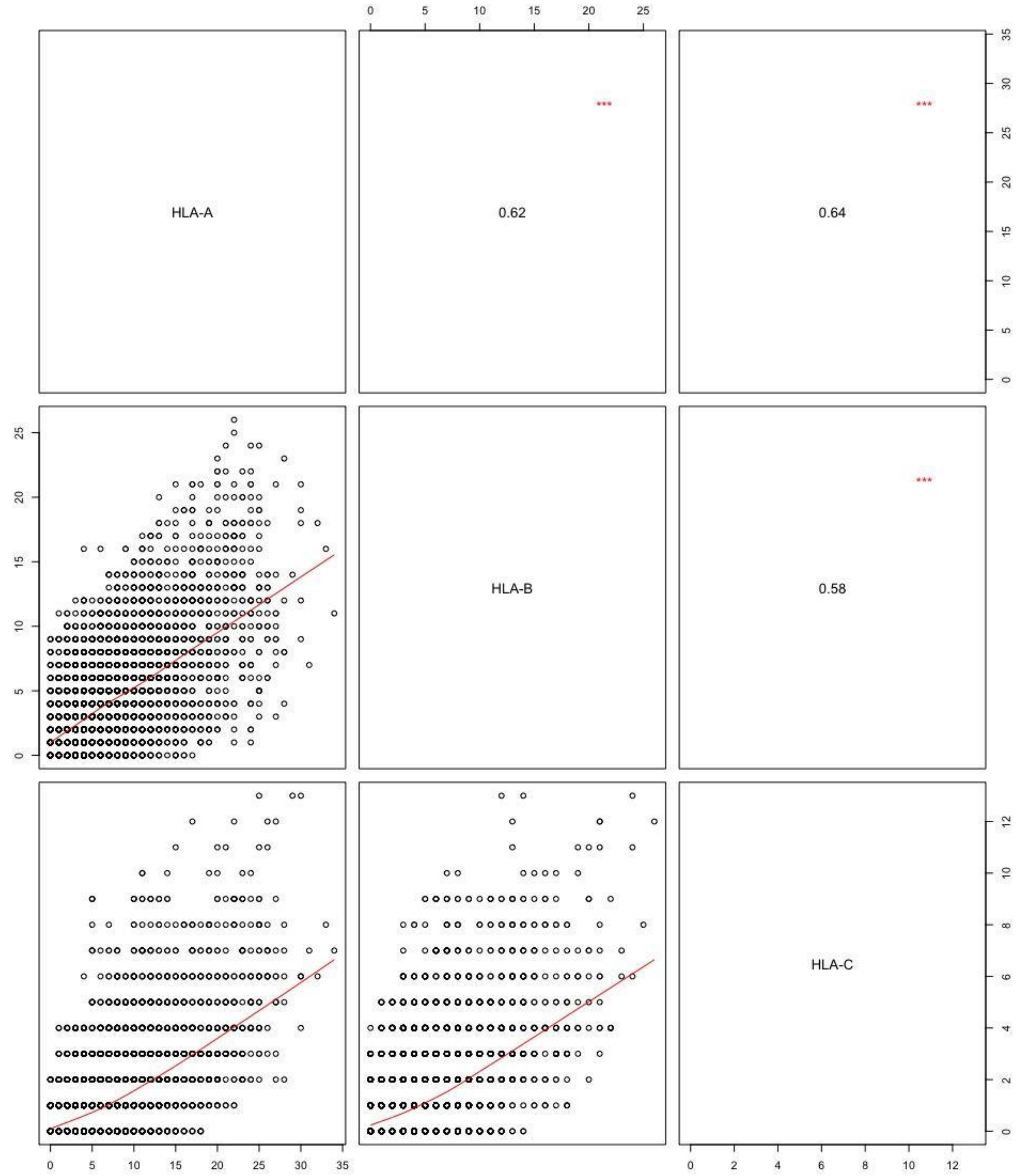

Supplementary Figure S3: Pairwise relationship of peptide presentation between HLA-A, -B, and -C. In the bottom left three panels, every point represents the pairwise comparison of the number of peptide-allele interactions for all position coordinates. Taken together, the position

*coordinates cover the entirety of the SARS-CoV-2 proteome. The top right three panels show the quantitative correlation scores between each pair of HLA type comparisons (*** indicates statistical significance).*

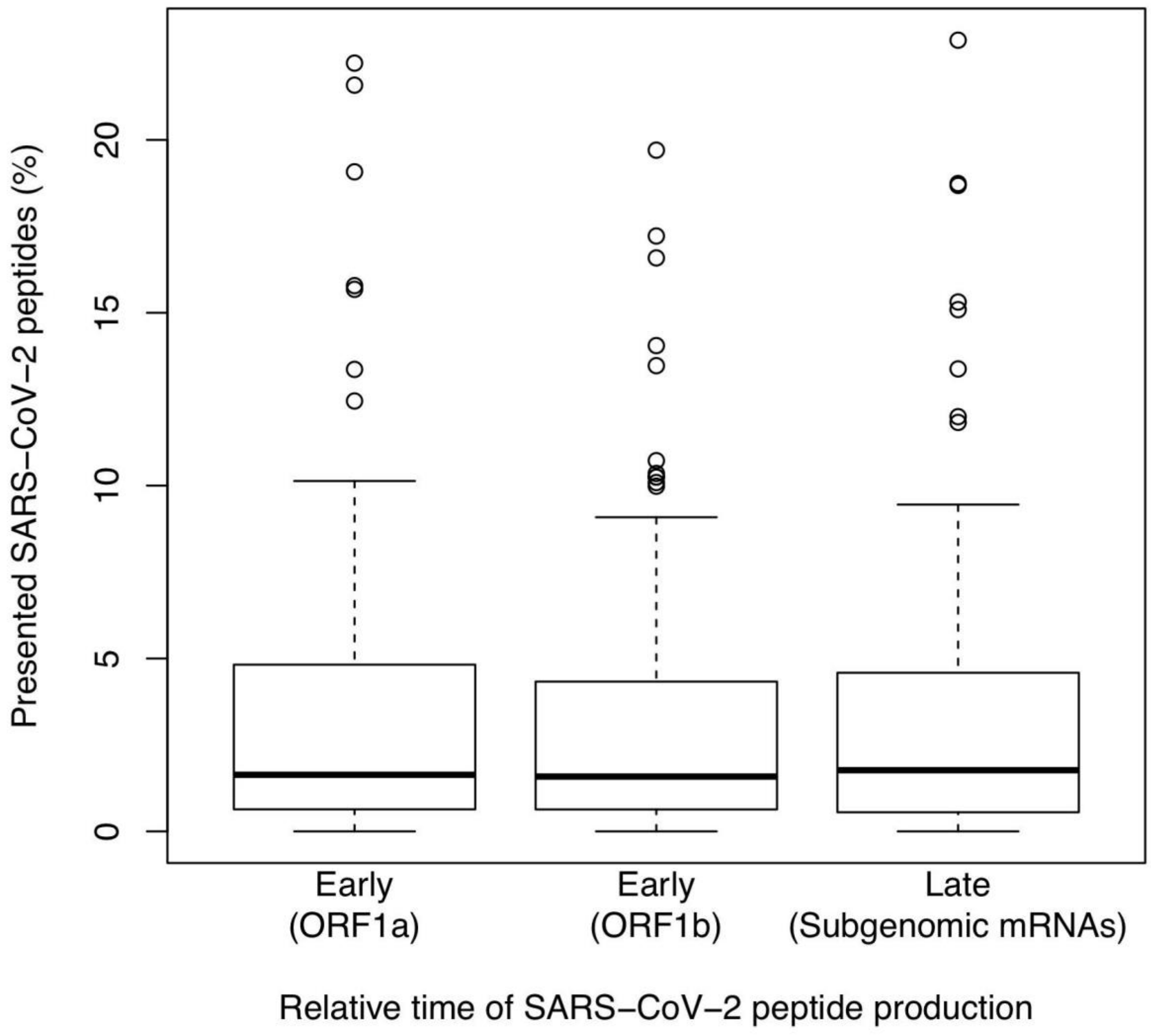

Supplementary Figure S4: Boxplot distributions of estimated epitope presentation across 145 HLA alleles for early and late SARS-CoV-2 peptides. Capacity for peptide presentation is shown

along the y-axis for 145 distinct HLA alleles, for three non-overlapping sets of peptides produced at different timepoints in the viral life cycle as indicated (x-axis). Y-axis percentiles are calculated as the number of peptides from the indicated compartment of the SARS-CoV-2 proteome divided by the total number of presentable 8- to 12-mer peptides from that compartment of the proteome. Dark black lines represent median values, with boxes indicating the 25% and 75% quantiles, with whiskers representing the 25% and 75% quantiles minus or plus the interquartile range, respectively, and with additional outliers shown as open circles.

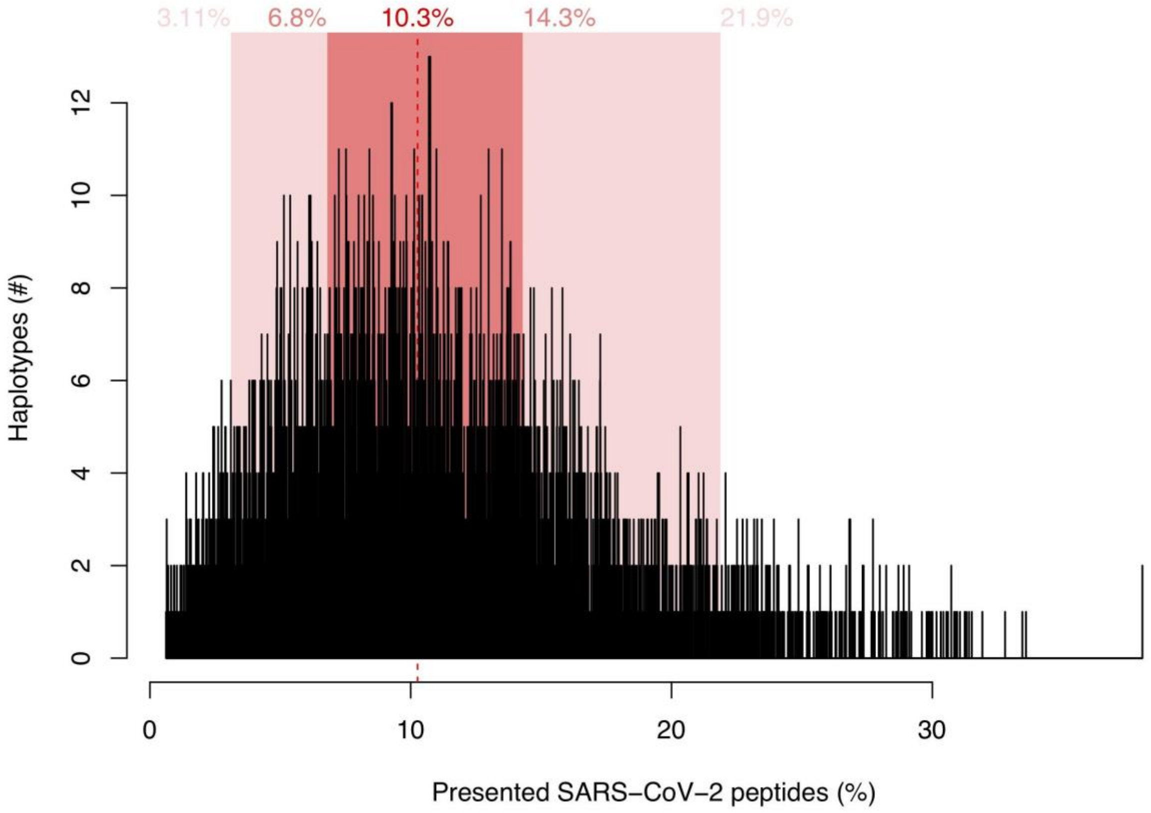

Supplementary Figure S5: Histogram of SARS-CoV-2 peptide presentation for 5,905 distinct HLA-A/B/C haplotypes. Number of haplotypes are counted along the y-axis, corresponding to their individual capacity (aggregated across all their three component HLA types) to present peptides from the SARS-CoV-2 proteome, shown along the x-axis (percentile is calculated as number of unique peptides presented divided by the total number of presentable 8- to 12-mer peptides from the SARS-CoV-2 proteome). Dashed red line corresponds to the median presentation capacity, while dark and light pink highlighted regions correspond to the 25/75% and 5/95% quantiles, respectively, with numerical values shown in the upper aspect of the plotting region.

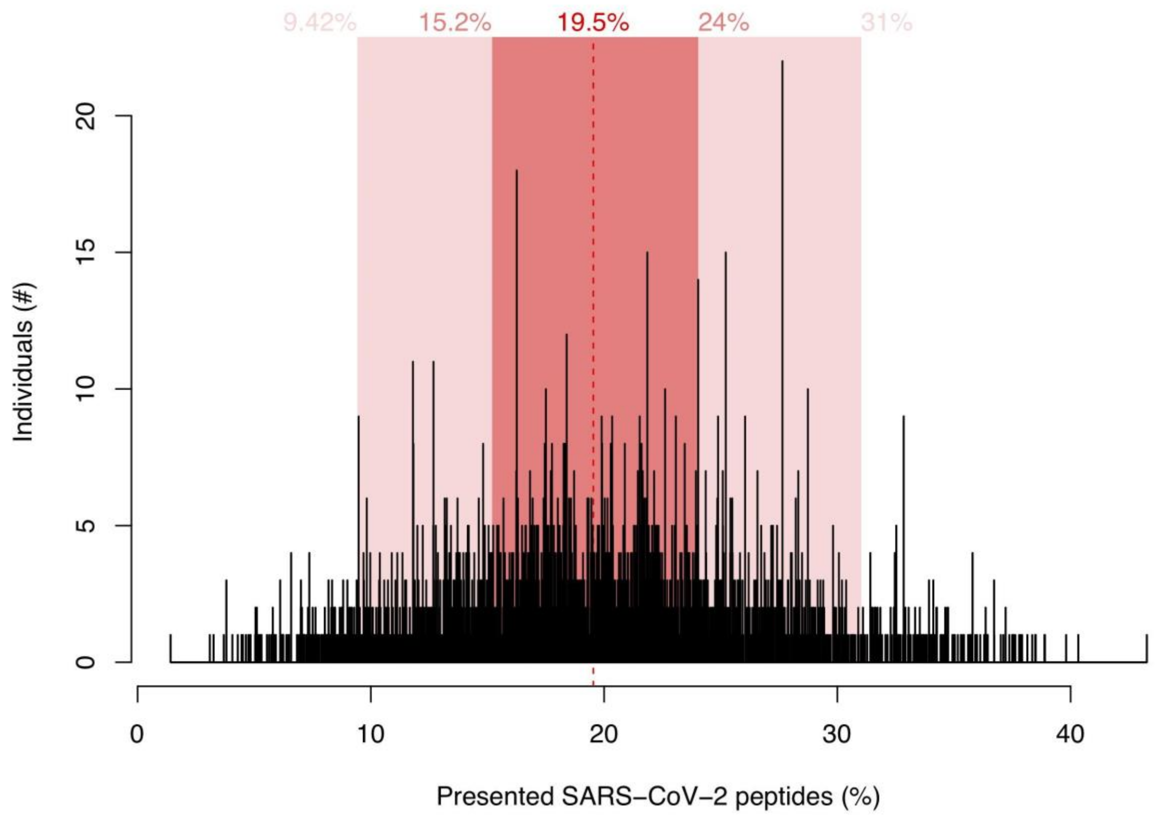

Supplementary Figure S6: Histogram of SARS-CoV-2 peptide presentation for 3,382 individuals' full HLA repertoires. Individuals are counted along the y-axis, corresponding to their individual capacity (aggregated across all 6 of their HLA types) to present peptides from the SARS-CoV-2 proteome, shown along the x-axis (percentile is calculated as number of unique peptides presented divided by the total number of presentable 8- to 12-mer peptides from the SARS-CoV-2 proteome). Dashed red line corresponds to the median presentation capacity, while dark and light pink highlighted regions correspond to the 25/75% and 5/95% quantiles, respectively, with numerical values shown in the upper aspect of the plotting region.

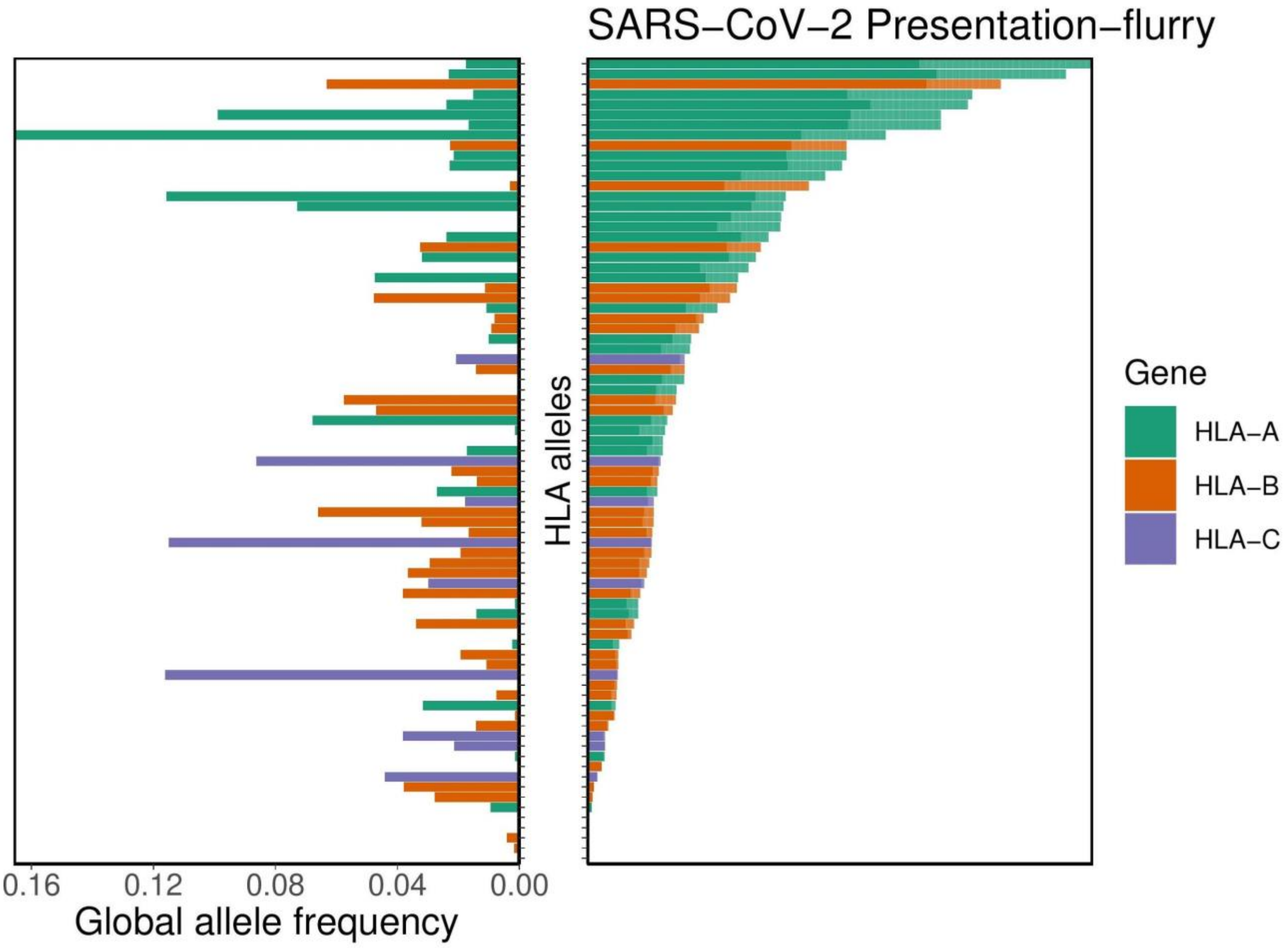

Supplementary Figure S7: Distribution of HLA allelic presentation of 8- to 12-mers from the SARS-CoV-2 proteome using the tool MHCflurry. At right, the number of peptides (see

Supplementary Table S7) that putatively bind to each of 66 HLA alleles is shown as a series of horizontal bars, with dark and light shading indicating the number of tightly (<50nM) and loosely (<500nM) binding peptides respectively, and with green, orange, and purple colors representing HLA-A, -B, and -C alleles, respectively. Alleles are sorted in descending order based on the number of peptides they bind (<500nM). The corresponding estimated allelic frequency in the global population is also shown (to left), with length of horizontal bar indicating absolute frequency in the population.

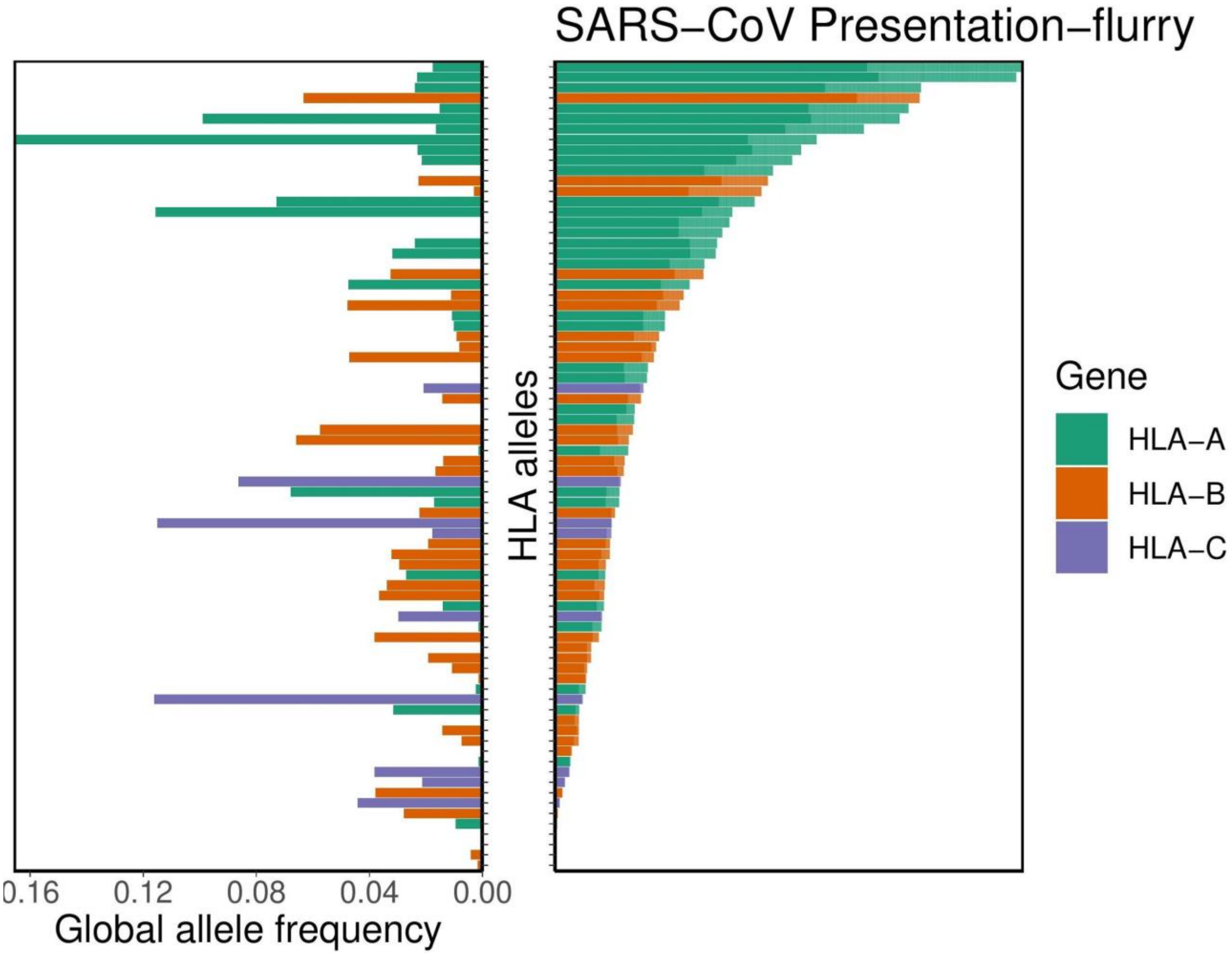

Supplementary Figure S8: Distribution of HLA allelic presentation of 8- to 12-mers from the SARS-CoV proteome using the tool MHCflurry. At right, the number of peptides (see Supplementary Table S8) that putatively bind to each of 66 HLA alleles is shown as a series of

horizontal bars, with dark and light shading indicating the number of tightly (<50nM) and loosely (<500nM) binding peptides respectively, and with green, orange, and purple colors representing HLA-A, -B, and -C alleles, respectively. Alleles are sorted in descending order based on the number of peptides they bind (<500nM). The corresponding estimated allelic frequency in the global population is also shown (to left), with length of horizontal bar indicating absolute frequency in the population.

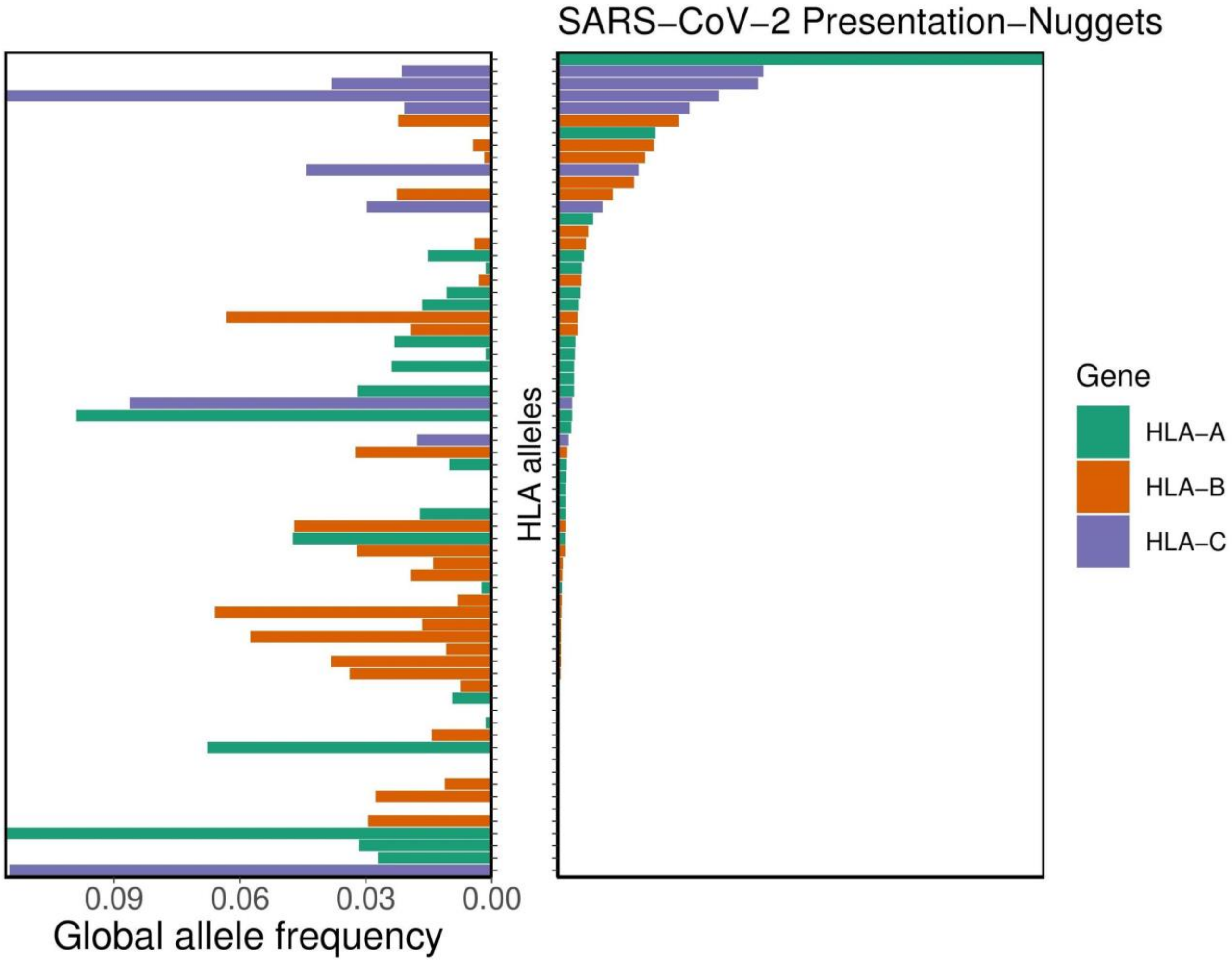

Supplementary Figure S9: Distribution of HLA allelic presentation of 8- to 12-mers from the SARS-CoV-2 proteome using the tool MHCnuggets. At right, the number of peptides (see Supplementary Table S7) that putatively bind to each of 66 HLA alleles is shown as a series of horizontal bars, with dark and light shading indicating the number of tightly (<50nM) and loosely (<500nM) binding peptides respectively, and with green, orange, and purple colors representing HLA-A, -B, and -C alleles, respectively. Alleles are sorted in descending order based on the number of peptides they bind (<500nM). The corresponding estimated allelic frequency in the global population is also shown (to left), with length of horizontal bar indicating absolute frequency in the population.

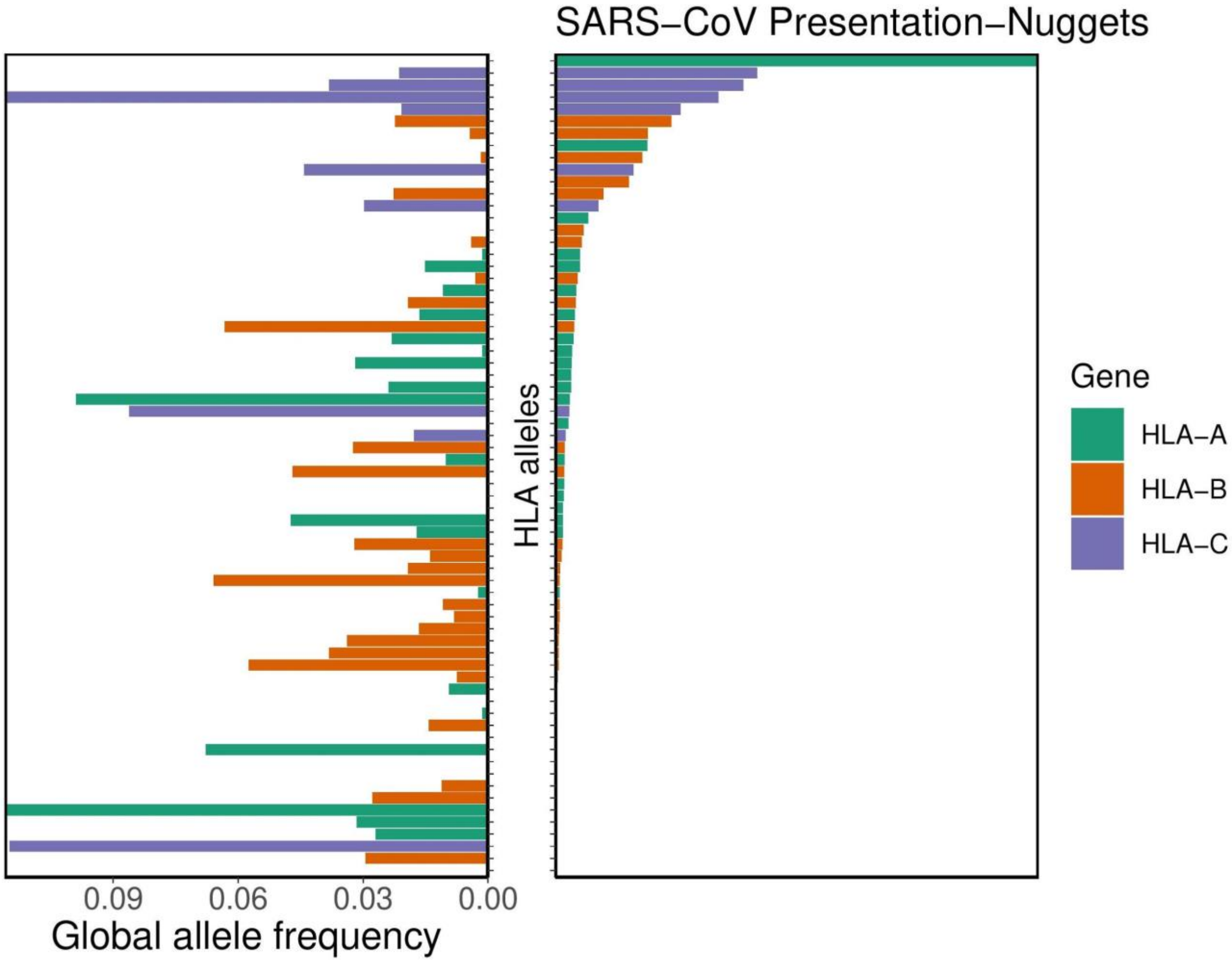

Supplementary Figure S10: Distribution of HLA allelic presentation of 8- to 12-mers from the SARS-CoV proteome using the tool MHCnuggets. At right, the number of peptides (see Supplementary Table S8) that putatively bind to each of 66 HLA alleles is shown as a series of horizontal bars, with dark and light shading indicating the number of tightly (<50nM) and loosely (<500nM) binding peptides respectively, and with green, orange, and purple colors representing HLA-A, -B, and -C alleles, respectively. Alleles are sorted in descending order based on the number of peptides they bind (<500nM). The corresponding estimated allelic frequency in the global population is also shown (to left), with length of horizontal bar indicating absolute frequency in the population.
